# Acridine photocatalysis enables tricomponent direct decarboxylative amine construction†

**DOI:** 10.1039/d4sc02356k

**Published:** 2024-05-22

**Authors:** Xianwei Sui, Hang T. Dang, Arka Porey, Ramon Trevino, Arko Das, Seth O. Fremin, William B. Hughes, William T. Thompson, Shree Krishna Dhakal, Hadi D. Arman, Oleg V. Larionov

**Affiliations:** a Department of Chemistry, The University of Texas at San Antonio One UTSA Circle San Antonio TX 78249 USA oleg.larionov@utsa.edu

## Abstract

Amines are centrally important motifs in medicinal chemistry and biochemistry, and indispensable intermediates and linchpins in organic synthesis. Despite their cross-disciplinary prominence, synthetic access to amine continues to rely on two-electron approaches based on reductions and additions of organometallic reagents, limiting their accessible chemical space and necessitating stepwise preassembly of synthetic precursors. We report herein a homogeneous photocatalytic tricomponent decarboxylative radical-mediated amine construction that enables modular access to α-branched secondary amines directly from the broad and structurally diverse chemical space of carboxylic acids in a tricomponent reaction with aldehydes and aromatic amines. Our studies reveal the key role of acridine photocatalysis acting in concert with copper and Brønsted acid catalytic processes in facilitating the previously inaccessible homogeneous photocatalytic reaction and provide a streamlined segue to a wide range of amines and nonproteinogenic α-amino acids.

## Abstract 2

Diverse amines and amino acids are now readily accessed from carboxylic acids, aldehydes, and amine precursors in a direct decarboxylative reaction enabled by a homogeneous triple catalytic system based on acridine photocatalysis.

## Graphical abstract

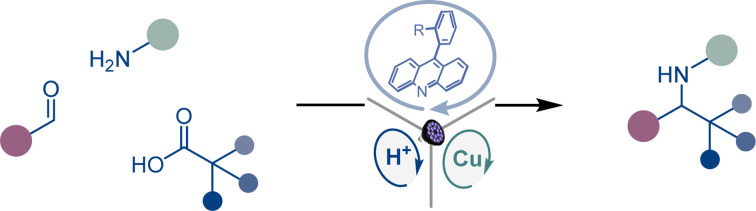

## Introduction

**Fig. 1:**
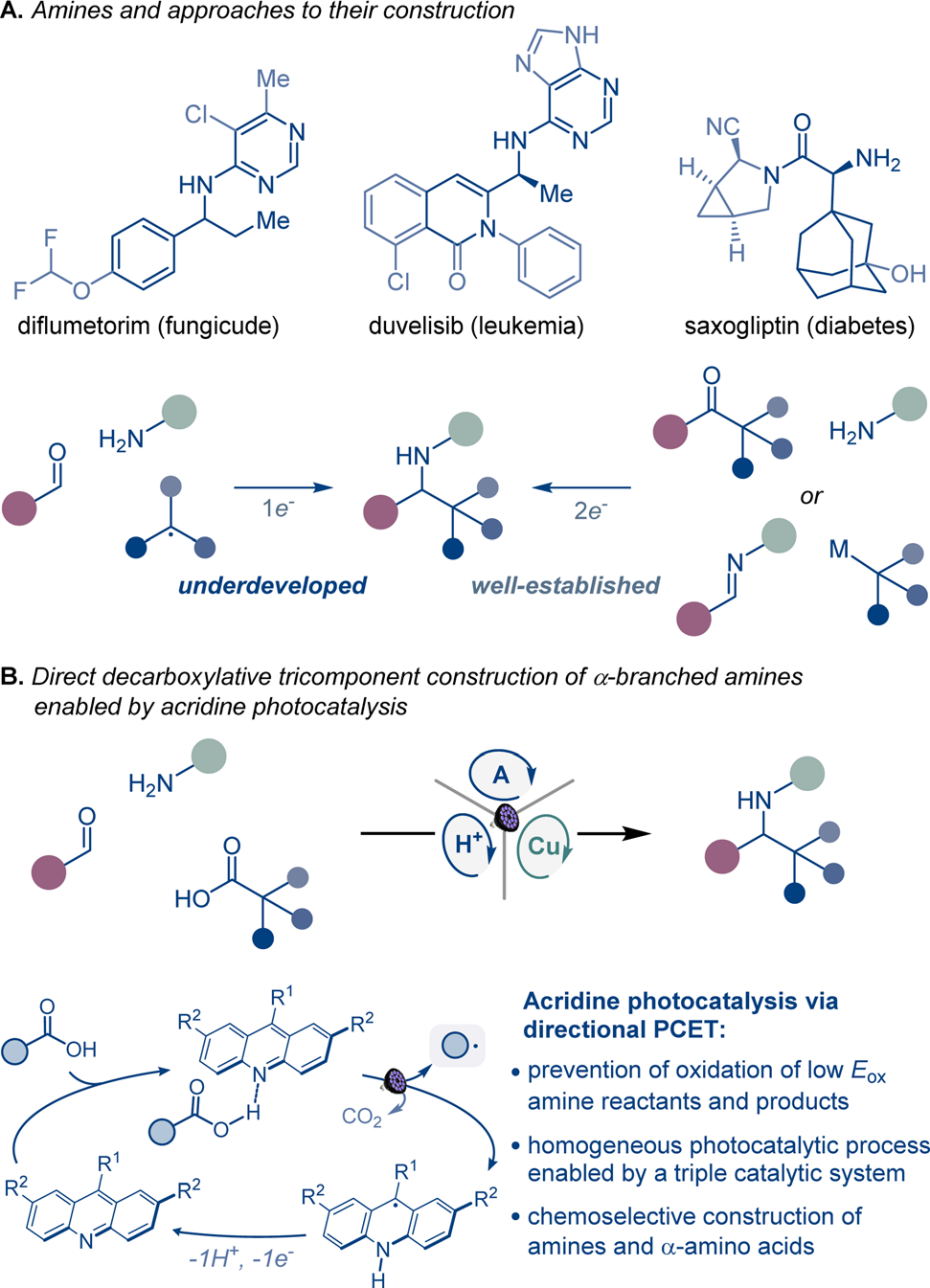
Direct decarboxylative construction of α-branched amines.

Homogeneous photocatalytic systems have recently received significant attention due to their ability to catalyze a broad range of complex synthetic transformations.^1^ Advantageously, homogeneous photocatalysts can effect light-driven reactions at low catalyst loadings and are readily accessible. Furthermore because of their modularity, they can be easily modified, enabling facile reaction optimization. However, homogeneous photocatalytic systems that are based on non-directional single electron transfer are prone to overoxidizing low oxidation potential reactants and products, catalyst deactivation, as well as other radical-based side processes resulting from the high diffusion rates observed in solution phase reactions.^1^ Consequently, the development of chemoselective homogeneous photocatalytic reactions, especially those that involve complex multicomponent and multicatalytic manifolds, remains a significant challenge (Fig. 1).

Continued progress in chemical, biomedical, and materials sciences relies on the development of new synthetic transformations that can provide rapid access to new chemical space and molecular complexity.^2^ α-Branched amines are ubiquitous structural motifs in therapeutic and agricultural agents, natural products, synthetic intermediates, catalysts, and advanced materials, where the conformational features and the proximity of the amino group to other functionalities in the branched chain define the properties and function of the molecule. For example, the π system in α-aromatic amines provides a combination of conformational rigidity and multivector noncovalent interactions with the aromatic ring and the basic nitrogen atom, facilitating molecular recognition and binding that are key to their applications in medicinal chemistry and catalysis.^3^ Likewise, nonproteinogenic α-amino acids are widely used to improve stability, potency, permeability, and oral bioavailability of peptide therapeutics,^4^ while sterically encumbered amino acids bearing tertiary alkyl groups in the side chain are key structural elements of catalysts and ligands.^5^

Despite their cross-disciplinary importance, synthetic access to α-branched amines continues to rely on reductive amination or additions of organometallic reagents that necessitate preassembly of the ketone and imine precursors or may be incompatible with base-sensitive functionalities.^6^ By contrast, modular approaches that enable the formation of the imine intermediate and the ensuing formation of the C–N bond in the same reaction can significantly streamline access to α-branched amines. Critically, the success of this approach hinges on the mutual compatibility of both processes, posing a challenge to the reaction design. However, recent examples of radical-mediated modular tricomponent α-branched amine construction by combining aldehydes and amines with alkyl halides, alkyldihydropyridines, and trifluoroborates point to the feasibility of the approach.^7^

Carboxylic acids are among some of the most abundant and structurally diverse feedstocks that are produced by the chemical industry, as well as derived from natural products, and commonly used as synthetic intermediates. A direct tricomponent decarboxylative reaction of carboxylic acids with aldehydes and amines effected by a homogeneous photocatalytic system could provide a modular synthetic shortcut that will expand the accessible chemical space of α-branched amines, leveraging the broad span of the chemical space of carboxylic acids across the domains of molecular complexity,^8^ fraction of sp^3^ carbon atoms (Fsp^3^),^9^ and geometric diversity.^10,11^

A broad-scope homogeneous photocatalytic reaction of this type would obviate the stepwise preassembly of imine precursors and the use of *N*-sulfonylimine substrates, as well preactivation of carboxylic acids that is typically required to circumvent the challenging oxidative cleavage of the carboxylic group, whose high oxidation potential renders the process incompatible with amines and other easily oxidizable functionalities under homogeneous photocatalytic conditions.^12^ Given the complexity of the process and the potential for overoxidation of easily oxidizable substrates, a homogeneous photocatalytic system that can enable the transformation has remained elusive, and only single example with a heterogeneous superstoichiometric inorganic semiconductor has been described.^13^ As a result, the scope of the direct decarboxylative tricomponent amine construction remains limited and does not permit construction of synthetically important nitrogenous compounds, for example nonproteinogenic α-amino acids.^14^ Additionally, the development of a homogeneous photocatalytic system that efficiently mediates direct decarboxylative tricomponent amine construction will provide insight into mechanisms of multicomponent photocatalytic processes and facilitate the design of other currently unavailable chemoselective multicomponent transformations.

Acridine photocatalysis has recently emerged as a new catalytic platform for direct decarboxylative functionalization that has demonstrated broad scope and compatibility with a variety of easily oxidizable functionalities.^15–17^ The chemoselectivity of the acridine-catalyzed direct decarboxylation is due to the directional character of the decarboxylative process within the acridine–carboxylic acid complex that undergoes photoinduced proton-coupled electron transfer (PCET). Despite the progress, the scope of the functionalizations that can be facilitated by acridine photocatalysis remains ill-defined.

We report herein the development of acridine-catalyzed decarboxylative tricomponent construction of α-branched amines directly from carboxylic acids, aldehydes, and aromatic amines that provides a pipeline to the broad chemical space of carboxylic acids.^18^ The modular synthesis is enabled by a choreographed interplay of the acridine photocatalysis with copper and Brønsted acid catalytic cycles. The reaction provides direct access to a wide range of α-branched amines including an array of nonproteinogenic α-amino acid derivatives. Mechanistic studies point to key roles of each of the three intertwined catalytic cycles in enabling the modular construction of α-branched amines that obviates preassembly and preactivation of amines and carboxylic acids.

## Results and discussion

**Fig. 2:**
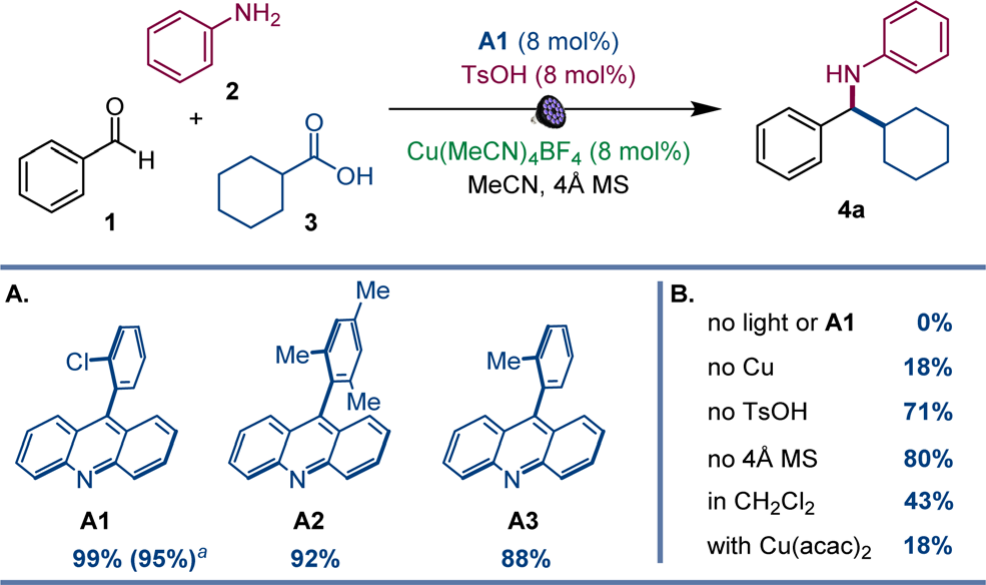
Reaction conditions for the photocatalytic direct decarboxylative tricomponent amine construction: aldehyde 1 (0.2 mmol), aniline 2 (0.24 mmol), carboxylic acid 3 (0.2 mmol), acridine A1 (8 mol%), Cu(MeCN)_4_BF_4_ (8 mol%), TsOH (8 mol%), MeCN (2 mL), 4 Å molecular sieves (60 mg), LED light (400 nm), 30 h. Yield was determined by ^1^H NMR spectroscopy with 1,3,5-trimethoxybenzene as an internal standard. ^*a*^Isolated yield.

After initial optimization studies, we found that a reaction of aldehyde 1, aniline 2, and carboxylic acid 3 efficiently produced amine 4a with acridine catalyst A1, as well as copper(i) and sulfonic acid co-catalysts under 400 nm LED irradiation (Fig. 2). Structurally related acridines A2 and A3 were also competent, albeit slightly less effective catalysts (Fig. 2A), and the reaction required light and the acridine catalyst to proceed (Fig. 2B). The copper(i) catalyst was also essential for the reaction, and a significantly lower yield was observed in the absence of the sulfonic acid. A lower yield was also observed without molecular sieves. Likewise, the reaction was less efficient in other solvents and with other copper co-catalysts (Fig. 2B). Significantly, no amine formation was observed with other types of photocatalysts, *e.g.*, Ir- and Ru-based photocatalysts, 4CzIPN, *N*-phenyl 9-mesitylacridinium salts, and eosin Y (Table S1†).

**Scheme 1:**
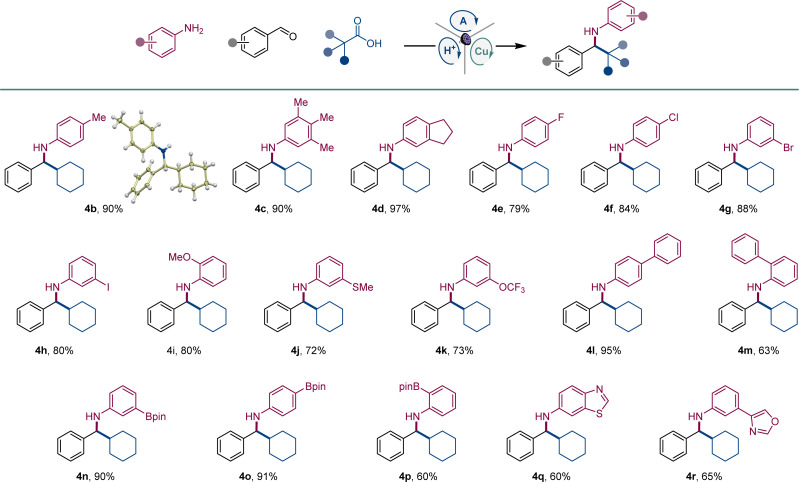
Scope of anilines in the direct decarboxylative tricomponent amine construction. Reaction conditions: aldehyde 1 (0.2 mmol), aniline 2 (0.24 mmol), carboxylic acid 3 (0.2 mmol), acridine A1 (8 mol%), Cu(MeCN)_4_BF_4_ (8 mol%), TsOH (8 mol%), MeCN (2 mL), 4 Å molecular sieves (60 mg), LED light (400 nm), 30 h.

The scope of aromatic amines was examined next in a reaction with aldehyde 2 and carboxylic acid 3 (Scheme 1). Anilines bearing alkyl groups were readily converted to the corresponding amines 4b–4d. A full range of halogen (4e–4h), methoxy (4i), thiomethoxy (4j), and trifluoromethoxy-substituted (4k) anilines were also suitable coupling partners. N-substituted biphenyl amines 4l and 4m were accessed in good yields. Amines 4n–4p bearing a boryl group in the *meta*, *para*, and *ortho* positions were likewise smoothly constructed. Anilines bearing heteroaryl groups also afforded corresponding amines 4q–4r, however, *o*-, *m*-, and *p*-aminopyridines were not compatible with the reaction.

**Scheme 2:**
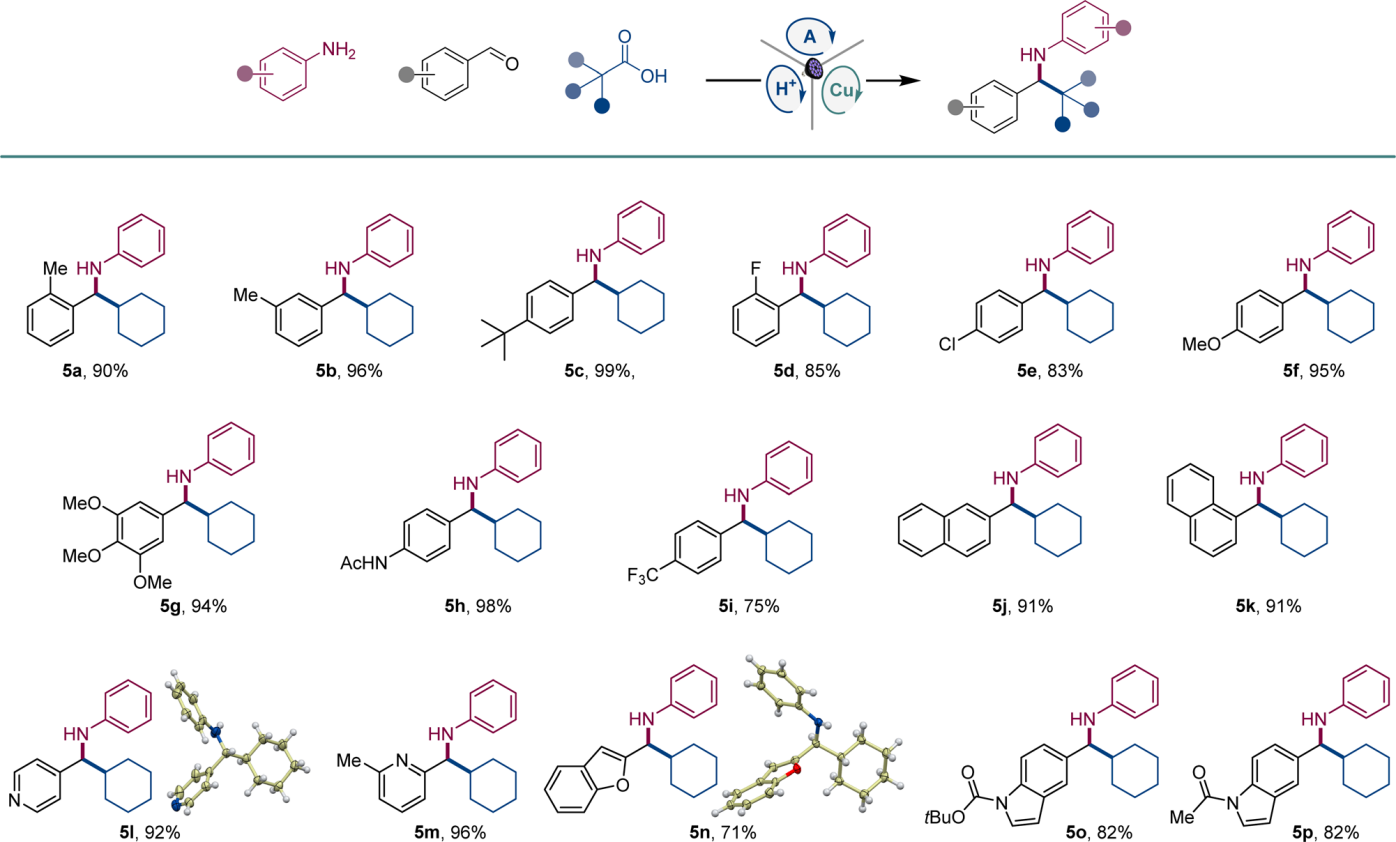
Scope of aldehydes in the direct decarboxylative tricomponent amine construction. Reaction conditions: see Scheme 1.

A range of aromatic aldehydes were examined next (Scheme 2). Aldehydes bearing alkyl (5a–5c), halogen (5d, 5e), and methoxy (5f, 5g) groups in the *meta*, *para*, and *ortho* positions were well tolerated. Amines featuring amide (5h), and trifluoromethyl (5i) groups in the benzylic aryl ring were similarly readily synthesized. Both isomeric naphthaldehyde precursors produced the corresponding amines 5j and 5k in excellent yields. Heteroaromatic aldehydes were also suitable coupling partners, and a range of amines containing pyridine (5l and 5m), benzofuran (5n), and indole (5o, 5p) were efficiently produced in a single step.

**Scheme 3:**
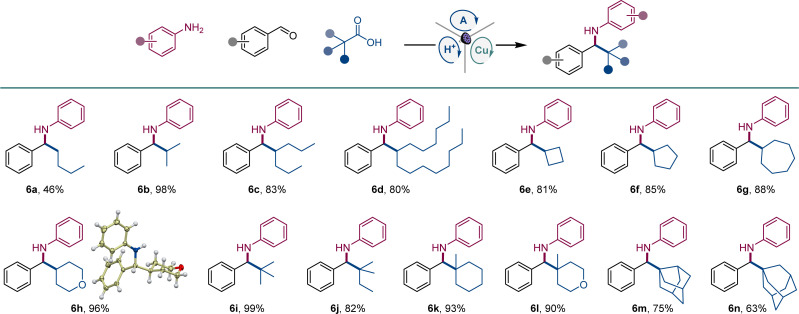
Scope of carboxylic acids in the direct decarboxylative tricomponent amine construction. Reaction conditions: see Scheme 1.

We next explored the scope of carboxylic acids (Scheme 3). An array of primary and secondary acyclic and cyclic carboxylic acids were converted to amines 6a–6h, including amines featuring small-ring and saturated heterocyclic systems (6e, 6h). Likewise, a variety of tertiary carboxylic acids were also suitable reactants, affording amines 6i–6n that bear acyclic, cyclic, and caged topologies in the alkyl moiety.

**Scheme 4:**
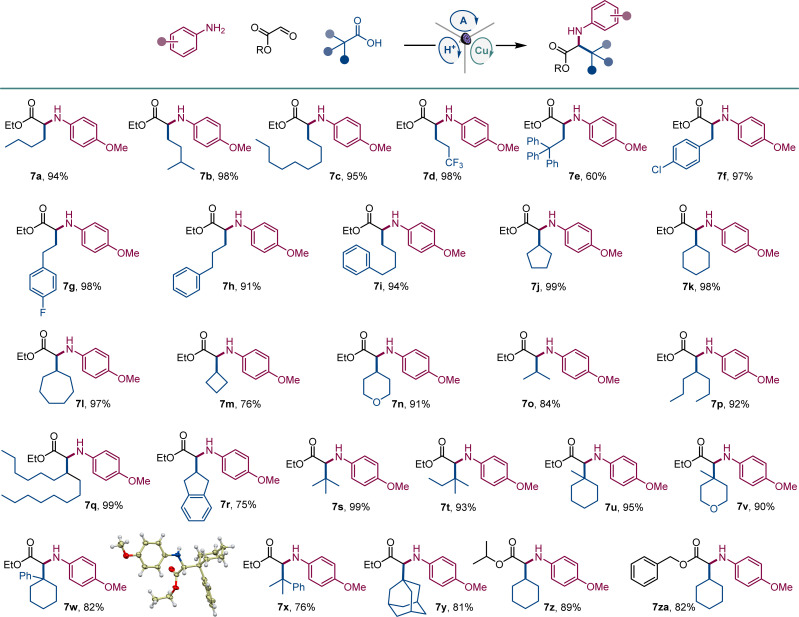
Direct decarboxylative tricomponent construction of α-amino acids. Reaction conditions: see Scheme 1.

Given the cross-disciplinary importance of nonproteinogenic α-amino acids, we examined the tricomponent decarboxylative reaction with glyoxylates as aldehyde components (Scheme 4). *p*-Methoxyaniline (*E*_1/2_ = −0.24 V *vs.* SCE^19^) was selected as an amine to test if easily oxidizable anilines are tolerated in the photocatalytic reaction and because of the well-documented protocols for the removal of the PMP group in the context of α-amino acid synthesis.^20^ Norleucine (7a) and homoleucine (7b), as well as long-chain (7c) and trifluoromethyl-substituted (7d) amino acid derivatives were readily accessed from the corresponding carboxylic acids. Amino acid derivatives 7e–7i bearing aromatic substituents, including sterically encumbered (7e) and halogen containing (7f, 7g) analogues of phenylalanine and homophenylalanine were similarly easily prepared in excellent yields. Likewise, an array of cyclic and acyclic carboxylic acids provided facile segue to amino acid derivatives 7j–7r featuring diverse secondary alkyl side chains. Notably, the reaction can also be used to access sterically hindered amino acids bearing bulky acyclic and cyclic tertiary alkyl groups (7s–7y). The reaction can also be conducted with isopropyl and benzyl glyoxylate, affording corresponding derivatives 7z and 7za in high yields.

**Scheme 5:**
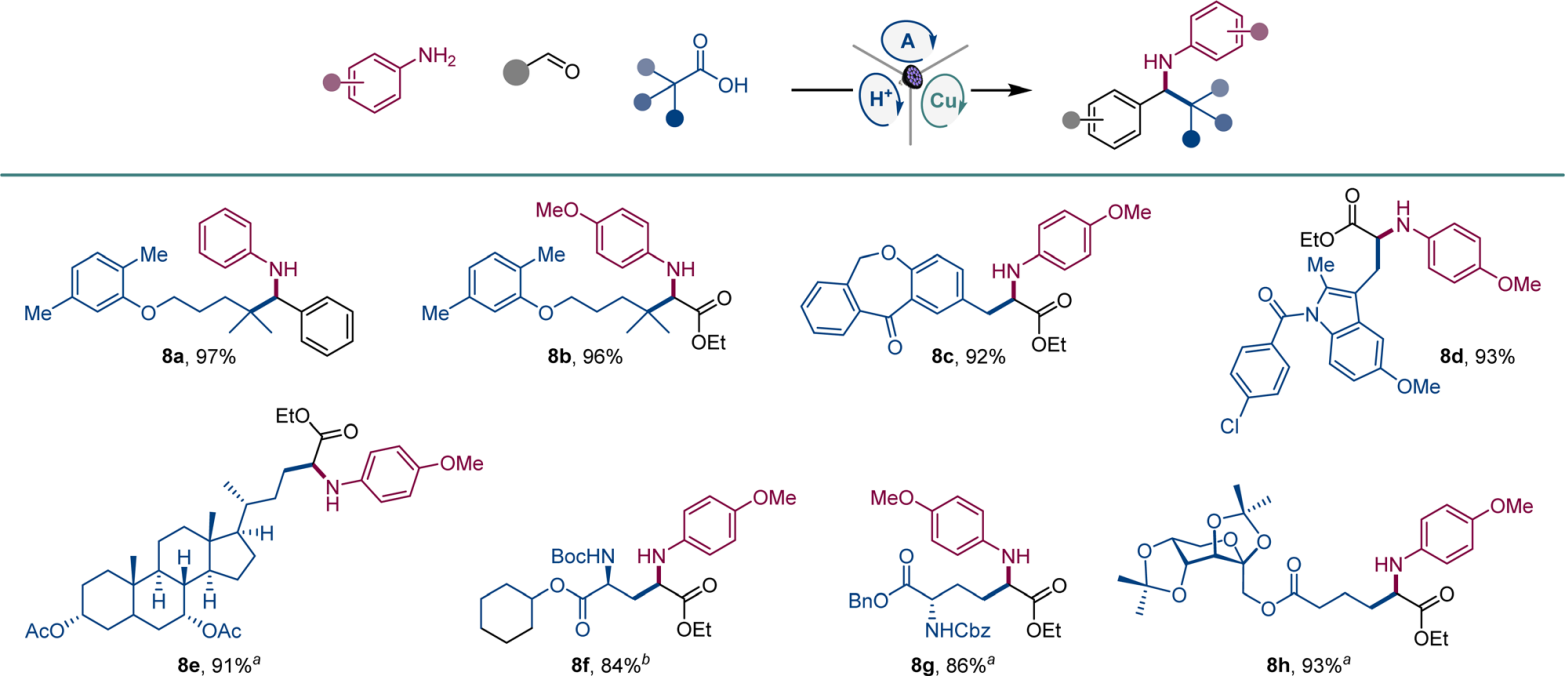
Scope of API and natural product derivatives in the direct decarboxylative tricomponent amine construction. Reaction conditions: see Scheme 1^*a*^1 : 1 dr. ^*b*^2 : 1 dr.

We also examined the scope of the decarboxylative tricomponent amine construction in the more structurally complex settings of active pharmaceutical ingredients (API) and natural products (Scheme 5). Amines derived from the lipid regulator gemfibrozil (8a, 8b), as well as nonsteroidal anti-inflammatory drugs isoxepac (8c) and indomethacin (8d) were readily derived from the corresponding coupling partners. Similarly, chenodeoxycholic acid afforded amino acid derivative 8e. Aspartic and glutamic were efficiently converted to orthogonally protected γ-aminoglutamic (8f) and δ-aminohomoglutamic (8g) acid derivatives.^21^ Amino acid product 8h featuring a carbohydrate-derived side chain was similarly easily constructed.

**Fig. 3:**
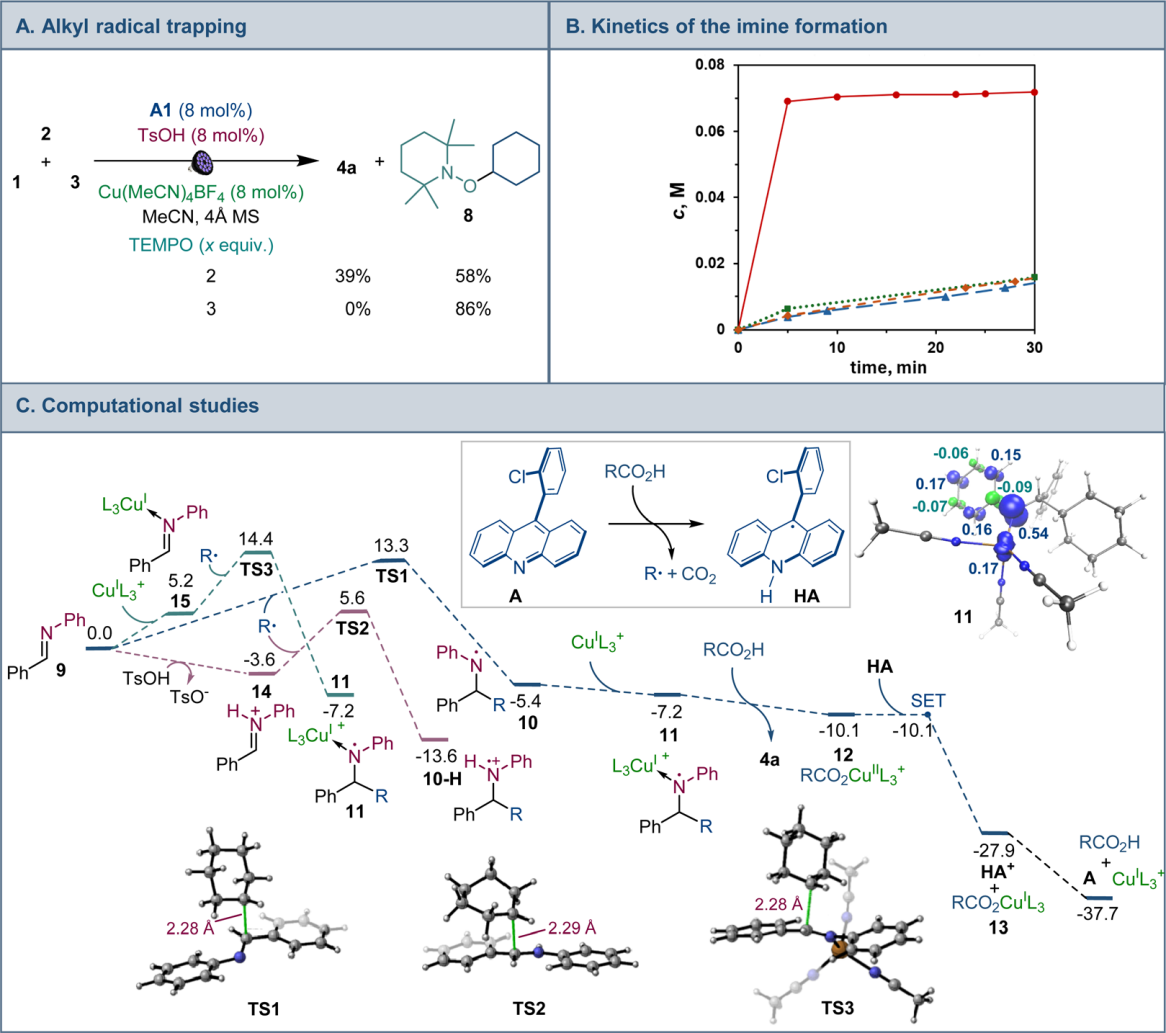
(A) Mechanistic studies of the tricomponent amine construction. (B) Kinetic profile of imine 9 formation. 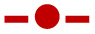 with TsOH (8 mol%); 
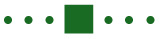
 with acid 3 (1 equiv.); 
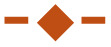
 with Cu(MeCN)_4_BF_4_ (8 mol%); 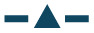 uncatalyzed reaction. (C) Computational studies of the radical amine formation, Δ*G*, kcal mol^−1^, and the spin density isosurface (isovalue = 0.05) for intermediate 11. R = cyclohexyl, L = MeCN.

Mechanistic and computational studies were carried out to gain insight into the roles of the catalysts in enabling the tricomponent amine construction (Fig. 3). Addition of TEMPO resulted in suppression of the reaction and formation of the corresponding product of cross-termination of TEMPO with the alkyl radical intermediate (8, Fig. 3A), pointing to the radical decarboxylative pathway, in line with the reactivity previously observed in other acridine-catalyzed reactions.^11,16,17^

Kinetic studies indicated that the Brønsted acid catalyst significantly accelerates the imine formation, while no catalytic effect was observed with the copper(i) catalyst and stoichiometric carboxylic acid (Fig. 3B), suggesting that the Brønsted acid facilitates the imine formation in the tricomponent process in addition to other potential downstream roles, while the acridine and copper catalysts enable the radical reaction with the imine intermediate.

Computational studies indicated that the alkyl radical addition to imine 9 is kinetically facile and exergonic. The resulting aminyl radical 10 is further intercepted by the copper catalyst, producing formal copper(ii) intermediate 11. Spin density analysis pointed to the noninnocence of the amido ligand and indicated that the aminyl residue retained most of the radical character after binding to copper. This conclusion is supported by effective oxidation state (EOS) analysis, suggesting that intermediate 11 is best described as a copper(i)-aminyl radical coordination complex. This assignment is in line with previous studies of formal high oxidation state complexes of copper.^22^

Subsequent proton-coupled electron transfer with the carboxylic acid produces copper(ii) carboxylate 12 whose oxidation state assignment was confirmed by EOS analysis. The ensuing exergonic and barrierless single electron transfer (SET) between copper complex 12 and acridinyl radical HA affords copper(i) carboxylate 13 and acridinium cation HA^+^ that further regenerate acridine catalyst A and the copper catalyst by an exergonic proton transfer.

**Fig. 4:**
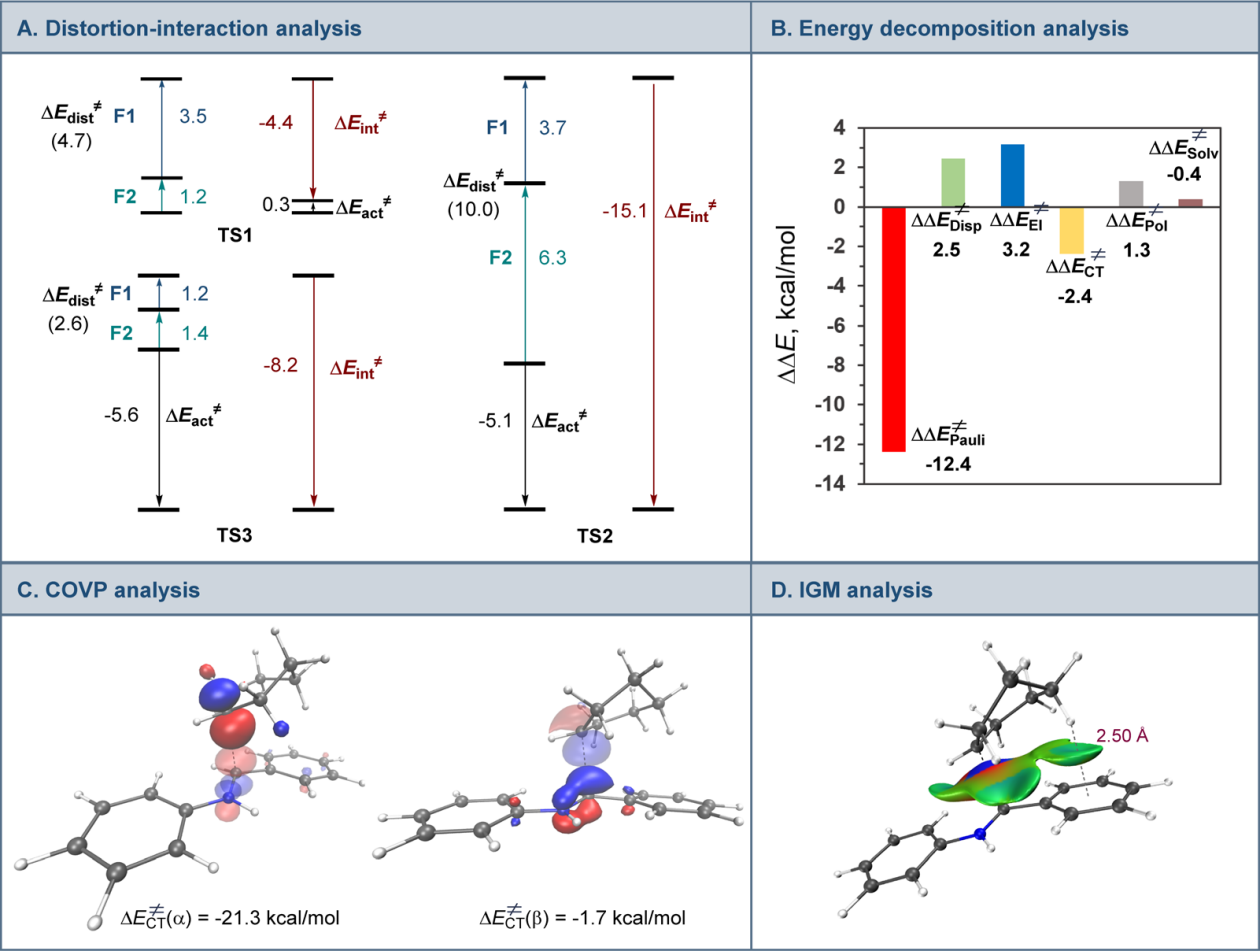
Computational studies of the radical addition to imine 9. (A) Distortion/interaction activation strain model analysis of TS1, TS2, and TS3, kcal mol^−1^. (B) Energy decomposition analysis for TS1 and TS2, ΔΔ*E*^≠^, = Δ*E*_TS2_^≠^ − Δ*E*_TS1_^≠^, kcal mol^−1^. (C) Complementary occupied-virtual pair (COVP) analysis of TS2. The donor orbitals are represented with an opaque surface while acceptor orbitals are represented with a transparent surface. (D) Independent gradient model (IGM) analysis of TS2.

Interestingly, addition of sulfinic acid also results in an acceleration of the radical addition to imine 9*via* a thermodynamically favorable formation of iminium intermediate 14. The subsequent radical addition proceeds over a lower barrier than for the Brønsted acid-free pathway (Δ*G*^≠^ = 5.6 kcal mol^−1^ for TS2*vs.* 13.3 kcal mol^−1^ for TS1). Aminium radical cation 10-H can then be intercepted by the copper catalyst and converted to the amine product by a similar sequence of steps as for aminyl radical 10. By contrast, the radical addition was less kinetically favorable when the Brønsted acid was replaced by the copper(i) catalyst because of the thermodynamically unfavorable formation of copper-bound imine intermediate 15 that could not be compensated by the reduced barrier to radical addition to complex 15. Distortion–interaction activated strain model (ASM) analysis^23^ indicates that both TS2 and TS3 benefit from the substantially stronger interfragment interaction that counterbalances the significantly increased distortion in the case of TS2 (Fig. 4A). Furthermore, energy decomposition analysis based on absolutely localized molecular orbitals (ALMO-EDA)^24^ points to the key role of reduced Pauli (steric) repulsion in TS2 as a consequence of the protonation of the nitrogen atom (Fig. 4B). This observation is congruent with prior studies that pointed to the primary role of reduced Pauli repulsion in the Brønsted acid catalyst-induced acceleration of other reactions.^25^ Additionally, higher charge transfer, describing interfragment orbital interactions also contributed to the stabilization of TS2. Complementary occupied-virtual pair (COVP) analysis of TS2 points to the radical α-SOMO → imine π* pair as the primary contributor to the charge transfer with the imine π → radical β-SOMO interaction playing a smaller role (Fig. 4C). Although dispersion was reduced in TS2, it contributed to the interfragment interactions as demonstrated by independent gradient model (IGM) analysis that points to stabilizing interfragment interactions, *e.g.*, a C–H–π interaction between the alkyl radical and the imine fragment. Taken together, these results indicate that the Brønsted acid catalyst may contribute to both in the imine formation and the radical addition phases of the tricomponent amine construction, while the copper catalyst serves as a linchpin between the acridine-catalyzed decarboxylation and the radical amine formation phases by facilitating proton and electron transfer.

## Conclusions

In conclusion, we have developed a decarboxylative tricomponent construction of α-branched amines directly from carboxylic acids, aldehydes, and aromatic amines that is enabled by acridine photocatalysis acting in concert with copper and Brønsted acid catalytic cycles. The transformation enables modular access to amines directly from the broad chemical space of carboxylic acids and obviates stepwise preassembly of imines, ketones, and activated carboxylic acid derivatives with a homogeneous photocatalytic system. The reaction provides a streamlined segue to a wide range of α-branched amines bearing α-aromatic substituent, as well as nonproteinogenic α-amino acid derivatives. The scope and functional group tolerance of the tricomponent decarboxylative amine construction were demonstrated with a range of functionalized aldehydes, aromatic amines, and carboxylic acids, including the structurally complex settings of active pharmaceutical ingredients and natural products. Mechanistic studies revealed the roles of the three intertwined catalytic cycles in enabling the modular amine synthesis and provided a blueprint for further development of direct decarboxylative multicomponent reactions. Unlike homogeneous photocatalytic systems that are based on non-directional single electron transfer that can lead to unselective oxidation of easily oxidizable reagents and products, the triple catalytic system relies on the directional PCET effected by acridine in concert with the supporting copper- and Brønsted acid catalytic cycles, enabling a previously inaccessible homogeneous photocatalytic process.

## Data availability

All experimental procedures, characterization data, NMR spectra for all new compounds, and details of the computational studies can be found in the ESI.†

## Author contributions

XS, HTD, AP, AD, WTT, and SKD carried out the experiments, and RT, WBH, and SOF performed the computational studies. HDA performed X-ray crystallography studies. OVL directed the project, wrote the manuscript, and co-wrote the ESI.† XS, HTD, AP, AD, SKD, and RT co-wrote the ESI† and contributed to writing the manuscript.

## Conflicts of interest

There are no conflicts to declare.

## Supplementary Material

SC-015-D4SC02356K-s001

SC-015-D4SC02356K-s002
