# Supplementary material for: Acridine photocatalysis enables tricomponent direct decarboxylative amine construction
Source: Chem Sci. 2024 May 22;15(25):9582–90. doi: 10.1039/d4sc02356k (PMC11206229; doi:10.1039/d4sc02356k)
Supplement: SC-015-D4SC02356K-s001 [file SC-015-D4SC02356K-s001.pdf]

Electronic Supplementary Material (ESI) for Chemical Science.
This journal is © The Royal Society of Chemistry 2024

Supporting Information

Acridine Photocatalysis Enables Tricomponent Direct Decarboxylative Amine Construction

Xianwei Sui, Hang T. Dang, Arka Porey, Ramon Trevino, Arko Das, Seth O. Fremin, William B. Hughes, William T. Thompson, Shree Krishna Dhakal, Hadi D. Arman, and Oleg V. Larionov*

Department of Chemistry, The University of Texas at San Antonio, San Antonio, Texas 78249, United States
oleg.larionov@utsa.edu

Contents

Materials and experimental details S1

General procedures S2

Additional experimental studies S2

Table S1 .... S2

Amine products S4

Computational studies S47

X-Ray crystallographic data S74

NMR spectroscopic data S79

References S245

Materials and experimental details

Materials: Acetonitrile was dried over 3Å molecular sieves and thoroughly degassed under the atmosphere of argon before use. Anhydrous *p*-toluenesulfonic acid was prepared by heating the monohydrate under vacuum at 70 °C for 4 h. 4Å Molecular sieves were dried under vacuum at 120 °C for 3 h before use. All other chemicals were used as commercially available.

Experimental equipment: The photoinduced reactions were conducted in borosilicate glass test-tubes (8 mL capacity, Duran) fitted with GL14 screw-caps placed in a test-tube rack on a magnetic stir plate that was flanked by two 400 nm 36W LED lights (λ_{max} = 400 nm, 2.6 mW/cm^2). The temperature in the test-tube rack was maintained at 25–27 °C with an air flow from a compressed air line. Eight parallel reactions arranged in two rows of four tubes were typically carried out in one test-tube rack.

Purification: Column chromatography was performed using CombiFlash Rf-200 (Teledyne-Isco) automated flash chromatography system, as well as manually. Thin layer chromatography was carried out on silica gel-coated glass plates (Merck Kieselgel 60 F254). Plates were visualized under ultraviolet light (254 nm) and using a potassium permanganate stain.

Characterization: ^{1}H, ^{13}C, ^{11}B, and ^{19}F NMR spectra were recorded at 500 MHz (^{1}H), 125 MHz (^{13}C), 202 MHz (^{31}P), 470.5 MHz (^{19}F), and 160.4 MHz (^{11}B) on Bruker AVANCE III 500 instruments in $CDCl_3$ or other specified deuterated solvents with and without tetramethylsilane (TMS) as an internal standard at 25 °C, unless specified

otherwise. Chemical shifts (δ) are reported in parts per million (ppm) from tetramethylsilane (^{1}H and ^{13}C), $BF_3{\cdot}OEt_2$ (^{11}B), and $CFCl_3$ (^{19}F). Coupling constants (*J*) are in Hz. Proton multiplicity is assigned using the following abbreviations: singlet (s), doublet (d), triplet (t), quartet (q), quintet (quint.), septet (sept.), heptet (hept.), multiplet (m), broad (br).

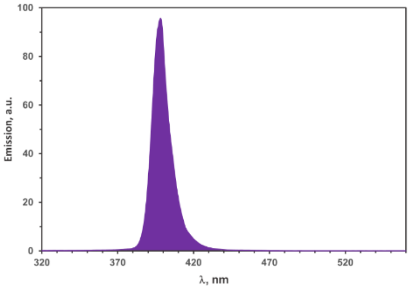

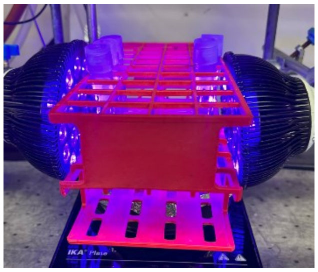

Irradiance was measured with a UVV420 radiometer at a distance of 2 cm from the light source. Infrared measurements were carried out neat on a Bruker Vector 22 FT-IR spectrometer fitted with a Specac diamond attenuated total reflectance (ATR) module.

General procedure for the photo-induced three component amine construction (GP1).

To a 8 mL test-tube equipped with a stir bar, $Cu(MeCN)BF_4$ (5.0 mg, 0.016 mmol, 8 mol%), acridine **A1** (4.7 mg, 0.016 mmol, 8 mol %), 4Å molecular sieves (60 mg), aldehyde (0.2 mmol), aniline (0.24 mmol, 1.2 equiv.), carboxylic acid (0.26 mmol, 1.3 equiv.), and anhydrous *p*-toluenesulfonic acid (2.8 mg, 0.016 mmol, 8 mol%) were added, followed by acetonitrile (2 mL). The test-tube was capped, and the reaction mixture was irradiated with LED light (λ = 400 nm) while stirring at room temperature for 30 h. The reaction mixture was then concentrated under reduced pressure, and the remaining material was purified by flash chromatography on silica gel to give the amine product.

Additional experimental studies

Table S1. Catalyst Performance in the Acridine-Catalyzed Direct Decarboxylative Tricomponent Amine Construction.[a]

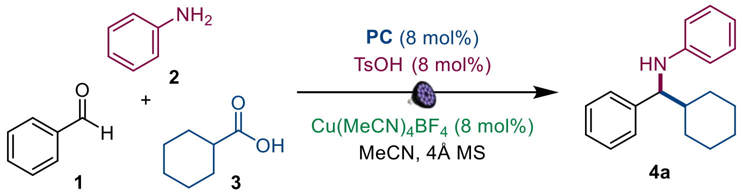

Entry	Photocatalyst	Yield, %
1	Eosin Y at 450 nm	0
2	Eosin Y at 420 nm	0
3	Eosin Y at 400 nm	0
4	Eosin Y disodium salt at 450 nm	0
5	4CzIPN at 450 nm	0
6	4CzIPN at 420 nm	0
7	4CzIPN at 400 nm	0
8	$[Acr\text{-}Mes]^{+}(BF_4)^{-}$ at 400 nm	0
9	$[Acr\text{-}Mes]^{+}(BF_4)^{-}$ at 450 nm	0[b]
10	$Ir(ppy)_3$ at 450 nm	0[b]

Go to table of contents

11	Ir(ppy)$_2$(pq) at 450 nm	0[b]
12	(Ir[dF(CF$_3$)ppy]$_2$(dtbpy))PF$_6$ at 450 nm	0[b]
13	Ru(bpm)$_2$Cl$_2$ at 450 nm	0[b]
14	Ru(*p*-CF$_3$-bpy)$_3$(BF$_4$)$_2$ at 450 nm	0[b]

[a] Reaction conditions: aldehyde **1** (0.2 mmol), aniline **2** (0.24 mmol), carboxylic acid **3** (0.2 mmol), acridine **A1** (8 mol%), Cu(MeCN)$_4$BF$_4$ (8 mol%), TsOH (8 mol%), MeCN (2 mL), 4Å molecular sieves (60 mg), LED light (400 nm), 30 h. Yield was determined by ^{1}H NMR spectroscopy with 1,4-dimethoxybenzene as an internal standard. [b] 2 mol% photocatalyst was used. 1,2,3,5-Tetrakis-(carbazol-9-yl)-4,6-dicyanobenzene, [Acr-Mes]$^+$(BF$_4$)$^-$*:* 10-Phenyl-9-(2,4,6-trimethylphenyl)acridinium tetrafluoro-borate, Ir(ppy)$_3$: Tris(2-phenylpyridine)iridium(III), Ir(ppy)$_2$(pq): bis(2-phenylpyridine)(2-phenyl-qui-noline)iridium(III), (Ir[dF(CF$_3$)ppy]$_2$(dtbpy))PF$_6$: [4,4′-Bis(1,1-dimethylethyl)-2,2′-bipyridine-*N1,N1′*]-bis[3,5-difluoro-2-[5-(trifluoromethyl)-2-pyridinyl-*N*]phenyl-*C*]Iridium(III) hexafluorophosphate, Ru(bpm)$_2$Cl$_2$: Tris(2,2′-bipyrimide)-ruthenium(II) dichloride, Ru(*p*-CF$_3$-bpy)$_3$(BF$_4$)$_2$: Tris(2,2′-(*p*CF$_3$)bi-pyridine)ruthenium(II) tetrafluoroborate.

Radical trapping studies with TEMPO

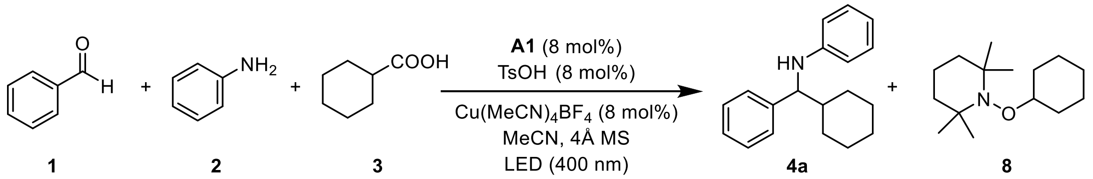

According to GP1, the reaction was carried out with Cu(MeCN)BF$_4$ (5.0 mg, 0.016 mmol, 8 mol%), acridine **A1** (4.7 mg, 0.016 mmol, 8 mol %), 4Å molecular sieves (60 mg), benzaldehyde (21.2 mg, 0.2 mmol), aniline (22.3 mg, 0.24 mmol, 1.2 equiv.), cyclohexanecarboxylic acid (33.3 mg, 0.26 mmol, 1.3 equiv.), anhydrous *p*-toluenesulfonic acid (2.8 mg, 0.016 mmol, 8 mol%), TEMPO (62.5 mg, 0.4 mmol or 93.75 mg, 0.6 mmol) and acetonitrile (2 mL). The test-tube was capped and the reaction mixture was irradiated with LED light (λ = 400 nm) while stirring at at room temperature for 20 h. The reaction mixture was then diluted with ethyl acetate (15 mL), and washed with a saturated solution of EDTA disodium salt (5 mL). The organic layer was collected, dried over anhydrous sodium sulfate, concentrated, and a ^{1}H NMR spectrum was recorded with 1,3,5-trimethoxybenezene as an internal standard.

Kinetics of the imine formation

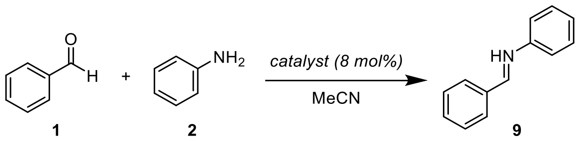

A solution of benzaldehyde (5.3 mg, 0.05 mmol), aniline (4.7 mg, 0.05 mmol), a catalyst or additive (0.004 mmol, 0.7 mg for anhydrous *p*-toluenesulfonic acid; or 0.004 mmol, 1.3 mg for Cu(MeCN)BF$_4$; or 0.05 mmol, 6.4 mg for acid **3**; one experiment was also carried out without a catalyst or additive) in acetonitrile-*d*$_3$ (0.5 mL) was monitored by ^{1}H NMR spectroscopy at rt.

Amine products

N-(Cyclohexyl(phenyl)methyl)aniline (4a)

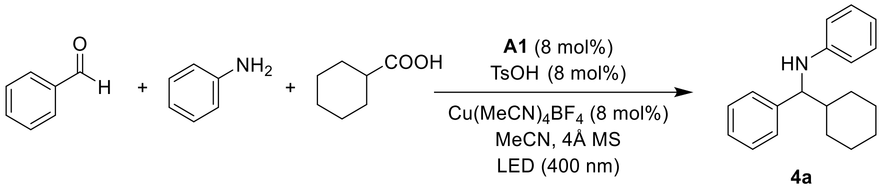

4a

According to GP1, the reaction was carried out with Cu(MeCN)BF_4 (5.0 mg, 0.016 mmol, 8 mol%), acridine **A1** (4.7 mg, 0.016 mmol, 8 mol %), 4Å molecular sieves (60 mg), aldehyde (21.2 mg, 0.2 mmol), aniline (22.3 mg, 0.24 mmol, 1.2 equiv.), carboxylic acid (33.3 mg, 0.26 mmol, 1.3 equiv.), anhydrous *p*-toluenesulfonic acid (2.8 mg, 0.016 mmol, 8 mol%), and acetonitrile (2 mL). The test-tube was capped and the reaction mixture was irradiated with LED light (λ = 400 nm) while stirring at at room temperature for 30 h. The reaction mixture was then concentrated under reduced pressure, and the remaining material was purified by flash chromatography on silica gel (EtOAc/hexane, 1 : 20 v/v) to give product **4a (**50.4 mg, 95%) as a colourless oil.

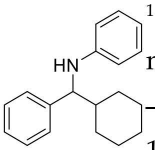

^{1}H NMR (500 MHz, $CDCl_3$): 7.31 (4 H, d, *J* = 4.4 Hz), 7.23 (1 H, dt, *J* = 8.7, 4.2 Hz), 7.13 – 7.04 (2 H, m), 6.63 (1 H, t, *J* = 7.3 Hz), 6.53 (2 H, d, *J* = 8.0 Hz), 4.14 (1 H, d, *J* = 6.3 Hz), 2.06 – 1.86 (1 H, m), 1.83 – 1.61 (4 H, m), 1.60 – 1.53 (1 H, m), 1.28 – 0.98 (5 H, m) ppm. – ^{13}C NMR (125 MHz, $CDCl_3$): 129.2, 128.3, 127.4, 126.9, 117.1, 113.4, 63.6, 45.0, 30.3, 29.6, 26.5, 26.5 ppm – IR: 2921, 2850, 1601, 1503, 1319, 748, 702 cm^{-1}. – HRMS: calcd for $C_{19}H_{23}N$: 266.1903, found 266.1904 [M+H$^+$].

N-(Cyclohexyl(phenyl)methyl)-4-methylaniline (4b)

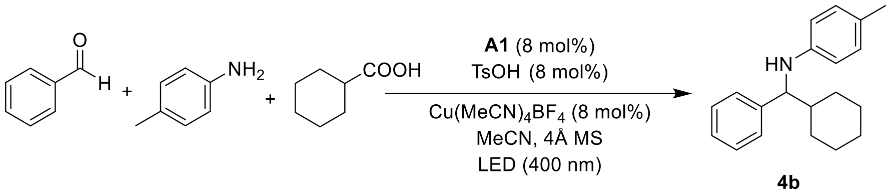

4b

According to GP1, the reaction was carried out with Cu(MeCN)BF_4 (5.0 mg, 0.016 mmol, 8 mol%), acridine **A1** (4.7 mg, 0.016 mmol, 8 mol %), 4Å molecular sieves (60 mg), aldehyde (21.2 mg, 0.2 mmol), aniline (25.7 mg, 0.24 mmol, 1.2 equiv.), carboxylic acid (33.3 mg, 0.26 mmol, 1.3 equiv.), anhydrous *p*-toluenesulfonic acid (2.8 mg, 0.016 mmol, 8 mol%), and acetonitrile (2 mL). The test-tube was capped and the reaction mixture was irradiated with LED light (λ = 400 nm) while stirring at at room temperature for 30 h. The reaction mixture was then concentrated under reduced pressure, and the remaining material was purified by flash chromatography on silica gel (EtOAc/hexane, 1 : 20 v/v) to give product **4b (**50.2 mg, 90%) as a white solid (m.p. 76 ^{o}C)..

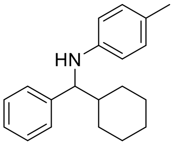

^{1}H NMR (500 MHz, $CDCl_3$): 7.30 (4 H, d, *J* = 4.3 Hz), 7.21 (1 H, ddd, *J* = 8.5, 5.1, 3.6 Hz), 7.03 – 6.76 (2 H, m), 6.44 (2 H, d, *J* = 8.4 Hz), 4.10 (2 H, d, *J* = 6.2 Hz), 2.18 (3 H, s), 1.90 (1 H, dt, *J* = 13.3, 3.0 Hz), 1.83 – 1.48 (5 H, m), 1.36 – 0.84 (5 H, m) ppm. – ^{13}C NMR (125 MHz, $CDCl_3$): 145.7, 143.0, 129.7, 128.3, 127.4, 126.8, 126.1, 113.4, 63.8, 45.1, 30.4, 29.6, 26.6, 26.5, 26.5, 20.4 ppm – IR: 2925, 1740, 1365, 1218 cm^{-1}. – HRMS: calcd for $C_{20}H_{25}N$: 280.2060, found 280.2058 [M+H$^+$].

N-(Cyclohexyl(phenyl)methyl)-3,4,5-trimethylaniline (4c)

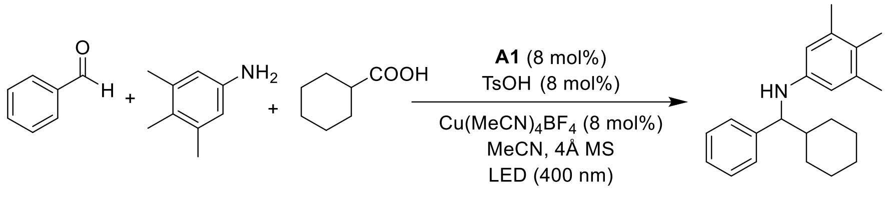

4c

According to GP1, the reaction was carried out with Cu(MeCN)BF$_4$ (5.0 mg, 0.016 mmol, 8 mol%), acridine **A1** (4.7 mg, 0.016 mmol, 8 mol %), 4Å molecular sieves (60 mg), aldehyde (21.2 mg, 0.2 mmol), aniline (32.4 mg, 0.24 mmol, 1.2 equiv.), carboxylic acid (33.3 mg, 0.26 mmol, 1.3 equiv.), anhydrous *p*-toluenesulfonic acid (2.8 mg, 0.016 mmol, 8 mol%), and acetonitrile (2 mL). The test-tube was capped and the reaction mixture was irradiated with LED light (λ = 400 nm) while stirring at at room temperature for 30 h. The reaction mixture was then concentrated under reduced pressure, and the remaining material was purified by flash chromatography on silica gel (EtOAc/hexane, 1 : 20 v/v) to give product **4c** (55.3 mg, 90%) as a white solid (m.p. 75 ^{o}C).

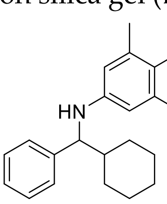

^{1}H NMR (500 MHz, CDCl$_3$): 7.35 – 7.29 (4 H, m), 7.23 (1 H, tt, *J* = 5.7, 3.1 Hz), 6.26 (2 H, s), 4.12 (1 H, d, *J* = 6.3 Hz), 3.96 (1 H, s), 2.16 (6 H, s), 2.04 (3 H, s), 1.91 (1 H, dt, *J* = 12.2, 3.2 Hz), 1.84 – 1.72 (2 H, m), 1.67 (2 H, tdd, *J* = 11.8, 5.7, 2.7 Hz), 1.59 – 1.50 (1 H, m), 1.29 – 1.03 (5 H, m) ppm. – ^{13}C NMR (125 MHz, CDCl$_3$): 145.5, 143.3, 137.1, 128.3, 127.3, 126.7, 123.5, 112.8, 63.5, 45.1, 30.5, 29.5, 26.6, 26.5, 26.5, 20.9, 14.4 ppm – IR: 2924, 1739, 1365, 1217 cm^{-1}. – HRMS: calcd for $C_{22}H_{29}N$: 308.2373, found 308.2372 [M+H$^+$].

N-(Cyclohexyl(phenyl)methyl)-2,3-dihydro-1H-inden-5-amine (4d)

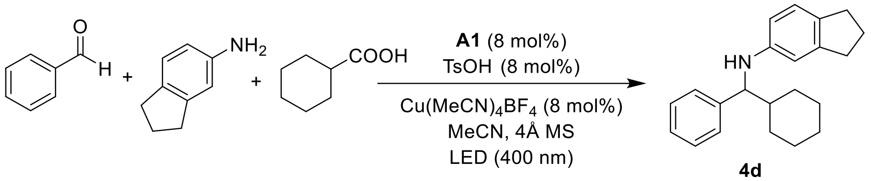

4d

According to GP1, the reaction was carried out with Cu(MeCN)BF$_4$ (5.0 mg, 0.016 mmol, 8 mol%), acridine **A1** (4.7 mg, 0.016 mmol, 8 mol %), 4Å molecular sieves (60 mg), aldehyde (21.2 mg, 0.2 mmol), aniline (31.9 mg, 0.24 mmol, 1.2 equiv.), carboxylic acid (33.3 mg, 0.26 mmol, 1.3 equiv.), anhydrous *p*-toluenesulfonic acid (2.8 mg, 0.016 mmol, 8 mol%), and acetonitrile (2 mL). The test-tube was capped and the reaction mixture was irradiated with LED light (λ = 400 nm) while stirring at at room temperature for 30 h. The reaction mixture was then concentrated under reduced pressure, and the remaining material was purified by flash chromatography on silica gel (EtOAc/hexane, 1 : 20 v/v) to give product **4d** (59.2 mg, 97%) as a colourless oil.

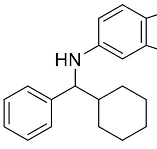

^{1}H NMR (300 MHz, CDCl$_3$): 7.36 – 7.28 (4 H, m), 7.22 (1 H, ddt, *J* = 5.5, 4.4, 3.6 Hz), 6.93 (1 H, d, *J* = 8.0 Hz), 6.44 (1 H, d, *J* = 2.3 Hz), 6.32 (1 H, dd, *J* = 8.1, 2.3 Hz), 4.11 (2 H, d, *J* = 6.1 Hz), 2.75 (4 H, td, *J* = 7.5, 6.5, 3.1 Hz), 2.11 – 1.83 (3 H, m), 1.83 – 1.45 (5 H, m), 1.33 – 0.96 (5 H, m) ppm. – ^{13}C NMR (125 MHz, CDCl$_3$):146.8, 145.3, 143.2, 132.7, 128.3, 127.3, 126.7, 124.7, 111.5, 109.4, 63.9, 45.1, 33.2, 32.0, 30.4, 29.5, 26.6, 26.5, 26.5, 25.8 ppm – IR: 2924, 2849, 1739, 1499, 1217, 702 cm^{-1}. – HRMS: calcd for $C_{22}H_{27}N$: 306.2216, found 306.2216 [M+H$^+$].

N-(Cyclohexyl(phenyl)methyl)-4-fluoroaniline (4e)

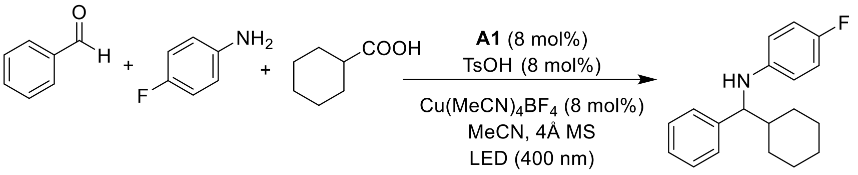

4e

According to GP1, the reaction was carried out with Cu(MeCN)BF$_4$ (5.0 mg, 0.016 mmol, 8 mol%), acridine **A1** (4.7 mg, 0.016 mmol, 8 mol %), 4Å molecular sieves (60 mg), aldehyde (21.2 mg, 0.2 mmol), aniline (26.6 mg, 0.24 mmol, 1.2 equiv.), carboxylic acid (33.3 mg, 0.26 mmol, 1.3 equiv.), anhydrous *p*-toluenesulfonic acid (2.8 mg, 0.016 mmol, 8 mol%), and acetonitrile (2 mL). The test-tube was capped and the reaction mixture was irradiated with LED light (λ = 400 nm) while stirring at at room temperature for 30 h. The reaction mixture was then concentrated under reduced pressure, and the remaining material was purified by flash chromatography on silica gel (EtOAc/hexane, 1 : 15 v/v) to give product **4e** (47.5 mg, 84%) as a colourless oil.

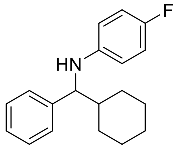

^{1}H NMR (500 MHz, CDCl$_3$): 7.37 – 7.27 (4 H, m), 7.27 – 7.21 (1 H, m), 6.91 – 6.68 (2 H, m), 6.49 – 6.38 (2 H, m), 4.07 (2 H, d, J = 6.2 Hz), 1.92 (1 H, ddd, J = 13.0, 3.5, 1.8 Hz), 1.85 – 1.72 (2 H, m), 1.71 – 1.62 (2 H, m), 1.60 – 1.51 (1 H, m), 1.34 – 1.00 (5 H, m) ppm. – ^{13}C NMR (125 MHz, CDCl$_3$): 155.63 (d, J = 234.3 Hz), 144.26, 142.57, 128.35, 127.36, 126.96, 115.56 (d, J = 22.3 Hz), 114.02 (d, J = 7.4 Hz), 64.22, 45.05, 30.33, 29.65, 26.55, 26.51, 26.47 ppm. – ^{19}F NMR (376 MHz, CDCl$_3$) δ -128.8 ppm. – IR: 2925, 2852, 1508, 1218, 817, 702 cm^{-1}. – HRMS: calcd for $C_{19}H_{22}FN$: 284.1809, found 284.1805 [M+H$^+$].

4-Chloro-N-(Cyclohexyl(phenyl)methyl)aniline (4f)

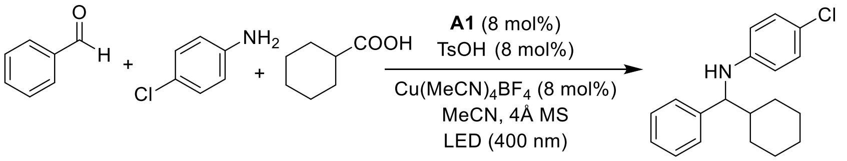

4f

According to GP1, the reaction was carried out with Cu(MeCN)BF$_4$ (5.0 mg, 0.016 mmol, 8 mol%), acridine **A1** (4.7 mg, 0.016 mmol, 8 mol %), 4Å molecular sieves (60 mg), aldehyde (21.2 mg, 0.2 mmol), aniline (30.5 mg, 0.24 mmol, 1.2 equiv.), carboxylic acid (33.3 mg, 0.26 mmol, 1.3 equiv.), anhydrous *p*-toluenesulfonic acid (2.8 mg, 0.016 mmol, 8 mol%), and acetonitrile (2 mL). The test-tube was capped and the reaction mixture was irradiated with LED light (λ = 400 nm) while stirring at at room temperature for 30 h. The reaction mixture was then concentrated under reduced pressure, and the remaining material was purified by flash chromatography on silica gel (EtOAc/hexane, 1 : 20 v/v) to give product **4f** (50.4 mg, 84%) as a white solid (m.p. 105 ^{o}C).

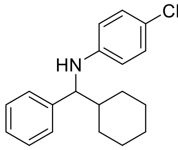

^{1}H NMR (500 MHz, CDCl$_3$): 7.34 – 7.19 (5 H, m), 6.99 (d, J = 8.7 Hz, 2 H), 6.40 (2 H, d, J = 8.4 Hz), 4.17 (1 H, s), 4.06 (1 H, d, J = 6.3 Hz), 1.88 (1 H, d, J = 12.9 Hz), 1.81 – 1.68 (2 H, m), 1.65 (2 H, dd, J = 9.2, 5.7 Hz), 1.52 (2 H, d, J = 13.8 Hz), 1.29 – 0.94 (5 H, m) ppm. – ^{13}C NMR (125 MHz, CDCl$_3$): 129.0, 128.4, 127.3, 127.0, 114.4, 63.7, 45.0, 30.3, 29.6, 26.5, 26.5, 26.4 ppm – IR: 2925, 2851, 1738, 1498, 1217, 703 cm^{-1}. – HRMS: calcd for $C_{19}H_{22}ClN$: 300.1514, found 300.1514 [M+H$^+$].

3-Bromo-*N*-(Cyclohexyl(phenyl)methyl)aniline (4g)

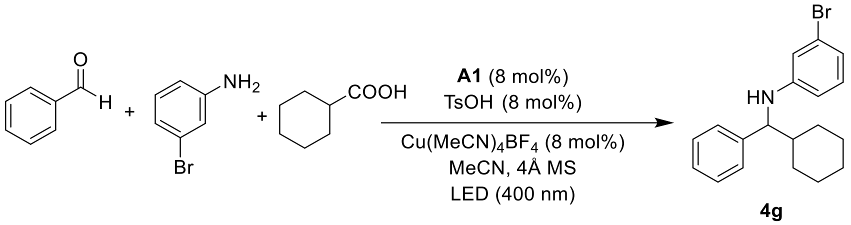

4g

According to GP1, the reaction was carried out with Cu(MeCN)BF_4 (5.0 mg, 0.016 mmol, 8 mol%), acridine **A1** (4.7 mg, 0.016 mmol, 8 mol %), 4Å molecular sieves (60 mg), aldehyde (21.2 mg, 0.2 mmol), aniline (41.3 mg, 0.24 mmol, 1.2 equiv.), carboxylic acid (33.3 mg, 0.26 mmol, 1.3 equiv.), anhydrous *p*-toluenesulfonic acid (2.8 mg, 0.016 mmol, 8 mol%), and acetonitrile (2 mL). The test-tube was capped and the reaction mixture was irradiated with LED light (λ = 400 nm) while stirring at at room temperature for 30 h. The reaction mixture was then concentrated under reduced pressure, and the remaining material was purified by flash chromatography on silica gel (EtOAc/hexane, 1 : 20 v/v) to give product **4g** (60.5 mg, 88%) as a colourless oil.

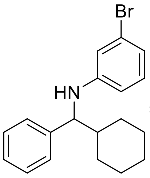

^{1}H NMR (500 MHz, $CDCl_3$): 7.34 – 7.28 (2 H, m), 7.27 – 7.19 (3 H, m), 6.89 (1 H, t, *J* = 8.0 Hz), 6.74 – 6.69 (1 H, m), 6.66 (1 H, t, *J* = 2.1 Hz), 6.38 (1 H, ddd, *J* = 8.2, 2.3, 0.9 Hz), 4.22 (1 H, s), 4.07 (1 H, d, *J* = 6.4 Hz), 1.88 (1 H, ddt, *J* = 12.9, 3.5, 1.8 Hz), 1.81 – 1.68 (2 H, m), 1.64 (2 H, dddd, *J* = 14.9, 11.7, 6.4, 3.3 Hz), 1.51 (1 H, ddt, *J* = 13.0, 3.5, 1.8 Hz), 1.27 – 0.97 (5 H, m) ppm. – ^{13}C NMR (125 MHz, $CDCl_3$): 149.2, 142.1, 130.5, 128.4, 127.2, 127.1, 123.1, 119.9, 116.1, 111.8, 63.4, 44.9, 30.3, 29.6, 26.5, 26.4, 26.4 ppm – IR: 2921, 2849, 1593, 1480, 985, 702, 681 cm^{-1}. – HRMS: calcd for $C_{19}H_{22}BrN$: 344.1008, found 344.1002 [M+H$^+$].

N-(Cyclohexyl(phenyl)methyl)-3-iodoaniline (4h)

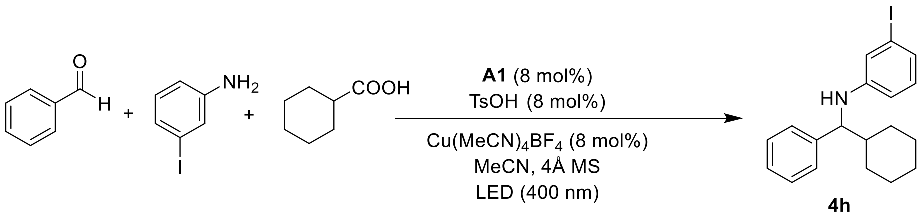

4h

According to GP1, the reaction was carried out with Cu(MeCN)BF_4 (5.0 mg, 0.016 mmol, 8 mol%), acridine **A1** (4.7 mg, 0.016 mmol, 8 mol %), 4Å molecular sieves (60 mg), aldehyde (21.2 mg, 0.2 mmol), aniline (52.6 mg, 0.24 mmol, 1.2 equiv.), carboxylic acid (33.3 mg, 0.26 mmol, 1.3 equiv.), anhydrous *p*-toluenesulfonic acid (2.8 mg, 0.016 mmol, 8 mol%), and acetonitrile (2 mL). The test-tube was capped and the reaction mixture was irradiated with LED light (λ = 400 nm) while stirring at at room temperature for 30 h. The reaction mixture was then concentrated under reduced pressure, and the remaining material was purified by flash chromatography on silica gel (EtOAc/hexane, 1 : 20 v/v) to give product **4h** (62.6 mg, 80%) as a colourless oil.

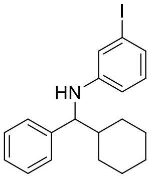

^{1}H NMR (500 MHz, $CDCl_3$): δ 7.35 – 7.28 (2 H, m), 7.27 – 7.19 (3 H, m), 6.98 – 6.85 (2 H, m), 6.79 – 6.70 (1 H, m), 6.45 – 6.38 (1 H, m), 4.17 (1 H, s), 4.06 (1 H, d, *J* = 6.4 Hz), 1.88 (1H, d, *J* = 13.1 Hz), 1.74 (2 H, dd, *J* = 25.4, 12.3 Hz), 1.64 (2 H, dddd, *J* = 11.8, 8.6, 5.9, 3.2 Hz), 1.58 – 1.44 (1 H, m), 1.34 – 0.96 (5 H, m) ppm. – ^{13}C NMR (125 MHz, $CDCl_3$): 149.1, 142.1, 130.6, 128.4, 127.2, 127.1, 125.9, 122.2, 112.3, 95.1, 63.3, 44.9, 30.3, 29.6, 26.5, 26.4, 26.4 ppm – IR: 2924, 2850, 1738, 1365, 1217 cm^{-1}. – HRMS: calcd for $C_{19}H_{22}IN$: 392.0807, found 392.0866 [M+H$^+$].

Go to table of contents

N-(Cyclohexyl(phenyl)methyl)-2-methoxyaniline (4i)

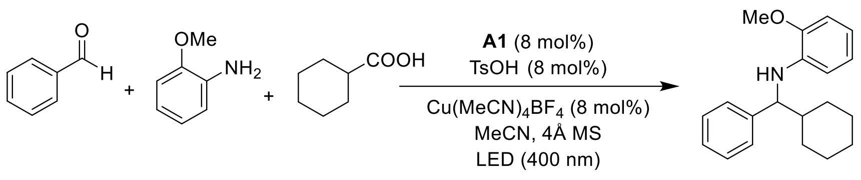

4i

According to GP1, the reaction was carried out with Cu(MeCN)BF_4 (5.0 mg, 0.016 mmol, 8 mol%), acridine **A1** (4.7 mg, 0.016 mmol, 8 mol %), 4Å molecular sieves (60 mg), aldehyde (21.2 mg, 0.2 mmol), aniline (29.5 mg, 0.24 mmol, 1.2 equiv.), carboxylic acid (33.3 mg, 0.26 mmol, 1.3 equiv.), anhydrous *p*-toluenesulfonic acid (2.8 mg, 0.016 mmol, 8 mol%), and acetonitrile (2 mL). The test-tube was capped and the reaction mixture was irradiated with LED light (λ = 400 nm) while stirring at at room temperature for 30 h. The reaction mixture was then concentrated under reduced pressure, and the remaining material was purified by flash chromatography on silica gel (EtOAc/hexane, 1 : 20 v/v) to give product **4i** (47.2 mg, 80%) as a colourless oil.

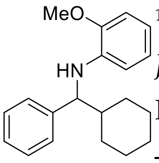

^{1}H NMR (500 MHz, $CDCl_3$): 7.31 (4 H, q, *J* = 4.0, 2.9 Hz), 7.21 (1 H, tq, *J* = 5.5, 2.6 Hz), 6.76 (1 H, dd, *J* = 7.8, 1.4 Hz), 6.68 (1 H, td, *J* = 7.7, 1.4 Hz), 6.58 (1 H, td, *J* = 7.7, 1.6 Hz), 6.33 (1 H, dd, *J* = 7.9, 1.6 Hz), 4.83 (1 H, s), 4.12 (1 H, d, *J* = 6.2 Hz), 3.91 (3 H, s), 2.01 – 1.87 (1 H, m), 1.83 – 1.62 (4 H, m), 1.62 – 1.49 (1 H, m), 1.33 – 1.00 (5 H, m) ppm. – ^{13}C NMR (125 MHz, $CDCl_3$): 146.8, 143.0, 137.8, 128.2, 127.4, 126.8, 121.3, 116.0, 110.8, 109.4, 63.4, 55.7, 45.0, 30.4, 29.6, 26.6, 26.5 ppm – IR: 2924, 2851, 1602, 1510, 1454, 1221, 1028, 702 cm^{-1}. – HRMS: calcd for $C_{20}H_{25}NO$: 296.2009, found 296.2004 [$M+H^+$].

N-(Cyclohexyl(phenyl)methyl)-3-(methylthio)aniline (4j)

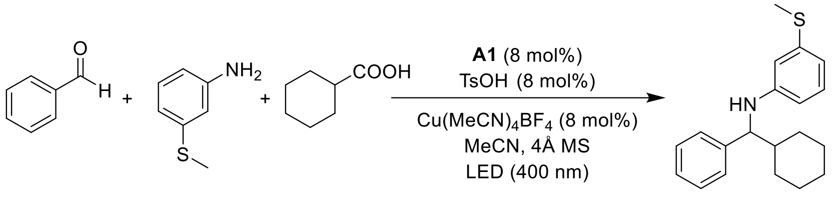

4j

According to GP1, the reaction was carried out with Cu(MeCN)BF_4 (5.0 mg, 0.016 mmol, 8 mol%), acridine **A1** (4.7 mg, 0.016 mmol, 8 mol %), 4Å molecular sieves (60 mg), aldehyde (21.2 mg, 0.2 mmol), aniline (33.4 mg, 0.24 mmol, 1.2 equiv.), carboxylic acid (33.3 mg, 0.26 mmol, 1.3 equiv.), anhydrous *p*-toluenesulfonic acid (2.8 mg, 0.016 mmol, 8 mol%), and acetonitrile (2 mL). The test-tube was capped and the reaction mixture was irradiated with LED light (λ = 400 nm) while stirring at at room temperature for 30 h. The reaction mixture was then concentrated under reduced pressure, and the remaining material was purified by flash chromatography on silica gel (EtOAc/hexane, 1 : 20 v/v) to give product **4j** (44.8 mg, 72%) as a colourless oil.

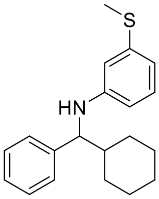

^{1}H NMR (500 MHz, $CDCl_3$): 7.34 – 7.26 (4 H, m), 7.24 – 7.19 (1 H, m), 6.97 (1 H, t, *J* = 7.9 Hz), 6.65 – 6.47 (1 H, m), 6.40 (1 H, s), 6.28 (1 H, dd, *J* = 8.2, 2.3 Hz), 4.19 (1 H, s), 4.11 (1 H, d, *J* = 6.4 Hz), 2.34 (3 H, s), 1.97 – 1.86 (1 H, m), 1.84 – 1.59 (4 H, m), 1.53 (1 H, ddd, *J* = 13.7, 5.3, 2.8 Hz), 1.36 – 0.94 (5 H, m) ppm. – ^{13}C NMR (125 MHz, $CDCl_3$): 148.2, 142.5, 139.0, 129.4, 128.4, 127.3, 126.9, 115.3, 111.1, 110.4, 63.5, 44.9, 30.3, 29.6, 26.5, 26.5, 26.4, 15.7 ppm – IR: 2920, 2850, 1738, 1590, 1217, 702 cm^{-1}. – HRMS: calcd for $C_{20}H_{25}NS$: 312.1780, found 312.1780 [$M+H^+$].

N-(Cyclohexyl(phenyl)methyl)-3-(trifluoromethoxy)aniline (4k)

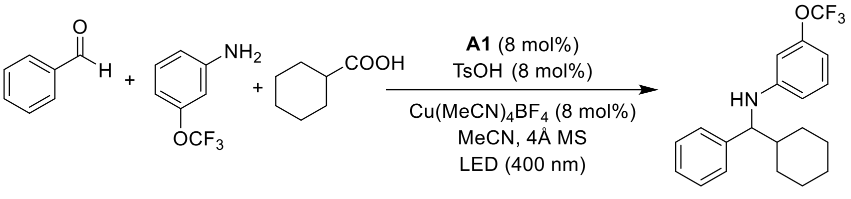

4k

According to GP1, the reaction was carried out with Cu(MeCN)BF_4 (5.0 mg, 0.016 mmol, 8 mol%), acridine **A1** (4.7 mg, 0.016 mmol, 8 mol %), 4Å molecular sieves (60 mg), aldehyde (21.2 mg, 0.2 mmol), aniline (42.5 mg, 0.24 mmol, 1.2 equiv.), carboxylic acid (33.3 mg, 0.26 mmol, 1.3 equiv.), anhydrous *p*-toluenesulfonic acid (2.8 mg, 0.016 mmol, 8 mol%), and acetonitrile (2 mL). The test-tube was capped and the reaction mixture was irradiated with LED light (λ = 400 nm) while stirring at at room temperature for 30 h. The reaction mixture was then concentrated under reduced pressure, and the remaining material was purified by flash chromatography on silica gel (EtOAc/hexane, 1 : 20 v/v) to give product **4k** (51.0 mg, 73%) as a colourless oil.

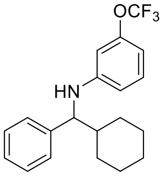

^{1}H NMR (500 MHz, $CDCl_3$): 7.41 – 7.15 (5 H, m), 7.02 (1 H, t, J = 8.2 Hz), 6.45 – 6.41 (1 H, m), 6.39 (1 H, dd, J = 8.2, 2.2 Hz), 6.32 (1 H, s), 4.30 (1 H, s), 4.08 (1 H, d, J = 6.4 Hz), 2.00 – 1.86 (1 H, m), 1.82 – 1.60 (4 H, m), 1.57 – 1.47 (1 H, m), 1.33 – 0.94 (5 H, m) ppm. – ^{13}C NMR (125 MHz, $CDCl_3$): 197.5, 150.4, 149.2, 142.0, 130.0, 128.5, 127.3, 127.1, 111.5, 108.8, 105.5, 63.6, 44.9, 30.3, 29.7, 26.5, 26.4, 26.4 ppm. – ^{19}F NMR (376MHz, $CDCl_3$) δ -57.5 ppm. – IR: 2928, 2853, 1738, 1615, 1217, 1157 cm^{-1}. – HRMS: calcd for $C_{20}H_{22}FNO$: 350.1726, found 350.1726 [M+H$^+$].

N-(Cyclohexyl(phenyl)methyl)-[1,1'-biphenyl]-4-amine (4l)

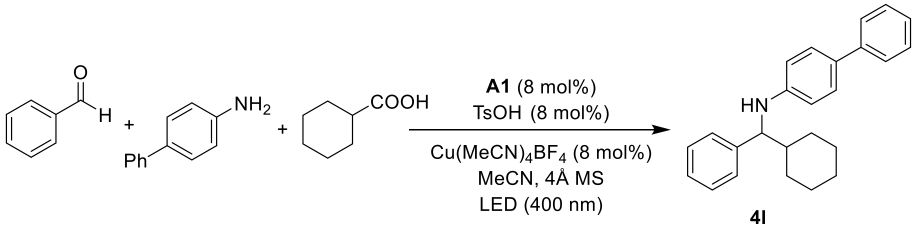

4l

According to GP1, the reaction was carried out with Cu(MeCN)BF_4 (5.0 mg, 0.016 mmol, 8 mol%), acridine **A1** (4.7 mg, 0.016 mmol, 8 mol %), 4Å molecular sieves (60 mg), aldehyde (21.2 mg, 0.2 mmol), aniline (40.6 mg, 0.24 mmol, 1.2 equiv.), carboxylic acid (33.3 mg, 0.26 mmol, 1.3 equiv.), anhydrous *p*-toluenesulfonic acid (2.8 mg, 0.016 mmol, 8 mol%), and acetonitrile (2 mL). The test-tube was capped and the reaction mixture was irradiated with LED light (λ = 400 nm) while stirring at at room temperature for 30 h. The reaction mixture was then concentrated under reduced pressure, and the remaining material was purified by flash chromatography on silica gel (EtOAc/hexane, 1 : 20 v/v) to give product **4l** (64.8 mg, 95%) as a colourless oil.

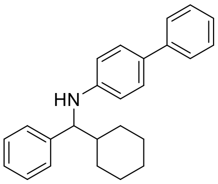

^{1}H NMR (500 MHz, $CDCl_3$): 7.48 (2 H, d, J = 7.7 Hz), 7.34 (8 H, dd, J = 14.0, 4.6 Hz), 7.23 (2 H, dq, J = 7.4, 3.7, 3.0 Hz), 6.58 (2 H, d, J = 8.2 Hz), 4.27 (1 H, s), 4.17 (1 H, d, J = 6.3 Hz), 1.97 – 1.88 (1 H, m), 1.85 – 1.63 (4 H, m), 1.56 (1 H, dd, J = 15.8, 3.8 Hz), 1.32 – 0.97 (5 H, m) ppm. – ^{13}C NMR (125 MHz, $CDCl_3$):147.2, 142.6, 141.3, 129.9, 128.6, 128.3, 127.8, 127.2, 126.8, 126.2, 125.9, 113.4, 63.4, 44.9, 30.3, 29.5, 26.4, 26.4, 26.4 ppm – IR: 2925, 2851, 1738, 1612, 1217, 700 cm^{-1}. – HRMS: calcd for $C_{25}H_{27}N$: 342.2216, found 342.2216 [M+H$^+$].

Go to table of contents

N-(Cyclohexyl(phenyl)methyl)-[1,1'-biphenyl]-2-amine (4m)

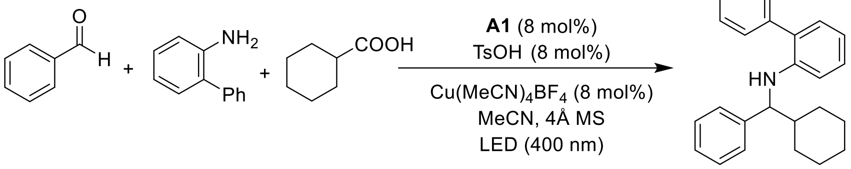

4m

According to GP1, the reaction was carried out with Cu(MeCN)BF$_4$ (5.0 mg, 0.016 mmol, 8 mol%), acridine **A1** (4.7 mg, 0.016 mmol, 8 mol %), 4Å molecular sieves (60 mg), aldehyde (21.2 mg, 0.2 mmol), aniline (40.6 mg, 0.24 mmol, 1.2 equiv.), carboxylic acid (33.3 mg, 0.26 mmol, 1.3 equiv.), anhydrous *p*-toluenesulfonic acid (2.8 mg, 0.016 mmol, 8 mol%), and acetonitrile (2 mL). The test-tube was capped and the reaction mixture was irradiated with LED light (λ = 400 nm) while stirring at at room temperature for 30 h. The reaction mixture was then concentrated under reduced pressure, and the remaining material was purified by flash chromatography on silica gel (EtOAc/hexane, 1 : 20 v/v) to give product **4m** (42.9 mg, 96%) as a colourless oil.

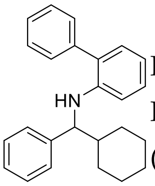

^{1}H NMR (500 MHz, CDCl$_3$): 7.49 (3 H, d, *J* = 7.0 Hz), 7.42 – 7.34 (1 H, m), 7.27 (3 H, dd, *J* = 14.2, 6.8 Hz), 7.20 (3 H, dd, *J* = 14.2, 7.2 Hz), 7.09 – 6.98 (2 H, m), 6.66 (1 H, t, *J* = 7.4 Hz), 6.40 (1 H, d, *J* = 8.2 Hz), 4.44 (1 H, s), 4.12 (1 H, d, *J* = 5.9 Hz), 1.71 – 1.47 (5 H, m), 1.43 (1 H, d, *J* = 13.2 Hz), 1.19 – 0.73 (5 H, m). ppm. – ^{13}C NMR (125 MHz, CDCl$_3$): 142.7, 139.8, 130.1, 129.6, 129.0, 128.6, 128.3, 127.4, 127.3, 126.8, 116.6, 111.5, 63.5, 45.0, 30.6, 29.1, 26.5, 26.4 ppm – IR: 2924, 2851, 1738, 1508, 1217, 702 cm^{-1}. – HRMS: calcd for $C_{25}H_{27}N$: 342.2216, found 342.2216 [M+H$^+$].

N-(Cyclohexyl(phenyl)methyl)-3-(4,4,5,5-tetramethyl-1,3,2-dioxaborolan-2-yl)aniline (4n)

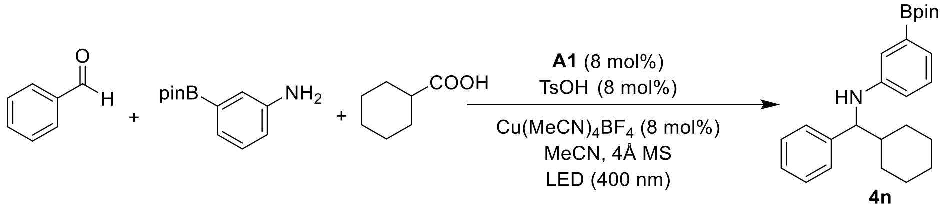

4n

According to GP1, the reaction was carried out with Cu(MeCN)BF$_4$ (5.0 mg, 0.016 mmol, 8 mol%), acridine **A1** (4.7 mg, 0.016 mmol, 8 mol %), 4Å molecular sieves (60 mg), aldehyde (21.2 mg, 0.2 mmol), aniline (52.6 mg, 0.24 mmol, 1.2 equiv.), carboxylic acid (33.3 mg, 0.26 mmol, 1.3 equiv.), anhydrous *p*-toluenesulfonic acid (2.8 mg, 0.016 mmol, 8 mol%), and acetonitrile (2 mL). The test-tube was capped and the reaction mixture was irradiated with LED light (λ = 400 nm) while stirring at at room temperature for 30 h. The reaction mixture was then concentrated under reduced pressure, and the remaining material was purified by flash chromatography on silica gel (EtOAc/hexane, 1 : 20 v/v) to give product **4n** (70.4 mg, 90%) as a white solid (m.p. 110 ^{o}C).

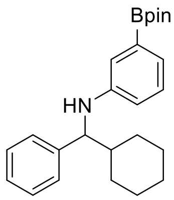

^{1}H NMR (500 MHz, CDCl$_3$): 7.29 (4 H, d, *J* = 4.3 Hz), 7.20 (1 H, p, *J* = 4.3 Hz), 7.11 (1 H, d, *J* = 2.5 Hz), 7.08 – 7.03 (2 H, m), 6.49 (1 H, dt, *J* = 5.8, 3.1 Hz), 4.36 – 4.04 (2 H, m), 1.87 (1 H, dd, *J* = 12.6, 3.3 Hz), 1.74 (2 H, tdd, *J* = 15.6, 5.6, 2.8 Hz), 1.65 (2 H, dddd, *J* = 14.7, 12.3, 6.0, 2.9 Hz), 1.59 – 1.50 (1 H, m), 1.34 (12 H, d, *J* = 3.7 Hz), 1.27 – 0.98 (5 H, m) ppm. – ^{13}C NMR (125 MHz, CDCl$_3$): 147.2, 142.8, 128.7, 128.3, 127.3, 126.8, 123.3, 120.6, 115.0, 83.7, 63.2, 45.1, 30.5, 29.3, 26.6, 26.5, 25.0, 24.9 ppm. – ^{11}B NMR (128 MHz, CDCl$_3$) δ 30.8 ppm. – IR: 2925, 2852, 1738, 1361, 1144, 704 cm^{-1}. – HRMS: calcd for $C_{25}H_{34}BNO_2$: 392.2755, found 392.2755 [M+H$^+$].

N-(Cyclohexyl(phenyl)methyl)-4-(4,4,5,5-tetramethyl-1,3,2-dioxaborolan-2-yl)aniline (4o)

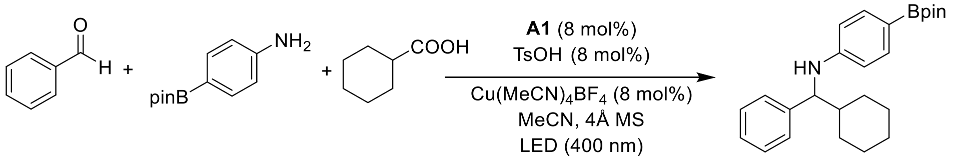

4o

According to GP1, the reaction was carried out with Cu(MeCN)BF$_4$ (5.0 mg, 0.016 mmol, 8 mol%), acridine **A1** (4.7 mg, 0.016 mmol, 8 mol %), 4Å molecular sieves (60 mg), aldehyde (21.2 mg, 0.2 mmol), aniline (52.6 mg, 0.24 mmol, 1.2 equiv.), carboxylic acid (33.3 mg, 0.26 mmol, 1.3 equiv.), anhydrous *p*-toluenesulfonic acid (2.8 mg, 0.016 mmol, 8 mol%), and acetonitrile (2 mL). The test-tube was capped and the reaction mixture was irradiated with LED light (λ = 400 nm) while stirring at at room temperature for 30 h. The reaction mixture was then concentrated under reduced pressure, and the remaining material was purified by flash chromatography on silica gel (EtOAc/hexane, 1 : 20 v/v) to give product **4o** (50.8 mg, 91%) as a white solid (m.p. 95 ^{o}C).

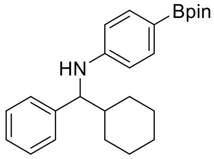

^{1}H NMR (500 MHz, CDCl$_3$): 7.53 (2 H, d, *J* = 8.4 Hz), 7.32 – 7.23 (4 H, m), 7.23 – 7.15 (1 H, m), 6.48 (2 H, d, *J* = 8.3 Hz), 4.36 (1 H, s), 4.18 (1 H, d, *J* = 6.3 Hz), 1.88 (1 H, dt, *J* = 13.0, 3.3 Hz), 1.81 – 1.62 (4 H, m), 1.57 – 1.48 (1 H, m), 1.28 (12 H, d, *J* = 2.6 Hz), 1.25 – 1.00 (5 H, m) ppm. – ^{13}C NMR (125 MHz, CDCl$_3$): 150.3, 142.3, 136.3, 128.3, 127.3, 126.9, 112.4, 83.2, 62.9, 44.9, 30.4, 29.5, 26.5, 26.5, 26.4, 25.0, 24.9 ppm. – ^{11}B NMR (128 MHz, CDCl$_3$) δ 31.3 ppm. – IR: 2926, 2851, 1738, 1360, 705 cm^{-1}. – HRMS: calcd for $C_{25}H_{34}BNO_2$: 392.2755, found 392.2756 [M+H$^+$].

N-(Cyclohexyl(phenyl)methyl)-2-(4,4,5,5-tetramethyl-1,3,2-dioxaborolan-2-yl)aniline (4p)

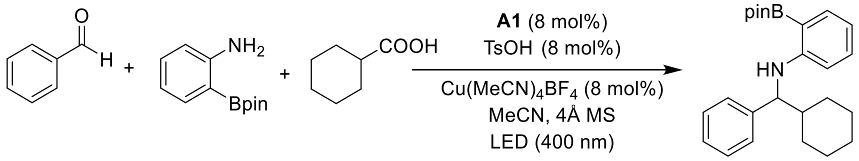

4p

According to GP1, the reaction was carried out with Cu(MeCN)BF$_4$ (5.0 mg, 0.016 mmol, 8 mol%), acridine **A1** (4.7 mg, 0.016 mmol, 8 mol %), 4Å molecular sieves (60 mg), aldehyde (21.2 mg, 0.2 mmol), aniline (52.6 mg, 0.24 mmol, 1.2 equiv.), carboxylic acid (33.3 mg, 0.26 mmol, 1.3 equiv.), anhydrous *p*-toluenesulfonic acid (2.8 mg, 0.016 mmol, 8 mol%), and acetonitrile (2 mL). The test-tube was capped and the reaction mixture was irradiated with LED light (λ = 400 nm) while stirring at at room temperature for 30 h. The reaction mixture was then concentrated under reduced pressure, and the remaining material was purified by flash chromatography on silica gel (EtOAc/hexane, 1 : 20 v/v) to give product **4p** (35.7 mg, 60%) as a colourless oil.

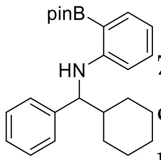

^{1}H NMR (300 MHz, CDCl$_3$): 7.62 (1 H, dd, *J* = 7.4, 1.8 Hz), 7.38 – 7.27 (3 H, m), 7.25 – 7.16 (1 H, m), 7.09 (1 H, ddd, *J* = 8.7, 7.2, 1.8 Hz), 6.61 (1 H, d, *J* = 5.5 Hz), 6.53 (1 H, td, *J* = 7.3, 0.9 Hz), 6.21 (1 H, d, *J* = 8.3 Hz), 4.21 (1 H, t, *J* = 5.1 Hz), 1.91 – 1.62 (6 H, m), 1.41 (12 H, d, *J* = 2.2 Hz), 1.35 – 1.05 (5 H, m) ppm. – ^{13}C NMR (125 MHz, CDCl$_3$): 154.4, 143.2, 137.0, 133.1, 128.2, 127.3, 126.6, 115.2, 110.6, 83.6, 63.0, 45.6, 30.8, 28.1, 26.8, 26.7, 25.2, 25.1 ppm. – ^{11}B NMR (128 MHz, CDCl$_3$) δ 31.0 ppm. – IR: 2925, 1738, 1453, 1360, 1217, 1143 cm^{-1}. – HRMS: calcd for $C_{25}H_{34}BNO_2$: 392.2755, found 392.2755 [M+H$^+$].

N-(Cyclohexyl(phenyl)methyl)benzo[d]thiazol-6-amine (4q)

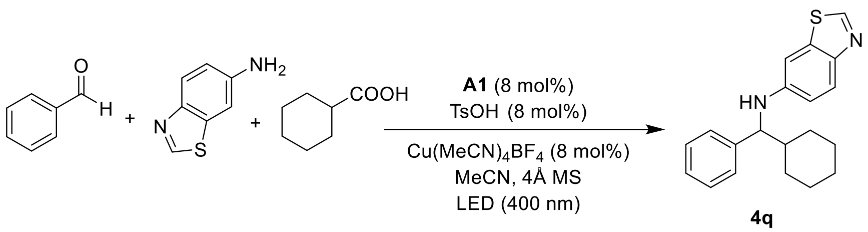

4q

According to GP1, the reaction was carried out with Cu(MeCN)BF_4 (5.0 mg, 0.016 mmol, 8 mol%), acridine **A1** (4.7 mg, 0.016 mmol, 8 mol %), 4Å molecular sieves (60 mg), aldehyde (21.2 mg, 0.2 mmol), aniline (36.0 mg, 0.24 mmol, 1.2 equiv.), carboxylic acid (33.3 mg, 0.26 mmol, 1.3 equiv.), anhydrous *p*-toluenesulfonic acid (2.8 mg, 0.016 mmol, 8 mol%), and acetonitrile (2 mL). The test-tube was capped and the reaction mixture was irradiated with LED light (λ = 400 nm) while stirring at at room temperature for 30 h. The reaction mixture was then concentrated under reduced pressure, and the remaining material was purified by flash chromatography on silica gel (EtOAc/hexane, 1 : 10 v/v) to give product **4q** (38.6 mg, 60%) as a colourless oil.

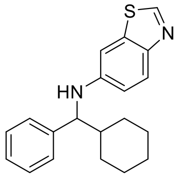

^{1}H NMR (500 MHz, $CDCl_3$): 8.81 (1 H, s), 7.58 (1 H, d, *J* = 8.6 Hz), 7.36 – 7.25 (4 H, m), 7.24 – 7.16 (1 H, m), 7.14 (1 H, d, *J* = 2.3 Hz), 6.77 (1 H, dd, *J* = 8.6, 2.3 Hz), 4.20 (1 H, d, *J* = 6.3 Hz), 1.94 (1 H, dt, *J* = 14.2, 2.5 Hz), 1.83 – 1.62 (3 H, m), 1.61 – 1.46 (1 H, m), 1.31 – 0.97 (5 H, m) ppm. – ^{13}C NMR (125 MHz, $CDCl_3$): 154.9, 154.3, 147.2, 142.2, 128.4, 127.4, 127.0, 122.0, 121.8, 115.1, 105.5, 63.9, 44.9, 30.3, 29.7, 26.5, 26.5, 26.4 ppm – IR: 2925, 1738, 1365, 1217, 702 cm^{-1}. – HRMS: calcd for $C_{20}H_{22}N_2S$: 323.1576, found 323.1571 [M+H$^+$].

N-(Cyclohexyl(phenyl)methyl)-3-(oxazol-4-yl)aniline (4r)

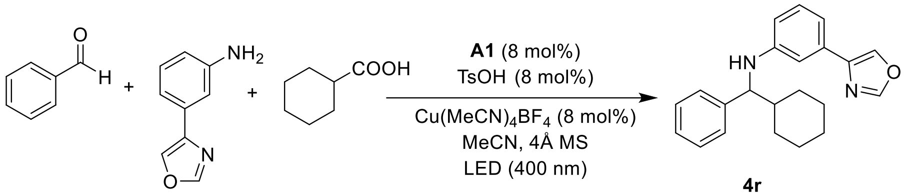

4r

According to GP1, the reaction was carried out with Cu(MeCN)BF_4 (5.0 mg, 0.016 mmol, 8 mol%), acridine **A1** (4.7 mg, 0.016 mmol, 8 mol %), 4Å molecular sieves (60 mg), aldehyde (21.2 mg, 0.2 mmol), aniline (38.4 mg, 0.24 mmol, 1.2 equiv.), carboxylic acid (33.3 mg, 0.26 mmol, 1.3 equiv.), anhydrous *p*-toluenesulfonic acid (2.8 mg, 0.016 mmol, 8 mol%), and acetonitrile (2 mL). The test-tube was capped and the reaction mixture was irradiated with LED light (λ = 400 nm) while stirring at at room temperature for 30 h. The reaction mixture was then concentrated under reduced pressure, and the remaining material was purified by flash chromatography on silica gel (EtOAc/hexane, 1 : 10 v/v) to give product **4r** (43.2 mg, 65%) as a colourless oil.

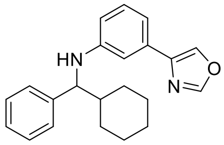

^{1}H NMR (500 MHz, $CDCl_3$): 7.85 (1 H, s), 7.35 – 7.27 (4 H, m), 7.25 – 7.17 (2 H, m), 7.09 (1 H, t, *J* = 7.9 Hz), 6.89 (1 H, dt, *J* = 7.7, 1.1 Hz), 6.80 (1 H, t, *J* = 2.0 Hz), 6.47 (1 H, dd, *J* = 8.1, 2.4 Hz), 4.15 (1 H, d, *J* = 6.4 Hz), 1.99 – 1.88 (1 H, m), 1.84 – 1.61 (4 H, m), 1.54 (1 H, dd, *J* = 12.5, 3.6 Hz), 1.37 – 0.95 (5 H, m) ppm. – ^{13}C NMR (125 MHz, $CDCl_3$): 152.2, 150.3, 148.2, 142.4, 129.8, 128.5, 128.4, 127.3, 127.0, 121.3, 113.6, 113.4, 109.1, 63.6, 45.0, 30.3, 29.7, 26.5, 26.5, 26.4 ppm – IR: 2926, 2852, 1738, 1365, 1217, 703 cm^{-1}. – HRMS: calcd for $C_{22}H_{24}N_2O$: 333.1961, found 333.1956 [M+H$^+$].

N-(Cyclohexyl(*o*-tolyl)methyl)aniline (5a)

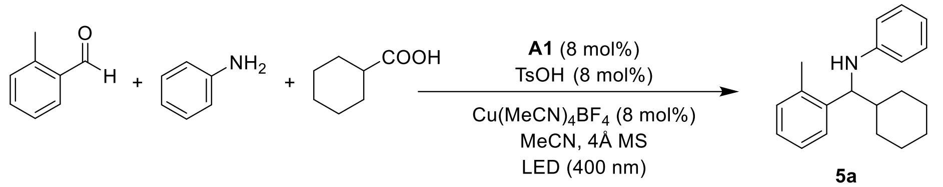

According to GP1, the reaction was carried out with Cu(MeCN)BF_4 (5.0 mg, 0.016 mmol, 8 mol%), acridine **A1** (4.7 mg, 0.016 mmol, 8 mol %), 4Å molecular sieves (60 mg), aldehyde (24.0 mg, 0.2 mmol), aniline (22.3 mg, 0.24 mmol, 1.2 equiv.), carboxylic acid (33.3 mg, 0.26 mmol, 1.3 equiv.), anhydrous *p*-toluenesulfonic acid (2.8 mg, 0.016 mmol, 8 mol%), and acetonitrile (2 mL). The test-tube was capped and the reaction mixture was irradiated with LED light (λ = 400 nm) while stirring at at room temperature for 30 h. The reaction mixture was then concentrated under reduced pressure, and the remaining material was purified by flash chromatography on silica gel (EtOAc/hexane, 1 : 20 v/v) to give product **5a** (50.2 mg, 90%) as a colourless oil.

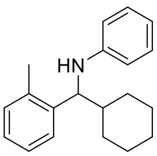

^{1}H NMR (500 MHz, $CDCl_3$): 7.36 – 7.29 (1 H, m), 7.21 – 7.11 (3 H, m), 7.13 – 7.06 (2 H, m), 6.63 (1 H, t, *J* = 7.4 Hz), 6.51 – 6.45 (2 H, m), 4.42 (1 H, d, *J* = 6.1 Hz), 4.14 (1 H, s), 2.49 (3 H, s), 2.00 – 1.90 (1 H, m), 1.87 – 1.55 (5 H, m), 1.36 – 1.06 (5 H, m) ppm. – ^{13}C NMR (125 MHz, $CDCl_3$): 148.0, 141.0, 135.3, 130.6, 129.2, 126.5, 126.4, 126.1, 117.0, 113.0, 59.3, 44.4, 30.8, 28.9, 26.7, 26.6, 19.7 ppm – IR: 2925, 2850, 1738, 1600, 1500, 1365, 1216 cm^{-1}. – HRMS: calcd for $C_{20}H_{25}N$: 280.2060, found 280.2061 [$M+H^+$].

N-(Cyclohexyl(*m*-tolyl)methyl)aniline (5b)

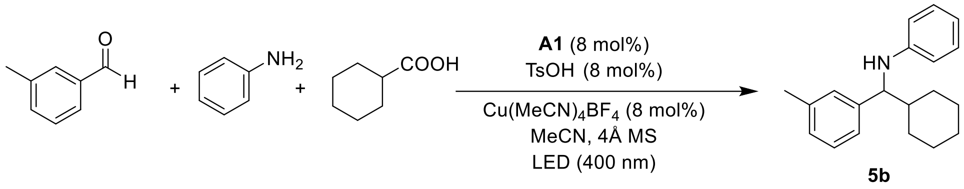

According to GP1, the reaction was carried out with Cu(MeCN)BF_4 (5.0 mg, 0.016 mmol, 8 mol%), acridine **A1** (4.7 mg, 0.016 mmol, 8 mol %), 4Å molecular sieves (60 mg), aldehyde (21.2 mg, 0.2 mmol), aniline (22.3 mg, 0.24 mmol, 1.2 equiv.), carboxylic acid (33.3 mg, 0.26 mmol, 1.3 equiv.), anhydrous *p*-toluenesulfonic acid (2.8 mg, 0.016 mmol, 8 mol%), and acetonitrile (2 mL). The test-tube was capped and the reaction mixture was irradiated with LED light (λ = 400 nm) while stirring at at room temperature for 30 h. The reaction mixture was then concentrated under reduced pressure, and the remaining material was purified by flash chromatography on silica gel (EtOAc/hexane, 1 : 20 v/v) to give product **5b** (53.6 mg, 96%) as a colourless oil.

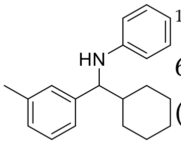

^{1}H NMR (500 MHz, $CDCl_3$): 7.18 (1 H, t, *J* = 7.4 Hz), 7.12 – 7.04 (4 H, m), 7.02 (1 H, d, *J* = 7.5 Hz), 6.61 (1 H, t, *J* = 7.3 Hz), 6.51 (2 H, d, *J* = 7.9 Hz), 4.20 – 4.10 (1 H, m), 4.08 (1 H, d, *J* = 6.3 Hz), 2.34 (3 H, s), 1.90 (1 H, d, *J* = 12.4 Hz), 1.82 – 1.69 (2 H, m), 1.66 (2 H, dq, *J* = 9.2, 2.9 Hz), 1.58 – 1.45 (1 H, m), 1.30 – 0.96 (5 H, m) ppm. – ^{13}C NMR (125 MHz, $CDCl_3$): 148.0, 142.8, 137.8, 129.2, 128.1, 128.0, 127.6, 124.5, 117.0, 113.3, 63.6, 45.1, 30.5, 29.6, 26.6, 26.5, 26.5, 21.7 ppm – IR: 2923, 2851, 1738, 1601, 1504, 1365, 1217 cm^{-1}. – HRMS: calcd for $C_{20}H_{25}N$: 280.2060, found 280.2053 [$M+H^+$].

Go to table of contents

N-((4-(*tert*-Butyl)phenyl)(cyclohexyl)methyl)aniline (5c)

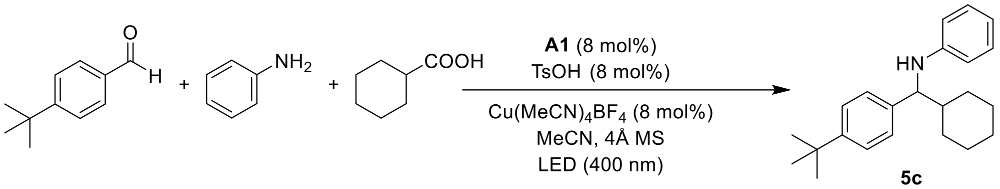

5c

According to GP1, the reaction was carried out with $Cu(MeCN)BF_4$ (5.0 mg, 0.016 mmol, 8 mol%), acridine **A1** (4.7 mg, 0.016 mmol, 8 mol %), 4Å molecular sieves (60 mg), aldehyde (32.4 mg, 0.2 mmol), aniline (22.3 mg, 0.24 mmol, 1.2 equiv.), carboxylic acid (33.3 mg, 0.26 mmol, 1.3 equiv.), anhydrous *p*-toluenesulfonic acid (2.8 mg, 0.016 mmol, 8 mol%), and acetonitrile (2 mL). The test-tube was capped and the reaction mixture was irradiated with LED light (λ = 400 nm) while stirring at at room temperature for 30 h. The reaction mixture was then concentrated under reduced pressure, and the remaining material was purified by flash chromatography on silica gel (EtOAc/hexane, 1 : 20 v/v) to give product **5c (**63.6 mg, 99%) as a colourless oil.

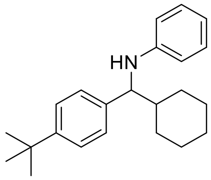

^{1}H NMR (500 MHz, $CDCl_3$): δ 7.31 (2 H, d, *J* = 8.3 Hz), 7.21 (2 H, d, *J* = 8.3 Hz), 7.09 (2 H, dd, *J* = 8.6, 7.3 Hz), 6.66 – 6.59 (1 H, m), 6.56 – 6.51 (2 H, m), 4.15 (1 H, s), 4.11 (1 H, d, *J* = 6.4 Hz), 1.92 (1 H, dt, *J* = 12.7, 1.8 Hz), 1.83 – 1.60 (4 H, m), 1.55 (1 H, ddd, *J* = 14.6, 4.6, 2.2 Hz), 1.32 (9 H, s), 1.27 – 0.98 (5 H, m) ppm. – ^{13}C NMR (125 MHz, $CDCl_3$): 149.5, 148.1, 139.6, 129.2, 126.9, 125.2, 116.9, 113.2, 63.1, 45.0, 34.5, 31.6, 30.4, 29.6, 26.6, 26.5, 26.5 ppm – IR: 2922, 2850, 1738, 1600, 1502, 1319, 1217, 747, 691 cm^{-1}. – HRMS: calcd for $C_{23}H_{31}N$: 322.2529, found 322.2524 [M+H$^+$].

N-(Cyclohexyl(2-fluorophenyl)methyl)aniline (5d)

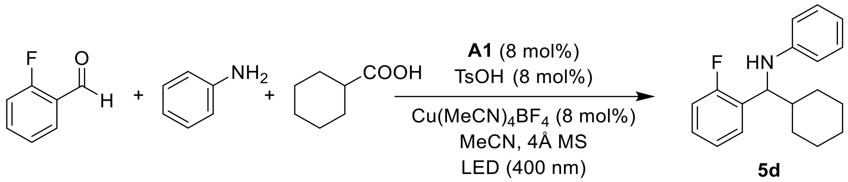

5d

According to GP1, the reaction was carried out with $Cu(MeCN)BF_4$ (5.0 mg, 0.016 mmol, 8 mol%), acridine **A1** (4.7 mg, 0.016 mmol, 8 mol %), 4Å molecular sieves (60 mg), aldehyde (24.8 mg, 0.2 mmol), aniline (22.3 mg, 0.24 mmol, 1.2 equiv.), carboxylic acid (33.3 mg, 0.26 mmol, 1.3 equiv.), anhydrous *p*-toluenesulfonic acid (2.8 mg, 0.016 mmol, 8 mol%), and acetonitrile (2 mL). The test-tube was capped and the reaction mixture was irradiated with LED light (λ = 400 nm) while stirring at at room temperature for 30 h. The reaction mixture was then concentrated under reduced pressure, and the remaining material was purified by flash chromatography on silica gel (EtOAc/hexane, 1 : 15 v/v) to give product **5d (**48.1 mg, 85%) as a colourless oil.

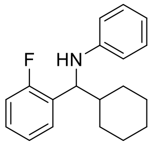

^{1}H NMR (500 MHz, $CDCl_3$): 7.35 – 7.24 (1 H, m), 7.18 (1 H, tdd, *J* = 7.5, 5.3, 1.8 Hz), 7.12 – 6.98 (4 H, m), 6.63 (1 H, dd, *J* = 7.9, 6.7 Hz), 6.56 – 6.52 (2 H, m), 4.48 (1 H, d, *J* = 7.2 Hz), 4.26 – 3.98 (1 H, m), 2.13 – 1.90 (1 H, m), 1.83 – 1.62 (4 H, m), 1.58 – 1.43 (1 H, m), 1.33 – 0.99 (5 H, m) ppm. – ^{13}C NMR (125 MHz, $CDCl_3$): 161.01 (d, *J* = 244.4 Hz), 147.43, 129.71, 129.14, 128.65 (d, *J* = 5.1 Hz), 128.19 (d, *J* = 8.3 Hz), 124.04 (d, *J* = 3.5 Hz), 117.18, 115.39, 115.21, 112.99, 57.02, 43.85, 29.98, 29.75, 26.42, 26.27 ppm. – ^{19}F NMR (376 MHz, $CDCl_3$) δ -118.8 (m) ppm. – IR: 2923, 2850, 1738, 1601, 1504, 1217, 749, 692 cm^{-1}. – HRMS: calcd for $C_{19}H_{22}FN$: 284.1809, found 284.1802 [M+H$^+$].

N-((4-Chlorophenyl)(cyclohexyl)methyl)aniline (5e)

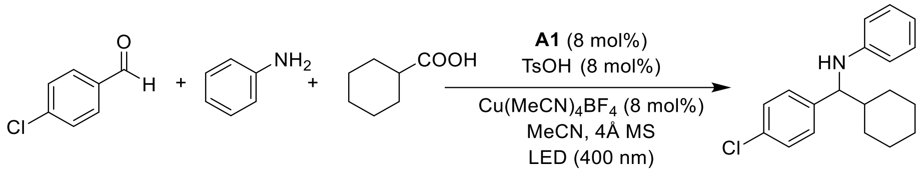

5e

According to GP1, the reaction was carried out with $Cu(MeCN)BF_4$ (5.0 mg, 0.016 mmol, 8 mol%), acridine **A1** (4.7 mg, 0.016 mmol, 8 mol %), 4Å molecular sieves (60 mg), aldehyde (28.0 mg, 0.2 mmol), aniline (22.3 mg, 0.24 mmol, 1.2 equiv.), carboxylic acid (33.3 mg, 0.26 mmol, 1.3 equiv.), anhydrous *p*-toluenesulfonic acid (2.8 mg, 0.016 mmol, 8 mol%), and acetonitrile (2 mL). The test-tube was capped and the reaction mixture was irradiated with LED light (λ = 400 nm) while stirring at at room temperature for 30 h. The reaction mixture was then concentrated under reduced pressure, and the remaining material was purified by flash chromatography on silica gel (EtOAc/hexane, 1 : 20 v/v) to give product **5e (**49.8 mg, 83%) as a white solid (m.p. 65 ^{o}C).

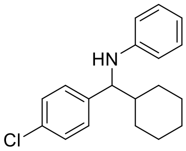

^{1}H NMR (500 MHz, $CDCl_3$): 7.34 – 7.19 (4 H, m), 7.09 (2 H, dd, *J* = 8.6, 7.2 Hz), 6.72 – 6.60 (1 H, m), 6.48 (2 H, d, *J* = 7.9 Hz), 4.14 (1 H, br), 4.11 (1 H, d, *J* = 6.1 Hz), 1.87 (1 H, dd, *J* = 13.4, 2.6 Hz), 1.83 – 1.72 (2 H, m), 1.71 – 1.48 (3 H, m), 1.31 – 0.98 (5 H, m) ppm. – ^{13}C NMR (125 MHz, $CDCl_3$): 132.5, 129.2, 128.7, 128.5, 117.4, 113.3, 63.0, 45.0, 30.2, 29.5, 26.5, 26.5, 26.4 ppm. – IR: 2925, 2851, 1601, 1504, 1152 cm^{-1}. – HRMS: calcd for $C_{19}H_{22}ClN$: 300.1514, found 300.1505 [M+H^{+}].

N-(Cyclohexyl(4-methoxyphenyl)methyl)aniline (5f)

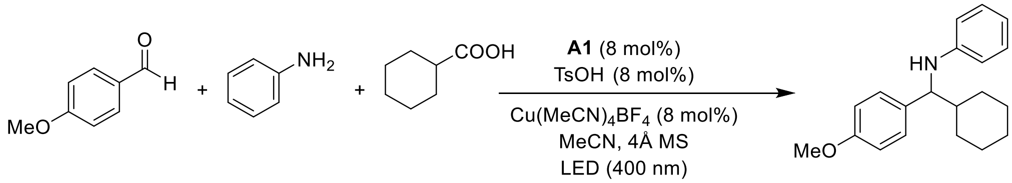

5f

According to GP1, the reaction was carried out with $Cu(MeCN)BF_4$ (5.0 mg, 0.016 mmol, 8 mol%), acridine **A1** (4.7 mg, 0.016 mmol, 8 mol %), 4Å molecular sieves (60 mg), aldehyde (27.2 mg, 0.2 mmol), aniline (22.3 mg, 0.24 mmol, 1.2 equiv.), carboxylic acid (33.3 mg, 0.26 mmol, 1.3 equiv.), anhydrous *p*-toluenesulfonic acid (2.8 mg, 0.016 mmol, 8 mol%), and acetonitrile (2 mL). The test-tube was capped and the reaction mixture was irradiated with LED light (λ = 400 nm) while stirring at at room temperature for 30 h. The reaction mixture was then concentrated under reduced pressure, and the remaining material was purified by flash chromatography on silica gel (EtOAc/hexane, 1 : 20 v/v) to give product **5f (**56.0 mg, 95%) as a colourless oil.

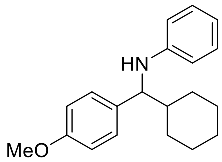

^{1}H NMR (500 MHz, $CDCl_3$): 7.22 (2 H, d, *J* = 8.7 Hz), 7.08 (2 H, dd, *J* = 8.6, 7.2 Hz), 6.85 (2 H, d, *J* = 8.7 Hz), 6.65 – 6.58 (1 H, m), 6.54 – 6.49 (2 H, m), 4.13 (1 H, s), 4.09 (1 H, d, *J* = 6.3 Hz), 3.79 (3 H, s), 1.91 (1 H, ddd, *J* = 13.0, 3.6, 1.9 Hz), 1.81 – 1.71 (2 H, m), 1.70 – 1.59 (2 H, m), 1.56 (1 H, ddt, *J* = 12.9, 3.4, 1.7 Hz), 1.29 – 0.98 (5 H, m) ppm. – ^{13}C NMR (125 MHz, $CDCl_3$): 158.5, 148.0, 134.7, 129.1, 128.4, 128.3, 117.0, 113.8, 113.7, 113.4, 62.9, 55.3, 45.1, 30.2, 29.7, 26.6, 26.5, 26.5 ppm. – IR: 2924, 2850,1739, 1601, 1505, 1243, 1034, 692 cm^{-1}. – HRMS: calcd for $C_{20}H_{25}NO$: 296.2009, found 296.2010 [M+H^{+}].

N-(Cyclohexyl(3,4,5-trimethoxyphenyl)methyl)aniline (5g)

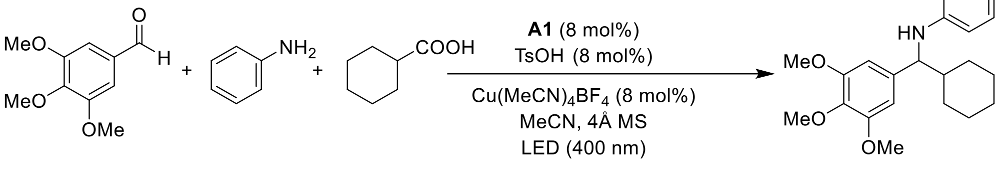

5g

According to GP1, the reaction was carried out with Cu(MeCN)BF4 (5.0 mg, 0.016 mmol, 8 mol%), acridine **A1** (4.7 mg, 0.016 mmol, 8 mol %), 4Å molecular sieves (60 mg), aldehyde (39.2 mg, 0.2 mmol), aniline (22.3 mg, 0.24 mmol, 1.2 equiv.), carboxylic acid (33.3 mg, 0.26 mmol, 1.3 equiv.), anhydrous *p*-toluenesulfonic acid (2.8 mg, 0.016 mmol, 8 mol%), and acetonitrile (2 mL). The test-tube was capped and the reaction mixture was irradiated with LED light (λ = 400 nm) while stirring at at room temperature for 30 h. The reaction mixture was then concentrated under reduced pressure, and the remaining material was purified by flash chromatography on silica gel (EtOAc/hexane, 1 : 20 v/v) to give product **5g (**66.7 mg, 94%) as a white solid (m.p. 111 °C).

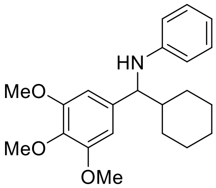

^{1}H NMR (500 MHz, $CDCl_3$): 7.08 (2 H, dd, *J* = 8.6, 7.2 Hz), 6.67 – 6.59 (1 H, m), 6.52 (4 H, d, *J* = 6.5 Hz), 4.08 (1 H, br), 4.00 (1 H, d, *J* = 6.2 Hz), 3.83 (9 H, d, *J* = 3.8 Hz), 1.88 (1 H, dd, *J* = 12.8, 3.1 Hz), 1.82 – 1.70 (2 H, m), 1.70 – 1.46 (3 H, m), 1.31 – 0.91 (5 H, m) ppm. – ^{13}C NMR (125 MHz, $CDCl_3$): 153.2, 148.0, 138.7, 136.7, 129.2, 117.2, 113.4, 104.1, 64.2, 60.9, 56.2, 45.2, 30.5, 29.6, 26.5, 26.5 ppm – IR: 2923, 1738, 1592, 1502, 1231, 1124, 749, 693 cm^{-1}. – HRMS: calcd for $C_{22}H_{29}NO_3$: 356.2220, found 356.2208 [M+H^{+}].

N-(4-(Cyclohexyl(phenylamino)methyl)phenyl)acetamide (5h)

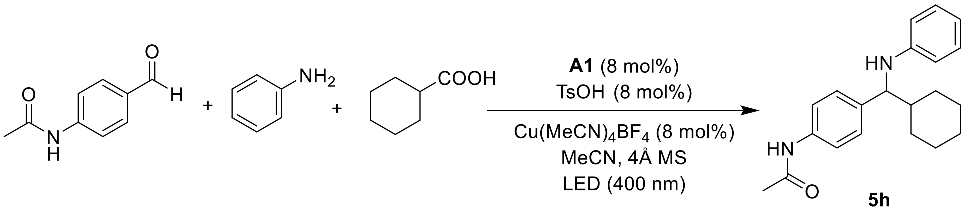

5h

According to GP1, the reaction was carried out with Cu(MeCN)BF4 (5.0 mg, 0.016 mmol, 8 mol%), acridine **A1** (4.7 mg, 0.016 mmol, 8 mol %), 4Å molecular sieves (60 mg), aldehyde (21.2 mg, 0.2 mmol), aniline (22.3 mg, 0.24 mmol, 1.2 equiv.), carboxylic acid (32.6 mg, 0.26 mmol, 1.3 equiv.), anhydrous *p*-toluenesulfonic acid (2.8 mg, 0.016 mmol, 8 mol%), and acetonitrile (2 mL). The test-tube was capped and the reaction mixture was irradiated with LED light (λ = 400 nm) while stirring at at room temperature for 30 h. The reaction mixture was then concentrated under reduced pressure, and the remaining material was purified by flash chromatography on silica gel (EtOAc/hexane, 1 : 20 v/v) to give product **5h (**63.1 mg, 98%) as a colourless oil.

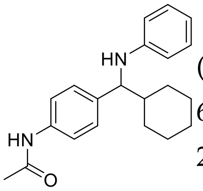

^{1}H NMR (500 MHz, $CDCl_3$): 7.41 (2 H, d, *J* = 8.5 Hz), 7.33 (1 H, s), 7.23 (2 H, d, *J* = 8.5 Hz), 7.05 (2 H, dd, *J* = 8.6, 7.2 Hz), 6.66 – 6.55 (1 H, m), 6.52 – 6.44 (2 H, m), 4.20 (1 H, s), 4.08 (1 H, d, *J* = 6.2 Hz), 2.13 (3 H, s), 1.91 – 1.82 (1 H, m), 1.81 – 1.68 (2 H, m), 1.63 (2 H, dddd, *J* = 20.5, 11.6, 4.8, 2.4 Hz), 1.57 – 1.49 (1 H, m), 1.31 – 0.91 (5 H, m) ppm. – ^{13}C NMR (125 MHz, $CDCl_3$): 168.4, 147.8, 138.7, 136.6, 129.2, 127.9, 119.9, 117.1, 113.3, 63.1, 45.0, 30.2, 29.6, 26.5, 26.5, 26.4, 24.6 ppm – IR: 2925, 1668, 1601, 1505, 1316, 749 cm^{-1}. – HRMS: calcd for $C_{21}H_{26}N_2O$: 323.2118, found 323.2111 [M+H^{+}].

N-(Cyclohexyl(4-(trifluoromethyl)phenyl)methyl)aniline (5i)

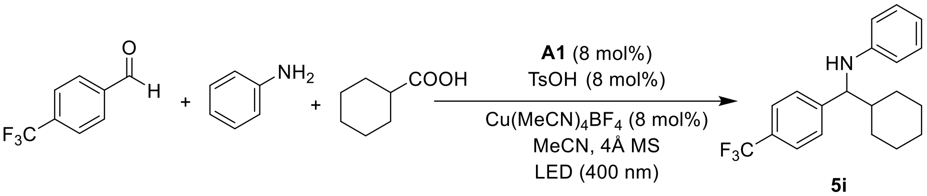

5i

According to GP1, the reaction was carried out with Cu(MeCN)BF_4 (5.0 mg, 0.016 mmol, 8 mol%), acridine **A1** (4.7 mg, 0.016 mmol, 8 mol %), 4Å molecular sieves (60 mg), aldehyde (34.8 mg, 0.2 mmol), aniline (22.3 mg, 0.24 mmol, 1.2 equiv.), carboxylic acid (33.3 mg, 0.26 mmol, 1.3 equiv.), anhydrous *p*-toluenesulfonic acid (2.8 mg, 0.016 mmol, 8 mol%), and acetonitrile (2 mL). The test-tube was capped and the reaction mixture was irradiated with LED light (λ = 400 nm) while stirring at at room temperature for 30 h. The reaction mixture was then concentrated under reduced pressure, and the remaining material was purified by flash chromatography on silica gel (EtOAc/hexane, 1 : 10 v/v) to give product **5i (**50.0 mg, 75%) as a colourless oil.

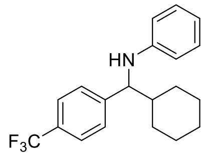

^{1}H NMR (500 MHz, $CDCl_3$): 7.56 (2 H, d, *J* = 8.0 Hz), 7.42 (2 H, d, *J* = 8.0 Hz), 7.07 (2 H, t, *J* = 7.9 Hz), 6.64 (1 H, t, *J* = 7.3 Hz), 6.46 (2 H, d, *J* = 7.9 Hz), 4.19 (1 H, d, *J* = 6.0 Hz), 1.92 – 1.81 (1 H, m), 1.81 – 1.70 (2 H, m), 1.71 – 1.64 (2 H, m), 1.55 (1 H, dd, *J* = 12.8, 3.6 Hz), 1.31 – 0.99 (5H, m) ppm. – ^{13}C NMR (125 MHz, $CDCl_3$): 147.3 (d, *J* = 35.1 Hz), 129.3, 127.7, 125.4 (d, *J* = 3.9 Hz), 117.5, 113.3, 63.3, 44.9, 30.3, 29.4, 26.4, 26.4 ppm. – ^{19}F NMR (376 MHz, $CDCl_3$) δ -62.3 ppm. – IR: 2927, 2853, 1738, 1602, 1325, 1217, 1067 cm^{-1}. – HRMS: calcd for $C_{20}H_{22}F_3N$: 334.1777, found 334.1777 [M+H$^+$].

N-(Cyclohexyl(naphthalen-2-yl)methyl)aniline (5j)

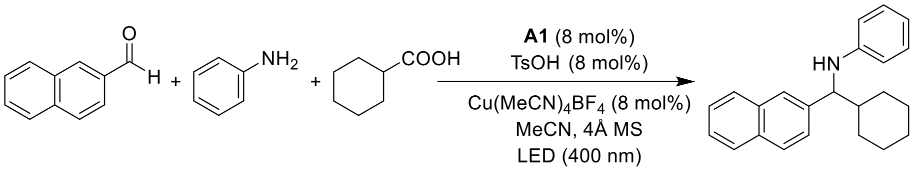

5j

According to GP1, the reaction was carried out with Cu(MeCN)BF_4 (5.0 mg, 0.016 mmol, 8 mol%), acridine **A1** (4.7 mg, 0.016 mmol, 8 mol %), 4Å molecular sieves (60 mg), aldehyde (31.2 mg, 0.2 mmol), aniline (22.3 mg, 0.24 mmol, 1.2 equiv.), carboxylic acid (33.3 mg, 0.26 mmol, 1.3 equiv.), anhydrous *p*-toluenesulfonic acid (2.8 mg, 0.016 mmol, 8 mol%), and acetonitrile (2 mL). The test-tube was capped and the reaction mixture was irradiated with LED light (λ = 400 nm) while stirring at at room temperature for 30 h. The reaction mixture was then concentrated under reduced pressure, and the remaining material was purified by flash chromatography on silica gel (EtOAc/hexane, 1 : 20 v/v) to give product **5j (**57.3 mg, 91%) as a colourless oil.

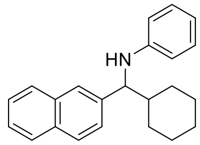

^{1}H NMR (500 MHz, $CDCl_3$): 7.89 – 7.76 (3 H, m), 7.74 (1 H, d, *J* = 1.6 Hz), 7.51 – 7.35 (3 H, m), 7.04 (2 H, dd, *J* = 8.6, 7.2 Hz), 6.59 (1 H, t, *J* = 7.3 Hz), 6.54 (2 H, d, *J* = 8.0 Hz), 4.28 (1 H, d, *J* = 6.2 Hz), 1.93 (1 H, d, *J* = 12.3 Hz), 1.84 – 1.47 (5 H, m), 1.33 – 1.00 (5 H, m) ppm. – ^{13}C NMR (125 MHz, $CDCl_3$): 133.4, 132.9, 129.2, 128.1, 128.0, 127.8, 126.2, 126.0, 125.6, 125.5, 117.2, 113.4, 63.8, 45.1, 30.5, 29.7, 26.5, 26.5 ppm – IR: 2924, 1738, 1601, 1503, 1365, 1228, 747, 691 cm^{-1}. – HRMS: calcd for $C_{23}H_{25}N$: 316.2060, found 316.2048 [M+H$^+$].

N-(Cyclohexyl(naphthalen-1-yl)methyl)aniline (5k)

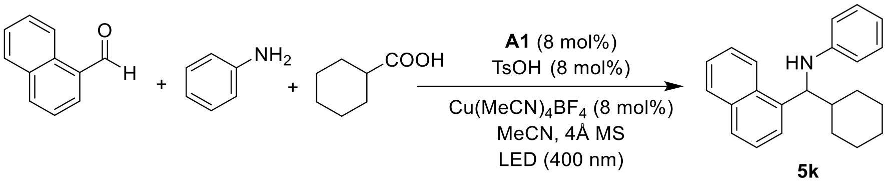

5k

According to GP1, the reaction was carried out with Cu(MeCN)BF_4 (5.0 mg, 0.016 mmol, 8 mol%), acridine **A1** (4.7 mg, 0.016 mmol, 8 mol %), 4Å molecular sieves (60 mg), aldehyde (31.2 mg, 0.2 mmol), aniline (22.3 mg, 0.24 mmol, 1.2 equiv.), carboxylic acid (33.3 mg, 0.26 mmol, 1.3 equiv.), anhydrous *p*-toluenesulfonic acid (2.8 mg, 0.016 mmol, 8 mol%), and acetonitrile (2 mL). The test-tube was capped and the reaction mixture was irradiated with LED light (λ = 400 nm) while stirring at at room temperature for 30 h. The reaction mixture was then concentrated under reduced pressure, and the remaining material was purified by flash chromatography on silica gel (EtOAc/hexane, 1 : 20 v/v) to give product **5k (**57.3 mg, 91%) as a colourless oil.

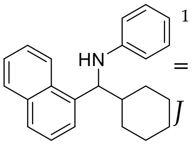

^{1}H NMR (500 MHz, $CDCl_3$): 8.21 (1 H, d, *J* = 8.4 Hz), 7.92 (1 H, dd, *J* = 8.1, 1.4 Hz), 7.74 (1 H, d, *J* = 8.1 Hz), 7.63 – 7.49 (3 H, m), 7.40 (1 H, t, *J* = 7.7 Hz), 7.02 (2 H, dd, *J* = 8.6, 7.2 Hz), 6.59 (1 H, dd, *J* = 7.9, 6.7 Hz), 6.47 (2 H, d, *J* = 8.0 Hz), 5.05 (1 H, d, *J* = 5.0 Hz), 4.32 (1 H, s), 1.90 (1 H, ddt, *J* = 11.6, 8.6, 4.3 Hz), 1.84 – 1.72 (3 H, m), 1.71 – 1.59 (2 H, m), 1.38 (1 H, qd, *J* = 12.4, 3.4 Hz), 1.27 – 1.05 (4 H, m) ppm. – ^{13}C NMR (125 MHz, $CDCl_3$): 147.9, 138.2, 134.2, 131.5, 129.4, 129.2, 127.4, 126.0, 125.6, 125.4, 124.1, 122.9, 117.1, 113.2, 58.8, 44.4, 31.4, 28.4, 26.8, 26.6 ppm – IR: 2925, 2850, 1738, 1601, 1365, 1217 cm^{-1}. – HRMS: calcd for $C_{23}H_{25}N$: 316.2060, found 316.2061 [M+H$^+$].

N-(Cyclohexyl(pyridin-4-yl)methyl)aniline (5l)

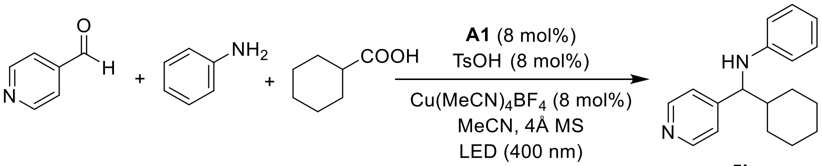

5l

According to GP1, the reaction was carried out with Cu(MeCN)BF_4 (5.0 mg, 0.016 mmol, 8 mol%), acridine **A1** (4.7 mg, 0.016 mmol, 8 mol %), 4Å molecular sieves (60 mg), aldehyde (21.4 mg, 0.2 mmol), aniline (22.3 mg, 0.24 mmol, 1.2 equiv.), carboxylic acid (33.3 mg, 0.26 mmol, 1.3 equiv.), anhydrous *p*-toluenesulfonic acid (2.8 mg, 0.016 mmol, 8 mol%), and acetonitrile (2 mL). The test-tube was capped and the reaction mixture was irradiated with LED light (λ = 400 nm) while stirring at at room temperature for 30 h. The reaction mixture was then concentrated under reduced pressure, and the remaining material was purified by flash chromatography on silica gel (EtOAc/hexane, 1 : 10 v/v) to give product **XX (**48.9 mg, 92%) as a white solid (m.p. 65 ^{o}C).

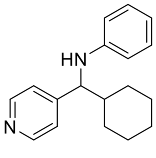

^{1}H NMR (500 MHz, $CDCl_3$): 8.62 – 8.40 (2 H, m), 7.38 – 7.16 (2 H, m), 7.07 (2 H, dd, *J* = 8.6, 7.2 Hz), 6.64 (1 H, t, *J* = 7.4 Hz), 6.53 – 6.36 (2 H, m), 4.16 (1 H, s), 4.13 (1 H, t, *J* = 4.5 Hz), 1.87 – 1.71 (3 H, m), 1.67 (2 H, dp, *J* = 11.8, 3.1 Hz), 1.61 – 1.49 (1 H, m), 1.29 – 1.01 (5 H, m) ppm. – ^{13}C NMR (125 MHz, $CDCl_3$): 152.2, 149.8, 147.2, 129.3, 122.7, 117.7, 113.2, 62.7, 44.5, 30.2, 29.1, 26.4 ppm – IR: 2925, 2851, 1738, 1599, 1365, 1216, 693 cm^{-1}. – HRMS: calcd for $C_{18}H_{22}N_2$: 267.1856, found 267.1851 [M+H$^+$].

N-(Cyclohexyl(6-methylpyridin-2-yl)methyl)aniline (5m)

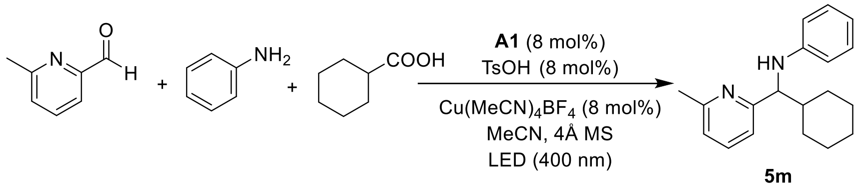

5m

According to GP1, the reaction was carried out with Cu(MeCN)BF4 (5.0 mg, 0.016 mmol, 8 mol%), acridine **A1** (4.7 mg, 0.016 mmol, 8 mol %), 4Å molecular sieves (60 mg), aldehyde (24.2 mg, 0.2 mmol), aniline (22.3 mg, 0.24 mmol, 1.2 equiv.), carboxylic acid (33.3 mg, 0.26 mmol, 1.3 equiv.), anhydrous *p*-toluenesulfonic acid (2.8 mg, 0.016 mmol, 8 mol%), and acetonitrile (2 mL). The test-tube was capped and the reaction mixture was irradiated with LED light (λ = 400 nm) while stirring at at room temperature for 30 h. The reaction mixture was then concentrated under reduced pressure, and the remaining material was purified by flash chromatography on silica gel (EtOAc/hexane, 1 : 10 v/v) to give product **5m (**53.8 mg, 96%) as a white solid (m.p. 66 ^{o}C).

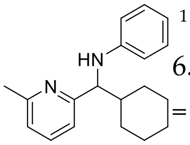

^{1}H NMR (500 MHz, $CDCl_3$): 7.44 (1 H, t, *J* = 7.7 Hz), 7.13 – 7.06 (2 H, m), 7.03 (1 H, d, *J* = 7.7 Hz), 6.96 (1 H, d, *J* = 7.6 Hz), 6.66 – 6.59 (1 H, m), 6.59 – 6.54 (2 H, m), 5.07 – 4.40 (1 H, m), 4.25 (1 H, t, *J* = 5.4 Hz), 1.95 – 1.60 (6 H, m), 1.58 – 1.46 (1 H, m), 1.32 – 0.98 (5 H, m) ppm. – ^{13}C NMR (125 MHz, $CDCl_3$): 161.4, 157.9, 148.1, 136.3, 129.2, 121.4, 119.0, 117.0, 113.4, 64.3, 43.8, 30.5, 29.0, 26.6, 26.5, 26.4, 24.8 ppm – IR: 2924, 2850, 1738, 1602, 1365, 1217 cm^{-1}. – HRMS: calcd for $C_{19}H_{24}N_2$: 281.2012, found 281.2007 [M+H^{+}].

N-(Benzofuran-2-yl(cyclohexyl)methyl)aniline (5n)

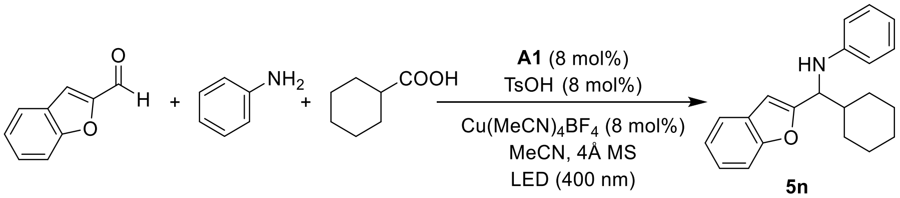

5n

According to GP1, the reaction was carried out with Cu(MeCN)BF4 (5.0 mg, 0.016 mmol, 8 mol%), acridine **A1** (4.7 mg, 0.016 mmol, 8 mol %), 4Å molecular sieves (60 mg), aldehyde (29.2 mg, 0.2 mmol), aniline (22.3 mg, 0.24 mmol, 1.2 equiv.), carboxylic acid (33.3 mg, 0.26 mmol, 1.3 equiv.), anhydrous *p*-toluenesulfonic acid (2.8 mg, 0.016 mmol, 8 mol%), and acetonitrile (2 mL). The test-tube was capped and the reaction mixture was irradiated with LED light (λ = 400 nm) while stirring at at room temperature for 30 h. The reaction mixture was then concentrated under reduced pressure, and the remaining material was purified by flash chromatography on silica gel (EtOAc/hexane, 1 : 15 v/v) to give product **5n (**43.3 mg, 71%) as a white solid (m.p. 90 ^{o}C).

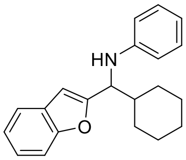

^{1}H NMR (500 MHz, $CDCl_3$): 7.50 – 7.41 (2 H, m), 7.23 (1 H, td, *J* = 7.7, 1.5 Hz), 7.18 (1 H, t, *J* = 7.4 Hz), 7.13 (2 H, dd, *J* = 8.5, 7.2 Hz), 6.68 (1 H, t, *J* = 7.3 Hz), 6.64 (2 H, d, *J* = 8.0 Hz), 6.53 (1 H, s), 4.39 (1 H, d, *J* = 6.3 Hz), 4.09 (1 H, s), 1.96 (2 H, dt, *J* = 11.1, 3.3 Hz), 1.86 – 1.71 (2 H, m), 1.71 – 1.57 (2 H, m), 1.36 – 1.08 (5 H, m) ppm. – ^{13}C NMR (125 MHz, $CDCl_3$): 158.4, 154.8, 147.5, 129.3, 128.5, 123.6, 122.7, 120.8, 117.8, 113.5, 111.2, 103.9, 57.7, 42.6, 30.0, 29.6, 26.5, 26.4, 26.3 ppm – IR: 2926, 1738, 1365, 1216 cm^{-1}. – HRMS: calcd for $C_{21}H_{23}NO$: 306.1852, found 306.1847 [M+H^{+}].

tert-Butyl 5-(Cyclohexyl(phenylamino)methyl)-1*H*-indole-1-carboxylate (5o)

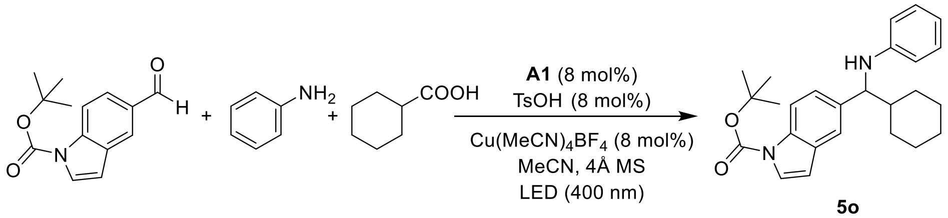

5o

According to GP1, the reaction was carried out with Cu(MeCN)BF4 (5.0 mg, 0.016 mmol, 8 mol%), acridine **A1** (4.7 mg, 0.016 mmol, 8 mol %), 4Å molecular sieves (60 mg), aldehyde (49.0 mg, 0.2 mmol), aniline (22.3 mg, 0.24 mmol, 1.2 equiv.), carboxylic acid (33.3 mg, 0.26 mmol, 1.3 equiv.), anhydrous *p*-toluenesulfonic acid (2.8 mg, 0.016 mmol, 8 mol%), and acetonitrile (2 mL). The test-tube was capped and the reaction mixture was irradiated with LED light (*λ* = 400 nm) while stirring at at room temperature for 30 h. The reaction mixture was then concentrated under reduced pressure, and the remaining material was purified by flash chromatography on silica gel (EtOAc/hexane, 1 : 5 v/v) to give product **5o (**66.2 mg, 82%) as a white solid.

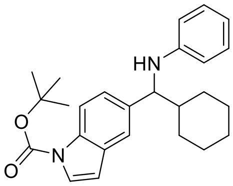

^{1}H NMR (500 MHz, $CDCl_3$): 8.05 (1 H, d, *J* = 8.1 Hz), 7.57 (1 H, d, *J* = 3.7 Hz), 7.48 (1 H, d, *J* = 1.7 Hz), 7.32 – 7.18 (1 H, m), 7.04 (2 H, dd, *J* = 8.6, 7.3 Hz), 6.59 (1 H, d, *J* = 7.3 Hz), 6.54 – 6.49 (3 H, m), 4.20 (1 H, d, *J* = 6.2 Hz), 1.92 (1 H, d, *J* = 12.8 Hz), 1.66 (12H, m), 1.60 – 1.46 (2 H, m), 1.33 – 0.94 (5 H, m) ppm. – ^{13}C NMR (125 MHz, $CDCl_3$): 149.9, 148.0, 137.2, 134.4, 130.7, 129.1, 126.2, 123.8, 119.6, 117.0, 114.9, 113.4, 107.5, 83.7, 63.6, 45.4, 30.4, 29.8, 28.3, 26.6, 26.5 ppm – IR: 2925, 2851, 1731, 1601, 1353, 1162, 692 cm^{-1}. – HRMS: calcd for $C_{26}H_{32}N_2O_2$: 405.2537, found 405.2536 [M+H$^+$].

1-(5-(Cyclohexyl(phenylamino)methyl)-1*H*-indol-1-yl)ethan-1-one (5p)

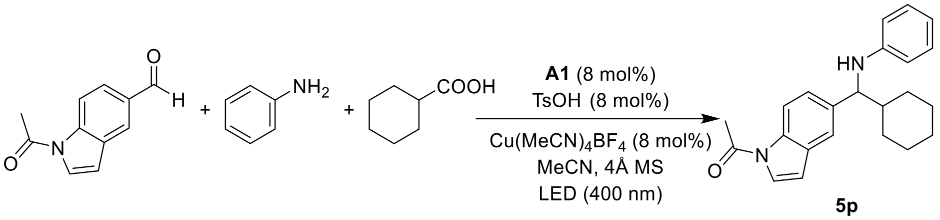

5p

According to GP1, the reaction was carried out with Cu(MeCN)BF4 (5.0 mg, 0.016 mmol, 8 mol%), acridine **A1** (4.7 mg, 0.016 mmol, 8 mol %), 4Å molecular sieves (60 mg), aldehyde (37.4 mg, 0.2 mmol), aniline (22.3 mg, 0.24 mmol, 1.2 equiv.), carboxylic acid (33.3 mg, 0.26 mmol, 1.3 equiv.), anhydrous *p*-toluenesulfonic acid (2.8 mg, 0.016 mmol, 8 mol%), and acetonitrile (2 mL). The test-tube was capped and the reaction mixture was irradiated with LED light (*λ* = 400 nm) while stirring at at room temperature for 30 h. The reaction mixture was then concentrated under reduced pressure, and the remaining material was purified by flash chromatography on silica gel (EtOAc/hexane, 1 : 20 v/v) to give product **5p (**56.7 mg, 82%) as a white solid.

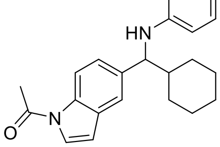

^{1}H NMR (500 MHz, $CDCl_3$): 8.35 (1 H, d, *J* = 8.5 Hz), 7.49 (1 H, d, *J* = 1.6 Hz), 7.38 (1 H, d, *J* = 3.7 Hz), 7.30 (1 H, dd, *J* = 8.5, 1.7 Hz), 7.04 (2 H, dd, *J* = 8.6, 7.2 Hz), 6.66 – 6.56 (2 H, m), 6.52 (2 H, d, *J* = 7.9 Hz), 4.21 (1 H, d, *J* = 6.2 Hz), 2.61 (3 H, s), 1.91 (1 H, d, *J* = 12.6 Hz), 1.81 – 1.60 (4 H, m), 1.55 (1 H, dt, *J* = 12.9, 3.2 Hz), 1.35 – 0.97 (5 H, m) ppm. – ^{13}C NMR (125 MHz, $CDCl_3$): 168.6, 147.9, 138.3, 134.8, 130.6, 129.2, 125.5, 124.8, 119.4, 117.1, 116.3, 113.4, 109.4, 63.6, 45.3, 30.4, 29.7, 26.6, 26.5, 26.5, 24.0 ppm – IR: 2926, 1738, 1365, 1217 cm^{-1}. – HRMS: calcd for $C_{23}H_{26}N_2O$: 347.2118, found 347.2114 [M+H$^+$].

N-(1-Phenylpentyl)aniline (6a)

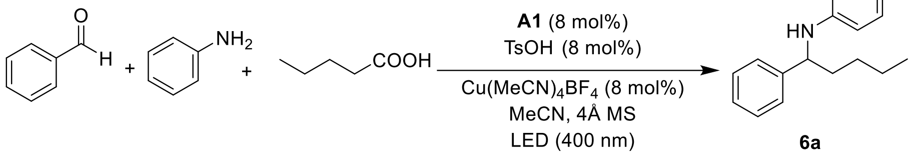

According to GP1, the reaction was carried out with Cu(MeCN)BF$_4$ (5.0 mg, 0.016 mmol, 8 mol%), acridine **A1** (4.7 mg, 0.016 mmol, 8 mol %), 4Å molecular sieves (60 mg), aldehyde (21.2 mg, 0.2 mmol), aniline (22.3 mg, 0.24 mmol, 1.2 equiv.), carboxylic acid (26.5 mg, 0.26 mmol, 1.3 equiv.), anhydrous *p*-toluenesulfonic acid (2.8 mg, 0.016 mmol, 8 mol%), and acetonitrile (2 mL). The test-tube was capped and the reaction mixture was irradiated with LED light (λ = 400 nm) while stirring at at room temperature for 30 h. The reaction mixture was then concentrated under reduced pressure, and the remaining material was purified by flash chromatography on silica gel (EtOAc/hexane, 1 : 20 v/v) to give product **6a (**21.7 mg, 46%) as a colourless oil.

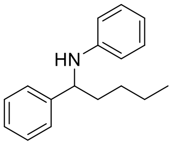

^{1}H NMR (500 MHz, CDCl$_3$): 7.43 – 7.27 (4 H, m), 7.25 – 7.18 (1 H, m), 7.08 (2 H, dd, *J* = 8.6, 7.3 Hz), 6.68 – 6.56 (1 H, m), 6.56 – 6.41 (2 H, m), 4.29 (1 H, t, *J* = 6.8 Hz), 4.08 (1 H, s), 1.94 – 1.70 (2 H, m), 1.47 – 1.22 (3 H, m), 0.89 (3 H, t, *J* = 7.0 Hz) ppm. – ^{13}C NMR (125 MHz, CDCl$_3$): 147.6, 144.5, 129.2, 128.7, 127.0, 126.5, 117.2, 113.3, 58.4, 38.9, 28.7, 22.7, 14.1 ppm – IR: 2928, 1738, 1601, 1504, 1217 cm^{-1}. – HRMS: calcd for $C_{17}H_{21}N$: 240.1747, found 240.1747 [M+H$^+$].

N-(2-Methyl-1-phenylpropyl)aniline (6b)

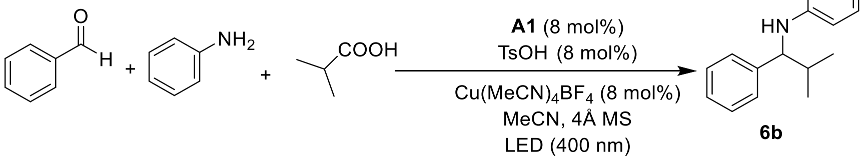

According to GP1, the reaction was carried out with Cu(MeCN)BF$_4$ (5.0 mg, 0.016 mmol, 8 mol%), acridine **A1** (4.7 mg, 0.016 mmol, 8 mol %), 4Å molecular sieves (60 mg), aldehyde (21.2 mg, 0.2 mmol), aniline (22.3 mg, 0.24 mmol, 1.2 equiv.), carboxylic acid (22.9 mg, 0.26 mmol, 1.3 equiv.), anhydrous *p*-toluenesulfonic acid (2.8 mg, 0.016 mmol, 8 mol%), and acetonitrile (2 mL). The test-tube was capped and the reaction mixture was irradiated with LED light (λ = 400 nm) while stirring at at room temperature for 30 h. The reaction mixture was then concentrated under reduced pressure, and the remaining material was purified by flash chromatography on silica gel (EtOAc/hexane, 1 : 20 v/v) to give product **6b (**44.1 mg, 98%) as a colourless oil.

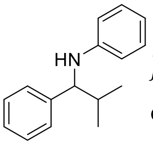

^{1}H NMR (500 MHz, CDCl$_3$): 7.36 – 7.28 (4 H, m), 7.22 (1 H, ddd, *J* = 8.7, 4.8, 3.4 Hz), 7.08 (2 H, dd, *J* = 8.6, 7.2 Hz), 6.62 (1 H, tt, *J* = 7.3, 1.2 Hz), 6.55 – 6.49 (2 H, m), 4.14 (2 H, d, *J* = 5.9 Hz), 2.05 (1 H, dq, *J* = 13.4, 6.7 Hz), 1.00 (3 H, d, *J* = 6.8 Hz), 0.94 (3 H, d, *J* = 6.9 Hz) ppm. – ^{13}C NMR (125 MHz, CDCl$_3$): 147.8, 142.7, 129.2, 128.3, 127.3, 126.9, 117.1, 113.4, 63.9, 35.0, 19.9, 18.8 ppm – IR: 2970, 1738, 1365, 1217 cm^{-1}. – HRMS: calcd for $C_{16}H_{19}N$: 226.1590, found 226.1586 [M+H$^+$].

N-(1-Phenyl-2-propylpentyl)aniline (6c)

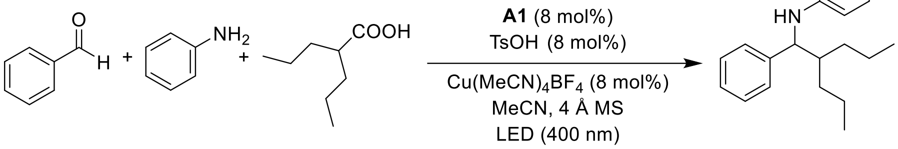

6c

According to GP1, the reaction was carried out with Cu(MeCN)BF_4 (5.0 mg, 0.016 mmol, 8 mol%), acridine **A1** (4.7 mg, 0.016 mmol, 8 mol %), 4Å molecular sieves (60 mg), aldehyde (21.2 mg, 0.2 mmol), aniline (22.3 mg, 0.24 mmol, 1.2 equiv.), carboxylic acid (37.4 mg, 0.26 mmol, 1.3 equiv.), anhydrous *p*-toluenesulfonic acid (2.8 mg, 0.016 mmol, 8 mol%), and acetonitrile (2 mL). The test-tube was capped and the reaction mixture was irradiated with LED light (λ = 400 nm) while stirring at at room temperature for 30 h. The reaction mixture was then concentrated under reduced pressure, and the remaining material was purified by flash chromatography on silica gel (EtOAc/hexane, 1 : 20 v/v) to give product **6c (**46.6 mg, 83%) as a colourless oil.

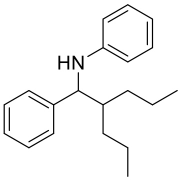

^{1}H NMR (500 MHz, $CDCl_3$): 7.30 (4 H, d, *J* = 4.3 Hz), 7.21 (1 H, h, *J* = 4.4, 3.9 Hz), 7.08 (2 H, dd, *J* = 8.6, 7.2 Hz), 6.67 – 6.59 (1 H, m), 6.53 – 6.45 (2 H, m), 4.43 (1 H, d, *J* = 4.6 Hz), 4.08 (1 H, s), 1.77 (1 H, tt, *J* = 7.5, 2.8 Hz), 1.48 – 1.04 (7 H, m), 0.90 (3 H, t, *J* = 7.0 Hz), 0.81 (3 H, t, *J* = 6.7 Hz) ppm. – ^{13}C NMR (125 MHz, $CDCl_3$): 147.9, 143.0, 129.2, 128.3, 127.2, 126.6, 117.0, 113.2, 59.6, 44.8, 33.4, 31.4, 20.8, 20.8, 14.5, 14.4 ppm – IR: 2955, 1739, 1504, 1217 cm^{-1}. – HRMS: calcd for $C_{20}H_{27}N$: 282.2216, found 282.2213 [M+H$^+$].

N-(2-Hexyl-1-phenyldecyl)aniline (6d)

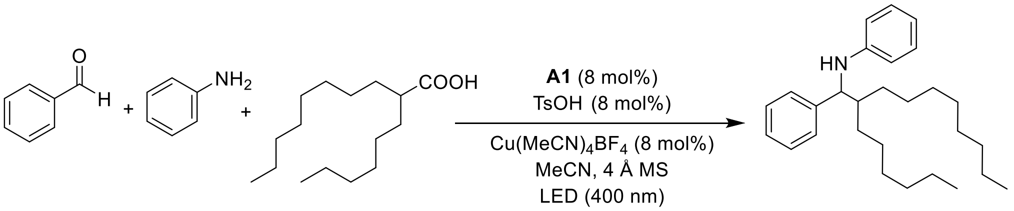

6d

According to GP1, the reaction was carried out with Cu(MeCN)BF_4 (5.0 mg, 0.016 mmol, 8 mol%), acridine **A1** (4.7 mg, 0.016 mmol, 8 mol %), 4Å molecular sieves (60 mg), aldehyde (21.2 mg, 0.2 mmol), aniline (22.3 mg, 0.24 mmol, 1.2 equiv.), carboxylic acid (66.6 mg, 0.26 mmol, 1.3 equiv.), anhydrous *p*-toluenesulfonic acid (2.8 mg, 0.016 mmol, 8 mol%), and acetonitrile (2 mL). The test-tube was capped and the reaction mixture was irradiated with LED light (λ = 400 nm) while stirring at at room temperature for 30 h. The reaction mixture was then concentrated under reduced pressure, and the remaining material was purified by flash chromatography on silica gel (EtOAc/hexane, 1 : 20 v/v) to give product **6d (**62.9 mg, 1:1 dr, 80%) as a colourless oil.

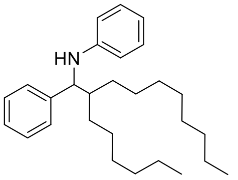

^{1}H NMR (500 MHz, $CDCl_3$): 7.33 (4 H, d, *J* = 4.4 Hz), 7.24 (1 H, dq, *J* = 8.6, 4.2 Hz), 7.11 (2 H, dd, *J* = 8.5, 7.2 Hz), 6.65 (1 H, t, *J* = 7.4 Hz), 6.52 (2 H, d, *J* = 7.9 Hz), 4.45 (1 H, d, *J* = 4.5 Hz), 4.10 (1 H, s), 1.75 (1 H, q, *J* = 6.1, 5.3 Hz), 1.50 – 1.11 (24 H, m), 0.99 – 0.80 (6 H, m) ppm. – ^{13}C NMR (125 MHz, $CDCl_3$): 147.9, 143.0, 129.2, 128.3, 127.2, 126.6, 117.0, 113.3, 59.6, 45.3, 32.0, 32.0, 31.8, 31.1, 30.0, 29.9, 29.7, 29.6, 29.5, 29.4, 29.4, 29.1, 27.7, 27.6, 22.8, 22.7, 14.3, 14.2 ppm – IR: 2922, 2852, 1601, 1503, 1317, 701 cm^{-1}. – HRMS: calcd for $C_{28}H_{43}N$: 394.3468, found 394.3468 [M+H$^+$].

N-(Cyclobutyl(phenyl)methyl)aniline (6e)

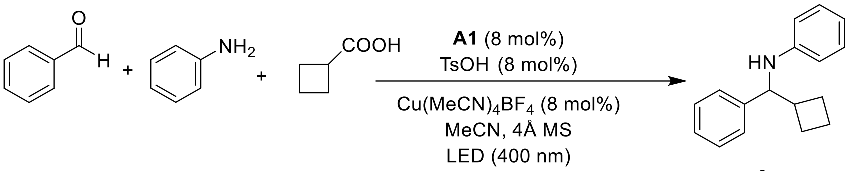

6e

According to GP1, the reaction was carried out with $Cu(MeCN)BF_4$ (5.0 mg, 0.016 mmol, 8 mol%), acridine **A1** (4.7 mg, 0.016 mmol, 8 mol %), 4Å molecular sieves (60 mg), aldehyde (21.2 mg, 0.2 mmol), aniline (22.3 mg, 0.24 mmol, 1.2 equiv.), carboxylic acid (26.0 mg, 0.26 mmol, 1.3 equiv.), anhydrous *p*-toluenesulfonic acid (2.8 mg, 0.016 mmol, 8 mol%), and acetonitrile (2 mL). The test-tube was capped and the reaction mixture was irradiated with LED light (λ = 400 nm) while stirring at at room temperature for 30 h. The reaction mixture was then concentrated under reduced pressure, and the remaining material was purified by flash chromatography on silica gel (EtOAc/hexane, 1 : 20 v/v) to give product **6e (**38.4 mg, 81%) as a colourless oil.

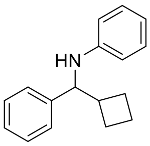

^{1}H NMR (500 MHz, $CDCl_3$): 7.38 – 7.28 (4 H, m), 7.27 – 7.18 (1 H, m), 7.08 (2 H, dd, *J* = 8.6, 7.2 Hz), 6.67 – 6.60 (1 H, m), 6.57 – 6.48 (2 H, m), 4.19 (1 H, d, *J* = 9.1 Hz), 4.03 (1 H, s), 2.65 – 2.47 (1 H, m), 2.25 – 2.08 (1 H, m), 2.01 – 1.73 (5 H, m) ppm. – ^{13}C NMR (125 MHz, $CDCl_3$): 147.8, 142.6, 129.2, 128.5, 127.0, 126.7, 117.3, 113.5, 63.9, 42.7, 26.2, 25.6, 17.7 ppm – IR: 2970, 1739, 1601, 1482, 1217, 701 cm^{-1}. – HRMS: calcd for $C_{17}H_{19}N$: 238.1590, found 238.1591 [$M+H^+$].

N-(Cyclopentyl(phenyl)methyl)aniline (6f)

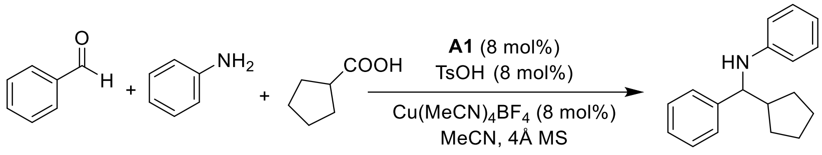

6f

According to GP1, the reaction was carried out with $Cu(MeCN)BF_4$ (5.0 mg, 0.016 mmol, 8 mol%), acridine **A1** (4.7 mg, 0.016 mmol, 8 mol %), 4Å molecular sieves (60 mg), aldehyde (21.2 mg, 0.2 mmol), aniline (22.3 mg, 0.24 mmol, 1.2 equiv.), carboxylic acid (29.6 mg, 0.26 mmol, 1.3 equiv.), anhydrous *p*-toluenesulfonic acid (2.8 mg, 0.016 mmol, 8 mol%), and acetonitrile (2 mL). The test-tube was capped and the reaction mixture was irradiated with LED light (λ = 400 nm) while stirring at at room temperature for 30 h. The reaction mixture was then concentrated under reduced pressure, and the remaining material was purified by flash chromatography on silica gel (EtOAc/hexane, 1 : 20 v/v) to give product **6f (**42.7 mg, 85%) as a colourless oil.

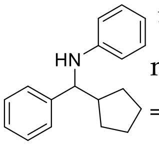

^{1}H NMR (500 MHz, $CDCl_3$): δ 7.39 – 7.33 (2 H, m), 7.30 (2 H, dd, *J* = 8.4, 6.8 Hz), 7.25 – 7.17 (1 H, m), 7.12 – 7.01 (2 H, m), 6.62 (1 H, tt, *J* = 7.3, 1.2 Hz), 6.56 – 6.49 (2 H, m), 4.20 (1 H, s), 4.10 (1 H, d, *J* = 8.4 Hz), 2.33 – 2.05 (1 H, m), 2.01 – 1.83 (1 H, m), 1.78 – 1.56 (3 H, m), 1.57 – 1.38 (3 H, m), 1.37 – 1.22 (1 H, m) ppm. – ^{13}C NMR (125 MHz, $CDCl_3$): 147.8, 144.1, 129.2, 128.4, 127.1, 126.9, 117.1, 113.4, 63.2, 47.9, 30.3, 30.1, 25.4, 25.3 ppm – IR: 2951, 1739, 1602, 1365, 1217 cm^{-1}. – HRMS: calcd for $C_{18}H_{21}N$: 252.1747, found 252.1747 [$M+H^+$].

N-(Cycloheptyl(phenyl)methyl)aniline (6g)

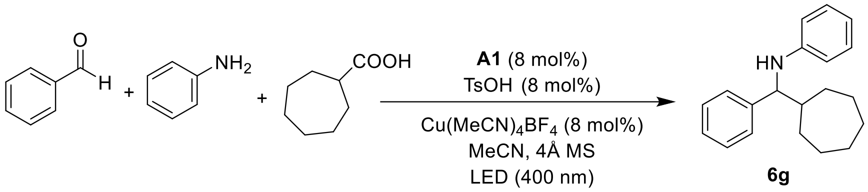

According to GP1, the reaction was carried out with Cu(MeCN)BF4 (5.0 mg, 0.016 mmol, 8 mol%), acridine **A1** (4.7 mg, 0.016 mmol, 8 mol %), 4Å molecular sieves (60 mg), aldehyde (21.2 mg, 0.2 mmol), aniline (22.3 mg, 0.24 mmol, 1.2 equiv.), carboxylic acid (36.9 mg, 0.26 mmol, 1.3 equiv.), anhydrous *p*-toluenesulfonic acid (2.8 mg, 0.016 mmol, 8 mol%), and acetonitrile (2 mL). The test-tube was capped and the reaction mixture was irradiated with LED light (λ = 400 nm) while stirring at at room temperature for 30 h. The reaction mixture was then concentrated under reduced pressure, and the remaining material was purified by flash chromatography on silica gel (EtOAc/hexane, 1 : 20 v/v) to give product **6g (**49.1 mg, 88%) as a colourless oil.

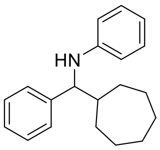

^{1}H NMR (500 MHz, $CDCl_3$): 7.37 – 7.27 (4 H, m), 7.26 – 7.19 (1 H, m), 7.09 (2 H, dd, J = 8.6, 7.2 Hz), 6.63 (1 H, t, J = 7.3 Hz), 6.55 – 6.47 (2 H, m), 4.25 (1 H, d, J = 5.4 Hz), 4.11 (1 H, s), 1.92 (1 H, dtt, J = 9.2, 6.9, 2.7 Hz), 1.81 (1 H, ddd, J = 10.8, 7.8, 4.1 Hz), 1.76 – 1.65 (3 H, m), 1.64 – 1.29 (8 H, m) ppm. – ^{13}C NMR (125 MHz, $CDCl_3$):148.0, 143.0, 129.2, 128.3, 127.3, 126.8, 117.0, 113.3, 63.8, 46.5, 32.4, 29.5, 28.4, 28.1, 27.1 ppm – IR: 2921, 1738, 1503, 1217, 701 cm^{-1}. – HRMS: calcd for $C_{20}H_{25}N$: 280.2060, found 280.2060 [M+H$^+$].

N-(Phenyl(tetrahydro-2H-pyran-4-yl)methyl)aniline (6h)

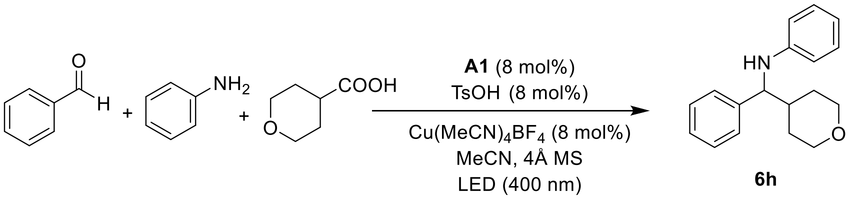

According to GP1, the reaction was carried out with Cu(MeCN)BF4 (5.0 mg, 0.016 mmol, 8 mol%), acridine **A1** (4.7 mg, 0.016 mmol, 8 mol %), 4Å molecular sieves (60 mg), aldehyde (21.2 mg, 0.2 mmol), aniline (22.3 mg, 0.24 mmol, 1.2 equiv.), carboxylic acid (33.8 mg, 0.26 mmol, 1.3 equiv.), anhydrous *p*-toluenesulfonic acid (2.8 mg, 0.016 mmol, 8 mol%), and acetonitrile (2 mL). The test-tube was capped and the reaction mixture was irradiated with LED light (λ = 400 nm) while stirring at at room temperature for 30 h. The reaction mixture was then concentrated under reduced pressure, and the remaining material was purified by flash chromatography on silica gel (EtOAc/hexane, 1 : 5 v/v) to give product **6h (**51.3 mg, 96%) as a white solid (m.p. 85 ^{o}C).

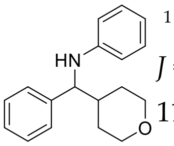

^{1}H NMR (500 MHz, $CDCl_3$): 7.36 – 7.28 (4 H, m), 7.24 (1 H, ddt, J = 10.9, 5.7, 2.6 Hz), 7.09 (2 H, dd, J = 8.6, 7.2 Hz), 6.69 – 6.61 (1 H, m), 6.57 – 6.50 (2 H, m), 4.15 (2 H, d, J = 7.0 Hz), 4.03 (1 H, ddd, J = 11.5, 4.9, 1.6 Hz), 3.96 (1 H, ddd, J = 11.6, 4.8, 1.8 Hz), 3.35 (2 H, dtd, J = 24.1, 11.9, 2.3 Hz), 2.02 – 1.74 (2 H, m), 1.61 – 1.41 (2 H, m), 1.39 – 1.25 (1 H, m). ppm. – ^{13}C NMR (125 MHz, $CDCl_3$): 147.5, 141.9, 129.2, 128.5, 127.2, 117.4, 113.4, 68.1, 68.0, 63.1, 42.4, 30.4, 29.9 ppm – IR: 2940, 1725, 1576, 1237, 824 cm^{-1}. – HRMS: calcd for $C_{18}H_{21}NO$: 268.1696, found 268.1689 [M+H$^+$].

N-(2,2-Dimethyl-1-phenylpropyl)aniline (6i)

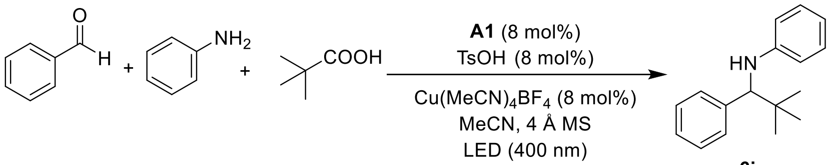

6i

According to GP1, the reaction was carried out with $Cu(MeCN)BF_4$ (5.0 mg, 0.016 mmol, 8 mol%), acridine **A1** (4.7 mg, 0.016 mmol, 8 mol %), 4Å molecular sieves (60 mg), aldehyde (21.2 mg, 0.2 mmol), aniline (22.3 mg, 0.24 mmol, 1.2 equiv.), carboxylic acid (26.5 mg, 0.26 mmol, 1.3 equiv.), anhydrous *p*-toluenesulfonic acid (2.8 mg, 0.016 mmol, 8 mol%), and acetonitrile (2 mL). The test-tube was capped and the reaction mixture was irradiated with LED light (λ = 400 nm) while stirring at at room temperature for 30 h. The reaction mixture was then concentrated under reduced pressure, and the remaining material was purified by flash chromatography on silica gel (EtOAc/hexane, 1 : 20 v/v) to give product **6i (**47.3 mg, 99%) as a colourless oil.

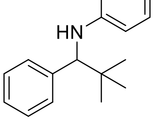

^{1}H NMR (500 MHz, $CDCl_3$): 7.34 – 7.25 (4 H, m), 7.24 – 7.16 (1 H, m), 7.05 (2 H, dd, *J* = 8.6, 7.2 Hz), 6.63 – 6.55 (1 H, m), 6.49 (2 H, dd, *J* = 8.7, 1.1 Hz), 4.27 (1 H, s), 4.05 (1 H, s), 1.01 (9 H, s) ppm. – ^{13}C NMR (125 MHz, $CDCl_3$):147.9, 141.3, 129.2, 128.6, 127.8, 126.9, 117.0, 113.3, 67.3, 35.1, 27.2 ppm – IR: 2969, 1739, 1504, 1366, 1217 cm^{-1}. – HRMS: calcd for $C_{17}H_{21}N$: 240.1747, found 240.1747 [$M+H^+$].

N-(2,2-Dimethyl-1-phenylbutyl)aniline (6j)

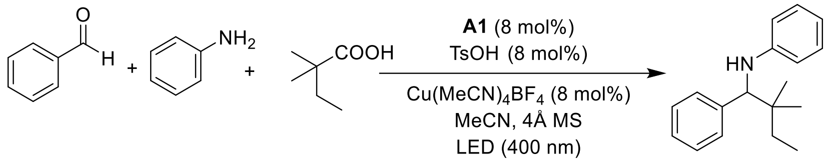

6j

According to GP1, the reaction was carried out with $Cu(MeCN)BF_4$ (5.0 mg, 0.016 mmol, 8 mol%), acridine **A1** (4.7 mg, 0.016 mmol, 8 mol %), 4Å molecular sieves (60 mg), aldehyde (21.2 mg, 0.2 mmol), aniline (22.3 mg, 0.24 mmol, 1.2 equiv.), carboxylic acid (30.2 mg, 0.26 mmol, 1.3 equiv.), anhydrous *p*-toluenesulfonic acid (2.8 mg, 0.016 mmol, 8 mol%), and acetonitrile (2 mL). The test-tube was capped and the reaction mixture was irradiated with LED light (λ = 400 nm) while stirring at at room temperature for 30 h. The reaction mixture was then concentrated under reduced pressure, and the remaining material was purified by flash chromatography on silica gel (EtOAc/hexane, 1 : 20 v/v) to give product **6j (**41.6 mg, 82%) as a colourless oil.

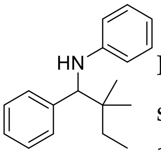

^{1}H NMR (500 MHz, $CDCl_3$): 7.37 – 7.26 (4 H, m), 7.26 – 7.19 (1 H, m), 7.10 – 7.03 (2 H, m), 6.61 (1 H, tt, *J* = 7.2, 1.1 Hz), 6.54 – 6.47 (2 H, m), 4.29 (1 H, s), 4.15 (1 H, s), 1.59 – 1.33 (2 H, m), 0.97 (3 H, s), 0.95 – 0.89 (6 H, m) ppm. – ^{13}C NMR (125 MHz, $CDCl_3$): 147.8, 141.2, 129.1, 128.8, 127.8, 126.9, 117.0, 113.2, 65.2, 37.6, 32.4, 24.0, 23.3, 8.4 ppm – IR: 2964, 1601, 1504, 1318 cm^{-1}. – HRMS: calcd for $C_{18}H_{23}N$: 254.1903, found 254.1904 [$M+H^+$].

N-((1-Methylcyclohexyl)(phenyl)methyl)aniline (6k)

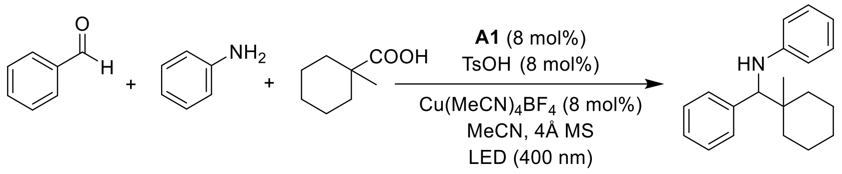

6k

According to GP1, the reaction was carried out with Cu(MeCN)BF$_4$ (5.0 mg, 0.016 mmol, 8 mol%), acridine **A1** (4.7 mg, 0.016 mmol, 8 mol %), 4Å molecular sieves (60 mg), aldehyde (21.2 mg, 0.2 mmol), aniline (22.3 mg, 0.24 mmol, 1.2 equiv.), carboxylic acid (36.9 mg, 0.26 mmol, 1.3 equiv.), anhydrous *p*-toluenesulfonic acid (2.8 mg, 0.016 mmol, 8 mol%), and acetonitrile (2 mL). The test-tube was capped and the reaction mixture was irradiated with LED light (λ = 400 nm) while stirring at at room temperature for 30 h. The reaction mixture was then concentrated under reduced pressure, and the remaining material was purified by flash chromatography on silica gel (EtOAc/hexane, 1 : 20 v/v) to give product **6k (**51.9 mg, 93%) as a colourless oil.

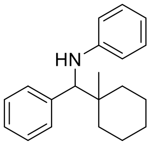

^{1}H NMR (500 MHz, CDCl$_3$): 7.36 – 7.24 (4 H, m), 7.25 – 7.18 (1 H, m), 7.09 – 7.02 (2 H, m), 6.59 (1 H, t, *J* = 7.3 Hz), 6.50 (2 H, d, *J* = 8.0 Hz), 4.31 (1 H, s), 4.15 (1 H, s), 1.73 – 1.51 (4 H, m), 1.53 – 1.37 (4 H, m), 1.33 – 1.15 (2 H, m), 0.96 (3 H, s) ppm. – ^{13}C NMR (125 MHz, CDCl$_3$): 148.0, 140.9, 129.2, 128.9, 127.8, 126.9, 116.9, 113.2, 66.9, 37.6, 35.7, 35.3, 26.4, 22.1, 21.9, 19.6 ppm – IR: 2923, 1738, 1399, 1501, 1318, 1217, 702 cm^{-1}. – HRMS: calcd for $C_{20}H_{25}N$: 280.2060, found 280.2054 [M+H$^+$].

N-((4-Methyltetrahydro-2*H*-pyran-4-yl)(phenyl)methyl)aniline (6l)

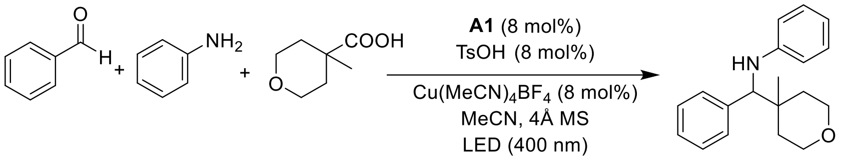

6l

According to GP1, the reaction was carried out with Cu(MeCN)BF$_4$ (5.0 mg, 0.016 mmol, 8 mol%), acridine **A1** (4.7 mg, 0.016 mmol, 8 mol %), 4Å molecular sieves (60 mg), aldehyde (21.2 mg, 0.2 mmol), aniline (22.3 mg, 0.24 mmol, 1.2 equiv.), carboxylic acid (37.4 mg, 0.26 mmol, 1.3 equiv.), anhydrous *p*-toluenesulfonic acid (2.8 mg, 0.016 mmol, 8 mol%), and acetonitrile (2 mL). The test-tube was capped and the reaction mixture was irradiated with LED light (λ = 400 nm) while stirring at at room temperature for 30 h. The reaction mixture was then concentrated under reduced pressure, and the remaining material was purified by flash chromatography on silica gel (EtOAc/hexane, 1 : 5 v/v) to give product **6l (**50.6 mg, 90%) as a white solid (m.p. 126 ^{o}C).

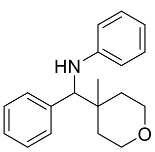

^{1}H NMR (500 MHz, CDCl$_3$): 7.35 – 7.26 (4 H, m), 7.23 (1 H, ddt, *J* = 8.4, 5.4, 2.3 Hz), 7.07 (2 H, dd, *J* = 8.6, 7.2 Hz), 6.68 – 6.57 (1 H, m), 6.55 – 6.49 (2 H, m), 4.28 (1 H, s), 4.15 (1 H, s), 3.85 (2 H, dddd, *J* = 12.2, 9.6, 4.8, 3.1 Hz), 3.59 (2 H, dtd, *J* = 26.1, 11.7, 2.5 Hz), 1.82 (2 H, dddd, J = 23.5, 13.5, 11.5, 4.8 Hz), 1.57 (1 H, dd, J = 13.6, 2.6 Hz), 1.08 (4 H, s) ppm. – ^{13}C NMR (125 MHz, CDCl$_3$): 147.6, 139.8, 129.2, 128.8, 128.0, 127.2, 117.4, 113.4, 67.5, 64.0, 63.7, 35.7, 35.5, 18.0 ppm – IR: 2970, 1738, 1365, 1229 cm^{-1}. – HRMS: calcd for $C_{19}H_{23}NO$: 282.1852, found 282.1846 [M+H$^+$].

N-((Hexahydro-2,5-methanopentalen-3a(1H)-yl)(phenyl)methyl)aniline (6m)

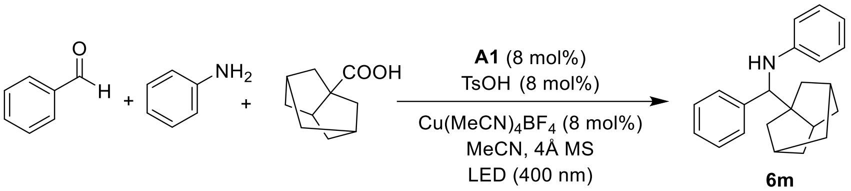

According to GP1, the reaction was carried out with Cu(MeCN)BF4 (5.0 mg, 0.016 mmol, 8 mol%), acridine **A1** (4.7 mg, 0.016 mmol, 8 mol %), 4Å molecular sieves (60 mg), aldehyde (21.2 mg, 0.2 mmol), aniline (22.3 mg, 0.24 mmol, 1.2 equiv.), carboxylic acid (43.2 mg, 0.26 mmol, 1.3 equiv.), anhydrous *p*-toluenesulfonic acid (2.8 mg, 0.016 mmol, 8 mol%), and acetonitrile (2 mL). The test-tube was capped and the reaction mixture was irradiated with LED light (λ = 400 nm) while stirring at at room temperature for 30 h. The reaction mixture was then concentrated under reduced pressure, and the remaining material was purified by flash chromatography on silica gel (EtOAc/hexane, 1 : 20 v/v) to give product **6m (**45.4 mg, 75%) as a colourless oil.

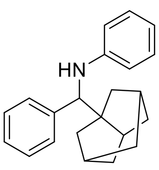

^{1}H NMR (500 MHz, $CDCl_3$): 7.36 (2 H, d, *J* = 7.0 Hz), 7.31 – 7.24 (2 H, m), 7.24 – 7.17 (1 H, m), 7.05 (2 H, dd, *J* = 8.6, 7.2 Hz), 6.60 (1 H, t, *J* = 7.3 Hz), 6.49 (2 H, d, *J* = 7.9 Hz), 4.31 (2 H, s), 2.44 (1 H, t, *J* = 6.7 Hz), 2.25 (1 H, s), 2.19 (1 H, s), 1.86 (1 H, d, *J* = 11.3 Hz), 1.80 (1 H, dt, *J* = 10.8, 2.1 Hz), 1.74 (1 H, dtd, *J* = 10.3, 3.7, 1.7 Hz), 1.68 – 1.44 (6 H, m), 1.37 (1 H, dd, *J* = 10.9, 3.0 Hz) ppm. – ^{13}C NMR (125 MHz, $CDCl_3$): 129.2, 128.1, 127.8, 126.9, 117.1, 113.5, 64.5, 54.1, 47.8, 45.2, 44.8, 44.1, 41.5, 37.3, 37.3, 35.6 ppm – IR: 2970, 1738, 1365, 1229, 1217 cm^{-1}. – HRMS: calcd for $C_{22}H_{25}N$: 304.2060, found 304.2054 [M+H$^+$].

N-(((1*s*,3*s*)-Adamantan-1-yl)(phenyl)methyl)aniline (6n)

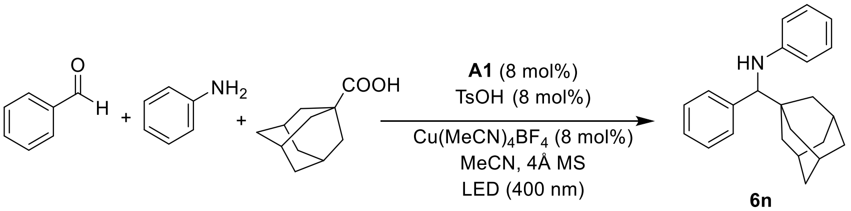

According to GP1, the reaction was carried out with Cu(MeCN)BF4 (5.0 mg, 0.016 mmol, 8 mol%), acridine **A1** (4.7 mg, 0.016 mmol, 8 mol %), 4Å molecular sieves (60 mg), aldehyde (21.2 mg, 0.2 mmol), aniline (22.3 mg, 0.24 mmol, 1.2 equiv.), carboxylic acid (46.8 mg, 0.26 mmol, 1.3 equiv.), anhydrous *p*-toluenesulfonic acid (2.8 mg, 0.016 mmol, 8 mol%), and acetonitrile (2 mL). The test-tube was capped and the reaction mixture was irradiated with LED light (λ = 400 nm) while stirring at at room temperature for 30 h. The reaction mixture was then concentrated under reduced pressure, and the remaining material was purified by flash chromatography on silica gel (EtOAc/hexane, 1 : 20 v/v) to give product **6n (**39.9 mg, 63%) as a colourless oil.

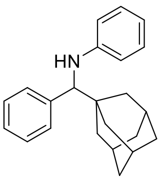

^{1}H NMR (500 MHz, $CDCl_3$): δ 7.36 – 7.14 (5 H, m), 7.03 (2 H, dd, *J* = 8.6, 7.2 Hz), 6.57 (1 H, t, *J* = 7.3 Hz), 6.49 (2 H, d, *J* = 8.0 Hz), 4.37 (1 H, s), 3.87 (1 H, s), 2.06 – 1.93 (3 H, m), 1.70 (3 H, ddd, *J* = 16.3, 12.4, 6.2 Hz), 1.63 – 1.46 (6 H, m). ppm. – ^{13}C NMR (125 MHz, $CDCl_3$): 140.4, 129.1, 128.8, 127.7, 126.9, 116.9, 113.3, 68.1, 39.4, 37.1, 36.6, 28.6 ppm – IR: 2902, 1738, 1502, 1365, 1217, 702 cm^{-1}. – HRMS: calcd for $C_{23}H_{27}N$: 318.2216, found 318.2218 [M+H$^+$].

Ethyl 2-((4-methoxyphenyl)amino)hexanoate (7a)

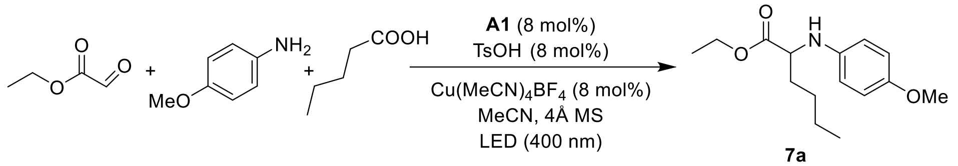

7a

According to GP1, the reaction was carried out with $Cu(MeCN)BF_4$ (5.0 mg, 0.016 mmol, 8 mol%), acridine **A1** (4.7 mg, 0.016 mmol, 8 mol %), 4Å molecular sieves (60 mg), aldehyde (14.8 mg, 0.2 mmol), aniline (29.5 mg, 0.24 mmol, 1.2 equiv.), carboxylic acid (26.5 mg, 0.26 mmol, 1.3 equiv.), anhydrous *p*-toluenesulfonic acid (2.8 mg, 0.016 mmol, 8 mol%), and acetonitrile (2 mL). The test-tube was capped and the reaction mixture was irradiated with LED light (λ = 400 nm) while stirring at at room temperature for 30 h. The reaction mixture was then concentrated under reduced pressure, and the remaining material was purified by flash chromatography on silica gel (EtOAc/hexane, 1 : 20 v/v) to give product **7a (**49.8 mg, 94%) as a colourless oil.

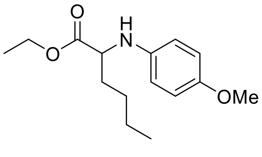

^{1}H NMR (500 MHz, $CDCl_3$): 6.76 (2 H, d, *J* = 8.9 Hz), 6.60 (2 H, d, *J* = 8.9 Hz), 4.25 – 4.11 (2 H, m), 3.95 (1 H, t, *J* = 6.6 Hz), 3.84 (1 H, s), 3.73 (3 H, s), 1.95 – 1.62 (2 H, m), 1.51 – 1.30 (4 H, m), 1.23 (3 H, t, *J* = 7.1 Hz), 0.91 (3 H, t, *J* = 7.1 Hz) ppm. – ^{13}C NMR (125 MHz, $CDCl_3$): 174.7, 152.8, 141.3, 115.2, 115.0, 60.9, 58.0, 55.8, 33.1, 27.9, 22.6, 14.4, 14.0 ppm – IR: 2956, 1732, 1514, 1237, 1035 cm^{-1}. – HRMS: calcd for $C_{15}H_{23}NO_3$: 266.1751, found 266.1751 $[M+H^+]$.

Ethyl 2-((4-methoxyphenyl)amino)-5-methylhexanoate (7b)

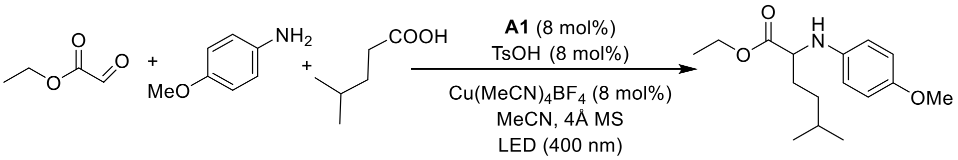

7b

According to GP1, the reaction was carried out with $Cu(MeCN)BF_4$ (5.0 mg, 0.016 mmol, 8 mol%), acridine **A1** (4.7 mg, 0.016 mmol, 8 mol %), 4Å molecular sieves (60 mg), aldehyde (14.8 mg, 0.2 mmol), aniline (29.5 mg, 0.24 mmol, 1.2 equiv.), carboxylic acid (30.2 mg, 0.26 mmol, 1.3 equiv.), anhydrous *p*-toluenesulfonic acid (2.8 mg, 0.016 mmol, 8 mol%), and acetonitrile (2 mL). The test-tube was capped and the reaction mixture was irradiated with LED light (λ = 400 nm) while stirring at at room temperature for 30 h. The reaction mixture was then concentrated under reduced pressure, and the remaining material was purified by flash chromatography on silica gel (EtOAc/hexane, 1 : 20 v/v) to give product **7b (**54.7 mg, 98%) as a colourless oil.

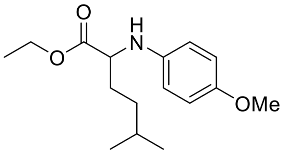

^{1}H NMR (500 MHz, $CDCl_3$): 6.76 (2 H, d, *J* = 8.9 Hz), 6.60 (2 H, d, *J* = 8.9 Hz), 4.16 (2 H, qd, *J* = 7.2, 1.8 Hz), 3.93 (1 H, t, *J* = 6.5 Hz), 3.84 (1 H, s), 3.73 (3 H, s), 1.93 – 1.66 (2 H, m), 1.56 (1 H, dq, *J* = 13.3, 6.7 Hz), 1.40 – 1.27 (2 H, m), 1.23 (3 H, t, *J* = 7.1 Hz), 0.89 (6 H, dd, *J* = 6.7, 3.2 Hz) ppm. – ^{13}C NMR (125 MHz, $CDCl_3$): 174.7, 152.8, 141.3, 115.2, 115.0, 61.0, 58.2, 55.8, 34.8, 31.3, 28.0, 22.6, 22.5, 14.4 ppm – IR: 2969, 1738, 1514, 1366, 1217, 1036 cm^{-1}. – HRMS: calcd for $C_{16}H_{25}NO_3$: 280.1907, found 280.1907 $[M+H^+]$.

Ethyl 2-((4-methoxyphenyl)amino)undecanoate (7c)

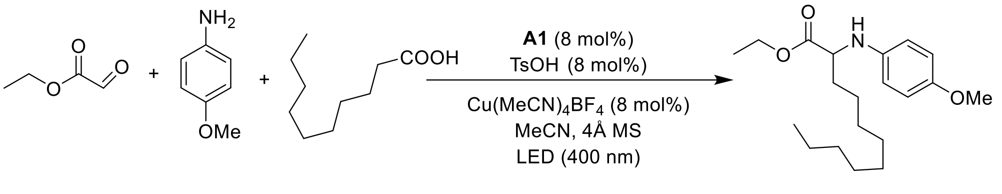

7c

According to GP1, the reaction was carried out with Cu(MeCN)BF$_4$ (5.0 mg, 0.016 mmol, 8 mol%), acridine **A1** (4.7 mg, 0.016 mmol, 8 mol %), 4Å molecular sieves (60 mg), aldehyde (14.8 mg, 0.2 mmol), aniline (29.5 mg, 0.24 mmol, 1.2 equiv.), carboxylic acid (44.7 mg, 0.26 mmol, 1.3 equiv.), anhydrous *p*-toluenesulfonic acid (2.8 mg, 0.016 mmol, 8 mol%), and acetonitrile (2 mL). The test-tube was capped and the reaction mixture was irradiated with LED light (λ = 400 nm) while stirring at at room temperature for 30 h. The reaction mixture was then concentrated under reduced pressure, and the remaining material was purified by flash chromatography on silica gel (EtOAc/hexane, 1 : 20 v/v) to give product **7c (**63.6 mg, 95%) as a colourless oil.

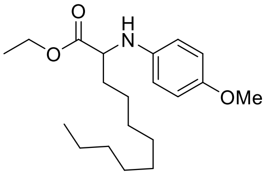

^{1}H NMR (500 MHz, CDCl$_3$): 6.81 – 6.72 (2 H, m), 6.66 – 6.56 (2 H, m), 4.16 (2 H, qd, J = 7.2, 0.9 Hz), 3.95 (1 H, t, J = 6.5 Hz), 3.84 (1 H, s), 3.73 (3 H, s), 1.75 (2 H, dddt, J = 26.7, 13.3, 9.6, 6.8 Hz), 1.42 (2 H, dddd, J = 12.9, 9.7, 6.6, 4.5 Hz), 1.38 – 1.18 (15 H, m), 0.88 (3 H, t, J = 6.9 Hz) ppm. – ^{13}C NMR (125 MHz, CDCl$_3$): 174.7, 152.8, 141.3, 115.2, 115.0, 60.9, 58.0, 55.8, 33.4, 32.0, 29.6, 29.5, 29.5, 29.4, 25.7, 22.8, 14.4, 14.2 ppm – IR: 2925, 1738, 1514, 1366, 1217 cm^{-1}. – HRMS: calcd for $C_{20}H_{33}NO_3$: 336.2533, found 336.2523 [M+H$^+$].

Ethyl 5,5,5-trifluoro-2-((4-methoxyphenyl)amino)pentanoate (7d)

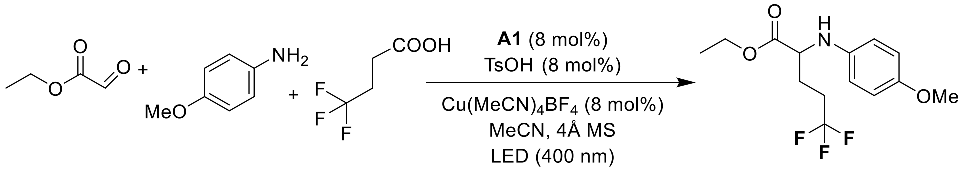

7d

According to GP1, the reaction was carried out with Cu(MeCN)BF$_4$ (5.0 mg, 0.016 mmol, 8 mol%), acridine **A1** (4.7 mg, 0.016 mmol, 8 mol %), 4Å molecular sieves (60 mg), aldehyde (14.8 mg, 0.2 mmol), aniline (29.5 mg, 0.24 mmol, 1.2 equiv.), carboxylic acid (36.9 mg, 0.26 mmol, 1.3 equiv.), anhydrous *p*-toluenesulfonic acid (2.8 mg, 0.016 mmol, 8 mol%), and acetonitrile (2 mL). The test-tube was capped and the reaction mixture was irradiated with LED light (λ = 400 nm) while stirring at at room temperature for 30 h. The reaction mixture was then concentrated under reduced pressure, and the remaining material was purified by flash chromatography on silica gel (EtOAc/hexane, 1 : 10 v/v) to give product **7d (**59.8 mg, 98%) as a colourless oil.

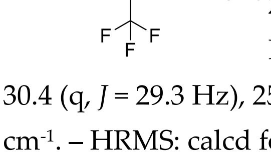

^{1}H NMR (500 MHz, CDCl$_3$): 6.78 (2 H, d, J = 8.9 Hz), 6.62 (2 H, d, J = 8.9 Hz), 4.19 (2 H, qd, J = 7.2, 2.1 Hz), 4.00 (1 H, dd, J = 7.8, 5.4 Hz), 3.74 (3 H, s), 2.43 – 2.19 (2 H, m), 2.17 – 2.05 (1 H, m), 1.94 (1 H, dddd, J = 13.5, 10.3, 7.7, 5.8 Hz), 1.25 (3 H, t, J = 7.1 Hz) ppm. – ^{13}C NMR (125 MHz, CDCl$_3$): 173.5, 153.3, 140.6, 115.7, 115.1, 79.8 – 74.0 (m), 61.6, 57.0, 55.8, 30.4 (q, J = 29.3 Hz), 25.8, 14.3 ppm. – ^{19}F NMR (376 MHz, CDCl$_3$) δ -66.3 ppm. – IR: 1732, 1515, 1241, 1136, 1031 cm^{-1}. – HRMS: calcd for $C_{14}H_{18}F_3NO_3$: 306.1312, found 306.1312 [M+H$^+$].

Ethyl 2-((4-methoxyphenyl)amino)-4,4,4-triphenylbutanoate (7e)

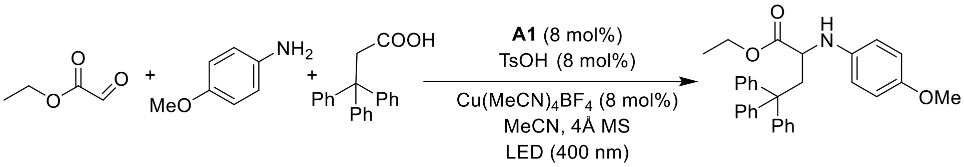

7e

According to GP1, the reaction was carried out with Cu(MeCN)BF4 (5.0 mg, 0.016 mmol, 8 mol%), acridine **A1** (4.7 mg, 0.016 mmol, 8 mol %), 4Å molecular sieves (60 mg), aldehyde (14.8 mg, 0.2 mmol), aniline (29.5 mg, 0.24 mmol, 1.2 equiv.), carboxylic acid (78.5 mg, 0.26 mmol, 1.3 equiv.), anhydrous *p*-toluenesulfonic acid (2.8 mg, 0.016 mmol, 8 mol%), and acetonitrile (2 mL). The test-tube was capped and the reaction mixture was irradiated with LED light (λ = 400 nm) while stirring at at room temperature for 30 h. The reaction mixture was then concentrated under reduced pressure, and the remaining material was purified by flash chromatography on silica gel (EtOAc/hexane, 1 : 20 v/v) to give product **7e (**55.8 mg, 60%) as a colourless oil.

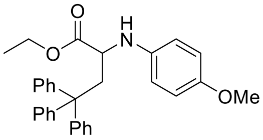

^{1}H NMR (500 MHz, $CDCl_3$): 7.37 – 7.28 (5 H, m), 7.22 (3 H, dd, *J* = 5.1, 2.0 Hz), 7.16 – 7.09 (3 H, m), 7.06 (2 H, dd, *J* = 8.2, 6.7 Hz), 6.77 – 6.70 (4 H, m), 6.53 (2 H, d, *J* = 8.9 Hz), 4.80 (1 H, s), 3.94 – 3.74 (3 H, m), 3.73 (4 H, s), 3.61 (1 H, d, *J* = 12.8 Hz), 0.88 (3 H, t, *J* = 7.1 Hz) ppm. – ^{13}C NMR (125 MHz, $CDCl_3$): 172.6, 152.9, 143.8, 143.5, 140.7, 137.4, 131.3, 130.1, 129.8, 127.7, 127.6, 127.4, 127.0, 126.7, 126.4, 115.5, 114.9, 60.8, 60.7, 55.8, 44.8, 13.9 ppm – IR: 1721, 1512, 1446, 1245, 1035, 704 cm^{-1}. – HRMS: calcd for $C_{31}H_{31}NO_3$: 466.2377, found 466.2377 [M+H$^+$].

Ethyl 3-(4-chlorophenyl)-2-((4-methoxyphenyl)amino)propanoate (7f)

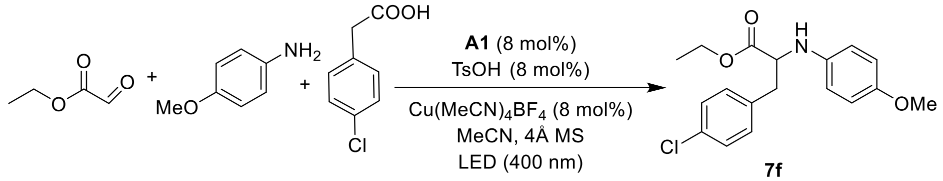

7f

According to GP1, the reaction was carried out with Cu(MeCN)BF4 (5.0 mg, 0.016 mmol, 8 mol%), acridine **A1** (4.7 mg, 0.016 mmol, 8 mol %), 4Å molecular sieves (60 mg), aldehyde (14.8 mg, 0.2 mmol), aniline (29.5 mg, 0.24 mmol, 1.2 equiv.), carboxylic acid (44.2 mg, 0.26 mmol, 1.3 equiv.), anhydrous *p*-toluenesulfonic acid (2.8 mg, 0.016 mmol, 8 mol%), and acetonitrile (2 mL). The test-tube was capped and the reaction mixture was irradiated with LED light (λ = 400 nm) while stirring at at room temperature for 30 h. The reaction mixture was then concentrated under reduced pressure, and the remaining material was purified by flash chromatography on silica gel (EtOAc/hexane, 1 : 15 v/v) to give product **7f (**64.6 mg, 97%) as a colourless oil.

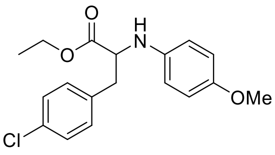

^{1}H NMR (500 MHz, $CDCl_3$): 7.26 (2 H, d, *J* = 8.4 Hz), 7.12 (2 H, d, *J* = 8.4 Hz), 6.77 (2 H, d, *J* = 8.9 Hz), 6.58 (2 H, d, *J* = 8.9 Hz), 4.24 (1 H, t, *J* = 6.3 Hz), 4.11 (2 H, qd, *J* = 7.1, 2.4 Hz), 3.74 (3 H, s), 3.24 – 2.95 (2 H, m), 1.17 (3 H, t, *J* = 7.1 Hz) ppm. – ^{13}C NMR (125 MHz, $CDCl_3$): 173.3, 153.0, 140.4, 135.2, 132.9, 130.8, 128.7, 115.5, 115.0, 61.3, 59.0, 55.8, 38.2, 14.3 ppm – IR: 1732, 1514, 1239, 1091 cm^{-1}. – HRMS: calcd for $C_{18}H_{20}ClNO_3$: 334.1204, found 334.1204 [M+H$^+$].

Go to table of contents

Ethyl 4-(4-fluorophenyl)-2-((4-methoxyphenyl)amino)butanoate (7g)

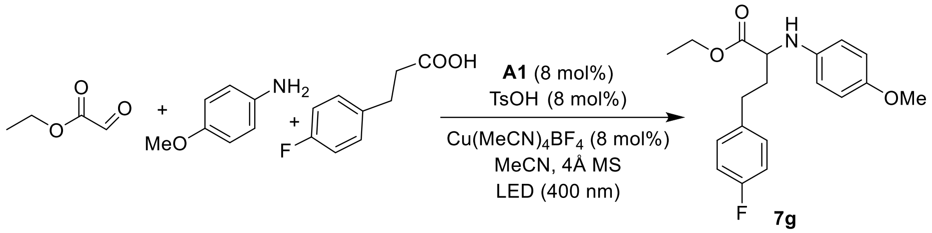

According to GP1, the reaction was carried out with Cu(MeCN)BF4 (5.0 mg, 0.016 mmol, 8 mol%), acridine **A1** (4.7 mg, 0.016 mmol, 8 mol %), 4Å molecular sieves (60 mg), aldehyde (14.8 mg, 0.2 mmol), aniline (29.5 mg, 0.24 mmol, 1.2 equiv.), carboxylic acid (43.7 mg, 0.26 mmol, 1.3 equiv.), anhydrous *p*-toluenesulfonic acid (2.8 mg, 0.016 mmol, 8 mol%), and acetonitrile (2 mL). The test-tube was capped and the reaction mixture was irradiated with LED light (λ = 400 nm) while stirring at at room temperature for 30 h. The reaction mixture was then concentrated under reduced pressure, and the remaining material was purified by flash chromatography on silica gel (EtOAc/hexane, 1 : 20 v/v) to give product **7g (**64.9 mg, 98%) as a colourless oil.

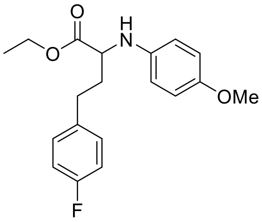

^{1}H NMR (500 MHz, CDCl$_3$): 7.15 (2 H, dd, *J* = 8.5, 5.5 Hz), 6.98 (2 H, t, *J* = 8.7 Hz), 6.76 (2 H, d, *J* = 9.0 Hz), 6.58 (2 H, d, *J* = 8.9 Hz), 4.16 (2 H, q, *J* = 7.1 Hz), 3.95 (1 H, dd, *J* = 7.4, 5.7 Hz), 3.74 (3 H, s), 2.76 (2 H, t, *J* = 7.8 Hz), 2.12 (1 H, dtd, *J* = 13.7, 8.0, 5.7 Hz), 2.00 (1 H, dq, *J* = 13.7, 7.5 Hz), 1.24 (3 H, t, *J* = 7.1 Hz) ppm. – ^{13}C NMR (125 MHz, CDCl$_3$): 174.4, 161.6 (d, *J* = 244.2 Hz), 153.0, 141.0, 136.7 (d, *J* = 3.4 Hz), 130.0 (d, *J* = 7.9 Hz), 115.4, 115.3, 115.0, 61.2, 57.3, 55.8, 35.0, 31.2, 14.4 ppm. – ^{19}F NMR (376 MHz, CDCl$_3$) δ -117.2 (tt, *J* = 9.3, 5.4 Hz) ppm. – IR: 1731, 1511, 1239, 1037 cm^{-1}. – HRMS: calcd for $C_{19}H_{22}FNO_3$: 332.1656, found 332.1656 [M+H$^+$].

Ethyl 2-((4-methoxyphenyl)amino)-5-phenylpentanoate (7h)

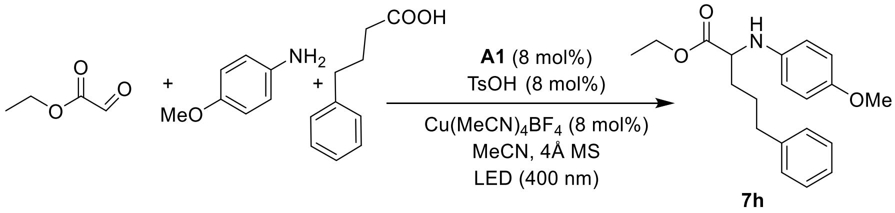

According to GP1, the reaction was carried out with Cu(MeCN)BF4 (5.0 mg, 0.016 mmol, 8 mol%), acridine **A1** (4.7 mg, 0.016 mmol, 8 mol %), 4Å molecular sieves (60 mg), aldehyde (14.8 mg, 0.2 mmol), aniline (29.5 mg, 0.24 mmol, 1.2 equiv.), carboxylic acid (42.6 mg, 0.26 mmol, 1.3 equiv.), anhydrous *p*-toluenesulfonic acid (2.8 mg, 0.016 mmol, 8 mol%), and acetonitrile (2 mL). The test-tube was capped and the reaction mixture was irradiated with LED light (λ = 400 nm) while stirring at at room temperature for 30 h. The reaction mixture was then concentrated under reduced pressure, and the remaining material was purified by flash chromatography on silica gel (EtOAc/hexane, 1 : 20 v/v) to give product **7h (**59.5 mg, 91%) as a colourless oil.

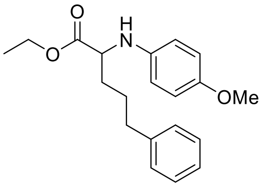

^{1}H NMR (500 MHz, CDCl$_3$): 7.31 – 7.25 (2 H, m), 7.22 – 7.13 (3 H, m), 6.76 (2 H, d, *J* = 8.9 Hz), 6.59 (2 H, d, *J* = 8.9 Hz), 4.15 (2 H, q, *J* = 7.1 Hz), 4.05 – 3.90 (1 H, m), 3.74 (3 H, s), 2.66 (2 H, td, *J* = 6.8, 2.8 Hz), 1.85 (1 H, ddd, *J* = 9.7, 5.4, 2.2 Hz), 1.82 – 1.70 (3 H, m), 1.22 (3 H, t, *J* = 7.1 Hz) ppm. – ^{13}C NMR (125 MHz, CDCl$_3$): 174.5, 152.9, 141.9, 141.1, 128.5, 128.5, 126.0, 115.3, 115.0, 61.1, 57.9, 55.8, 35.6, 32.8, 27.5, 14.4 ppm – IR: 2935, 1732, 1514, 1237, 1036, 821 cm^{-1}. – HRMS: calcd for $C_{20}H_{25}NO_3$: 328.1907, found 328.1906 [M+H$^+$].

Ethyl 2-((4-methoxyphenyl)amino)-6-phenylhexanoate (7i)

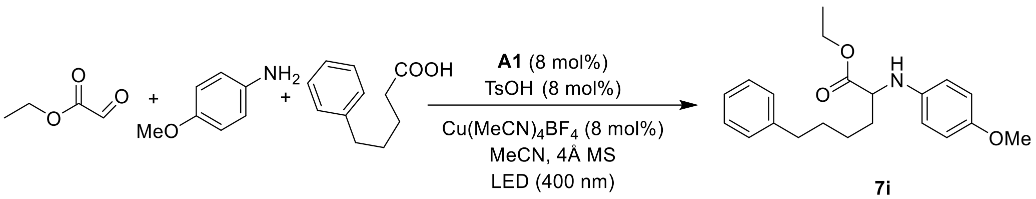

7i

According to GP1, the reaction was carried out with Cu(MeCN)BF_4 (5.0 mg, 0.016 mmol, 8 mol%), acridine **A1** (4.7 mg, 0.016 mmol, 8 mol %), 4Å molecular sieves (60 mg), aldehyde (14.8 mg, 0.2 mmol), aniline (29.5 mg, 0.24 mmol, 1.2 equiv.), carboxylic acid (46.3 mg, 0.26 mmol, 1.3 equiv.), anhydrous *p*-toluenesulfonic acid (2.8 mg, 0.016 mmol, 8 mol%), and acetonitrile (2 mL). The test-tube was capped and the reaction mixture was irradiated with LED light (λ = 400 nm) while stirring at at room temperature for 30 h. The reaction mixture was then concentrated under reduced pressure, and the remaining material was purified by flash chromatography on silica gel (EtOAc/hexane, 1 : 20 v/v) to give product **7i (**64.1 mg, 94%) as a colourless oil.

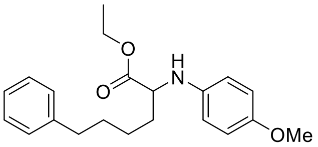

^{1}H NMR (500 MHz, $CDCl_3$): 7.34 – 7.24 (2 H, m), 7.22 – 7.14 (3 H, m), 6.77 (2 H, d, *J* = 8.9 Hz), 6.60 (2 H, d, *J* = 8.9 Hz), 4.15 (2 H, q, *J* = 7.1 Hz), 3.96 (1 H, t, *J* = 6.5 Hz), 3.84 (1 H, s), 3.74 (3 H, s), 2.74 – 2.53 (2 H, m), 1.81 (2 H, ddq, *J* = 31.2, 9.3, 6.8 Hz), 1.67 (2 H, q, *J* = 7.7 Hz), 1.57 – 1.39 (2 H, m), 1.22 (3 H, t, *J* = 7.1 Hz) ppm. – ^{13}C NMR (125 MHz, $CDCl_3$): 174.6, 152.8, 142.4, 141.2, 128.5, 128.4, 125.9, 115.2, 115.0, 61.0, 57.9, 55.8, 35.8, 33.2, 31.3, 25.4, 14.4 ppm – IR: 2936, 1732, 1514, 1238, 1036 cm^{-1}. – HRMS: calcd for $C_{21}H_{27}NO_3$: 342.2064, found 342.2063 [M+H$^+$].

Ethyl 2-cyclopentyl-2-((4-methoxyphenyl)amino)acetate (7j)

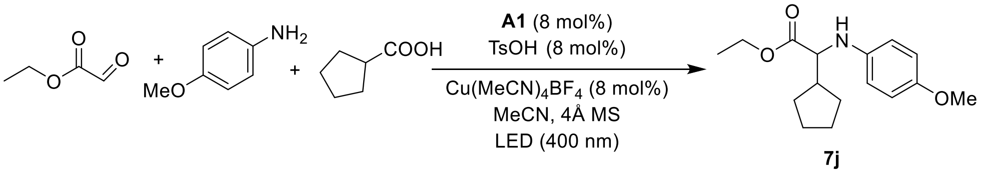

7j

According to GP1, the reaction was carried out with Cu(MeCN)BF_4 (5.0 mg, 0.016 mmol, 8 mol%), acridine **A1** (4.7 mg, 0.016 mmol, 8 mol %), 4Å molecular sieves (60 mg), aldehyde (14.8 mg, 0.2 mmol), aniline (29.5 mg, 0.24 mmol, 1.2 equiv.), carboxylic acid (29.6 mg, 0.26 mmol, 1.3 equiv.), anhydrous *p*-toluenesulfonic acid (2.8 mg, 0.016 mmol, 8 mol%), and acetonitrile (2 mL). The test-tube was capped and the reaction mixture was irradiated with LED light (λ = 400 nm) while stirring at at room temperature for 30 h. The reaction mixture was then concentrated under reduced pressure, and the remaining material was purified by flash chromatography on silica gel (EtOAc/hexane, 1 : 20 v/v) to give product **7j (**54.8 mg, 99%) as a colourless oil.

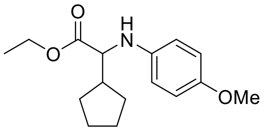

^{1}H NMR (500 MHz, $CDCl_3$): 6.75 (2 H, d, *J* = 8.9 Hz), 6.61 (2 H, d, *J* = 8.9 Hz), 4.20 – 4.04 (2 H, m), 3.85 (1 H, s), 3.77 (1 H, d, *J* = 7.9 Hz), 3.73 (3 H, s), 2.20 (1 H, p, *J* = 8.1 Hz), 1.83 (1 H, dtd, *J* = 14.5, 7.6, 4.4 Hz), 1.77 – 1.52 (5 H, m), 1.46 (2 H, dddd, *J* = 15.5, 12.6, 8.8, 7.0 Hz), 1.22 (3 H, t, *J* = 7.1 Hz) ppm. – ^{13}C NMR (125 MHz, $CDCl_3$): 174.6, 152.8, 141.6, 115.2, 114.9, 62.3, 60.8, 55.8, 43.3, 29.6, 29.2, 25.5, 25.3, 14.4 ppm – IR: 2969, 1737, 1514, 1366, 1230, 1035 cm^{-1}. – HRMS: calcd for $C_{16}H_{23}NO_3$: 278.1751, found 278.1750 [M+H$^+$].

Ethyl 2-cyclohexyl-2-((4-methoxyphenyl)amino)acetate (7k)

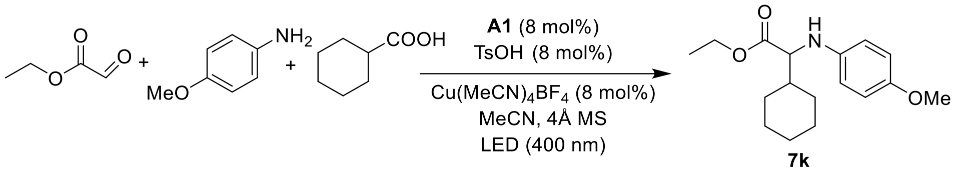

7k

According to GP1, the reaction was carried out with Cu(MeCN)BF_4 (5.0 mg, 0.016 mmol, 8 mol%), acridine **A1** (4.7 mg, 0.016 mmol, 8 mol %), 4Å molecular sieves (60 mg), aldehyde (14.8 mg, 0.2 mmol), aniline (29.5 mg, 0.24 mmol, 1.2 equiv.), carboxylic acid (33.3 mg, 0.26 mmol, 1.3 equiv.), anhydrous *p*-toluenesulfonic acid (2.8 mg, 0.016 mmol, 8 mol%), and acetonitrile (2 mL). The test-tube was capped and the reaction mixture was irradiated with LED light (λ = 400 nm) while stirring at at room temperature for 30 h. The reaction mixture was then concentrated under reduced pressure, and the remaining material was purified by flash chromatography on silica gel (EtOAc/hexane, 1 : 20 v/v) to give product **7k (**57.0 mg, 98%) as a colourless oil.

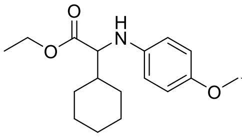

^{1}H NMR (500 MHz, $CDCl_3$): 6.75 (2 H, d, *J* = 8.9 Hz), 6.60 (2 H, d, *J* = 9.0 Hz), 4.15 (2 H, qd, *J* = 7.1, 1.2 Hz), 3.86 (1 H, s), 3.76 (1 H, d, *J* = 6.1 Hz), 3.73 (3 H, s), 1.93 – 1.83 (1 H, m), 1.82 – 1.57 (5 H, m), 1.42 – 1.03 (8 H, m) ppm. – ^{13}C NMR (125 MHz, $CDCl_3$): 174.1, 152.7, 141.8, 115.3, 115.0, 63.5, 60.8, 55.8, 41.4, 29.8, 29.3, 26.3, 26.2, 26.2, 14.4 ppm – IR: 2925, 2851, 1729, 1512, 1239, 1036, 821 cm^{-1}. – HRMS: calcd for $C_{17}H_{25}NO_3$: 292.1907, found 292.1904 [M+H$^+$].

Ethyl 2-cycloheptyl-2-((4-methoxyphenyl)amino)acetate (7l)

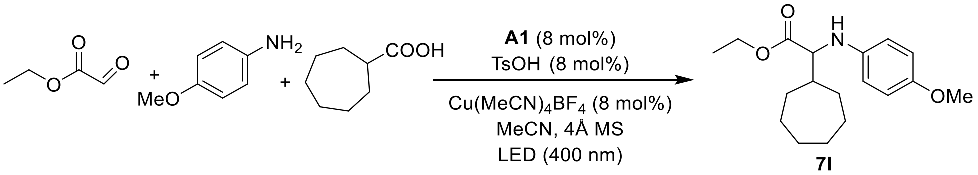

7l

According to GP1, the reaction was carried out with Cu(MeCN)BF_4 (5.0 mg, 0.016 mmol, 8 mol%), acridine **A1** (4.7 mg, 0.016 mmol, 8 mol %), 4Å molecular sieves (60 mg), aldehyde (14.8 mg, 0.2 mmol), aniline (29.5 mg, 0.24 mmol, 1.2 equiv.), carboxylic acid (36.9 mg, 0.26 mmol, 1.3 equiv.), anhydrous *p*-toluenesulfonic acid (2.8 mg, 0.016 mmol, 8 mol%), and acetonitrile (2 mL). The test-tube was capped and the reaction mixture was irradiated with LED light (λ = 400 nm) while stirring at at room temperature for 30 h. The reaction mixture was then concentrated under reduced pressure, and the remaining material was purified by flash chromatography on silica gel (EtOAc/hexane, 1 : 20 v/v) to give product **7l (**59.2 mg, 97%) as a colourless oil.

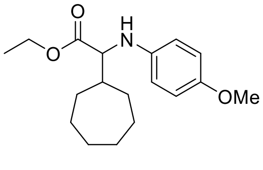

^{1}H NMR (500 MHz, $CDCl_3$): 6.82 – 6.70 (2 H, m), 6.66 – 6.56 (2 H, m), 4.15 (2 H, qd, *J* = 7.1, 4.2 Hz), 3.88 (1 H, s), 3.79 (1 H, d, *J* = 5.8 Hz), 3.73 (3 H, s), 2.00 – 1.88 (1 H, m), 1.89 – 1.80 (1 H, m), 1.79 – 1.63 (3 H, m), 1.64 – 1.36 (8 H, m), 1.23 (3 H, t, *J* = 7.1 Hz) ppm. – ^{13}C NMR (125 MHz, $CDCl_3$): 174.2, 152.7, 141.7, 115.3, 115.0, 63.8, 60.8, 55.8, 42.8, 31.3, 30.1, 28.6, 28.0, 26.8, 26.7, 14.4 ppm – IR: 2926, 1732, 1514, 1238, 1036 cm^{-1}. – HRMS: calcd for $C_{18}H_{27}NO_3$: 306.2064, found 306.2064 [M+H$^+$].

Ethyl 2-cyclobutyl-2-((4-methoxyphenyl)amino)acetate (7m)

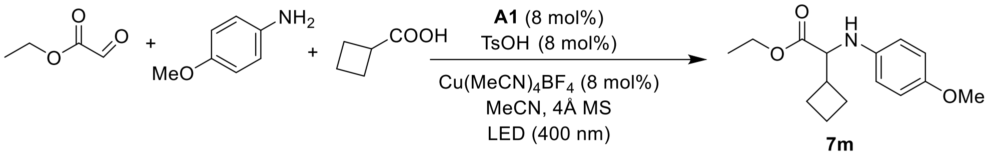

According to GP1, the reaction was carried out with $Cu(MeCN)BF_4$ (5.0 mg, 0.016 mmol, 8 mol%), acridine **A1** (4.7 mg, 0.016 mmol, 8 mol %), 4Å molecular sieves (60 mg), aldehyde (14.8 mg, 0.2 mmol), aniline (29.5 mg, 0.24 mmol, 1.2 equiv.), carboxylic acid (26.0 mg, 0.26 mmol, 1.3 equiv.), anhydrous *p*-toluenesulfonic acid (2.8 mg, 0.016 mmol, 8 mol%), and acetonitrile (2 mL). The test-tube was capped and the reaction mixture was irradiated with LED light (λ = 400 nm) while stirring at at room temperature for 30 h. The reaction mixture was then concentrated under reduced pressure, and the remaining material was purified by flash chromatography on silica gel (EtOAc/hexane, 1 : 20 v/v) to give product **7m (**40.0 mg, 76%) as a colourless oil.

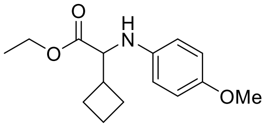

^1H NMR (500 MHz, $CDCl_3$): 6.76 (2 H, d, *J* = 8.9 Hz), 6.60 (2 H, d, *J* = 8.9 Hz), 4.14 (2 H, p, *J* = 7.1 Hz), 3.88 (1 H, d, *J* = 8.1 Hz), 3.78 (1 H, s), 3.73 (3 H, s), 2.65 (1 H, q, *J* = 8.2 Hz), 2.22 – 1.73 (6 H, m), 1.22 (3 H, t, *J* = 7.1 Hz) ppm. – ^{13}C NMR (125 MHz, $CDCl_3$): 173.8, 152.8, 141.6, 115.2, 115.0, 62.5, 60.9, 55.8, 38.5, 25.7, 24.9, 18.2, 14.5 ppm – IR: 2970, 1738, 1514, 1230 cm^{-1}. – HRMS: calcd for $C_{15}H_{21}NO_3$: 264.1594, found 264.1589 $[M+H^+]$.

Ethyl 2-((4-methoxyphenyl)amino)-2-(tetrahydro-2*H*-pyran-4-yl)acetate (7n)

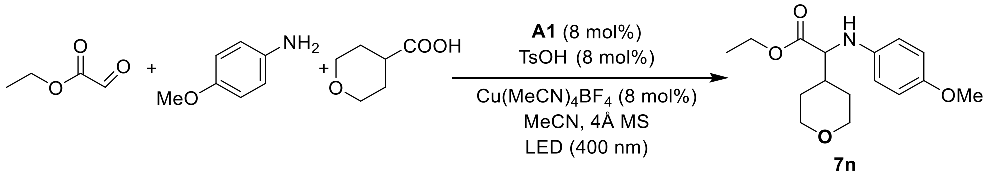

According to GP1, the reaction was carried out with $Cu(MeCN)BF_4$ (5.0 mg, 0.016 mmol, 8 mol%), acridine **A1** (4.7 mg, 0.016 mmol, 8 mol %), 4Å molecular sieves (60 mg), aldehyde (14.8 mg, 0.2 mmol), aniline (29.5 mg, 0.24 mmol, 1.2 equiv.), carboxylic acid (33.8 mg, 0.26 mmol, 1.3 equiv.), anhydrous *p*-toluenesulfonic acid (2.8 mg, 0.016 mmol, 8 mol%), and acetonitrile (2 mL). The test-tube was capped and the reaction mixture was irradiated with LED light (λ = 400 nm) while stirring at at room temperature for 30 h. The reaction mixture was then concentrated under reduced pressure, and the remaining material was purified by flash chromatography on silica gel (EtOAc/hexane, 1 : 20 v/v) to give product **7n (**53.3 mg, 91%) as a colourless oil.

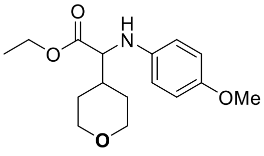

^1H NMR (500 MHz, $CDCl_3$): 6.79 – 6.73 (2 H, m), 6.61 (2 H, dd, *J* = 9.0, 0.9 Hz), 4.16 (2 H, qd, *J* = 7.2, 1.0 Hz), 4.07 – 3.95 (2 H, m), 3.79 (1 H, d, *J* = 6.7 Hz), 3.73 (3 H, d, *J* = 1.1 Hz), 3.46 – 3.32 (2 H, m), 1.95 (1 H, dtd, *J* = 11.5, 7.2, 3.3 Hz), 1.84 – 1.72 (1 H, m), 1.69 – 1.45 (3 H, m), 1.23 (3 H, td, *J* = 7.1, 1.0 Hz) ppm. – ^{13}C NMR (125 MHz, $CDCl_3$): 173.6, 153.0, 141.3, 115.5, 115.0, 68.0, 67.7, 63.0, 61.1, 55.8, 38.8, 29.6, 29.5, 14.4 ppm – IR: 2948, 1732, 1514, 1239, 1033 cm^{-1}. – HRMS: calcd for $C_{16}H_{23}NO_4$: 294.1700, found 294.1692 $[M+H^+]$.

Ethyl (4-methoxyphenyl)valinate (7o)

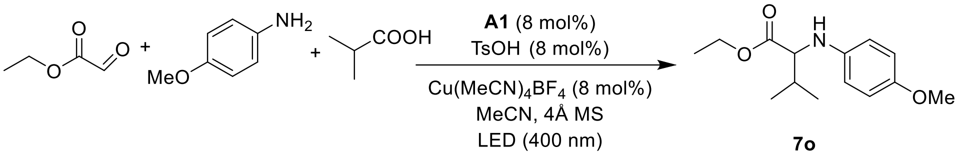

According to GP1, the reaction was carried out with Cu(MeCN)BF4 (5.0 mg, 0.016 mmol, 8 mol%), acridine **A1** (4.7 mg, 0.016 mmol, 8 mol %), 4Å molecular sieves (60 mg), aldehyde (14.8 mg, 0.2 mmol), aniline (29.5 mg, 0.24 mmol, 1.2 equiv.), carboxylic acid (22.9 mg, 0.26 mmol, 1.3 equiv.), anhydrous *p*-toluenesulfonic acid (2.8 mg, 0.016 mmol, 8 mol%), and acetonitrile (2 mL). The test-tube was capped and the reaction mixture was irradiated with LED light (λ = 400 nm) while stirring at at room temperature for 30 h. The reaction mixture was then concentrated under reduced pressure, and the remaining material was purified by flash chromatography on silica gel (EtOAc/hexane, 1 : 20 v/v) to give product **7o (**42.2 mg, 84%) as a colourless oil.

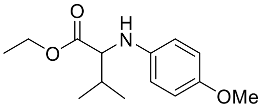

^{1}H NMR (500 MHz, $CDCl_3$): 6.76 (2 H, d, *J* = 8.9 Hz), 6.61 (2 H, d, *J* = 8.9 Hz), 4.16 (2 H, qt, *J* = 7.2, 3.9 Hz), 3.87 (1 H, s), 3.73 (3 H, s), 2.09 (1 H, dq, *J* = 13.4, 6.7 Hz), 1.23 (3 H, t, *J* = 7.1 Hz), 1.03 (6 H, dd, *J* = 10.2, 6.8 Hz) ppm. – ^{13}C NMR (125 MHz, $CDCl_3$): 174.1, 152.8, 141.7, 115.4, 115.0, 64.0, 60.9, 55.9, 31.7, 19.3, 18.8, 14.4 ppm – IR: 2963, 1732, 1514, 1234, 1035, 821 cm^{-1}. – HRMS: calcd for $C_{14}H_{21}NO_3$: 252.1594, found 252.1593 [M+H$^+$].

Ethyl 2-((4-methoxyphenyl)amino)-3-propylhexanoate (7p)

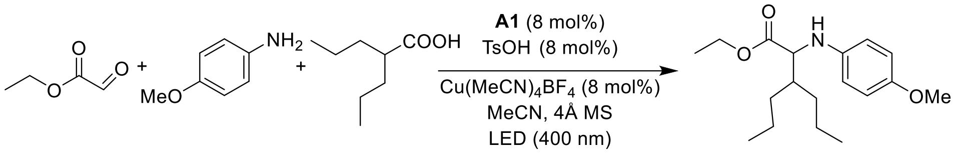

According to GP1, the reaction was carried out with Cu(MeCN)BF4 (5.0 mg, 0.016 mmol, 8 mol%), acridine **A1** (4.7 mg, 0.016 mmol, 8 mol %), 4Å molecular sieves (60 mg), aldehyde (14.8 mg, 0.2 mmol), aniline (29.5 mg, 0.24 mmol, 1.2 equiv.), carboxylic acid (37.4 mg, 0.26 mmol, 1.3 equiv.), anhydrous *p*-toluenesulfonic acid (2.8 mg, 0.016 mmol, 8 mol%), and acetonitrile (2 mL). The test-tube was capped and the reaction mixture was irradiated with LED light (λ = 400 nm) while stirring at at room temperature for 30 h. The reaction mixture was then concentrated under reduced pressure, and the remaining material was purified by flash chromatography on silica gel (EtOAc/hexane, 1 : 20 v/v) to give product **7p (**56.5 mg, 92%) as a colourless oil.

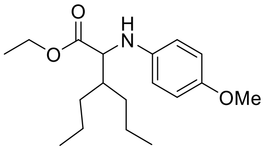

^{1}H NMR (500 MHz, $CDCl_3$): 6.76 (d, *J* = 8.9 Hz, 2 H), 6.60 (d, *J* = 9.0 Hz, 2 H), 4.15 (qq, *J* = 10.8, 7.1 Hz, 2 H), 3.97 (d, *J* = 5.0 Hz, 1 H), 3.82 (s, 1 H), 3.73 (s, 3 H), 1.84 (q, *J* = 4.9 Hz, 1 H), 1.55 – 1.25 (m, 8 H), 1.23 (t, *J* = 7.1 Hz, 3 H), 0.98 – 0.83 (m, 6 H) ppm. – ^{13}C NMR (125 MHz, $CDCl_3$): 174.4, 152.8, 141.8, 115.4, 115.0, 60.8, 60.5, 55.8, 40.9, 32.9, 32.3, 20.5, 20.4, 14.4, 14.4 ppm – IR: 2956, 1732, 1513, 1240, 1036, 820 cm^{-1}. – HRMS: calcd for $C_{18}H_{29}NO_3$: 308.2220, found 308.2220 [M+H$^+$].

Ethyl 3-hexyl-2-((4-methoxyphenyl)amino)decanoate (7q)

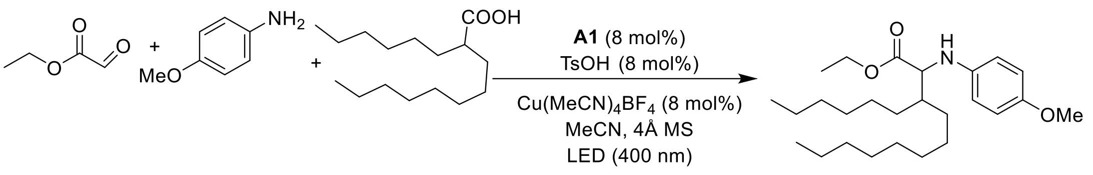

7q

According to GP1, the reaction was carried out with Cu(MeCN)BF4 (5.0 mg, 0.016 mmol, 8 mol%), acridine **A1** (4.7 mg, 0.016 mmol, 8 mol %), 4Å molecular sieves (60 mg), aldehyde (14.8 mg, 0.2 mmol), aniline (29.5 mg, 0.24 mmol, 1.2 equiv.), carboxylic acid (62.9 mg, 0.26 mmol, 1.3 equiv.), anhydrous *p*-toluenesulfonic acid (2.8 mg, 0.016 mmol, 8 mol%), and acetonitrile (2 mL). The test-tube was capped and the reaction mixture was irradiated with LED light (λ = 400 nm) while stirring at at room temperature for 30 h. The reaction mixture was then concentrated under reduced pressure, and the remaining material was purified by flash chromatography on silica gel (EtOAc/hexane, 1 : 20 v/v) to give product **7q (**80.4 mg, 1:1 dr, 99%) as a colourless oil.

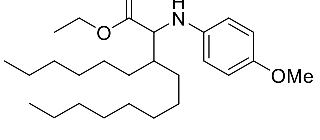

^{1}H NMR (500 MHz, $CDCl_3$): 6.76 (2 H, d, *J* = 8.9 Hz), 6.60 (2 H, d, *J* = 8.9 Hz), 4.30 – 4.04 (2 H, m), 3.97 (1 H, d, *J* = 4.9 Hz), 3.86 – 3.77 (1 H, m), 3.73 (3 H, s), 1.80 (1 H, q, *J* = 5.7 Hz), 1.44 (2 H ,td, *J* = 5.1, 4.7, 2.5 Hz), 1.40 – 1.16 (23 H, m), 0.88 (6 H, td, *J* = 6.9, 1.8 Hz) ppm. – ^{13}C NMR (125 MHz, $CDCl_3$): 174.4, 152.8, 141.8, 115.4, 115.0, 60.8, 60.5, 55.9, 41.4, 32.0, 31.9, 31.9, 30.6, 30.0, 30.0, 29.7, 29.6, 29.4, 27.3, 27.2, 22.8, 22.8, 14.4, 14.2, 14.2 ppm – IR: 2926, 1738, 1365, 1217 cm^{-1}. – HRMS: calcd for $C_{26}H_{45}NO_3$: 372.1594, found 372.1596 [M+H$^+$].

Ethyl 2-(2,3-dihydro-1*H*-inden-2-yl)-2-((4-methoxyphenyl)amino)acetate (7r)

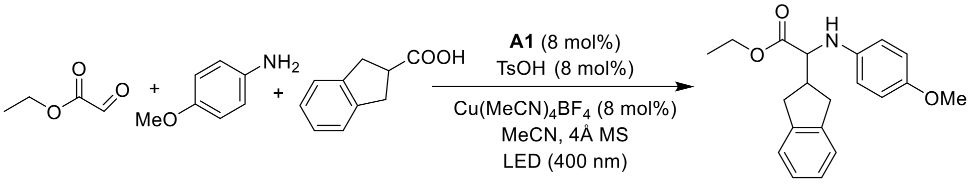

7r

According to GP1, the reaction was carried out with Cu(MeCN)BF4 (5.0 mg, 0.016 mmol, 8 mol%), acridine **A1** (4.7 mg, 0.016 mmol, 8 mol %), 4Å molecular sieves (60 mg), aldehyde (14.8 mg, 0.2 mmol), aniline (29.5 mg, 0.24 mmol, 1.2 equiv.), carboxylic acid (42.1 mg, 0.26 mmol, 1.3 equiv.), anhydrous *p*-toluenesulfonic acid (2.8 mg, 0.016 mmol, 8 mol%), and acetonitrile (2 mL). The test-tube was capped and the reaction mixture was irradiated with LED light (λ = 400 nm) while stirring at at room temperature for 30 h. The reaction mixture was then concentrated under reduced pressure, and the remaining material was purified by flash chromatography on silica gel (EtOAc/hexane, 1 : 20 v/v) to give product **7r (**48.8 mg, 75%) as a white solid (m.p. 49 ^{o}C).

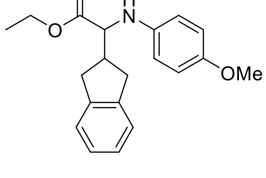

^{1}H NMR (500 MHz, $CDCl_3$): 7.23 – 7.09 (4 H, m), 6.77 (2 H, d, *J* = 8.9 Hz), 6.63 (2 H, d, *J* = 9.0 Hz), 4.13 (2 H, p, *J* = 7.0 Hz), 4.00 (2 H, d, *J* = 7.6 Hz), 3.74 (3 H, s), 3.13 (1 H, dd, *J* = 15.9, 8.1 Hz), 3.06 – 2.94 (3 H, m), 2.89 (1 H, p, *J* = 7.8 Hz), 1.22 (3 H, t, *J* = 7.1 Hz) ppm. – ^{13}C NMR (125 MHz, $CDCl_3$): 174.0, 153.0, 142.4, 142.2, 141.2, 126.6, 124.6, 115.4, 115.0, 61.8, 61.1, 55.9, 42.8, 36.3, 35.8, 14.4 ppm – IR: 1732, 1514, 1238, 1037 cm^{-1}. – HRMS: calcd for $C_{20}H_{23}NO_3$: 326.1751, found 326.1751 [M+H$^+$].

Go to table of contents

Ethyl 2-((4-methoxyphenyl)amino)-3,3-dimethylbutanoate (7s)

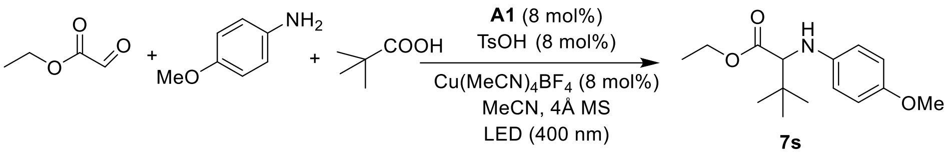

7s

According to GP1, the reaction was carried out with Cu(MeCN)BF_4 (5.0 mg, 0.016 mmol, 8 mol%), acridine **A1** (4.7 mg, 0.016 mmol, 8 mol %), 4Å molecular sieves (60 mg), aldehyde (14.8 mg, 0.2 mmol), aniline (29.5 mg, 0.24 mmol, 1.2 equiv.), carboxylic acid (26.5 mg, 0.26 mmol, 1.3 equiv.), anhydrous *p*-toluenesulfonic acid (2.8 mg, 0.016 mmol, 8 mol%), and acetonitrile (2 mL). The test-tube was capped and the reaction mixture was irradiated with LED light (λ = 400 nm) while stirring at at room temperature for 30 h. The reaction mixture was then concentrated under reduced pressure, and the remaining material was purified by flash chromatography on silica gel (EtOAc/hexane, 1 : 20 v/v) to give product **7s (**52.5 mg, 99%) as a colourless oil.

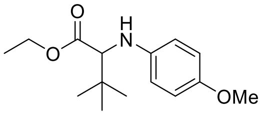

^{1}H NMR (500 MHz, $CDCl_3$): 6.76 (2 H, d, *J* = 8.9 Hz), 6.63 (2 H, d, *J* = 8.9 Hz), 4.13 (2 H, qd, *J* = 7.1, 5.3 Hz), 3.98 – 3.85 (1 H, m), 3.73 (3 H, s), 3.67 (1 H, d, *J* = 6.4 Hz), 1.22 (3 H, t, *J* = 7.1 Hz), 1.06 (9 H, s). ppm. – ^{13}C NMR (125 MHz, $CDCl_3$): 173.8, 152.9, 142.0, 115.7, 114.9, 67.2, 60.6, 55.8, 34.4, 26.9, 14.4 ppm – IR: 2970, 1737, 1514, 1366, 1230, 1036 cm^{-1}. – HRMS: calcd for $C_{15}H_{23}NO_3$: 266.1751, found 266.1751 [M+H$^+$].

Ethyl 2-((4-methoxyphenyl)amino)-3,3-dimethylpentanoate (7t)

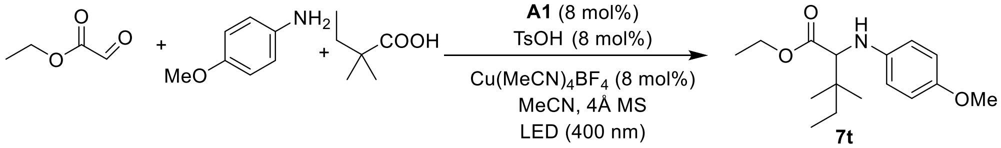

7t

According to GP1, the reaction was carried out with Cu(MeCN)BF_4 (5.0 mg, 0.016 mmol, 8 mol%), acridine **A1** (4.7 mg, 0.016 mmol, 8 mol %), 4Å molecular sieves (60 mg), aldehyde (14.8 mg, 0.2 mmol), aniline (29.5 mg, 0.24 mmol, 1.2 equiv.), carboxylic acid (30.2 mg, 0.26 mmol, 1.3 equiv.), anhydrous *p*-toluenesulfonic acid (2.8 mg, 0.016 mmol, 8 mol%), and acetonitrile (2 mL). The test-tube was capped and the reaction mixture was irradiated with LED light (λ = 400 nm) while stirring at at room temperature for 30 h. The reaction mixture was then concentrated under reduced pressure, and the remaining material was purified by flash chromatography on silica gel (EtOAc/hexane, 1 : 20 v/v) to give product **7t (**51.9 mg, 93%) as a colourless oil.

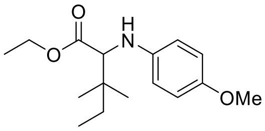

^{1}H NMR (500 MHz, $CDCl_3$): 6.76 (2 H, d, *J* = 9.0 Hz), 6.63 (2 H, d, *J* = 8.9 Hz), 4.27 – 4.02 (2 H, m), 3.93 – 3.83 (1 H, m), 3.73 (4 H, s), 1.55 – 1.34 (2 H, m), 1.21 (3 H, t, *J* = 7.1 Hz), 1.01 (3 H, s), 0.99 (3 H, s), 0.91 (3 H, t, *J* = 7.5 Hz) ppm. – ^{13}C NMR (125 MHz, $CDCl_3$): 173.9, 152.9, 141.9, 115.7, 114.9, 65.4, 60.6, 55.8, 37.0, 32.3, 23.5, 23.2, 14.4, 8.3 ppm – IR: 2970, 1738, 1514, 1365, 1217 cm^{-1}. – HRMS: calcd for $C_{16}H_{25}NO_3$: 280.1907, found 280.1908 [M+H$^+$].

Ethyl 2-((4-methoxyphenyl)amino)-2-(1-methylcyclohexyl)acetate (7u)

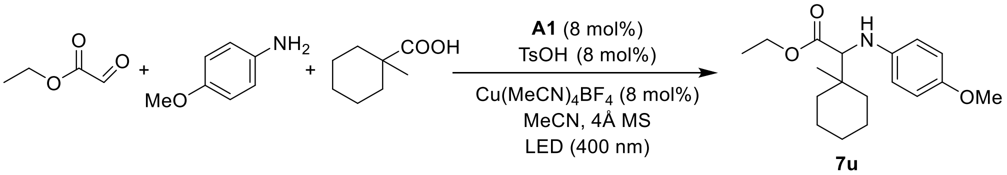

7u

According to GP1, the reaction was carried out with Cu(MeCN)BF$_4$ (5.0 mg, 0.016 mmol, 8 mol%), acridine **A1** (4.7 mg, 0.016 mmol, 8 mol %), 4Å molecular sieves (60 mg), aldehyde (14.8 mg, 0.2 mmol), aniline (29.5 mg, 0.24 mmol, 1.2 equiv.), carboxylic acid (36.9 mg, 0.26 mmol, 1.3 equiv.), anhydrous *p*-toluenesulfonic acid (2.8 mg, 0.016 mmol, 8 mol%), and acetonitrile (2 mL). The test-tube was capped and the reaction mixture was irradiated with LED light (λ = 400 nm) while stirring at at room temperature for 30 h. The reaction mixture was then concentrated under reduced pressure, and the remaining material was purified by flash chromatography on silica gel (EtOAc/hexane, 1 : 20 v/v) to give product **7u (**58.0 mg, 95%) as a colourless oil.

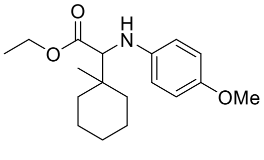

^{1}H NMR (500 MHz, CDCl$_3$): 6.75 (2 H, d, *J* = 8.9 Hz), 6.64 (2 H, d, *J* = 8.9 Hz), 4.12 (2 H, qd, *J* = 7.1, 4.7 Hz), 3.94 – 3.85 (1 H, m), 3.82 (1 H, s), 3.73 (3 H, s), 1.73 – 1.40 (8 H, m), 1.31 (2 H, dt, *J* = 13.2, 4.7 Hz), 1.21 (3 H, t, *J* = 7.1 Hz), 1.03 (3 H, s). ppm. – ^{13}C NMR (125 MHz, CDCl$_3$): 173.8, 152.9, 142.1, 115.8, 115.0, 66.2, 60.6, 55.9, 37.1, 35.1, 35.1, 26.3, 22.0, 21.8, 20.6, 14.5 ppm – IR: 2928, 1735, 1513, 1366, 1232, 1037 cm^{-1}. – HRMS: calcd for $C_{18}H_{27}NO_3$: 306.2064, found 306.2062 [M+H$^+$].

Ethyl 2-((4-methoxyphenyl)amino)-2-(4-methyltetrahydro-2H-pyran-4-yl)acetate (7v)

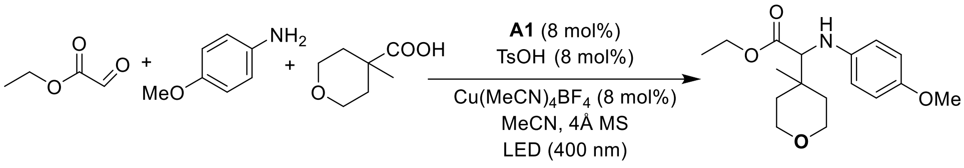

7v

According to GP1, the reaction was carried out with Cu(MeCN)BF$_4$ (5.0 mg, 0.016 mmol, 8 mol%), acridine **A1** (4.7 mg, 0.016 mmol, 8 mol %), 4Å molecular sieves (60 mg), aldehyde (14.8 mg, 0.2 mmol), aniline (29.5 mg, 0.24 mmol, 1.2 equiv.), carboxylic acid (37.4 mg, 0.26 mmol, 1.3 equiv.), anhydrous *p*-toluenesulfonic acid (2.8 mg, 0.016 mmol, 8 mol%), and acetonitrile (2 mL). The test-tube was capped and the reaction mixture was irradiated with LED light (λ = 400 nm) while stirring at at room temperature for 30 h. The reaction mixture was then concentrated under reduced pressure, and the remaining material was purified by flash chromatography on silica gel (EtOAc/hexane, 1 : 5 v/v) to give product **7v (**55.3 mg, 90%) as a colourless oil.

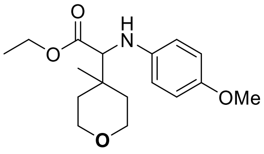

^{1}H NMR (500 MHz, CDCl$_3$): 6.80 – 6.72 (2 H, m), 6.68 – 6.60 (2 H, m), 4.22 – 4.07 (2 H, m), 3.90 (1 H, s), 3.81 (3 H, tt, *J* = 16.2, 4.3 Hz), 3.73 (3 H, d, *J* = 2.2 Hz), 3.70 – 3.58 (2 H, m), 1.88 (1 H, ddd, *J* = 14.3, 10.4, 4.4 Hz), 1.77 (1 H, ddd, *J* = 14.3, 10.2, 4.4 Hz), 1.59 – 1.48 (1 H, m), 1.31 (1 H, dq, *J* = 13.5, 2.2 Hz), 1.22 (3 H, t, *J* = 7.1 Hz), 1.14 (3 H, s) ppm. – ^{13}C NMR (125 MHz, CDCl$_3$): 173.1, 153.1, 141.7, 116.0, 115.0, 66.7, 63.9, 63.6, 60.8, 55.8, 35.2, 35.1, 35.0, 19.2, 14.4 ppm – IR: 2939, 1728, 1514, 1234, 1037 cm^{-1}. – HRMS: calcd for $C_{17}H_{25}NO_4$: 308.1856, found 308.1854 [M+H$^+$].

Ethyl 2-((4-methoxyphenyl)amino)-2-(1-phenylcyclohexyl)acetate (7w)

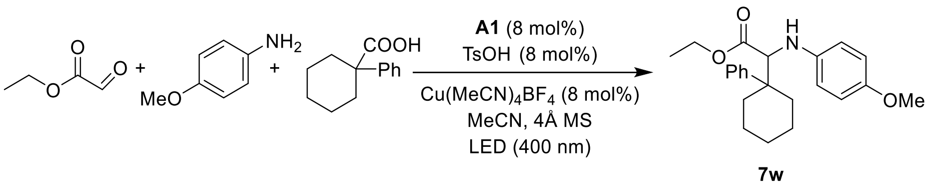

7w

According to GP1, the reaction was carried out with Cu(MeCN)BF_4 (5.0 mg, 0.016 mmol, 8 mol%), acridine **A1** (4.7 mg, 0.016 mmol, 8 mol %), 4Å molecular sieves (60 mg), aldehyde (14.8 mg, 0.2 mmol), aniline (29.5 mg, 0.24 mmol, 1.2 equiv.), carboxylic acid (53.0 mg, 0.26 mmol, 1.3 equiv.), anhydrous *p*-toluenesulfonic acid (2.8 mg, 0.016 mmol, 8 mol%), and acetonitrile (2 mL). The test-tube was capped and the reaction mixture was irradiated with LED light (λ = 400 nm) while stirring at at room temperature for 30 h. The reaction mixture was then concentrated under reduced pressure, and the remaining material was purified by flash chromatography on silica gel (EtOAc/hexane, 1 : 20 v/v) to give product **7w (**60.2 mg, 82%) as a white solid (m.p. 98 ^{o}C).

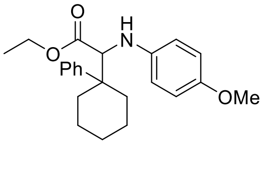

^{1}H NMR (500 MHz, $CDCl_3$): 7.40 – 7.34 (4 H, m), 7.31 – 7.20 (1 H, m), 6.79 – 6.67 (2 H, m), 6.59 – 6.51 (2 H, m), 3.99 – 3.81 (3 H, m), 3.72 (3 H, s), 3.68 (1 H, s), 2.65 – 2.49 (1 H, m), 2.45 – 2.29 (1 H, m), 2.05 – 1.87 (1 H, m), 1.83 – 1.50 (4 H, m), 1.42 – 1.22 (3 H, m), 1.01 (3 H, t, *J* = 7.1 Hz) ppm. – ^{13}C NMR (125 MHz, $CDCl_3$): 172.7, 152.9, 141.8, 140.9, 128.4, 128.2, 126.5, 115.9, 114.9, 68.4, 60.5, 55.8, 45.8, 39.0, 33.4, 33.0, 26.5, 22.2, 14.1 ppm – IR: 2935, 1737, 1512, 1366, 1217 cm^{-1}. – HRMS: calcd for $C_{23}H_{29}NO_3$: 368.2220, found 368.2220 [M+H$^+$].

Ethyl 2-((4-methoxyphenyl)amino)-3-methyl-3-phenylbutanoate (7x)

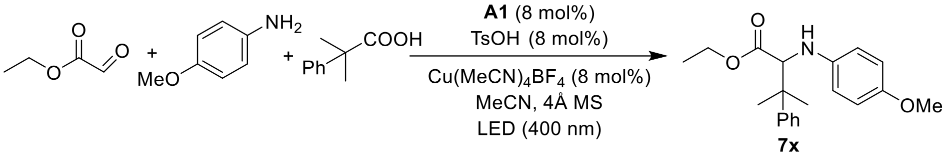

7x

According to GP1, the reaction was carried out with Cu(MeCN)BF_4 (5.0 mg, 0.016 mmol, 8 mol%), acridine **A1** (4.7 mg, 0.016 mmol, 8 mol %), 4Å molecular sieves (60 mg), aldehyde (14.8 mg, 0.2 mmol), aniline (29.5 mg, 0.24 mmol, 1.2 equiv.), carboxylic acid (42.6 mg, 0.26 mmol, 1.3 equiv.), anhydrous *p*-toluenesulfonic acid (2.8 mg, 0.016 mmol, 8 mol%), and acetonitrile (2 mL). The test-tube was capped and the reaction mixture was irradiated with LED light (λ = 400 nm) while stirring at at room temperature for 30 h. The reaction mixture was then concentrated under reduced pressure, and the remaining material was purified by flash chromatography on silica gel (EtOAc/hexane, 1 : 20 v/v) to give product **7x (**49.7 mg, 76%) as a colourless oil.

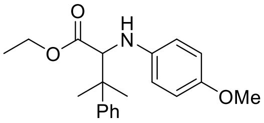

^{1}H NMR (500 MHz, $CDCl_3$): 7.45 – 7.39 (2 H, m), 7.33 (2 H, dd, *J* = 8.6, 6.9 Hz), 7.27 – 7.20 (1 H, m), 6.72 (2 H, d, *J* = 8.9 Hz), 6.55 (2 H, d, *J* = 8.9 Hz), 4.04 (1 H, s), 3.93 (2 H, qd, *J* = 7.2, 1.3 Hz), 3.84 – 3.78 (1 H, m), 3.72 (3 H, s), 1.53 (3 H, s), 1.50 (3 H, s), 1.00 (3 H, t, *J* = 7.1 Hz) ppm. – ^{13}C NMR (125 MHz, $CDCl_3$)173.2, 153.0, 145.7, 141.7, 128.2, 126.7, 126.6, 115.9, 114.9, 67.9, 60.6, 55.8, 41.5, 25.7, 25.3, 14.1 ppm – IR: 2975, 1727, 1512, 1239, 1032, 700 cm^{-1}. – HRMS: calcd for $C_{20}H_{25}NO_3$: 328.1907, found 328.1907 [M+H$^+$].

Ethyl 2-((3r,5r,7r)-adamantan-1-yl)-2-((4-methoxyphenyl)amino)acetate (7y)

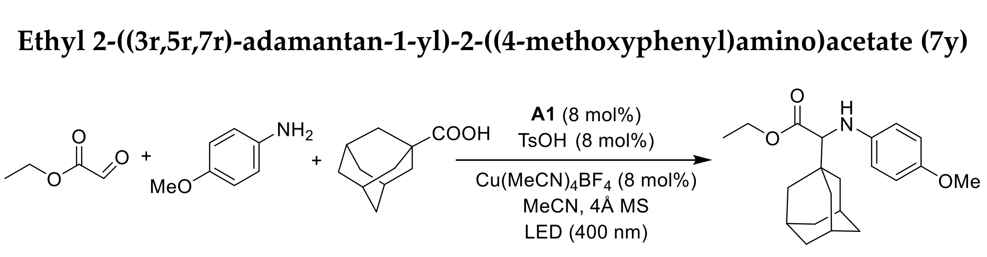

7x

According to GP1, the reaction was carried out with Cu(MeCN)BF_4 (5.0 mg, 0.016 mmol, 8 mol%), acridine **A1** (4.7 mg, 0.016 mmol, 8 mol %), 4Å molecular sieves (60 mg), aldehyde (14.8 mg, 0.2 mmol), aniline (29.5 mg,

0.24 mmol, 1.2 equiv.), carboxylic acid (46.8 mg, 0.26 mmol, 1.3 equiv.), anhydrous *p*-toluenesulfonic acid (2.8 mg, 0.016 mmol, 8 mol%), and acetonitrile (2 mL). The test-tube was capped and the reaction mixture was irradiated with LED light (λ = 400 nm) while stirring at at room temperature for 30 h. The reaction mixture was then concentrated under reduced pressure, and the remaining material was purified by flash chromatography on silica gel (EtOAc/hexane, 1 : 20 v/v) to give product **7y (**55.6 mg, 81%) as a colourless oil.

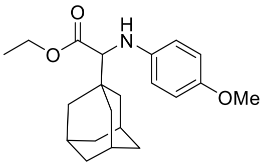

^{1}H NMR (500 MHz, $CDCl_3$): 6.75 (2 H, d, *J* = 8.9 Hz), 6.63 (2 H, d, *J* = 8.9 Hz), 4.13 (2 H, q, *J* = 7.2 Hz), 3.92 (1 H, s), 3.73 (3 H, s), 3.53 (1 H, d, *J* = 4.7 Hz), 2.06 – 1.98 (3 H, m), 1.82 (3 H, dt, *J* = 12.3, 2.7 Hz), 1.73 (3 H, dt, *J* = 12.4, 2.7 Hz), 1.69 – 1.63 (3 H, m), 1.58 (3 H, dq, *J* = 12.3, 2.6 Hz), 1.23 (3 H, t, *J* = 7.1 Hz) ppm. – ^{13}C NMR (125 MHz, $CDCl_3$): 173.3, 152.8, 142.1, 115.6, 114.9, 68.1, 60.5, 55.8, 39.2, 37.0, 37.0, 36.3, 28.5, 14.5 ppm – IR: 2901, 2848, 1728, 1513, 1240, 1037 cm^{-1}. – HRMS: calcd for $C_{21}H_{29}NO_3$: 344.2220, found 344.2216 [M+H$^+$].

Isopropyl 2-cyclohexyl-2-((4-methoxyphenyl)amino)acetate (7z)

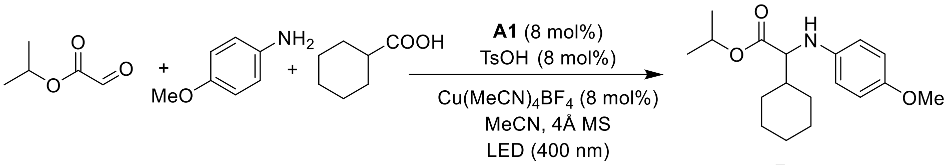

7z

According to GP1, the reaction was carried out with Cu(MeCN)BF_4 (5.0 mg, 0.016 mmol, 8 mol%), acridine **A1** (4.7 mg, 0.016 mmol, 8 mol %), 4Å molecular sieves (60 mg), aldehyde (17.6 mg, 0.2 mmol), aniline (29.5 mg, 0.24 mmol, 1.2 equiv.), carboxylic acid (33.3 mg, 0.26 mmol, 1.3 equiv.), anhydrous *p*-toluenesulfonic acid (2.8 mg, 0.016 mmol, 8 mol%), and acetonitrile (2 mL). The test-tube was capped and the reaction mixture was irradiated with LED light (λ = 400 nm) while stirring at at room temperature for 30 h. The reaction mixture was then concentrated under reduced pressure, and the remaining material was purified by flash chromatography on silica gel (EtOAc/hexane, 1 : 20 v/v) to give product **7z (**54.3 mg, 89%) as a white solid (m.p. 93 ^{o}C).

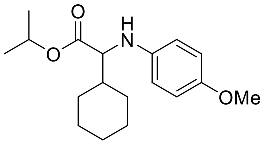

^{1}H NMR (500 MHz, $CDCl_3$): 6.75 (2 H, d, *J* = 8.9 Hz), 6.60 (2 H, d, *J* = 8.9 Hz), 5.02 (1 H, p, *J* = 6.3 Hz), 3.87 (1 H, d, *J* = 12.1 Hz), 3.73 (4 H, s), 2.00 – 1.84 (1 H, m), 1.82 – 1.56 (5 H, m), 1.44 – 1.07 (11 H, m) ppm. – ^{13}C NMR (125 MHz, $CDCl_3$): 173.6, 152.7, 141.9, 115.3, 114.9, 68.4, 63.5, 55.9, 41.4, 29.8, 29.4, 26.4, 26.3, 26.2, 22.0, 22.0 ppm – IR: 2928, 1728, 1514, 1239, 1106 cm^{-1}. – HRMS: calcd for $C_{18}H_{27}NO_3$: 306.2064, found 306.2056 [M+H$^+$].

Benzyl 2-cyclohexyl-2-((4-methoxyphenyl)amino)acetate (7za)

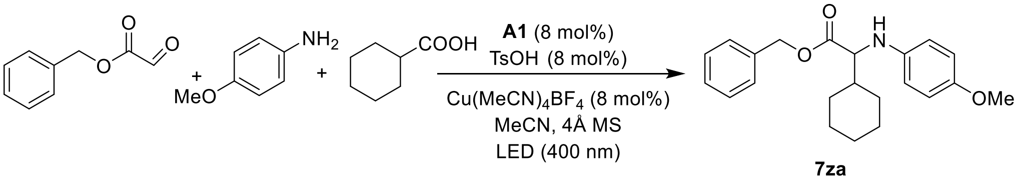

7za

According to GP1, the reaction was carried out with Cu(MeCN)BF_4 (5.0 mg, 0.016 mmol, 8 mol%), acridine **A1** (4.7 mg, 0.016 mmol, 8 mol %), 4Å molecular sieves (60 mg), aldehyde (14.8 mg, 0.2 mmol), aniline (29.5 mg, 0.24 mmol, 1.2 equiv.), carboxylic acid (27.2 mg, 0.26 mmol, 1.3 equiv.), anhydrous *p*-toluenesulfonic acid (2.8 mg, 0.016 mmol, 8 mol%), and acetonitrile (2 mL). The test-tube was capped and the reaction mixture was irradiated with LED light (λ = 400 nm) while stirring at at room temperature for 30 h. The reaction mixture was

then concentrated under reduced pressure, and the remaining material was purified by flash chromatography on silica gel (EtOAc/hexane, 1 : 20 v/v) to give product **7za** (57.9 mg, 82%) as a colourless oil.

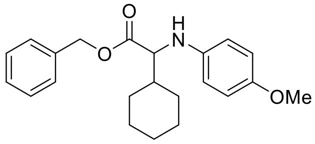

^{1}H NMR (500 MHz, $CDCl_3$): 7.33 (3 H, td, *J* = 4.7, 2.2 Hz), 7.29 – 7.21 (2 H, m), 6.75 (2 H, d, *J* = 8.9 Hz), 6.60 (2 H, d, *J* = 8.9 Hz), 5.13 (2 H, d, *J* = 2.2 Hz), 3.85 (2 H, t, *J* = 10.0 Hz), 3.74 (3 H, s), 1.91 – 1.81 (1 H, m), 1.80 – 1.72 (3 H, m), 1.71 – 1.61 (2 H, m), 1.33 – 1.06 (5 H, m) ppm. – ^{13}C NMR (125 MHz, $CDCl_3$): 174.1, 152.8, 141.7, 135.8, 128.6, 128.4, 115.4, 115.0, 66.6, 63.6, 55.8, 41.4, 29.8, 29.3, 26.3, 26.2 ppm – IR: 2927, 1734, 1514, 1239, 821 cm^{-1}. – HRMS: calcd for $C_{22}H_{27}NO_3$: 354.2064, found 354.2063 [M+H$^+$].

N-(5-(2,5-Dimethylphenoxy)-2,2-dimethyl-1-phenylpentyl)aniline (8a)

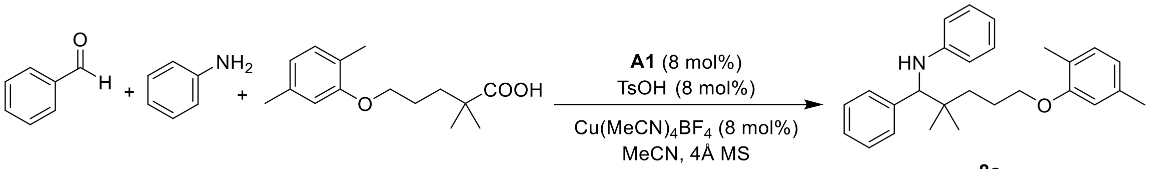

8a

According to GP1, the reaction was carried out with Cu(MeCN)BF_4 (5.0 mg, 0.016 mmol, 8 mol%), acridine **A1** (4.7 mg, 0.016 mmol, 8 mol %), 4Å molecular sieves (60 mg), aldehyde (21.2 mg, 0.2 mmol), aniline (22.3 mg, 0.24 mmol, 1.2 equiv.), carboxylic acid (65.0 mg, 0.26 mmol, 1.3 equiv.), anhydrous *p*-toluenesulfonic acid (2.8 mg, 0.016 mmol, 8 mol%), and acetonitrile (2 mL). The test-tube was capped and the reaction mixture was irradiated with LED light (λ = 400 nm) while stirring at at room temperature for 30 h. The reaction mixture was then concentrated under reduced pressure, and the remaining material was purified by flash chromatography on silica gel (EtOAc/hexane, 1 : 20 v/v) to give product **8a** (75.1 mg, 97%) as a colourless oil.

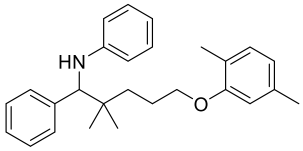

^{1}H NMR (500 MHz, $CDCl_3$): 7.35 – 7.24 (4 H, m), 7.24 – 7.17 (1 H, m), 7.04 (2 H, dd, *J* = 8.6, 7.2 Hz), 7.00 (1 H, d, *J* = 7.4 Hz), 6.65 (1 H, d, *J* = 7.4 Hz), 6.62 – 6.55 (2 H, m), 6.49 (2 H, d, *J* = 7.9 Hz), 4.16 (1 H, s), 3.99 – 3.82 (2 H, m), 2.29 (3 H, s), 2.15 (3 H, s), 1.94 – 1.72 (2 H, m), 1.69 – 1.42 (2 H, m), 1.01 (3 H, s), 0.96 (3 H, s) ppm. – ^{13}C NMR (125 MHz, $CDCl_3$): 157.1, 136.6, 130.4, 129.2, 128.8, 127.9, 127.0, 123.7, 120.8, 117.2, 113.4, 112.1, 68.5, 37.4, 36.4, 24.7, 24.3, 23.9, 21.5, 15.9 ppm – IR: 2951, 1738, 1600, 1504, 1205, 1152, 704 cm^{-1}. – HRMS: calcd for $C_{27}H_{33}NO$: 388.2635, found 388.2637 [M+H$^+$].

Ethyl 6-(2,5-dimethylphenoxy)-2-((4-methoxyphenyl)amino)-3,3-dimethylhexanoate (8b)

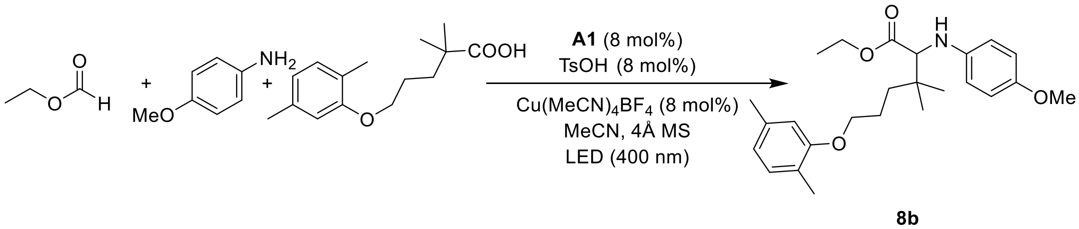

8b

According to GP1, the reaction was carried out with Cu(MeCN)BF_4 (5.0 mg, 0.016 mmol, 8 mol%), acridine **A1** (4.7 mg, 0.016 mmol, 8 mol %), 4Å molecular sieves (60 mg), aldehyde (14.8 mg, 0.2 mmol), aniline (29.5 mg, 0.24 mmol, 1.2 equiv.), carboxylic acid (65.0 mg, 0.26 mmol, 1.3 equiv.), anhydrous *p*-toluenesulfonic acid (2.8 mg, 0.016 mmol, 8 mol%), and acetonitrile (2 mL). The test-tube was capped and the reaction mixture was irradiated with LED light (λ = 400 nm) while stirring at at room temperature for 30 h. The reaction mixture was

then concentrated under reduced pressure, and the remaining material was purified by flash chromatography on silica gel (EtOAc/hexane, 1 : 20 v/v) to give product **8b (**79.3 mg, 96%) as a colourless oil.

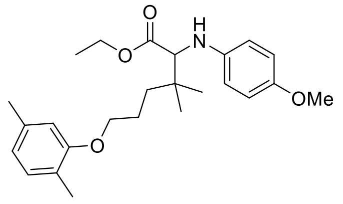

[1] H NMR (500 MHz, $CDCl_3$): 7.02 (1 H, d, *J* = 7.4 Hz), 6.78 (2 H, d, *J* = 8.9 Hz), 6.70 – 6.63 (3 H, m), 6.63 (1 H, d, *J* = 1.5 Hz), 4.15 (2 H, qd, *J* = 7.1, 3.1 Hz), 3.95 (3 H, t, *J* = 6.4 Hz), 3.83 – 3.77 (1 H, m), 3.75 (3 H, s), 2.32 (3 H, s), 2.19 (3 H, s), 2.03 – 1.76 (2 H, m), 1.71 – 1.50 (2 H, m), 1.23 (3 H, t, *J* = 7.1 Hz), 1.10 (3 H, s), 1.08 (3 H, s) ppm. – ^{13}C NMR (125 MHz, $CDCl_3$): 173.7, 157.1, 153.0, 141.8, 136.6, 130.4, 123.7, 120.8, 115.9, 114.9, 112.1, 68.4, 65.8, 60.7, 55.8, 36.8, 36.2, 24.2, 24.1, 23.7, 21.5, 15.9, 14.4 ppm – IR: 2919, 1728, 1511, 1366, 1234 cm^{-1}. – HRMS: calcd for $C_{25}H_{35}NO_4$: 414.2639, found 414.2639 [M+H$^+$].

Ethyl 2-((4-methoxyphenyl)amino)-3-(11-oxo-6,11-dihydrodibenzo[*b*,*e*]oxepin-2-yl)propanoate (8c)

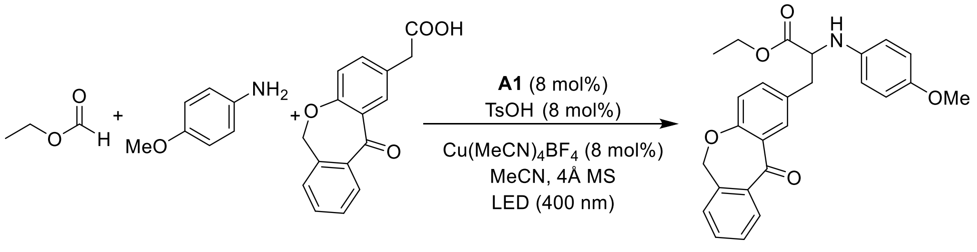

According to GP1, the reaction was carried out with Cu(MeCN)BF_4 (5.0 mg, 0.016 mmol, 8 mol%), acridine **A1** (4.7 mg, 0.016 mmol, 8 mol %), 4Å molecular sieves (60 mg), aldehyde (14.8 mg, 0.2 mmol), aniline (29.5 mg, 0.24 mmol, 1.2 equiv.), carboxylic acid (69.7 mg, 0.26 mmol, 1.3 equiv.), anhydrous *p*-toluenesulfonic acid (2.8 mg, 0.016 mmol, 8 mol%), and acetonitrile (2 mL). The test-tube was capped and the reaction mixture was irradiated with LED light (λ = 400 nm) while stirring at at room temperature for 30 h. The reaction mixture was then concentrated under reduced pressure, and the remaining material was purified by flash chromatography on silica gel (EtOAc/hexane, 1 : 10 v/v) to give product **8c (**79.3 mg, 92%) as a colourless oil.

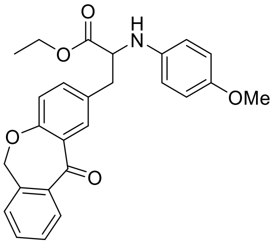

[1] H NMR (500 MHz, $CDCl_3$): 8.06 (1 H, d, *J* = 2.4 Hz), 7.89 (1 H, dd, *J* = 7.7, 1.4 Hz), 7.55 (1 H, td, *J* = 7.4, 1.4 Hz), 7.47 (1 H, td, *J* = 7.6, 1.3 Hz), 7.35 (1 H, dd, *J* = 7.5, 1.2 Hz), 7.31 (1 H, dd, *J* = 8.4, 2.4 Hz), 6.98 (1 H, d, *J* = 8.4 Hz), 6.76 (1 H, d, *J* = 9.0 Hz), 6.60 (2 H, d, *J* = 8.9 Hz), 5.17 (2 H, s), 4.27 (1 H, t, *J* = 6.3 Hz), 4.15 (2 H, qd, *J* = 7.1, 1.4 Hz), 3.73 (3 H, s), 3.11 (2 H, qd, *J* = 13.7, 6.2 Hz), 1.21 (3 H, t, *J* = 7.1 Hz) ppm. – ^{13}C NMR (125 MHz, $CDCl_3$): 191.0, 173.3, 160.5, 152.9, 140.5, 136.6, 135.7, 132.9, 132.5, 130.5, 129.6, 129.3, 127.9, 125.2, 120.9, 115.5, 115.0, 73.7, 61.3, 59.0, 55.8, 37.9, 14.3 ppm – IR: 2932, 1732, 1647, 1513, 1300, 1240, 823 cm^{-1}. – HRMS: calcd for $C_{26}H_{25}NO_5$: 432.1805, found 432.1805 [M+H$^+$].

Ethyl 3-(1-(4-chlorobenzoyl)-5-methoxy-2-methyl-1*H*-indol-3-yl)-2-((4-methoxyphenyl)amino)propanoate (8d)

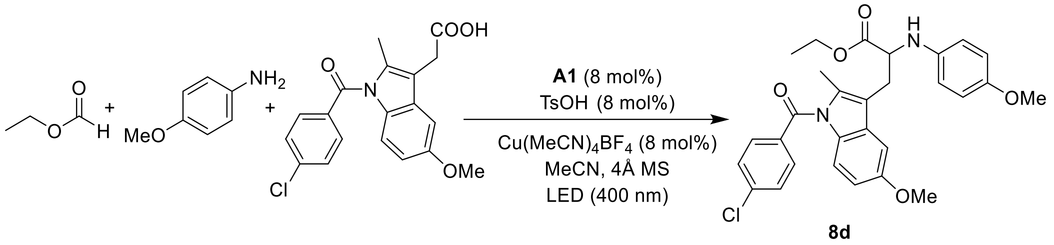

According to GP1, the reaction was carried out with Cu(MeCN)BF_4 (5.0 mg, 0.016 mmol, 8 mol%), acridine **A1** (4.7 mg, 0.016 mmol, 8 mol %), 4Å molecular sieves (60 mg), aldehyde (14.8 mg, 0.2 mmol), aniline (29.5 mg, 0.24 mmol, 1.2 equiv.), carboxylic acid (93.1 mg, 0.26 mmol, 1.3 equiv.), anhydrous *p*-toluenesulfonic acid (2.8 mg, 0.016 mmol, 8 mol%), and acetonitrile (2 mL). The test-tube was capped and the reaction mixture was irradiated with LED light (λ = 400 nm) while stirring at at room temperature for 30 h. The reaction mixture was then concentrated under reduced pressure, and the remaining material was purified by flash chromatography on silica gel (EtOAc/hexane, 1 : 8 v/v) to give product **8d (**96.9 mg, 93%) as a colourless oil.

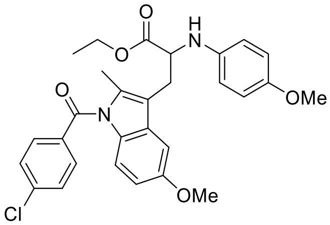

^{1}H NMR (500 MHz, $CDCl_3$): 7.57 (2 H, d, *J* = 8.1 Hz), 7.49 – 7.40 (2 H, m), 6.96 (1 H, d, *J* = 2.5 Hz), 6.93 (1 H, d, *J* = 9.0 Hz), 6.73 (2 H, d, *J* = 8.9 Hz), 6.68 (1 H, dd, *J* = 9.0, 2.5 Hz), 6.54 (2 H, d, *J* = 8.9 Hz), 4.30 (1 H, t, *J* = 6.4 Hz), 4.17 – 3.99 (2 H, m), 3.80 (3 H, s), 3.72 (3 H, s), 3.30 – 3.08 (2 H, m), 2.29 (3 H, s), 1.14 (3 H, t, *J* = 7.1 Hz) ppm. – ^{13}C NMR (125 MHz, $CDCl_3$): 173.7, 168.4, 156.1, 152.9, 140.7, 139.3, 135.8, 134.1, 131.3, 131.2, 131.0, 129.2, 115.1, 115.0, 114.9, 111.6, 101.4, 61.4, 57.9, 55.8, 28.4, 14.2, 13.7 ppm – IR: 1732, 1682, 1513, 1477, 1316, 1237, 1037, 824 cm^{-1}. – HRMS: calcd for $C_{29}H_{29}ClN_2O_5$: 521.1838, found 521.1838 [M+H$^+$].

(3*R*,7*R*,8*R*,9*R*,10*S*,13*R*,14*R*,17*R*)-17-((2*R*)-6-Ethoxy-5-((4-methoxyphenyl)amino)-6-oxohexan-2-yl)-8,10,13-trimethylhexadecahydro-1*H*-cyclopenta[a]phenanthrene-3,7-diyl diacetate (8e)

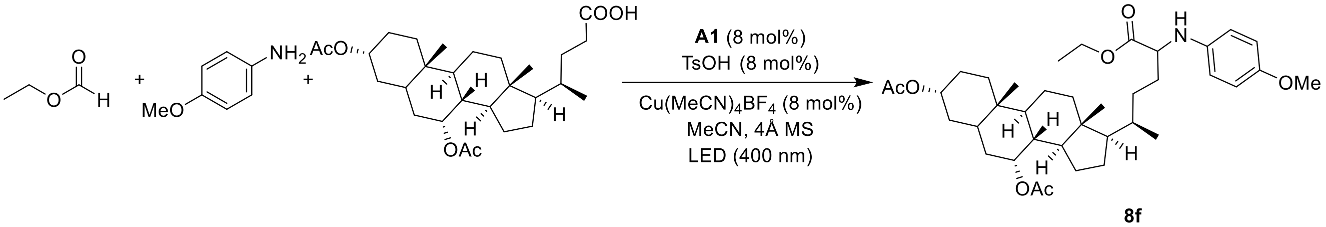

According to GP1, the reaction was carried out with Cu(MeCN)BF_4 (5.0 mg, 0.016 mmol, 8 mol%), acridine **A1** (4.7 mg, 0.016 mmol, 8 mol %), 4Å molecular sieves (60 mg), aldehyde (14.8 mg, 0.2 mmol), aniline (29.5 mg, 0.24 mmol, 1.2 equiv.), carboxylic acid (127.4 mg, 0.26 mmol, 1.3 equiv.), anhydrous *p*-toluenesulfonic acid (2.8 mg, 0.016 mmol, 8 mol%), and acetonitrile (2 mL). The test-tube was capped and the reaction mixture was irradiated with LED light (λ = 400 nm) while stirring at at room temperature for 30 h. The reaction mixture was then concentrated under reduced pressure, and the remaining material was purified by flash chromatography on silica gel (EtOAc/hexane, 1 : 3 v/v) to give product **8e (**118.8 mg, 1:1 dr, 91%) as a colourless oil. $[\alpha]^{20}_{D}$ = + 3.5 (c = 1.0, CH_2Cl_2).

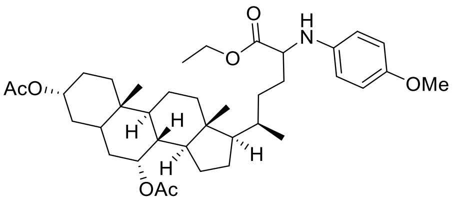

[1] H NMR (500 MHz, $CDCl_3$): 6.75 (2 H, d, *J* = 8.9 Hz), 6.58 (2 H, dd, *J* = 8.9, 1.5 Hz), 4.87 (1 H, d, *J* = 3.1 Hz), 4.58 (1 H, tt, *J* = 11.4, 4.5 Hz), 4.25 – 4.07 (2 H, m), 3.96 – 3.85 (1 H, m), 3.72 (3 H, s), 2.05 (3 H, s), 2.02 (3 H, s), 2.00 – 1.90 (2 H, m), 1.90 – 1.75 (4 H, m), 1.74 – 1.63 (2 H, m), 1.63 – 1.52 (3 H, m), 1.45 (5 H, dddd, *J* = 23.4, 12.1, 8.9, 5.4 Hz), 1.38 – 0.99 (12 H, m), 0.95 – 0.86 (6 H, m), 0.63 (3 H d, *J* = 5.8 Hz,) ppm. – ^{13}C NMR (125 MHz, $CDCl_3$): 174.7, 174.6, 170.7, 170.5, 152.8, 141.2, 141.1, 115.3, 115.0, 74.3, 71.4, 61.0, 58.4, 55.8, 55.8, 50.5, 42.8, 41.1, 39.6, 38.0, 35.6, 35.5, 35.0, 34.9, 34.8, 34.2, 31.8, 31.7, 31.4, 29.8, 29.8, 28.2, 28.1, 26.9, 23.7, 22.8, 21.7, 21.6, 20.8, 18.7, 14.4, 11.8 ppm – IR: 2940, 1732, 1513, 1246, 1025, 758 cm^{-1}. – HRMS: calcd for $C_{38}H_{57}NO_7$: 640.4208, found 640.4209 [M+H$^+$].

1-Cyclohexyl 5-ethyl (2*S*)-2-((tert-butoxycarbonyl)amino)-4-((4-methoxyphenyl)amino)pentanedioate (8f)

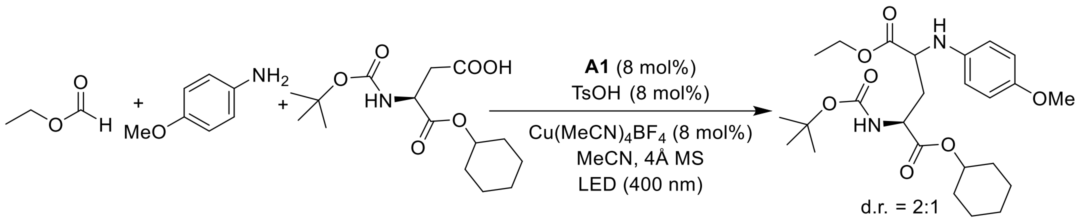

8g

According to GP1, the reaction was carried out with Cu(MeCN)BF_4 (5.0 mg, 0.016 mmol, 8 mol%), acridine **A1** (4.7 mg, 0.016 mmol, 8 mol %), 4Å molecular sieves (60 mg), aldehyde (14.8 mg, 0.2 mmol), aniline (29.5 mg, 0.24 mmol, 1.2 equiv.), carboxylic acid (81.9 mg, 0.26 mmol, 1.3 equiv.), anhydrous *p*-toluenesulfonic acid (2.8 mg, 0.016 mmol, 8 mol%), and acetonitrile (2 mL). The test-tube was capped and the reaction mixture was irradiated with LED light (λ = 400 nm) while stirring at at room temperature for 30 h. The reaction mixture was then concentrated under reduced pressure, and the remaining material was purified by flash chromatography on silica gel (EtOAc/hexane, 1 : 2 v/v) to give product **8f (**80.3 mg, 2:1 dr, 84%) as a white solid (m.p. 79 ^oC). $[\alpha]^{20}_D$ = 0 (c = 0.5, CH_2Cl_2).

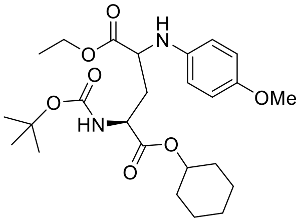

[1] H NMR (500 MHz, $CDCl_3$, rotamers): 6.79 – 6.70 (3 H, m), 6.63 (3 H, dd, *J* = 8.9, 4.1 Hz), 5.22 (1.5 H, dd, *J* = 15.3, 9.5 Hz), 4.78 (1.5 H, dq, *J* = 9.0, 4.4 Hz), 4.50 (1 H, q, *J* = 4.7 Hz), 4.35 (0.5 H, d, *J* = 7.0 Hz), 4.27 (0.5 H, s), 4.26 – 4.07 (0.5 H, m), 3.72 (4.5 H, s), 2.66 (1.5 H, ddd, *J* = 25.0, 15.9, 6.8 Hz), 2.53 (1.5 H, ddd, *J* = 15.8, 5.9, 2.8 Hz), 1.89 – 1.77 (3 H, m), 1.76 – 1.60 (3 H, m), 1.56 – 1.30 (22.5 H, m), 1.25 (4.5 H, dt, *J* = 10.3, 7.1 Hz) ppm. – ^{13}C NMR (125 MHz, $CDCl_3$): 172.4, 172.3, 170.7, 155.3, 153.3, 153.1, 141.3, 123.7, 116.0, 115.7, 115.0, 114.9, 114.6, 79.8, 77.4, 77.2, 76.9, 73.4, 73.4, 61.8, 61.6, 61.2, 61.1, 55.8, 55.8, 50.1, 49.8, 37.0, 36.3, 31.6, 31.6, 28.4, 28.4, 25.4, 23.8, 14.3, 14.2 ppm – IR: 2936, 1720, 1512, 1237, 1170, 1038 cm^{-1}. – HRMS: calcd for $C_{25}H_{38}N_2O_7$: 479.2752, found 479.2755 [M+H$^+$].

1-Benzyl 6-ethyl (2*S*)-2-(((benzyloxy)carbonyl)amino)-5-((4-methoxyphenyl)amino)hexanedioate (8g)

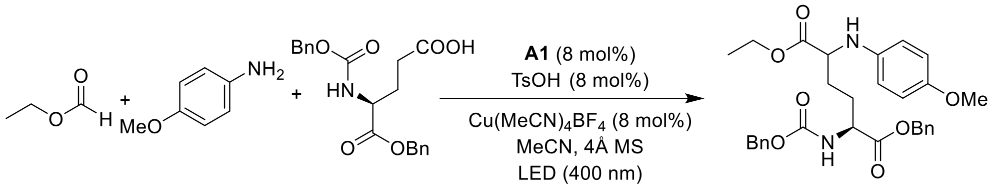

8h

According to GP1, the reaction was carried out with Cu(MeCN)BF_4 (5.0 mg, 0.016 mmol, 8 mol%), acridine **A1** (4.7 mg, 0.016 mmol, 8 mol %), 4Å molecular sieves (60 mg), aldehyde (14.8 mg, 0.2 mmol), aniline (29.5 mg, 0.24 mmol, 1.2 equiv.), carboxylic acid (96.5 mg, 0.26 mmol, 1.3 equiv.), anhydrous *p*-toluenesulfonic acid (2.8 mg, 0.016 mmol, 8 mol%), and acetonitrile (2 mL). The test-tube was capped and the reaction mixture was irradiated with LED light (λ = 400 nm) while stirring at at room temperature for 30 h. The reaction mixture was then concentrated under reduced pressure, and the remaining material was purified by flash chromatography on silica gel (EtOAc/hexane, 1 : 2 v/v) to give product **8g (**91.8 mg, 1:1 dr, 86%) as a colourless oil. $[\alpha]^{20}_{D}$ = 0 (c = 0.5, CH_2Cl_2).

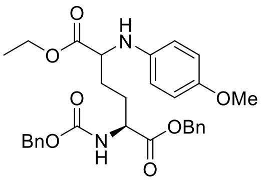

^{1}H NMR (500 MHz, $CDCl_3$): 7.33 (10 H, dq, *J* = 10.0, 4.8, 4.3 Hz), 6.74 (2 H, d, *J* = 8.9 Hz), 6.57 (2 H, t, *J* = 8.1 Hz), 5.41 (1 H, dd, *J* = 29.9, 8.2 Hz), 5.27 – 4.99 (4 H, m), 4.47 (1 H, q, *J* = 6.9 Hz), 4.20 – 4.05 (2 H, m), 3.93 (1 H, dt, *J* = 16.0, 5.2 Hz), 3.73 (3 H, d, *J* = 1.5 Hz), 2.02 (1 H, dt, *J* = 15.7, 5.2 Hz), 1.94 – 1.75 (2 H, m), 1.66 (1 H, dt, *J* = 11.3, 8.1 Hz), 1.19 (3 H, td, *J* = 7.1, 5.0 Hz) ppm. – ^{13}C NMR (125 MHz, $CDCl_3$): 173.9, 172.0, 153.1, 140.9, 140.7, 136.3, 135.3, 128.8, 128.7, 128.5, 128.4, 128.2, 115.6, 115.0, 67.5, 67.5, 67.2, 62.7, 61.3, 57.6, 55.8, 53.8, 29.2, 29.2, 29.0, 28.8, 14.3 ppm – IR: 1724, 1513, 1239, 1183, 1029, 699 cm^{-1}. – HRMS: calcd for $C_{30}H_{34}N_2O_7$: 535.2439, found 535.2439 [M+H$^+$].

1-Ethyl 6-((3a*S*,5a*R*,8a*R*,8b*S*)-2,2,7,7-tetramethyltetrahydrobenzo[1,2-d:3,4-*d*']bis([1,3]dioxole)-3a(4*H*)-yl) 2-((4-methoxyphenyl)amino)hexanedioate (8h)

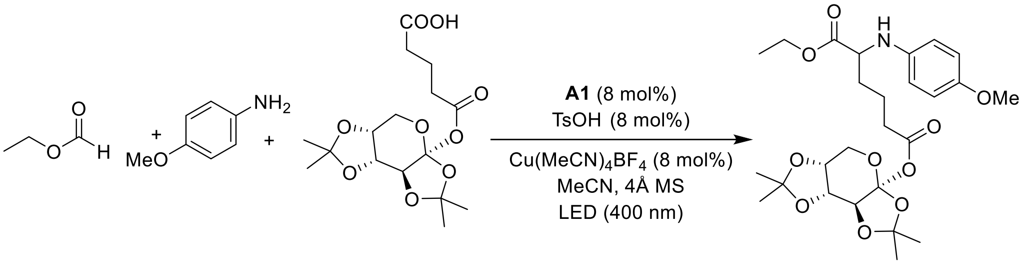

8i

According to GP1, the reaction was carried out with Cu(MeCN)BF_4 (5.0 mg, 0.016 mmol, 8 mol%), acridine **A1** (4.7 mg, 0.016 mmol, 8 mol %), 4Å molecular sieves (60 mg), aldehyde (14.8 mg, 0.2 mmol), aniline (29.5 mg, 0.24 mmol, 1.2 equiv.), carboxylic acid (90.1 mg, 0.26 mmol, 1.3 equiv.), anhydrous *p*-toluenesulfonic acid (2.8 mg, 0.016 mmol, 8 mol%), and acetonitrile (2 mL). The test-tube was capped and the reaction mixture was irradiated with LED light (λ = 400 nm) while stirring at at room temperature for 30 h. The reaction mixture was then concentrated under reduced pressure, and the remaining material was purified by flash chromatography on silica gel (EtOAc/hexane, 1 : 1 v/v) to give product **8h (**96.9 mg, 1:1 dr, 93%) as a colourless oil. $[\alpha]^{20}_{D}$ = - 0.60 (c = 1.0, CH_2Cl_2).

Go to table of contents

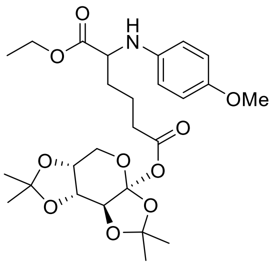

[1] H NMR (500 MHz, $CDCl_3$): 6.75 (2 H, d, *J* = 8.9 Hz), 6.58 (2 H, d, *J* = 8.9 Hz), 4.59 (1 H, dt, *J* = 7.9, 2.4 Hz), 4.40 (1 H, dd, *J* = 11.7, 8.2 Hz), 4.28 (1 H, dd, *J* = 2.6, 1.8 Hz), 4.23 (1 H, dt, *J* = 8.0, 1.8 Hz), 4.15 (2 H, qd, *J* = 7.1, 0.9 Hz), 4.04 (1 H, dd, *J* = 11.7, 8.9 Hz), 3.95 (1 H, dt, *J* = 6.1, 3.1 Hz), 3.90 (1 H, dt, *J* = 12.9, 2.0 Hz), 3.80 – 3.70 (5 H, m), 2.46 – 2.36 (2 H, m), 1.94 – 1.70 (4 H, m), 1.53 (3 H, s), 1.48 (3 H, s), 1.38 (3 H, s), 1.34 (3 H, d, *J* = 1.6 Hz), 1.23 (3 H, t, *J* = 7.1 Hz) ppm. – ^{13}C NMR (125 MHz, $CDCl_3$): 174.2, 172.5, 152.9, 141.1, 116.7, 115.3, 115.0, 114.9, 109.3, 108.8, 101.6, 70.9, 70.7, 70.2, 65.5, 65.5, 61.4, 61.2, 57.7, 55.8, 33.7, 32.5, 26.6, 26.0, 25.3, 24.2, 21.2, 21.1, 14.4 ppm – IR: 2987, 2935, 1736, 1514, 1250, 1209, 1071, 823 cm^{-1}. – HRMS: calcd for $C_{26}H_{37}NO_{10}$: 538.2647, found 538.2635 [M+H$^+$].

Computational studies

Calculations were performed using computational resources at the Texas Advanced Computing Centers (TACC) hosted by The University of Texas at Austin and the Advanced Cyberinfrastructure Coordination Ecosystem: Services and Support (ACCESS). DFT optimization, vibrational analysis, and IRC calculations were conducted with Gaussian 16 (rA.03).[1] Energy decomposition analysis was performed for the optimized transition state structures using the second generation energy decomposition analysis using absolutely localized molecular orbitals (ALMO-EDA2)[2] and complementary occupied virtual orbital pairs (COVP) methods in Q-chem 6.0.[3] Visualizations and monitoring of calculations were performed using Chemcraft.[4] Images were rendered using CYLview2.0[5] and VMD 1.9.3.[6] Spin density information was collected from the optimized geometry check file and later rendered in VMD using an isovalue of 0.05. The contribution of each atom to the spin density was evaluated using NBOpro7.[7] Calculations related to effective oxidation states (EOS) and intrinsic bonding orbitals (IBOs) were performed with IboView.[8]

Details of computational methods

Ground state minima and transition states were optimized without constraints using PW6B95 density functional approximation with D3BJ dispersion correction and Def2-TZVP basis set in acetonitrile using the SMD solvation model. Optimizations were performed with "tight" convergence criteria and an ultrafine grid. Frequency calculations at the same level of theory were used to confirm the nature of the stationary points. Geometries with no imaginary frequencies were deemed minima, whereas those with exactly one imaginary frequency along the chemical path of interest were deemed transition states. An IRC calculation was performed for each transition state to further corroborate the transition state connected reactants and products. A cut-off frequency of 50 cm^{-1} was applied for all structures to correct for potential errors associated with low magnitude vibrational frequencies, in addition to aa 1M concentration correction, via GoodVibes.[9] Single point calculations were performed at the M06-L-D3/def2-TZVPPD level of theory in acetonitrile using the SMD solvation model.

Distortion/Interaction-Activation Strain Analysis

A distortion/interaction-activation strain model analysis[10] was performed on **TS1**, **TS2**, and **TS3** at the M06-L–D3/Def2-TZVPPD/SMD(MeCN) level of theory from the previously optimized geometries. A detailed discussion of distortion/interaction-activation strain analysis can be found in our previous work.[11] Fragment definitions were created for each transition state (Figure S1), with the red fragment representing the imine analogues (Fragment **F1**) and the green fragment representing the radical structure (Fragment **F2**). The results of distortion/interaction-activation strain analysis are provided in Figure S2.

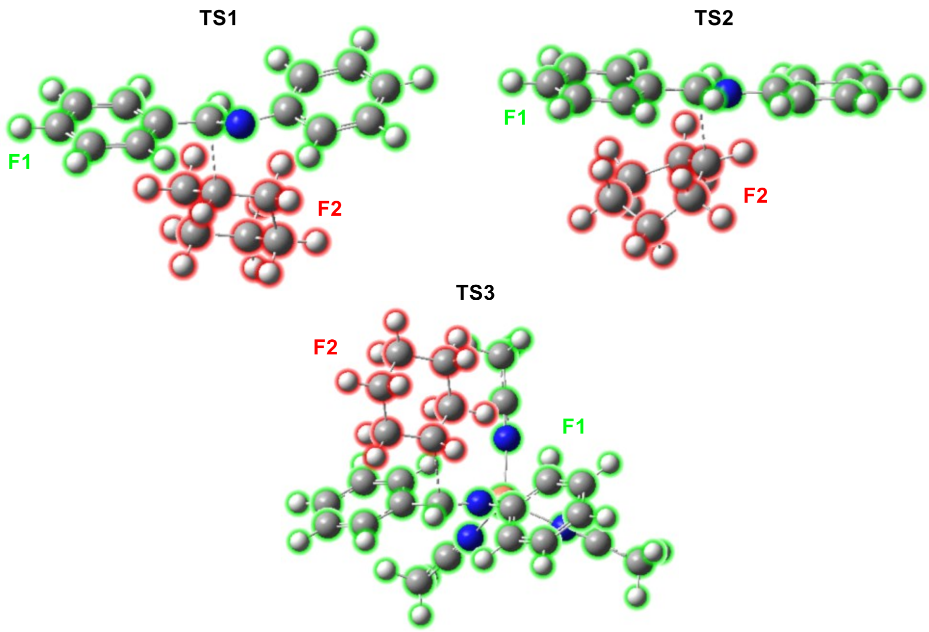

Figure S1. Fragment definition for distortion/interaction-activation strain analysis. The imine fragment is highlighted green (**F1**), and the radical fragment is highlighted red (**F2**).

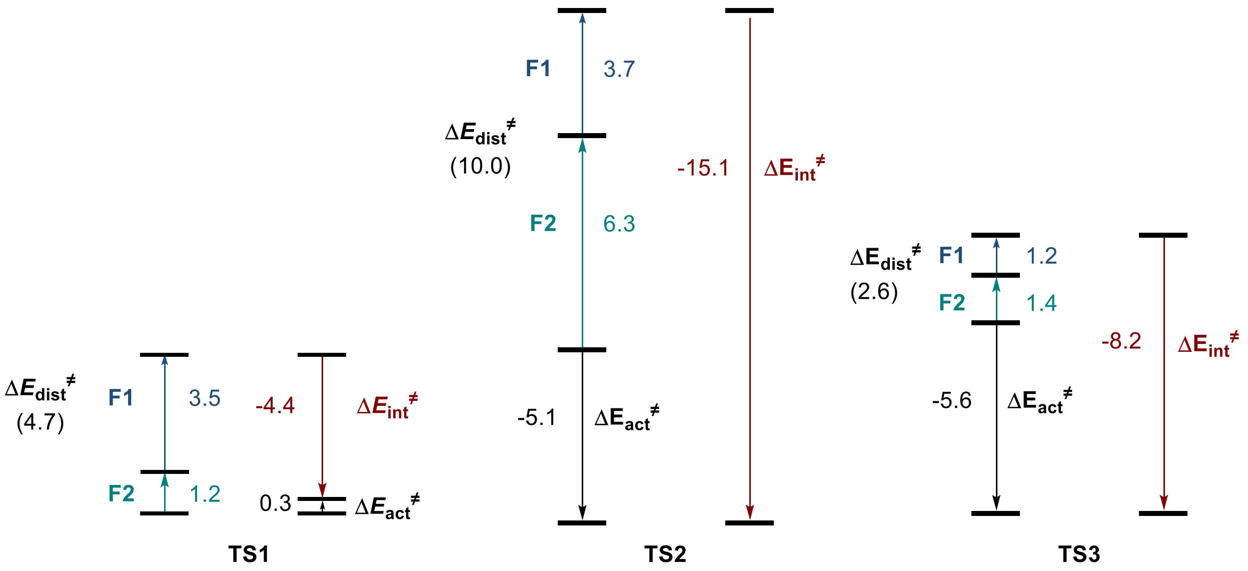

Figure S2. Results of distortion/interaction-activation strain analysis in kcal/mol for **TS1**, **TS2**, and **TS3**.

Energy Decomposition Analysis via ALMO-EDA2

The second generation Absolutely Localized Molecular Orbital Energy Decomposition Analysis (ALMO-EDA2) method of Head-Gordon and co-workers was employed to gain quantitative insight into the intermolecular forces governing the interaction energies of the previously optimized transition state structures. ALMO-EDA2 calculations were performed at the M06-L–D3/def2-TZVPPD/SMD(MeCN) level of theory in Q-Chem 6.0 using the optimized geometries at the same level of theory. The results of ALMO-EDA2 are visualized in Figure SY1 and tabulated in Table S2.

Go to table of contents

Table S2. Energy decomposition analysis of **TS1**, **TS2**, and **TS3**, kcal/mol.

Structure	prep	ΔE_{Pauli}	ΔE_{Elec}	ΔE_{CT}	ΔE_{Disp}	ΔE_{Pol}	ΔE_{Solv}	Total $\Delta E^{\ddagger}$ *int*
**TS1**	0	56.4	-23.1	-19.7	-15.6	-3.7	1.2	-4.4
**TS2**	0	56.8	-25.3	-27.2	-17.6	-3.9	1.9	-15.2
**TS3**	0	57.1	-24.6	-20.5	-18.3	-4.0	1.8	-8.4

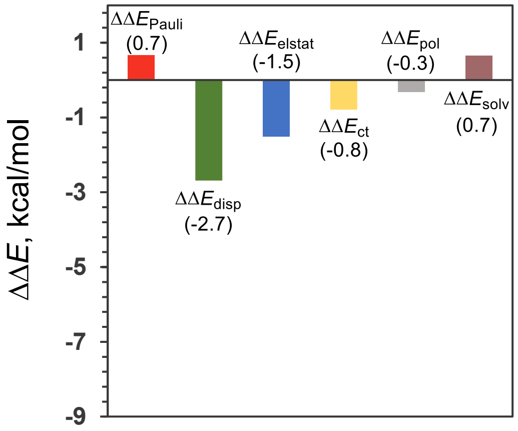

Figure S3. Energy decomposition analysis for **TS3** with respect to **TS1**, $\Delta\Delta E^{\neq}$, = $\Delta E^{\neq}_{TS2}$ - $\Delta E^{\neq}_{TS1}$, kcal/mol.

Complementary occupied-virtual orbital pairs (COVP) analysis

The complementary occupied-virtual orbital pairs (COVP) analysis was employed in tandem with ALMO-EDA2. This method provides insight into the donor/acceptor orbital interactions that contribute to the $\Delta E_{CT}^{\neq}$ term. The images were generated in VMD using an isovalue of ±0.01 from for the two COVPs that contributed the most to the charge transfer term. The donor orbitals are represented with an opaque surface while acceptor orbitals are represented by transparent surface. The results for **TS1**, **TS2**, and **TS3** are presented in Figures S4-S6.

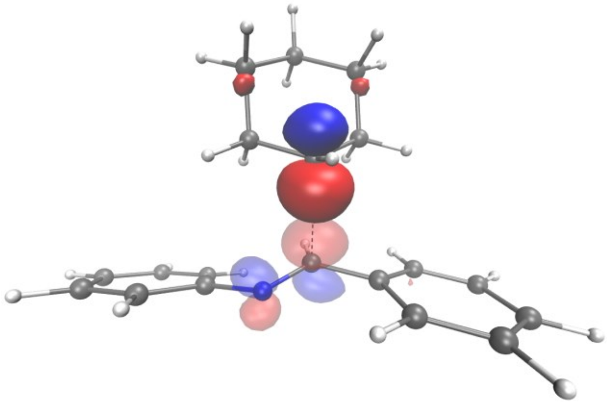

TS1α

(-13.1)

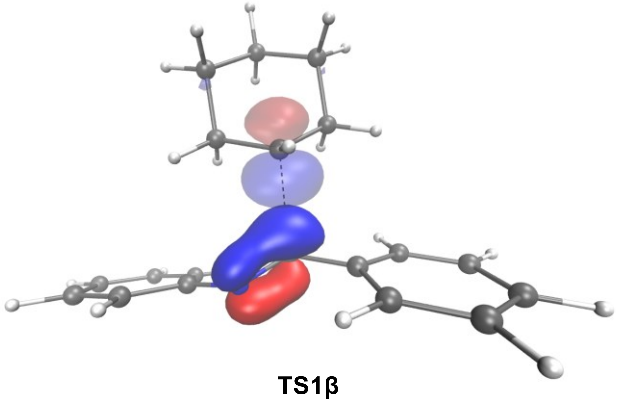

TS1β

(-2.6)

Figure S4. The most significant COVPs for **TS1** and their energy contribution in kcal/mol.

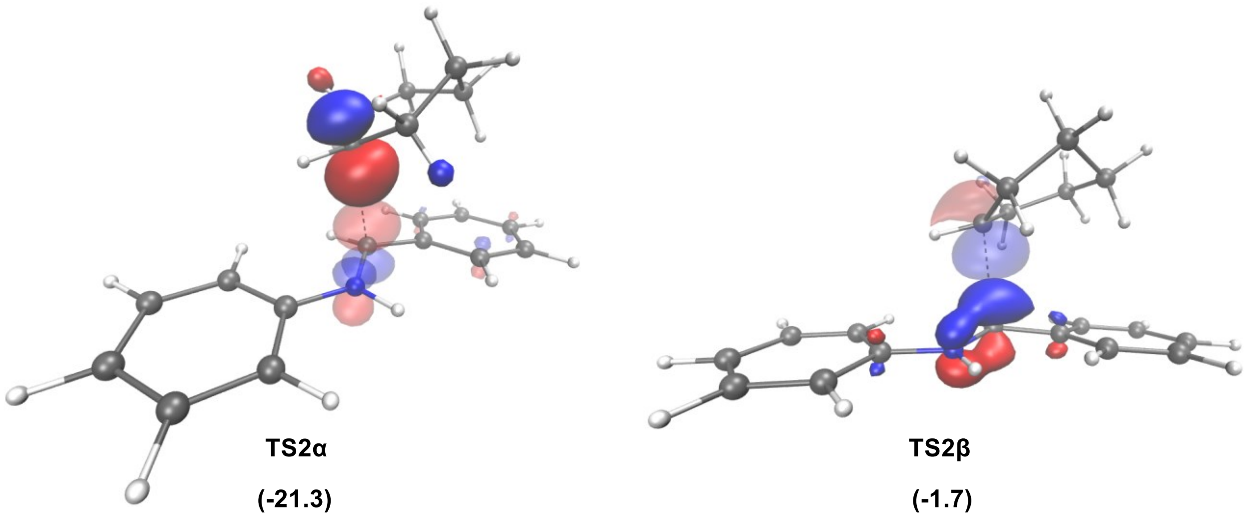

Figure S5. The most significant COVPs for **TS2** and their energy contribution in kcal/mol.

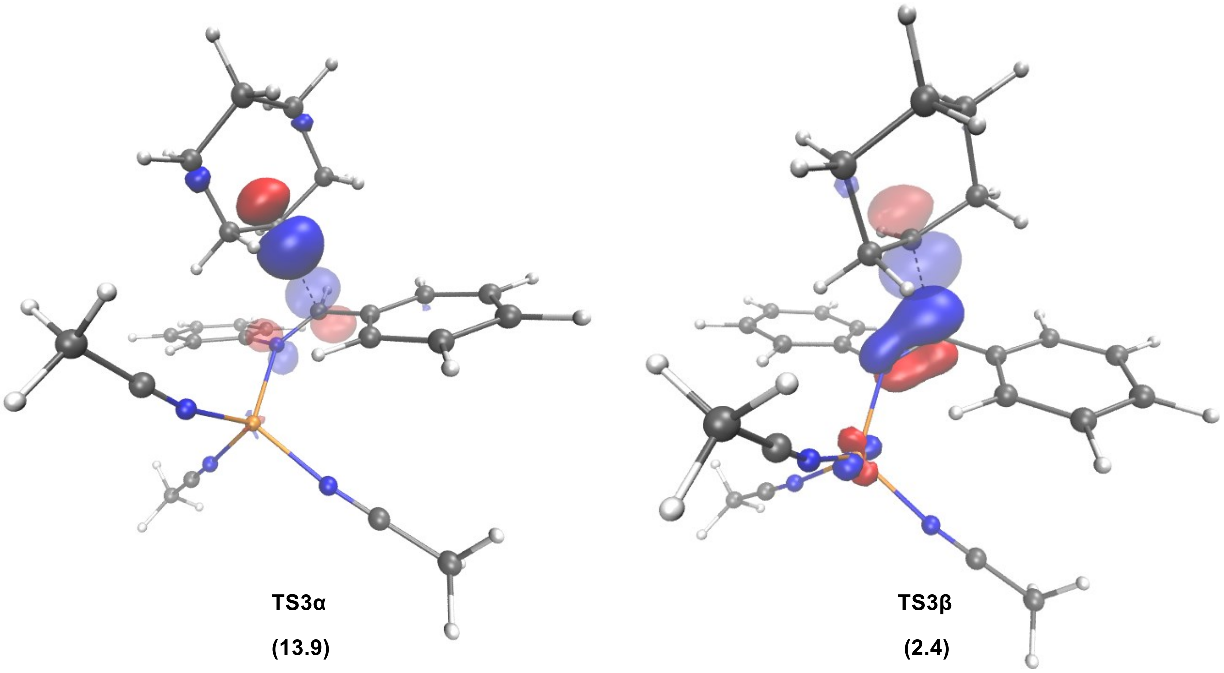

Figure S6. The most significant COVPs for **TS3** and their energy contribution in kcal/mol.

Effective oxidation state analysis

Calculations were performed on structures **11** and **12** to determine the effective oxidation states (EOS)[12] of the metal center and ligands (Figures S7 and S8). Each complex was partitioned into fragments and the electronic population of each fragment was evaluated using intrinsic bonding orbitals (IBOs).[13] Wavefunctions for IBO analysis were performed at the PBE0/Def2-SVP level of theory on the optimized geometries collected from G16.

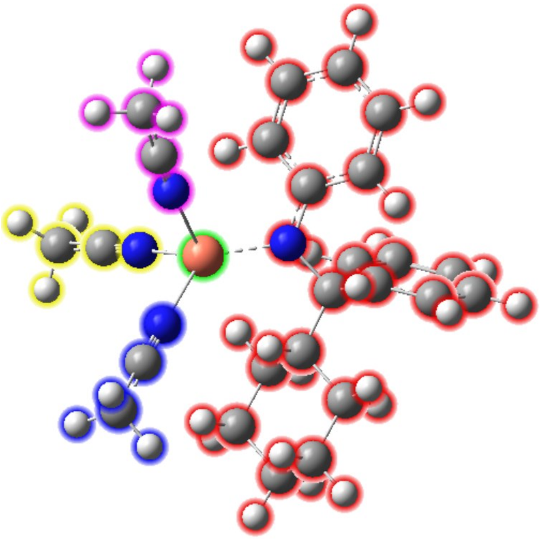

Fragment	Partial Charge	Partial Spin
Imine	-0.03	0.80
Copper	0.83	0.18
MeCN (blue)	0.07	0.01
MeCN (yellow)	0.06	0.00
MeCN (purple)	0.07	0.01

Figure S7. Fragment definitions used in EOS analysis for **11** and the partial charge and partial spin of each fragment.

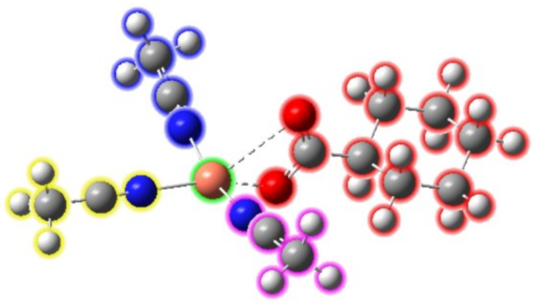

Fragment	Partial Charge	Partial Spin
Imine	-0.47	0.37
Copper	1.07	0.48
MeCN (blue)	0.13	0.06
MeCN (yellow)	0.11	0.03
MeCN (purple)	0.14	0.06

Figure S8. Fragment definitions used in EOS analysis for **12** and the partial charge and partial spin of each fragment.

Marcus theory calculations

The following equations, derived from Marcus-Hush Theory,[14] can approximate the SET process:

$$\Delta G_{ET}^{\ddagger} = \Delta G_0^{\ddagger}\left(1 + \frac{\Delta G_r}{4\Delta G_0}\right)^2$$

$$\Delta G_0 = \frac{\lambda}{4}$$

$$\lambda_0 = \left(332\,\frac{kcal}{mol}\right)\left(\frac{1}{2a_1} + \frac{1}{2a_2} + \frac{1}{R}\right)\left(\frac{1}{\varepsilon_{op}} - \frac{1}{\varepsilon}\right)$$

The intrinsic barrier, $\Delta G_0^{\ddagger}$, is estimated by first determining the reorganization energy, λ. As the inner reorganization is expected to have a small contribution to λ, the reorganization energy is approximated by the

outer reorganization energy, $\lambda_0 \approx \lambda$. The a_1 term is the sphere radius of the donor species; a_2 is the sphere radius of the acceptor species; ε_{op} is the square of the refractive index of solvent acetonitrile; ε is the dielectric constant of the solvent reported from G16; and *R* is the inter-center distance between the donor and acceptor.

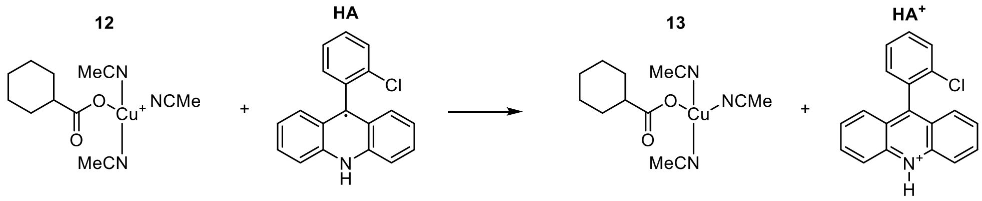

a_1 / Å	a_2 / Å	ε_{op}	ε	λ_0	ΔG_r	$\Delta G^{\ddagger}_{ET}$
9.58	13.07	1.81	35.69	16.15	-16.5	0.0018

Non-covalent interaction analysis

The independent gradient model (IGM)[15] was employed to detect non-covalent interactions present in **TS1** and **TS2**. Cube files were generated on MultiWFN[16] from the optimized PW6B95-D3BJ/Def2-TZVP/SMD(MeCN) geometries. The files were then exported to VMD for visualization using an isovalue of 0.01 (Figure S9).

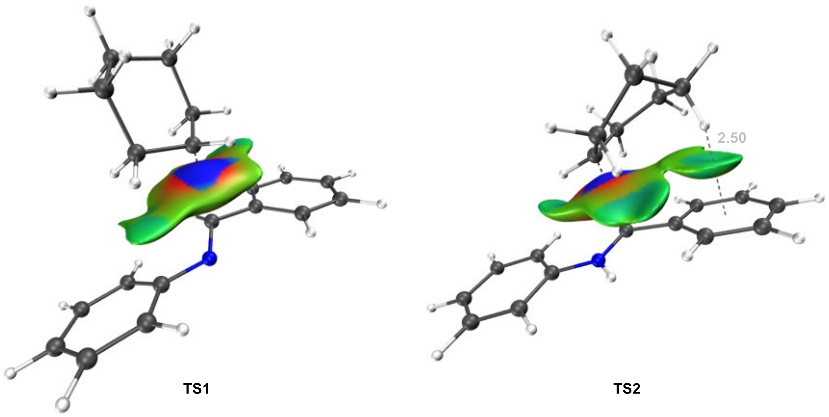

Figure S9. Independent gradient model for **TS1** and **TS2**.

Optimized geometries

R·

E(UPW6B95D3) = -235.607139049

E(UM06L) = -235.269087994

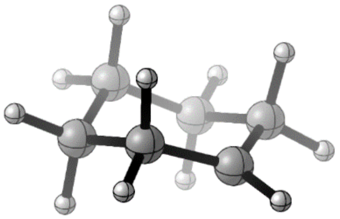

Charge = 0 Multiplicity = 2

C	-2.1776123781	-0.9994810159	0.1290995819
C	-0.6632243592	-0.8883624349	-0.0404584075
C	-0.2265618794	0.5657087826	-0.1163686307
C	-0.6588117321	1.3310170887	1.1238744905
C	-2.1732306264	1.2595702528	1.3144597423
C	-2.6769466577	-0.1307692864	1.2208076543

H	0.8541734547	0.6270476685	-0.2376024278
H	-0.1728971437	-1.3630587080	0.8110816041
H	-0.3499875986	-1.4295412345	-0.9320150410
H	-2.6437542826	-0.6964764339	-0.8208834379
H	-2.4730925050	-2.0349234266	0.2900292428
H	-0.1683470822	0.8984361208	1.9974865926
H	-0.3420848552	2.3710640342	1.0618866758
H	-2.4654523026	1.7166898181	2.2583857813
H	-2.6387223001	1.8706539655	0.5261118785
H	-3.5709400117	-0.4112445218	1.7588996788
H	-0.6710148300	1.0301171806	-0.9998849681

RCO_2H

E(RPW6B95D3) = -425.153611424

E(RM06L) = -424.569853863

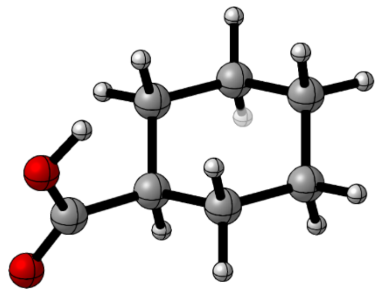

Charge = 0 Multiplicity = 1

C	-2.4068217162	-1.3075391786	0.0136961514
C	-0.8880224908	-1.3309663493	0.0687342457
C	-0.3305400829	0.0929928070	0.0345139268
C	-0.9000351083	0.9173226041	1.1914182682
C	-2.4186428231	0.9360384826	1.1328366289
C	-2.9878098266	-0.4731506636	1.1435325536
H	-0.5743404487	-1.8262241265	0.9908971867
H	-0.4819396761	-1.9088873142	-0.7595298855
H	-2.7210875110	-0.8885927969	-0.9439183039
H	-2.7907004750	-2.3252942980	0.0558691483
H	-0.5885257021	0.4795773910	2.1424068431
H	-0.5009596453	1.9294839668	1.1575856357
H	-2.8098401766	1.5109787329	1.9703813494
H	-2.7335673323	1.4472376720	0.2213859832
H	-2.7497306575	-0.9478192870	2.0977148563
H	-4.0734670563	-0.4412729765	1.0679220081
H	-0.6249780638	0.5606942107	-0.9027917454
C	1.1729244071	0.1043393851	0.0376193357
O	1.7735277003	-0.4596764635	1.0990404950
H	1.1167167549	-0.8032287091	1.7186937108
O	1.8559755701	0.5785255210	-0.8295109621

$Cu^{I}L_3^{+}$

E(RPW6B95D3) = -2040.27052865

E(RM06L) = -2038.82910189

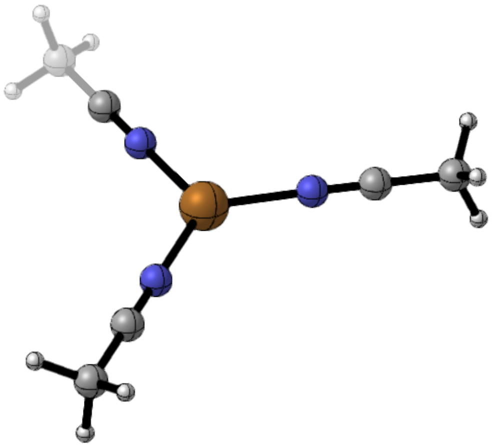

Charge = 1	Multiplicity = 1		
Cu	0.1830660908	-0.0620514084	-0.1116717420
N	0.0275021135	0.1102921371	1.8535775356
N	1.9507609554	-0.1017328752	-0.9762612575
N	-1.4821453647	-0.0635087200	-1.1946238023
C	-0.0674816884	0.1991134216	2.9899472512
C	-2.4371970155	-0.0307898517	-1.8231260680
C	2.9833442282	-0.1410055158	-1.4666513286
C	-3.6374004273	0.0121039133	-2.6151761705
H	-3.9164544440	1.0473538460	-2.7904182375
H	-3.4619605814	-0.4813370971	-3.5669484673
H	-4.4401556325	-0.4938570159	-2.0865261365
C	-0.1874311156	0.3081416750	4.4193587247
H	-1.0720213993	0.8882062703	4.6665800016
H	-0.2753579405	-0.6855517762	4.8496812406
H	0.6934161047	0.7998890753	4.8221003408
C	4.2828606029	-0.1938466026	-2.0814520318
H	4.9450146583	0.5147422579	-1.5920114525
H	4.6883439755	-1.1966621894	-1.9799985175
H	4.1977386489	0.0569487245	-3.1349606724

9

E(RPW6B95D3) = -557.711544033

E(RM06L) = -556.890262700

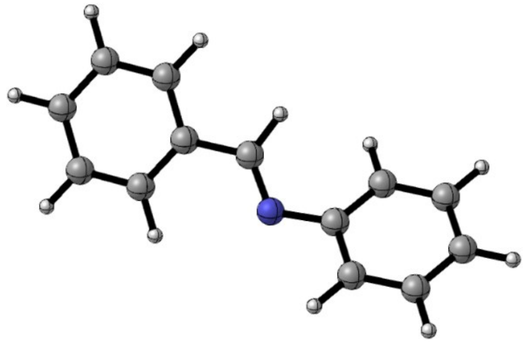

Charge = 0	Multiplicity = 1		
C	-1.5494837499	0.0656027264	0.5013483670
C	-0.3122088336	0.6837737241	0.5284134556
C	-0.1639421406	1.9867435047	0.0638285418
C	-1.2761433859	2.6656808651	-0.4310537587
C	-2.5088109477	2.0474604961	-0.4588495233
C	-2.6487481096	0.7465401402	0.0072950878
H	-1.6556561079	-0.9448757592	0.8643254950
H	0.5510915984	0.1599593282	0.9122769632
H	-1.1585632677	3.6758825521	-0.7906811164
H	-3.3672505326	2.5762637469	-0.8435387039

H	-3.6153914250	0.2673050671	-0.0158356758
C	1.1576913991	2.6002198313	0.1128156317
H	1.9525263175	1.9732510326	0.5204974179
N	1.3872431482	3.7785777490	-0.3008377517
C	2.6718094086	4.3167161991	-0.1693383239
C	3.1373708374	5.1515110073	-1.1815496509
C	3.4752908693	4.0902077700	0.9468689184
C	4.3973725472	5.7132500808	-1.1002496087
H	2.5014465933	5.3410475249	-2.0328570013
C	4.7289841284	4.6686567522	1.0292401563
H	3.1034831750	3.4832698669	1.7583308120
C	5.1993933763	5.4746398314	0.0050859042
H	4.7515911702	6.3471960816	-1.8987425219
H	5.3385041611	4.4942043239	1.9029177904
H	6.1774267505	5.9248509376	0.0743730752

10

E(UPW6B95D3) = -793.354110699

E(UM06L) = -792.192071306

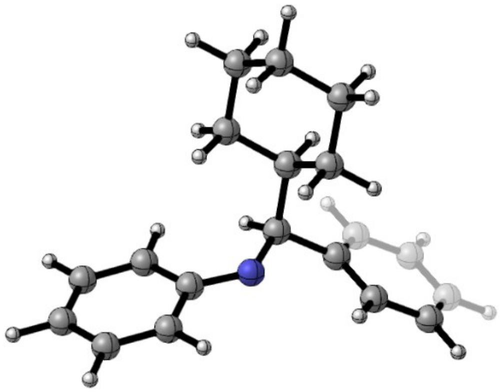

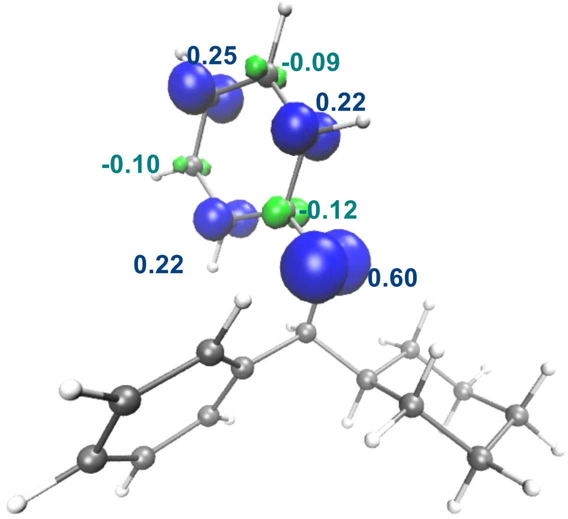

Charge = 0 Multiplicity = 2

C	-2.1158165680	1.8011550543	0.9384445596
C	-0.8354113427	2.2336954312	0.6323451525
C	-0.1764747506	1.7492232553	-0.4888469674
C	-0.8250769567	0.8255384375	-1.2990407285
C	-2.1036687254	0.3925679494	-0.9959194786
C	-2.7545411777	0.8796417071	0.1266717306
H	-2.6144439340	2.1870714619	1.8147394918
H	-0.3457267438	2.9534029462	1.2699485205
H	-0.3222854757	0.4483230683	-2.1782714444
H	-2.5947972061	-0.3215467683	-1.6395372792
H	-3.7531679970	0.5459921015	0.3635465858
C	1.2395520880	2.1631797815	-0.8382010688
N	1.6165000334	3.4125432285	-0.2265817122
C	1.1044777482	4.5484535913	-0.7139559585
C	1.5228673443	5.7528152785	-0.0927370326
C	0.1776423952	4.6485876976	-1.7846847215
C	1.0526299272	6.9716323048	-0.5131339656
H	2.2257919121	5.6764552562	0.7226783718
C	-0.2856991600	5.8762109506	-2.1914329308

H	-0.1735680152	3.7579485692	-2.2787206430
C	0.1445856805	7.0428558885	-1.5653830757
H	1.3860148518	7.8755063595	-0.0271329674
H	-0.9928368386	5.9366178228	-3.0046437120
H	-0.2269738426	8.0001934320	-1.8959005821
C	2.2477801718	1.0695119442	-0.4486533138
C	3.6325137459	1.4037806093	-0.9851355710
C	2.3049648811	0.8140879974	1.0502149367
H	1.9079011987	0.1523807948	-0.9367805615
C	4.6432430364	0.3204088207	-0.6464369933
H	3.9547273375	2.3512056818	-0.5479687966
H	3.5810754291	1.5546197462	-2.0640823791
C	3.3145376222	-0.2716408690	1.3898471948
H	2.5855208649	1.7410678347	1.5526772492
H	1.3193059760	0.5372963868	1.4219324307
C	4.6944063339	0.0689147890	0.8517864534
H	5.6285728654	0.5967453403	-1.0199292201
H	4.3611851429	-0.6046223482	-1.1543341821
H	3.3535680689	-0.4205799412	2.4682602401
H	2.9846388881	-1.2169744765	0.9530785303
H	5.3987916246	-0.7305111356	1.0786651127
H	5.0619158491	0.9673146997	1.3527208318
H	1.2839321969	2.2507216503	-1.9287562863

10-H

E(UPW6B95D3) = -793.820666855

E(UM06L) = -792.659887658

Charge = 1 Multiplicity = 2

C	-3.3540634029	0.2306919735	0.0748805786
C	-2.2229507927	1.0048577964	0.2649322514
C	-1.3212204459	1.1919930193	-0.7732401360
C	-1.5687971421	0.6019891822	-2.0039397041
C	-2.6964701374	-0.1779248659	-2.1925232261
C	-3.5920381665	-0.3648521201	-1.1528942220
H	-4.0504185290	0.0926417311	0.8876234319
H	-2.0438310207	1.4668433469	1.2245119316
H	-0.8759518868	0.7609069695	-2.8172616598
H	-2.8791967262	-0.6322390141	-3.1541483264
H	-4.4754742756	-0.9665985226	-1.3004738263

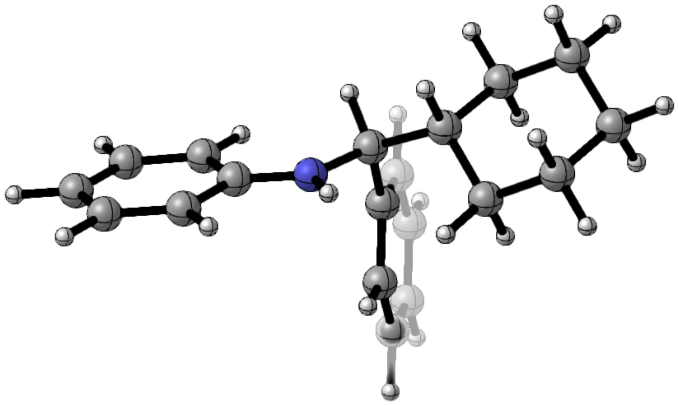

C	-0.0617050812	2.0083597824	-0.5916731882
N	-0.3076615744	3.1650072441	0.2578832082
C	-1.0111040812	4.2469424154	-0.0560631401
C	-1.1330126532	5.2743276301	0.9180000941
C	-1.6375375356	4.3925015814	-1.3201632139
C	-1.8540263198	6.3965395665	0.6318248728
H	-0.6489461685	5.1419191292	1.8735111480
C	-2.3527443672	5.5257903468	-1.5796692238
H	-1.5484092762	3.6158607344	-2.0604284993
C	-2.4678260551	6.5311401791	-0.6147162129
H	-1.9502597238	7.1787986759	1.3668638457
H	-2.8336270866	5.6477402339	-2.5364993338
H	-3.0364677379	7.4198252283	-0.8373385602
C	1.1330565526	1.2319464904	-0.0211212104
C	0.8585020736	0.5576855346	1.3161474183
C	1.6430515566	0.2087689673	-1.0259437349
H	1.9229172168	1.9737050755	0.1260105744
C	2.1184535951	-0.1127706327	1.8426180391
H	0.0817137285	-0.1957647958	1.1818433311
H	0.4829868659	1.2693635418	2.0527250414
C	2.8995710731	-0.4744845850	-0.5106412289
H	0.8688158687	-0.5412738587	-1.1955475327
H	1.8354576647	0.6924506600	-1.9832027355
C	2.6568210055	-1.1227963077	0.8424397423
H	1.9092597863	-0.5961439553	2.7953487660
H	2.8779106517	0.6483436765	2.0320288922
H	3.2393672183	-1.2159161992	-1.2318916630
H	3.6963855145	0.2660273527	-0.4175606534
H	3.5753489360	-1.5725358669	1.2163615601
H	1.9314714797	-1.9308008673	0.7267599634
H	0.2344602251	2.3886505088	-1.5679469680
H	0.1007031739	3.1472520173	1.1844715091

11

E(UPW6B95D3) = -2833.64514467

E(UM06L) = -2831.04770164

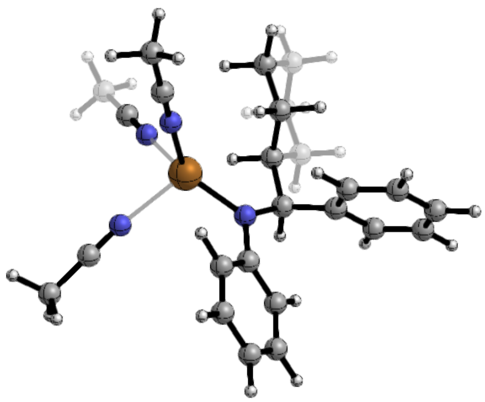

Charge = 1 Multiplicity = 2

C	-2.3006840456	1.4595849831	-0.0972308384
C	-0.9650021595	1.7981248174	0.0293353195

Go to table of contents

C	0.0014147278	1.1623330757	-0.7399295927
C	-0.3970807302	0.1860837887	-1.6417652007
C	-1.7326226947	-0.1578312302	-1.7684907577
C	-2.6891237270	0.4785982634	-0.9955852555
H	-3.0403842037	1.9631328635	0.5065462580
H	-0.6673313847	2.5655355186	0.7281081918
H	0.3470509340	-0.3015208683	-2.2550094796
H	-2.0260682654	-0.9179226292	-2.4764700851
H	-3.7312134584	0.2166739490	-1.0962799608
C	1.4692725475	1.4994177284	-0.5869921170
N	1.6653803272	2.9267575326	-0.3614330501
C	1.1936363456	3.7764624667	-1.3237079677
C	1.0197090086	5.1318962748	-0.9842424788
C	0.8696274512	3.3815988610	-2.6369828454
C	0.5562271037	6.0428036781	-1.9049412229
H	1.2278087149	5.4381376309	0.0296740666
C	0.4211806978	4.3058697458	-3.5541286968
H	0.9947631352	2.3574386076	-2.9442347417
C	0.2582366355	5.6383892746	-3.1993104466
H	0.4187561261	7.0728116384	-1.6142865562
H	0.1943066995	3.9860016969	-4.5594993667
H	-0.1026145209	6.3523137148	-3.9230053356
C	2.1544407417	0.6722179898	0.5087260119
C	1.4983164081	0.7814466083	1.8781046677
C	2.2903779811	-0.7915522419	0.1161889204
H	3.1639267102	1.0806662979	0.5871452492
C	2.2949433644	0.0230445983	2.9281220911
H	0.4929806671	0.3605383651	1.8252377791
H	1.3895045527	1.8280004605	2.1644317248
C	3.0989007133	-1.5541916260	1.1543541826
H	1.2984805001	-1.2399752599	0.0346039709
H	2.7575070895	-0.8718528185	-0.8657523514
C	2.4707755217	-1.4345321434	2.5336584988
H	1.7997720987	0.0964595341	3.8955591439
H	3.2765922473	0.4859653505	3.0398632754
H	3.1860272831	-2.6010204681	0.8667832970
H	4.1128075864	-1.1483144382	1.1854815456
H	3.0766218415	-1.9573171036	3.2727640168
H	1.4934568742	-1.9219578378	2.5227440391
H	1.9643578455	1.2275144070	-1.5239099723

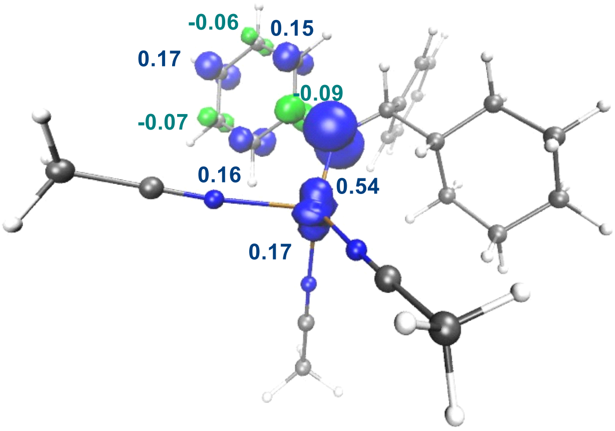

Cu	2.7834957755	3.6665950070	1.0580070966
N	4.0086309696	5.2455298803	0.1411805617
C	5.3381612235	7.0401883023	-1.1602128297
H	4.6457451831	7.7623024848	-1.5835833724
H	6.0246475756	7.5484053160	-0.4893244467
H	5.8997996249	6.5677990216	-1.9609054190
C	4.6014673882	6.0389577102	-0.4323445726
C	1.8787363686	5.0649793925	3.7539170237
C	1.4733790464	5.6787941781	4.9907592875
H	1.5851583170	4.9648606688	5.8019316926
H	2.0950544972	6.5478672390	5.1859360268
H	0.4338179026	5.9856371045	4.9199708269
N	2.2007980376	4.5758859819	2.7717411751
N	4.5496511750	2.7662167600	1.8346646284
C	6.6841297585	1.5945323633	2.6985823403
H	6.8266574728	1.8194772478	3.7515832553
H	6.5726905823	0.5211879388	2.5700597541
H	7.5473140658	1.9378173117	2.1356918275
C	5.4989977446	2.2563470347	2.2185772133

12

E(UPW6B95D3) = -2464.80142037

E(UM06L) = -2462.77989777

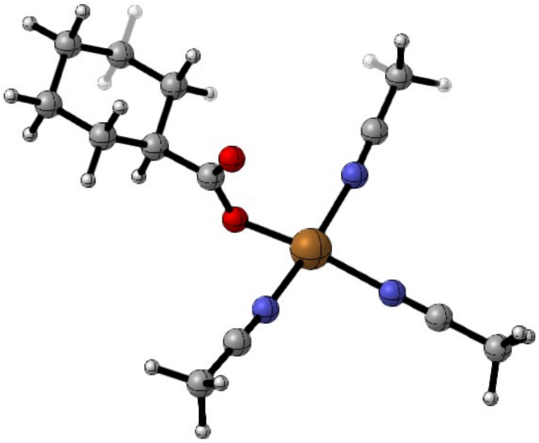

Charge = 1 Multiplicity = 2

Cu	2.9037974988	4.1063133521	1.0434026300
N	4.3194981055	4.2971059883	-0.3622854973
C	6.2268121622	4.6501548671	-2.0590715329
H	6.0401039454	5.5602255487	-2.6223133704
H	7.1692597497	4.7369299359	-1.5259506914
H	6.2674298970	3.8032093385	-2.7382669428
C	5.1635286341	4.4513101548	-1.1145637829
C	0.8848807849	4.6670956440	3.3922266556
C	0.0421896932	4.9461578528	4.5211494926
H	0.3970246082	4.3835316504	5.3801925047
H	0.0805206321	6.0092770653	4.7418214035
H	-0.9793339073	4.6577180484	4.2908331313
N	1.5591464734	4.4479173805	2.4981031740
O	4.2080227576	3.6213353261	2.3643187076
C	4.7319965606	4.6599888976	2.9193883902

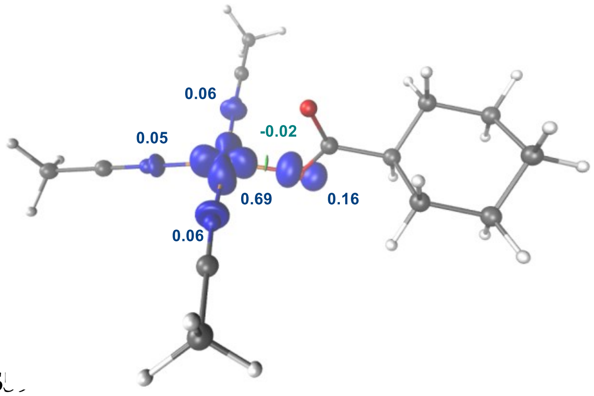

Go to table of contents

O	4.4760900899	5.8099128379	2.5759723754
C	5.6644550150	4.3647520475	4.0727090973
C	6.9254720967	5.2158682542	4.0171383017
C	4.9194181645	4.6041263721	5.3879022112
H	5.9384314140	3.3114938035	4.0218960825
C	7.8251483426	4.9354851545	5.2100118096
H	6.6326807799	6.2658762841	4.0129021296
H	7.4590403694	5.0293245063	3.0855681872
C	5.8175505895	4.3240951381	6.5814701009
H	4.5908344229	5.6444675488	5.4128999654
H	4.0252893189	3.9825762157	5.4273619540
C	7.0876737904	5.1571816445	6.5209022165
H	8.7120197974	5.5659833105	5.1631525353
H	8.1711928247	3.9006821487	5.1640188233
H	5.2772581178	4.5217276523	7.5063516308
H	6.0819754014	3.2647090330	6.5894408940
H	7.7351525662	4.9200101609	7.3640170602
H	6.8261231956	6.2138476847	6.6093849490
N	1.4431191066	4.0831835123	-0.3573124210
C	-0.4334095024	4.0806636457	-2.1239750009
H	-0.6670032132	5.1058891714	-2.3974055410
H	-0.1007321773	3.5355462261	-3.0027797767
H	-1.3165808306	3.6032350677	-1.7092874230
C	0.6127276340	4.0812575989	-1.1403237264

13

E(RPW6B95D3) = -2464.96147156

E(RM06L) = -2462.93602524

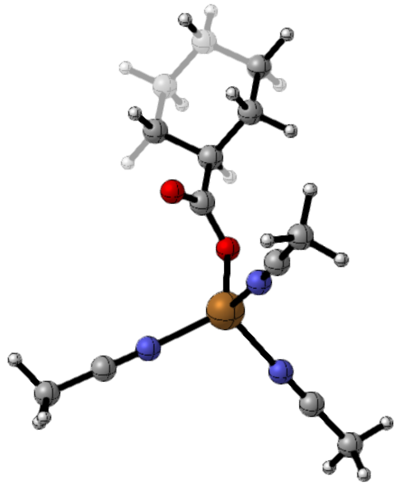

Charge = 0 Multiplicity = 1

Cu	2.9468609971	4.8439322009	0.9346779466
N	3.8691207345	5.8379602860	-0.5668708219
C	5.1437725646	7.1112599062	-2.4209419712
H	4.4706424007	7.8032235641	-2.9189944778
H	5.9675641671	7.6650114858	-1.9796335956
H	5.5342650438	6.4034564966	-3.1465928305
C	4.4323401042	6.4018112966	-1.3887397427
C	0.8726088678	6.7471931194	2.4157075617
C	-0.0141062839	7.6195251798	3.1425717346
H	0.2375545394	7.5956157595	4.1990520907

H	0.0889679816	8.6353057703	2.7716374053
H	-1.0407457468	7.2905171158	3.0096293167
N	1.5807139318	6.0548729849	1.8415138356
O	4.1767732327	3.9171448994	2.3004063405
C	4.8535404838	4.6578284415	3.0793682186
O	4.9041874891	5.8938359130	3.0369006005
C	5.6355835205	3.9314409702	4.1695535681
C	7.0767924391	4.4153875890	4.2592429163
C	4.9375113707	4.1206941172	5.5155458330
H	5.6329769402	2.8675921491	3.9300461988
C	7.8328378746	3.7161782257	5.3778624657
H	7.0632486072	5.4910287167	4.4378158536
H	7.5817757454	4.2588597216	3.3056289039
C	5.6868981876	3.4216148753	6.6381033949
H	4.8827666414	5.1908947918	5.7237537515
H	3.9129170076	3.7531521665	5.4566743078
C	7.1291419627	3.8965674649	6.7135517277
H	8.8536503679	4.0932030275	5.4343034686
H	7.9039927364	2.6494792095	5.1531132221
H	5.1816986094	3.5906864315	7.5886917327
H	5.6737077239	2.3437819591	6.4614978898
H	7.6633505376	3.3637391304	7.4995221567
H	7.1422122146	4.9550011179	6.9834485701
N	1.8825699149	3.2806555312	0.0981493266
C	0.5687818265	1.2866884884	-0.8936781898
H	-0.3218269948	1.6513453093	-1.3974811678
H	1.1943055735	0.7574231435	-1.6068798249
H	0.2791097805	0.6084241972	-0.0962479529
C	1.3007418142	2.3978333172	-0.3409090567

14

E(RPW6B95D3) = -558.168370985

E(RM06L) = -557.349825518

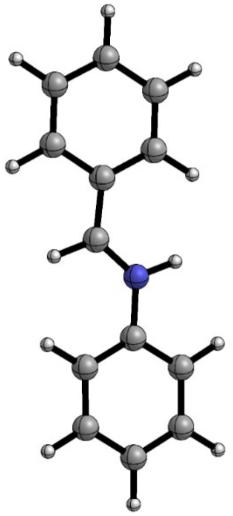

Charge = 1 Multiplicity = 1

C	-1.6863279772	0.1795027215	0.7048763965
C	-0.4530852050	0.7899743952	0.7923187110
C	-0.2144012830	1.9933434985	0.1224973507
C	-1.2336857469	2.5794742773	-0.6365604944
C	-2.4605107392	1.9639646841	-0.7178825106

C	-2.6880211020	0.7663198044	-0.0494740479
H	-1.8678612808	-0.7484391139	1.2227413098
H	0.3395436349	0.3477987290	1.3764510606
H	-1.0766334284	3.5111726877	-1.1578261475
H	-3.2483506956	2.4137484257	-1.3003681903
H	-3.6547977884	0.2925717032	-0.1192125685
C	1.0909152502	2.5549376828	0.2732463831
H	1.7888131247	2.0261116498	0.9034998505
N	1.5049460120	3.6424514241	-0.2827841133
C	2.7703502658	4.2574761065	-0.1614971812
C	2.9936213157	5.3685011975	-0.9594825611
C	3.7462635701	3.7940048707	0.7076507671
C	4.2110175569	6.0181974846	-0.8975366888
H	2.2137971496	5.7141594266	-1.6213135933
C	4.9583899897	4.4528612514	0.7583931945
H	3.5760413813	2.9416049759	1.3436991598
C	5.1966620843	5.5607276458	-0.0407971775
H	4.3860793852	6.8814143580	-1.5195061930
H	5.7218069891	4.0982545585	1.4324516228
H	6.1475987285	6.0669264735	0.0094858553
H	0.8681961307	4.1386776631	-0.8964282015

15

E(RPW6B95D3) = -2597.99433093

E(RM06L) = -2595.73210614

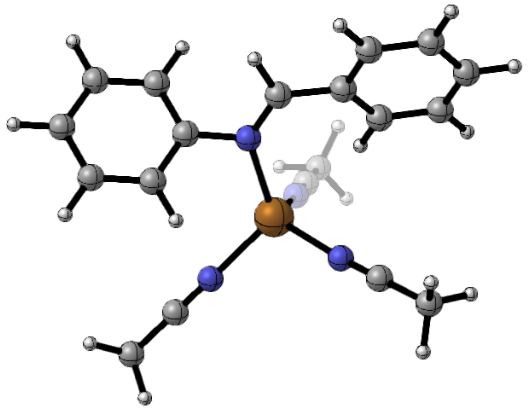

Charge = 1   Multiplicity = 1

C	-1.3063185872	4.2725562884	-2.6726916665
C	-1.6059356240	5.4883918659	-2.0761768740
C	-2.4336557852	5.5301110510	-0.9664172387
C	-2.9520698012	4.3571828874	-0.4529409685
C	-2.6294868786	3.1275809216	-1.0256789144
C	-1.8118486828	3.0976338493	-2.1529953178
C	-3.1949014225	1.9430908748	-0.3993785705
N	-2.7600118031	0.7479496785	-0.5035994732
Cu	-1.0842654295	-0.0718338419	-1.4963564374
C	-3.5076856543	-0.2567176470	0.1571034734
C	-4.8746518544	-0.3833396918	-0.0532169120
C	-5.5812963634	-1.3858197434	0.5891546915
C	-4.9337023167	-2.2569727207	1.4480518583

C	-3.5692816686	-2.1252742099	1.6588337617
C	-2.8544638358	-1.1374519501	1.0090361625
N	0.5729890516	1.0455413943	-1.1180180582
C	1.4500864955	1.7386009595	-0.8737166722
C	2.5460186692	2.6211225740	-0.5693749606
C	-2.2065655878	-0.4482743986	-6.0031873577
C	-1.8398166866	-0.3199753205	-4.6167502836
N	-1.5487126956	-0.2139896783	-3.5149830373
N	-0.6085671124	-2.0355736447	-1.1217549769
C	-0.3608276484	-3.1418436521	-0.9663178385
C	-0.0556003123	-4.5344669397	-0.7643996046
H	-0.6795798571	4.2426363849	-3.5505293122
H	-1.2045342669	6.4027673365	-2.4851664215
H	-2.6791449671	6.4746717046	-0.5068065676
H	-3.6061049008	4.3817351683	0.4062279083
H	-1.5899924296	2.1609900670	-2.6347720837
H	-4.0531950821	2.1337361716	0.2422350854
H	-5.3708012720	0.2890516256	-0.7364529612
H	-6.6411154574	-1.4864649921	0.4121138500
H	-5.4861830982	-3.0380100406	1.9467023224
H	-3.0581250494	-2.7981430339	2.3301752519
H	-1.7918059071	-1.0302137540	1.1603177266
H	3.4277108988	2.0374377129	-0.3207906574
H	2.7581147236	3.2457722364	-1.4325364489
H	2.2801423407	3.2509473388	0.2748989009
H	-2.7521720568	-1.3758231201	-6.1512370220
H	-2.8349588592	0.3898226970	-6.2908684810
H	-1.3107162256	-0.4548606090	-6.6174181339
H	0.9995309149	-4.6465855827	-0.5318614510
H	-0.6497764080	-4.9192988785	0.0597701192
H	-0.2873748019	-5.0923472412	-1.6672480417

4a

E(RPW6B95D3) = -794.007962980

E(RM06L) = -792.842152861

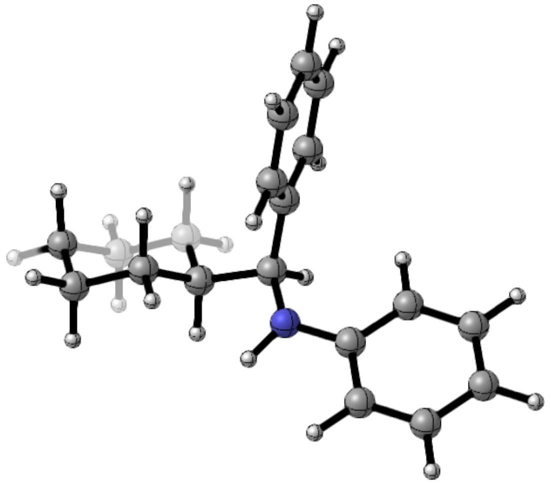

Charge = 0 Multiplicity = 1

C	-3.3643277888	0.1896690979	0.0032922531
C	-2.2546304760	0.9869407412	0.2277811364
C	-1.3159549754	1.1906419420	-0.7735507200

C	-1.5167197359	0.5896756898	-2.0089121820
C	-2.6231679035	-0.2104177514	-2.2376135730
C	-3.5514630762	-0.4139879831	-1.2295844268
H	-4.0866654551	0.0418820463	0.7919368181
H	-2.1138553915	1.4653962231	1.1852525085
H	-0.8002623273	0.7595496993	-2.8001863296
H	-2.7649110909	-0.6681026899	-3.2049830068
H	-4.4188432198	-1.0314300358	-1.4064303085
C	-0.0835798736	2.0378099070	-0.5398810797
N	-0.3407681535	3.1297472729	0.3686972882
C	-1.0363067578	4.2544810810	0.0143652398
C	-1.0485745253	5.3575474103	0.8788950616
C	-1.7592596887	4.3444235890	-1.1791844393
C	-1.7568727184	6.4963988697	0.5615546849
H	-0.4899006734	5.3021557922	1.8024954657
C	-2.4681090157	5.4954932534	-1.4821076780
H	-1.7716549819	3.5163586137	-1.8686467191
C	-2.4781468848	6.5798665945	-0.6229264337
H	-1.7453825724	7.3311391066	1.2467549792
H	-3.0193486552	5.5380962748	-2.4099278589
H	-3.0332166685	7.4717640865	-0.8672670324
C	1.1106396809	1.2289731097	-0.0062200867
C	0.8447631283	0.5428976375	1.3279887426
C	1.6188809449	0.2114546689	-1.0163153703
H	1.9080875045	1.9623041370	0.1488150937
C	2.1034021836	-0.1359574339	1.8464491895
H	0.0644967848	-0.2076560196	1.1915518173
H	0.4717990127	1.2552310556	2.0633602437
C	2.8810351933	-0.4708635937	-0.5122086745
H	0.8491574598	-0.5435587275	-1.1849037179
H	1.8029199966	0.6975763979	-1.9744660686
C	2.6440995927	-1.1338685131	0.8351990491
H	1.8966542520	-0.6312228553	2.7941678387
H	2.8636930058	0.6224944624	2.0449893558
H	3.2253143566	-1.2040910831	-1.2402261981
H	3.6745484334	0.2727261997	-0.4123367796
H	3.5645904246	-1.5856717710	1.2026434226
H	1.9208830014	-1.9429624112	0.7119472914
H	0.2195873975	2.4346178845	-1.5143727166
H	0.3198871062	3.2472482139	1.1160131405

HA

E(UPW6B95D3) = -1248.55343885

E(UM06L) = -1247.02164538

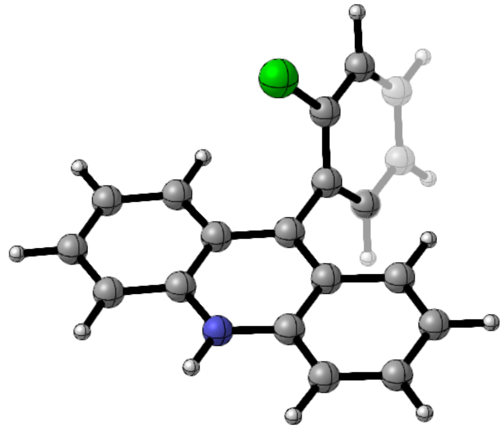

	Charge = 0	Multiplicity = 2	
C	-4.4316167967	-1.2280411041	0.1058875838
C	-3.0443969755	-1.2012160362	0.0369868141
C	-2.3382696759	0.0250783153	0.0424775019
C	-3.1009908511	1.2044555509	0.1014645520
C	-4.4757712470	1.1710884296	0.1681286433
C	-5.1464139308	-0.0479881129	0.1739236362
H	-4.9360880738	-2.1831830429	0.1022342459
H	-2.5892435151	2.1539138313	0.0922246001
H	-5.0325362511	2.0941424103	0.2139160242
H	-6.2234522311	-0.0770548360	0.2271541168
C	-0.9201047506	0.0091498111	-0.0265796987
C	-0.9709599700	-2.4337310224	-0.1274281556
C	-0.2288375803	-1.2277193818	-0.1171548091
C	-0.3337346344	-3.6647919579	-0.2149450768
C	1.0442540699	-3.7312688368	-0.2848860433
H	1.5309204327	-4.6915415942	-0.3530623606
C	1.7968554594	-2.5616872718	-0.2612599548
H	2.8737496977	-2.6111694589	-0.3060380641
C	1.1723221067	-1.3373662099	-0.1767885599
H	1.7642394190	-0.4366063147	-0.1522828336
N	-2.3342712511	-2.3672996257	-0.0438146265
H	-2.8485628660	-3.2329116882	-0.0510441695
C	-0.1646518113	1.2746781123	-0.0475544492
C	-0.0421788118	2.1010593364	1.0658544959
C	0.4705103269	1.6915809040	-1.2159882344
C	0.6591308685	3.2925416915	1.0259219038
C	1.1783154954	2.8764477746	-1.2745714555
H	0.3948843003	1.0640659541	-2.0911115653
C	1.2698069017	3.6814334843	-0.1513480815
H	0.7287514287	3.9021313211	1.9122495173
H	1.6546416397	3.1725850882	-2.1960497436
H	1.8183604480	4.6096244537	-0.1851415741
Cl	-0.7646838419	1.6281669095	2.5740347057
H	-0.9334768417	-4.5629591391	-0.2250355462

A

E(RPW6B95D3) = -1247.95790632

E(RM06L) = -1246.42602246

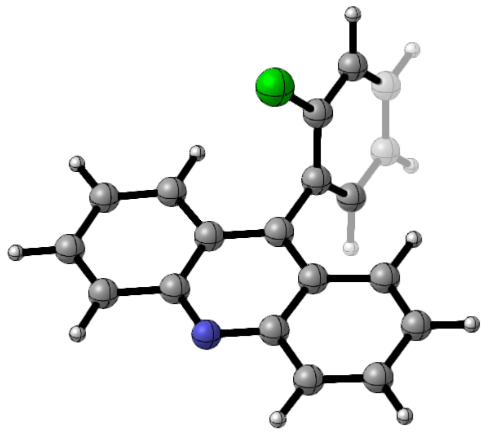

	Charge = 0	Multiplicity = 1	
C	-4.4223305096	-1.2388980687	0.0635864388
C	-3.0041214315	-1.2316999433	0.0043056749
C	-2.3236240654	0.0250940315	0.0098257032
C	-3.0903338559	1.2177303137	0.0680311225
C	-4.4459190482	1.1686551099	0.1250950180
C	-5.1197855884	-0.0759541040	0.1238190344
H	-4.9195040251	-2.1965583994	0.0588310683
H	-2.5795952579	2.1678997823	0.0650269067
H	-5.0189576792	2.0815529048	0.1696467481
H	-6.1978146784	-0.0934835241	0.1692851429
C	-0.9285313373	0.0213665927	-0.0447367803
C	-1.0363066122	-2.4003388567	-0.1087830043
C	-0.2568238673	-1.2009383488	-0.1028593262
C	-0.3644903238	-3.6494567976	-0.1694559825
C	0.9908632316	-3.7069726590	-0.2124041785
H	1.4906618021	-4.6623088026	-0.2571410698
C	1.7627424812	-2.5210242334	-0.1936814926
H	2.8390949199	-2.5888049840	-0.2196549113
C	1.1587196675	-1.3062701356	-0.1406412721
H	1.7499845401	-0.4047285617	-0.1226404836
N	-2.3668721136	-2.3999906339	-0.0561566776
C	-0.1722021737	1.2933951252	-0.0643870887
C	0.0130932847	2.0655557904	1.0753615751
C	0.3822046940	1.7522543990	-1.2543104028
C	0.7186264972	3.2541415389	1.0430017283
C	1.0866221470	2.9399809579	-1.3042943726
H	0.2491259914	1.1619056556	-2.1478271094
C	1.2542984483	3.6914684249	-0.1538122362
H	0.8461464145	3.8256243010	1.9479139851
H	1.5038170279	3.2771365401	-2.2399068758
H	1.8041933262	4.6189623073	-0.1818645712
Cl	-0.6391032291	1.5323886377	2.5938011693
H	-0.9688704507	-4.5433499939	-0.1772933358

HA⁺

E(UPW6B95D3) = -1248.41967033

E(RM06L) = -1246.89103525

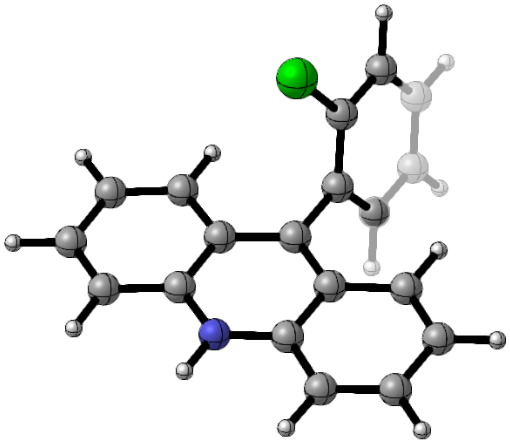

	Charge = 1	Multiplicity = 1	
C	-4.4277023678	-1.2398803970	0.0877830797
C	-3.0270595859	-1.2082857450	0.0219575475
C	-2.3237973136	0.0225622322	0.0279852441
C	-3.0804373277	1.2178946905	0.0874116286
C	-4.4367031516	1.1733231813	0.1514860339
C	-5.1126623838	-0.0649360542	0.1544266406
H	-4.9325063728	-2.1932913361	0.0834219900
H	-2.5638251752	2.1633216164	0.0753299126
H	-5.0065210507	2.0871600751	0.1961779796
H	-6.1898393519	-0.0793410283	0.2056696238
C	-0.9246979121	0.0050461875	-0.0347003002
C	-0.9870494541	-2.4199163890	-0.1211369426
C	-0.2401063747	-1.2141267795	-0.1076101989
C	-0.3457986840	-3.6648040773	-0.1995099712
C	1.0141787615	-3.7060617099	-0.2532366593
H	1.5151602225	-4.6592296439	-0.3128534838
C	1.7804538976	-2.5223974778	-0.2248451749
H	2.8558713917	-2.5875753465	-0.2572714943
C	1.1728455743	-1.3093643793	-0.1531868964
H	1.7563904461	-0.4045319161	-0.1259637149
N	-2.3277856946	-2.3539806469	-0.0533281763
H	-2.8440776874	-3.2251240575	-0.0618366742
C	-0.1667051616	1.2734425957	-0.0553975843
C	-0.0085785534	2.0589069578	1.0791328555
C	0.4141729151	1.7047145998	-1.2425005513
C	0.7008786686	3.2440768553	1.0411138672
C	1.1180131234	2.8920259692	-1.2945679065
H	0.3010479326	1.0979497857	-2.1274386055
C	1.2606187228	3.6606250965	-0.1521949489
H	0.8134839752	3.8286963739	1.9394314487
H	1.5563258478	3.2139192694	-2.2255992750
H	1.8127140281	4.5865531652	-0.1833404352
Cl	-0.6875280916	1.5416094650	2.5889360144
H	-0.9422778126	-4.5633741323	-0.2133698727

TS1

E(UPW6B95D3) = -793.316671142

E(UM06L) = -792.158874675

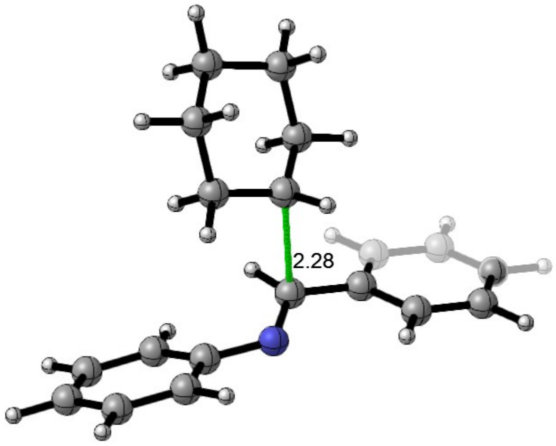

	Charge = 0	Multiplicity = 2	
C	-1.6548108251	0.2075919823	0.0702238756
C	-0.4204291779	0.7757328129	0.3326649208
C	-0.2171416107	2.1402759699	0.1577731172
C	-1.2765459134	2.9299522670	-0.2811651164
C	-2.5104287498	2.3639798242	-0.5354328875
C	-2.7043403282	1.0005079988	-0.3621165365
H	-1.7973614350	-0.8537340965	0.2048193342
H	0.3994776076	0.1604345244	0.6736556819
H	-1.1196261791	3.9886327505	-0.4176344263
H	-3.3268408495	2.9855969481	-0.8705560223
H	-3.6690170395	0.5607310123	-0.5637733854
C	1.1105166067	2.7078702209	0.4249783711
H	1.8465327204	2.0019464634	0.8074236575
N	1.4592837006	3.8357356354	-0.1204734548
C	2.7866440841	4.2331807679	-0.1221721359
C	3.0473555617	5.6034112655	-0.2148094032
C	3.8755638885	3.3540904584	-0.0841372600
C	4.3423503915	6.0819070757	-0.2253100622
H	2.2085678949	6.2814893017	-0.2622575336
C	5.1691467681	3.8390681323	-0.1069146585
H	3.7079569513	2.2886956703	-0.0622396340
C	5.4135224609	5.2028617746	-0.1674978128
H	4.5183737739	7.1454337164	-0.2834295820
H	5.9959020947	3.1448413358	-0.0849738882
H	6.4263142885	5.5742650187	-0.1848982543
C	0.4559896200	1.9285123396	3.2942427618
C	0.4635473202	2.1422937309	4.8159980946
C	1.7608908172	2.7890738901	5.2689719685
C	2.0018439024	4.1007213481	4.5418045695
C	2.0001847116	3.8896355793	3.0203940591
C	0.7417363726	3.2164846640	2.6128236393
H	1.7404005264	2.9552326965	6.3451955119
H	-0.3758907953	2.7829337063	5.0894465931
H	0.3122628455	1.1853751402	5.3136990210
H	1.2337976323	1.2008869113	3.0481184300

H	-0.4971389739	1.5131494278	2.9802133571
H	1.2170015669	4.8111752185	4.8050766548
H	2.9501496459	4.5433821951	4.8431597679
H	2.1273147973	4.8388999907	2.5079273231
H	2.8540604386	3.2578502444	2.7632293710
H	-0.1053852929	3.8535893406	2.3936231571
H	2.5912441801	2.1090657458	5.0677088156

TS2

E(UPW6B95D3) = -793.783111322

E(UM06L) = -792.627108644

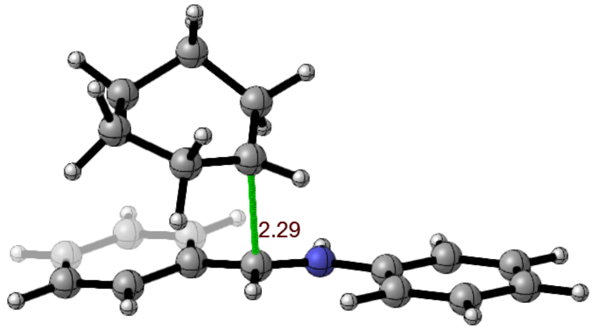

	Charge = 1	Multiplicity = 2	
C	-1.7881853312	1.2273677443	1.8347925235
C	-0.5397393548	1.4038746673	1.2754876595
C	0.0536775224	2.6673721523	1.2553264609
C	-0.6353179886	3.7531644776	1.8011142545
C	-1.8845444833	3.5699559700	2.3561447644
C	-2.4619713569	2.3089701207	2.3793014966
H	-2.2370082096	0.2467599275	1.8466948258
H	-0.0083534802	0.5664970454	0.8493474515
H	-0.2035766280	4.7423799309	1.8114112943
H	-2.4086179384	4.4122083504	2.7793526962
H	-3.4368726873	2.1716825086	2.8203822940
C	1.3727742008	2.7785411607	0.6795316795
H	1.7854694548	1.8987756192	0.2149807510
N	1.8819709418	3.9482105187	0.3229614928
C	3.0847353582	4.1816472916	-0.3471598518
C	3.4689514389	5.5065615361	-0.5261191864
C	3.8841285685	3.1502863657	-0.8264023580
C	4.6470787158	5.7969506119	-1.1826781683
H	2.8391599510	6.2982048344	-0.1478068854
C	5.0620341979	3.4566305177	-1.4803574045
H	3.5999257312	2.1188590544	-0.6987018273
C	5.4497848169	4.7740683189	-1.6620669271
H	4.9390744535	6.8263384924	-1.3185102624
H	5.6805184281	2.6545759543	-1.8514268963
H	6.3708722847	5.0017493989	-2.1747677335
C	2.4883403595	3.2903012643	3.4696986290
C	1.9550130041	2.7703357672	4.7978016045

C	0.8442127583	1.7644021060	4.5626606600
C	1.3861432046	0.5134769441	3.8865055612
C	2.3982062554	0.8019646390	2.7681564083
C	2.6869048674	2.2184949415	2.4645010280
H	0.3701966524	1.4894071753	5.5024884920
H	2.7629685619	2.2937418083	5.3540290700
H	1.6051347456	3.6074496216	5.3968925974
H	1.7979729282	4.0434312633	3.0758187954
H	3.4320580084	3.8224452847	3.5993069685
H	1.8752429470	-0.1024157997	4.6386767038
H	0.5642317023	-0.0779945177	3.4920086326
H	3.3730739980	0.3758036842	3.0372591833
H	2.1370679358	0.2798521693	1.8469058097
H	3.4788783596	2.3738099856	1.7451923817
H	0.0725666510	2.2242055959	3.9460305689
H	1.3945064542	4.7829914971	0.6147217623

TS3

E(UPW6B95D3) = -2833.60793761

E(UM06L) = -2831.01007754

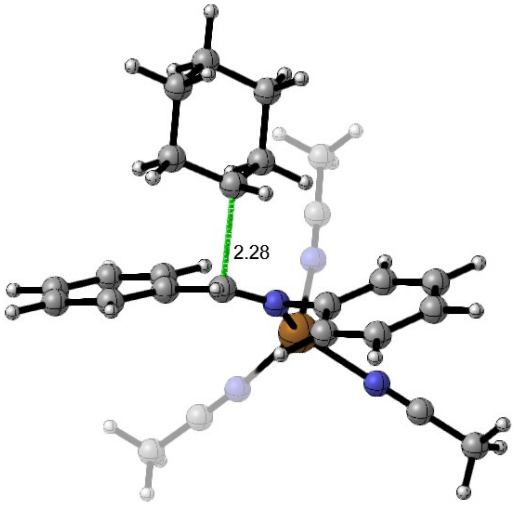

Charge = 1 Multiplicity = 2

C	3.9935392749	2.3316884472	-3.6902013886
C	3.0733732232	2.6513363927	-2.7096524629
C	2.2398021560	1.6705305462	-2.1805001133
C	2.3324823002	0.3713668987	-2.6743843371
C	3.2597651450	0.0499615242	-3.6478690027
C	4.0955526623	1.0304578908	-4.1574683394
H	4.6323350151	3.1020050338	-4.0945992245
H	2.9921978480	3.6672843539	-2.3600147568
H	1.6725689622	-0.3886539571	-2.2822922475
H	3.3274251663	-0.9635543386	-4.0120001973
H	4.8163312325	0.7846585258	-4.9220480205
C	1.2275568033	1.9438164283	-1.1614696642
N	1.3534141782	2.8676598818	-0.2443646743
C	0.3476472243	2.9277221888	0.7355932301
C	-0.1888728228	1.7903799683	1.3360365492
C	-0.1113491970	4.1812086463	1.1334450100
C	-1.1690156707	1.9115084160	2.3046180341
H	0.1846685388	0.8145024252	1.0666919463

C	-1.1004123421	4.2949210199	2.0909671109
H	0.3220920287	5.0586664026	0.6773743178
C	-1.6346956541	3.1604362237	2.6824372948
H	-1.5650446386	1.0230396898	2.7724699571
H	-1.4519921333	5.2734989267	2.3805076666
H	-2.3996142436	3.2496593817	3.4380733085
C	-0.4297041997	2.8094504819	-2.4667937017
C	-0.0721944819	4.2308562091	-2.6788271289
C	-0.5330954581	1.9872682465	-3.6949845348
H	-1.1431748989	2.6034782139	-1.6779957108
C	-1.1156005113	4.8700003653	-3.6089474364
H	0.9032457260	4.2970947589	-3.1652025887
H	-0.0146775050	4.7729392660	-1.7389985395
C	-1.5886569181	2.6192414984	-4.6185863115
H	0.4215527885	1.9921686170	-4.2243696357
H	-0.7878778880	0.9538127767	-3.4708743407
C	-1.2507578422	4.0747393110	-4.8969515866
H	-0.8298064102	5.9001479645	-3.8180115640
H	-2.0781993416	4.9010900280	-3.0970606624
H	-1.6424249314	2.0503051458	-5.5456034315
H	-2.5680026896	2.5553609040	-4.1430781496
H	-2.0142142681	4.5205111596	-5.5330175673
H	-0.3094817435	4.1232316829	-5.4482301899
H	0.5122119395	1.1418335250	-1.0098630615
Cu	2.9273023495	4.1732388292	0.1568559424
N	2.6996473333	5.8676864956	-0.9717872605
C	2.0280421616	7.6979761541	-2.6680901205
H	2.7421956550	8.5161160343	-2.6421003252
H	2.0100742521	7.2665951344	-3.6652424851
H	1.0395476834	8.0742315824	-2.4200210313
C	2.4101498214	6.6865940023	-1.7174407220
C	2.7155515596	4.8668619364	3.2788119084
C	2.5363954247	5.1157975907	4.6856323054
H	1.4742914405	5.1597786637	4.9110785885
H	2.9934753270	4.3141161689	5.2585299299
H	3.0019436860	6.0603228528	4.9521589731
N	2.8438248090	4.6660311068	2.1595548166
N	4.7893208695	3.3797720228	-0.0954979657
C	7.0105955734	2.1863228976	-0.6592691650
H	7.8367005965	2.8889918438	-0.5982989485

H	7.1782798513	1.3708442882	0.0382944604
H	6.9476825368	1.7896457729	-1.6691578222
C	5.7777366462	2.8552095521	-0.3349239336

TsO$^-$

E(RPW6B95D3) = -896.143503551

E(RM06L) = -895.134743536

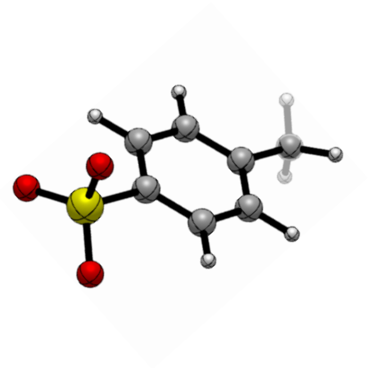

Charge = -1 Multiplicity = 1

C	-0.3922939448	0.7337995954	-0.1405138736
C	0.9890706195	0.7543427365	-0.0762706533
C	1.6596113066	1.9566526072	0.0824864476
C	0.9353863508	3.1329110779	0.1792824369
C	-0.4468044242	3.1041032024	0.1144157117
C	-1.1338447341	1.9064698636	-0.0447574797
H	-0.9059279667	-0.2088421759	-0.2649444683
H	1.5533639592	-0.1628882156	-0.1423018138
H	1.4575688846	4.0676065244	0.3118301456
H	-1.0032971605	4.0272644441	0.1907437314
S	3.4405107633	1.9920476295	0.0989145287
C	-2.6291405800	1.8744005848	-0.0803154557
H	-3.0354606890	2.8194985796	-0.4303264204
H	-2.9913805809	1.0801024857	-0.7279362045
H	-3.0357285934	1.6922352838	0.9144824804
O	3.8432769505	0.7302247877	0.7009877002
O	3.7962929041	3.1588310498	0.8918235791
O	3.8260179350	2.1092669389	-1.3012123922

TsOH

E(RPW6B95D3) = -896.589700974

E(RM06L) = -895.584096480

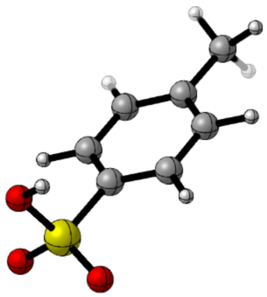

Charge = 0 Multiplicity = 1

C	-0.3956036135	0.7226967162	-0.1212523724
C	0.9830576341	0.7322566547	-0.0452989063
C	1.6384994689	1.9427521608	0.1078758116
C	0.9350203869	3.1343523685	0.1870719884
C	-0.4420230568	3.1041350361	0.1097571134
C	-1.1277040553	1.9025067379	-0.0436474963
H	-0.9133714124	-0.2168716310	-0.2413116995
H	1.5438621704	-0.1867380352	-0.0989372610

H	1.4592932195	4.0680748947	0.3119305792
H	-0.9972044799	4.0279307682	0.1704904012
S	3.3840788318	1.9736132456	0.1833128981
C	-2.6198518017	1.8783223966	-0.0942286492
H	-3.0138626317	2.7913890096	-0.5313875782
H	-2.9803821036	1.0284471440	-0.6664985804
H	-3.0302089117	1.7941158263	0.9119346279
O	3.8613572348	0.6724166032	0.5324393012
O	3.8123668595	3.1207747599	0.9126665677
O	3.8298980965	2.2778515646	-1.3085289878
H	3.7527126743	1.4781921190	-1.8538918577

X-Ray crystallogrophic data

N-(Cyclohexyl(phenyl)methyl)-4-methylaniline (4b)

CCDC 2340500

Bond precision: C-C = 0.0015 A Wavelength=1.54184

Cell: a=5.64642(5) b=20.42415(18) c=13.59672(11)

alpha=90 beta=92.2646(7) gamma=90

Temperature: 100 K

	Calculated	Reported
Volume	1566.79(2)	1566.79(2)
Space group	P 21/n	P 1 21/n 1
Hall group	-P 2yn	-P 2yn
Moiety formula	C20 H25 N	C20 H25 N
Sum formula	C20 H25 N	C20 H25 N
Mr	279.41	279.41
Dx,g cm-3	1.184	1.185
Z	4	4
Mu (mm-1)	0.507	0.507
F000	608.0	608.0
F000'	609.48	
h,k,lmax	7,25,17	7,25,17
Nref	3296	3256
Tmin,Tmax	0.928,0.978	0.465,1.000
Tmin'	0.881	

Correction method= # Reported T Limits: Tmin=0.465 Tmax=1.000 AbsCorr = GAUSSIAN

Data completeness= 0.988 Theta(max)= 76.664

R(reflections)= 0.0389(3003) wR2(reflections)= 0.1021(3256)

S = 1.041 Npar= 192

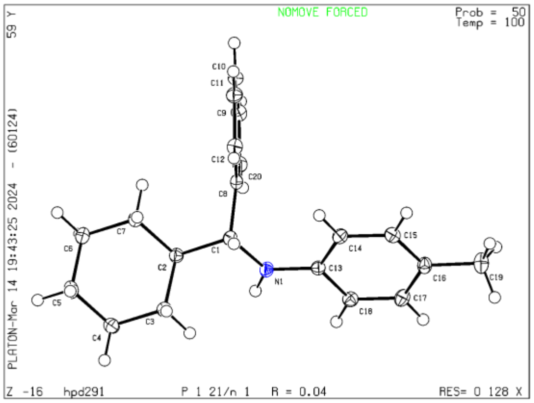

Go to table of contents

N-(Cyclohexyl(pyridin-4-yl)methyl)aniline (5l)
CCDC 2340502

Bond precision:	C-C = 0.0024 A		Wavelength=1.54184
Cell:	a=9.2584(2)	b=9.9261(2)	c=11.8278(3)
	alpha=93.448(2)	beta=90.414(2)	gamma=96.639(2)
Temperature:	100 K		

	Calculated	Reported
Volume	1077.62(4)	1077.62(4)
Space group	P -1	P -1
Hall group	-P 1	-P 1
Moiety formula	C18 H22 N2, C7 H12 O2	C7 H12 O2, C18 H22 N2
Sum formula	C25 H34 N2 O2	C25 H34 N2 O2
Mr	394.54	394.54
Dx,g cm-3	1.216	1.216
Z	2	2
Mu (mm-1)	0.598	0.598
F000	428.0	428.0
F000'	429.16	
h,k,lmax	11,12,14	11,12,14
Nref	4534	4297
Tmin,Tmax	0.934,0.958	0.667,1.000
Tmin'	0.909	

Correction method= # Reported T Limits: Tmin=0.667 Tmax=1.000 AbsCorr = GAUSSIAN

Data completeness= 0.948 Theta(max)= 76.849

R(reflections)= 0.0521(3797) wR2(reflections)= 0.1297(4297)

S = 1.022 Npar= 296

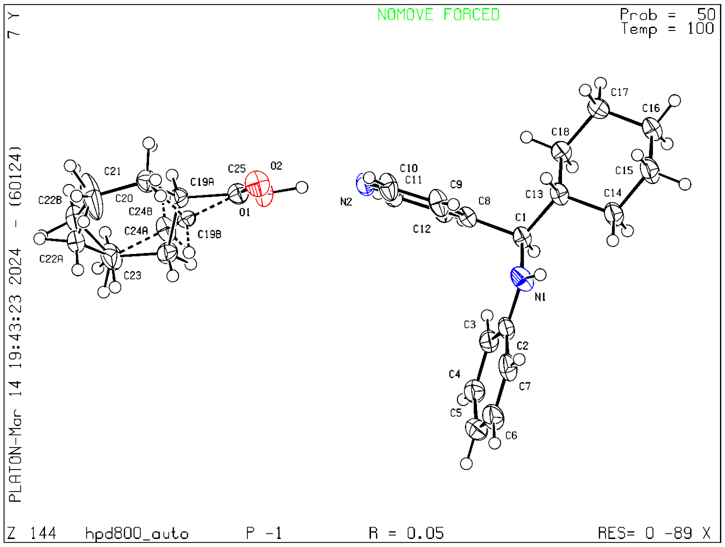

Go to table of contents

N-(Benzofuran-2-yl(cyclohexyl)methyl)aniline (5n)

CCDC 2340501

Bond precision:		C-C = 0.0019 A		Wavelength=1.54184
Cell:	a=5.71447(11)	b=17.9506(3)	c=16.3618(3)	
	alpha=90	beta=99.380(2)	gamma=90	
Temperature:	100 K			

	Calculated	Reported
Volume	1655.92(5)	1655.93(6)
Space group	P 21/n	P 1 21/n 1
Hall group	-P 2yn	-P 2yn
Moiety formula	C21 H23 N O	C21 H23 N O
Sum formula	C21 H23 N O	C21 H23 N O
Mr	305.40	305.40
Dx,g cm-3	1.225	1.225
Z	4	4
Mu (mm-1)	0.575	0.575
F000	656.0	656.0
F000'	657.75	
h,k,lmax	7,22,20	7,22,20
Nref	3495	3206
Tmin,Tmax	0.917,0.960	0.536,1.000
Tmin'	0.901	

Correction method= # Reported T Limits: Tmin=0.536 Tmax=1.000 AbsCorr = GAUSSIAN

Data completeness= 0.917 Theta(max)= 76.736

R(reflections)= 0.0409(2778) wR2(reflections)= 0.1053(3206)

S = 1.051 Npar= 208

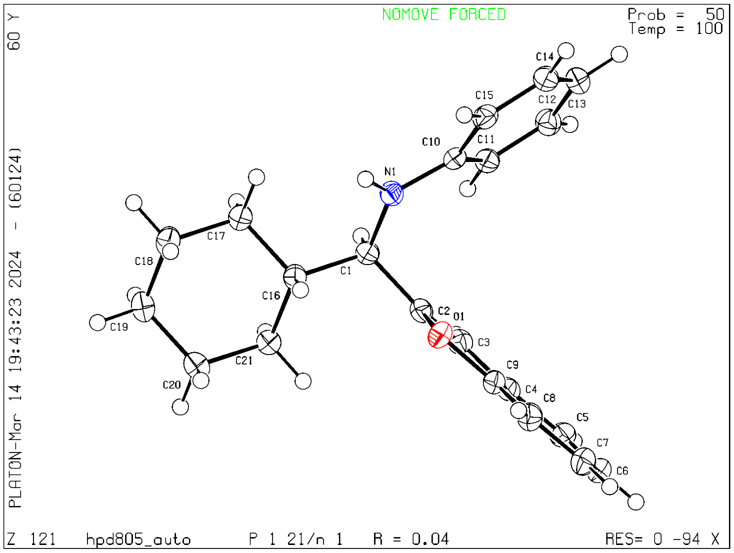

N-(Phenyl(tetrahydro-2H-pyran-4-yl)methyl)aniline (6h)

CCDC 2340499

Bond precision:	C-C = 0.0015 A		Wavelength=1.54184
Cell:	a=9.75307(9)	b=16.23315(13)	c=18.76027(18)
	alpha=90	beta=90	gamma=90
Temperature:	100 K		

	Calculated	Reported
Volume	2970.18(5)	2970.18(5)
Space group	P b c a	P b c a
Hall group	-P 2ac 2ab	-P 2ac 2ab
Moiety formula	C18 H21 N O	C18 H21 N O
Sum formula	C18 H21 N O	C18 H21 N O
Mr	267.36	267.36
Dx,g cm-3	1.196	1.196
Z	8	8
Mu (mm-1)	0.568	0.568
F000	1152.0	1152.0
F000'	1155.08	
h,k,lmax	12,20,23	12,19,23
Nref	3107	3000
Tmin,Tmax	0.949,0.959	0.547,1.000
Tmin'	0.887	

Correction method= # Reported T Limits: Tmin=0.547 Tmax=1.000 AbsCorr = GAUSSIAN

Data completeness= 0.966 Theta(max)= 76.115

R(reflections)= 0.0348(2725) wR2(reflections)= 0.0928(3000)

S = 1.019 Npar= 185

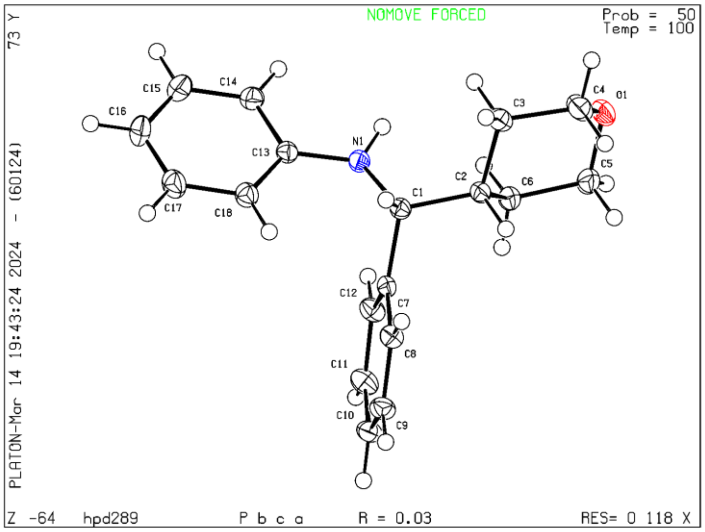

Ethyl 2-((4-methoxyphenyl)amino)-2-(1-phenylcyclohexyl)acetate (7w)

CCDC 2340503

Bond precision:	C-C = 0.0044 A		Wavelength=1.54184
Cell:	a=8.8935(2)	b=14.9861(4)	c=16.6162(3)
	alpha=112.739(2)	beta=90.306(2)	gamma=103.676(2)
Temperature:	100 K		

	Calculated	Reported
Volume	1972.96(9)	1972.96(8)
Space group	P -1	P -1
Hall group	-P 1	-P 1
Moiety formula	C23 H29 N O3	C23 H29 N O3
Sum formula	C23 H29 N O3	C23 H29 N O3
Mr	367.47	367.47
Dx,g cm-3	1.237	1.237
Z	4	4
Mu (mm-1)	0.643	0.643
F000	792.0	792.0
F000'	794.27	
h,k,lmax	11,18,21	11,18,20
Nref	8377	8018
Tmin,Tmax	0.916,0.944	0.648,1.000
Tmin'	0.873	

Correction method= # Reported T Limits: Tmin=0.648 Tmax=1.000 AbsCorr = GAUSSIAN

Data completeness= 0.957 Theta(max)= 77.326

R(reflections)= 0.0783(6789) wR2(reflections)= 0.1732(8018)

S = 1.073 Npar= 497

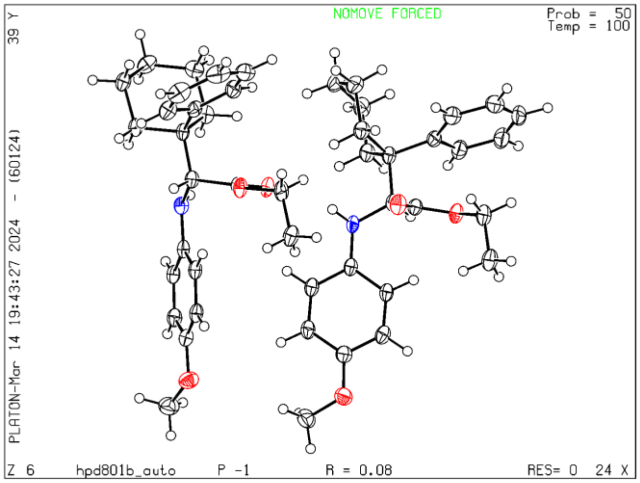

Go to table of contents

NMR Spectroscopic data

***N*-(Cyclohexyl(phenyl)methyl)aniline (4a)**

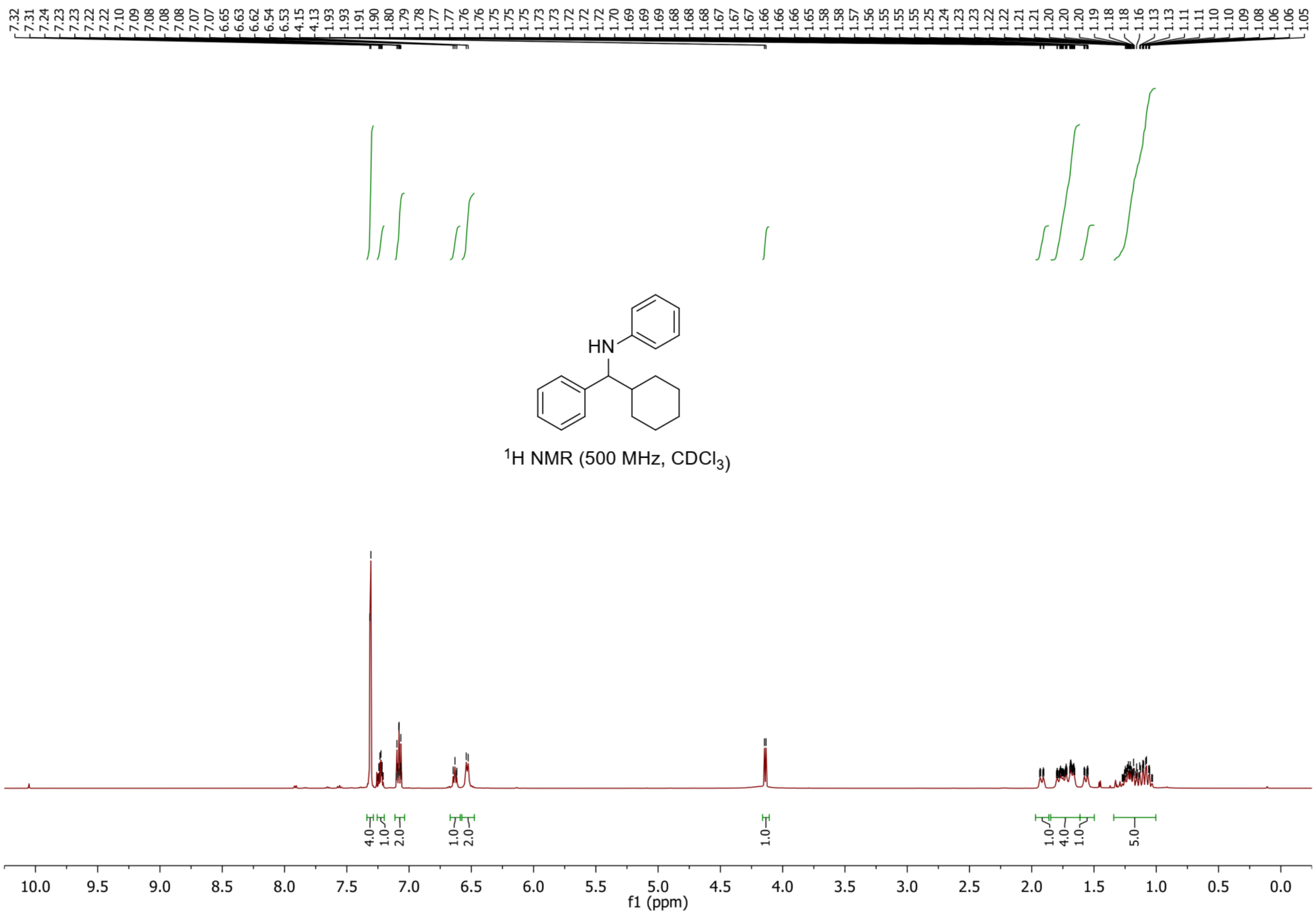

N-(Cyclohexyl(phenyl)methyl)aniline (4a)

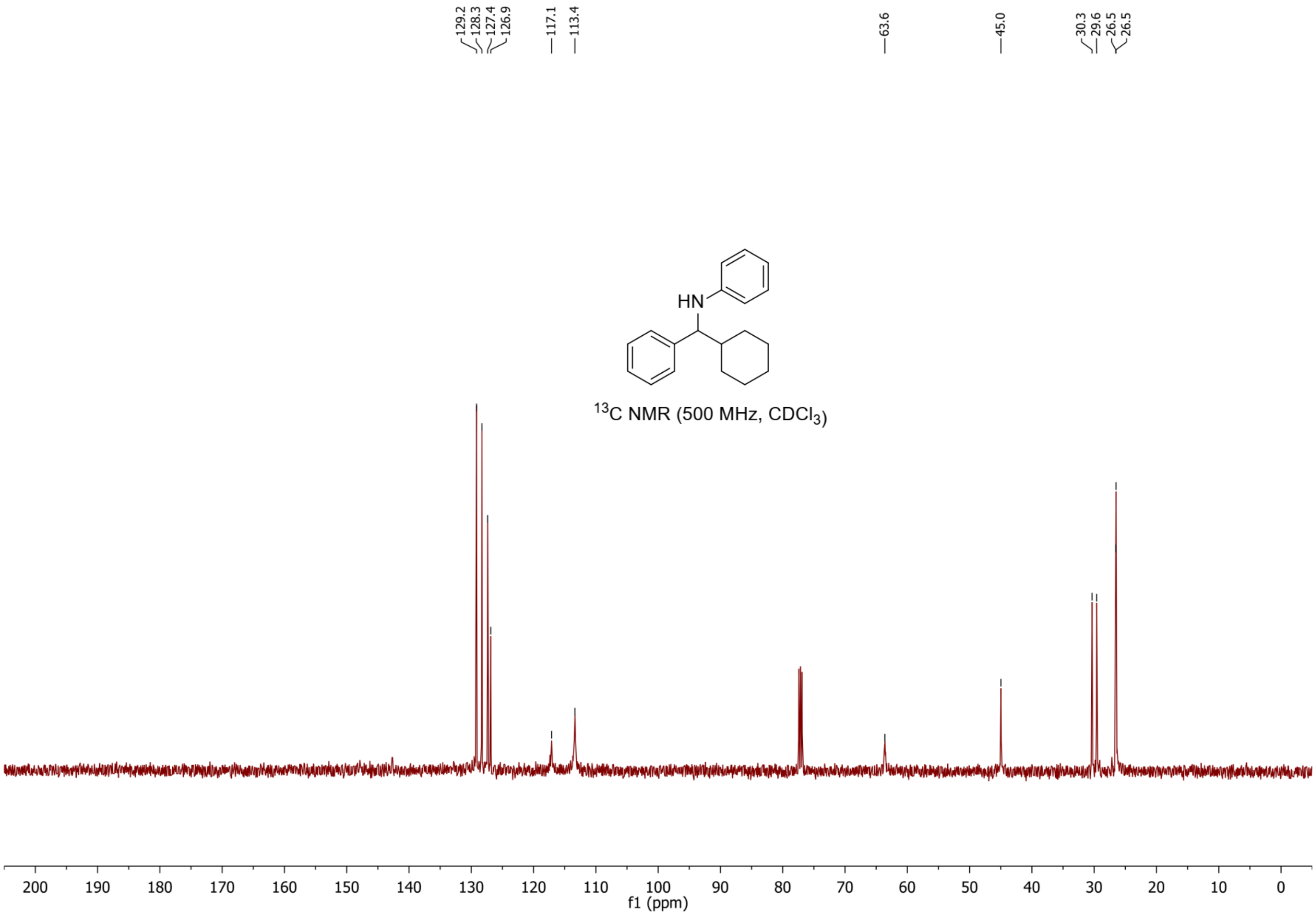

Go to table of contents

N-(Cyclohexyl(phenyl)methyl)-4-methylaniline (4b)

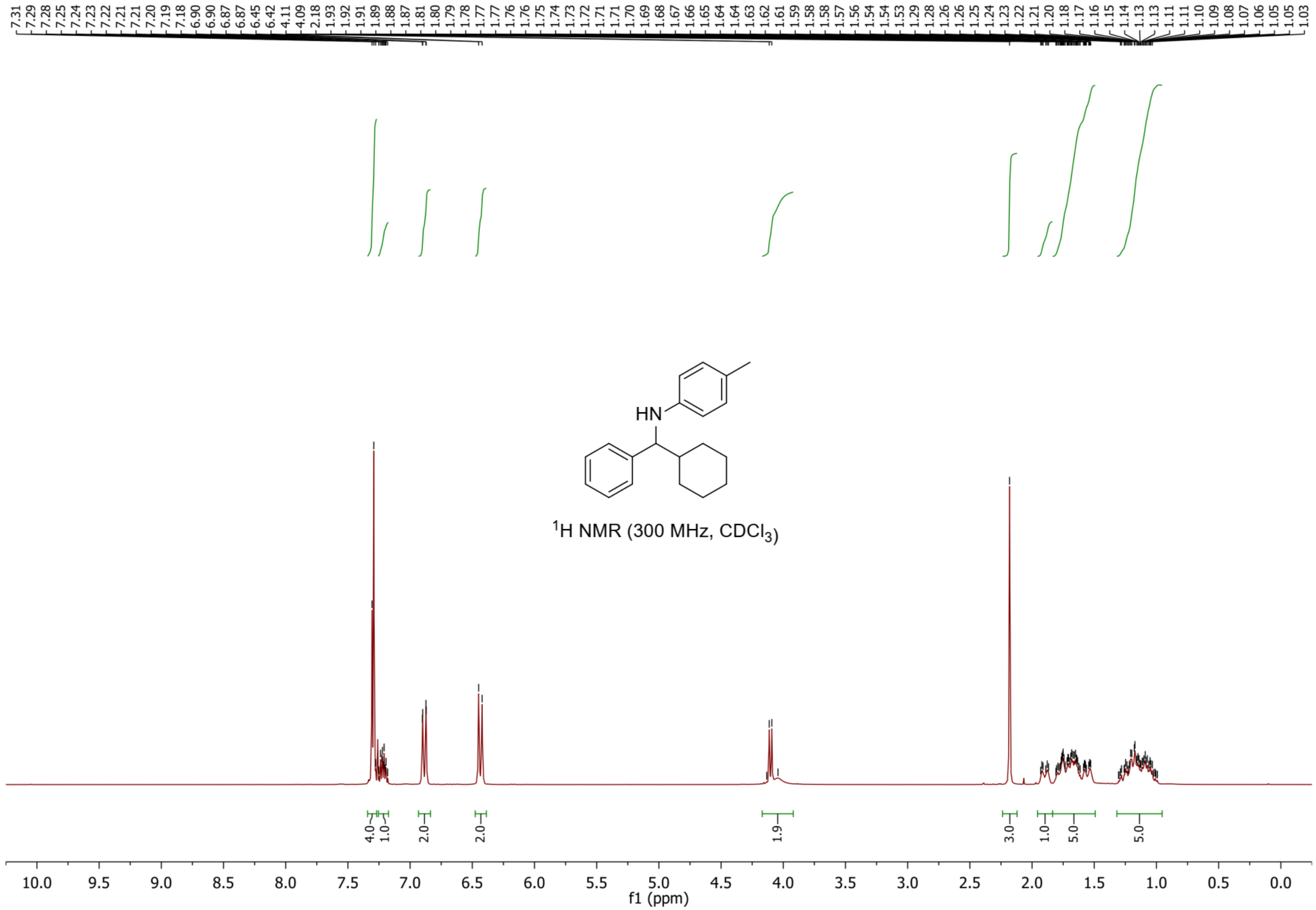

Go to table of contents

N-(Cyclohexyl(phenyl)methyl)-4-methylaniline (4b)

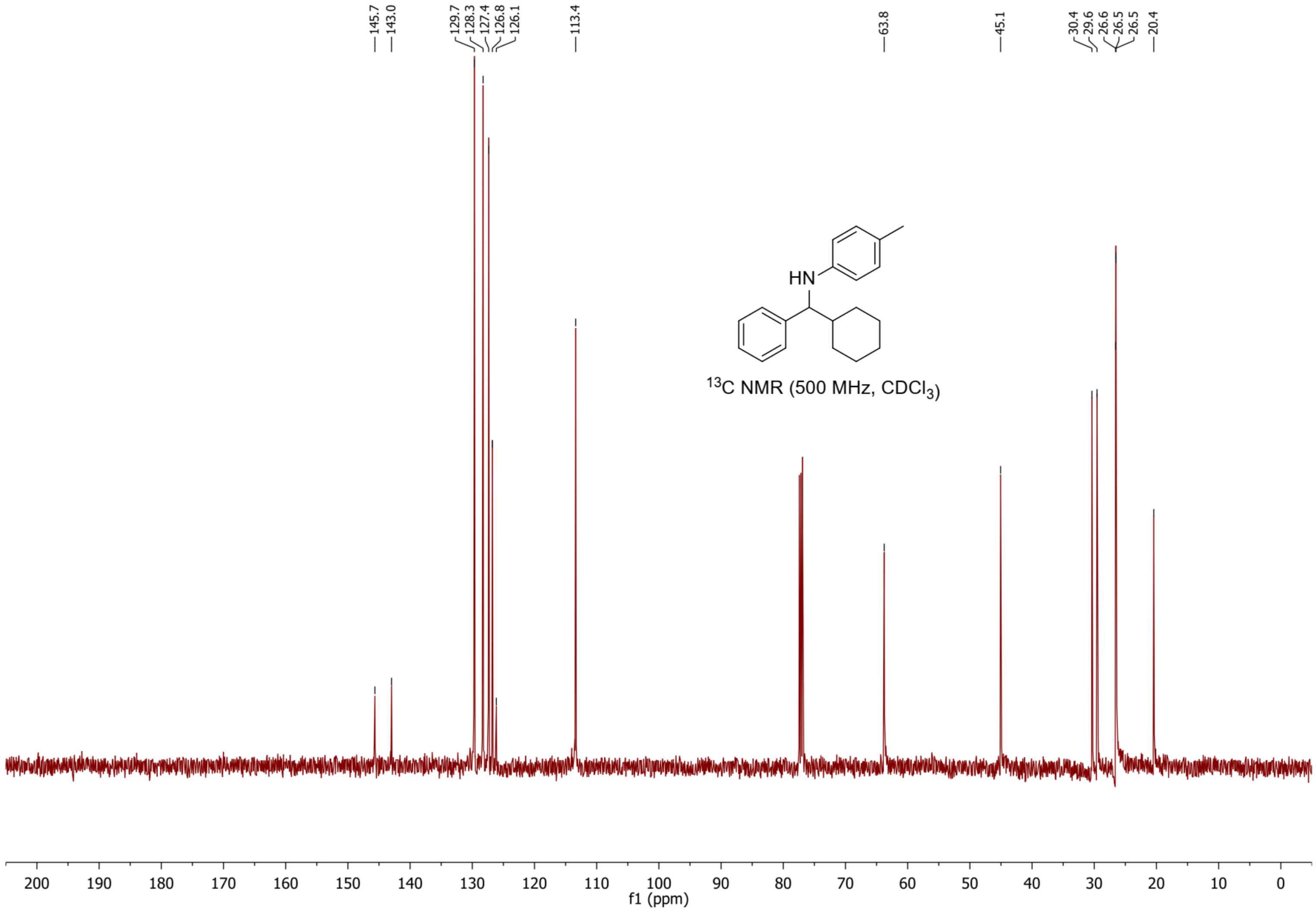

Go to table of contents

N-(Cyclohexyl(phenyl)methyl)-3,4,5-trimethylaniline (4c)

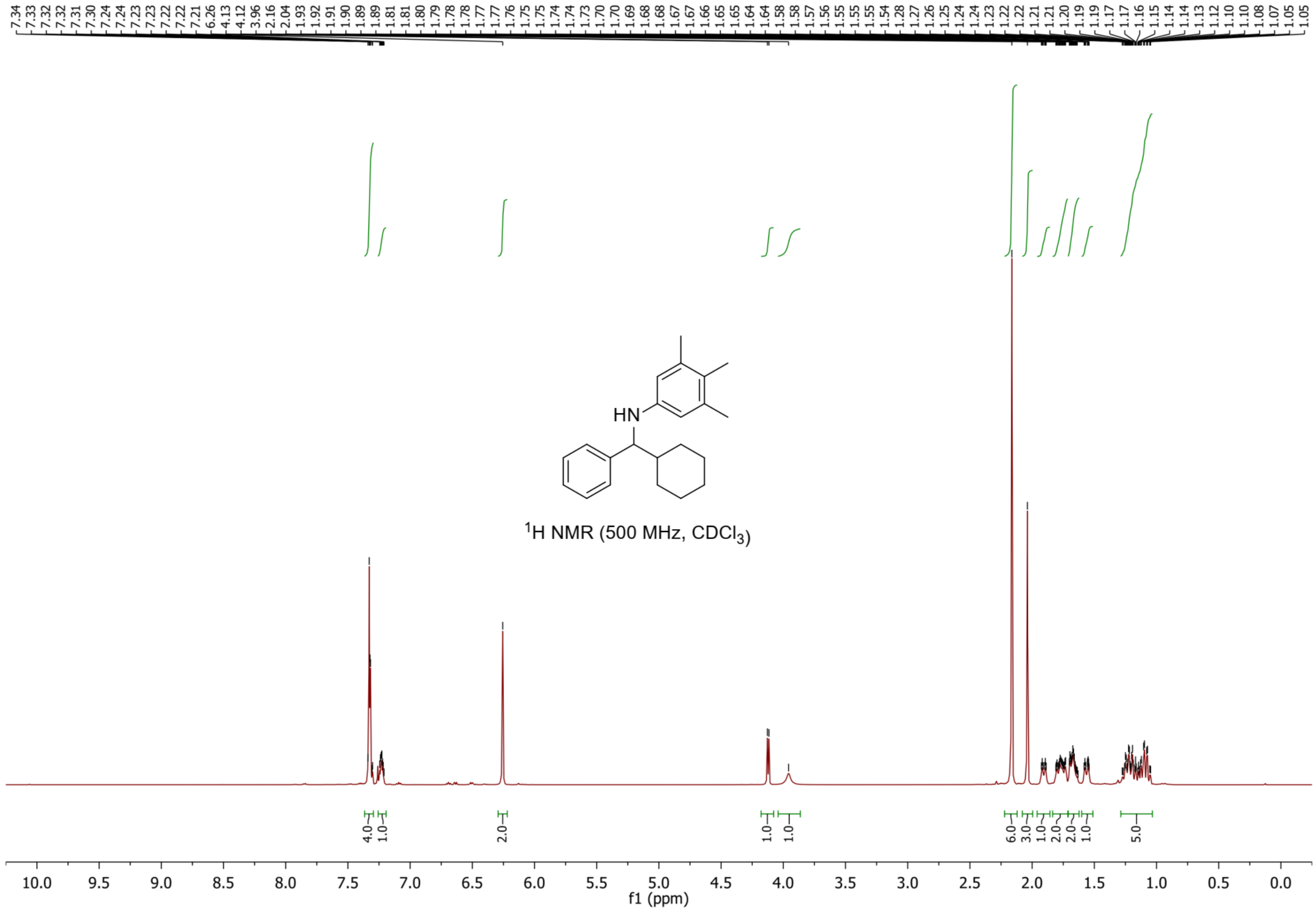

Go to table of contents

_N_-(Cyclohexyl(phenyl)methyl)-3,4,5-trimethylaniline (4c)

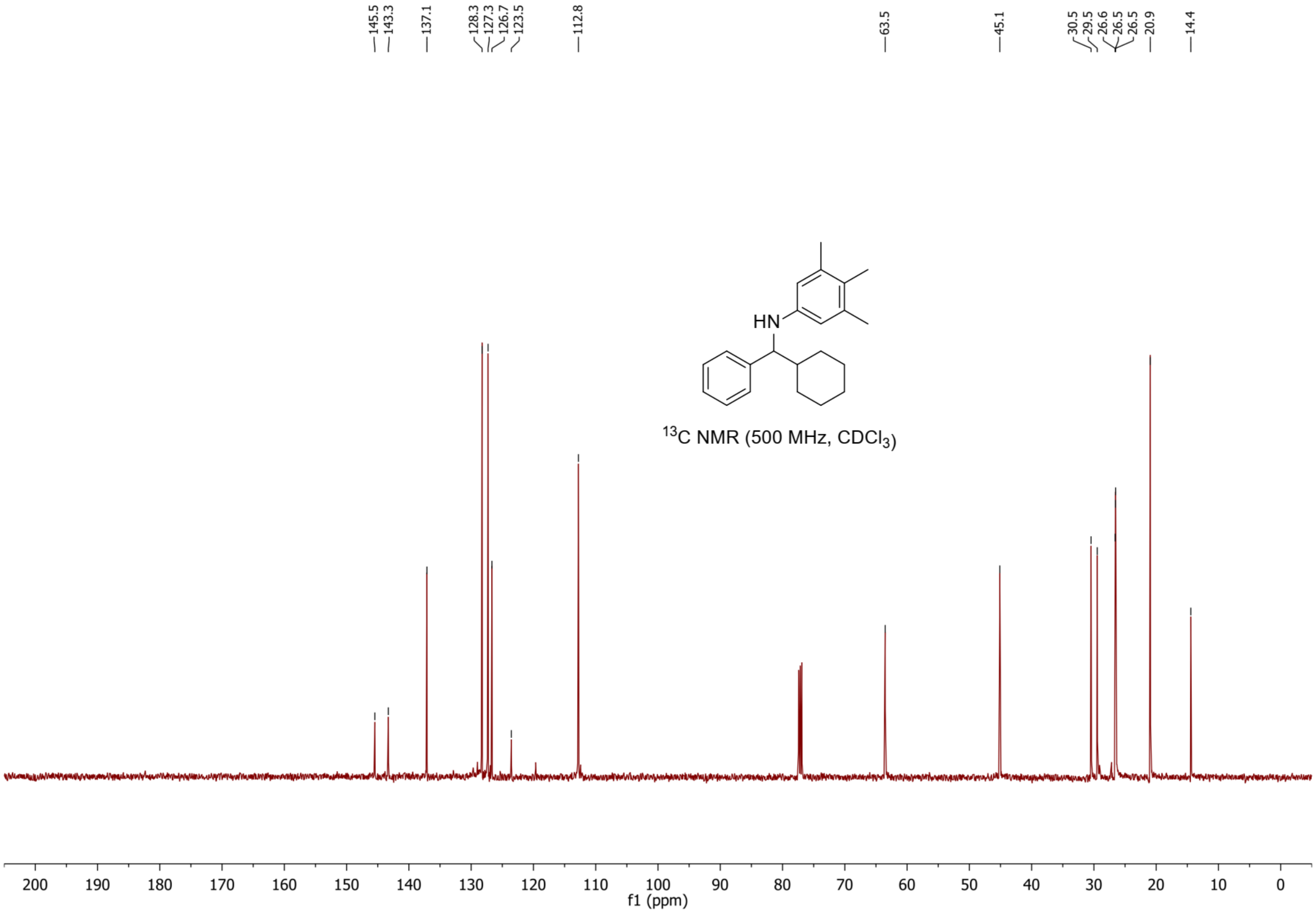

Go to table of contents

N-(Cyclohexyl(phenyl)methyl)-2,3-dihydro-1H-inden-5-amine (4d)

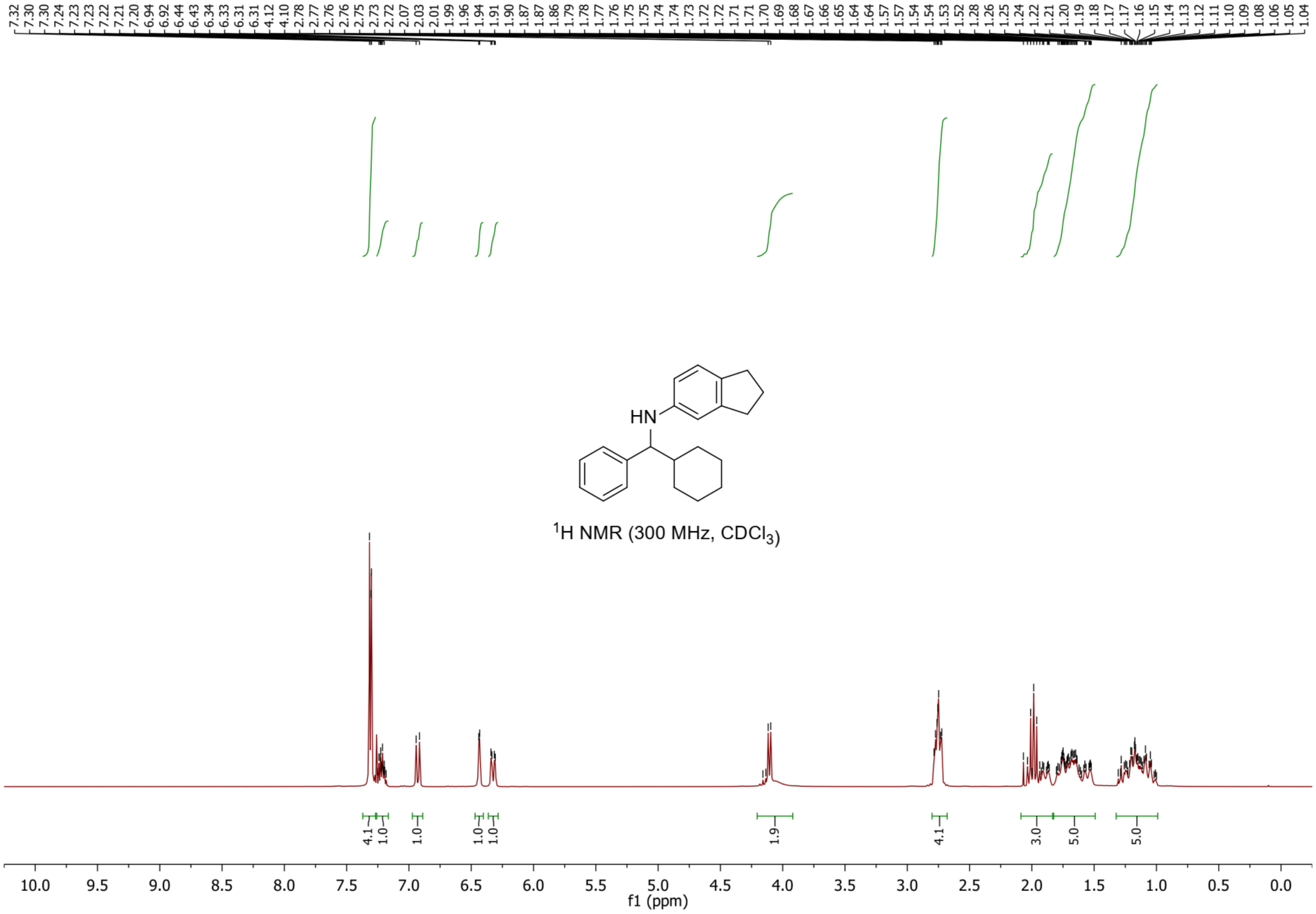

Go to table of contents

N-(Cyclohexyl(phenyl)methyl)-2,3-dihydro-1H-inden-5-amine (4d)

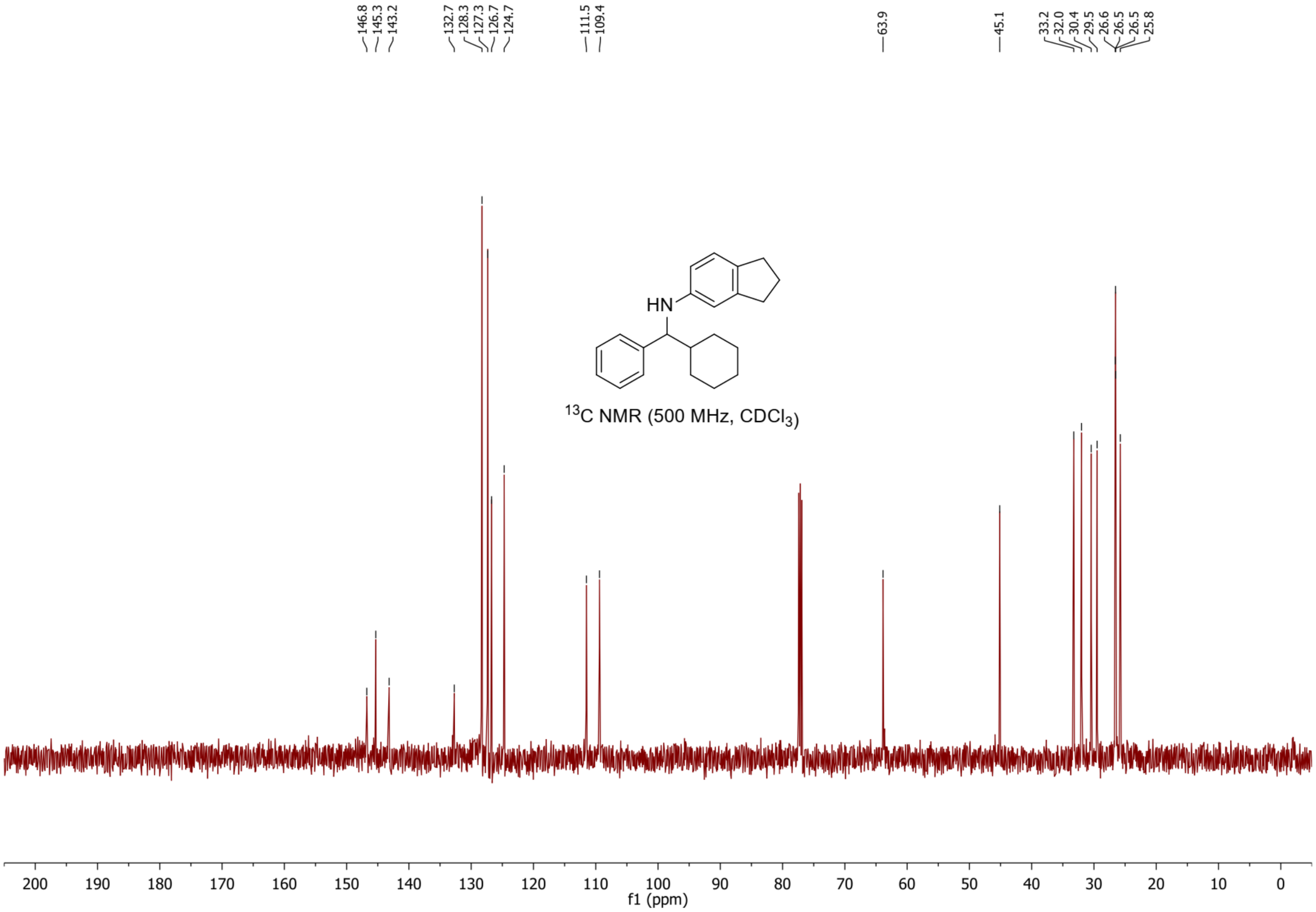

Go to table of contents

N-(Cyclohexyl(phenyl)methyl)-4-fluoroaniline (4e)

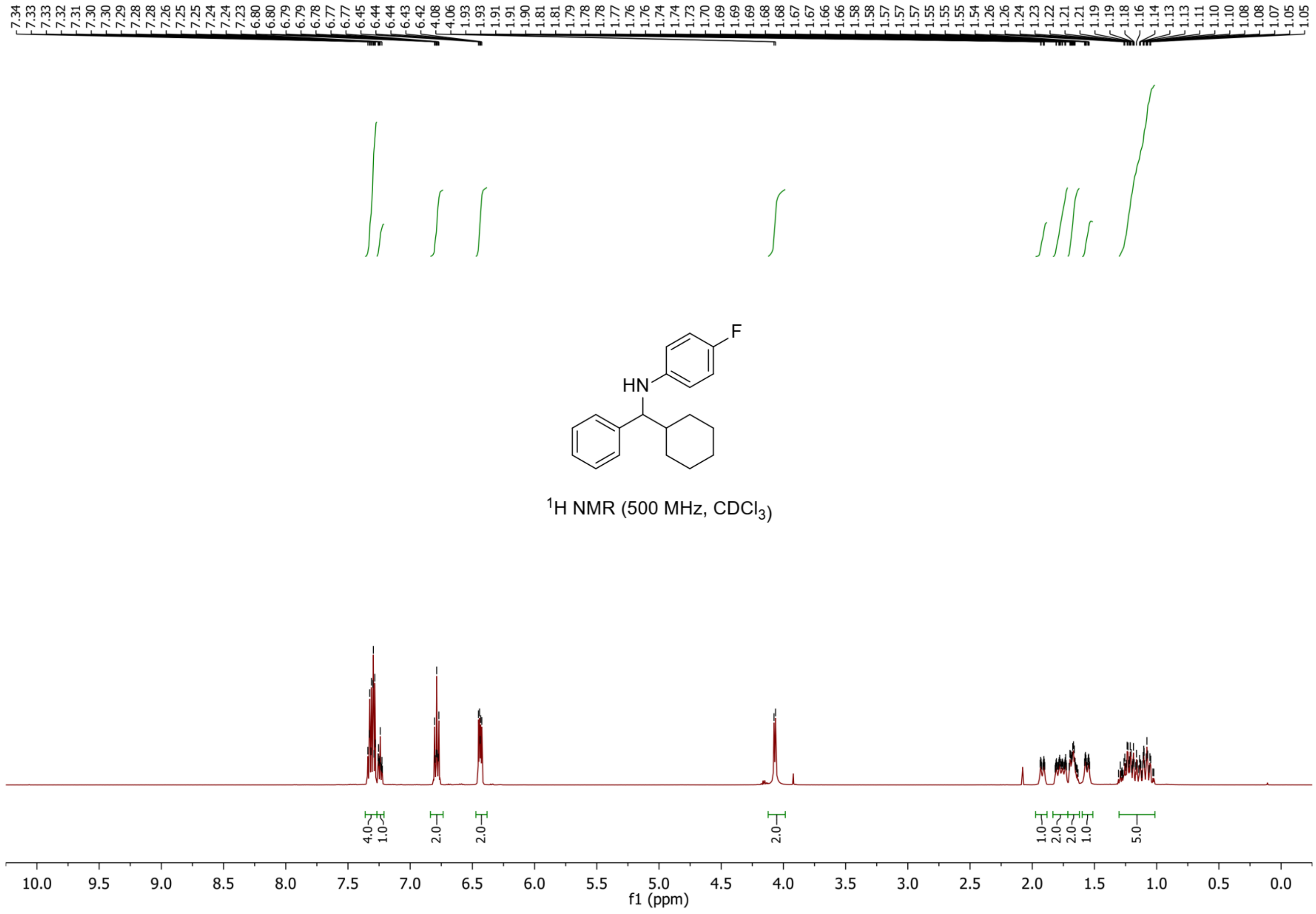

Go to table of contents

N-(Cyclohexyl(phenyl)methyl)-4-fluoroaniline (4e)

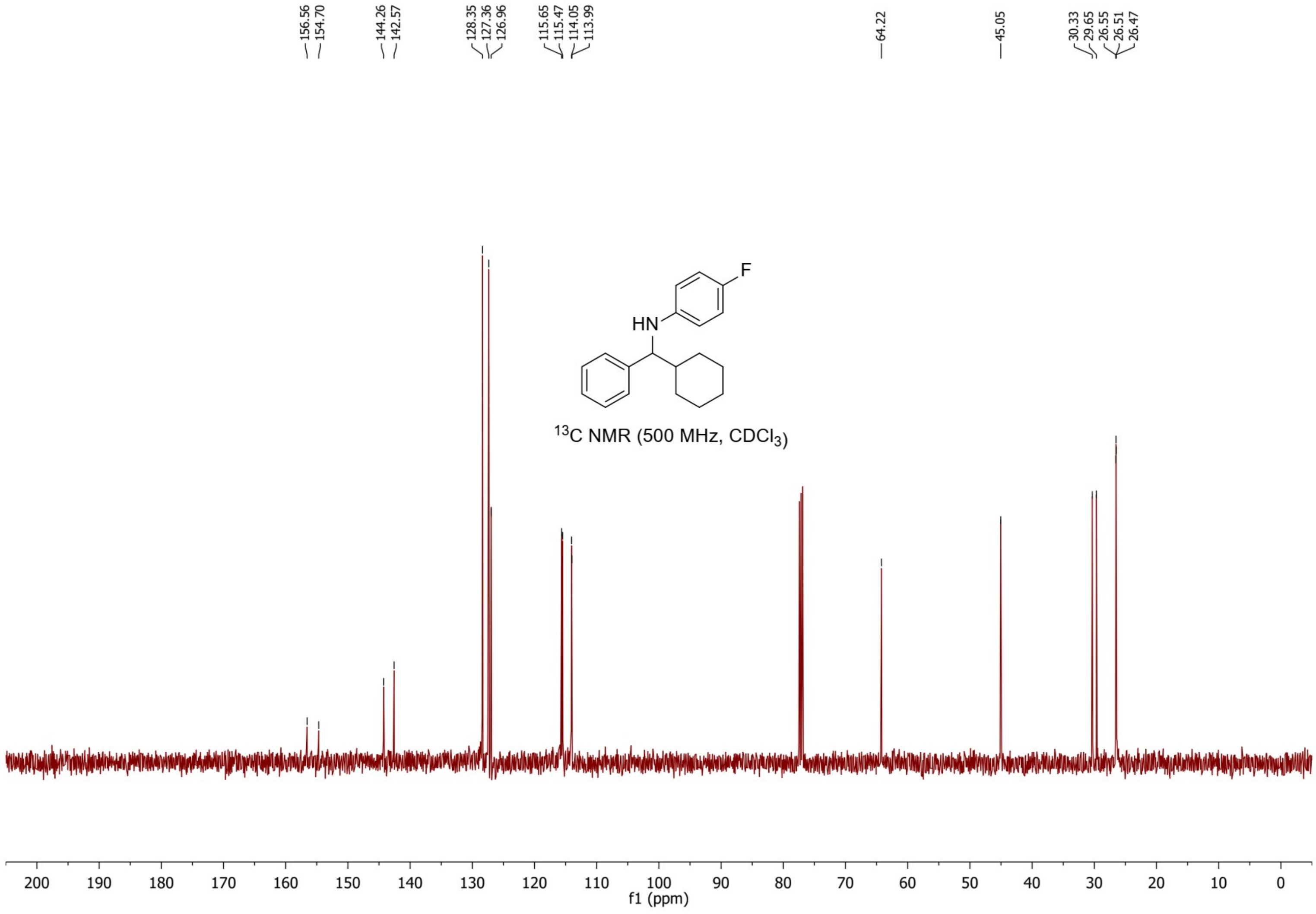

Go to table of contents

4-Chloro-*N*-(cyclohexyl(phenyl)methyl)aniline (4f)

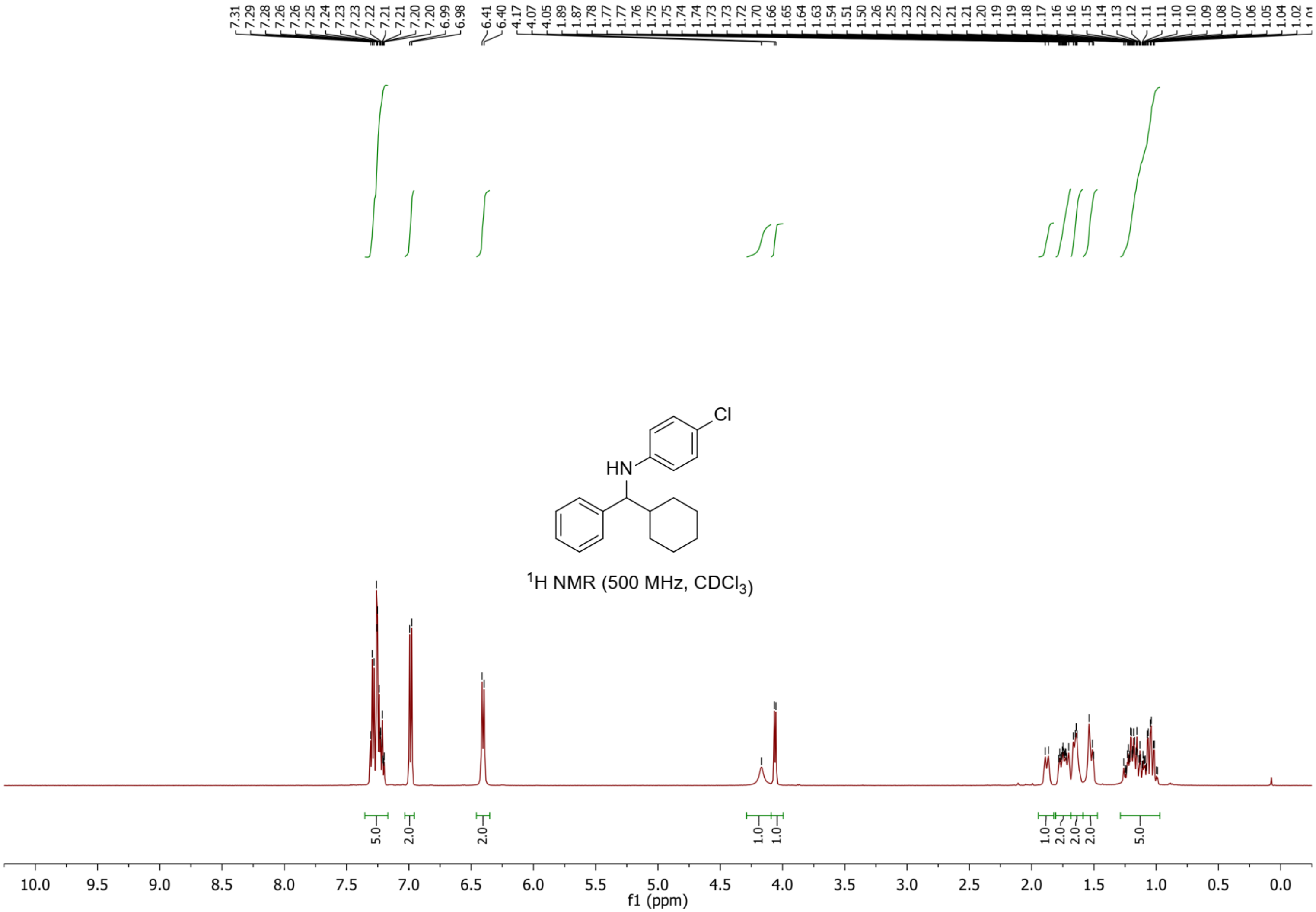

4-Chloro-*N*-(cyclohexyl(phenyl)methyl)aniline (4f)

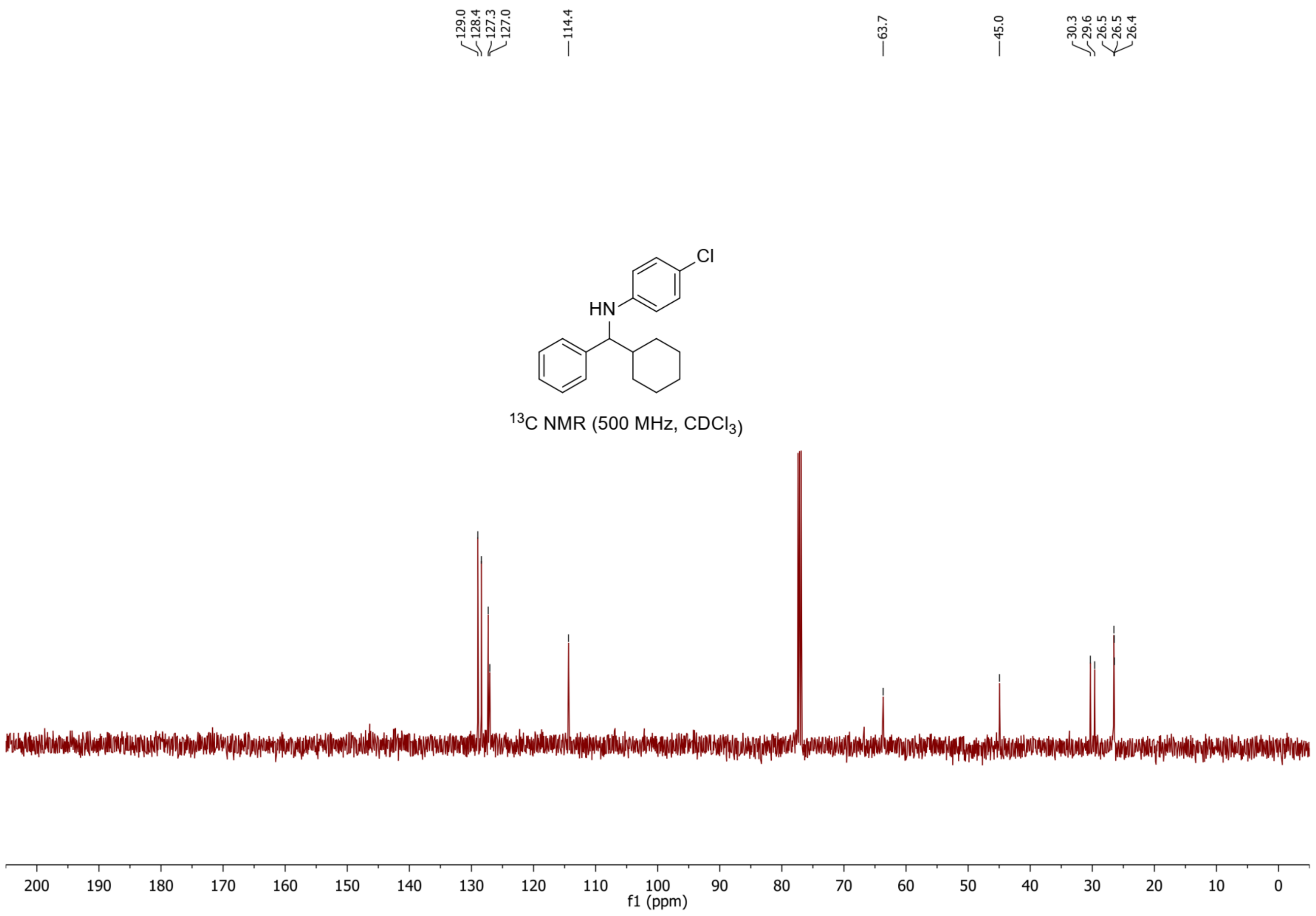

Go to table of contents

3-Bromo-*N*-(cyclohexyl(phenyl)methyl)aniline (4g)

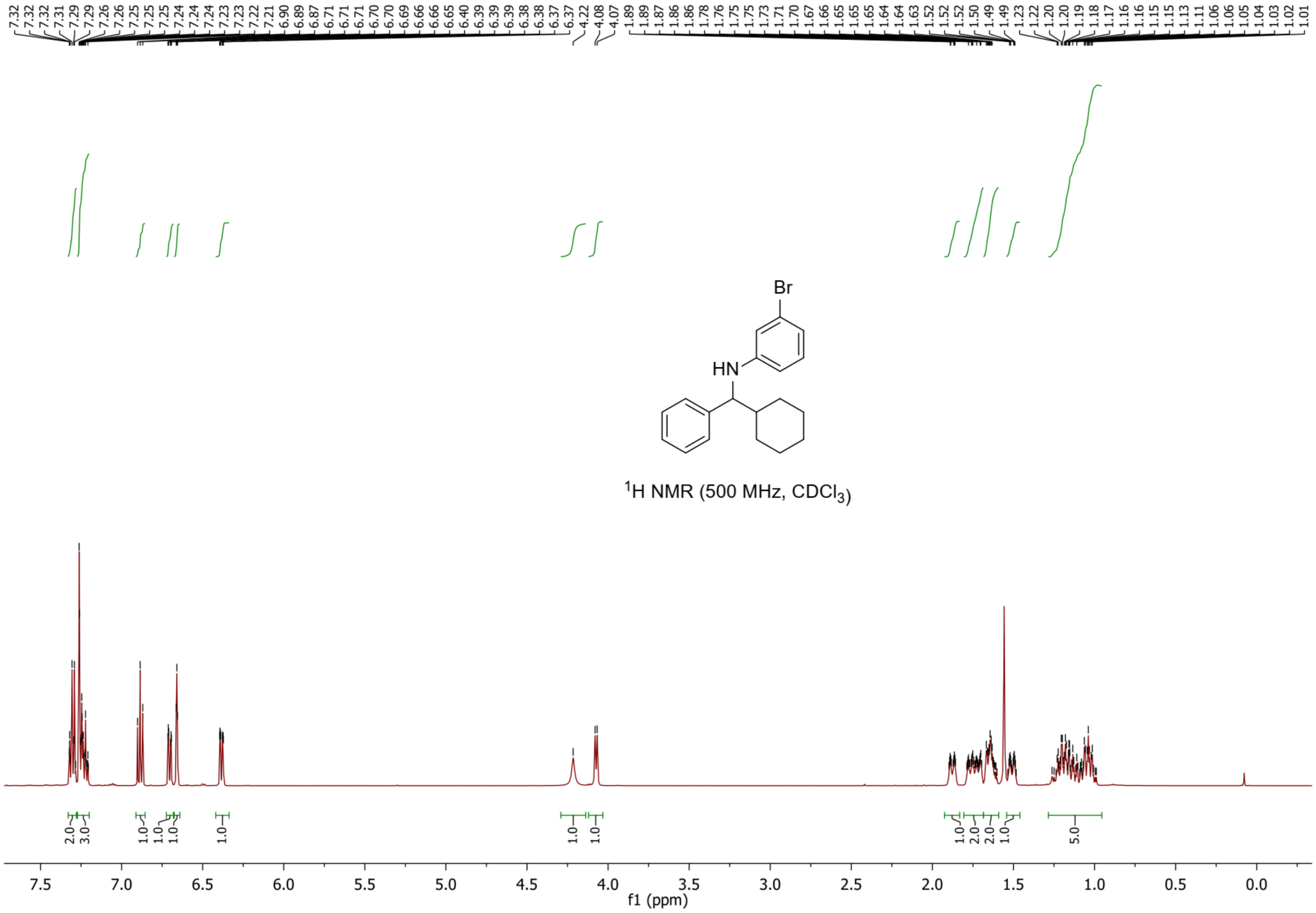

Go to table of contents

3-Bromo-*N*-(cyclohexyl(phenyl)methyl)aniline (4g)

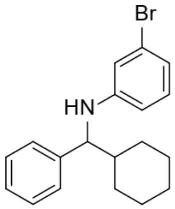

^{13}C NMR (500 MHz, $CDCl_3$)

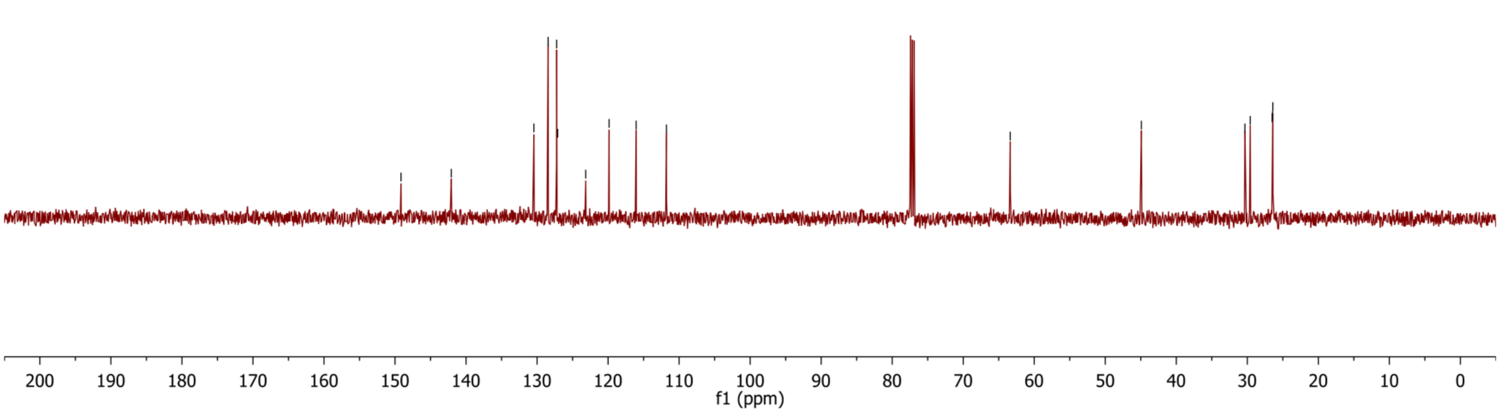

Go to table of contents

N-(Cyclohexyl(phenyl)methyl)-3-iodoaniline (4h)

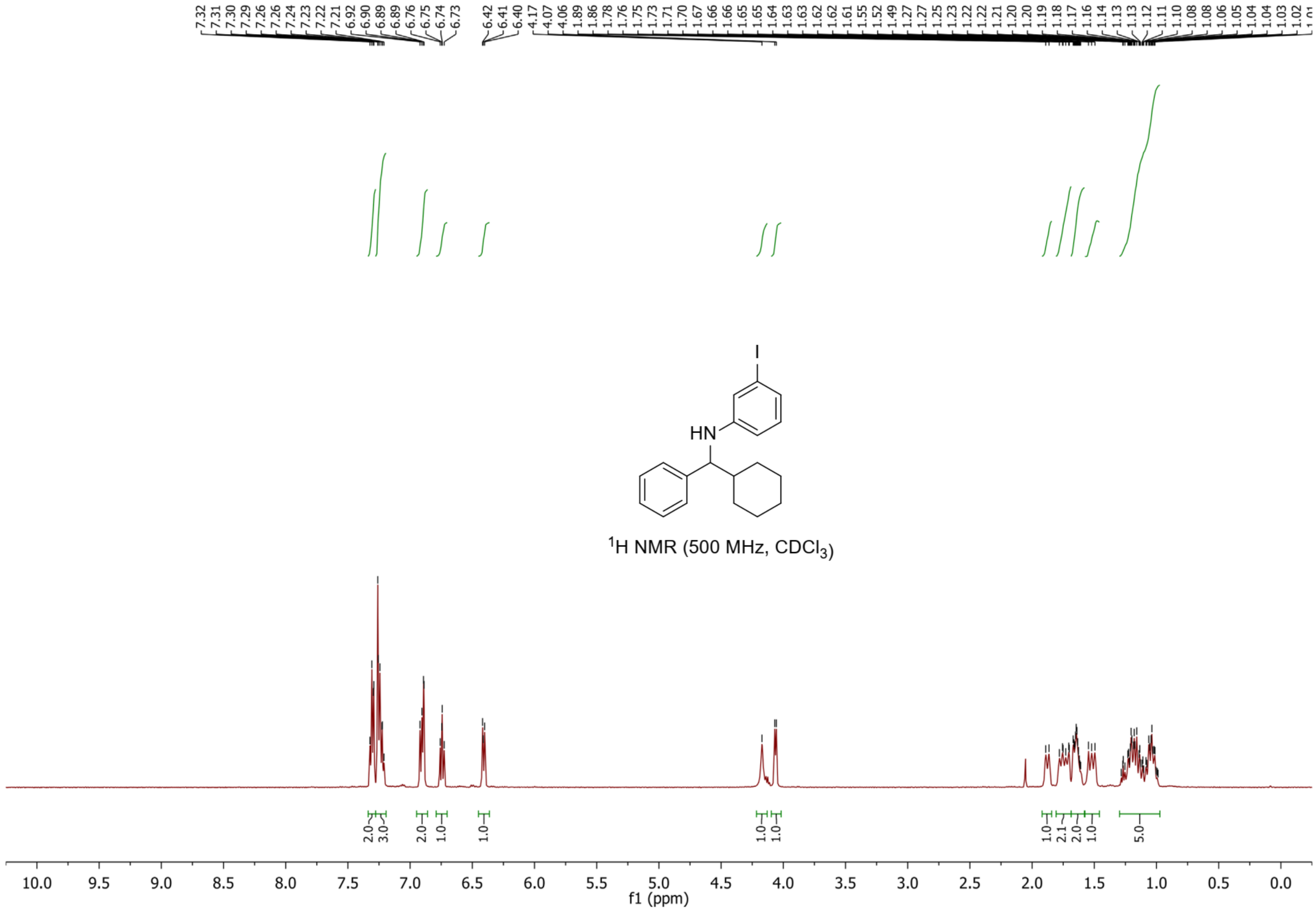

Go to table of contents

_N_-(Cyclohexyl(phenyl)methyl)-3-iodoaniline (4h)

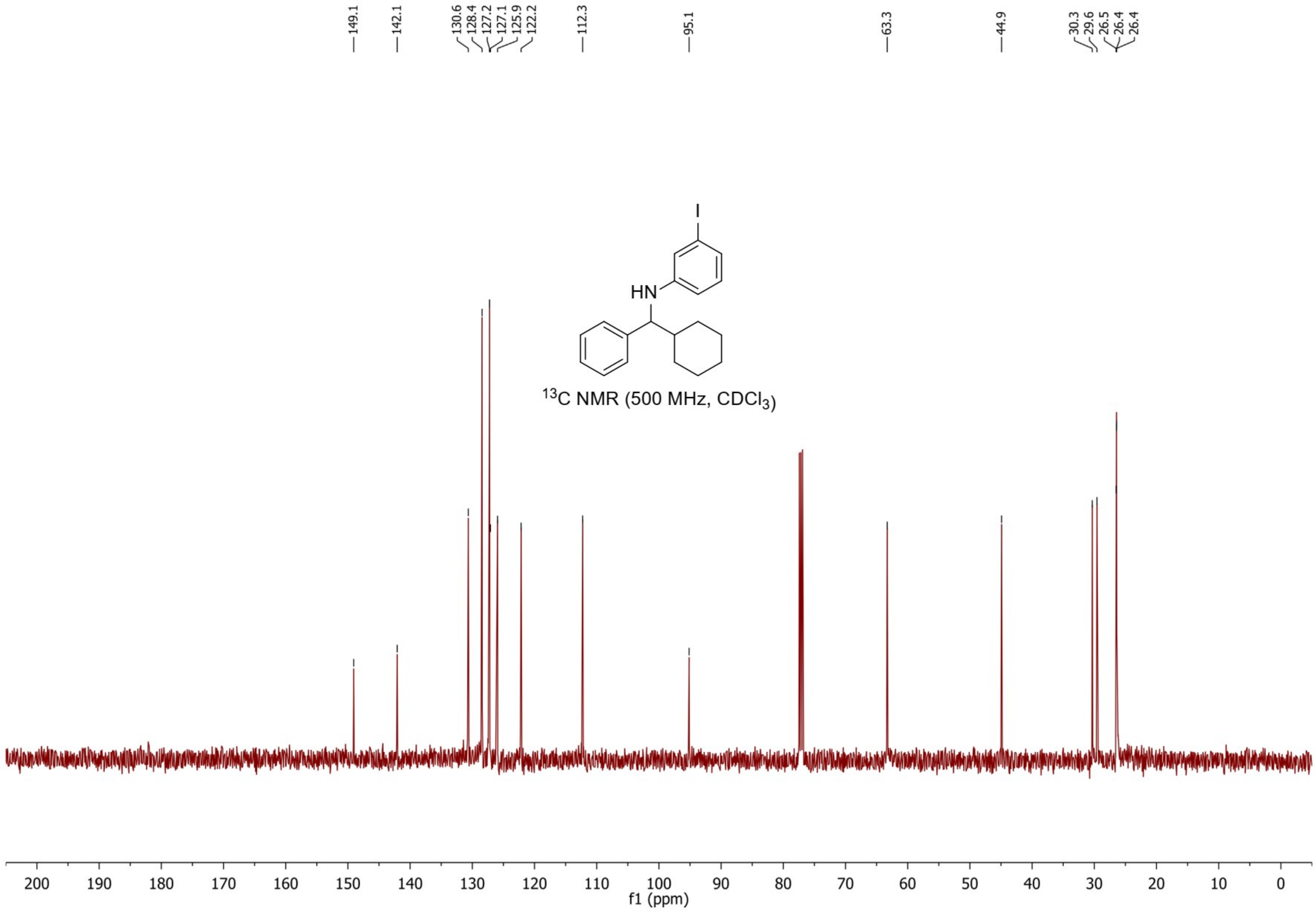

N-(Cyclohexyl(phenyl)methyl)-2-methoxyaniline (4i)

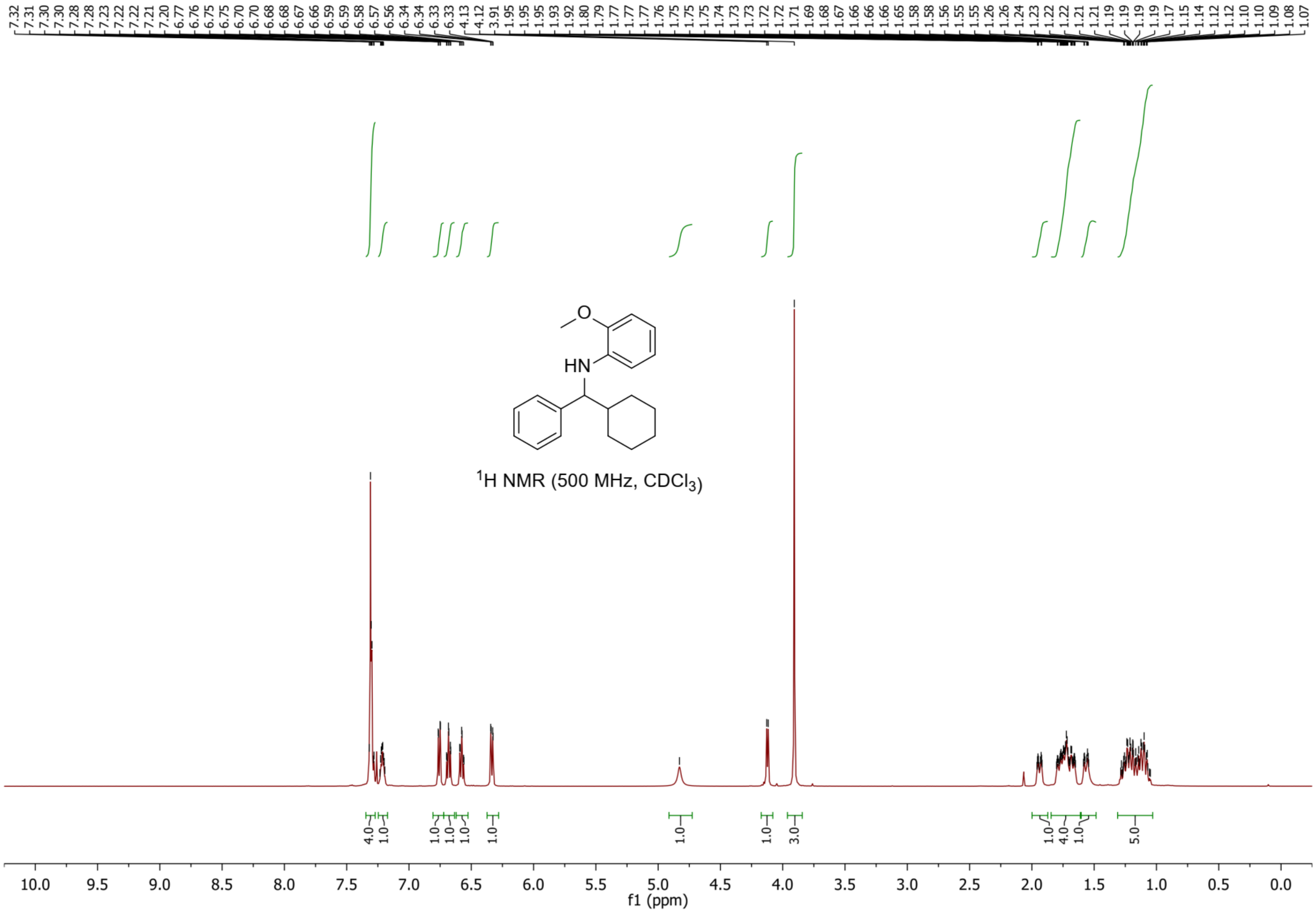

Go to table of contents

N-(Cyclohexyl(phenyl)methyl)-2-methoxyaniline (4i)

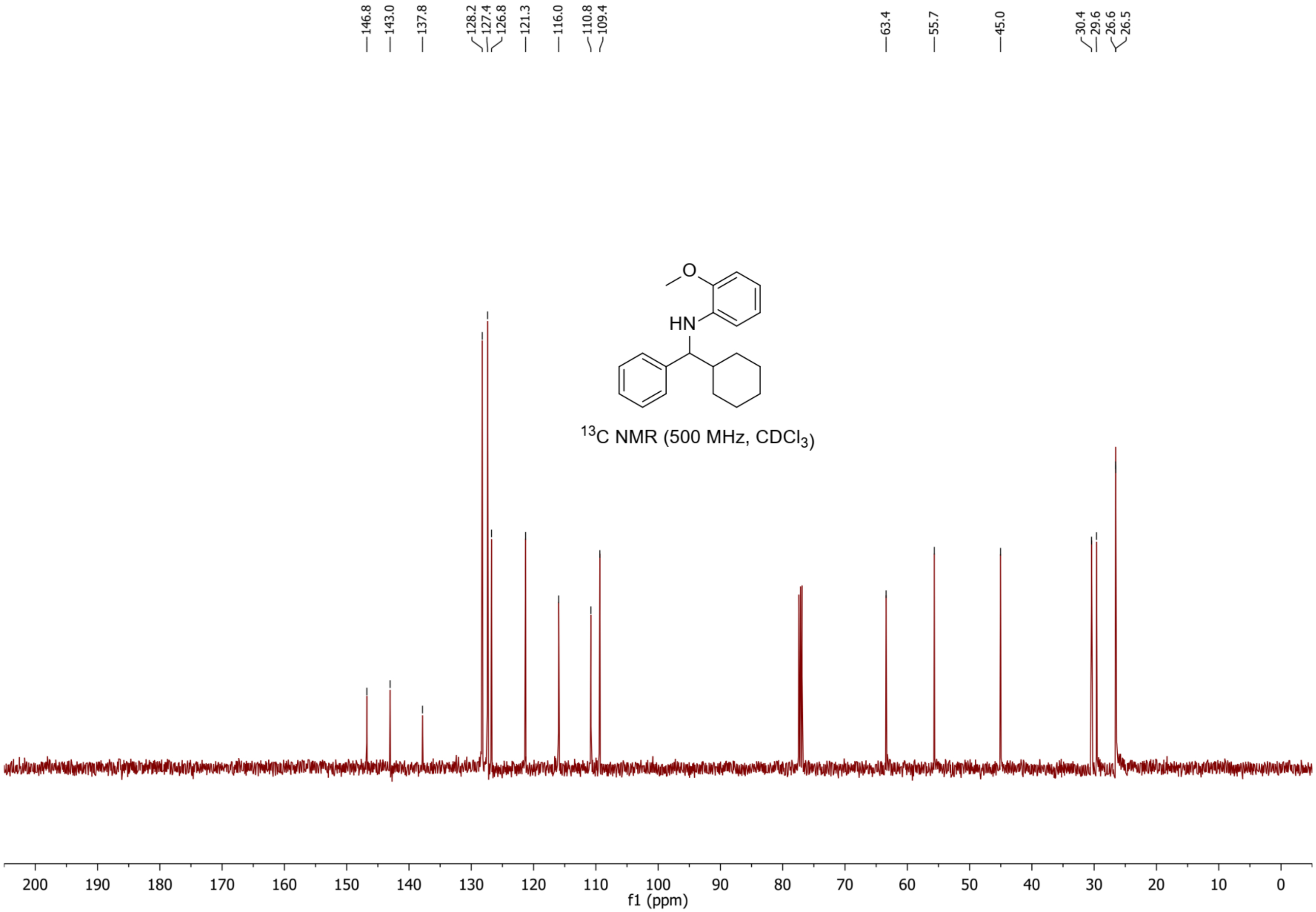

_N_-(Cyclohexyl(phenyl)methyl)-3-(methylthio)aniline (4j)

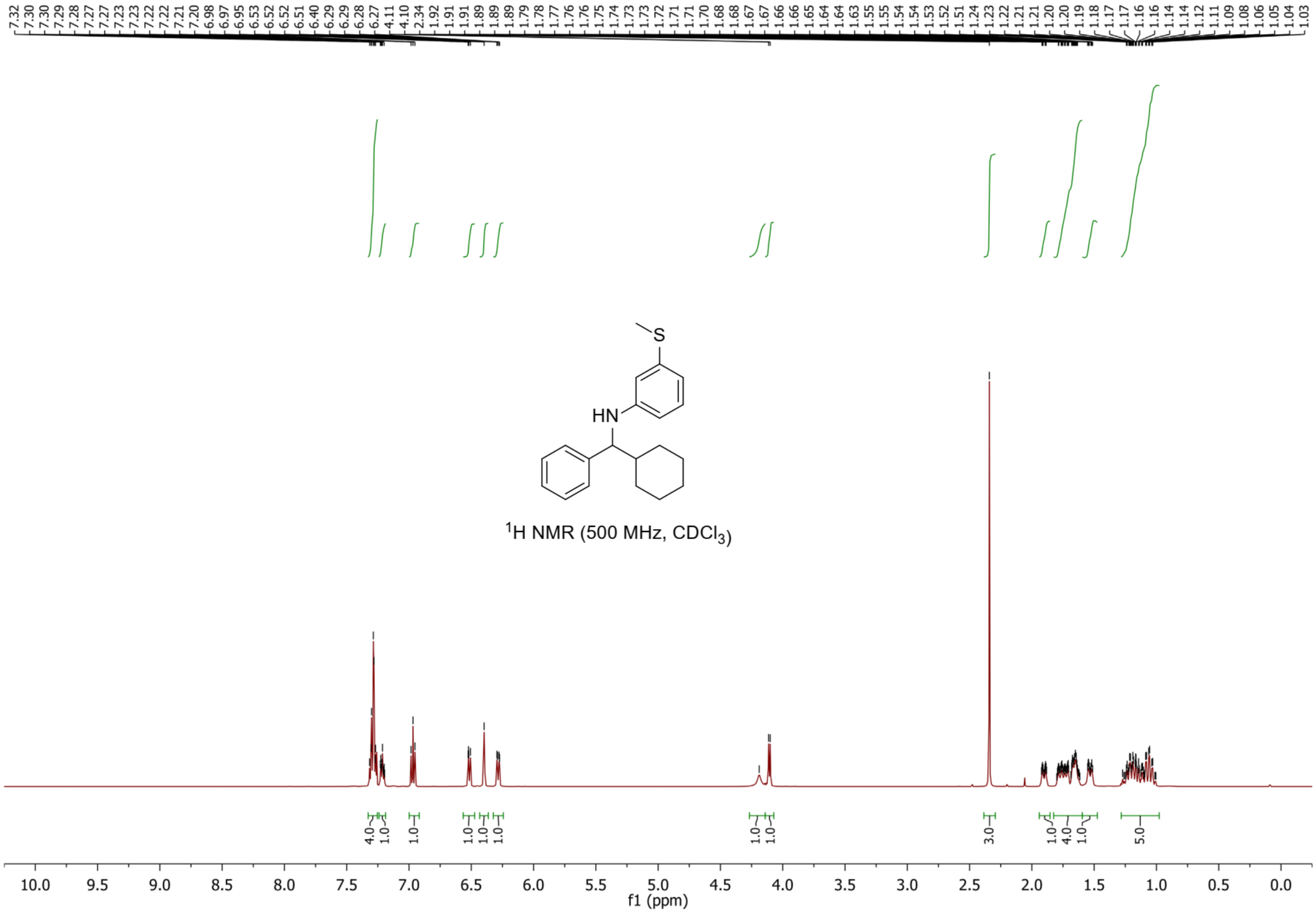

Go to table of contents

N-(Cyclohexyl(phenyl)methyl)-3-(methylthio)aniline (4j)

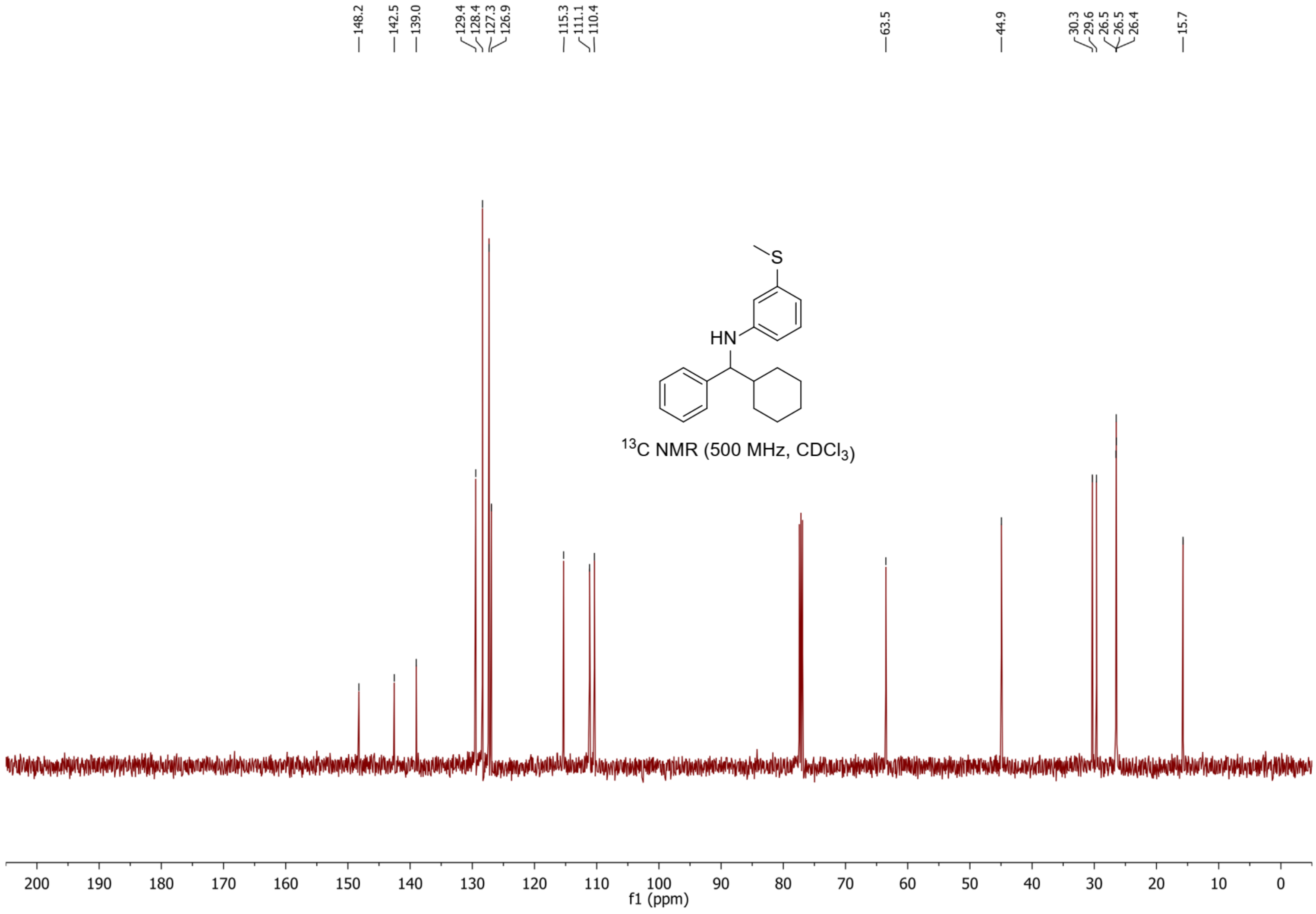

Go to table of contents

N-(Cyclohexyl(phenyl)methyl)-3-(trifluoromethoxy)aniline (4k)

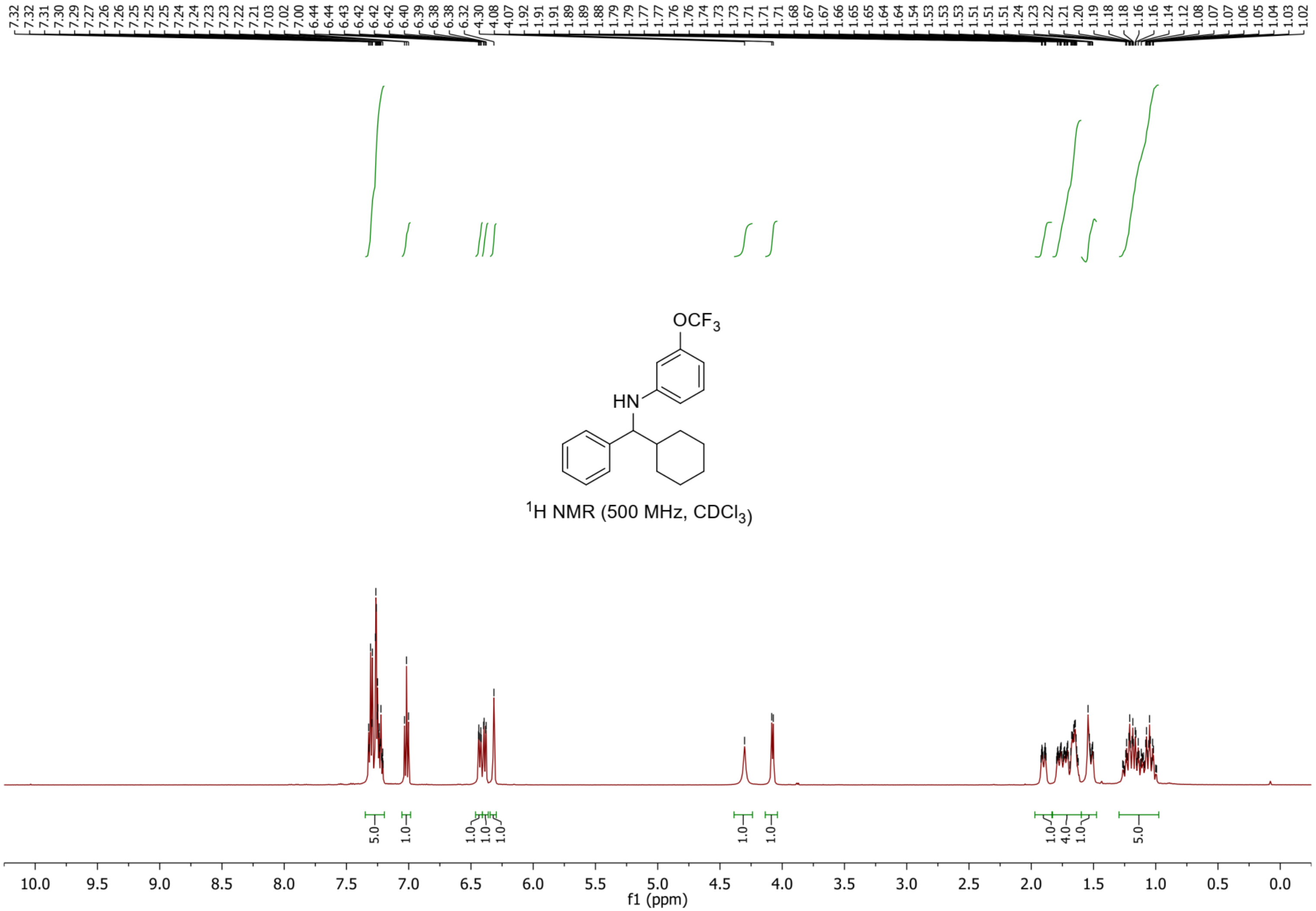

***N*-(Cyclohexyl(phenyl)methyl)-3-(trifluoromethoxy)aniline (4k)**

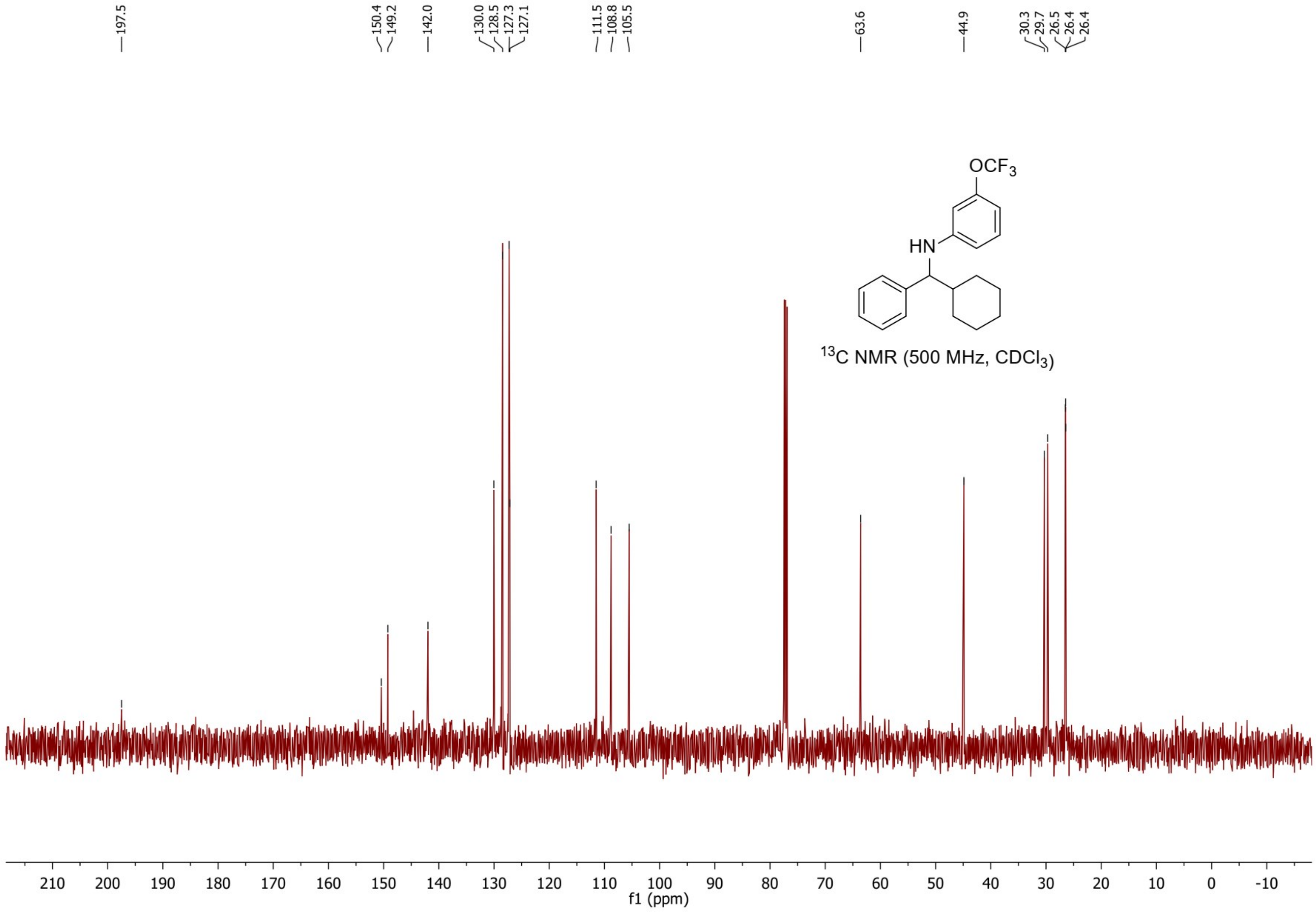

Go to table of contents

N-(Cyclohexyl(phenyl)methyl)-[1,1'-biphenyl]-4-amine (4l)

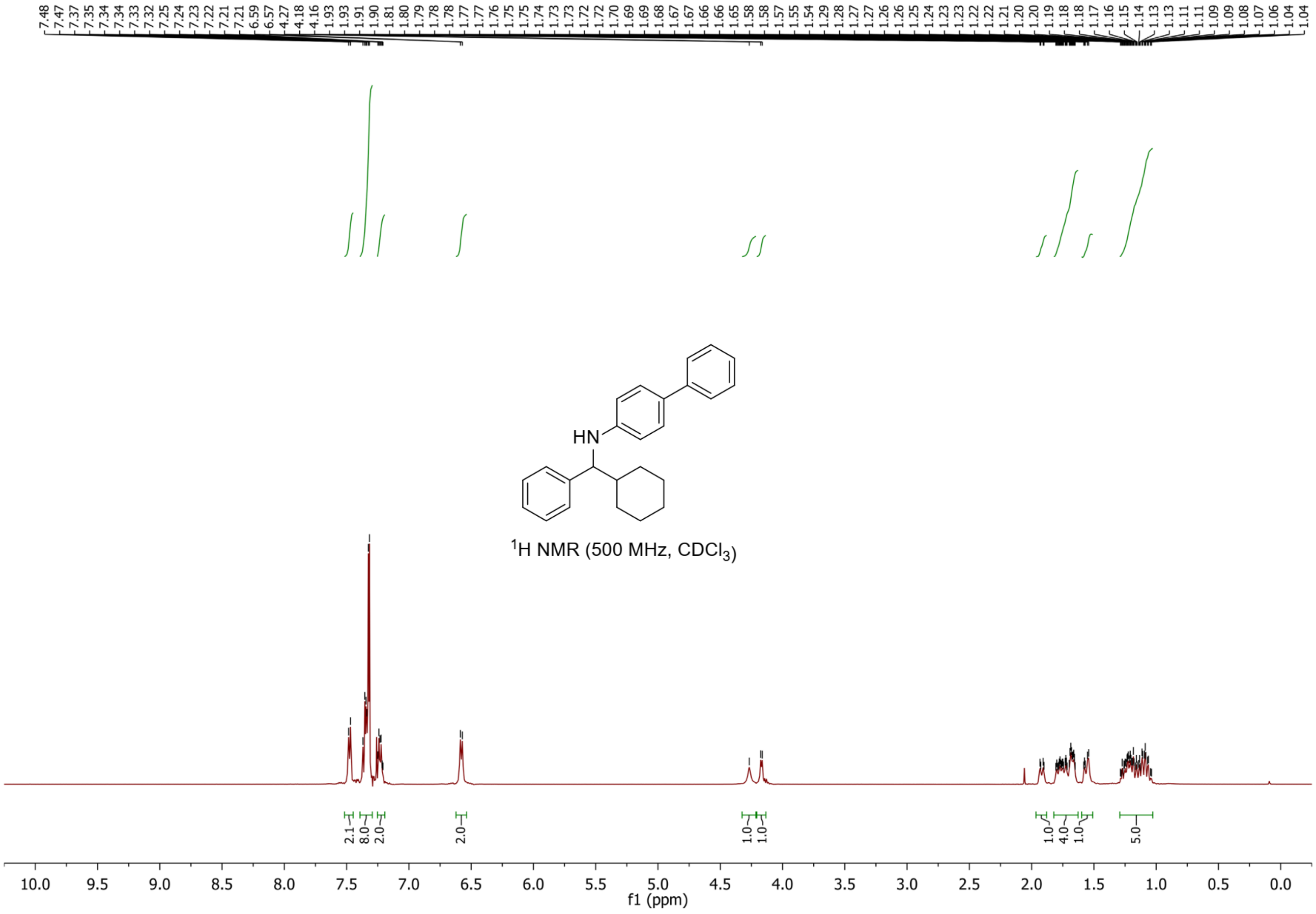

Go to table of contents

_N_-(Cyclohexyl(phenyl)methyl)-[1,1'-biphenyl]-4-amine (4l)

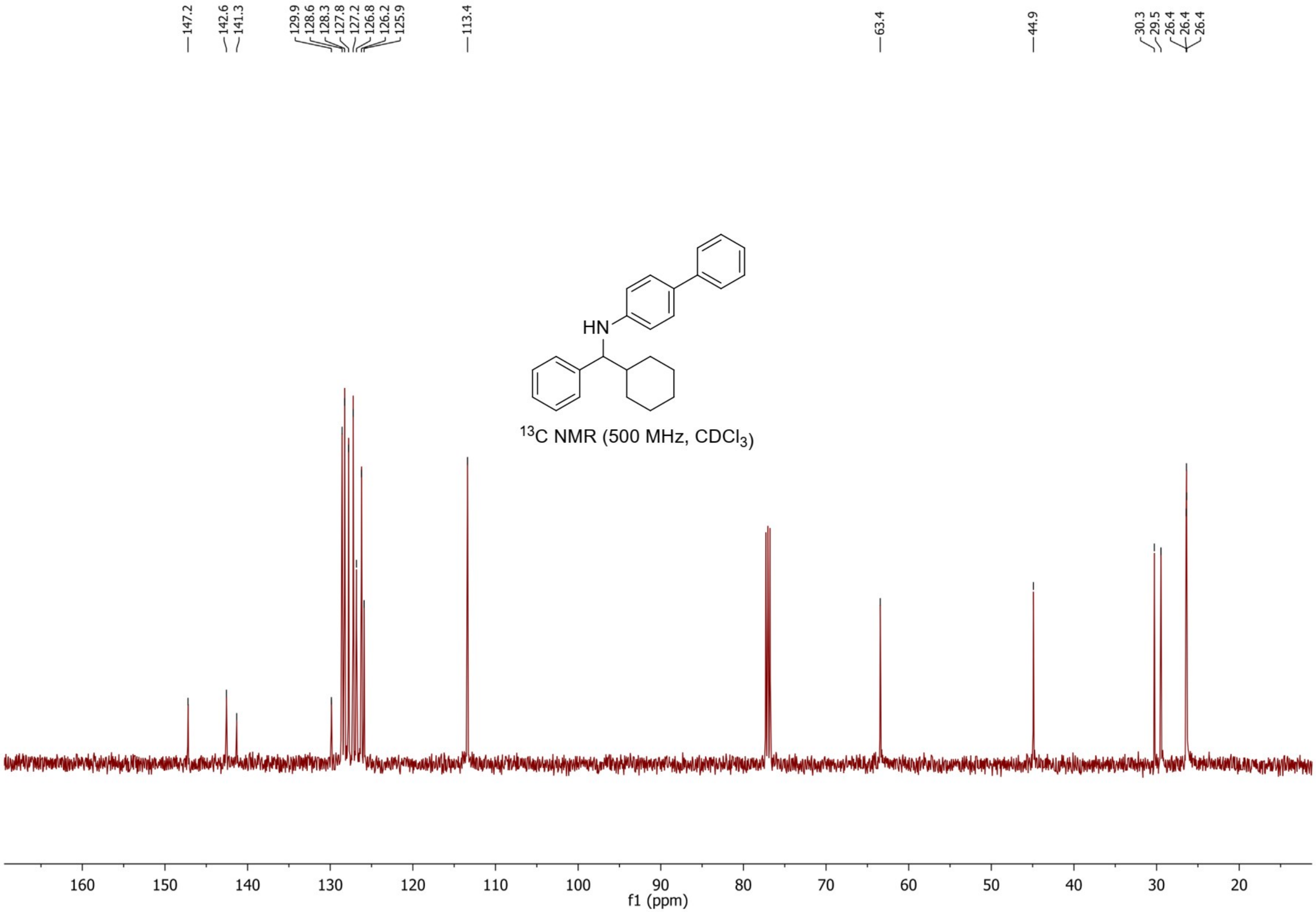

Go to table of contents

N-(Cyclohexyl(phenyl)methyl)-[1,1'-biphenyl]-2-amine (4m)

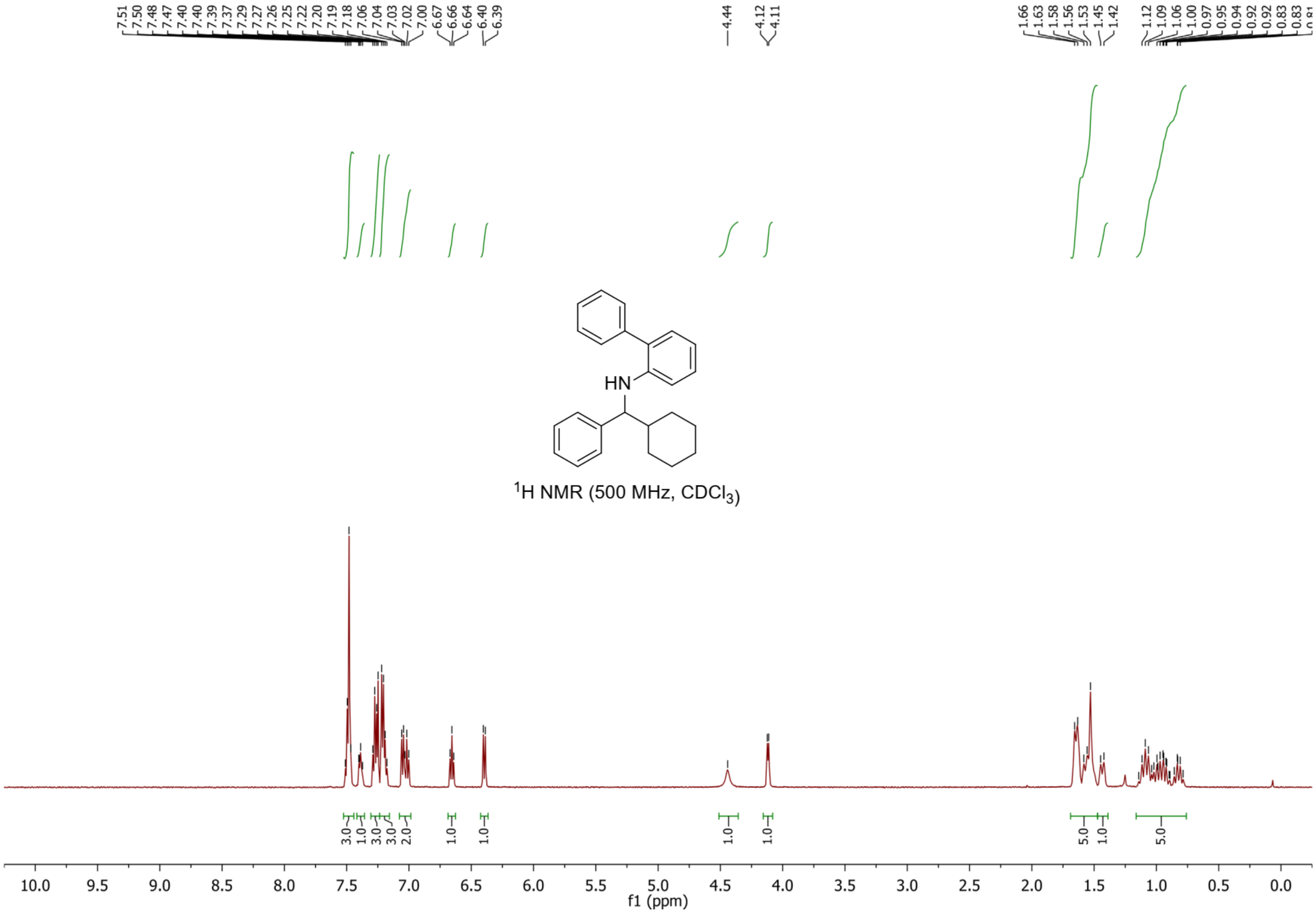

Go to table of contents

N-(Cyclohexyl(phenyl)methyl)-[1,1'-biphenyl]-2-amine (4m)

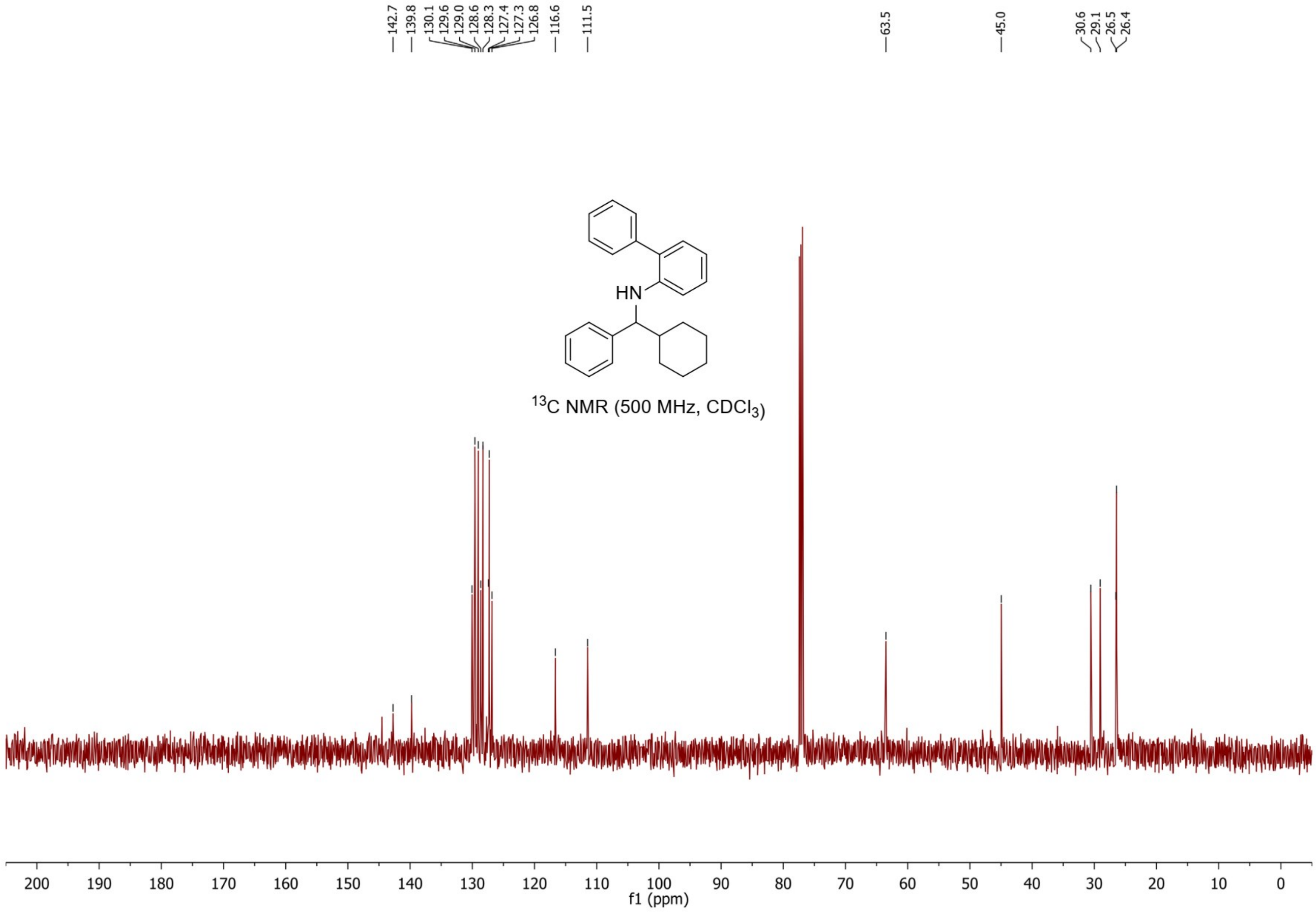

Go to table of contents

N-(Cyclohexyl(phenyl)methyl)-3-(4,4,5,5-tetramethyl-1,3,2-dioxaborolan-2-yl)aniline (4n)

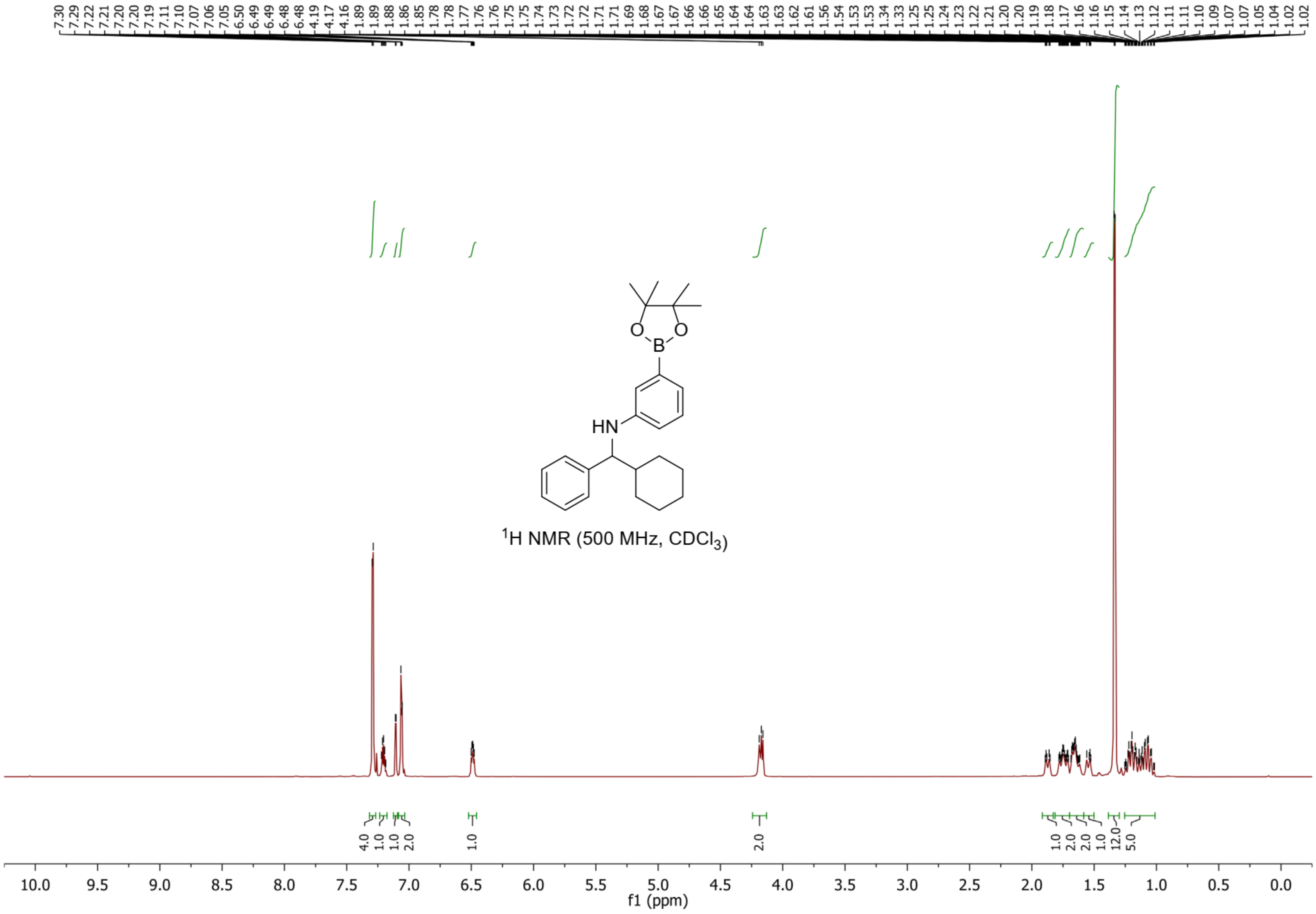

Go to table of contents

N-(Cyclohexyl(phenyl)methyl)-3-(4,4,5,5-tetramethyl-1,3,2-dioxaborolan-2-yl)aniline (4n)

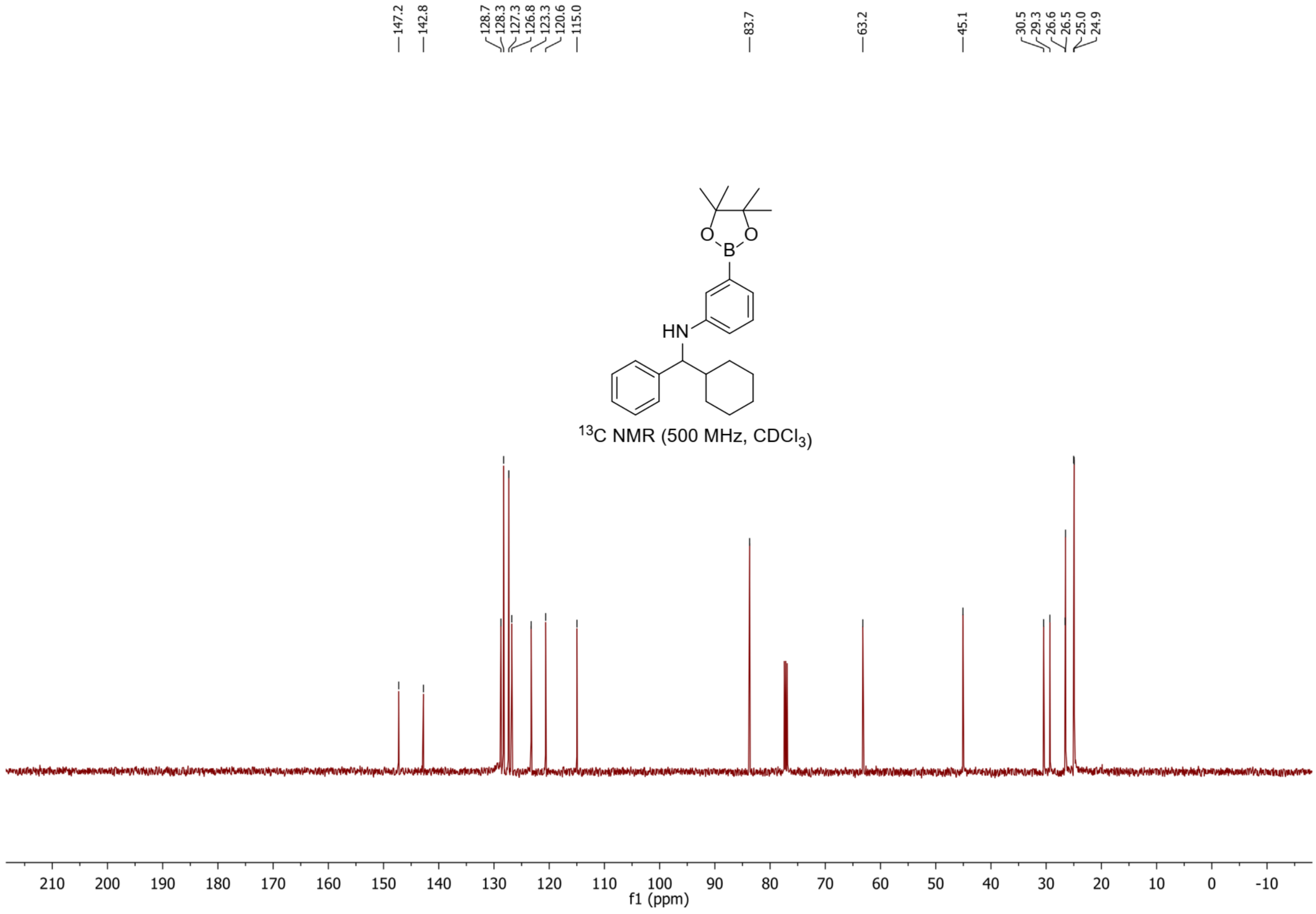

Go to table of contents

N-(Cyclohexyl(phenyl)methyl)-4-(4,4,5,5-tetramethyl-1,3,2-dioxaborolan-2-yl)aniline (4o)

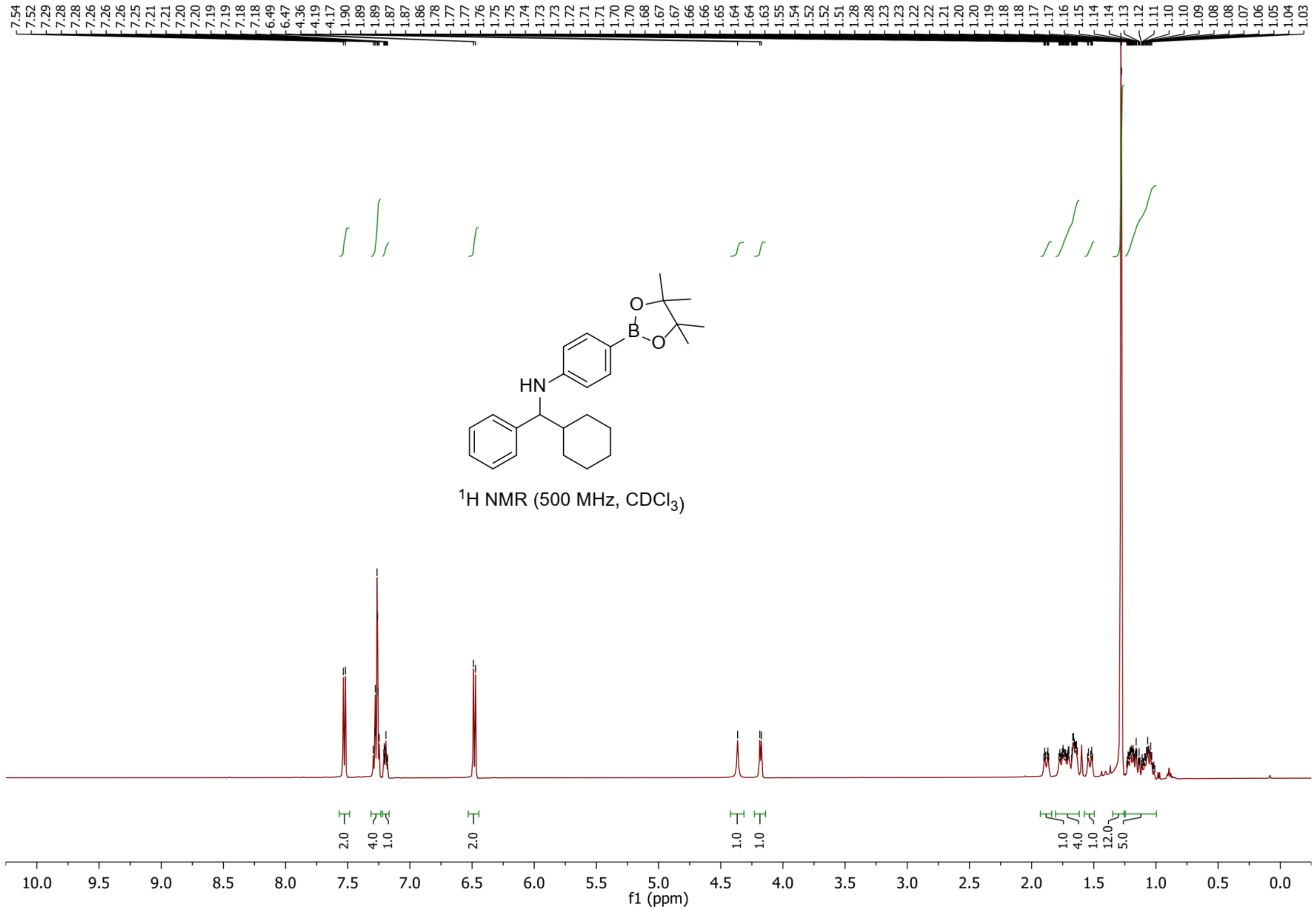

Go to table of contents

_N_-(Cyclohexyl(phenyl)methyl)-4-(4,4,5,5-tetramethyl-1,3,2-dioxaborolan-2-yl)aniline (4o)

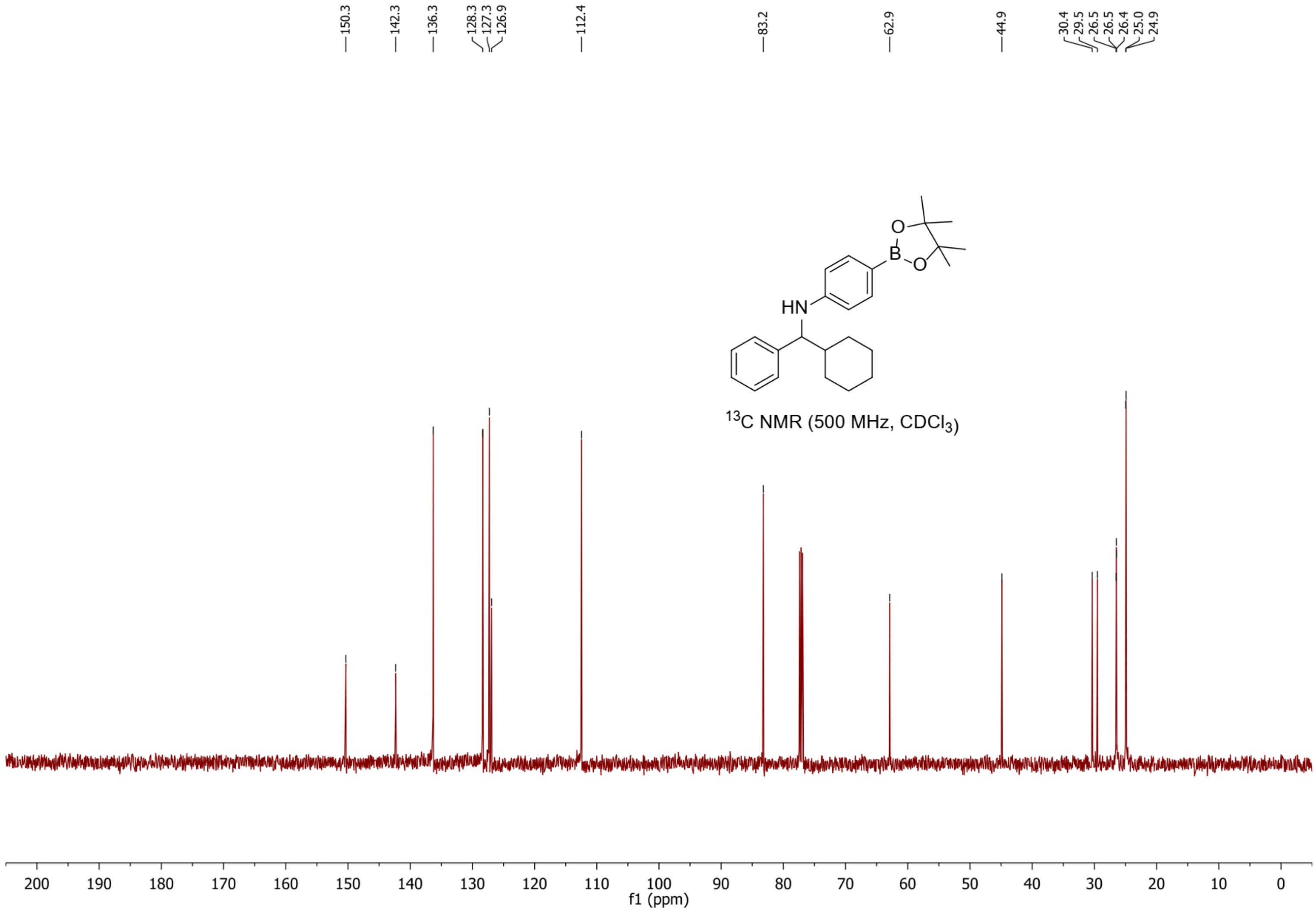

Go to table of contents

_N_-(Cyclohexyl(phenyl)methyl)-2-(4,4,5,5-tetramethyl-1,3,2-dioxaborolan-2-yl)aniline (4p)

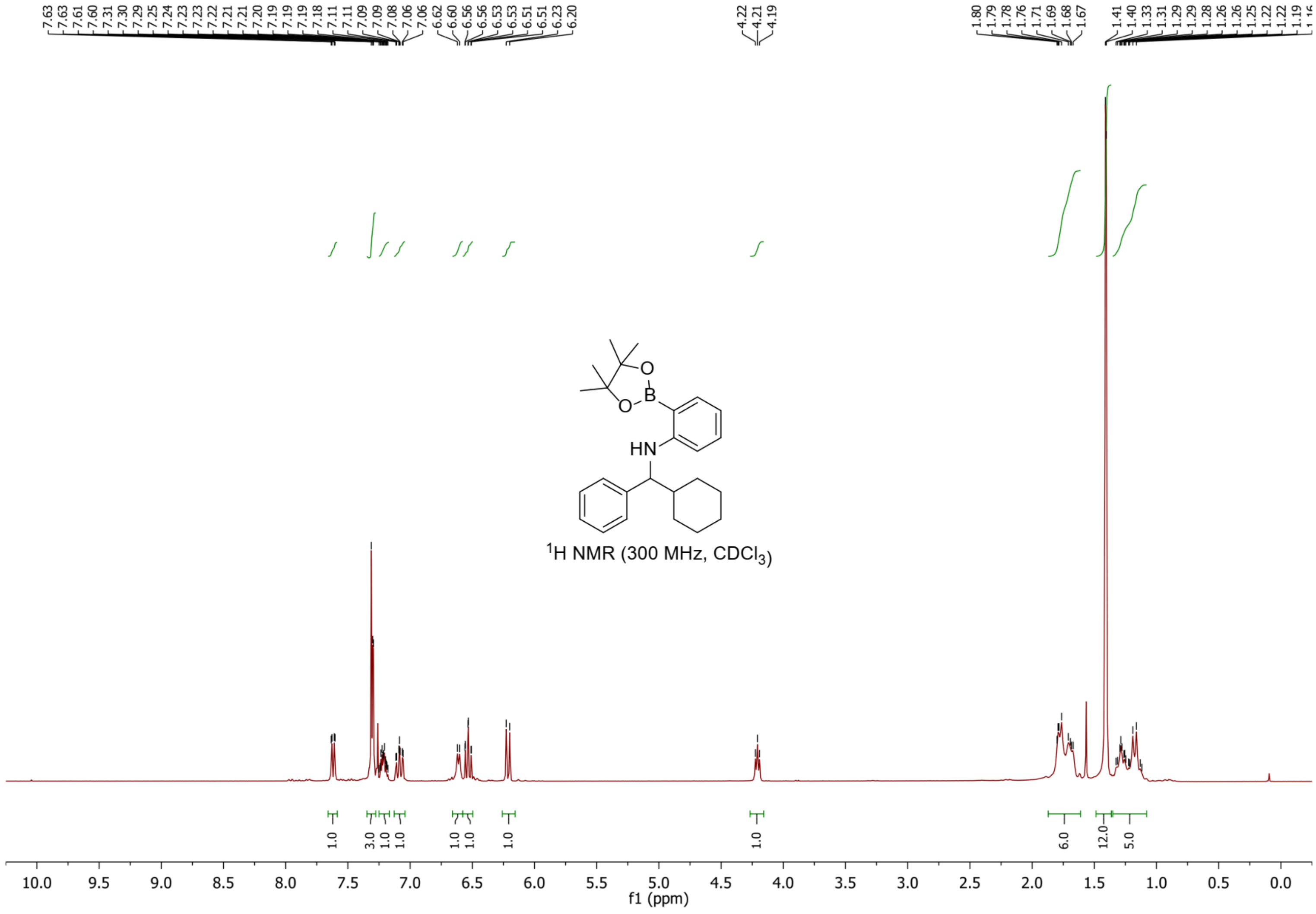

Go to table of contents

N-(Cyclohexyl(phenyl)methyl)-2-(4,4,5,5-tetramethyl-1,3,2-dioxaborolan-2-yl)aniline (4p)

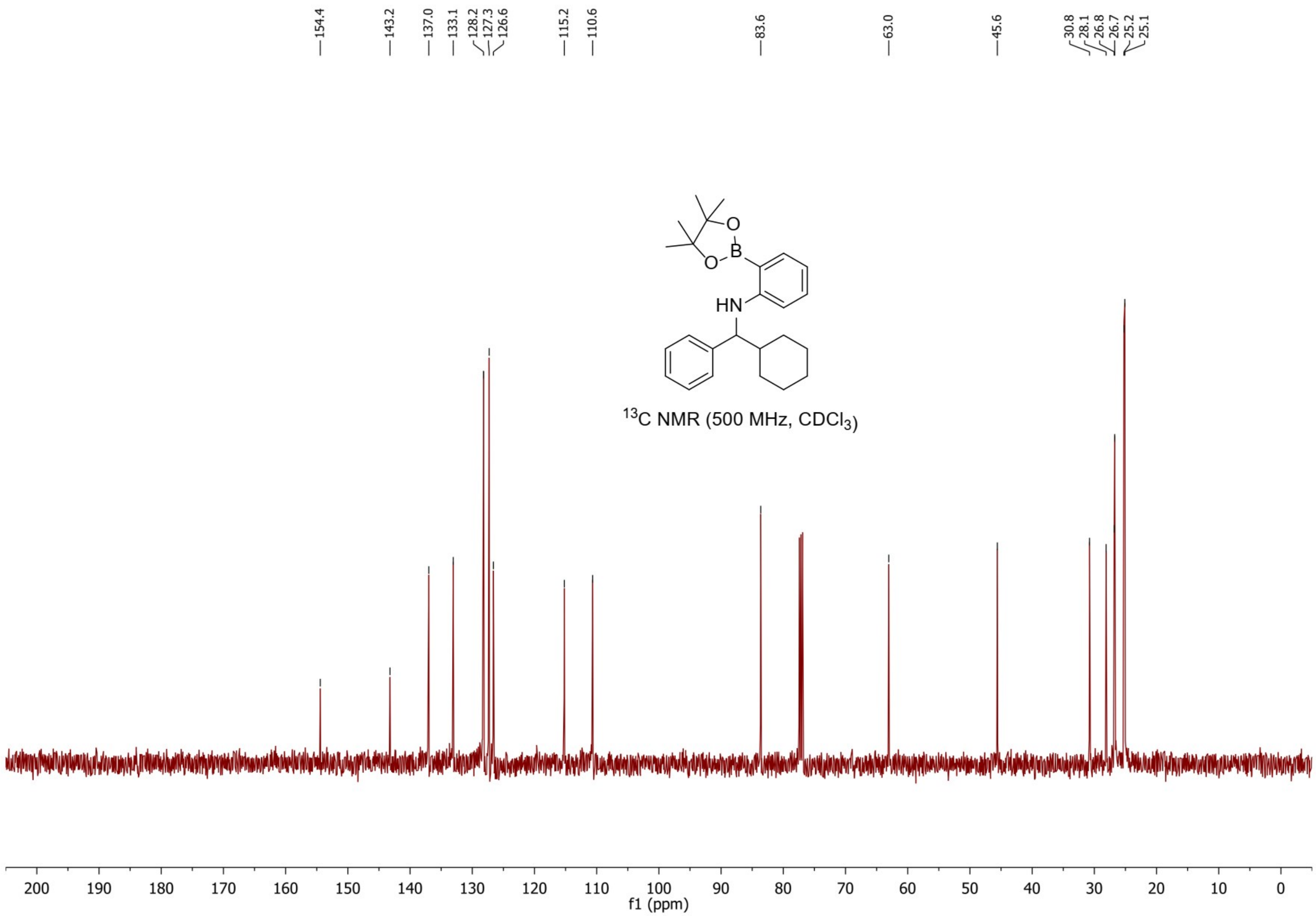

Go to table of contents

N-(Cyclohexyl(phenyl)methyl)benzo[d]thiazol-6-amine (4q)

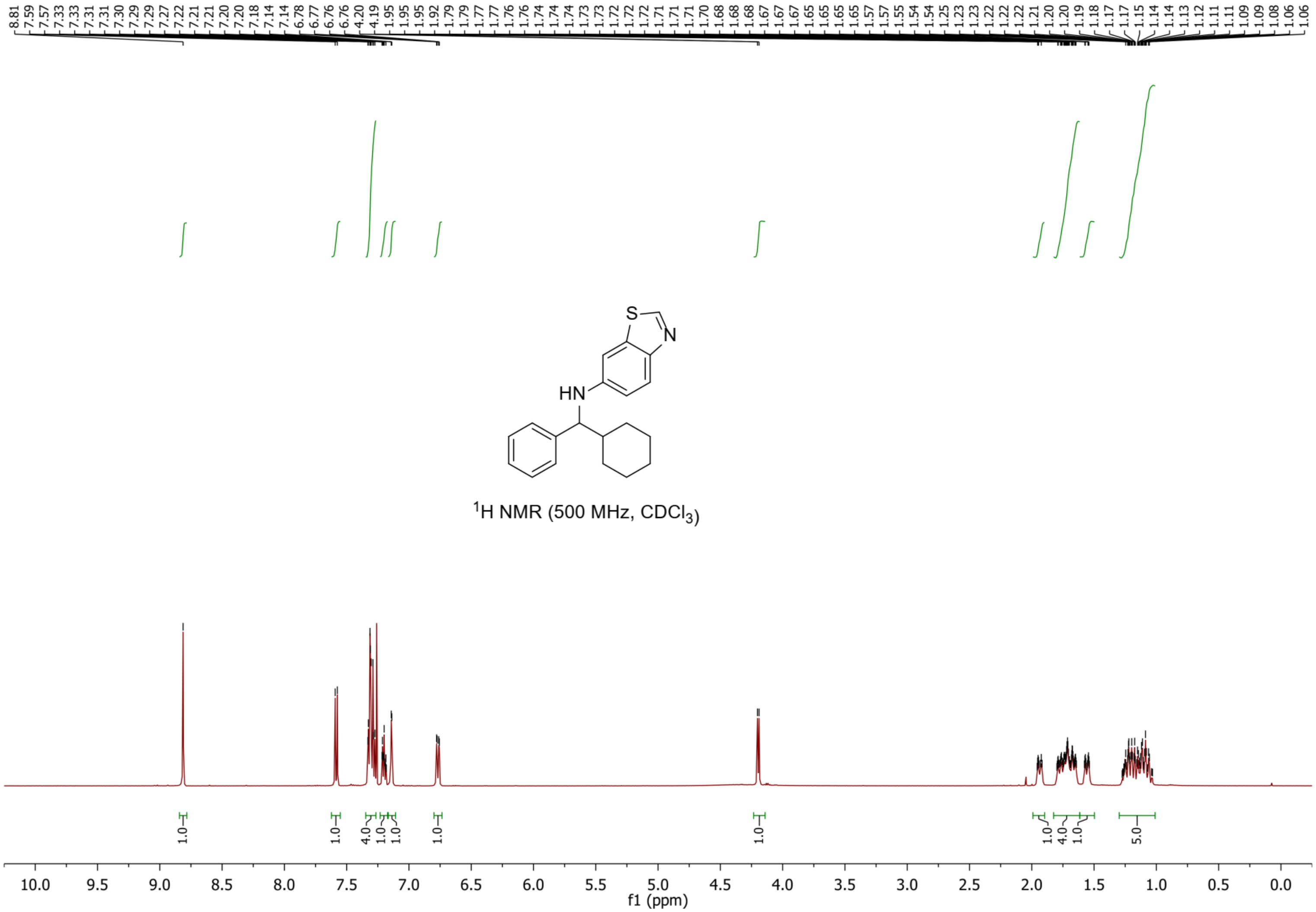

Go to table of contents

N-(Cyclohexyl(phenyl)methyl)benzo[d]thiazol-6-amine (4q)

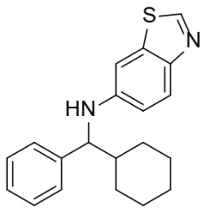

^{13}C NMR (500 MHz, $CDCl_3$)

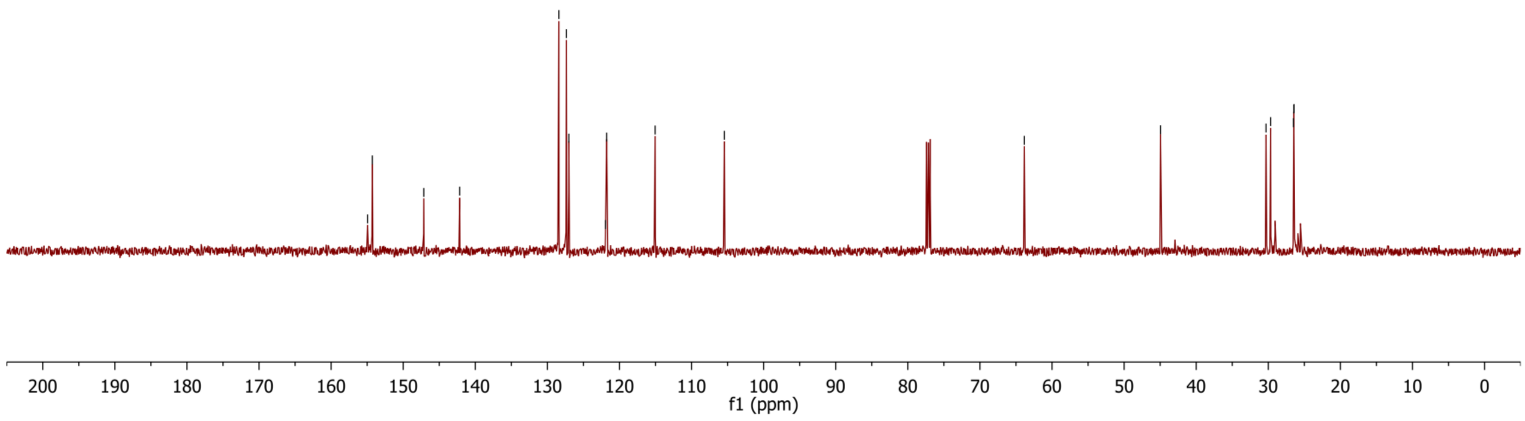

Go to table of contents

N-(Cyclohexyl(phenyl)methyl)-3-(oxazol-4-yl)aniline (4r)

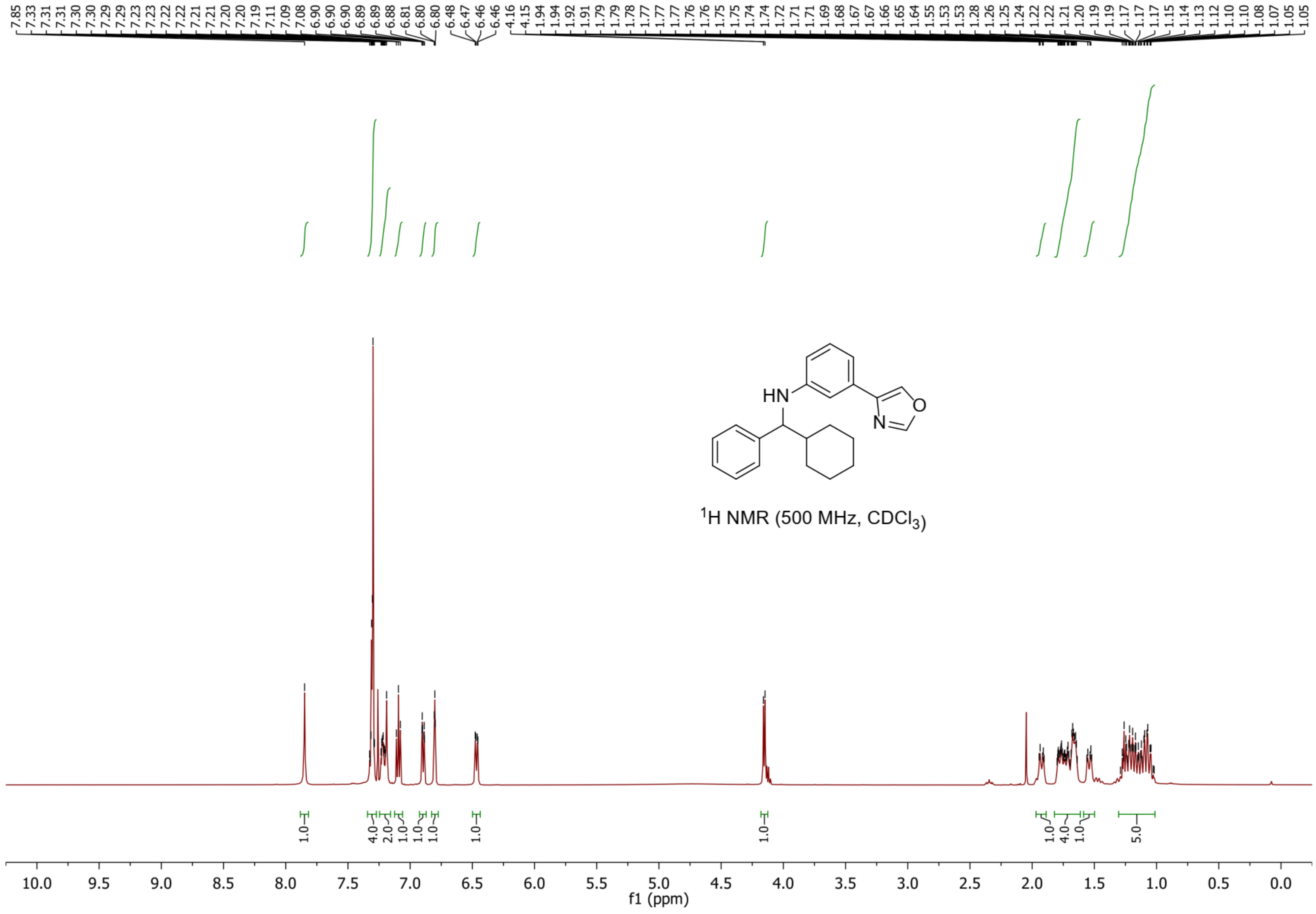

Go to table of contents

N-(Cyclohexyl(phenyl)methyl)-3-(oxazol-4-yl)aniline (4r)

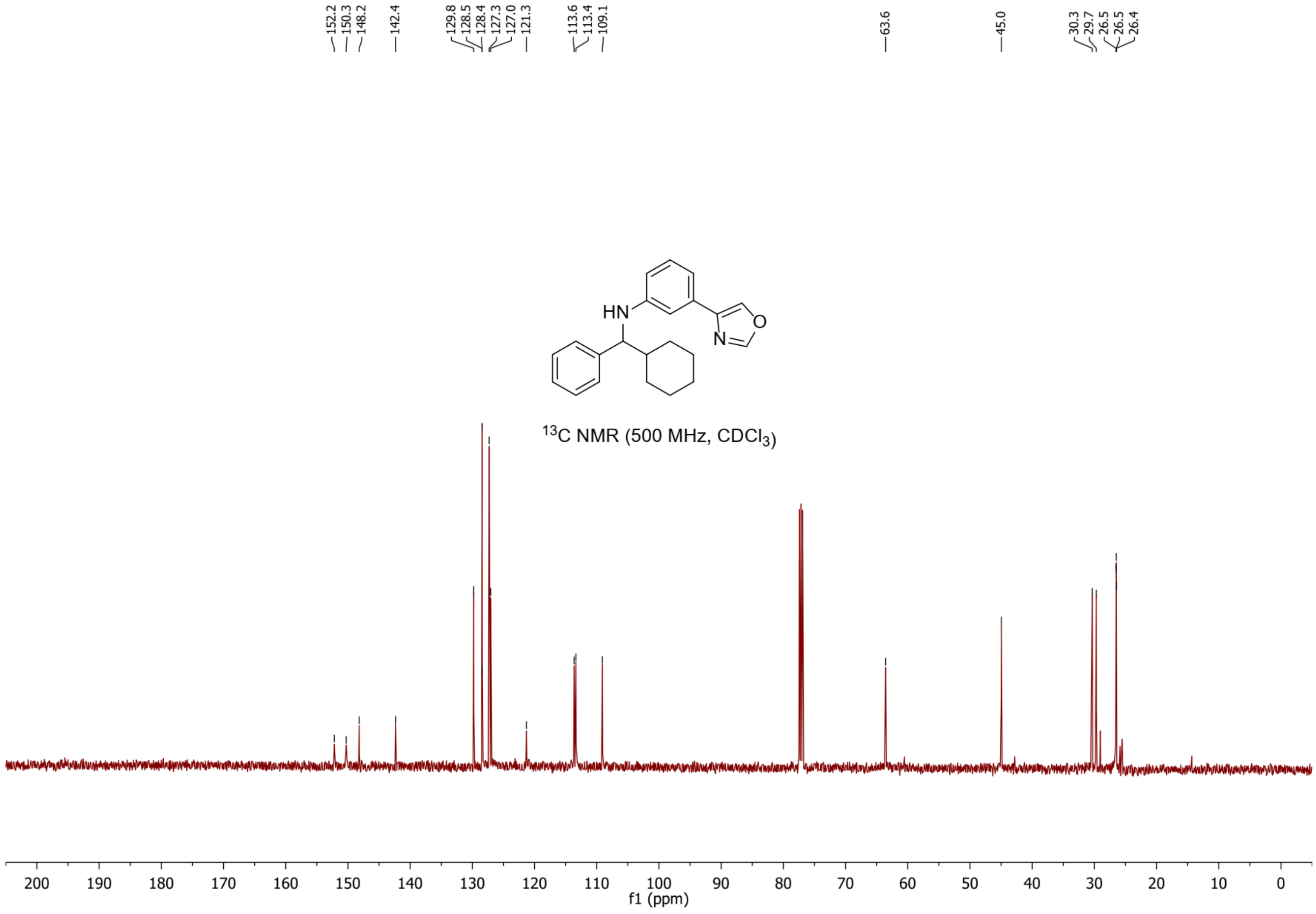

Go to table of contents

N-(cyclohexyl(*o*-tolyl)methyl)aniline (5a)

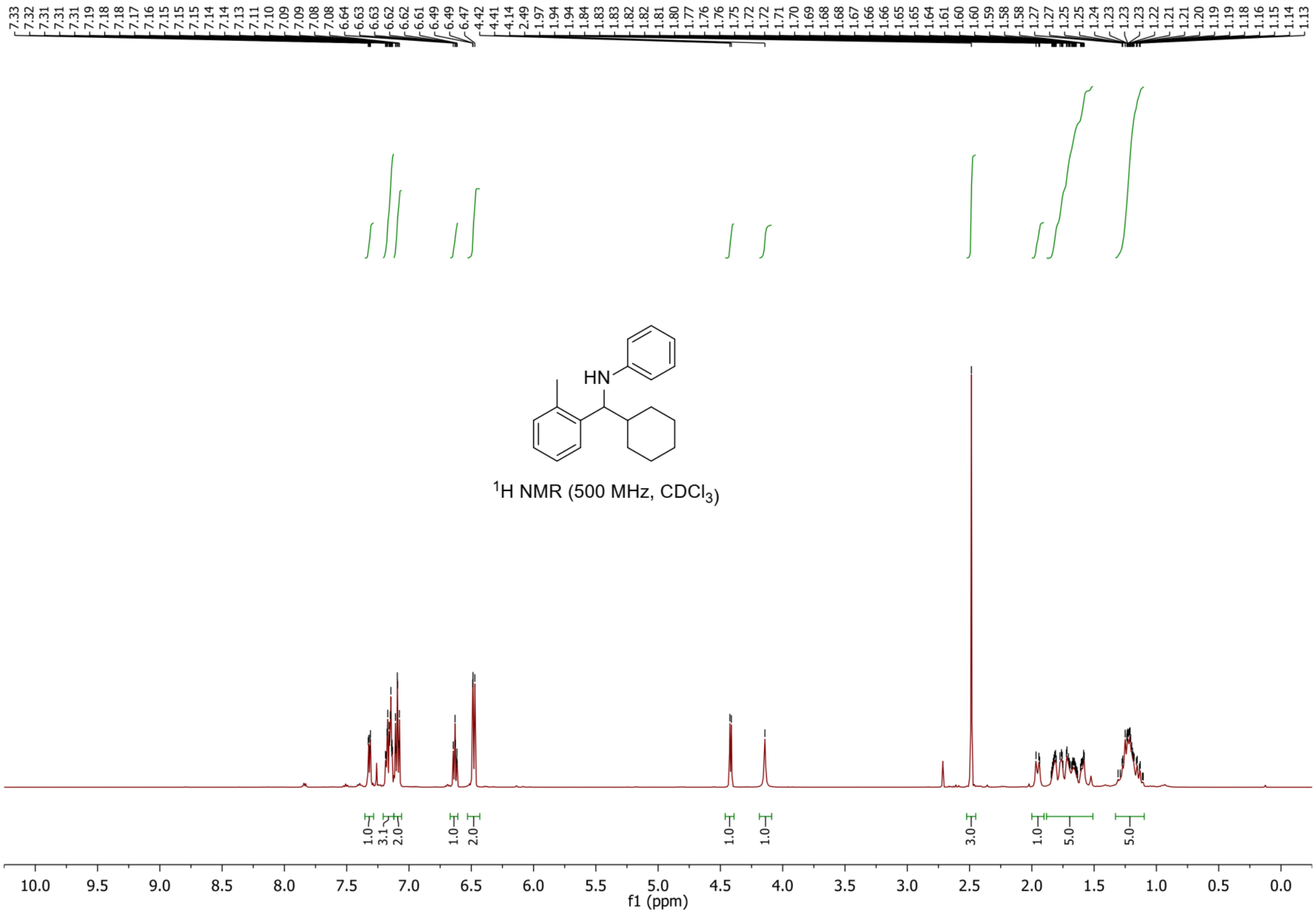

Go to table of contents

_N_-(Cyclohexyl(_o_-tolyl)methyl)aniline (5a)

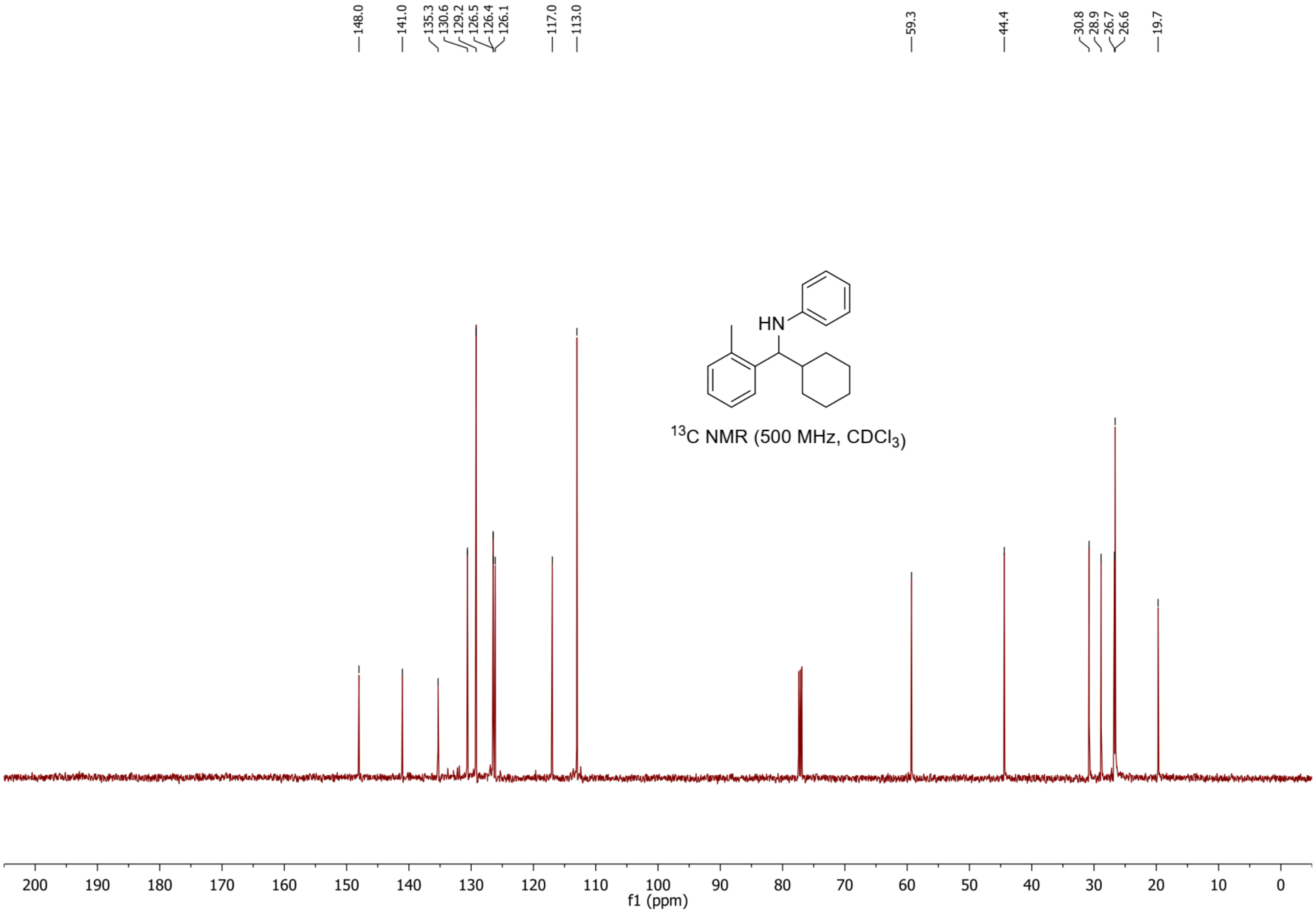

Go to table of contents

N-(Cyclohexyl(m-tolyl)methyl)aniline (5b)

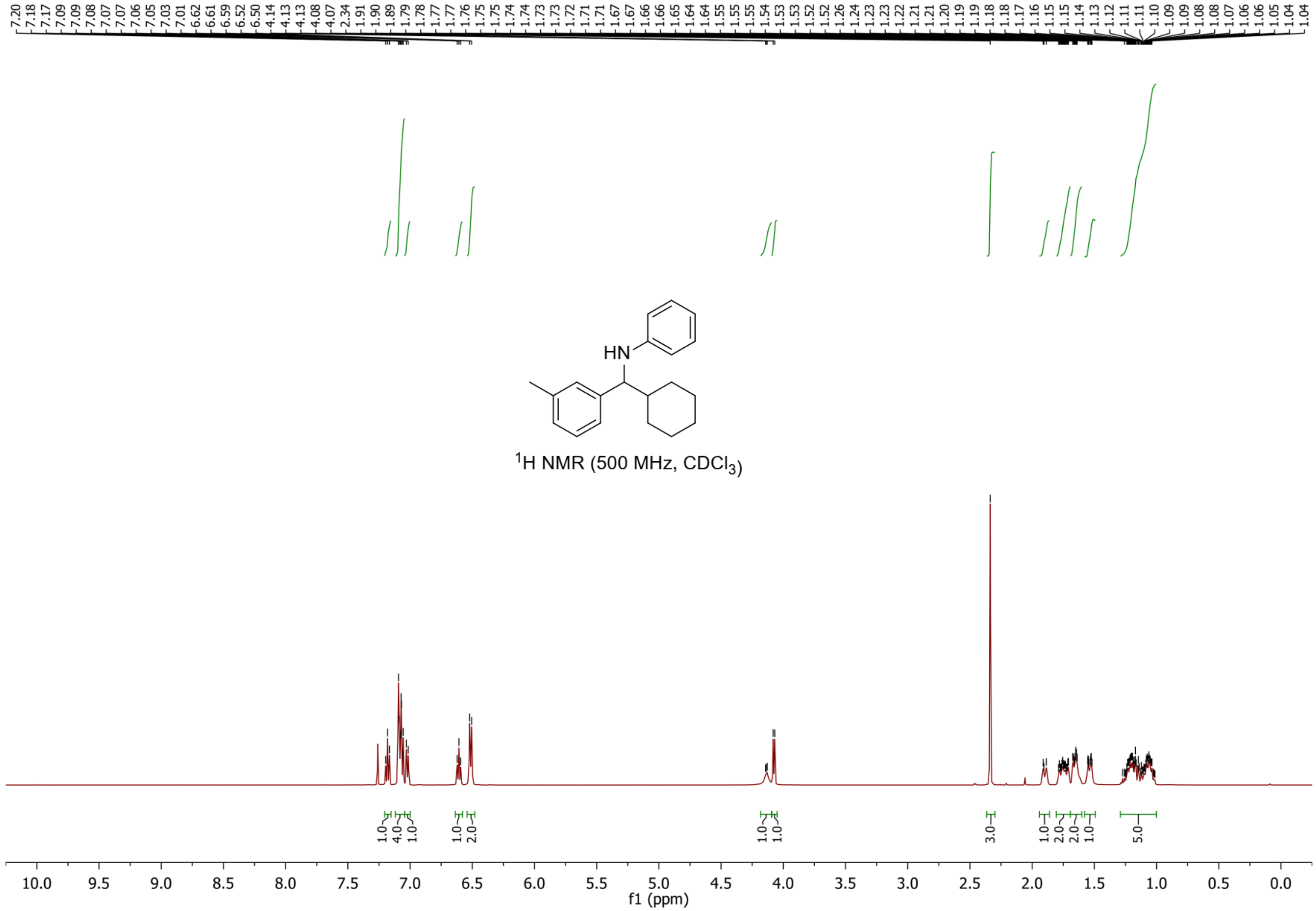

Go to table of contents

N-(Cyclohexyl(m-tolyl)methyl)aniline (5b)

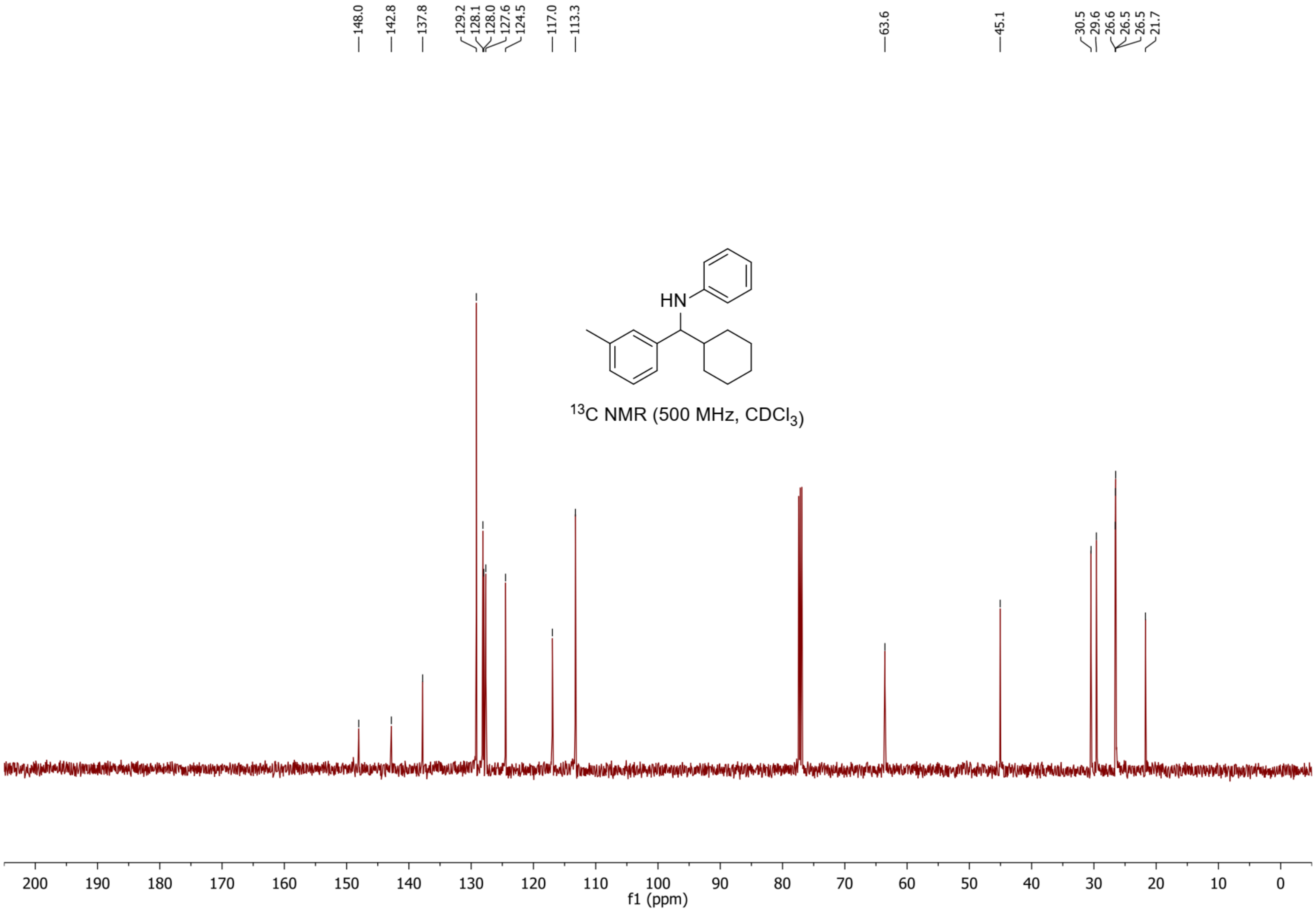

Go to table of contents

N-((4-(tert-butyl)phenyl)(cyclohexyl)methyl)aniline (5c)

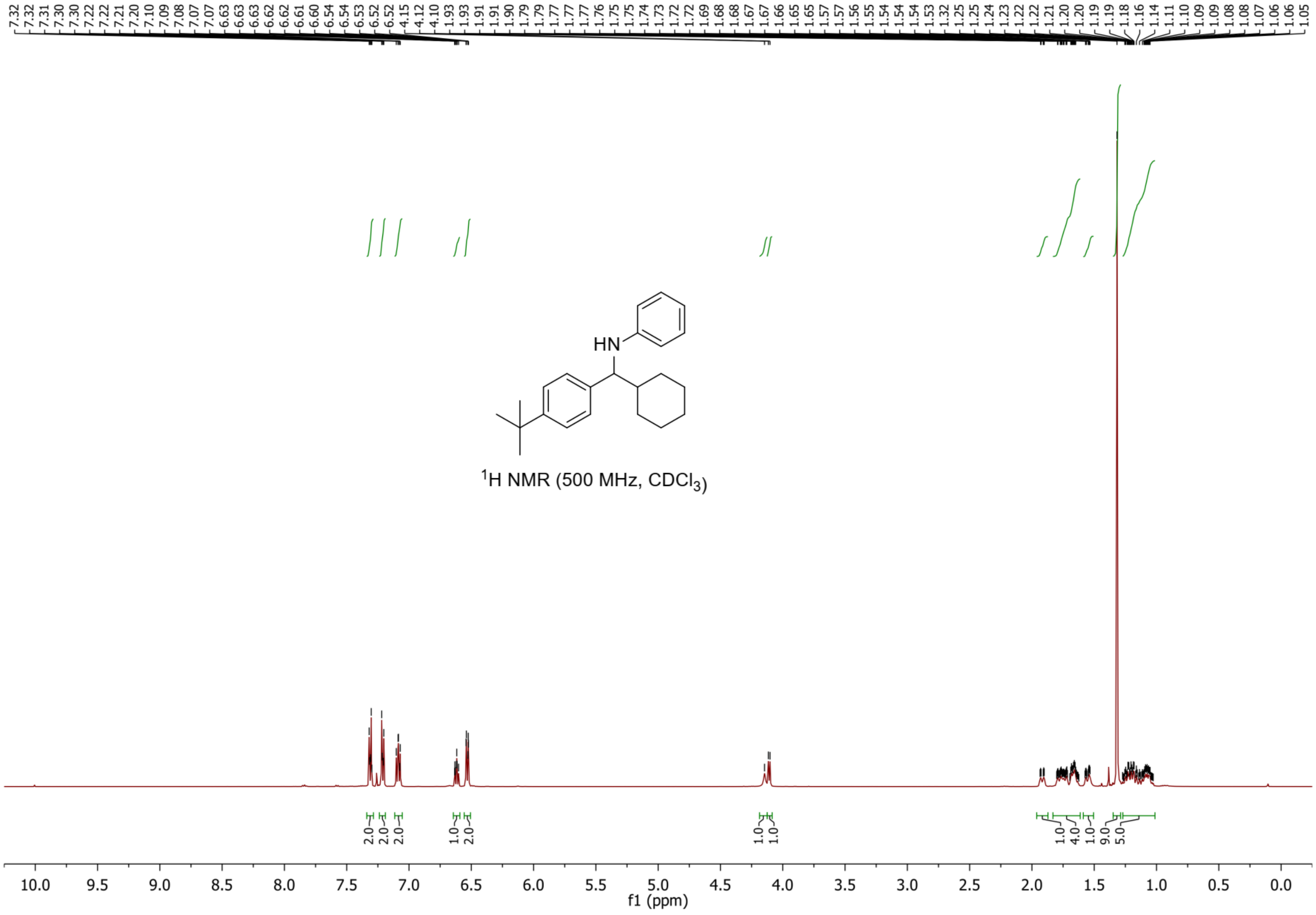

N-((4-(tert-butyl)phenyl)(cyclohexyl)methyl)aniline (5c)

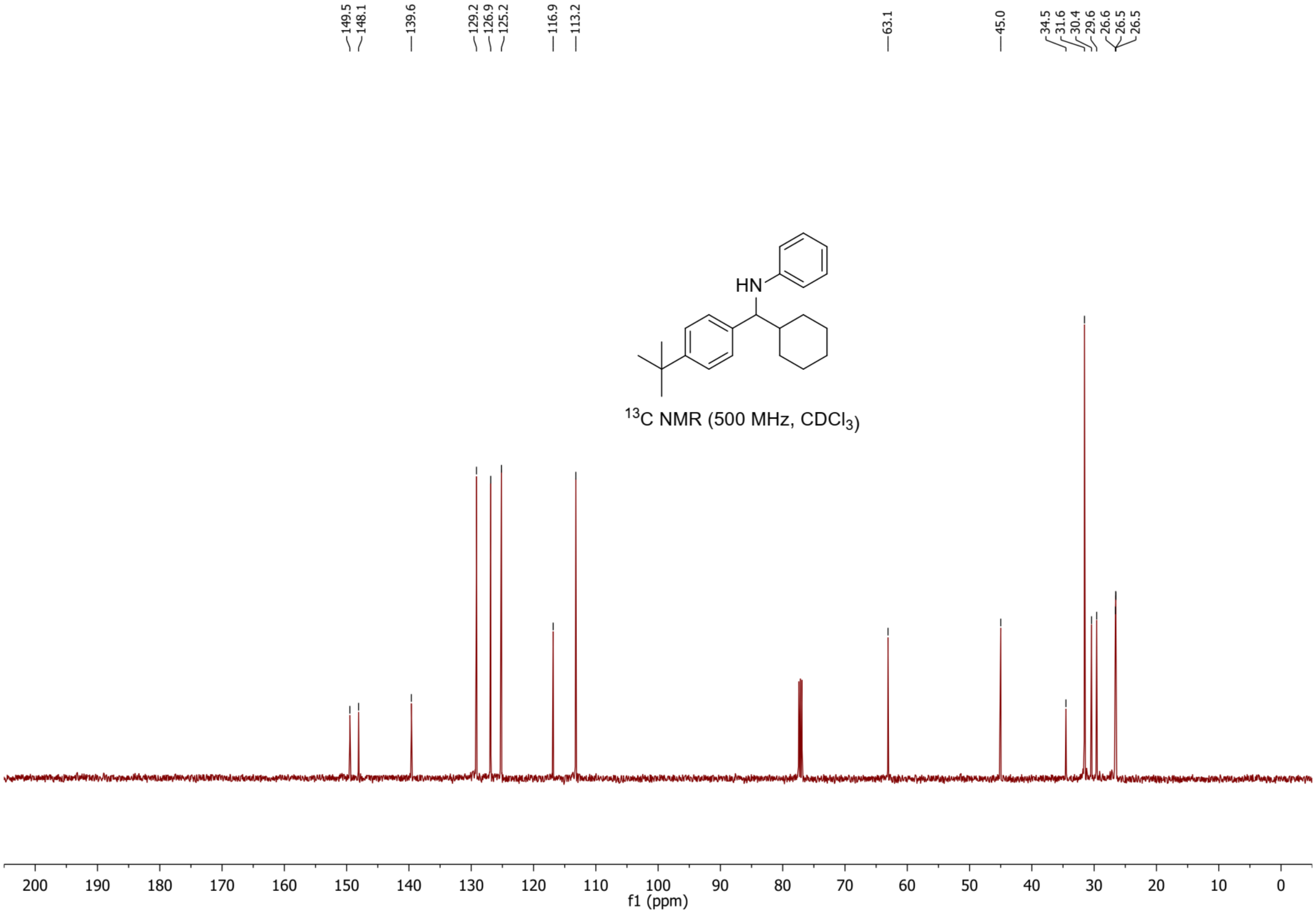

_N_-(Cyclohexyl(2-fluorophenyl)methyl)aniline (5d)

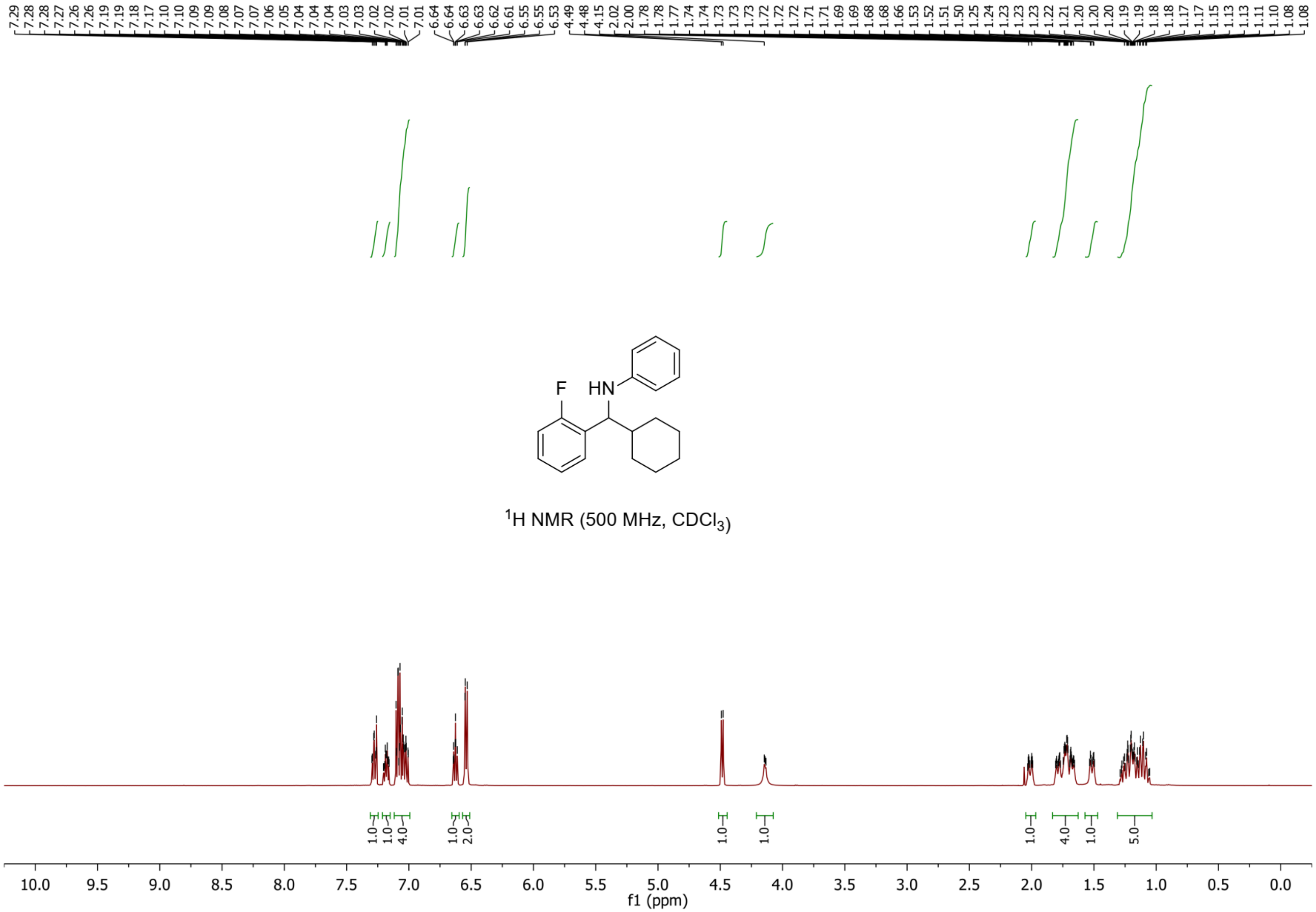

Go to table of contents

N-(Cyclohexyl(2-fluorophenyl)methyl)aniline (5d)

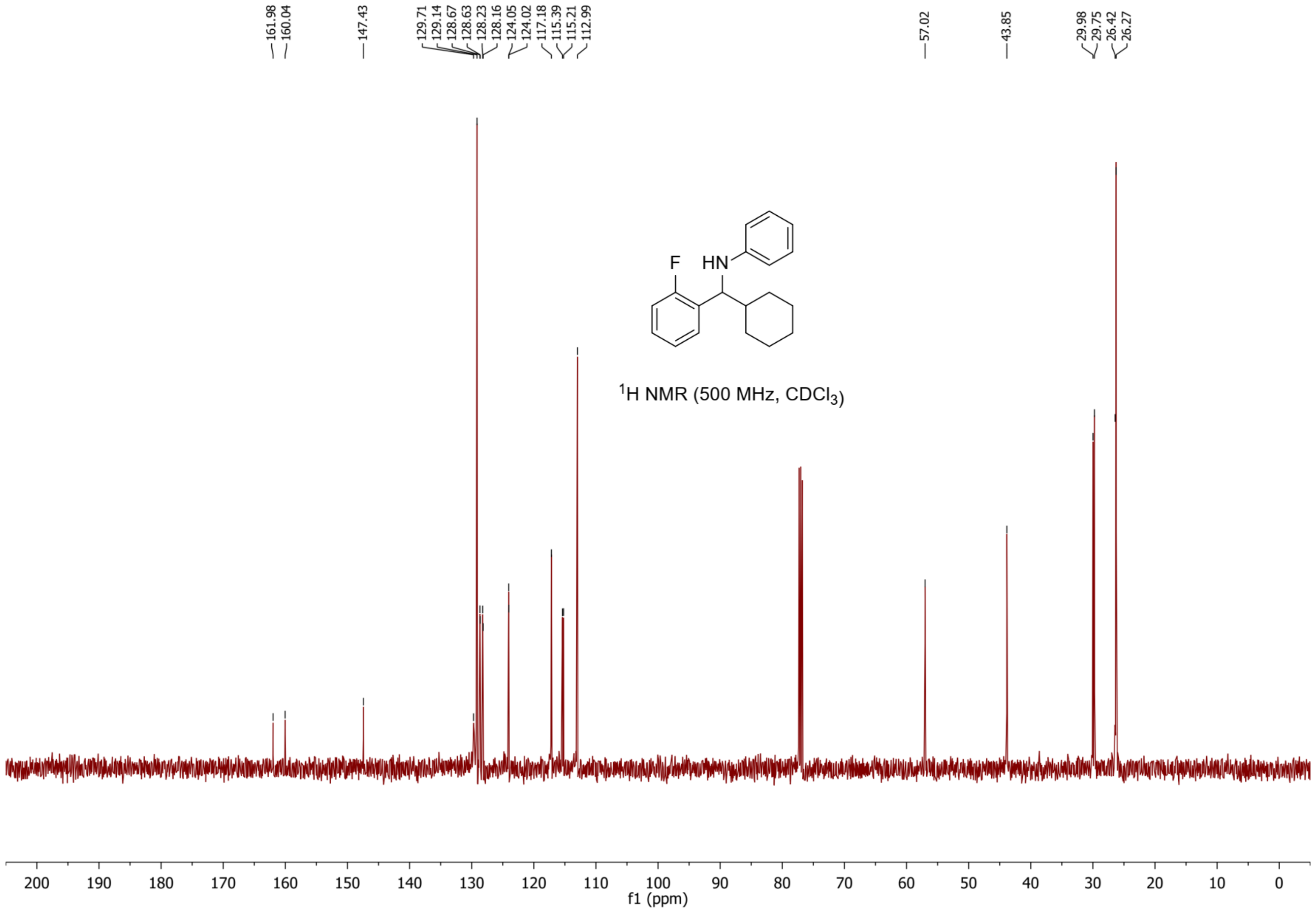

***N*-((4-Chlorophenyl)(cyclohexyl)methyl)aniline (5e)**

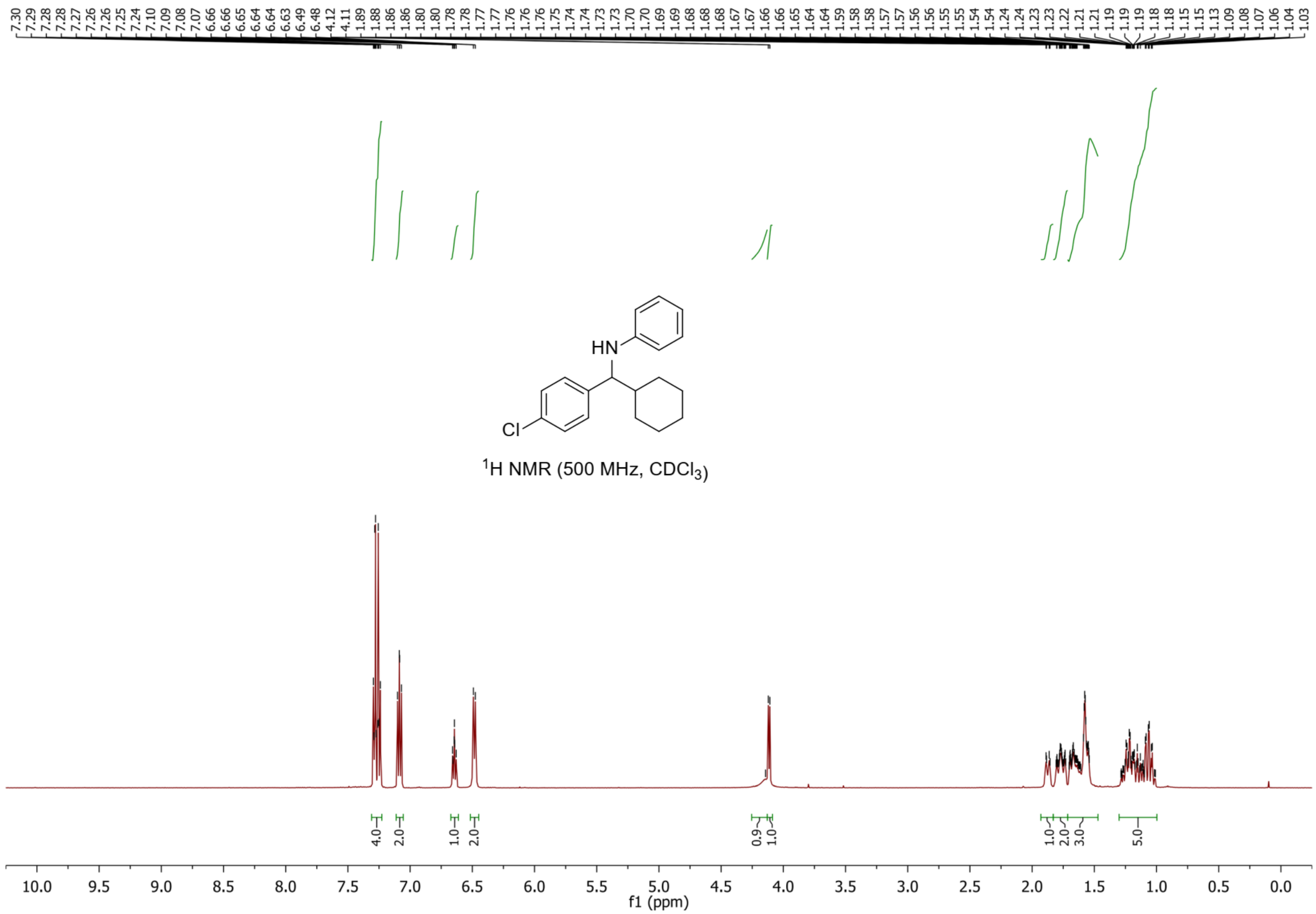

***N*-((4-Chlorophenyl)(cyclohexyl)methyl)aniline (5e)**

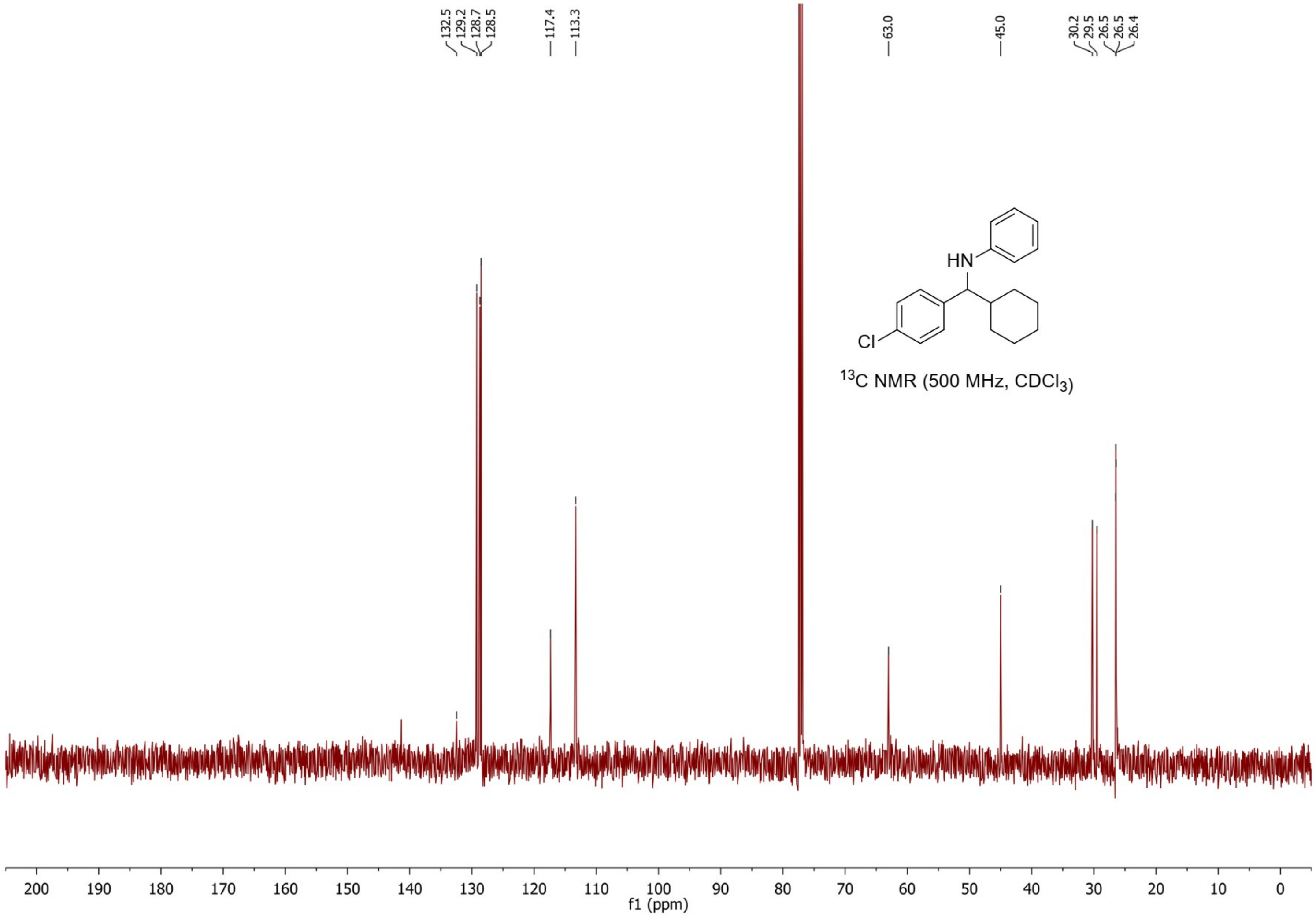

N-(Cyclohexyl(4-methoxyphenyl)methyl)aniline (5f)

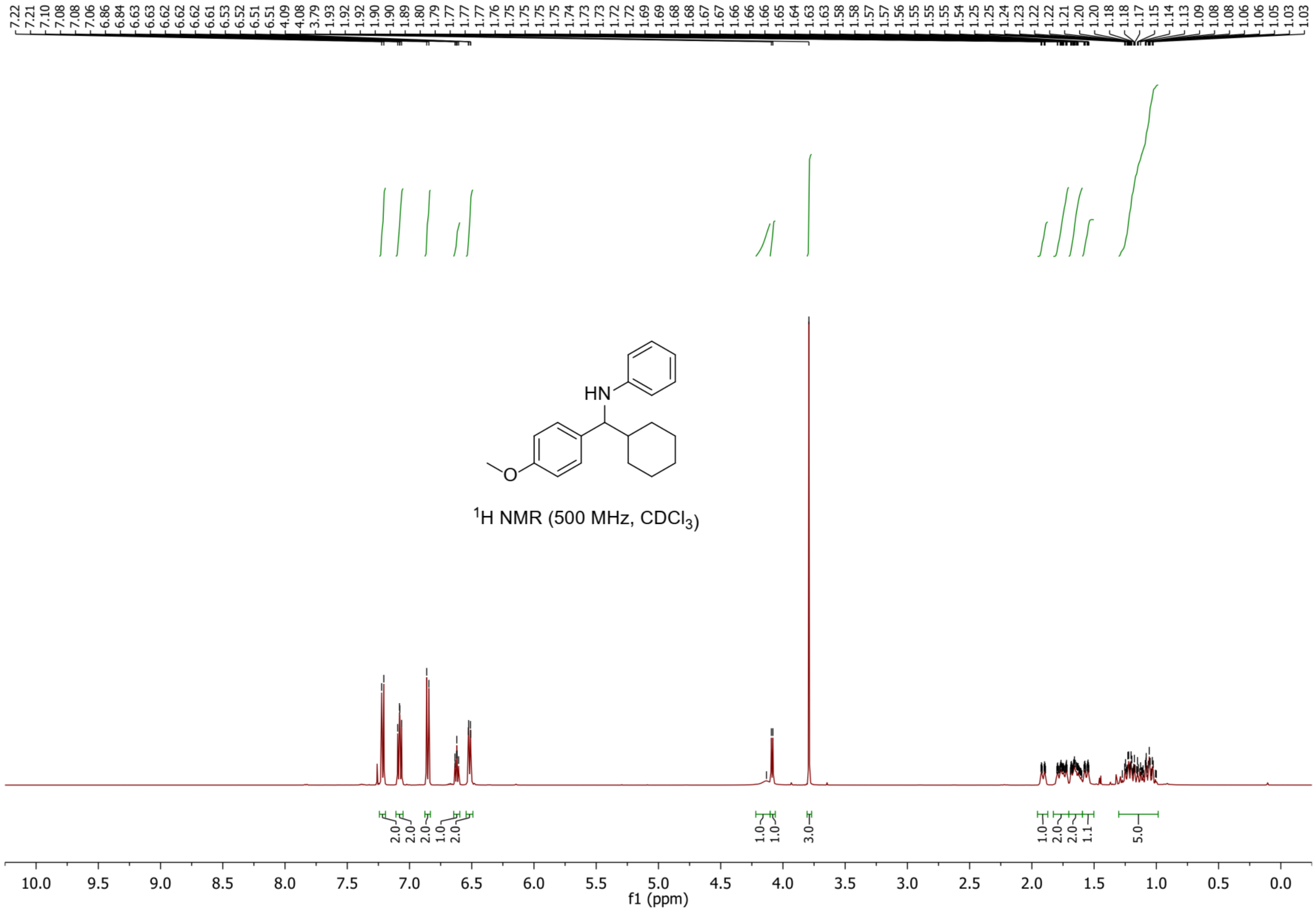

Go to table of contents

N-(Cyclohexyl(4-methoxyphenyl)methyl)aniline (5f)

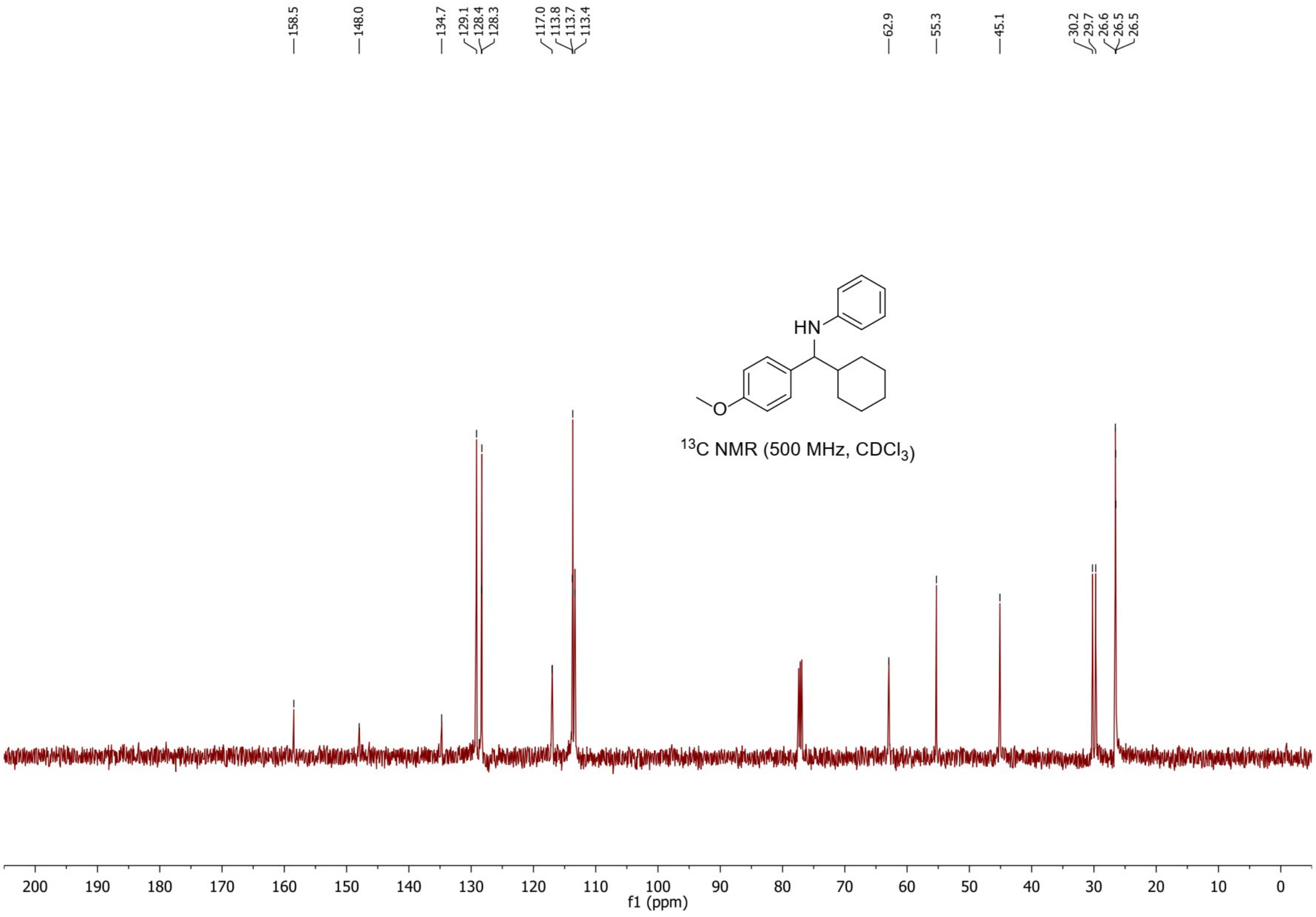

_N_-(cyclohexyl(3,4,5-trimethoxyphenyl)methyl)aniline (5g)

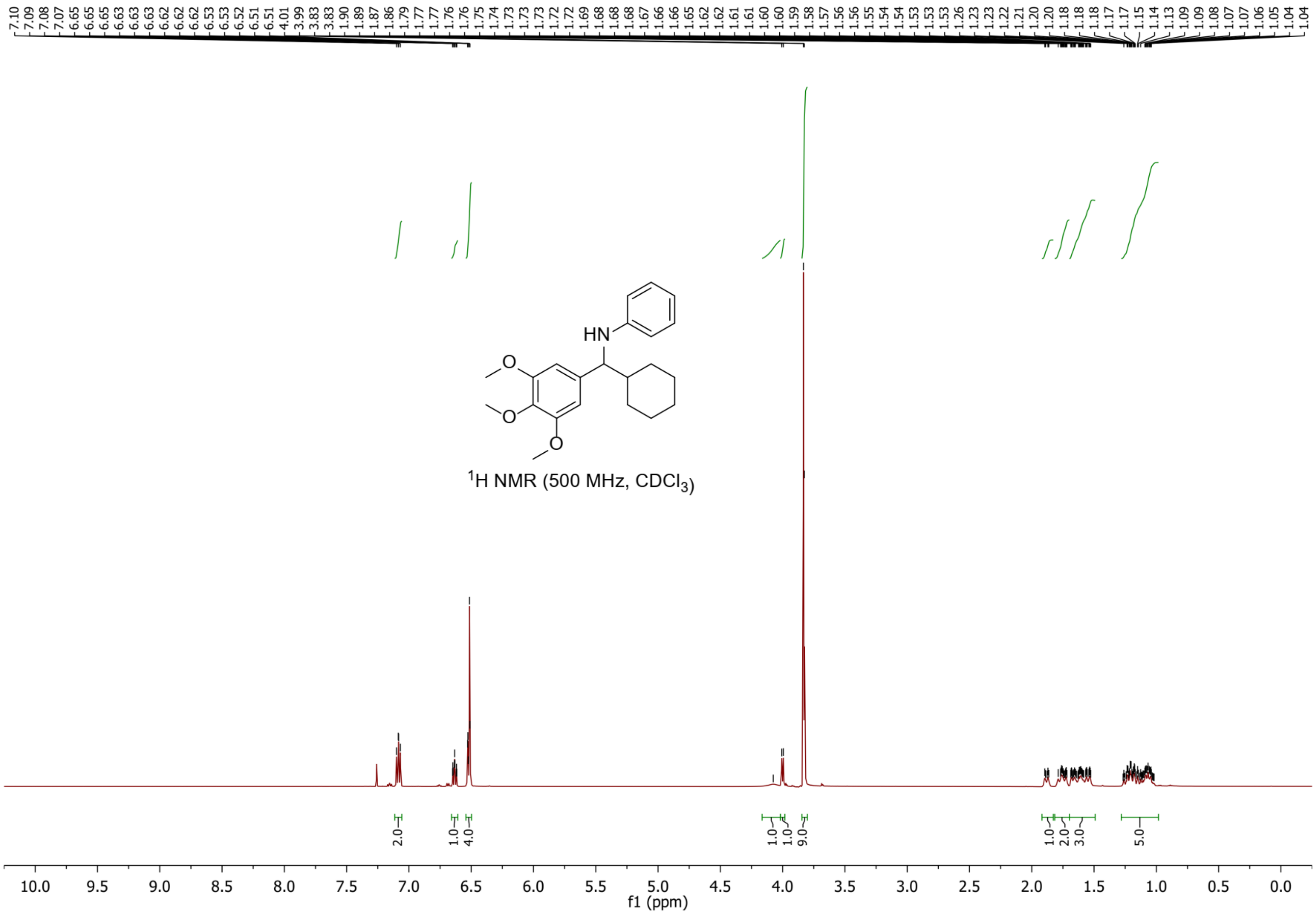

Go to table of contents

N-(cyclohexyl(3,4,5-trimethoxyphenyl)methyl)aniline (5g)

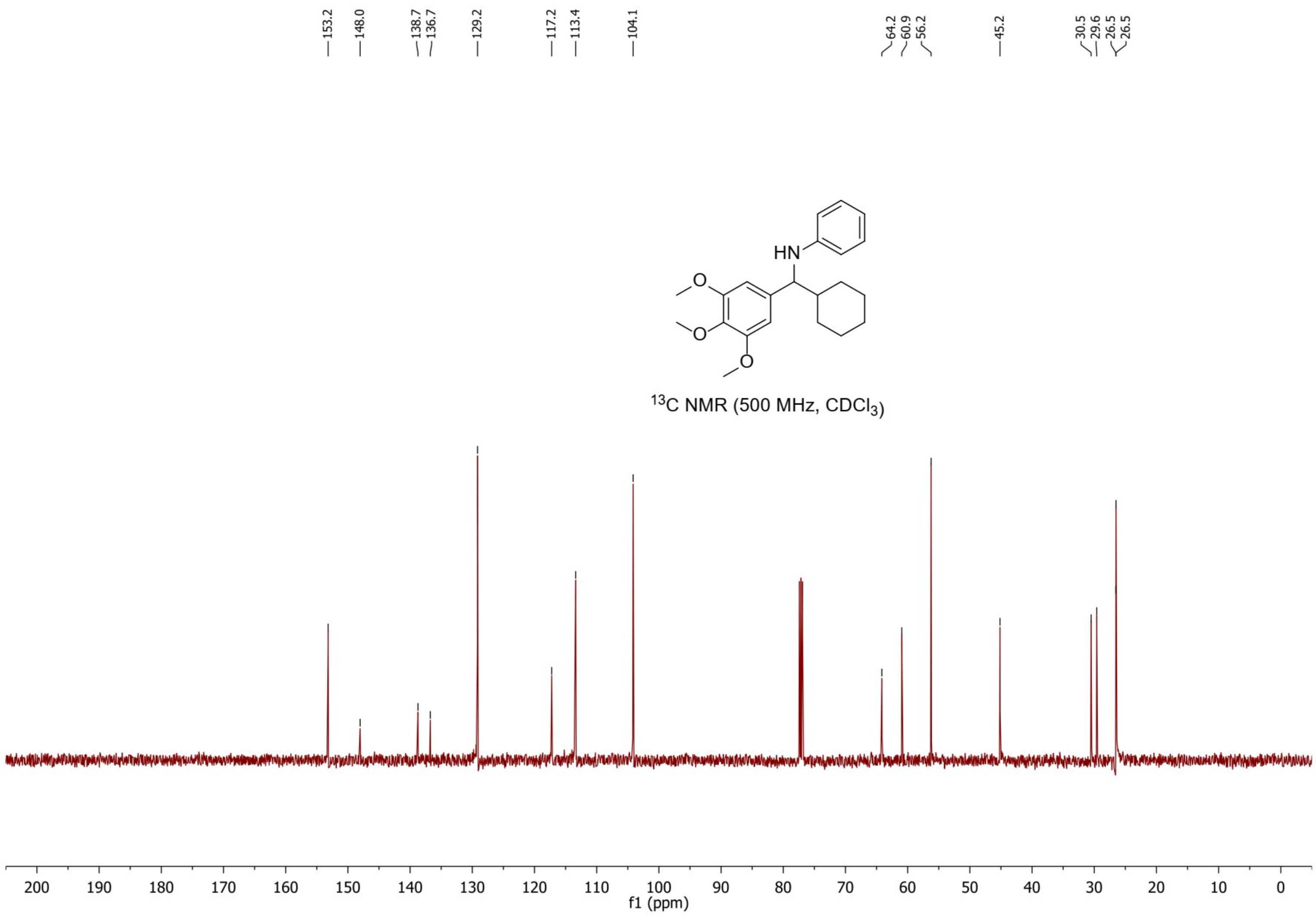

Go to table of contents

N-(4-(Cyclohexyl(phenylamino)methyl)phenyl)acetamide (5h)

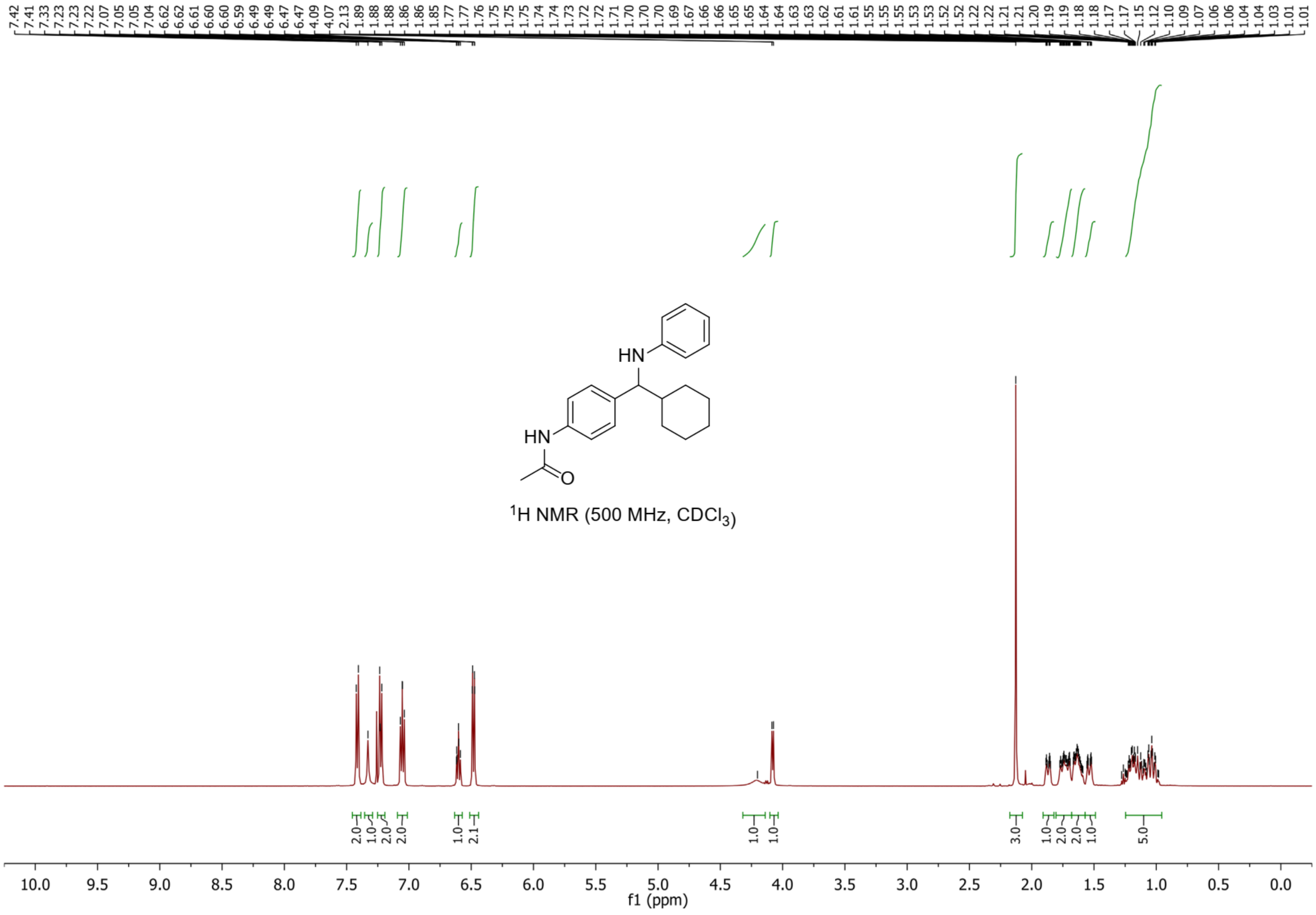

Go to table of contents

N-(4-(Cyclohexyl(phenylamino)methyl)phenyl)acetamide (5h)

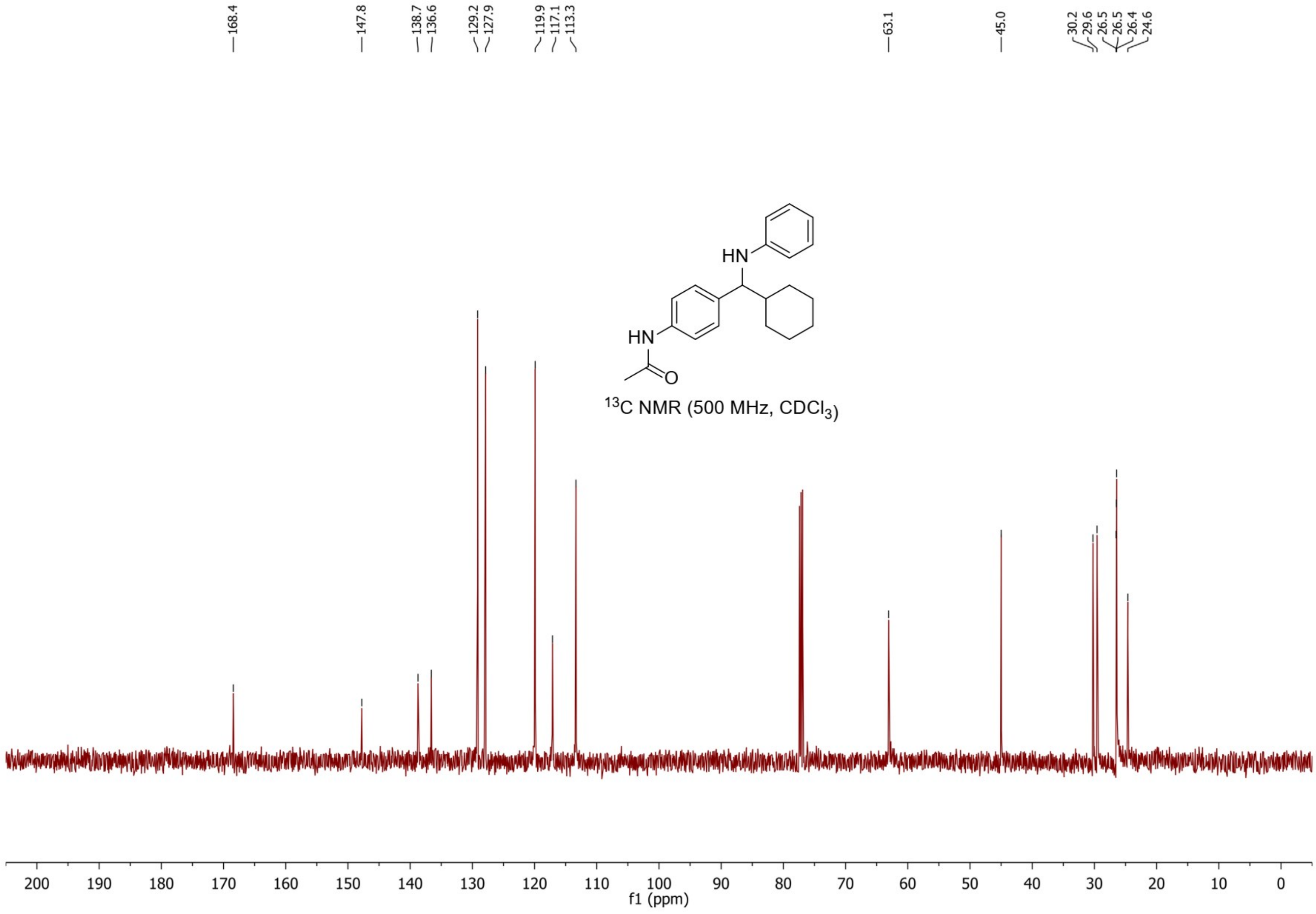

Go to table of contents

_N_-(Cyclohexyl(4-(trifluoromethyl)phenyl)methyl)aniline (5i)

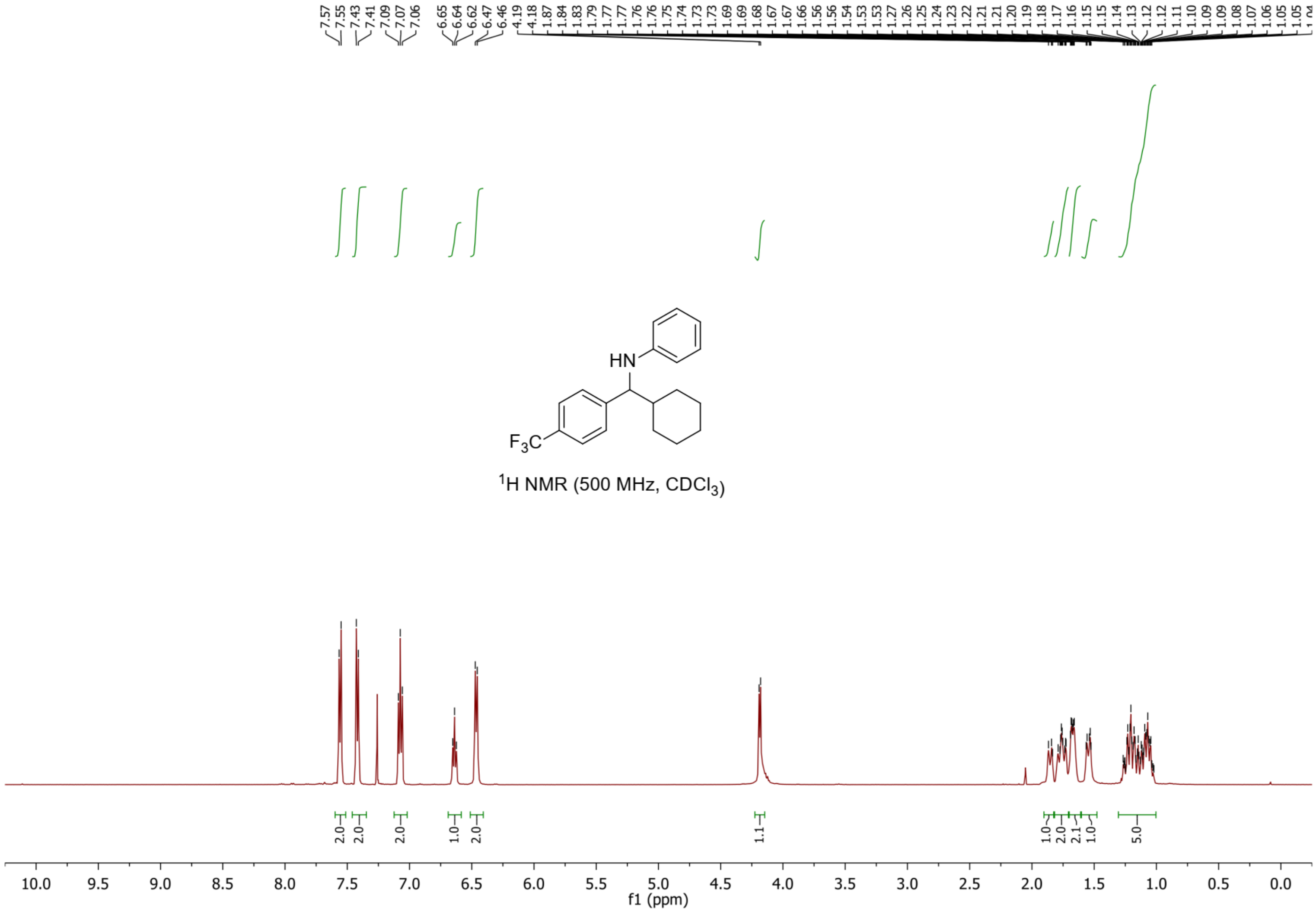

Go to table of contents

N-(Cyclohexyl(4-(trifluoromethyl)phenyl)methyl)aniline (5i)

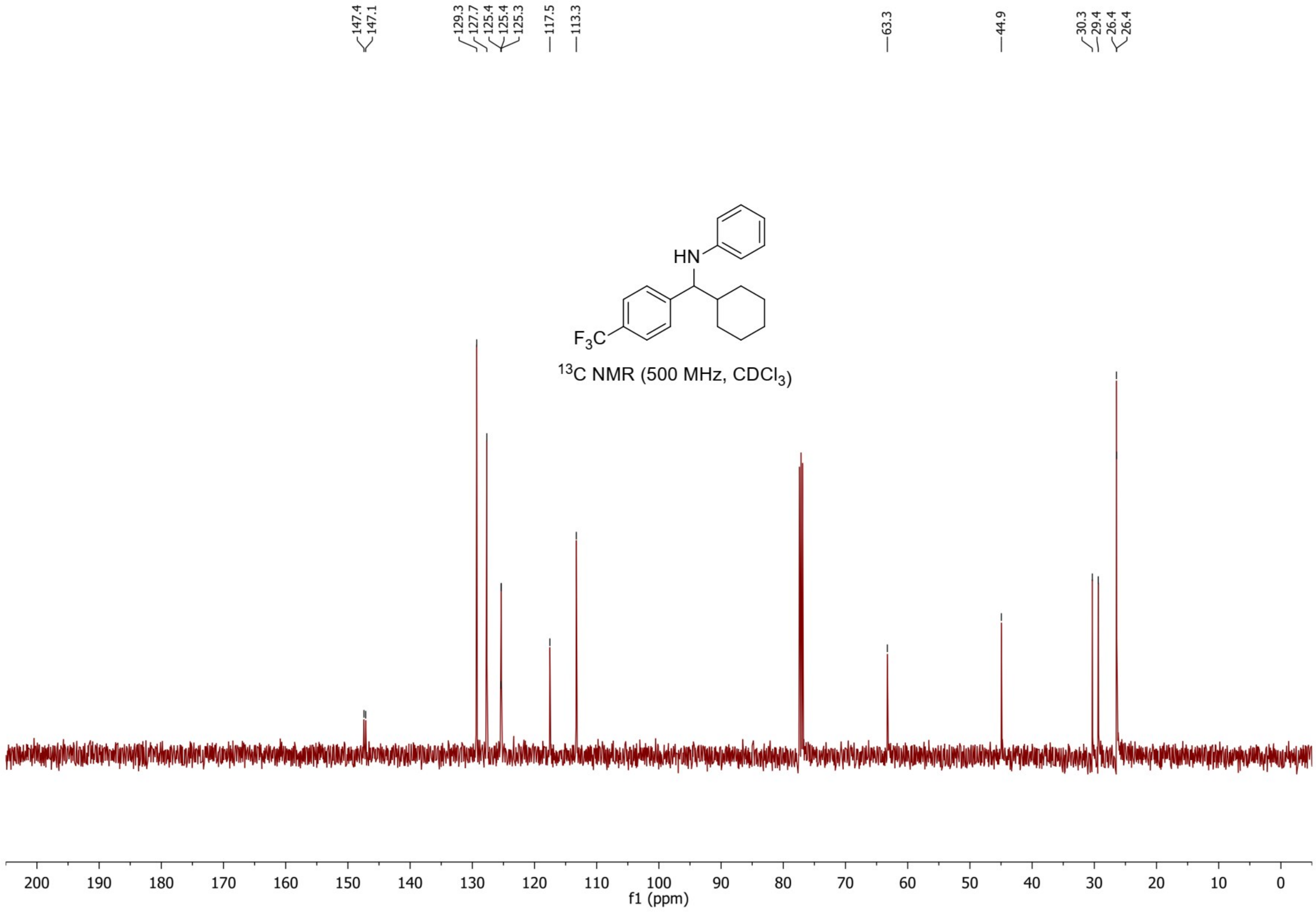

Go to table of contents

N-(Cyclohexyl(naphthalen-2-yl)methyl)aniline (5j)

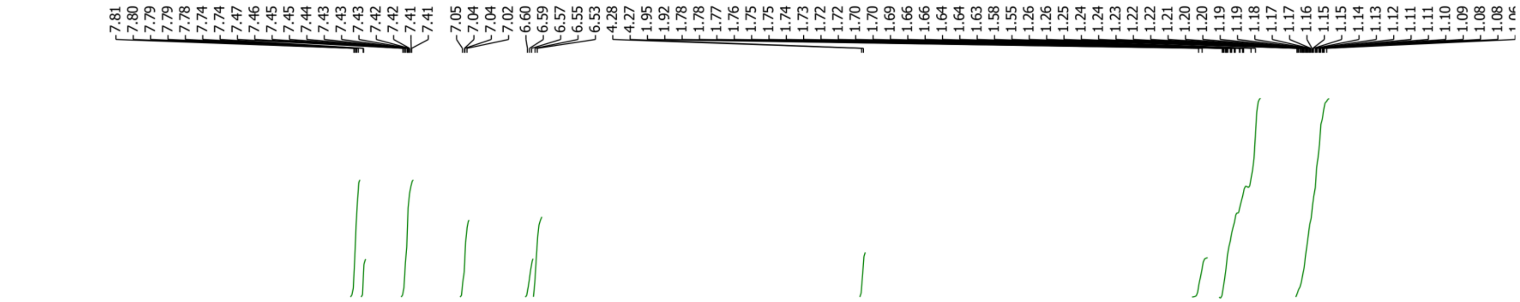

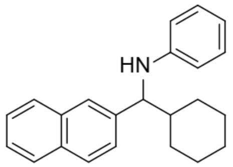

^{1}H NMR (500 MHz, $CDCl_3$)

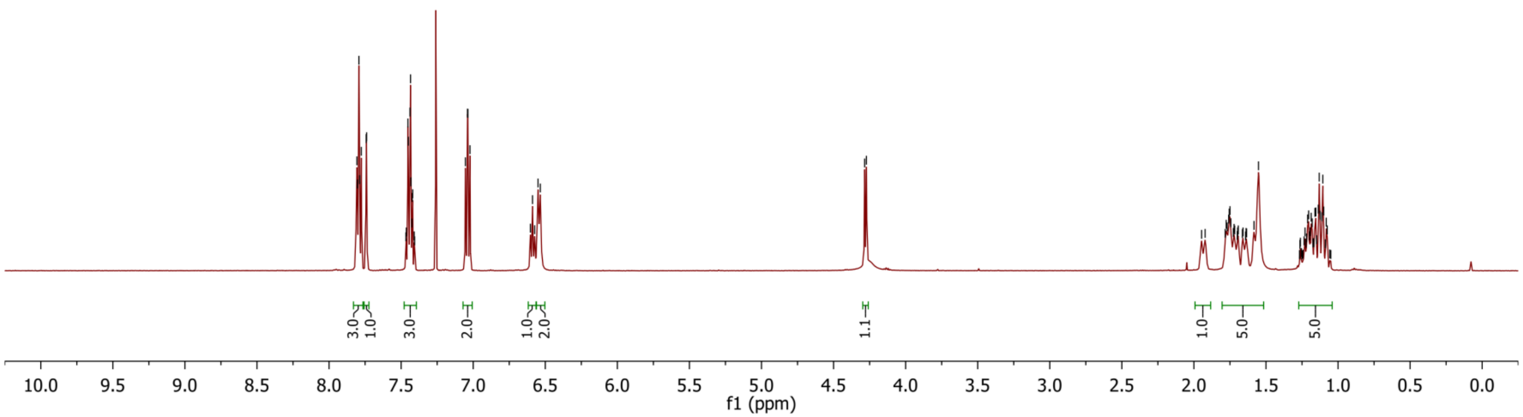

Go to table of contents

N-(Cyclohexyl(naphthalen-2-yl)methyl)aniline (5j)

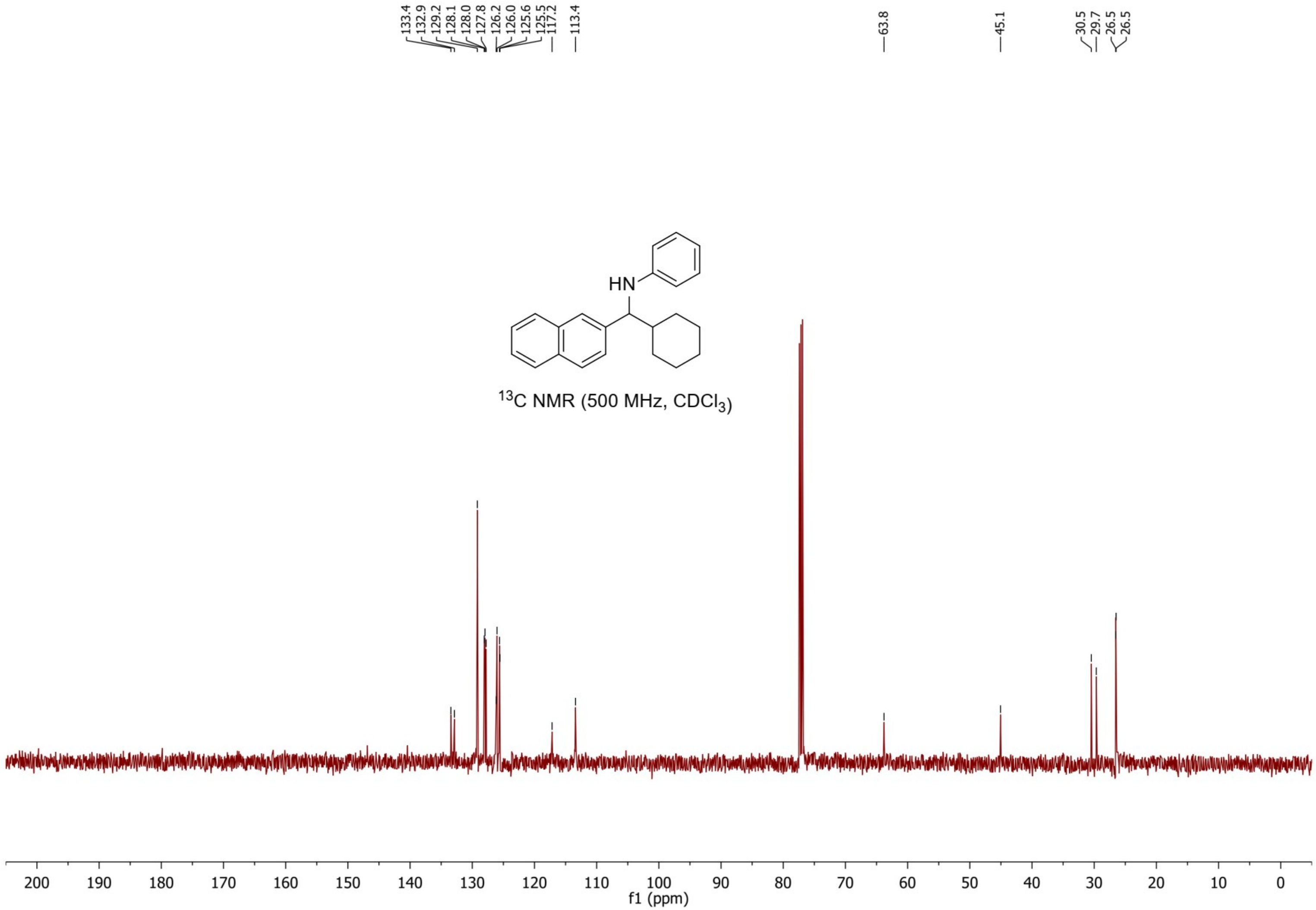

Go to table of contents

N-(Cyclohexyl(naphthalen-1-yl)methyl)aniline (5k)

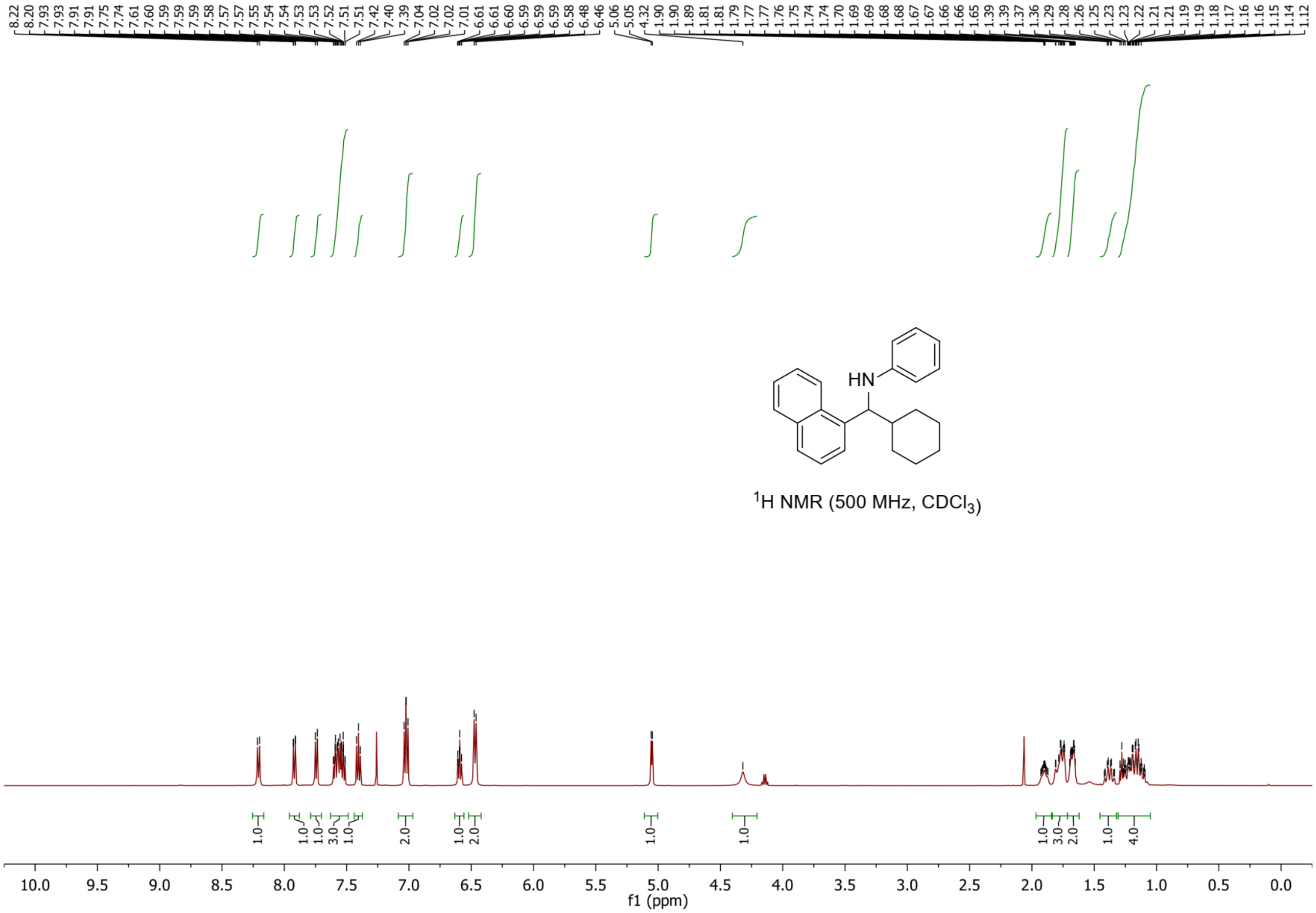

_N_-(Cyclohexyl(naphthalen-1-yl)methyl)aniline (5k)

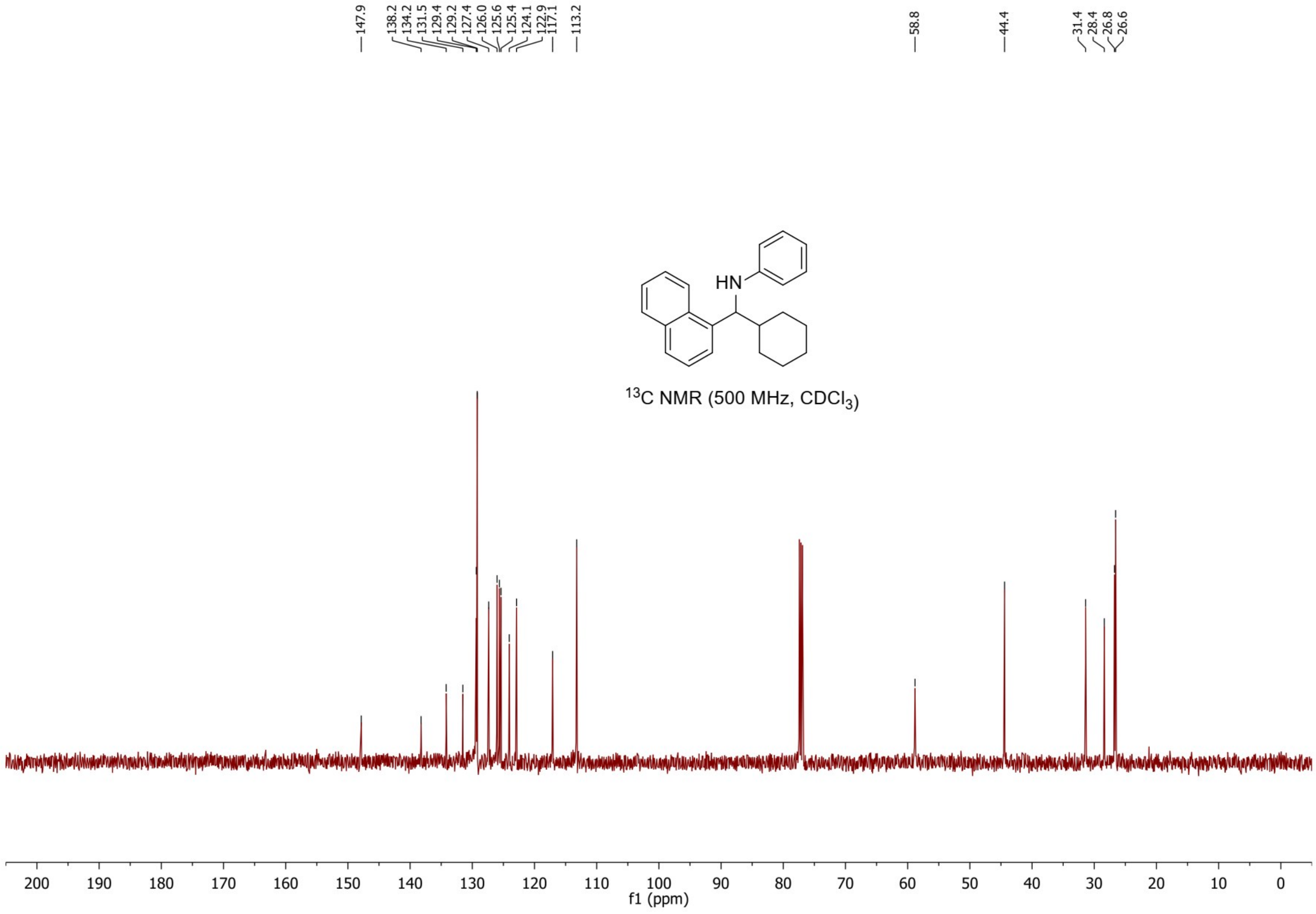

Go to table of contents

N-(Cyclohexyl(pyridin-4-yl)methyl)aniline (5l)

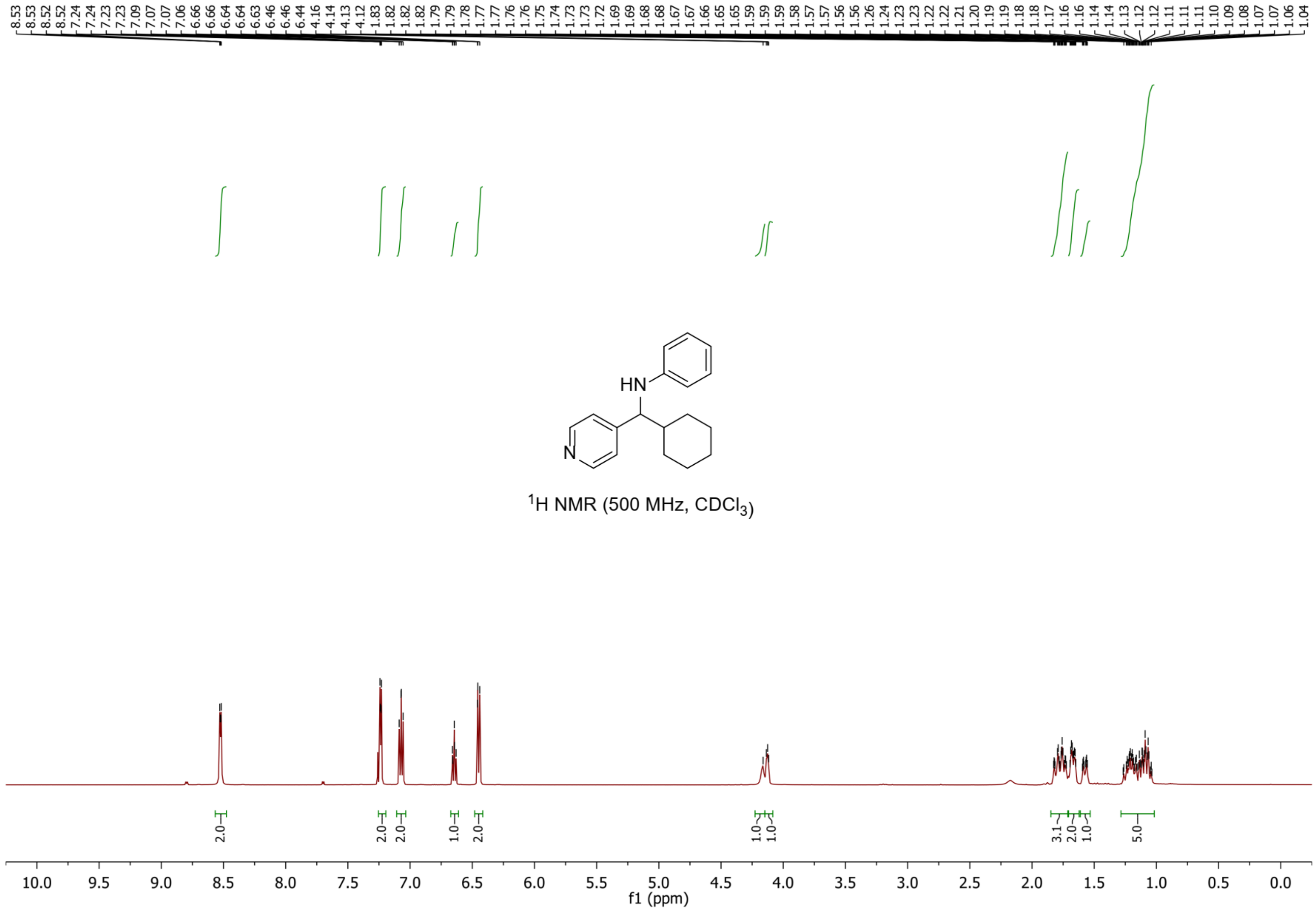

N-(Cyclohexyl(pyridin-4-yl)methyl)aniline (5l)

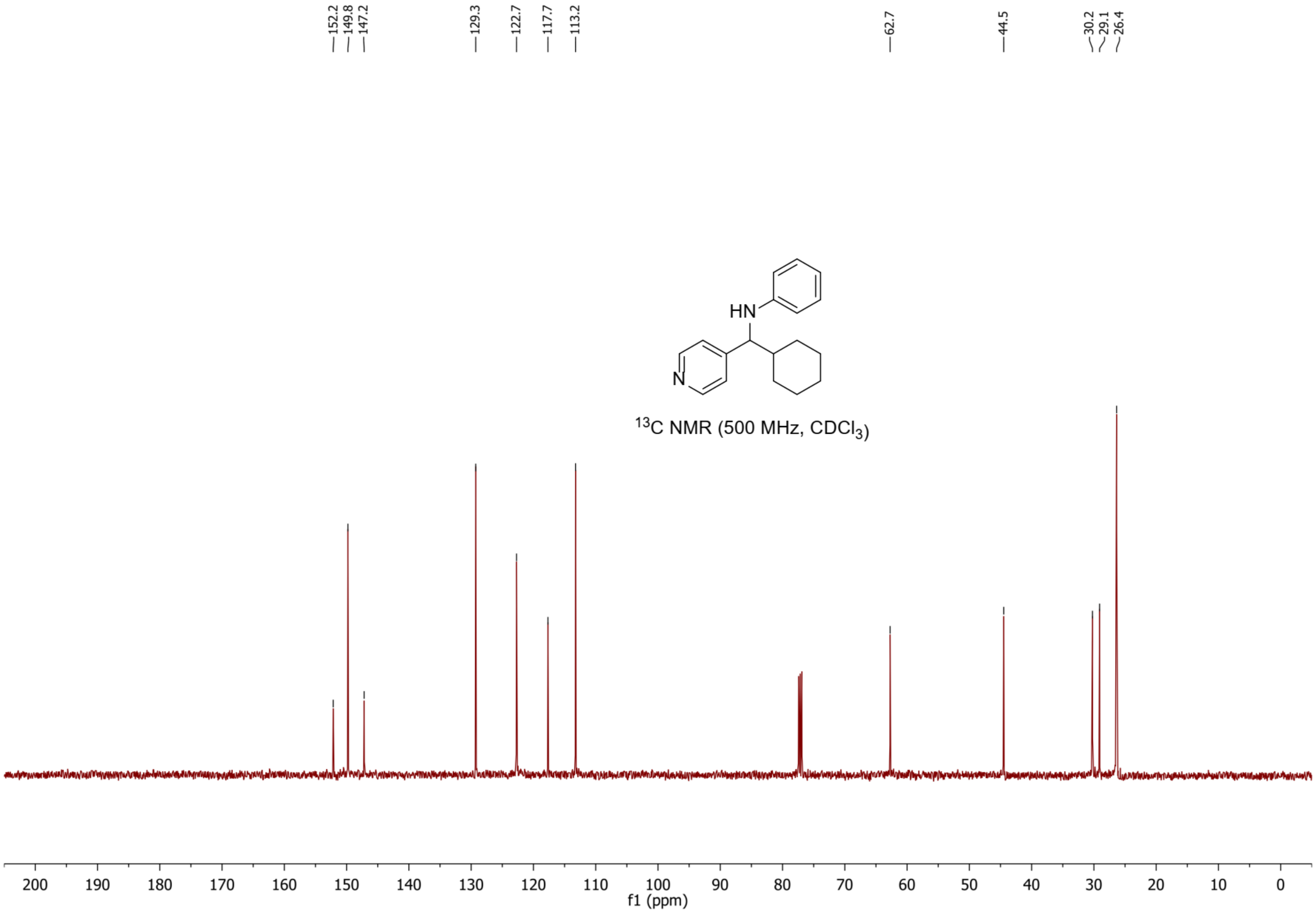

Go to table of contents

N-(Cyclohexyl(6-methylpyridin-2-yl)methyl)aniline (5m)

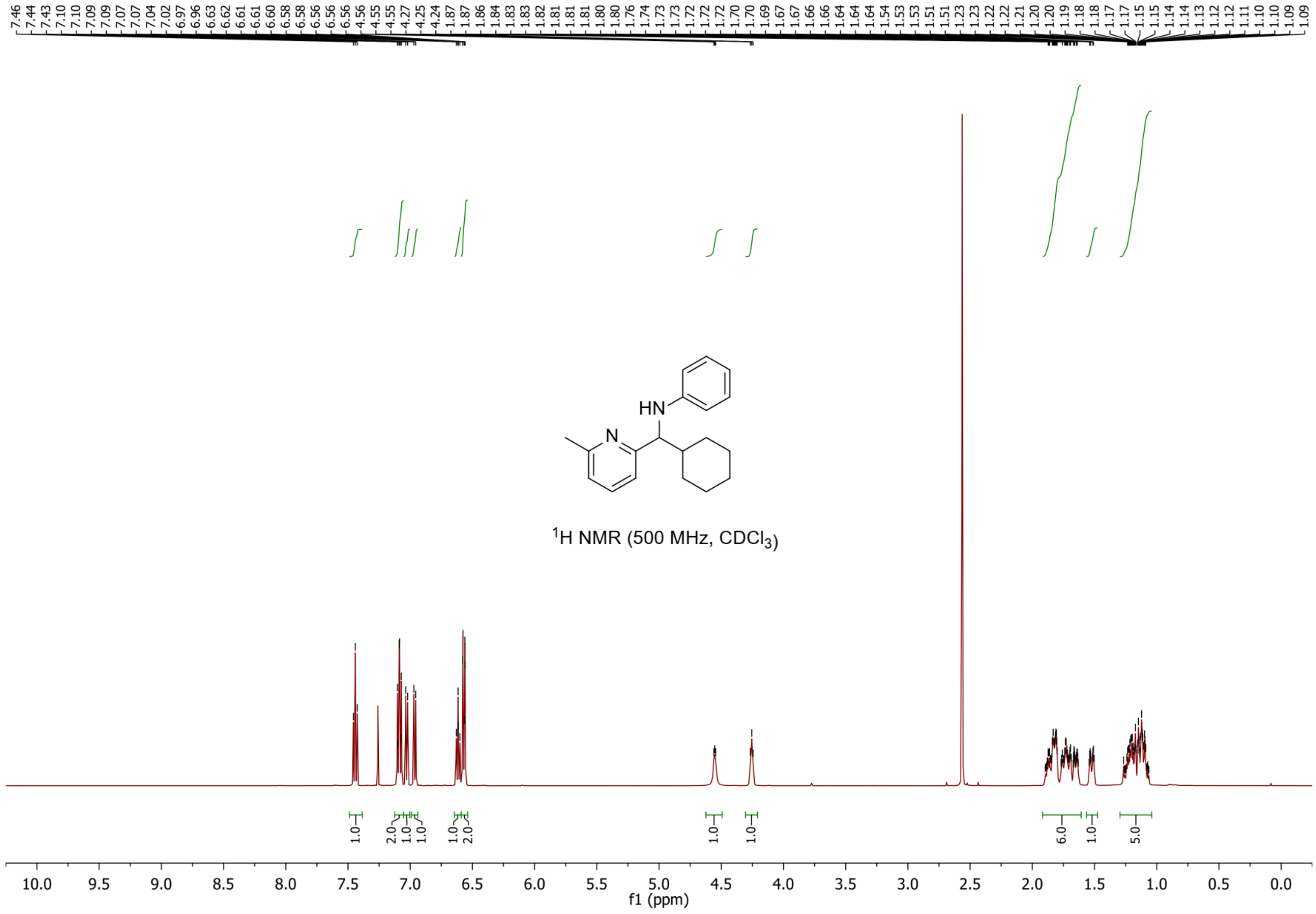

N-(Cyclohexyl(6-methylpyridin-2-yl)methyl)aniline (5m)

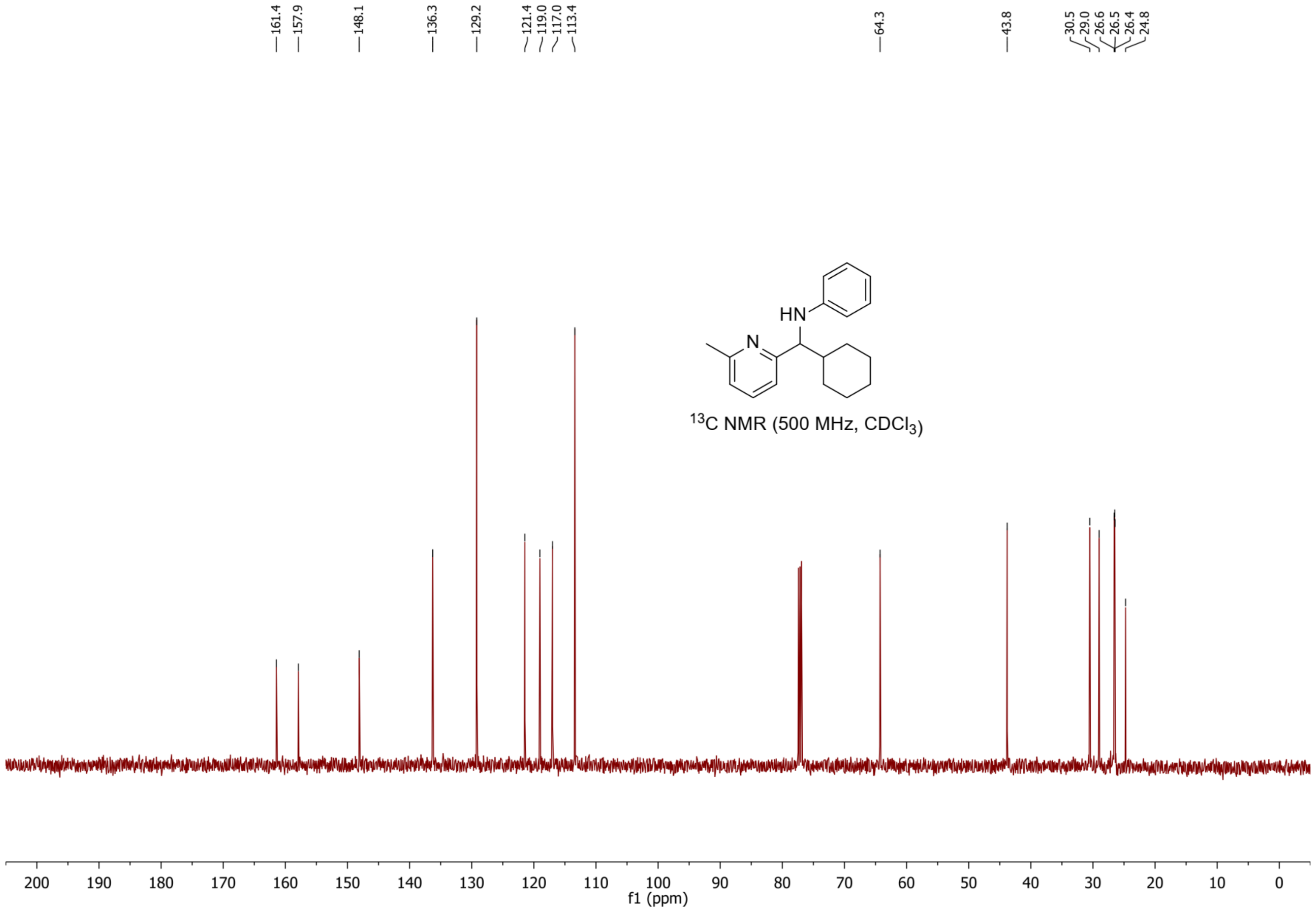

N-(Benzofuran-2-yl(cyclohexyl)methyl)aniline (5n)

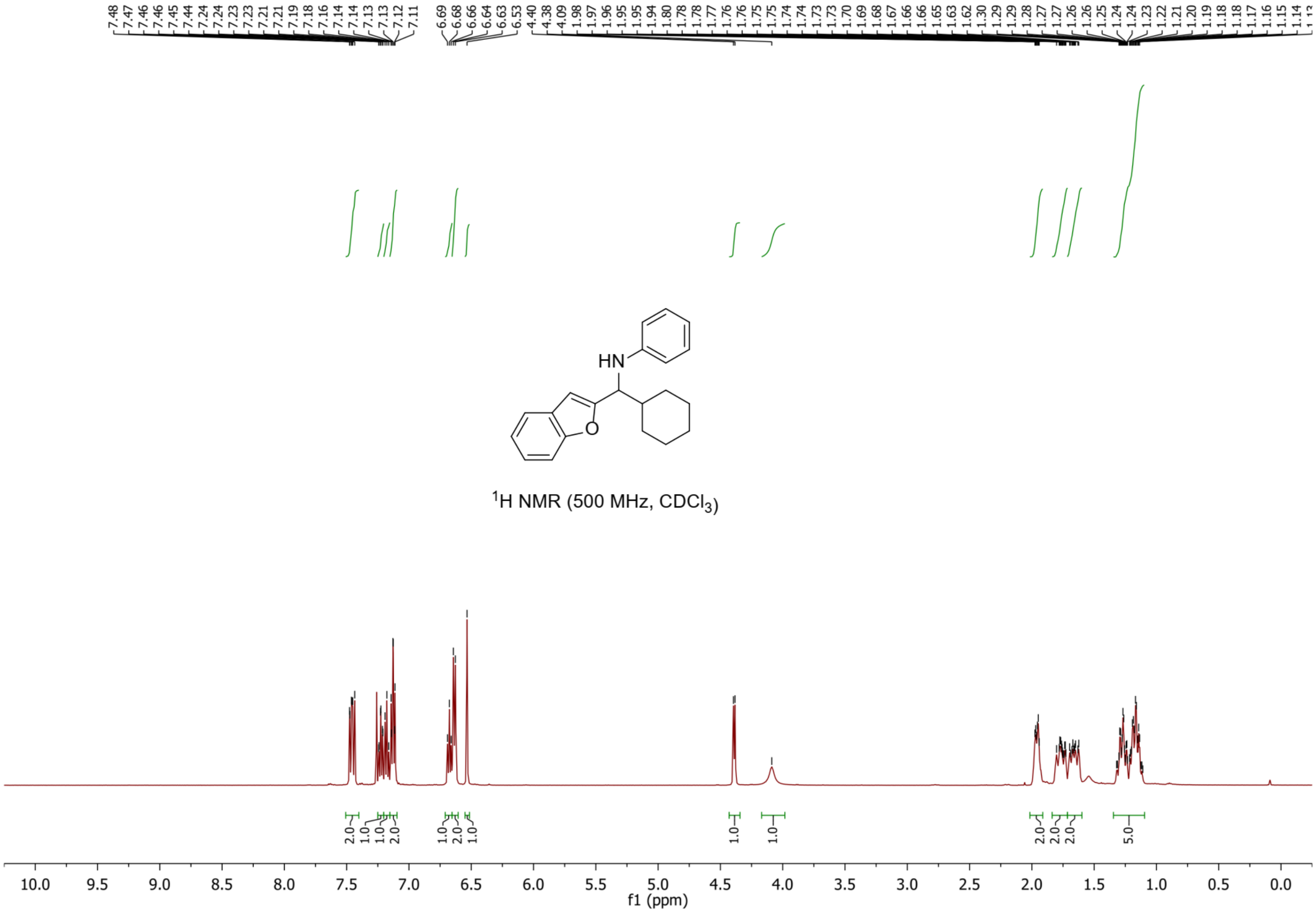

Go to table of contents

_N_-(Benzofuran-2-yl(cyclohexyl)methyl)aniline (5n)

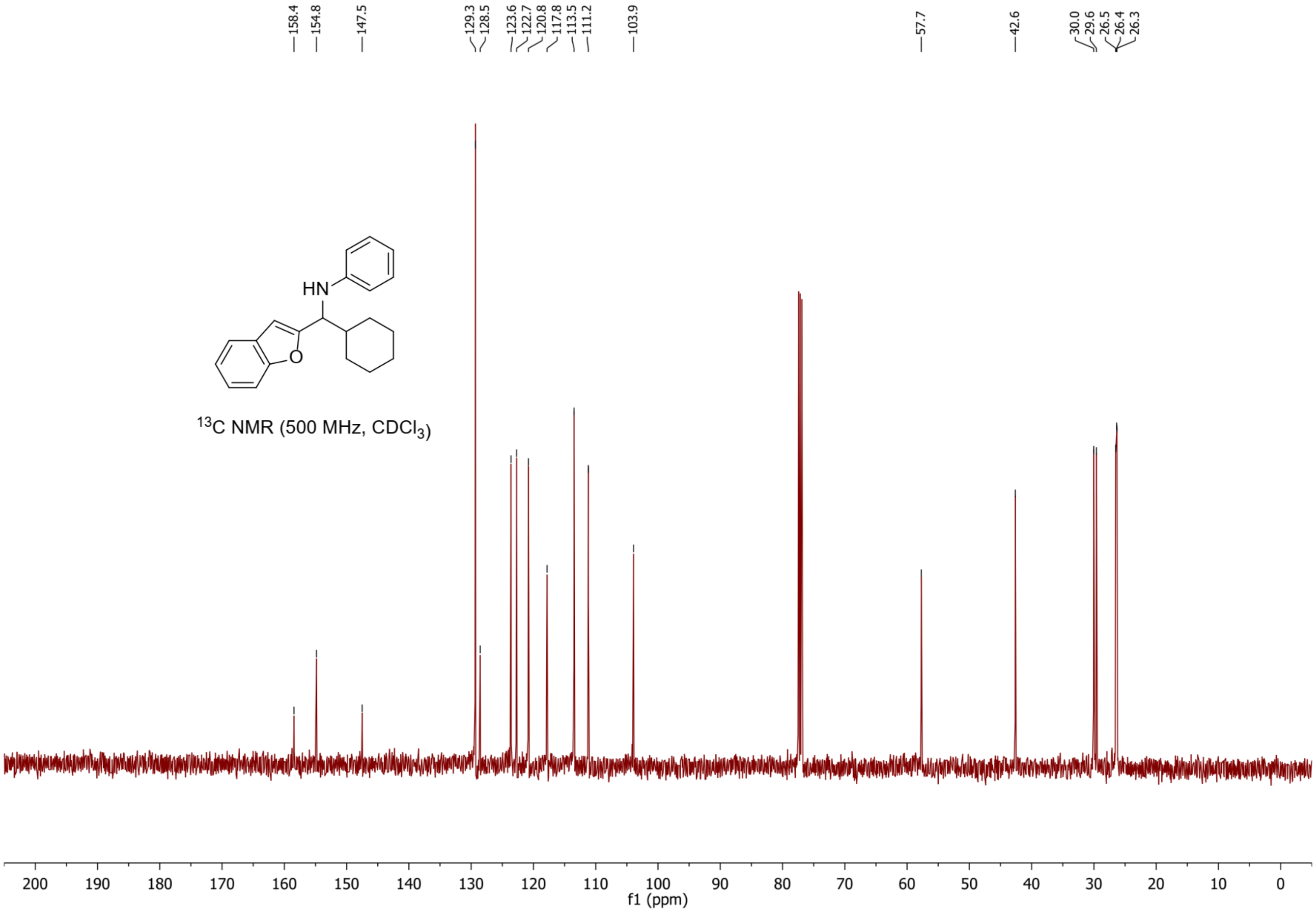

tert-butyl 5-(cyclohexyl(phenylamino)methyl)-1H-indole-1-carboxylate (5o)

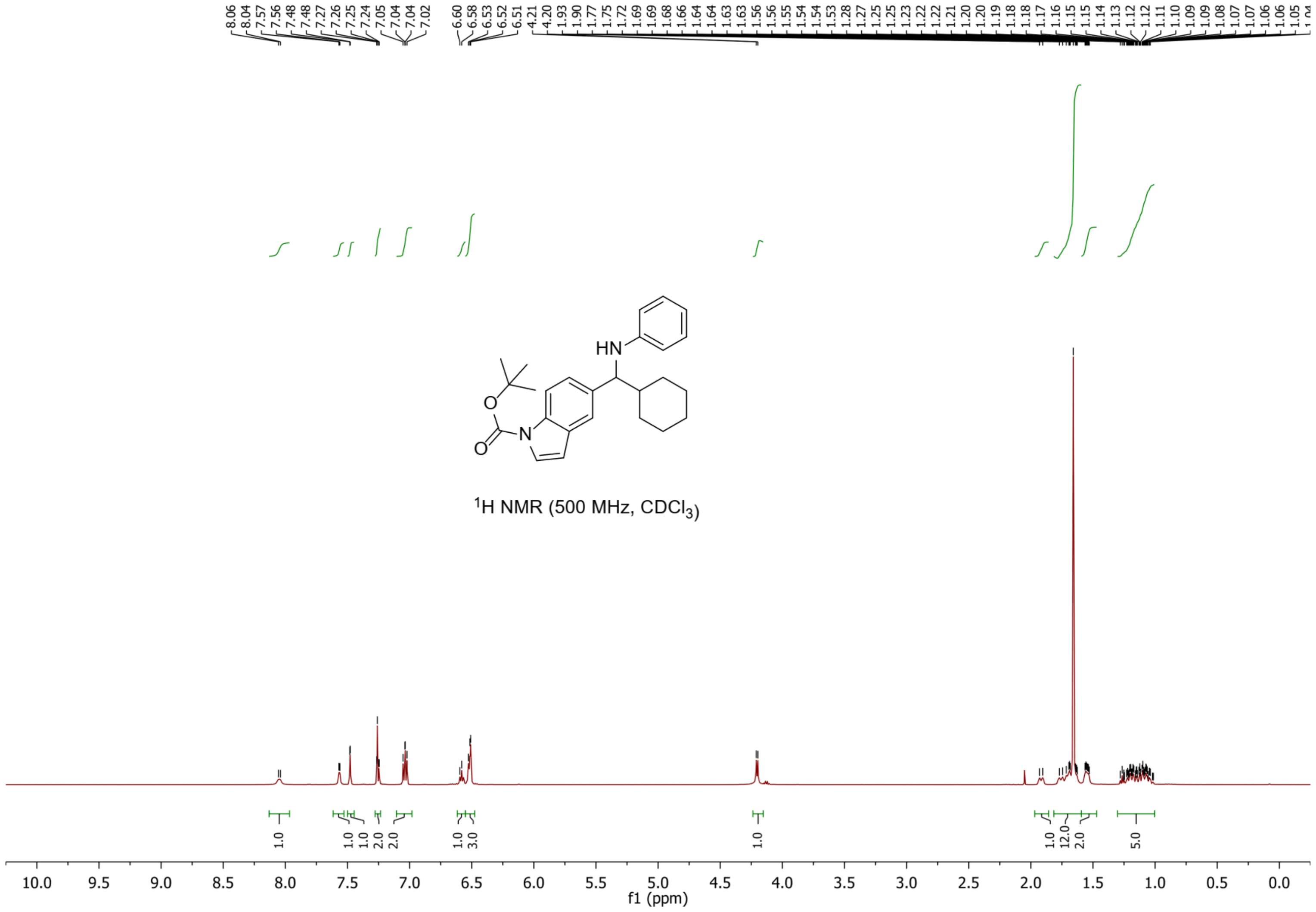

Go to table of contents

tert-butyl 5-(cyclohexyl(phenylamino)methyl)-1H-indole-1-carboxylate (5o)

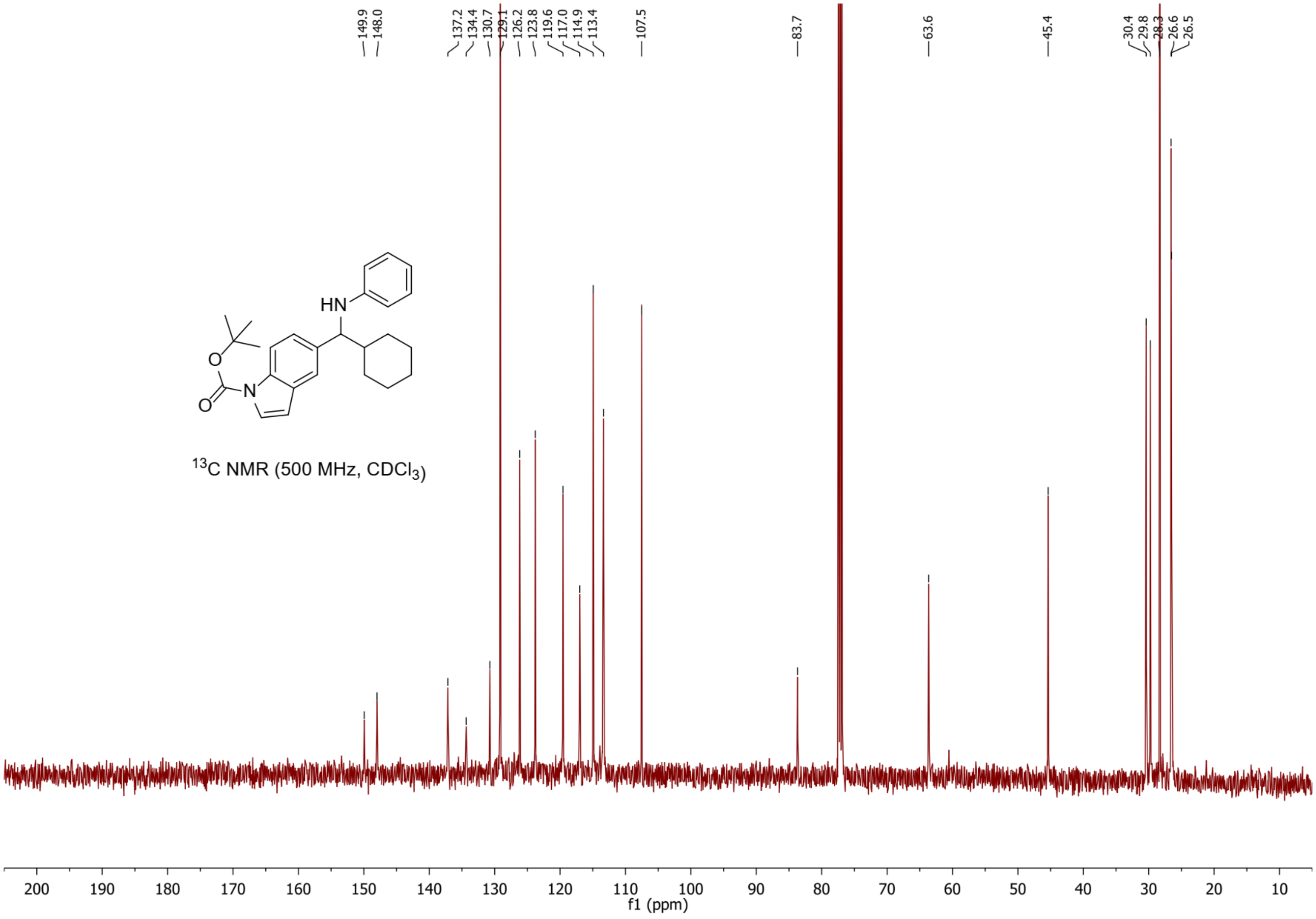

Go to table of contents

1-(5-(Cyclohexyl(phenylamino)methyl)-1*H*-indol-1-yl)ethan-1-one (5p)

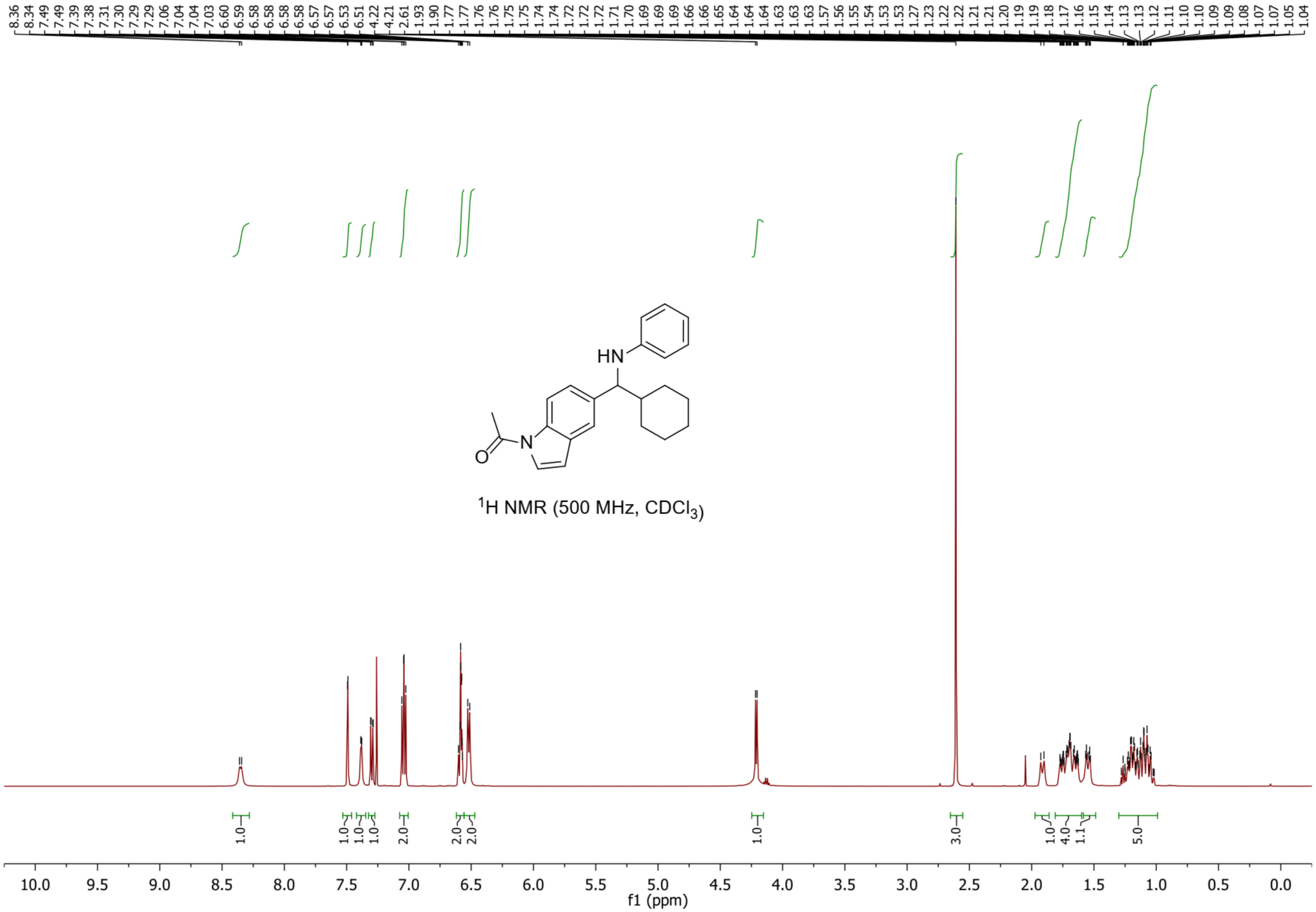

1-(5-(Cyclohexyl(phenylamino)methyl)-1*H*-indol-1-yl)ethan-1-one (5p)

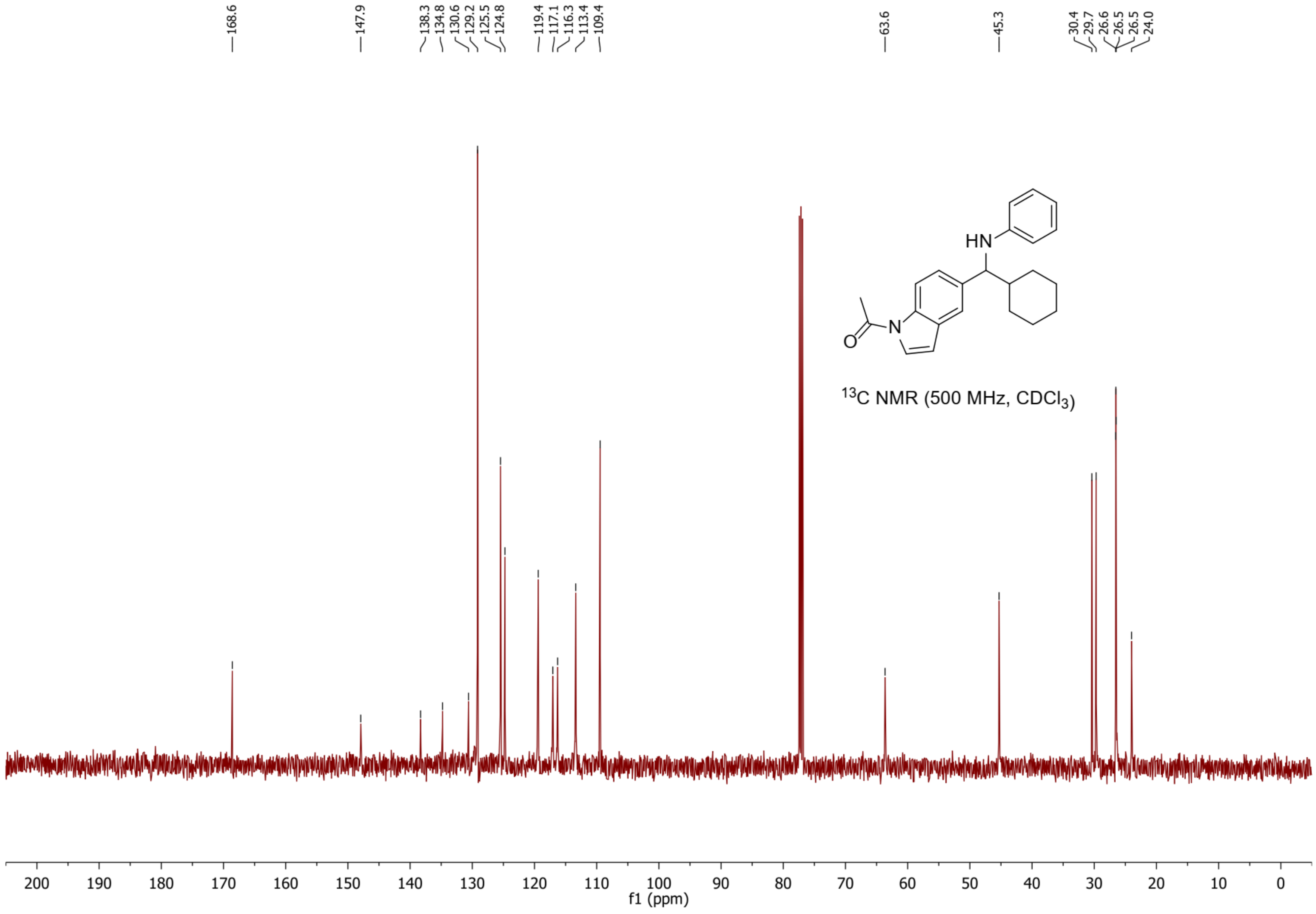

Go to table of contents

N-(1-Phenylpentyl)aniline (6a)

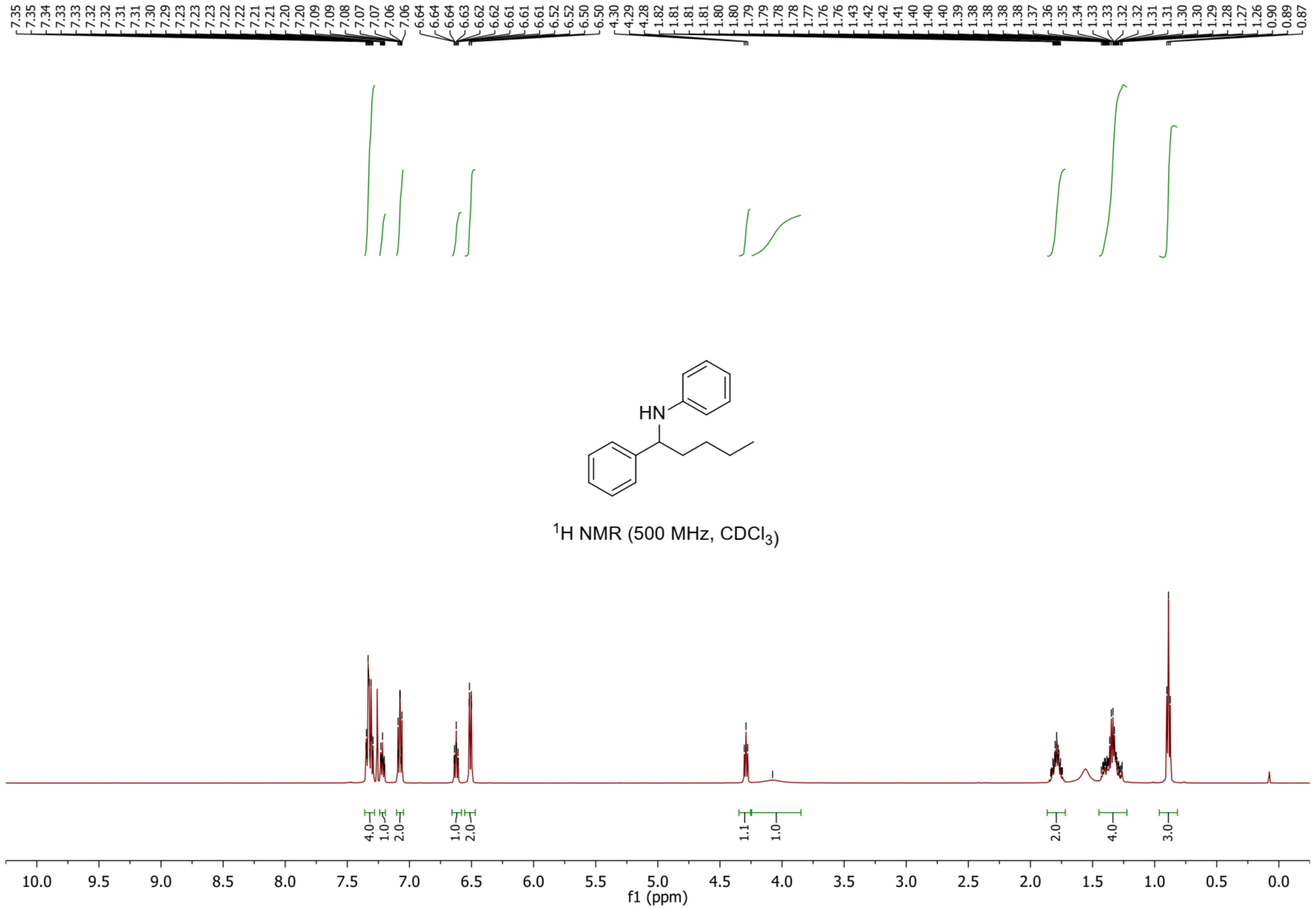

Go to table of contents

N-(1-Phenylpentyl)aniline (6a)

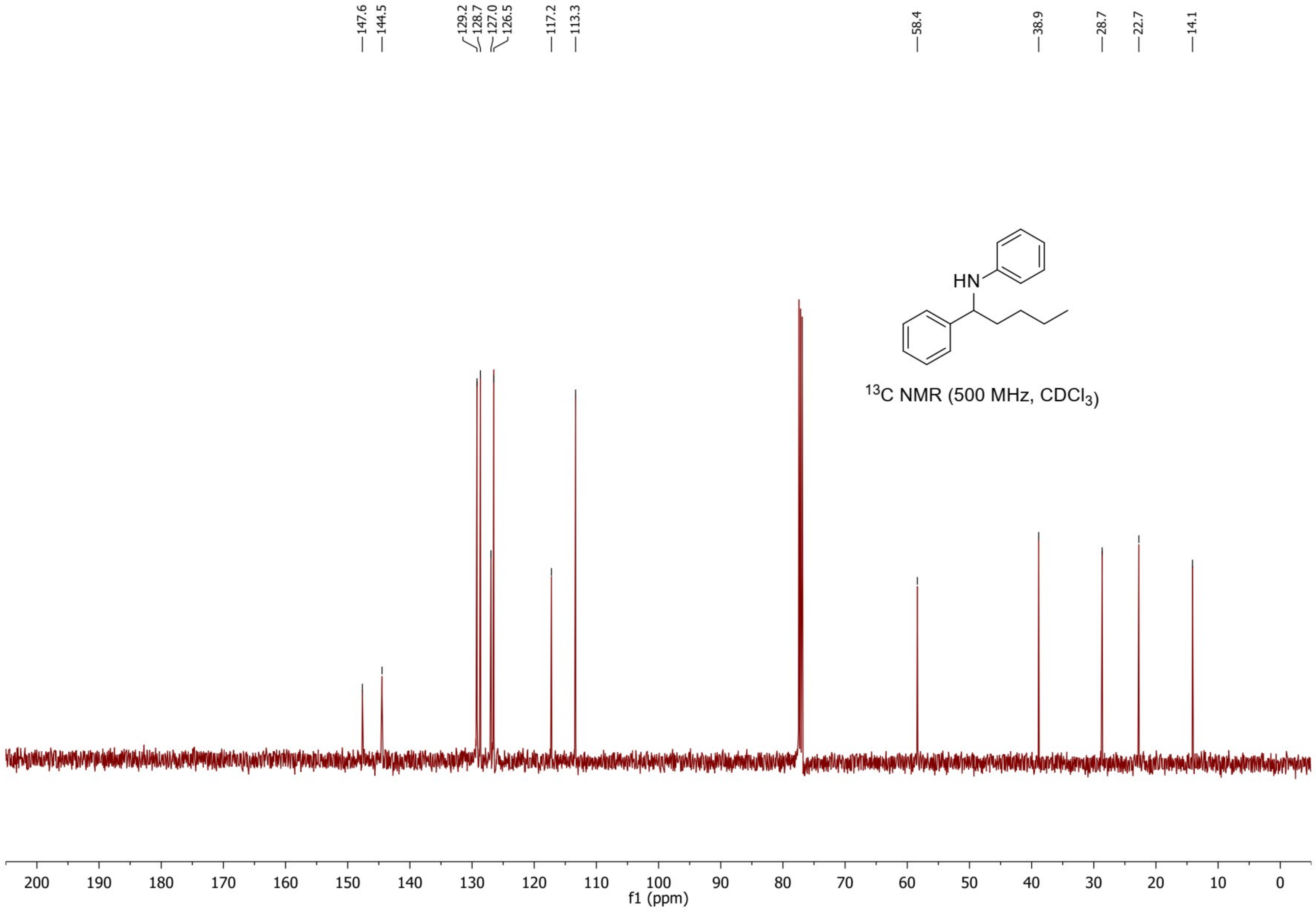

Go to table of contents

N-(2-Methyl-1-phenylpropyl)aniline (6b)

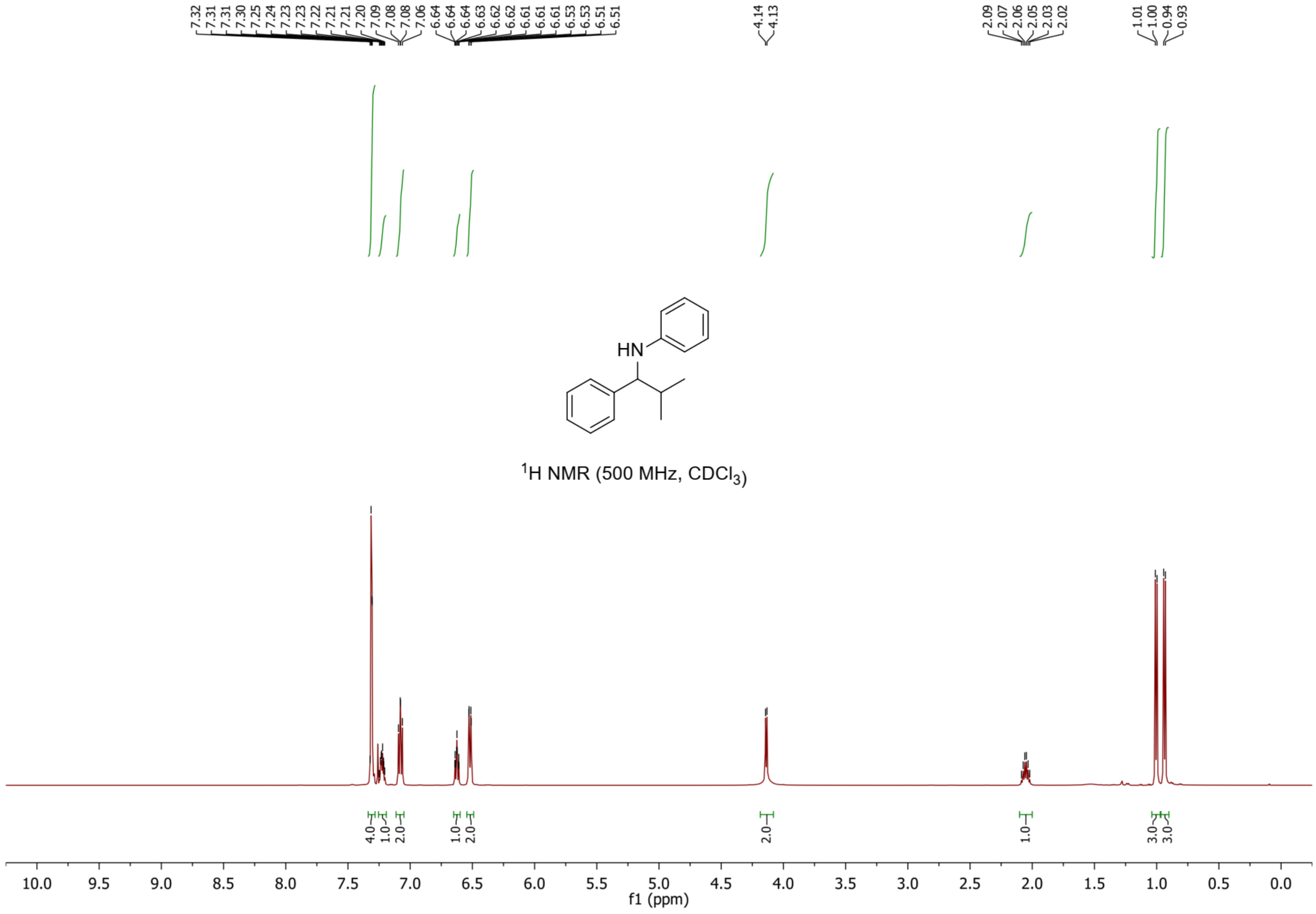

Go to table of contents

_N_-(2-Methyl-1-phenylpropyl)aniline (6b)

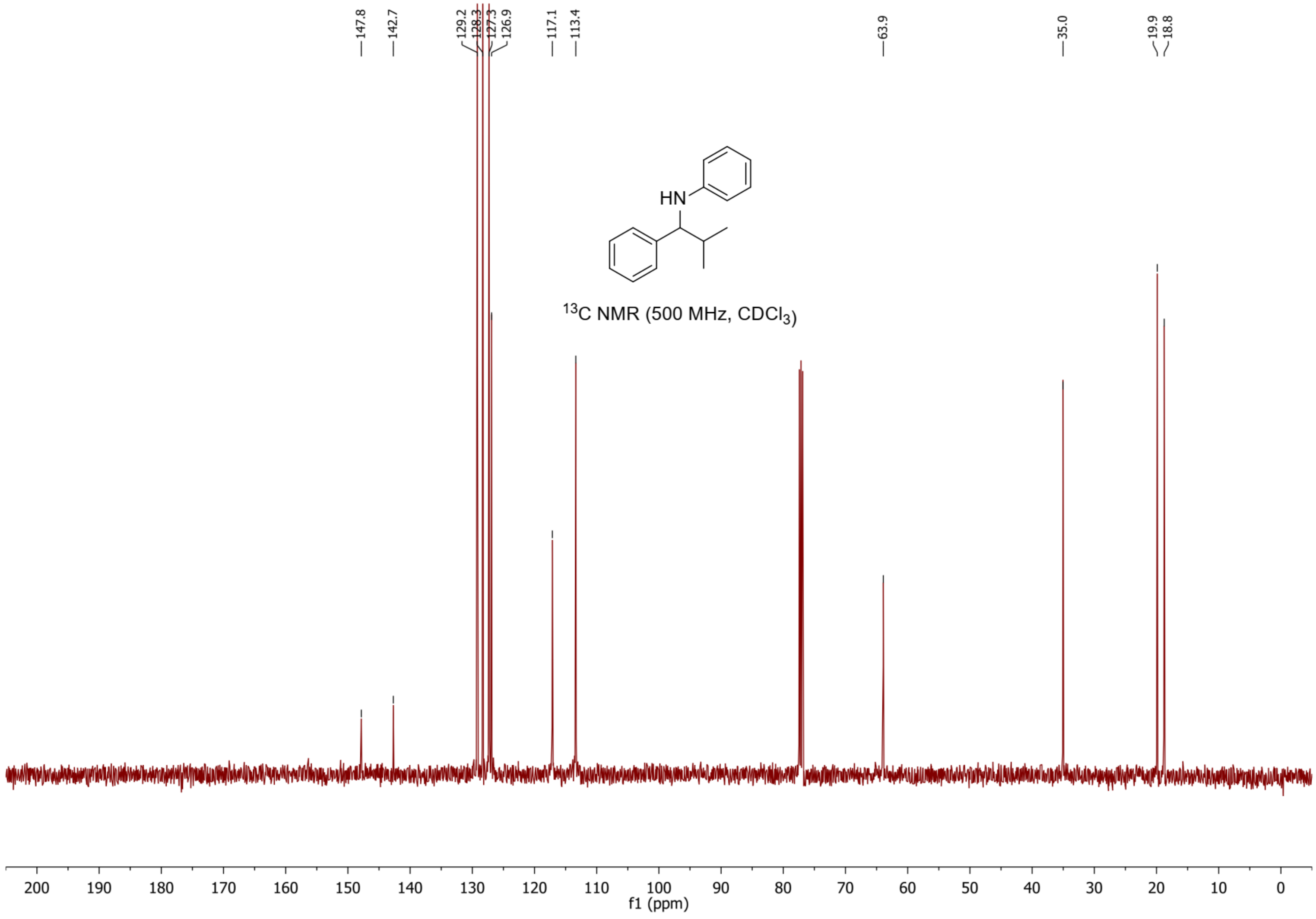

Go to table of contents

N-(1-Phenyl-2-propylpentyl)aniline (6c)

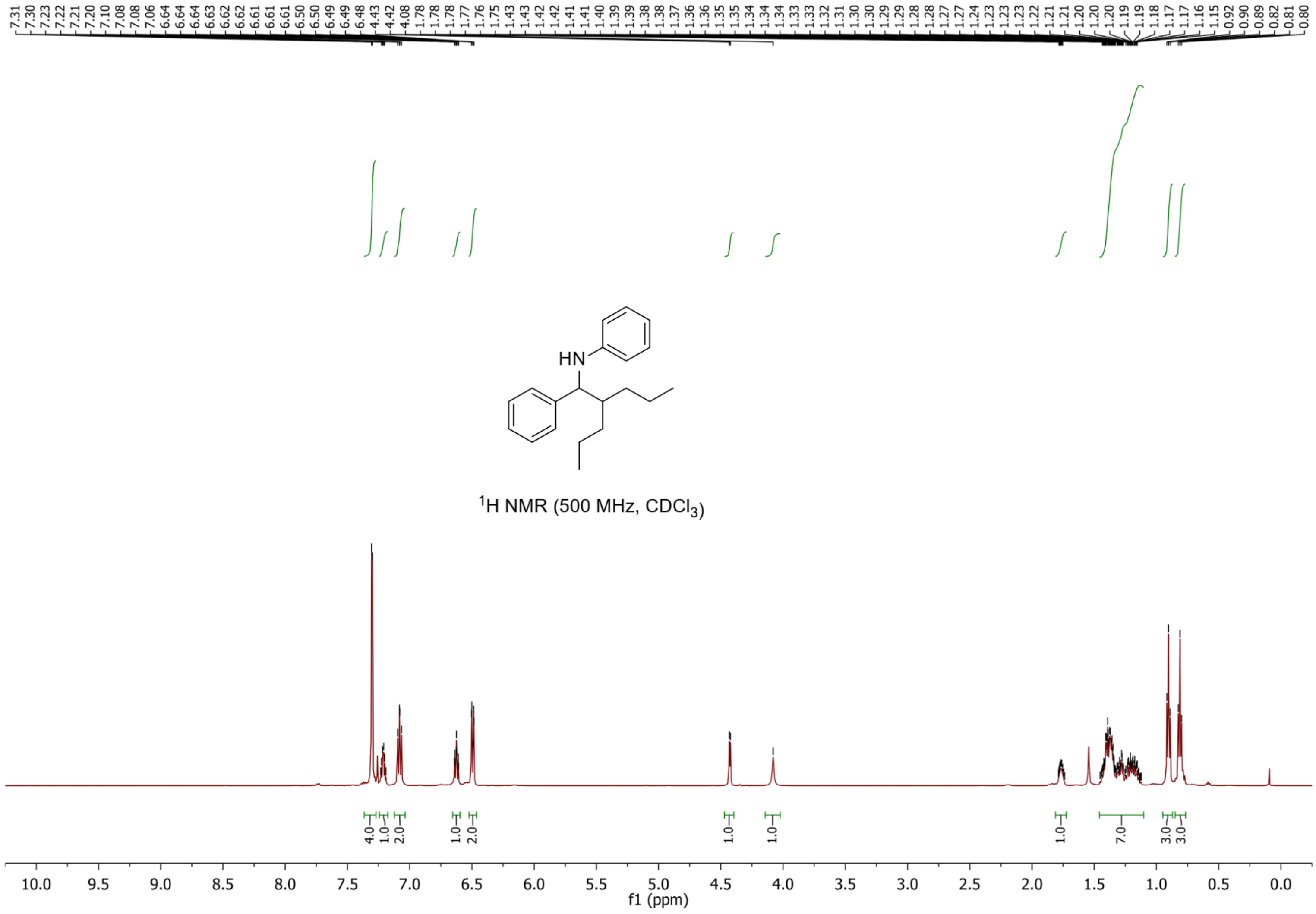

N-(1-Phenyl-2-propylpentyl)aniline (6c)

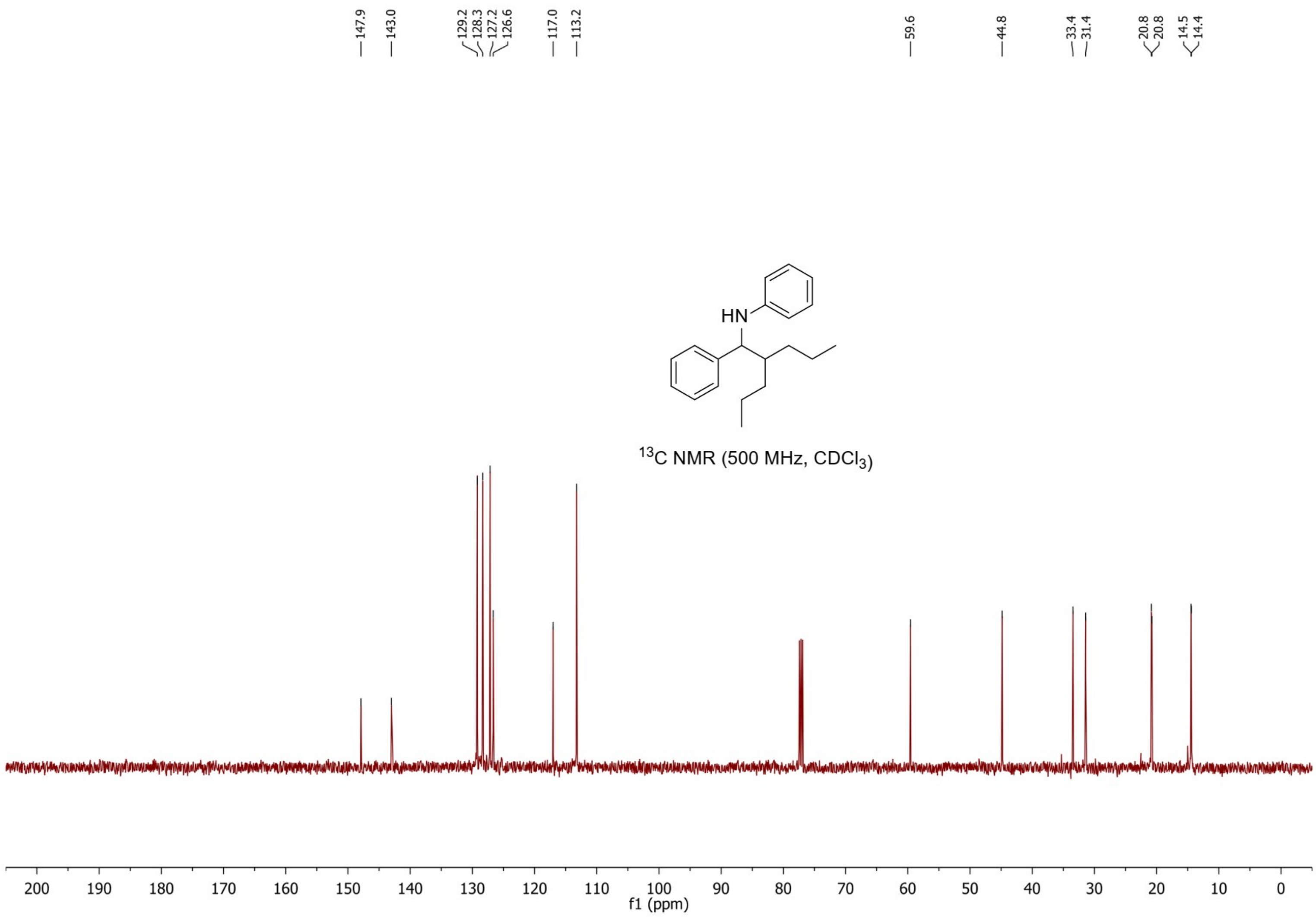

_N_-(2-Hexyl-1-phenyldecyl)aniline (6d)

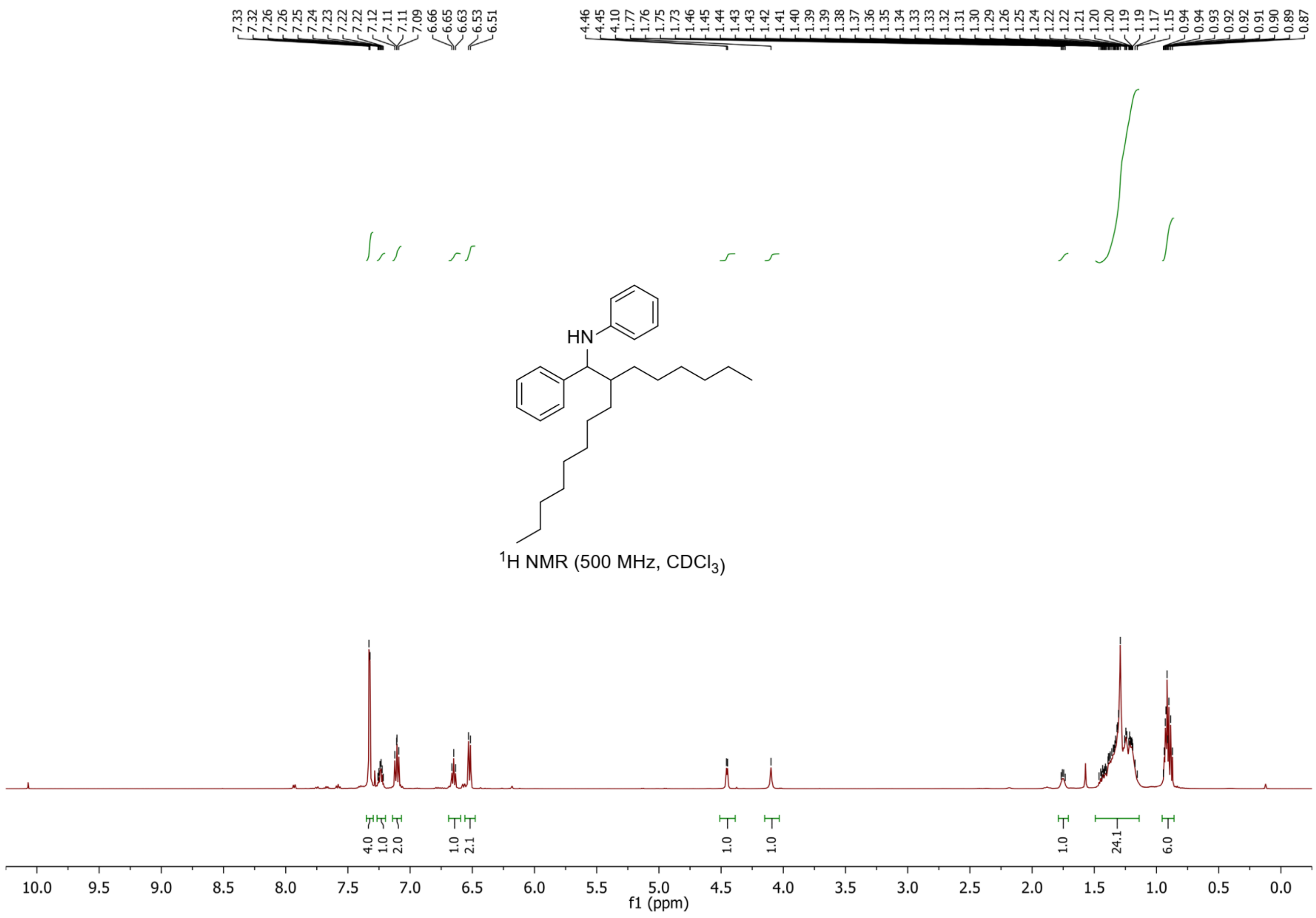

Go to table of contents

N-(2-Hexyl-1-phenyldecyl)aniline (6d)

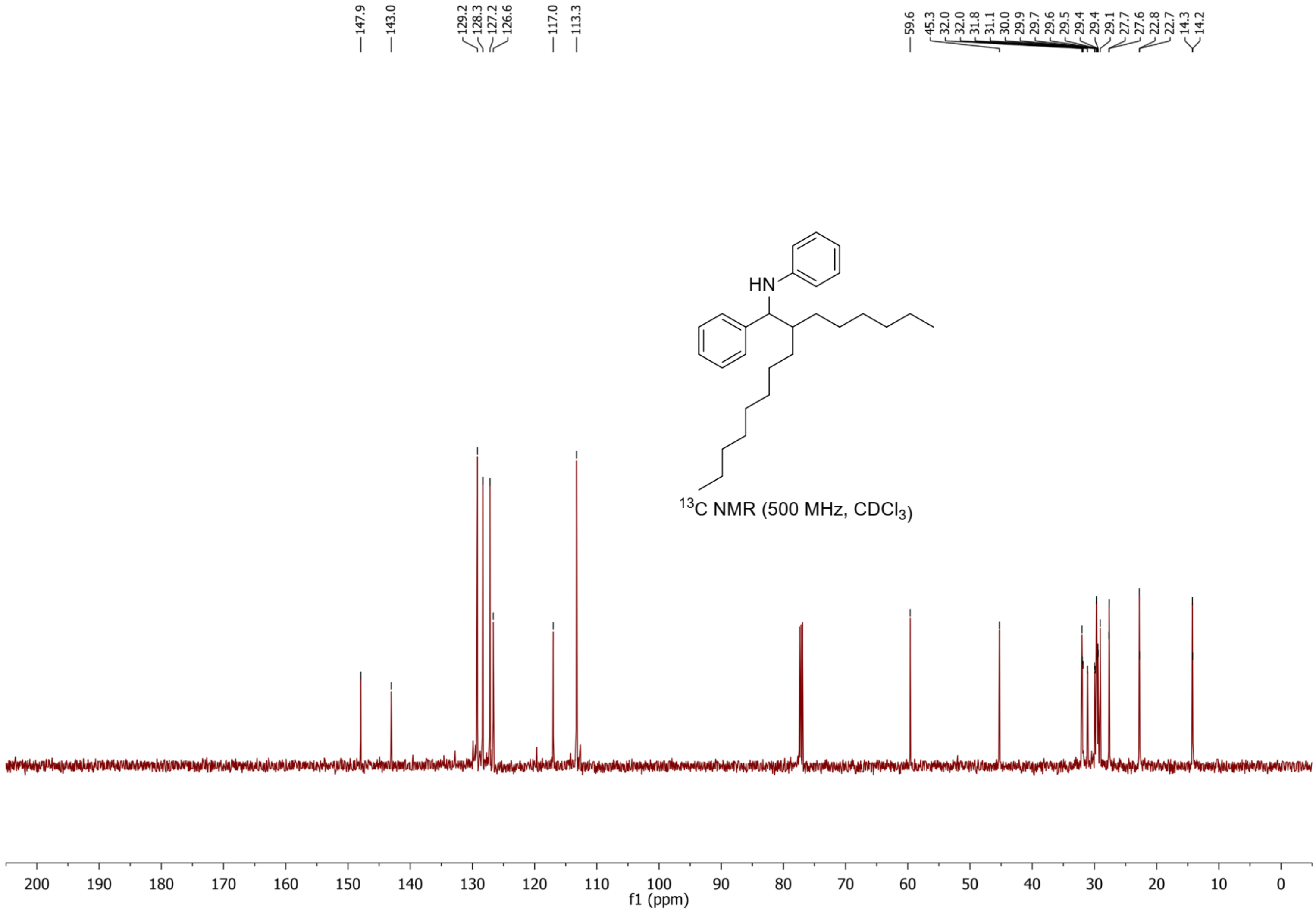

Go to table of contents

N-(Cyclobutyl(phenyl)methyl)aniline (6e)

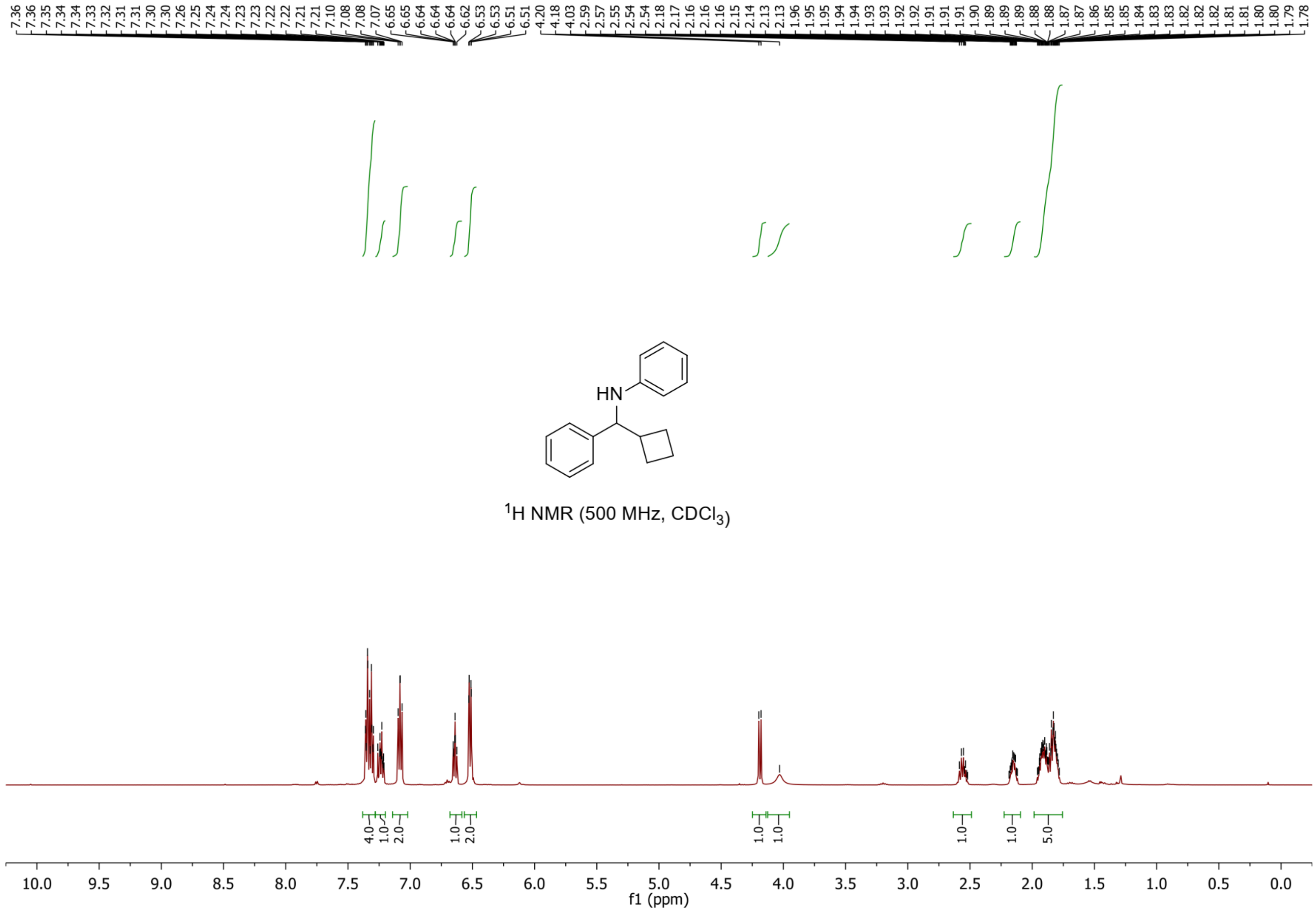

Go to table of contents

N-(Cyclobutyl(phenyl)methyl)aniline (6e)

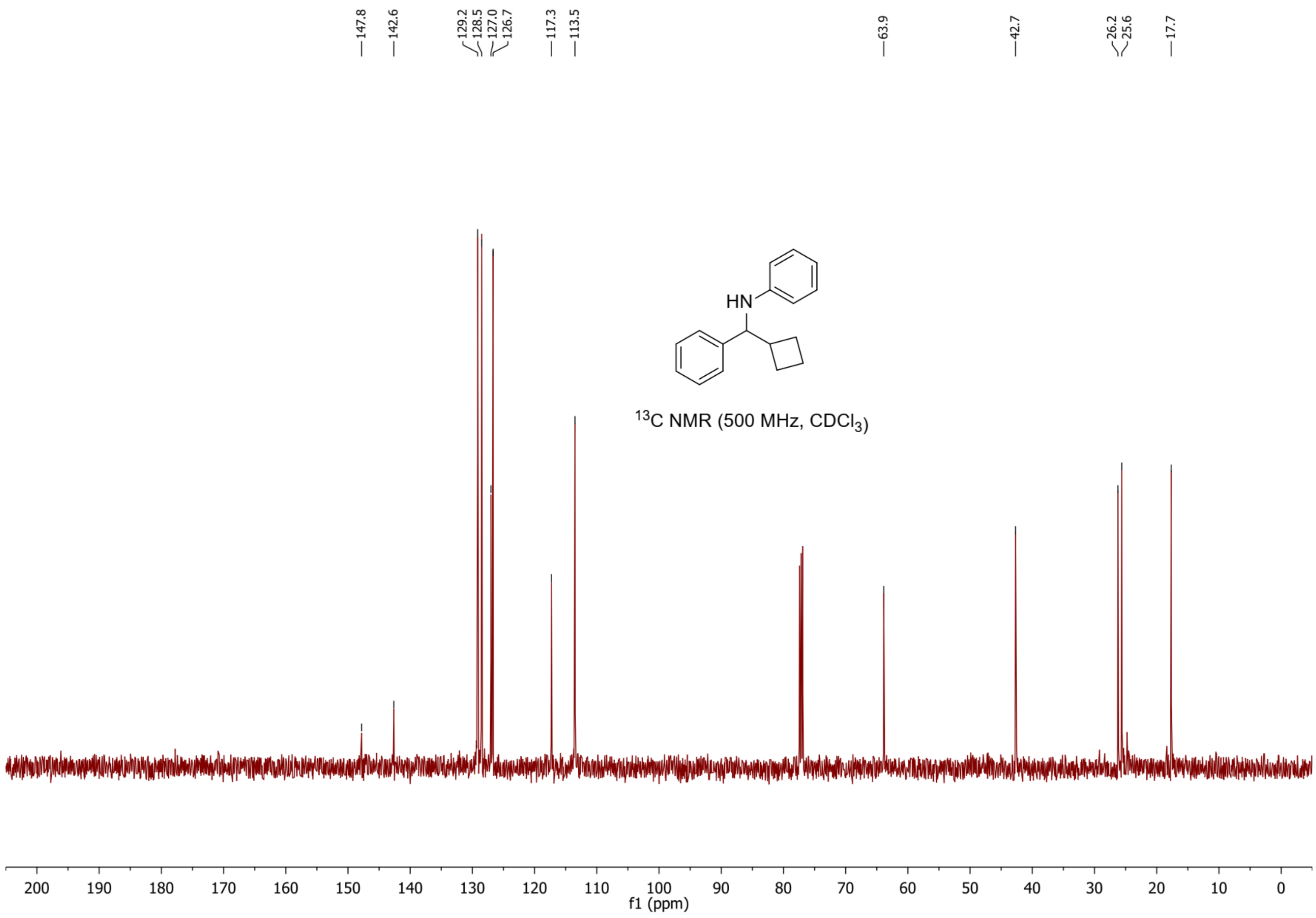

_N_-(Cyclopentyl(phenyl)methyl)aniline (6f)

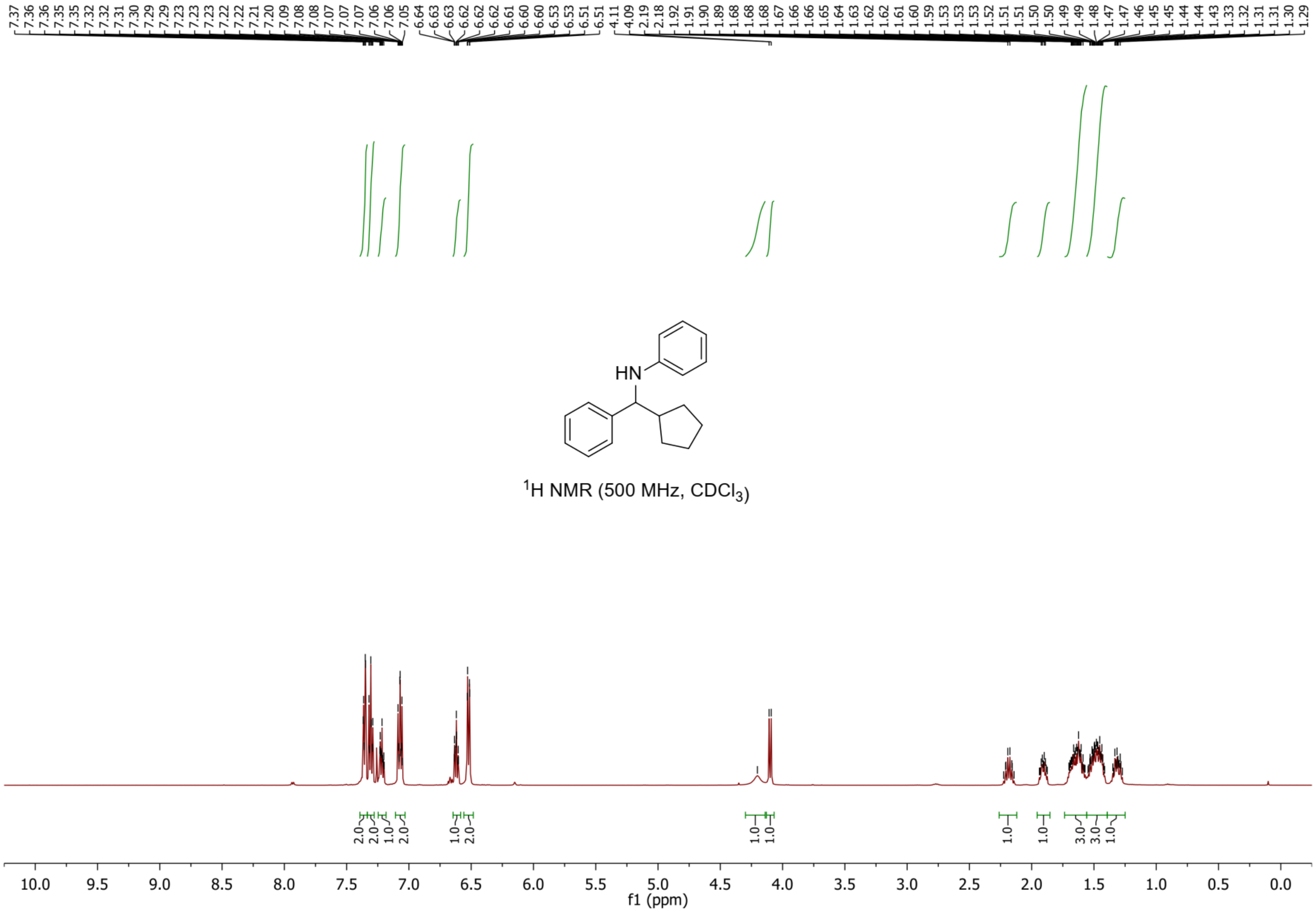

Go to table of contents

N-(Cyclopentyl(phenyl)methyl)aniline (6f)

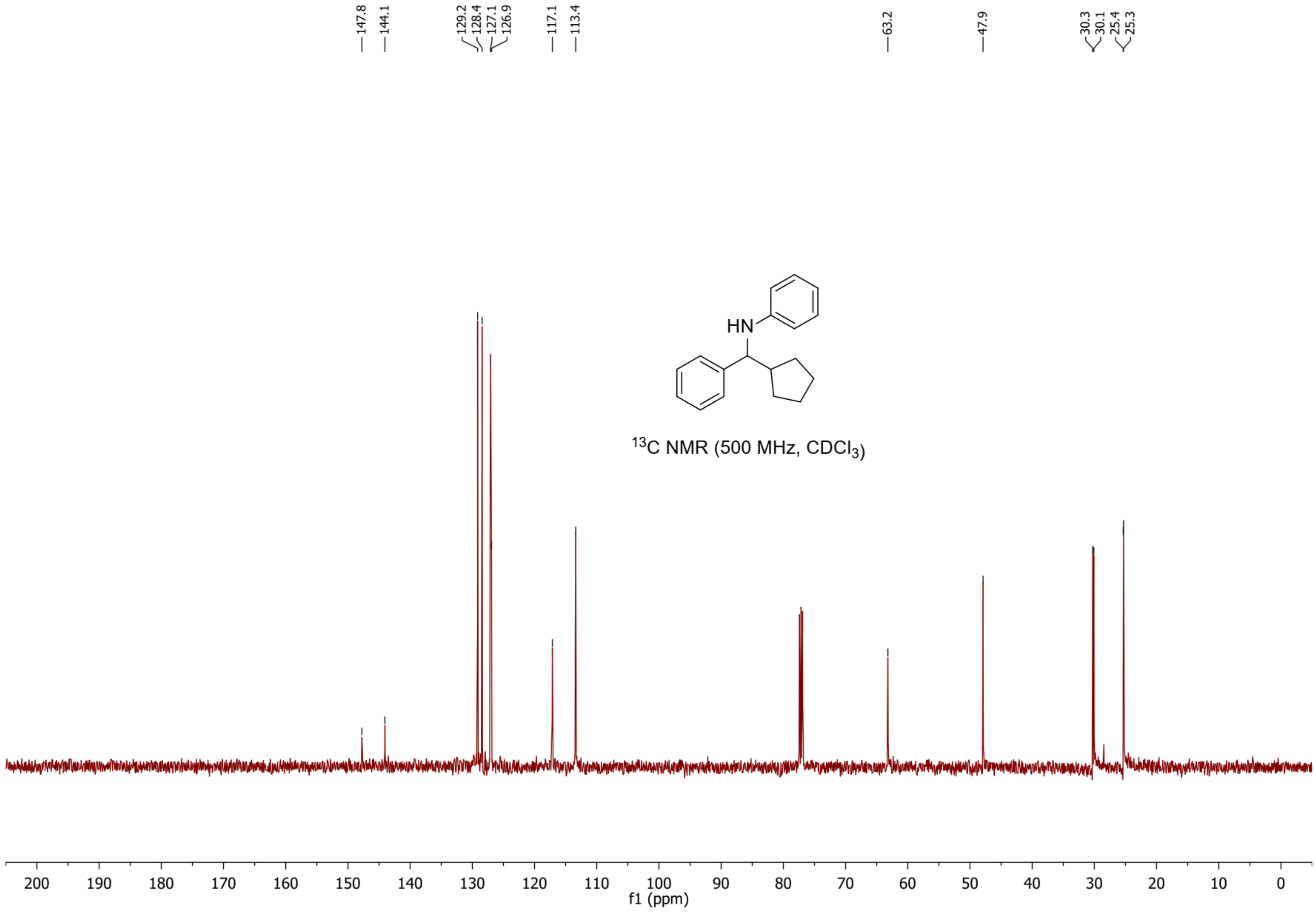

_N_-(Cycloheptyl(phenyl)methyl)aniline (6g)

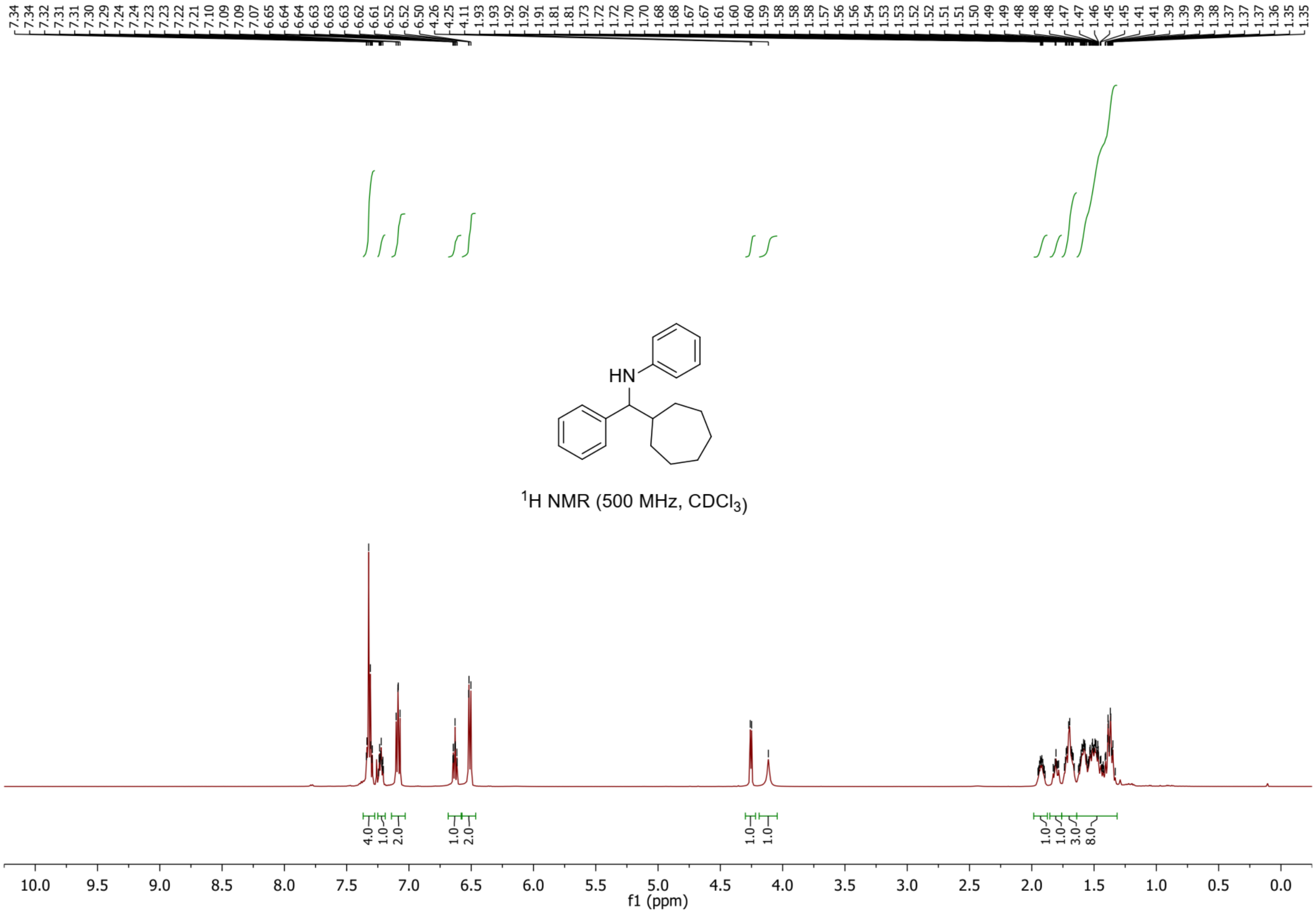

N-(Cycloheptyl(phenyl)methyl)aniline (6g)

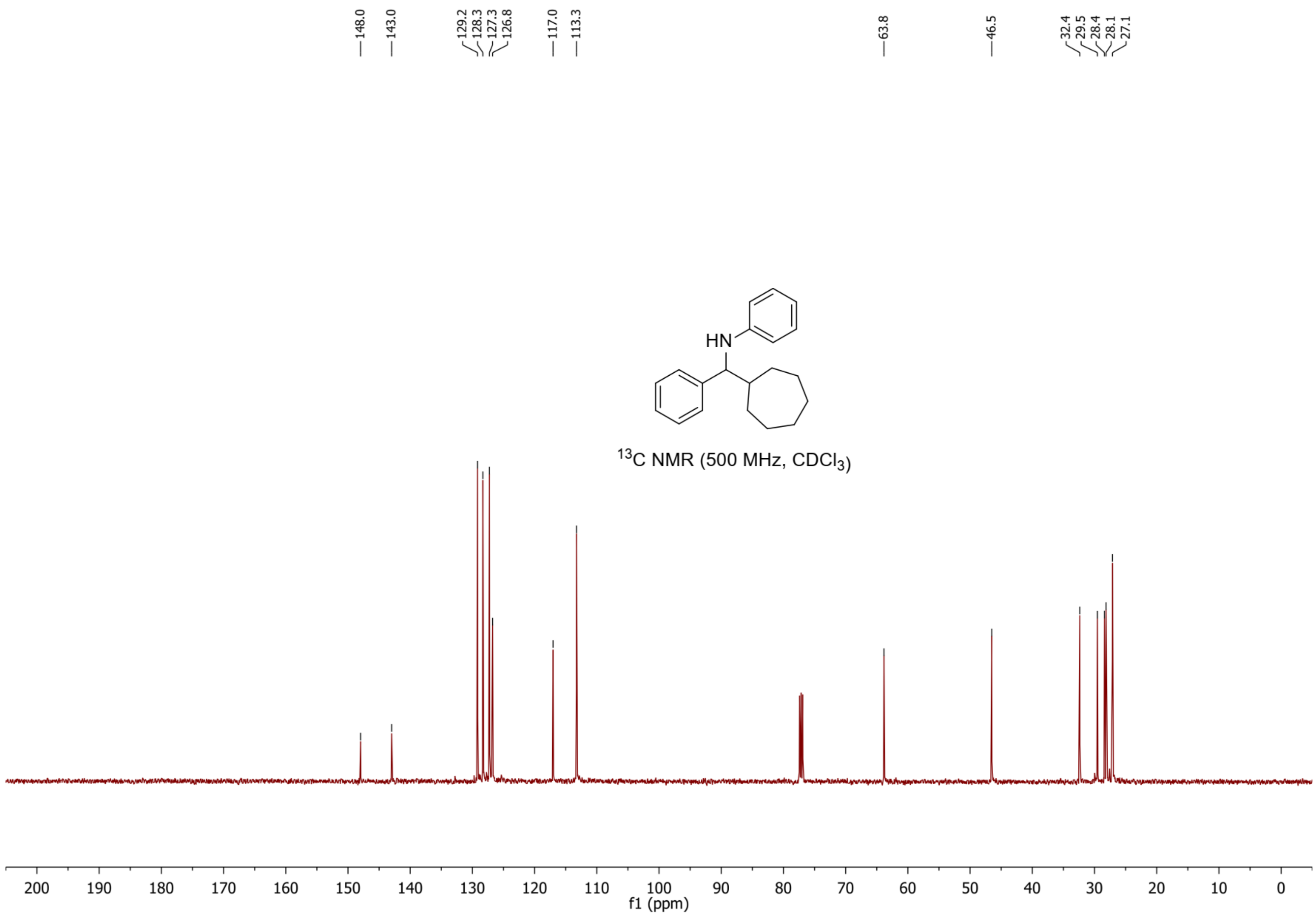

Go to table of contents

_N_-(Phenyl(tetrahydro-2_H_-pyran-4-yl)methyl)aniline (6h)

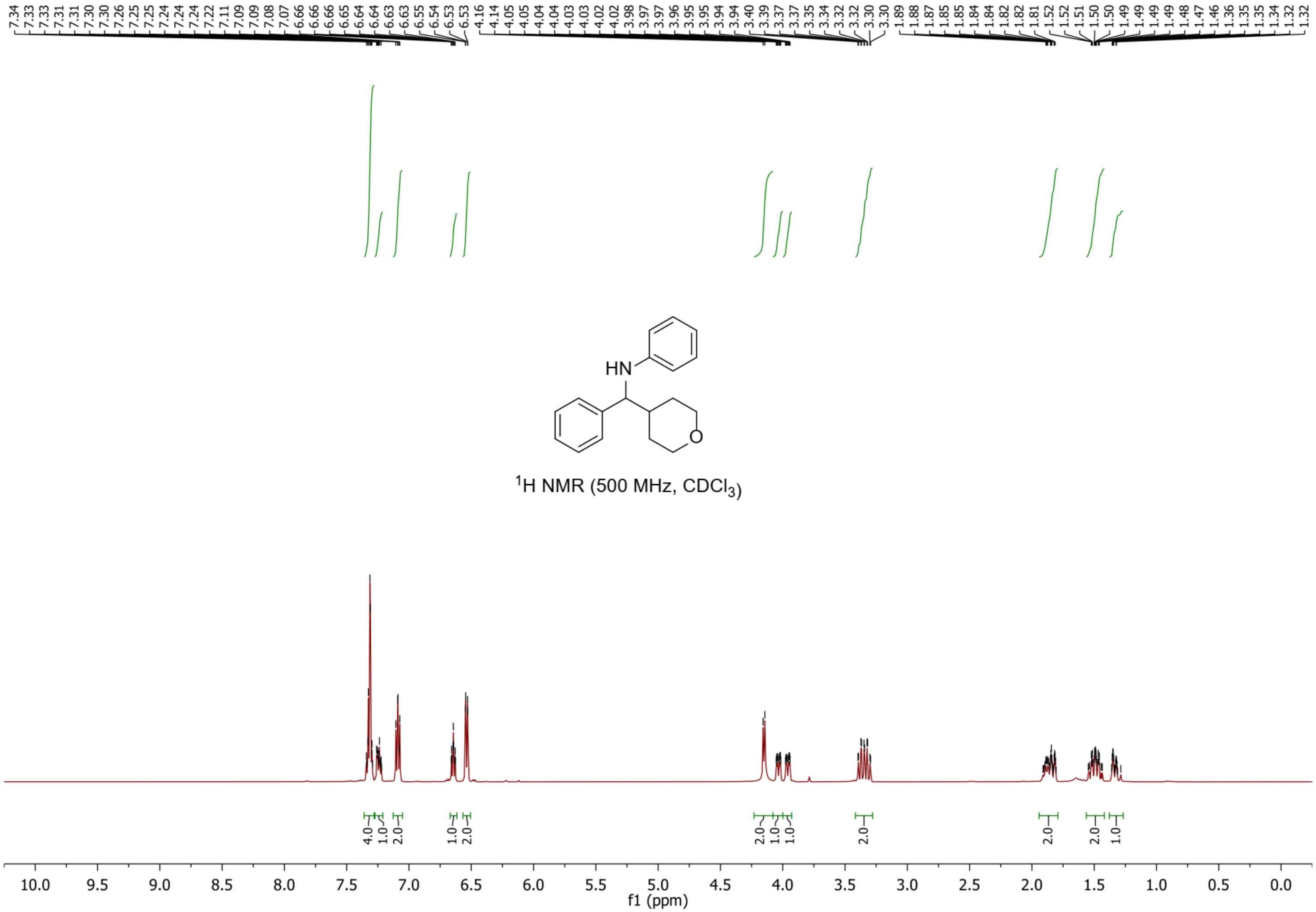

Go to table of contents

N-(Phenyl(tetrahydro-2H-pyran-4-yl)methyl)aniline (6h)

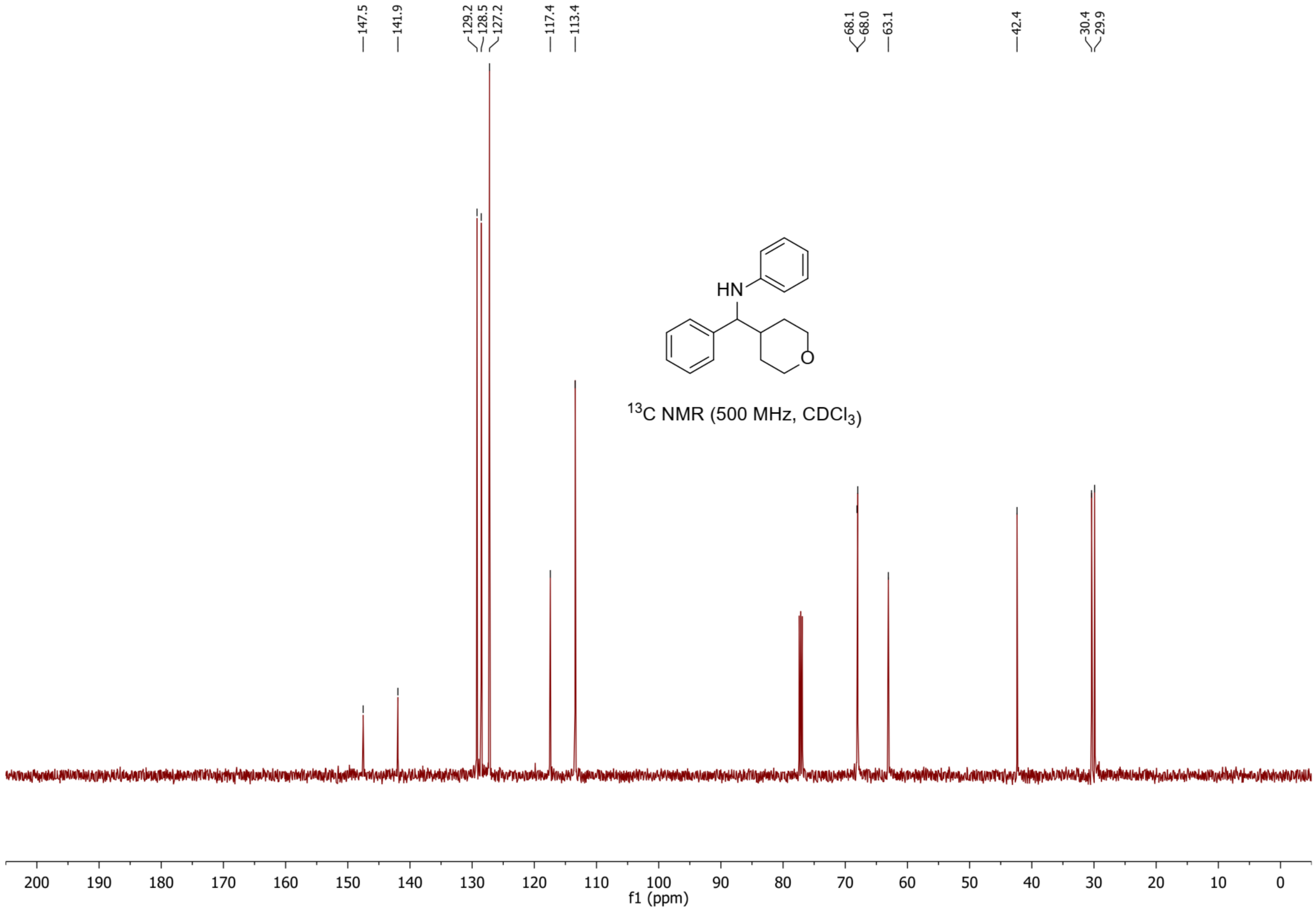

Go to table of contents

N-(2,2-Dimethyl-1-phenylpropyl)aniline (6i)

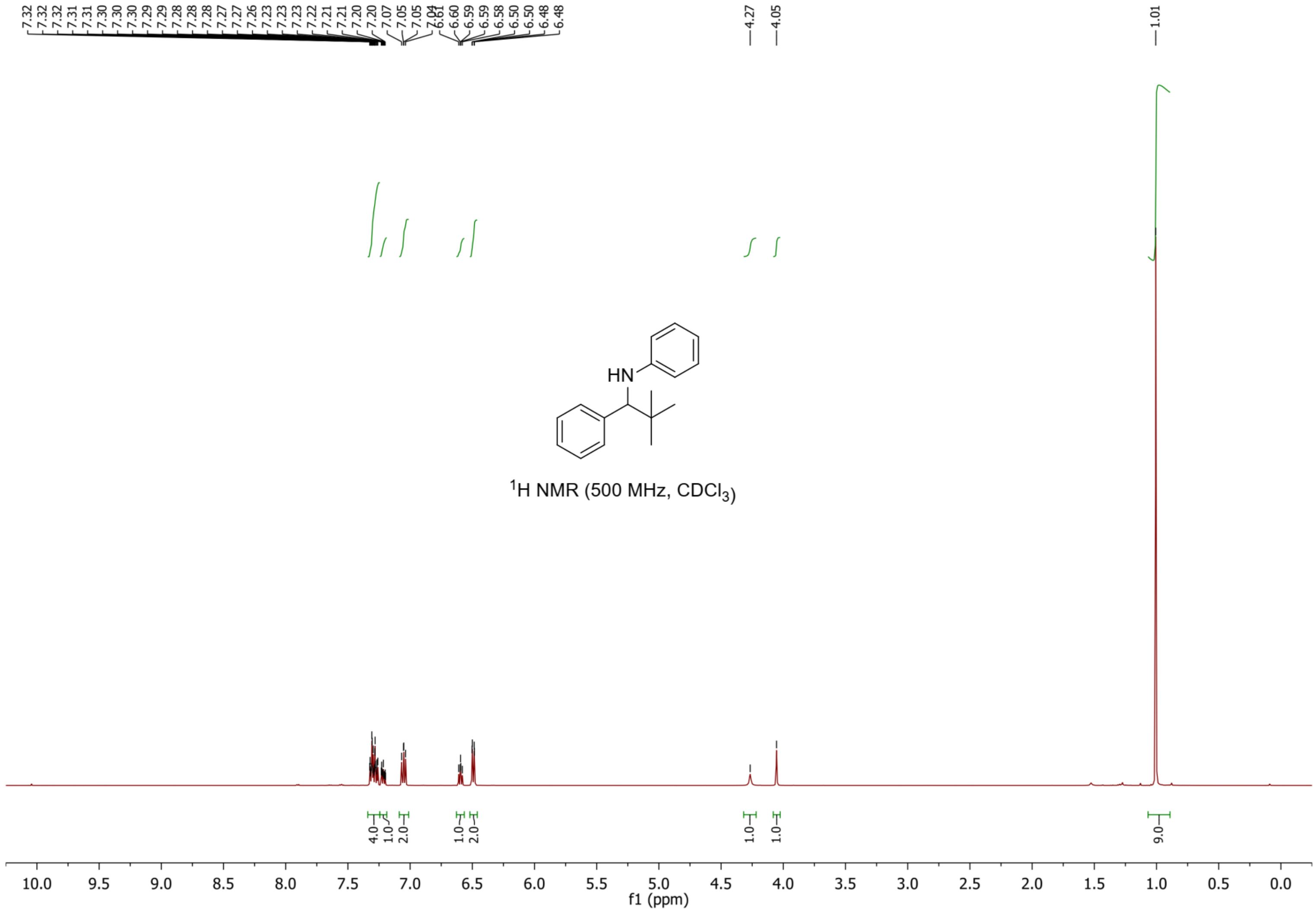

Go to table of contents

N-(2,2-Dimethyl-1-phenylpropyl)aniline (6i)

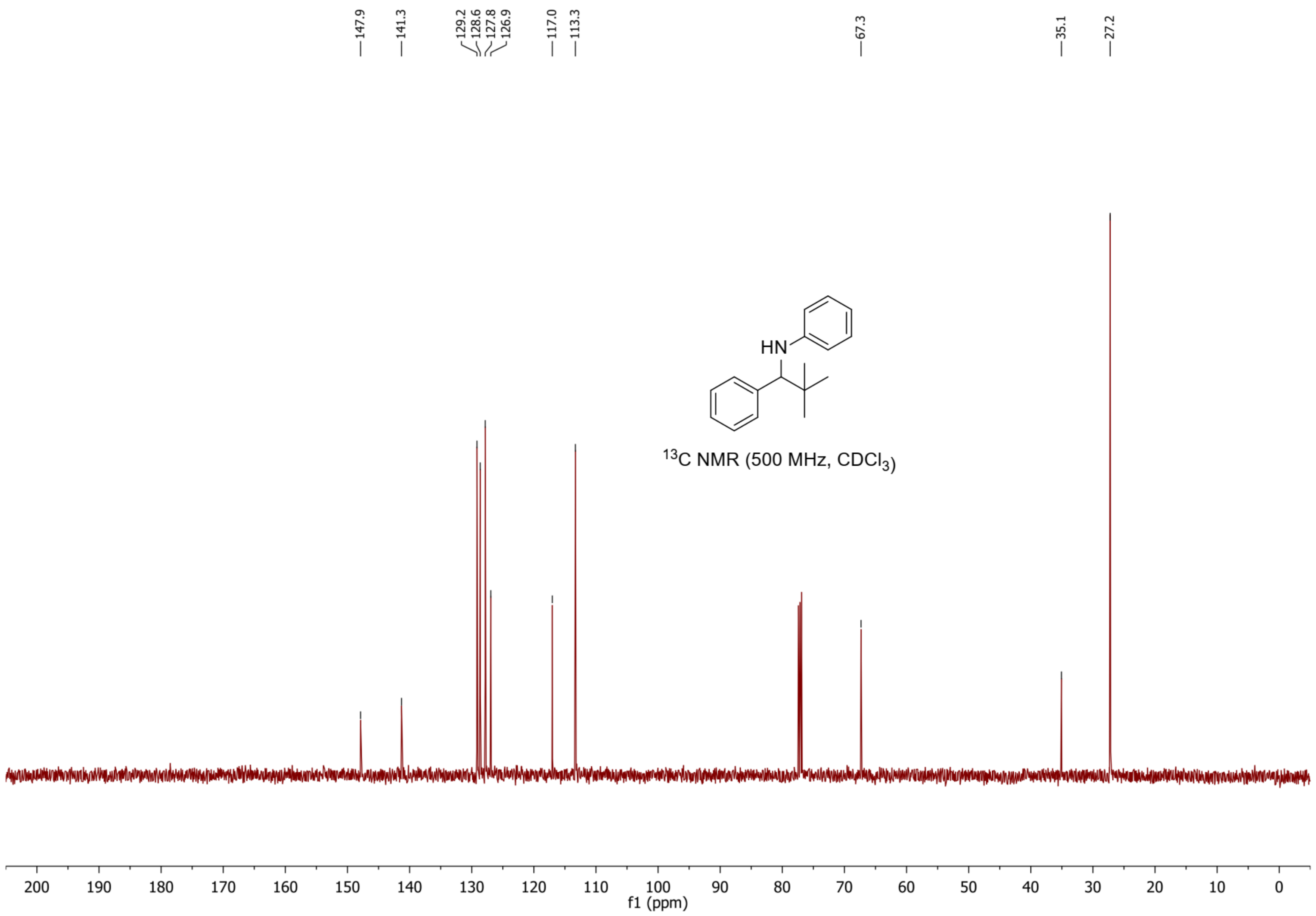

Go to table of contents

N-(2,2-Dimethyl-1-phenylbutyl)aniline (6j)

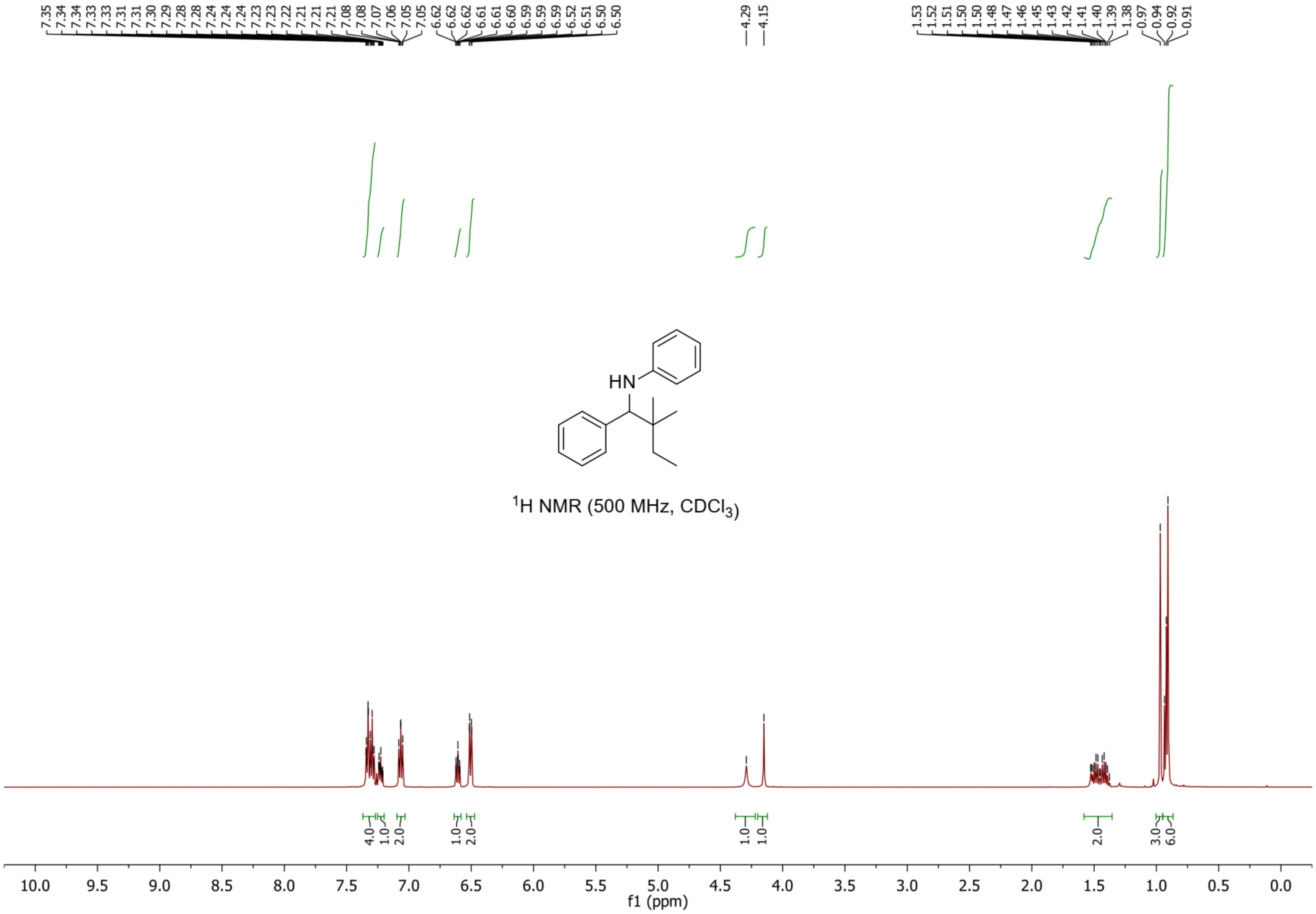

Go to table of contents

N-(2,2-Dimethyl-1-phenylbutyl)aniline (6j)

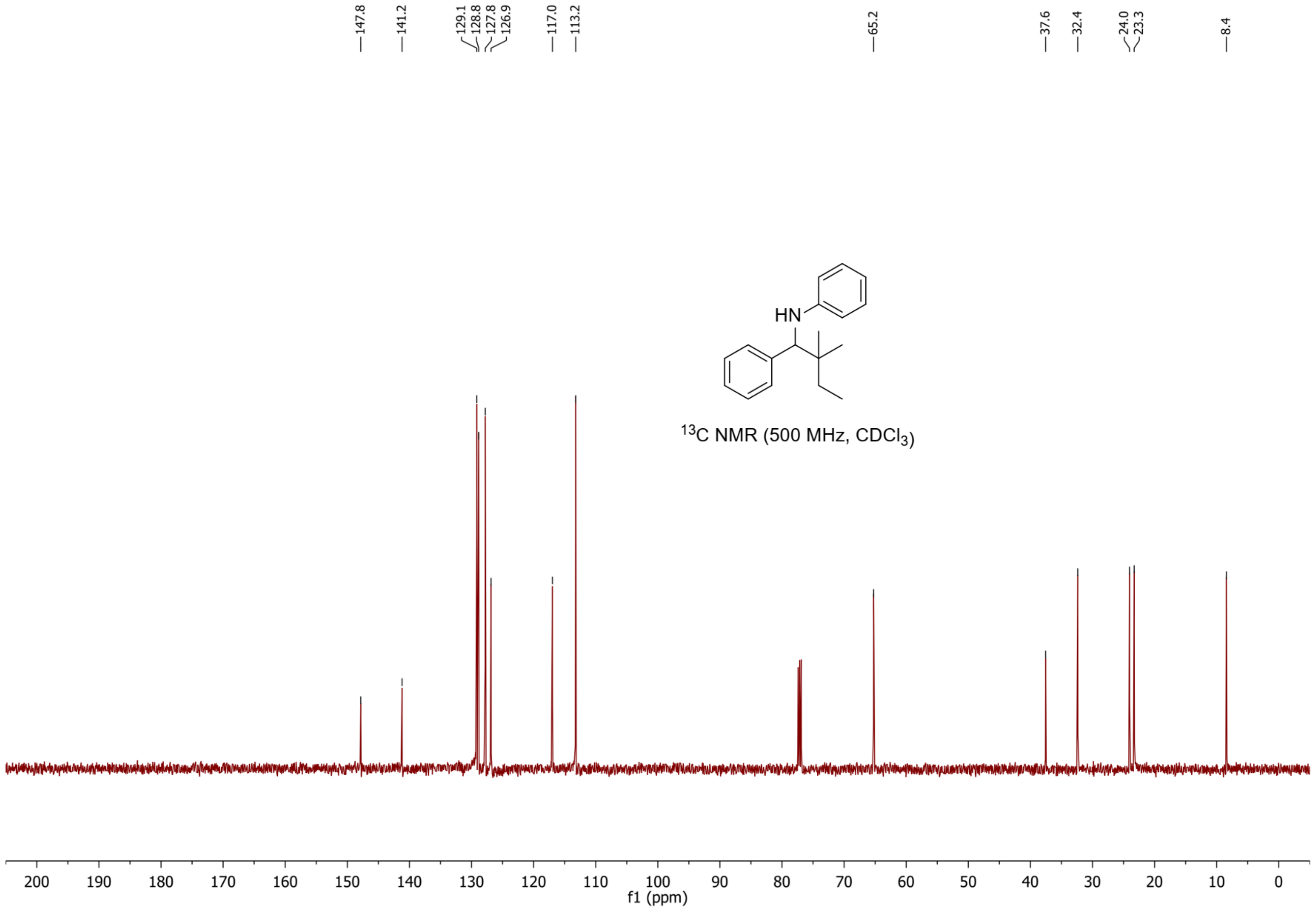

Go to table of contents

N-((1-Methylcyclohexyl)(phenyl)methyl)aniline (6k)

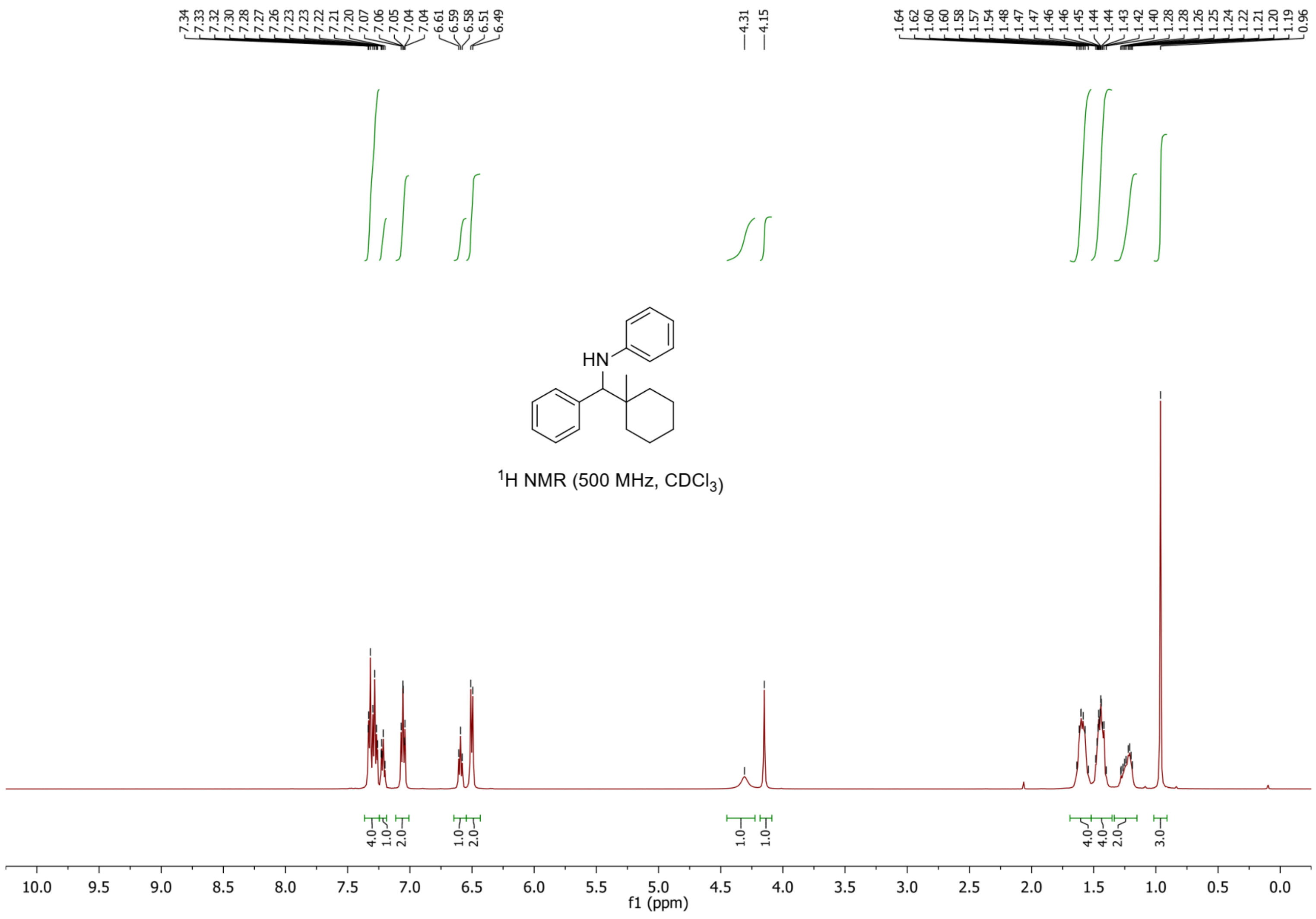

_N_-((1-Methylcyclohexyl)(phenyl)methyl)aniline (6k)

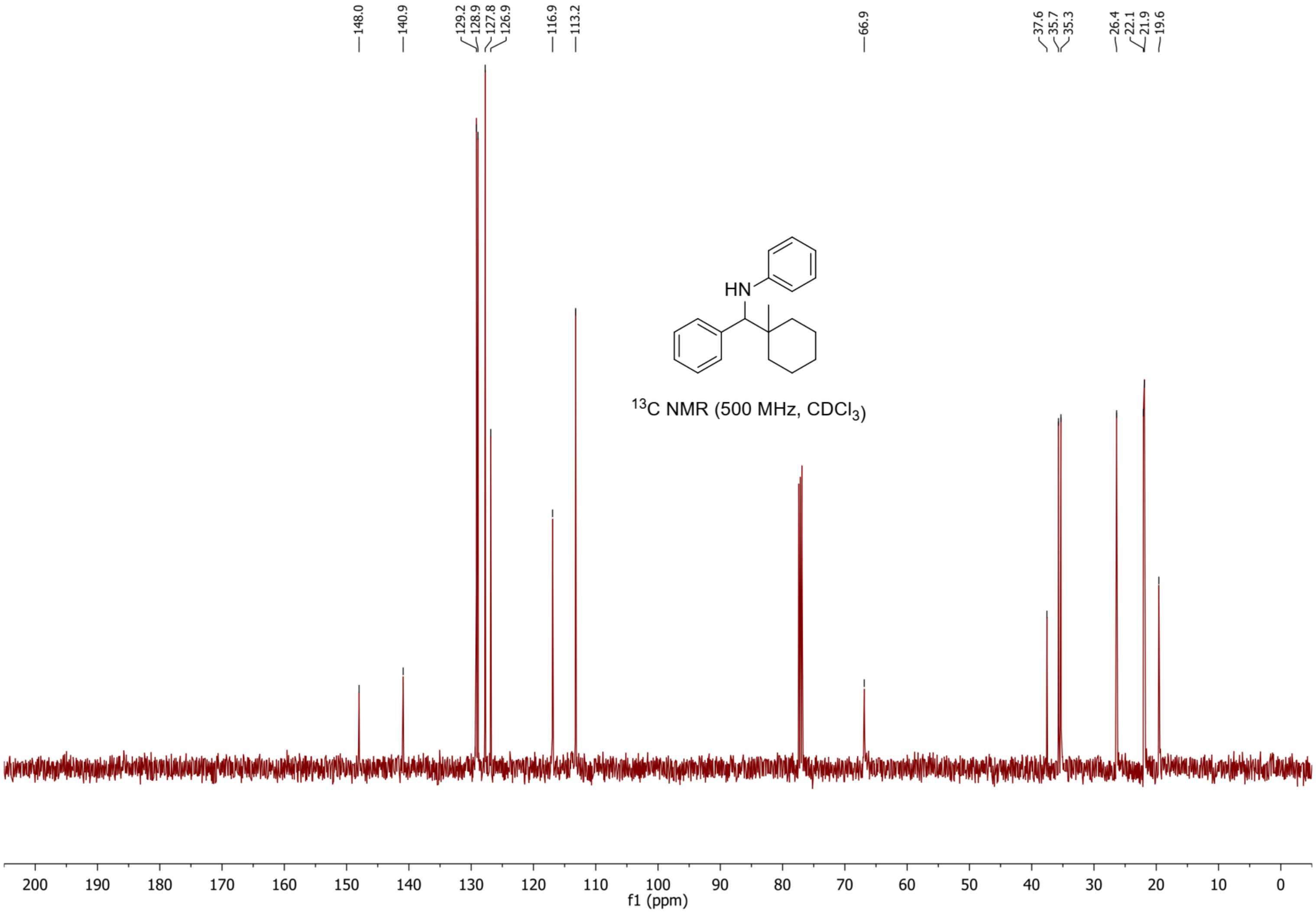

Go to table of contents

_N_-((4-Methyltetrahydro-2_H_-pyran-4-yl)(phenyl)methyl)aniline (6l)

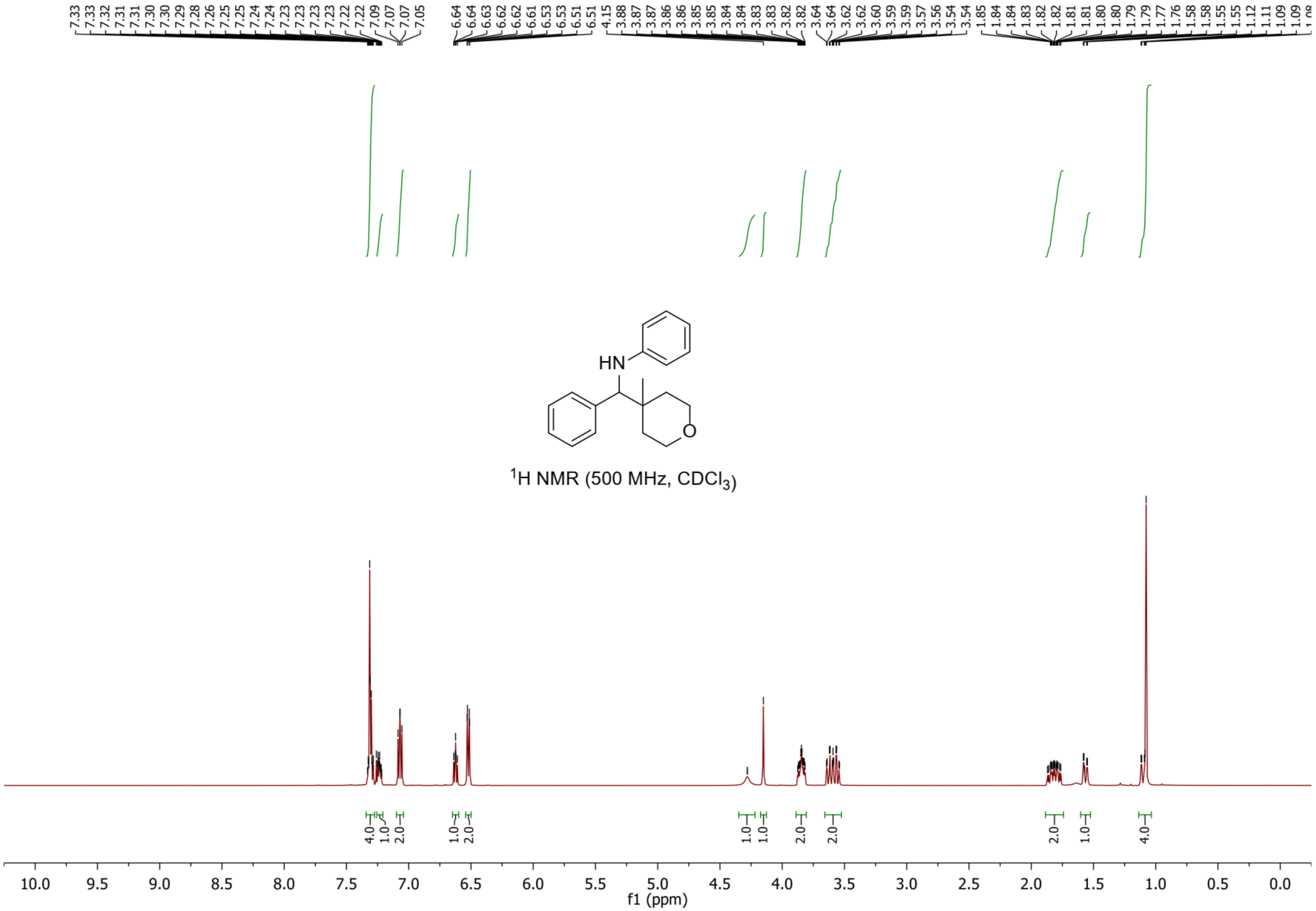

Go to table of contents

N-((4-Methyltetrahydro-2H-pyran-4-yl)(phenyl)methyl)aniline (6l)

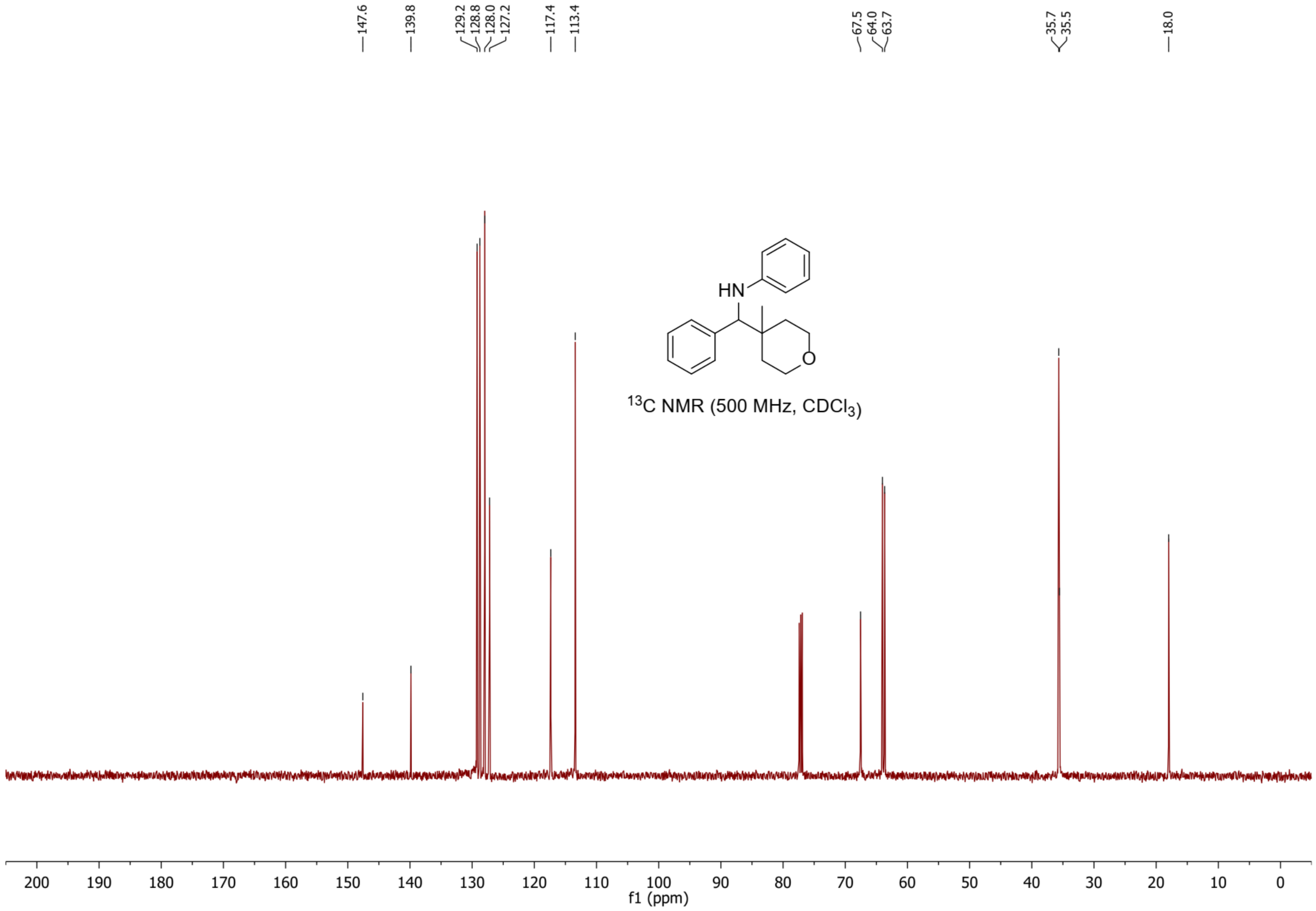

Go to table of contents

N-((Hexahydro-2,5-methanopentalen-3a(1H)-yl)(phenyl)methyl)aniline (6m)

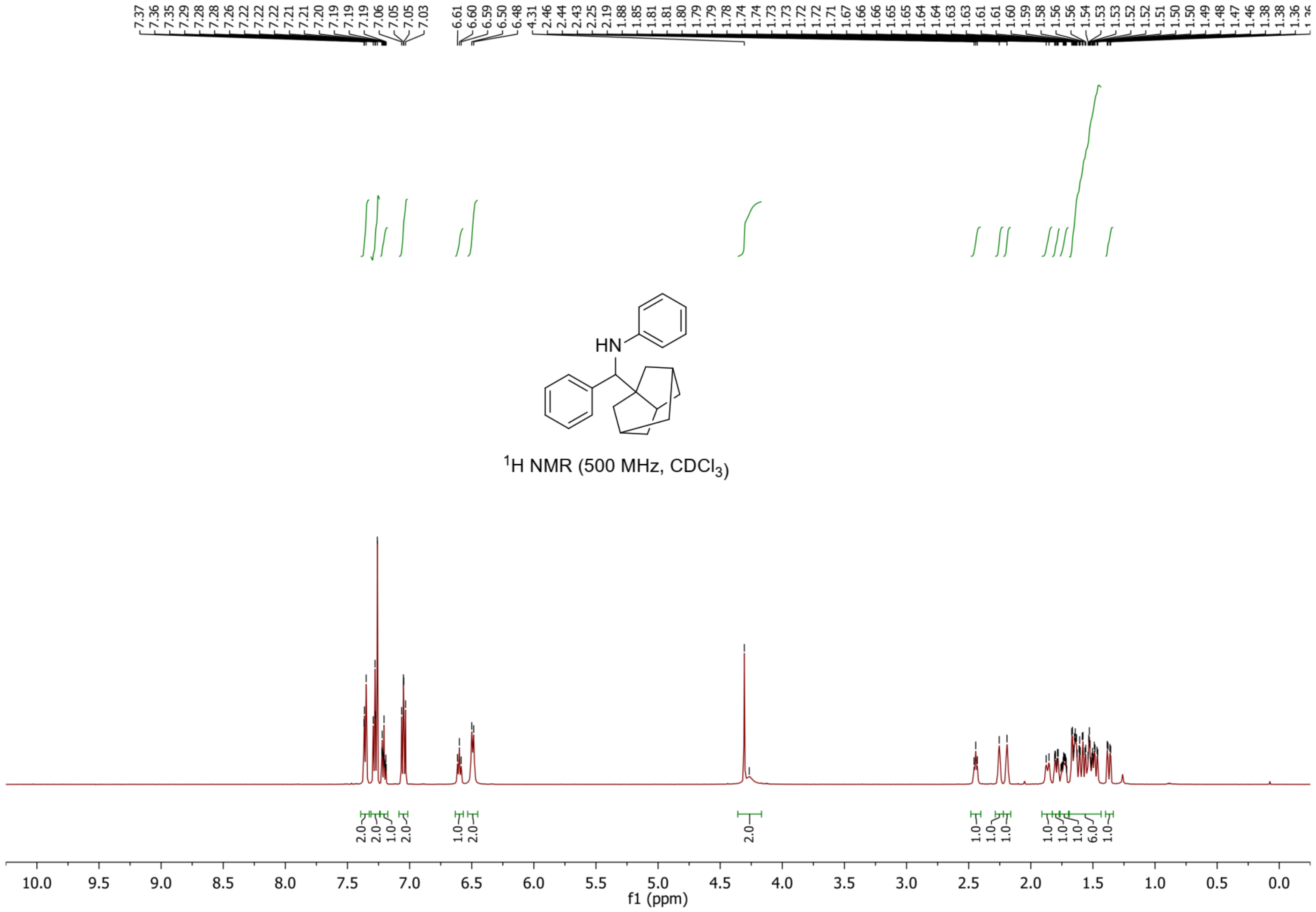

Go to table of contents

N-((Hexahydro-2,5-methanopentalen-3a(1H)-yl)(phenyl)methyl)aniline (6m)

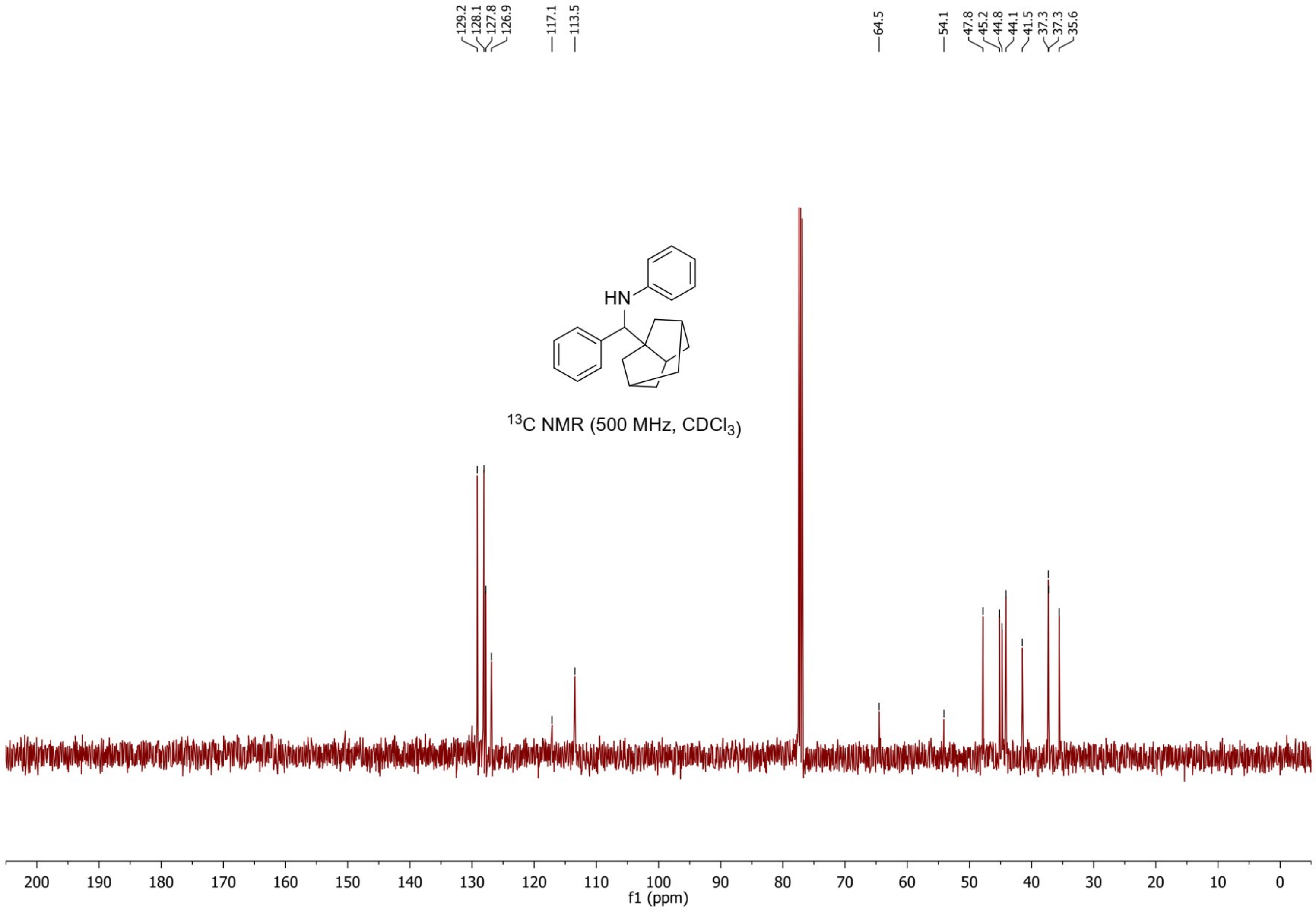

Go to table of contents

_N_-(((1_s_,3_s_)-Adamantan-1-yl)(phenyl)methyl)aniline (6n)

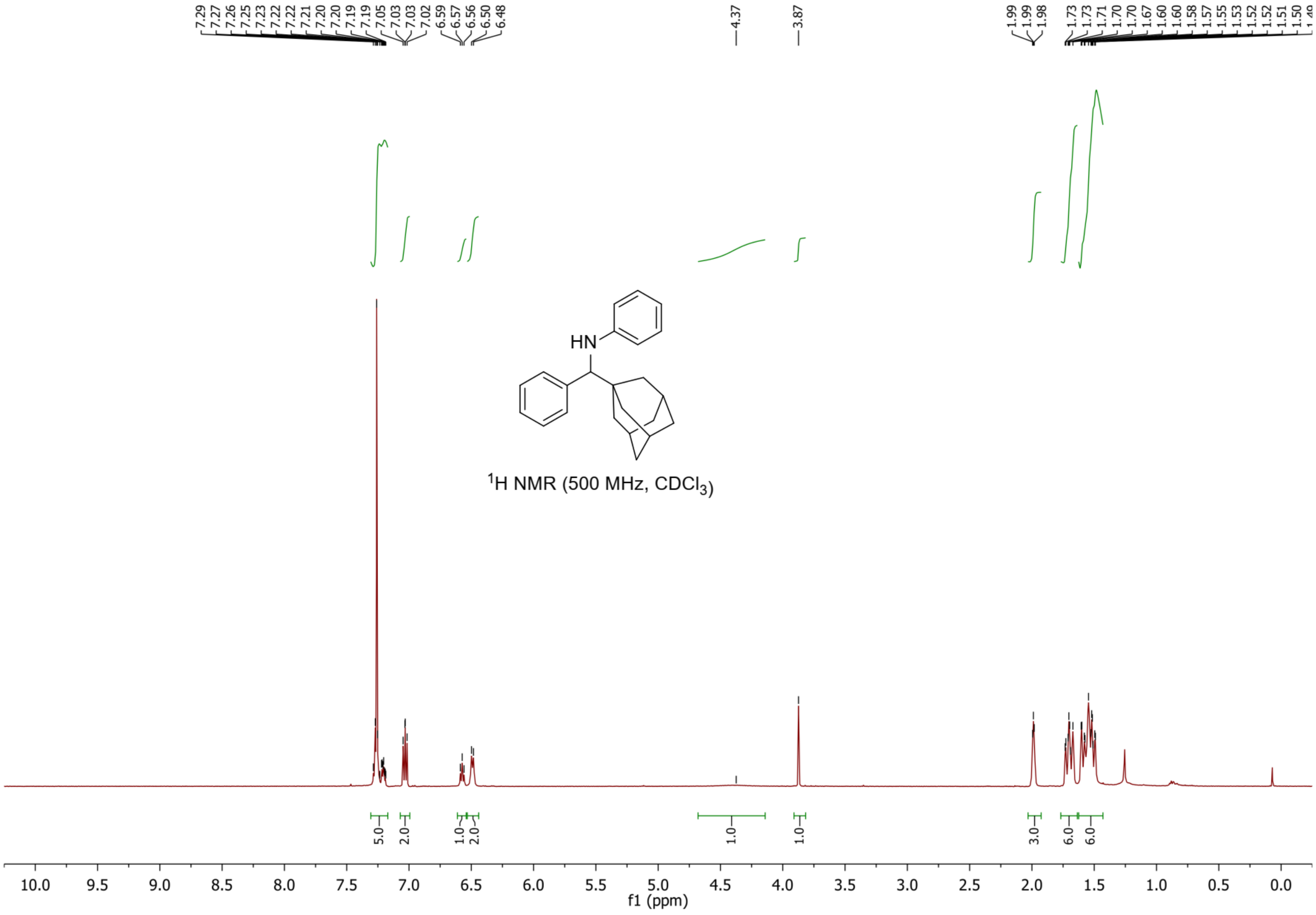

N-(((1*s*,3*s*)-Adamantan-1-yl)(phenyl)methyl)aniline (6n)

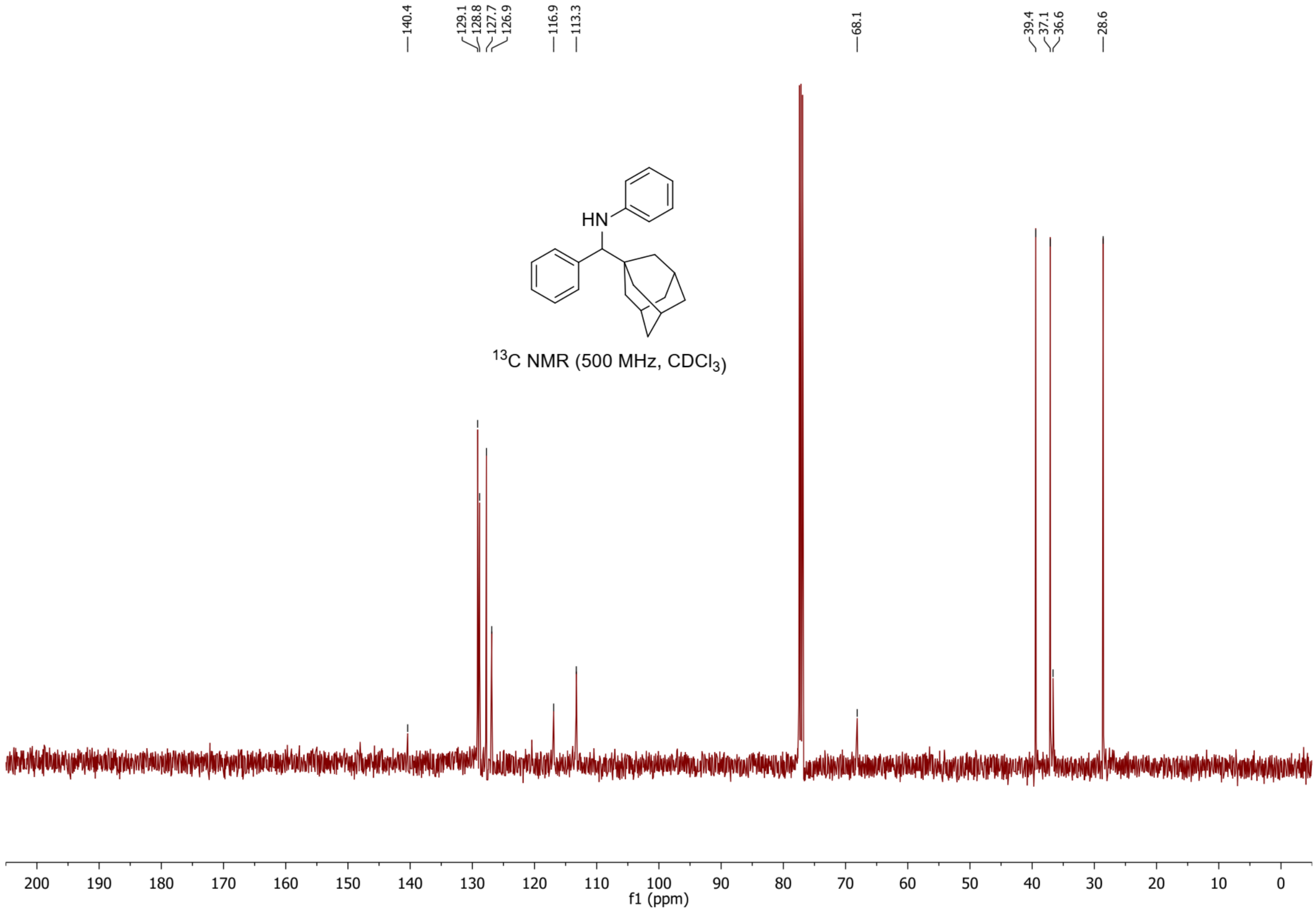

Go to table of contents

Ethyl 2-((4-methoxyphenyl)amino)hexanoate (7a)

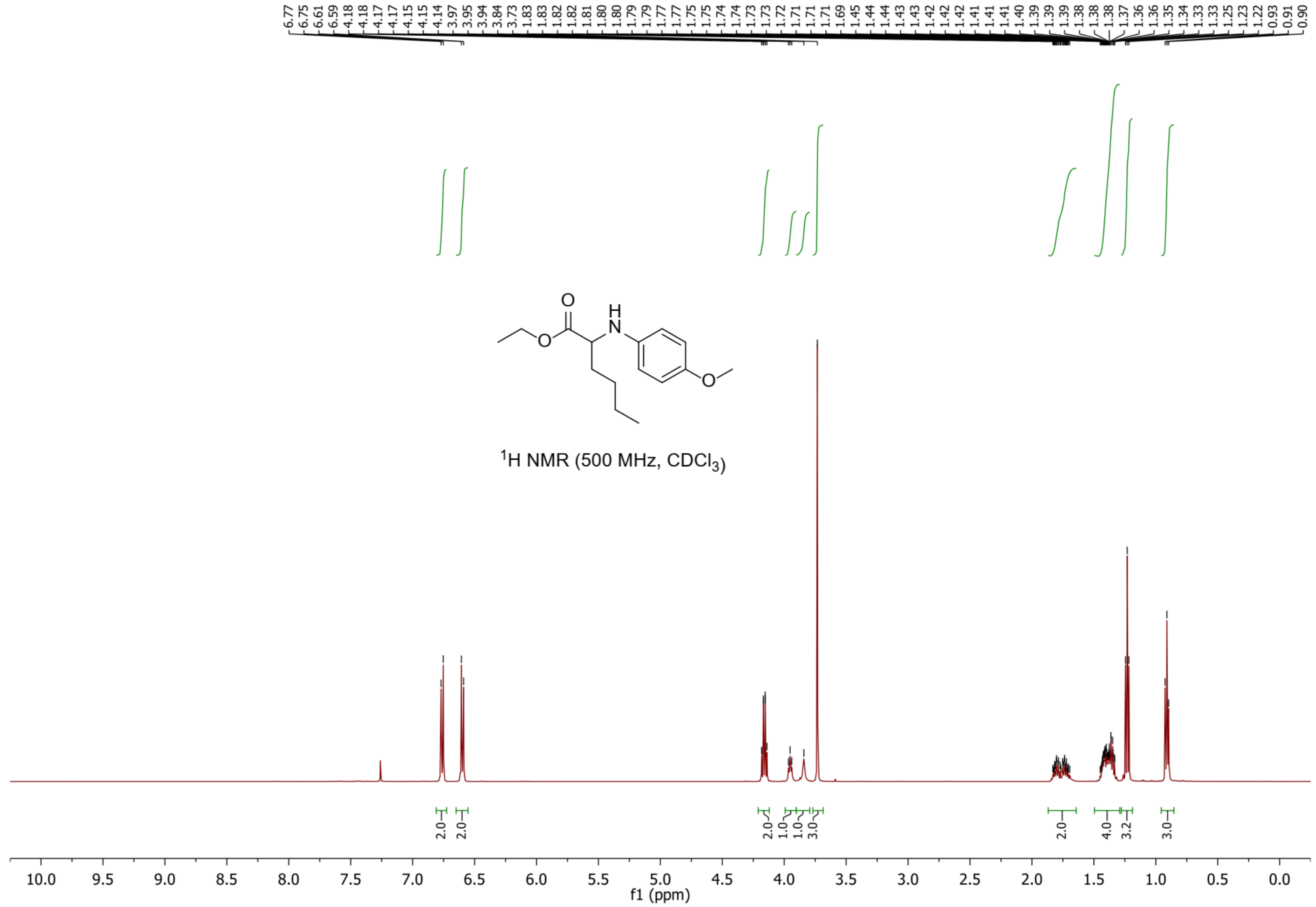

Go to table of contents

Ethyl 2-((4-methoxyphenyl)amino)hexanoate (7a)

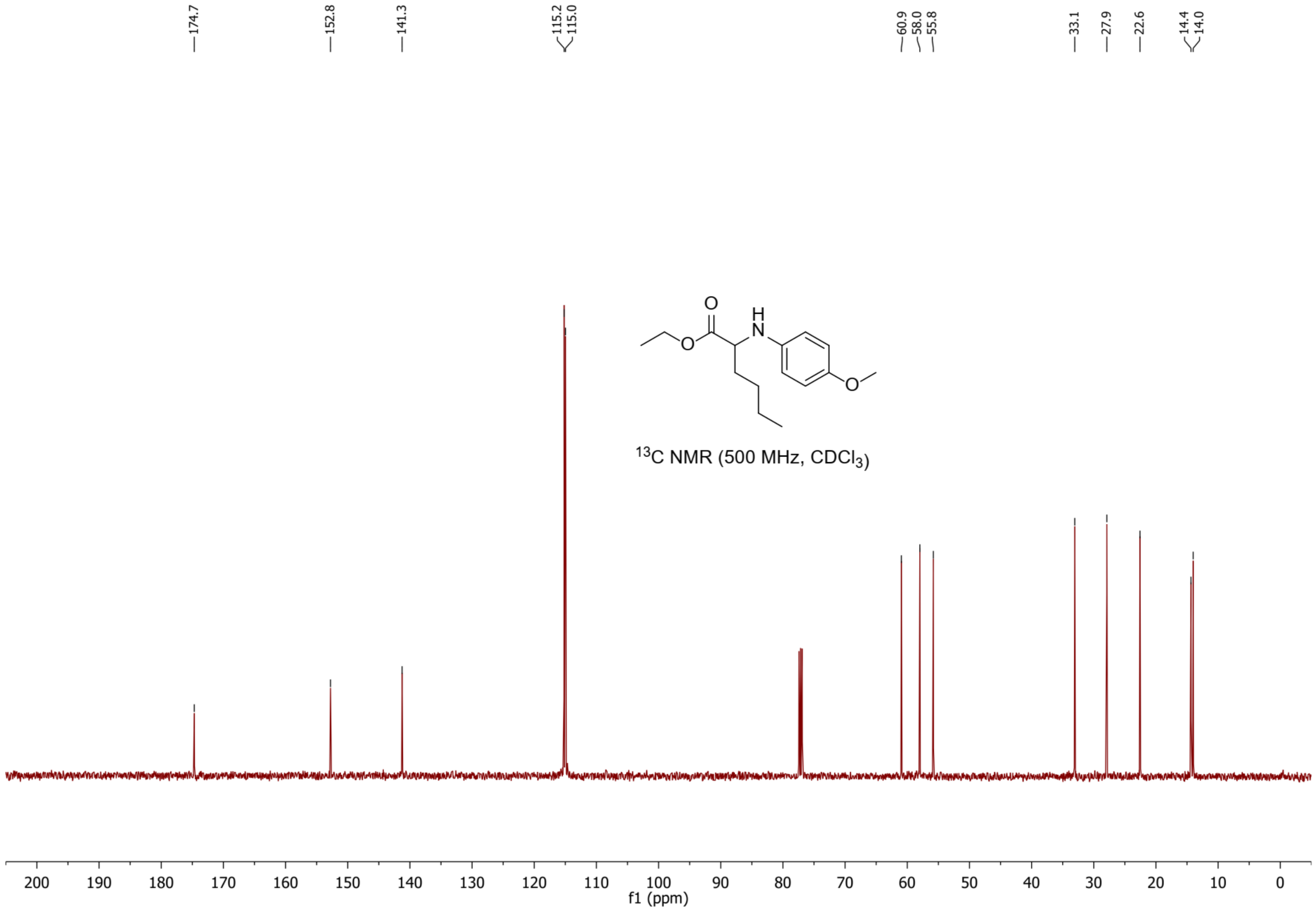

Go to table of contents

Ethyl 2-((4-methoxyphenyl)amino)-5-methylhexanoate (7b)

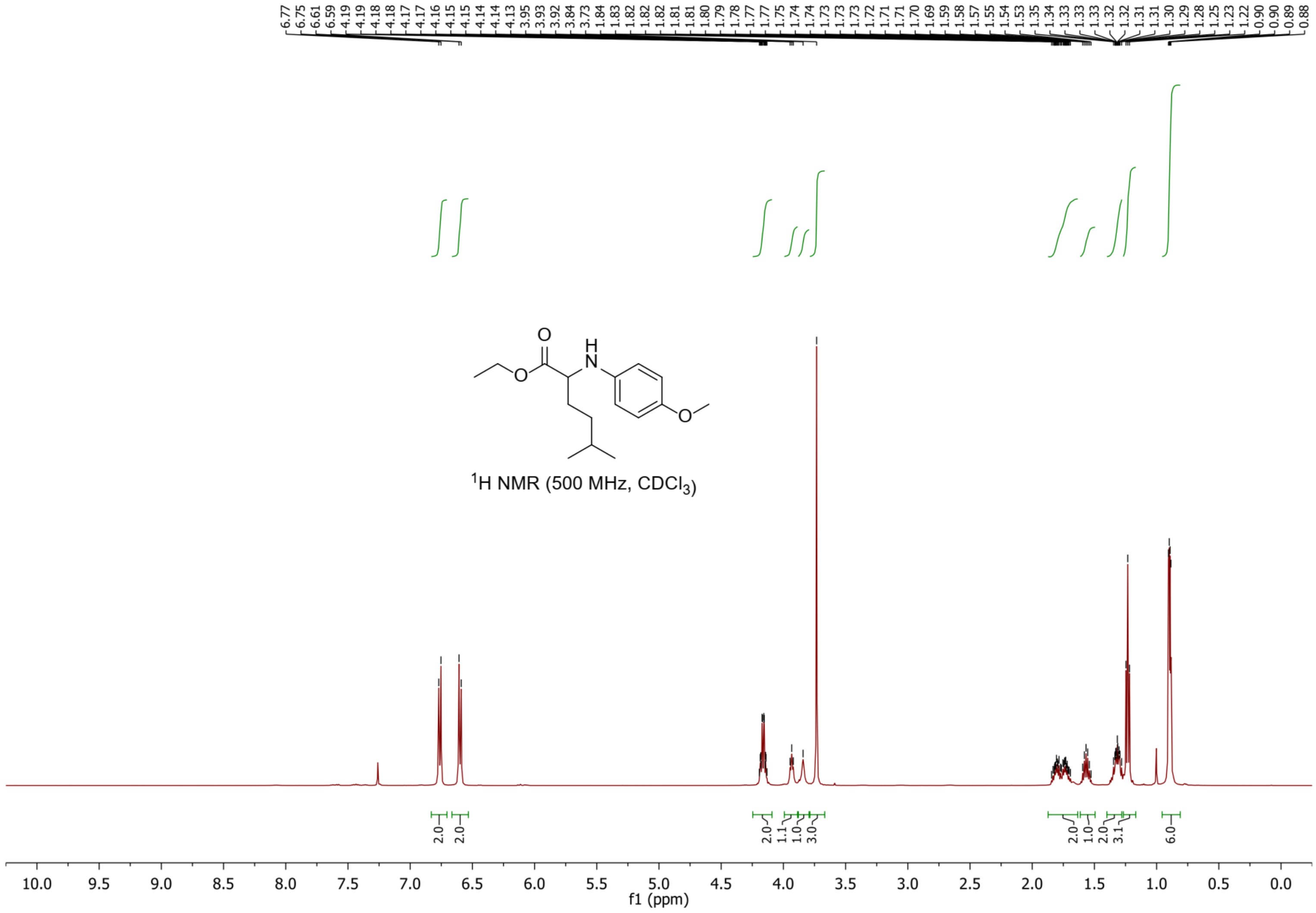

Go to table of contents

Ethyl 2-((4-methoxyphenyl)amino)-5-methylhexanoate (7b)

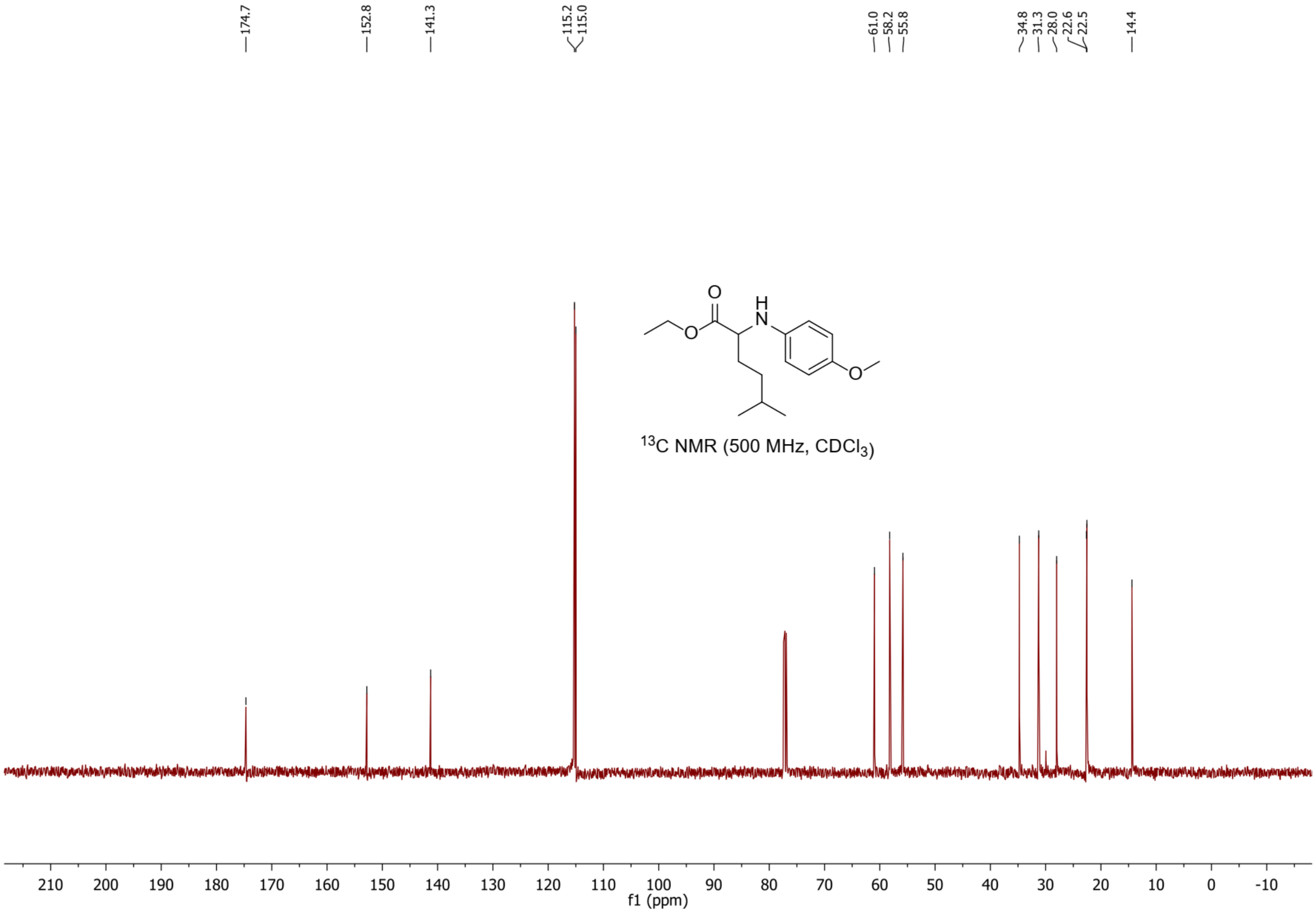

Go to table of contents

Ethyl 2-((4-methoxyphenyl)amino)undecanoate (7c)

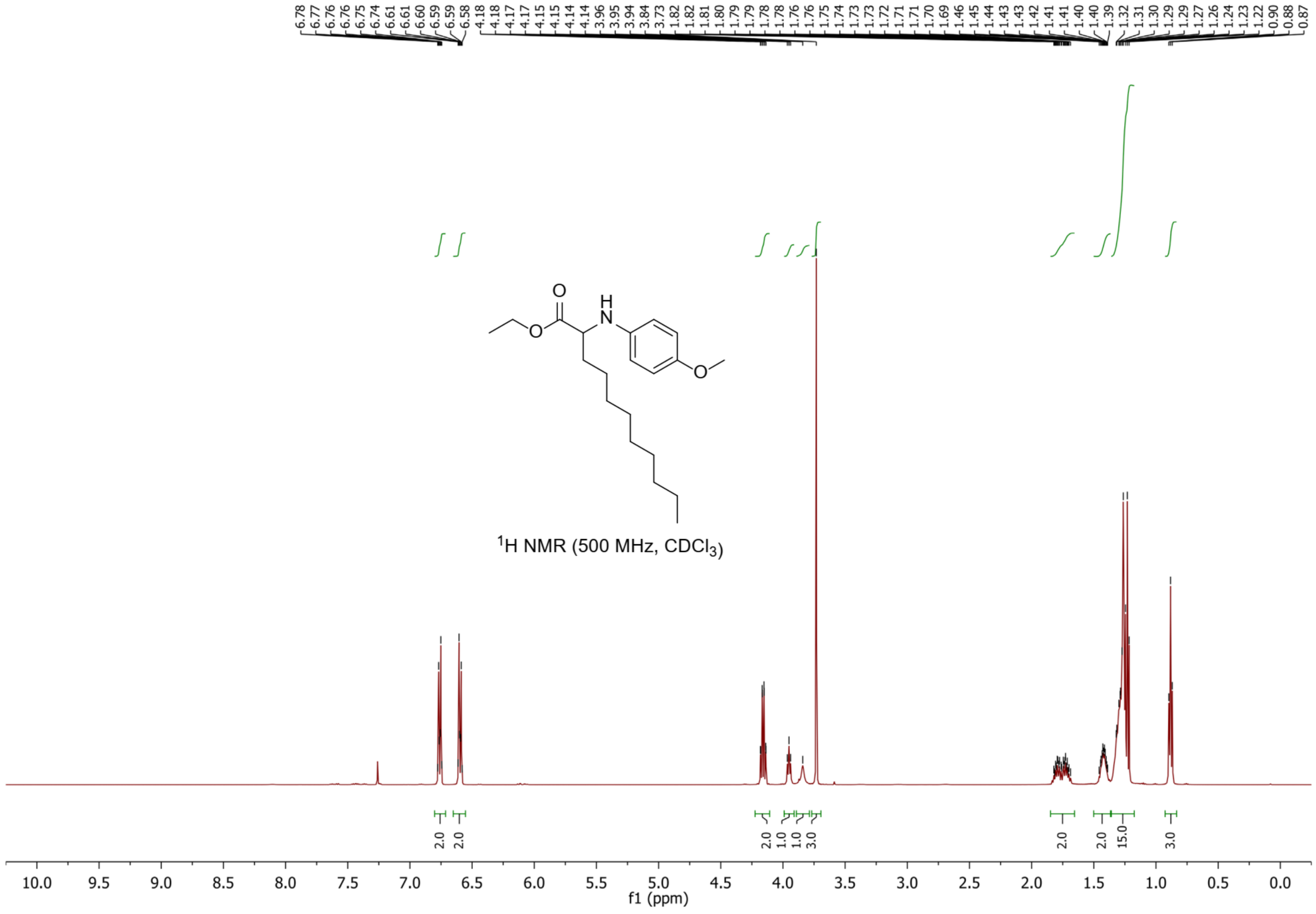

Go to table of contents

Ethyl 2-((4-methoxyphenyl)amino)undecanoate (7c)

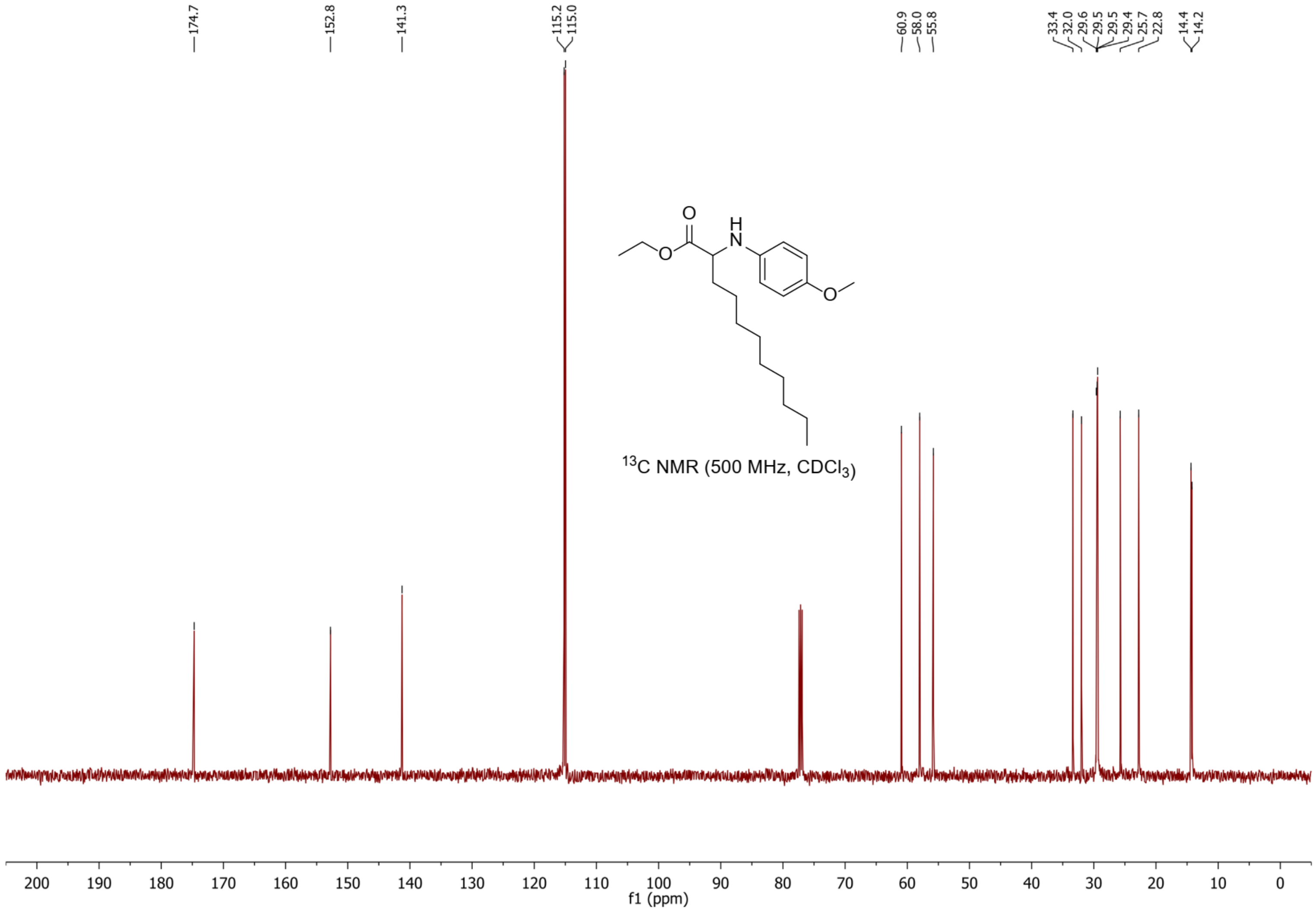

Go to table of contents

Ethyl 5,5,5-trifluoro-2-((4-methoxyphenyl)amino)pentanoate (7d)

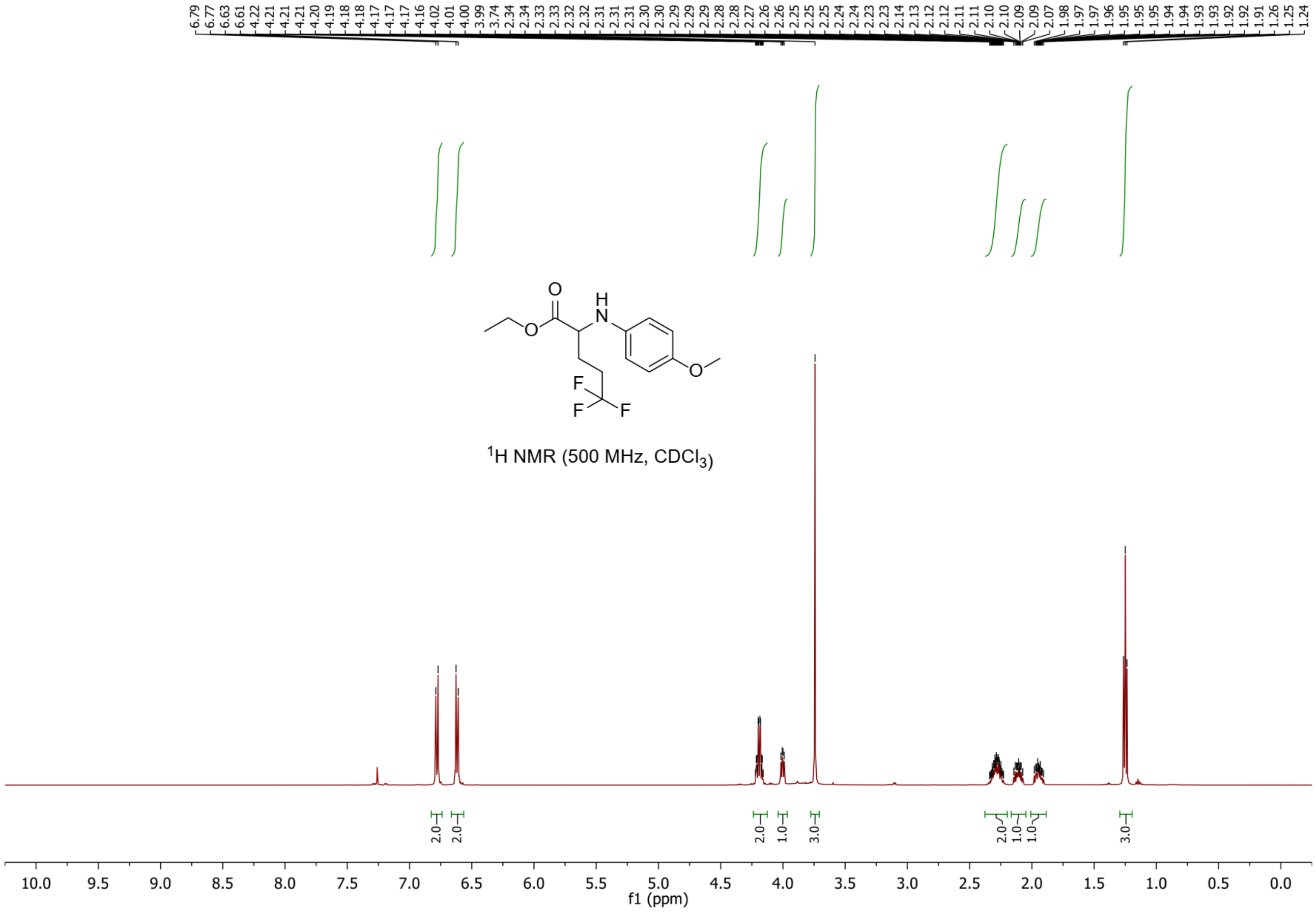

Ethyl 5,5,5-trifluoro-2-((4-methoxyphenyl)amino)pentanoate (7d)

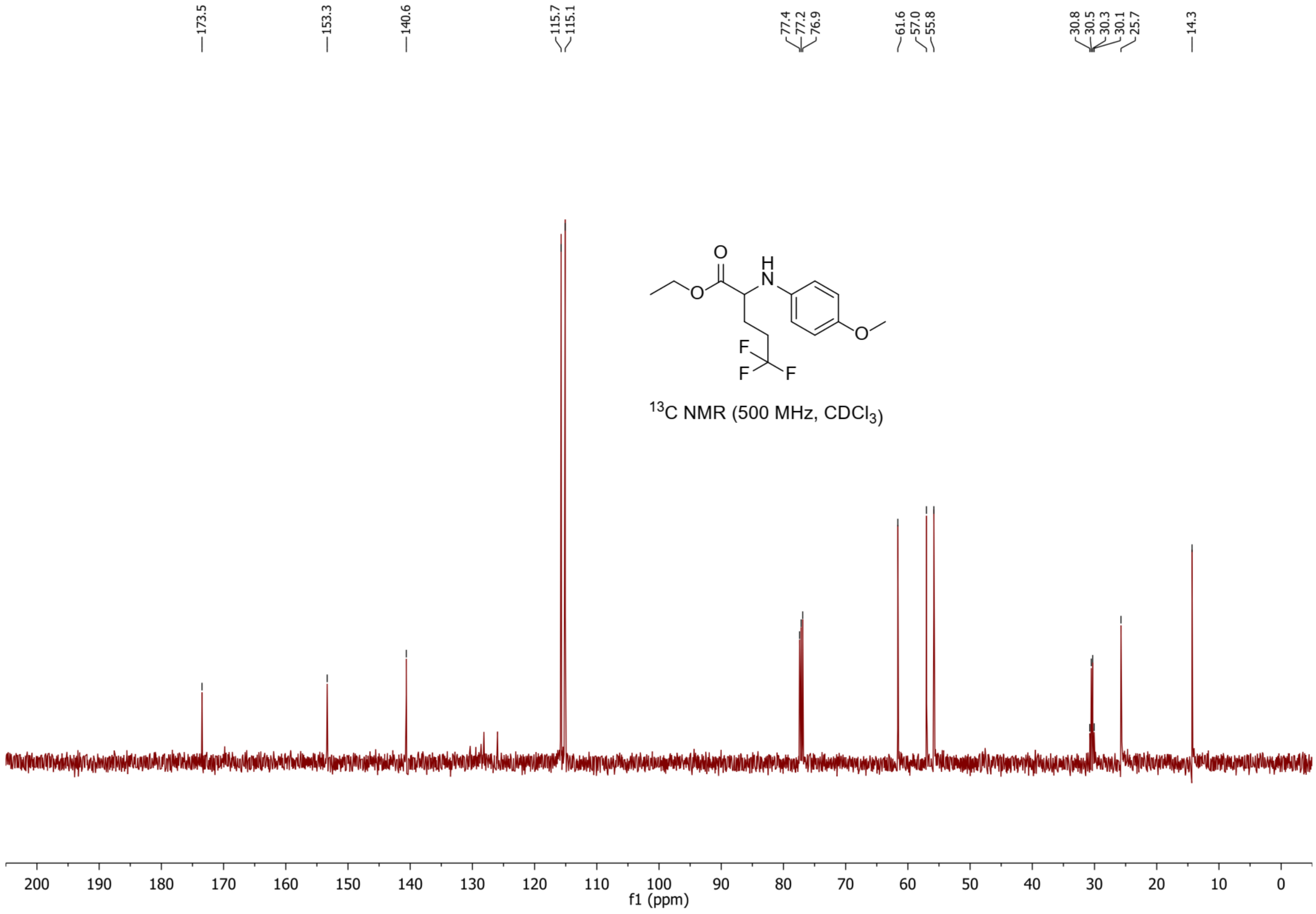

Ethyl 2-((4-methoxyphenyl)amino)-4,4,4-triphenylbutanoate (7e)

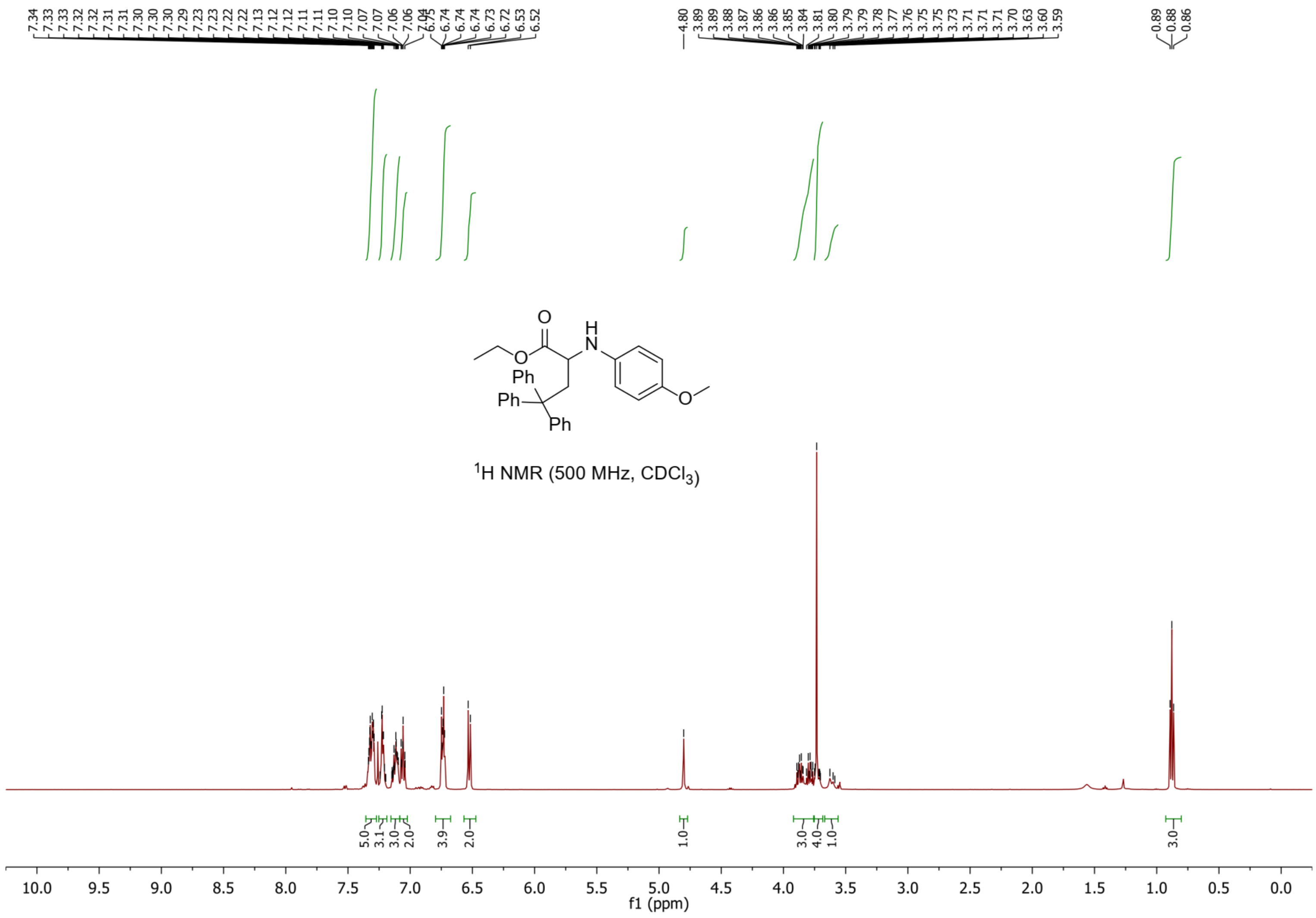

Go to table of contents

Ethyl 2-((4-methoxyphenyl)amino)-4,4,4-triphenylbutanoate (7e)

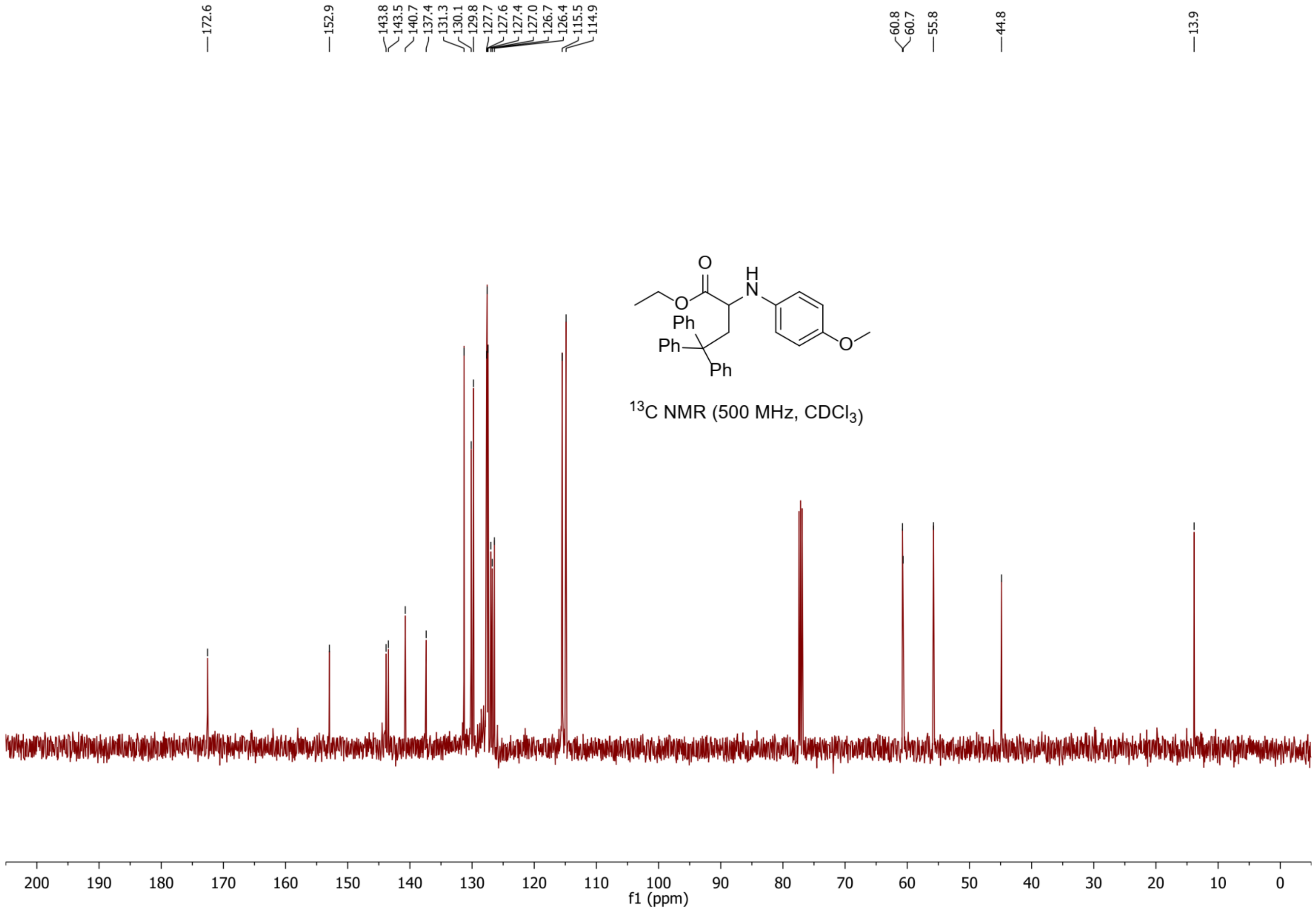

Go to table of contents

Ethyl 3-(4-chlorophenyl)-2-((4-methoxyphenyl)amino)propanoate (7f)

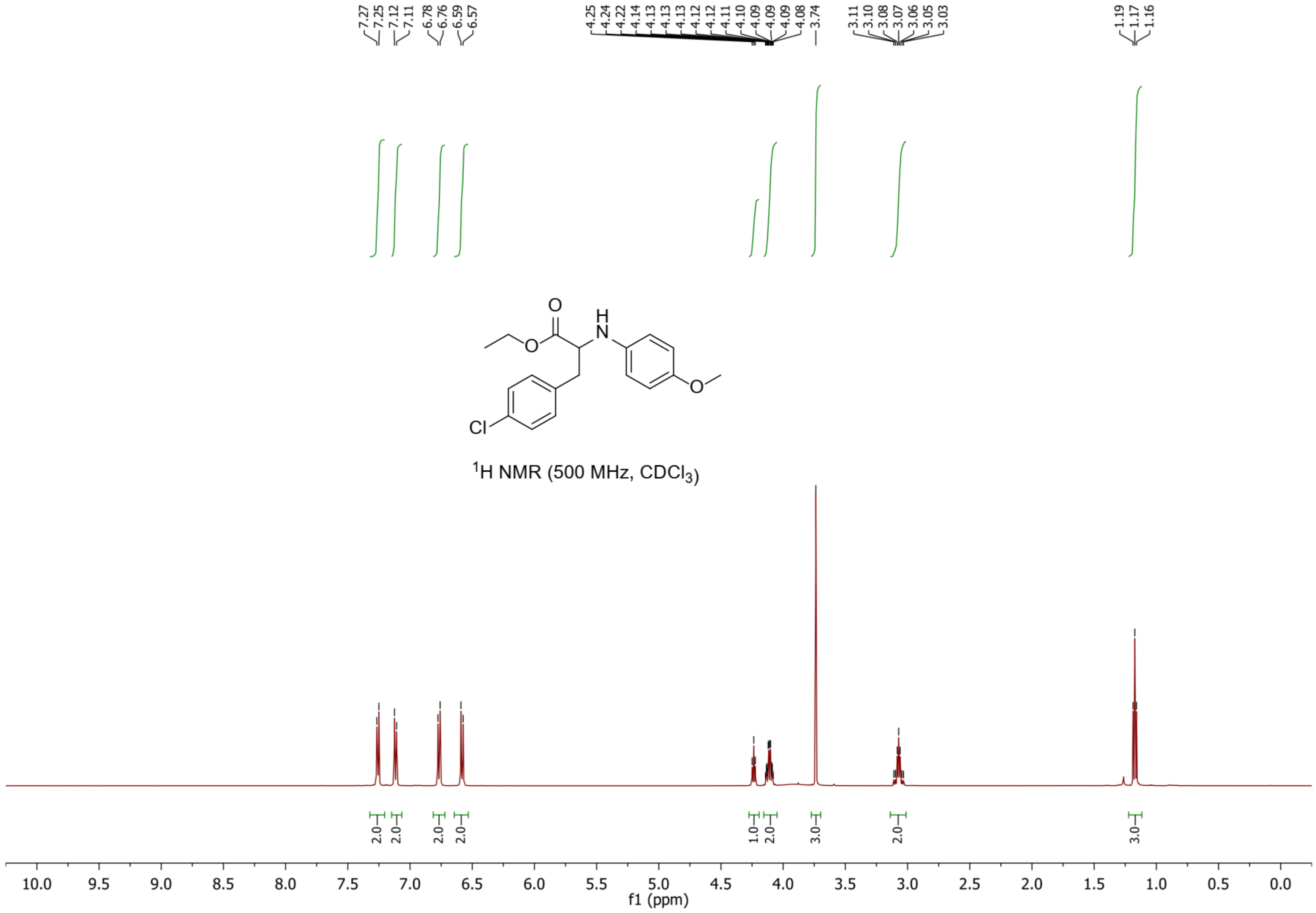

Go to table of contents

Ethyl 3-(4-chlorophenyl)-2-((4-methoxyphenyl)amino)propanoate (7f)

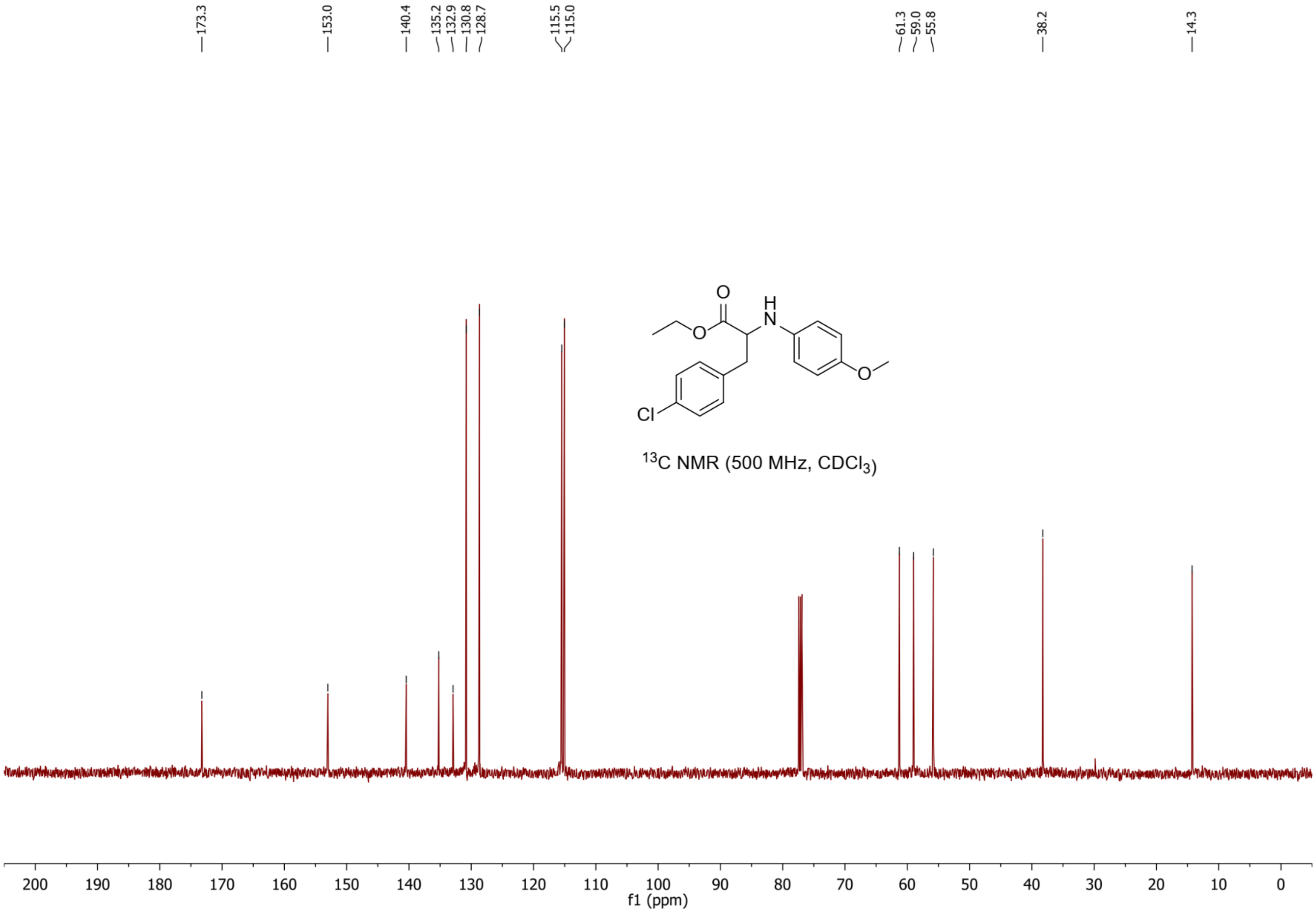

Go to table of contents

Ethyl 4-(4-fluorophenyl)-2-((4-methoxyphenyl)amino)butanoate (7g)

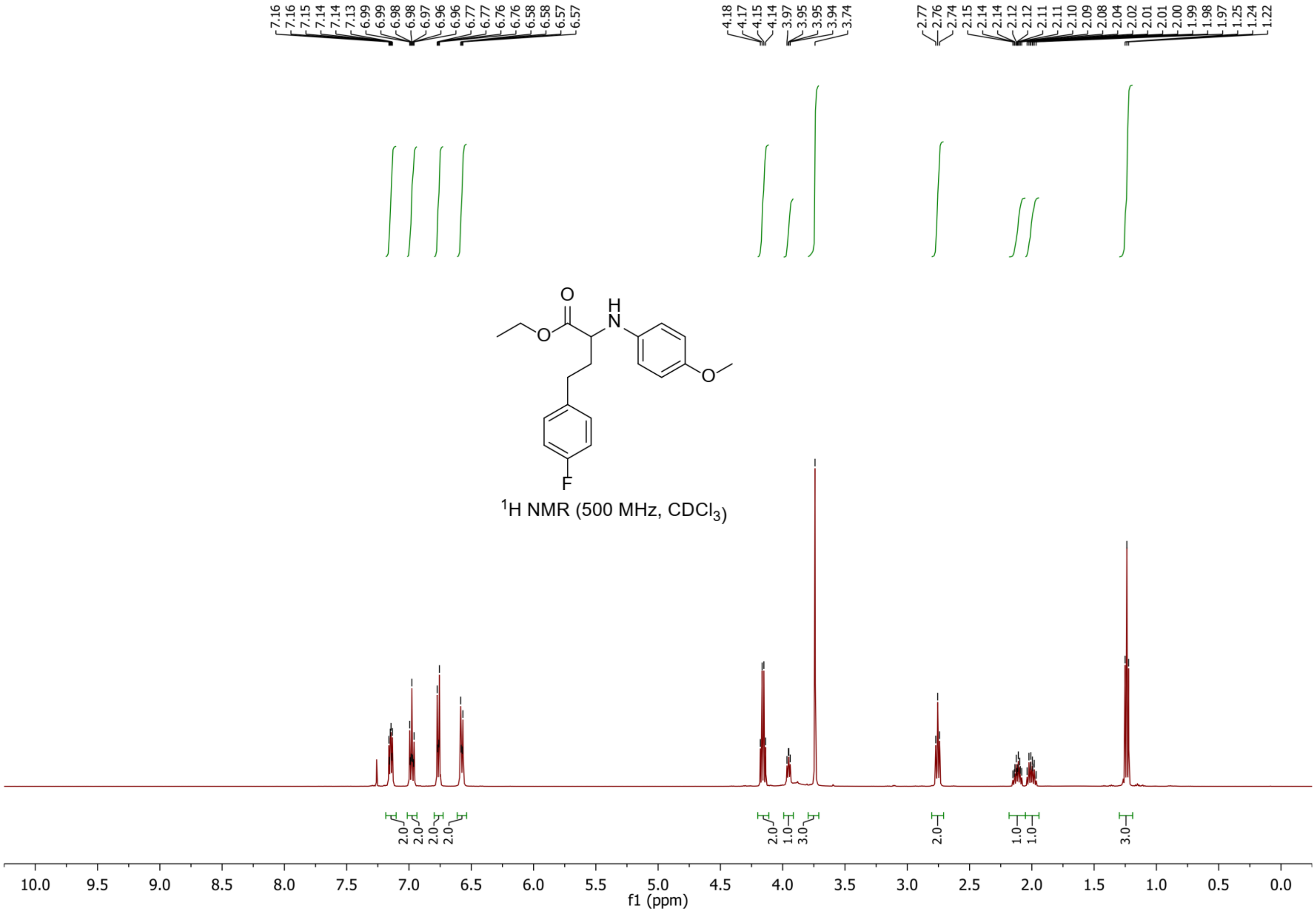

Go to table of contents

Ethyl 4-(4-fluorophenyl)-2-((4-methoxyphenyl)amino)butanoate (7g)

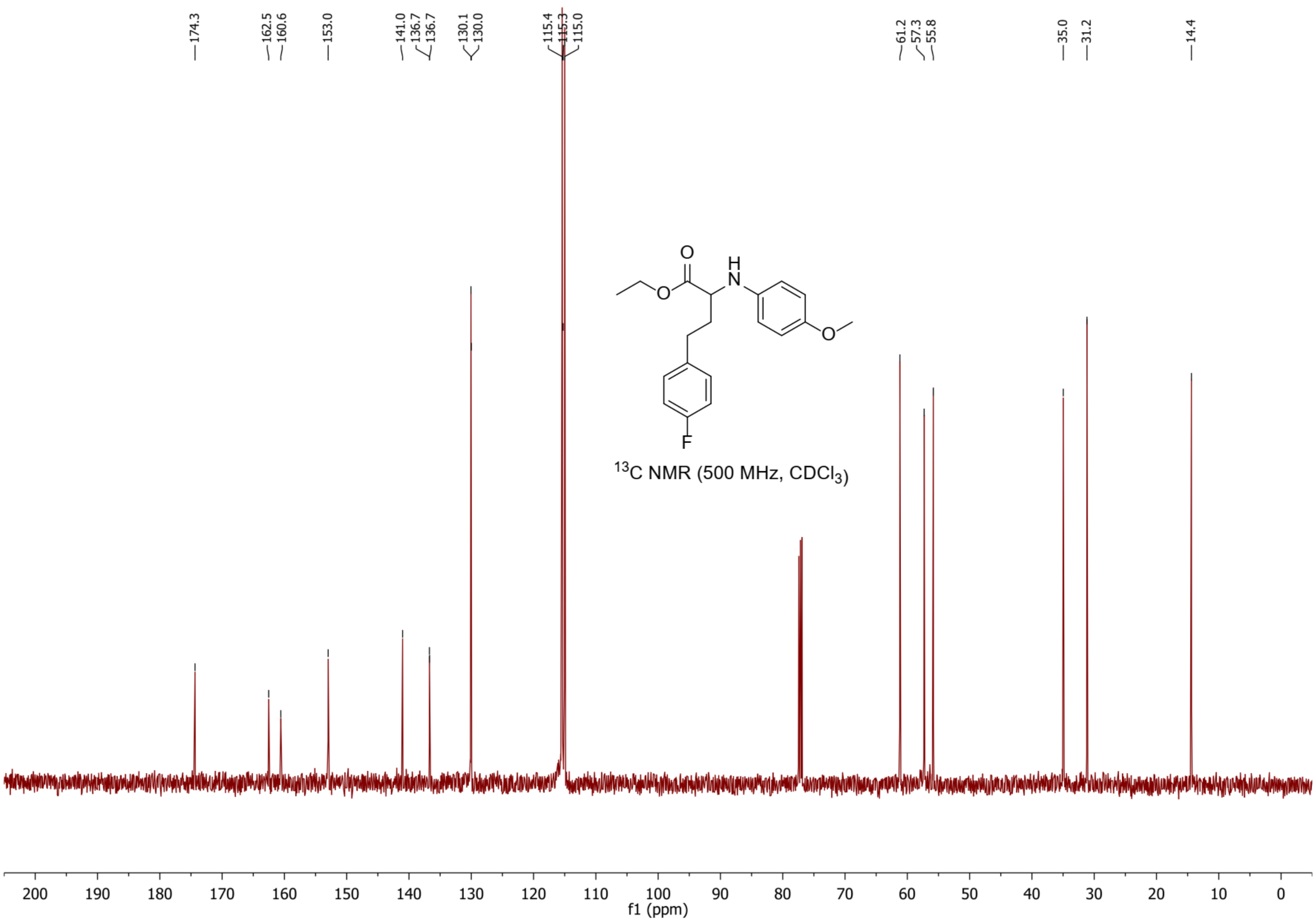

Go to table of contents

Ethyl 2-((4-methoxyphenyl)amino)-5-phenylpentanoate (7h)

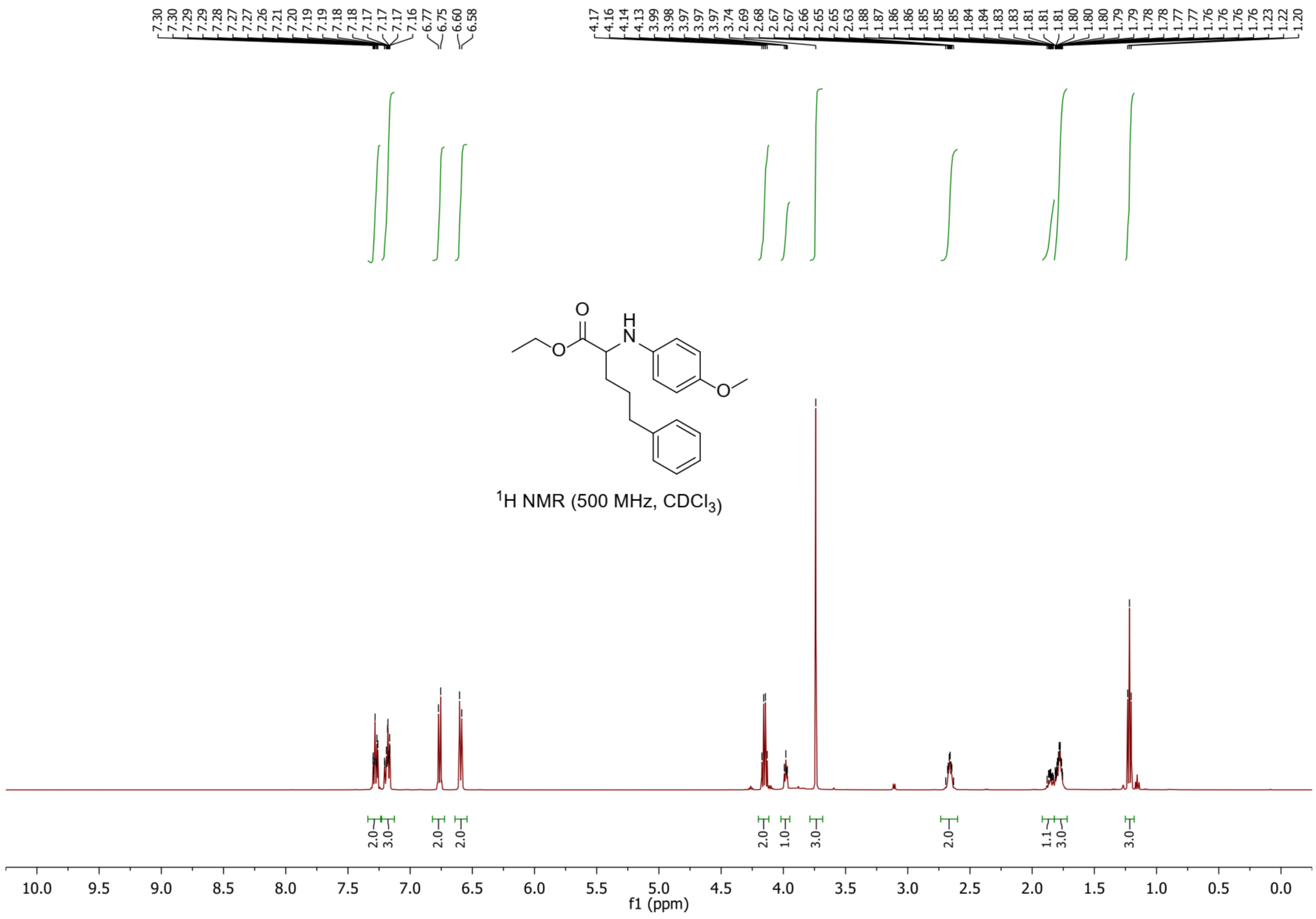

Go to table of contents

Ethyl 2-((4-methoxyphenyl)amino)-5-phenylpentanoate (7h)

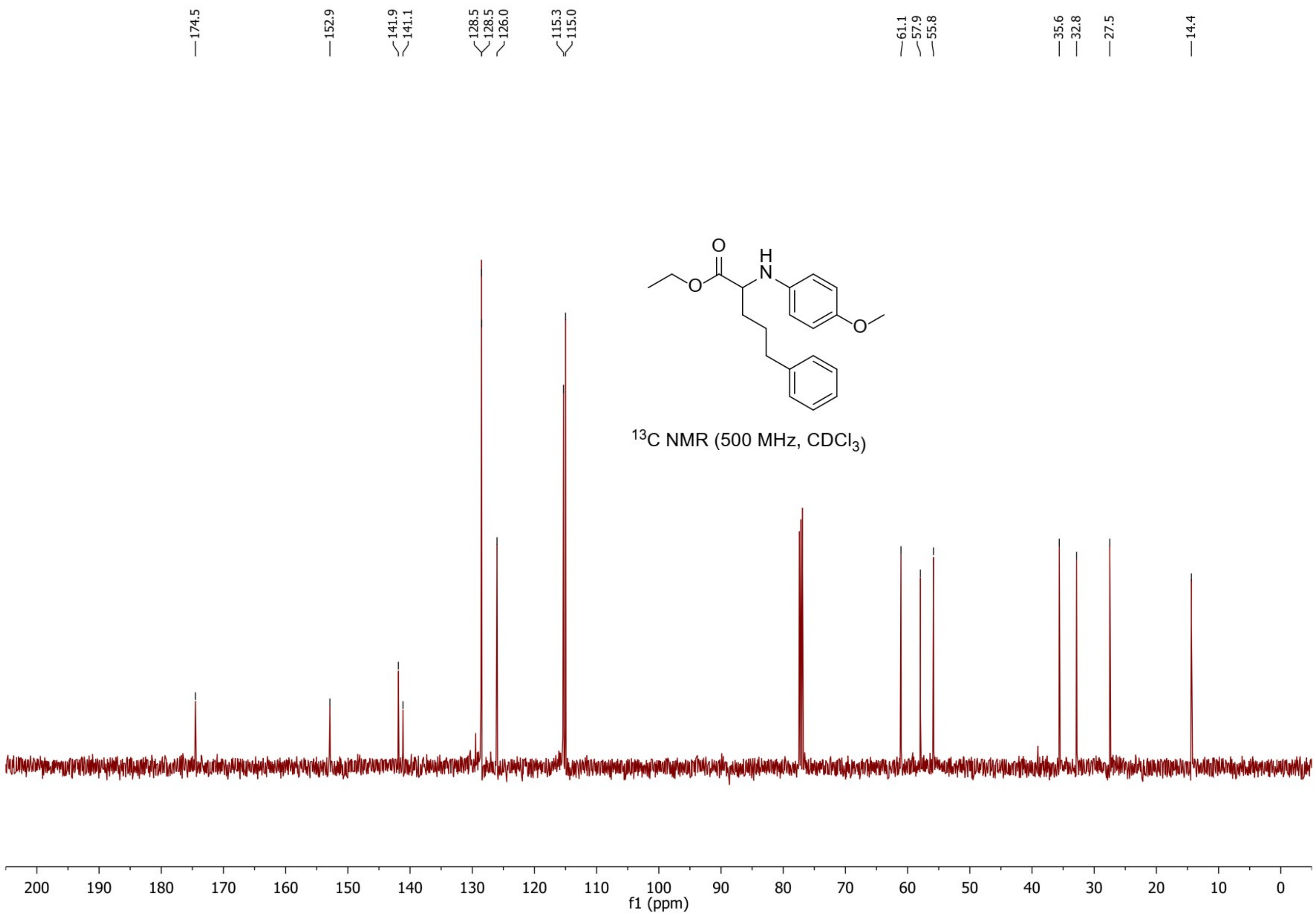

Go to table of contents

Ethyl 2-((4-methoxyphenyl)amino)-6-phenylhexanoate (7i)

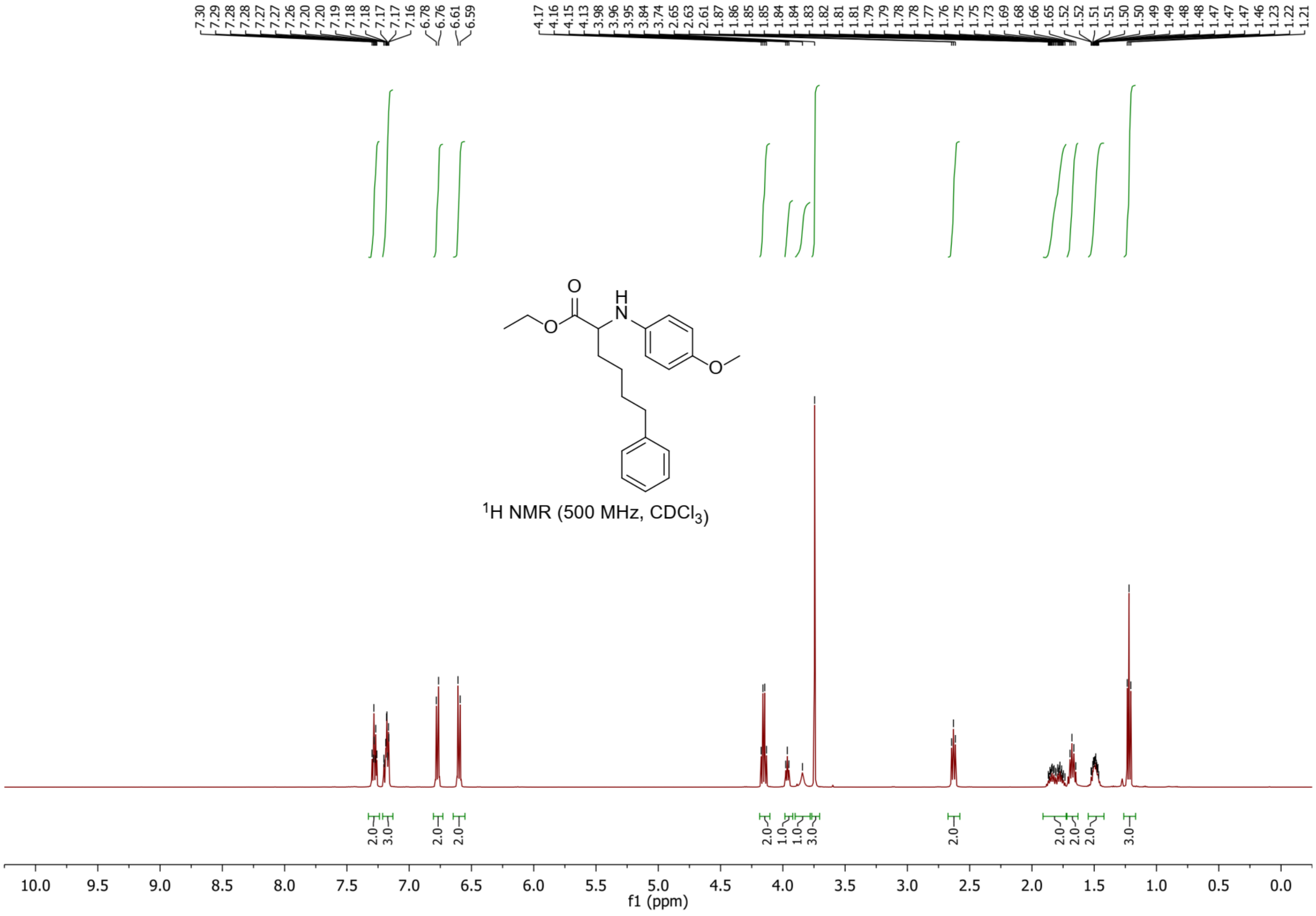

Go to table of contents

Ethyl 2-((4-methoxyphenyl)amino)-6-phenylhexanoate (7i)

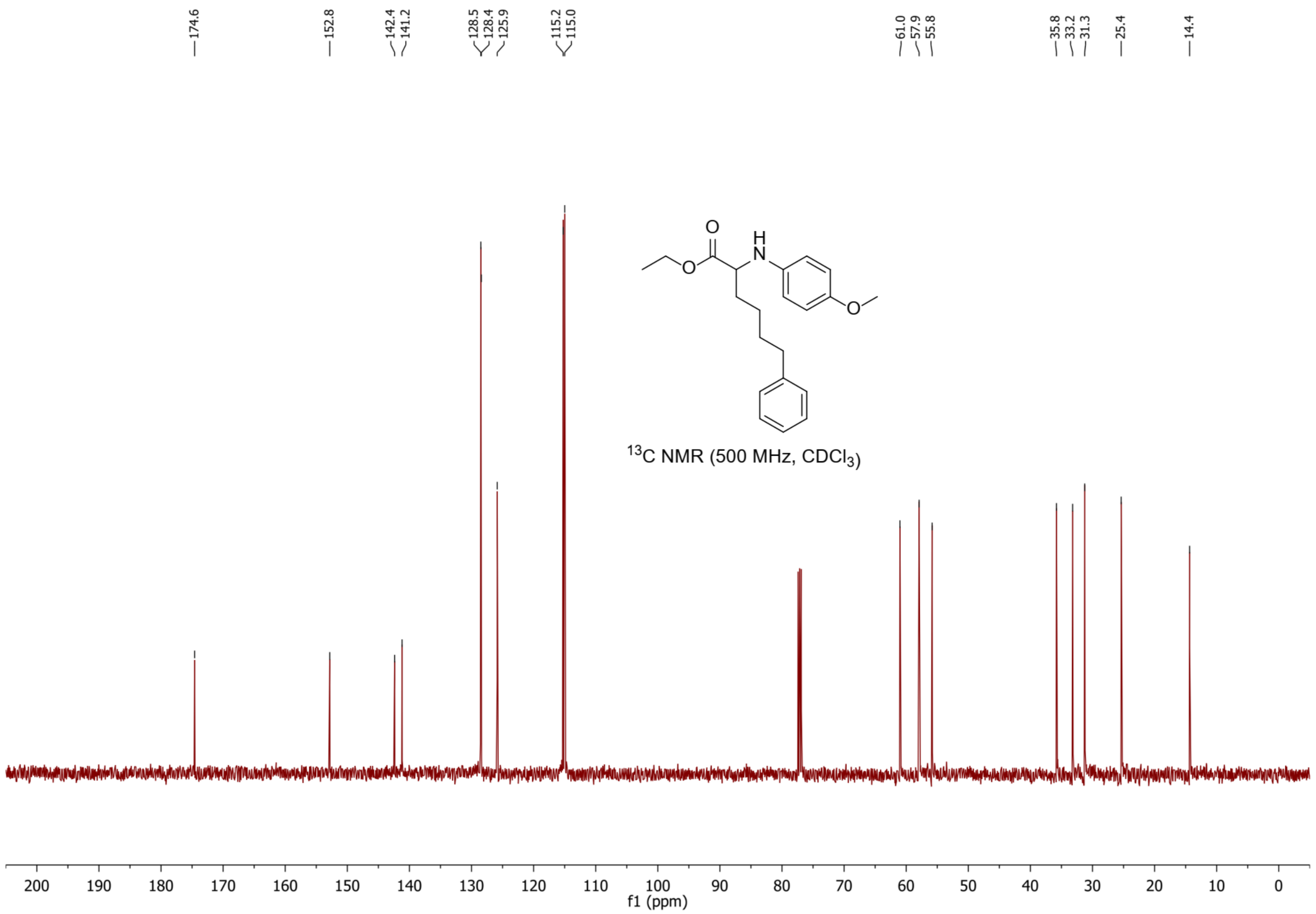

Go to table of contents

Ethyl 2-cyclopentyl-2-((4-methoxyphenyl)amino)acetate (7j)

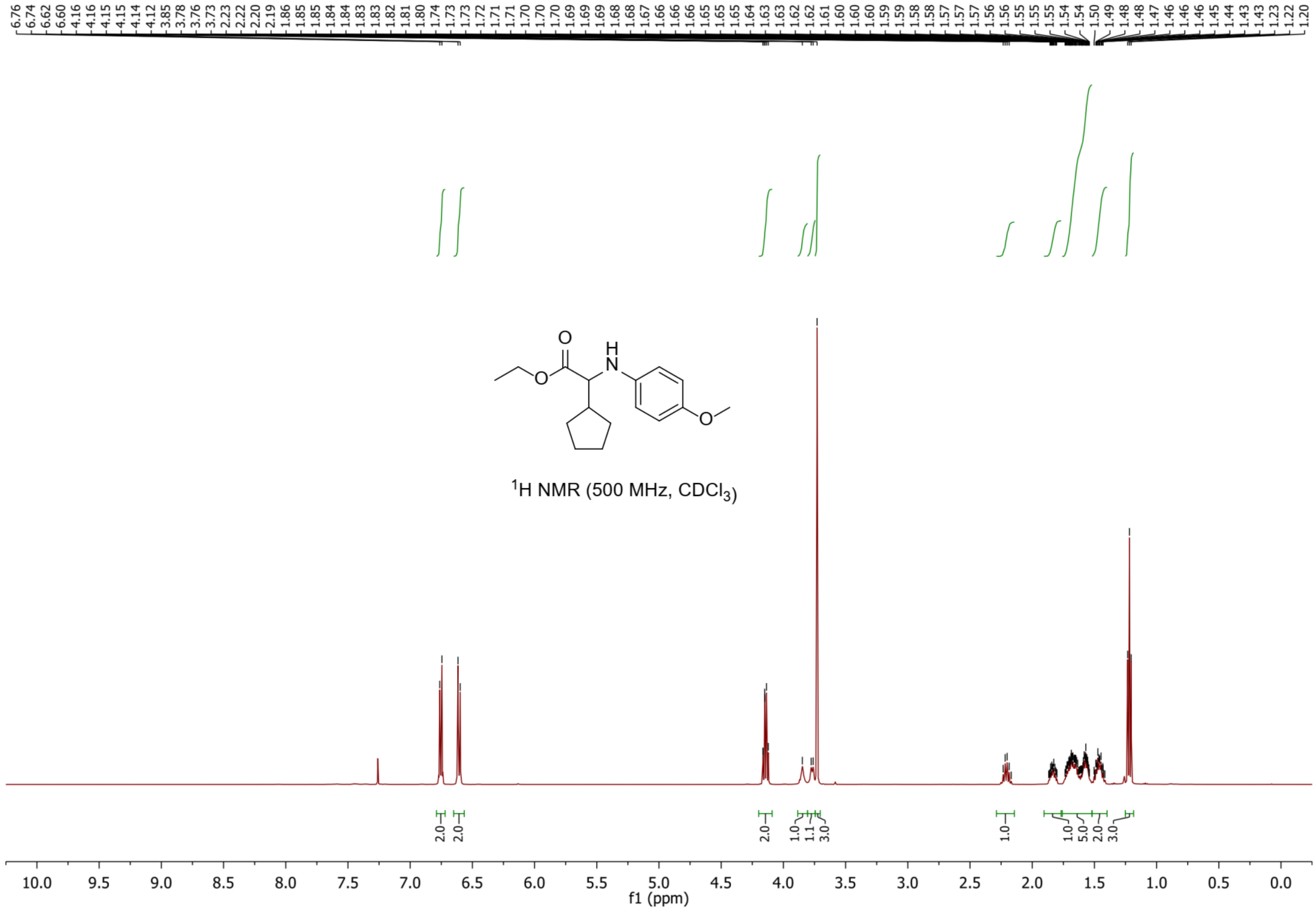

Go to table of contents

Ethyl 2-cyclopentyl-2-((4-methoxyphenyl)amino)acetate (7j)

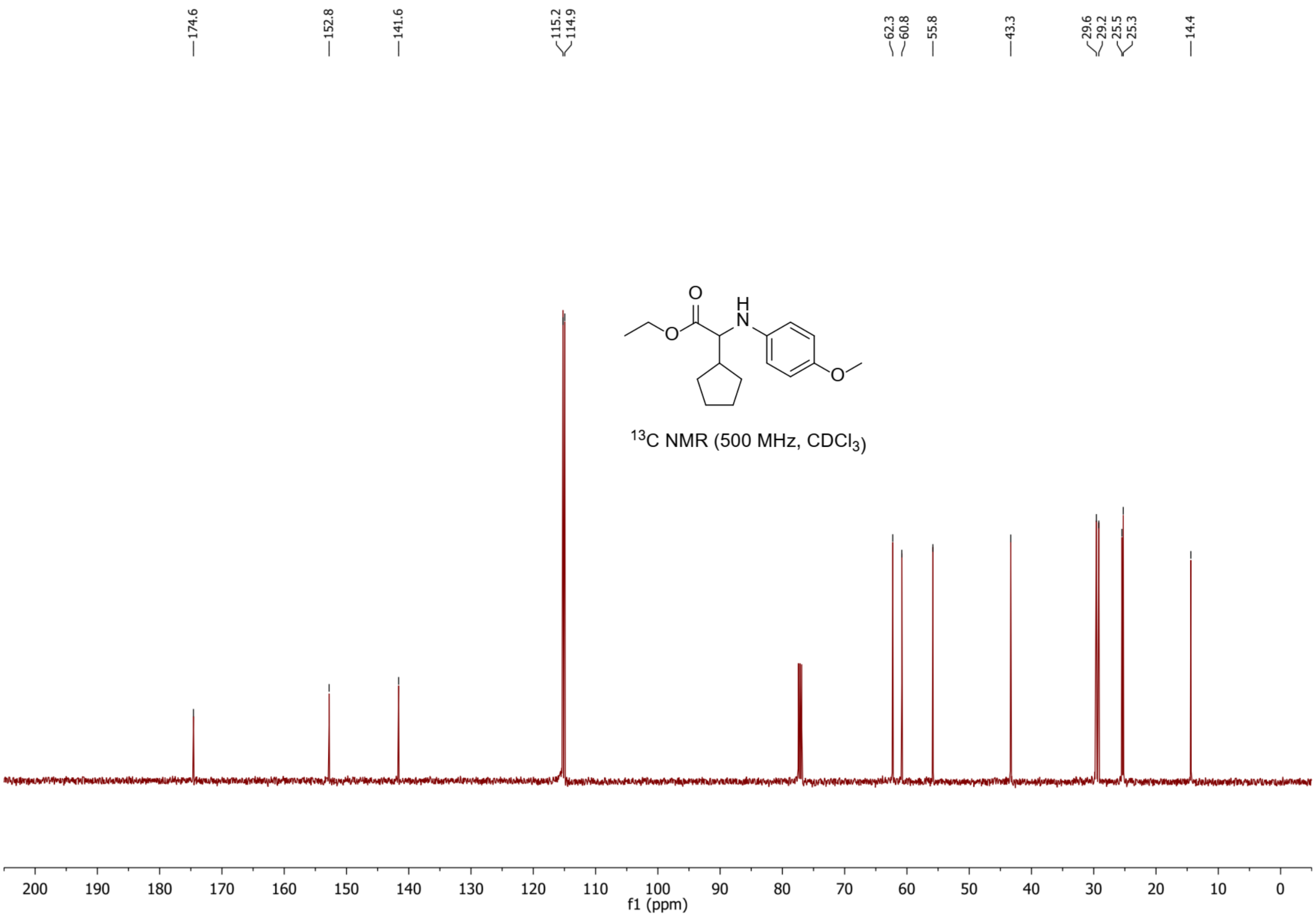

Go to table of contents

Ethyl 2-cyclohexyl-2-((4-methoxyphenyl)amino)acetate (7k)

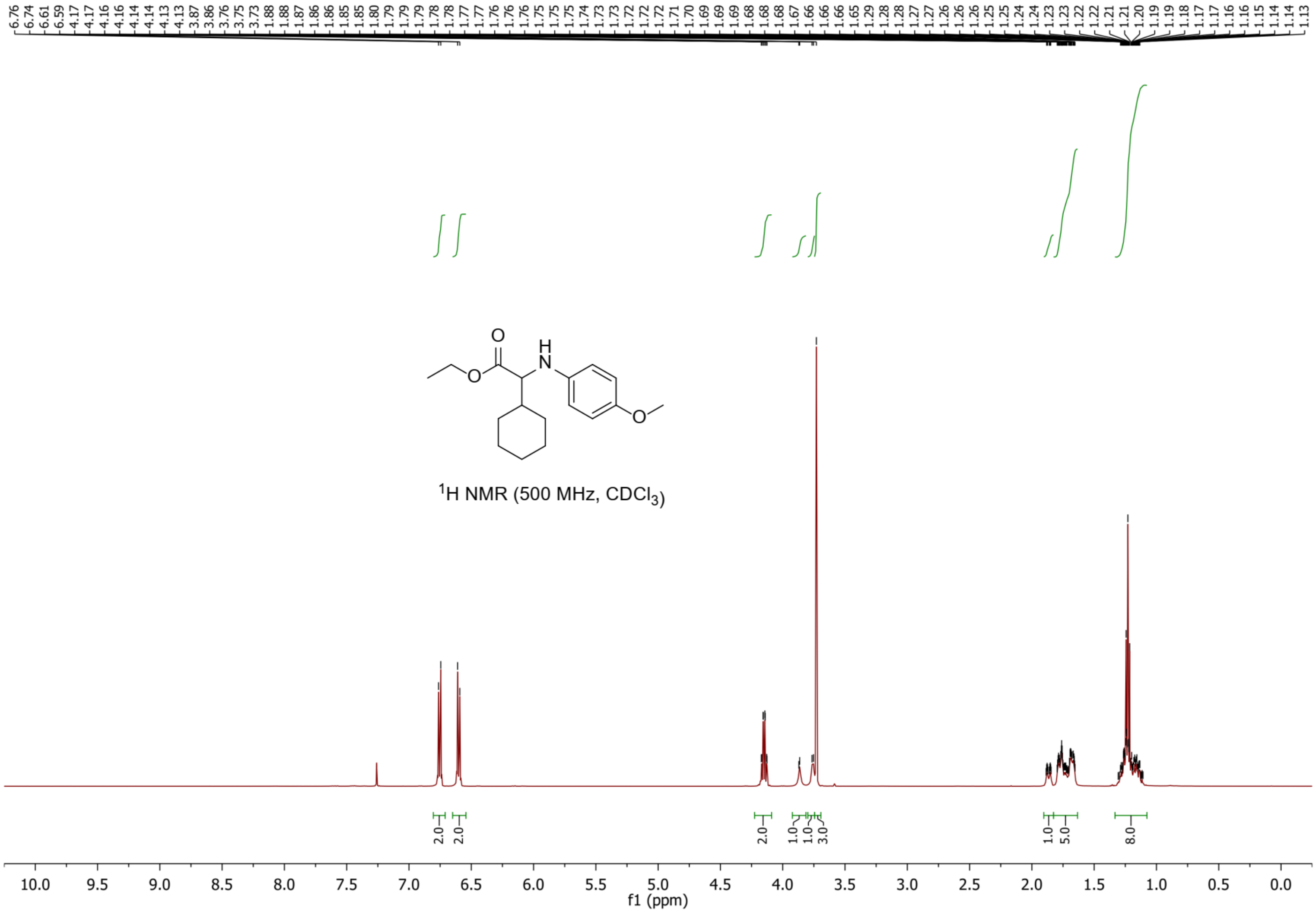

Go to table of contents

Ethyl 2-cyclohexyl-2-((4-methoxyphenyl)amino)acetate (7k)

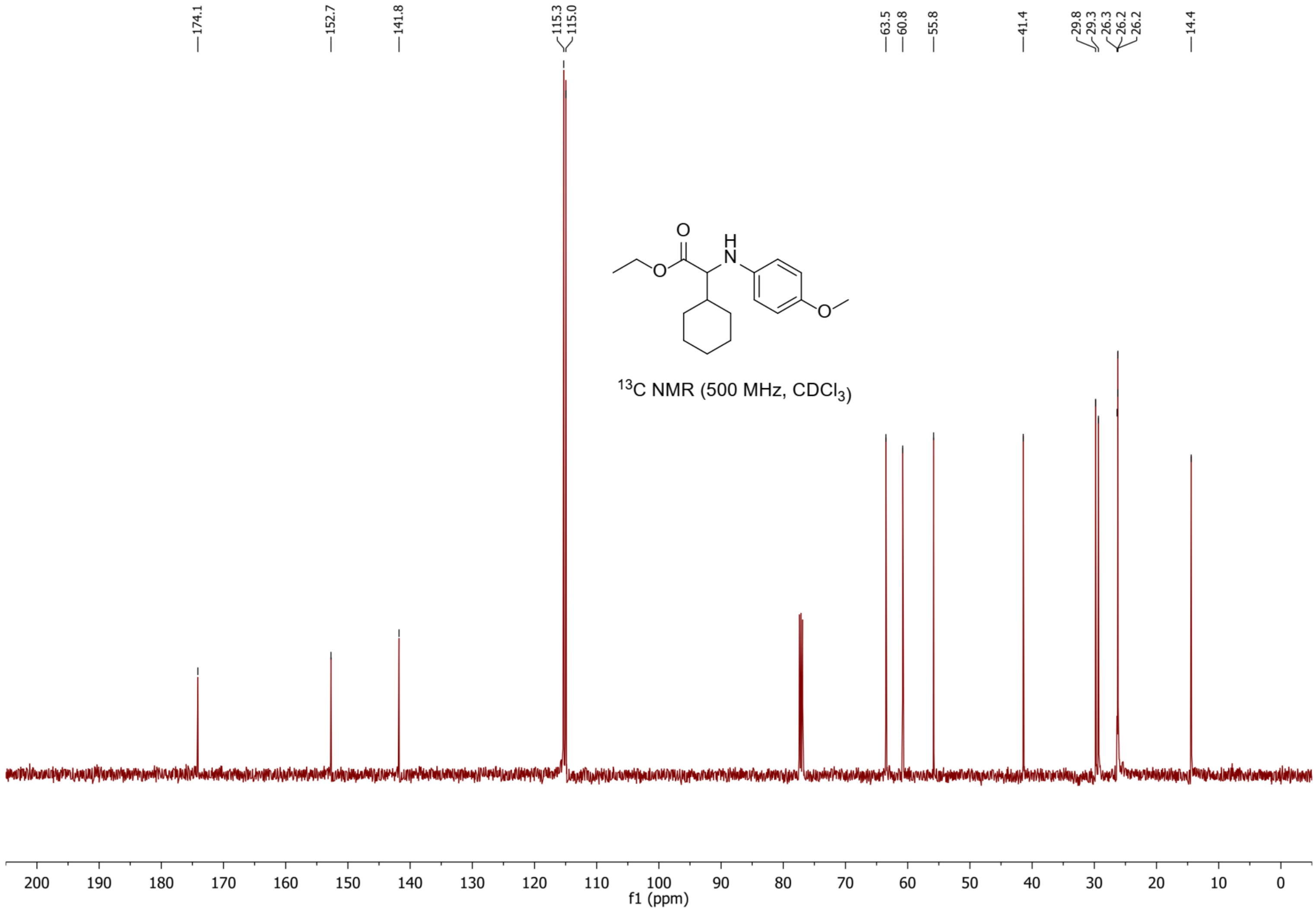

Go to table of contents

Ethyl 2-cycloheptyl-2-((4-methoxyphenyl)amino)acetate (7l)

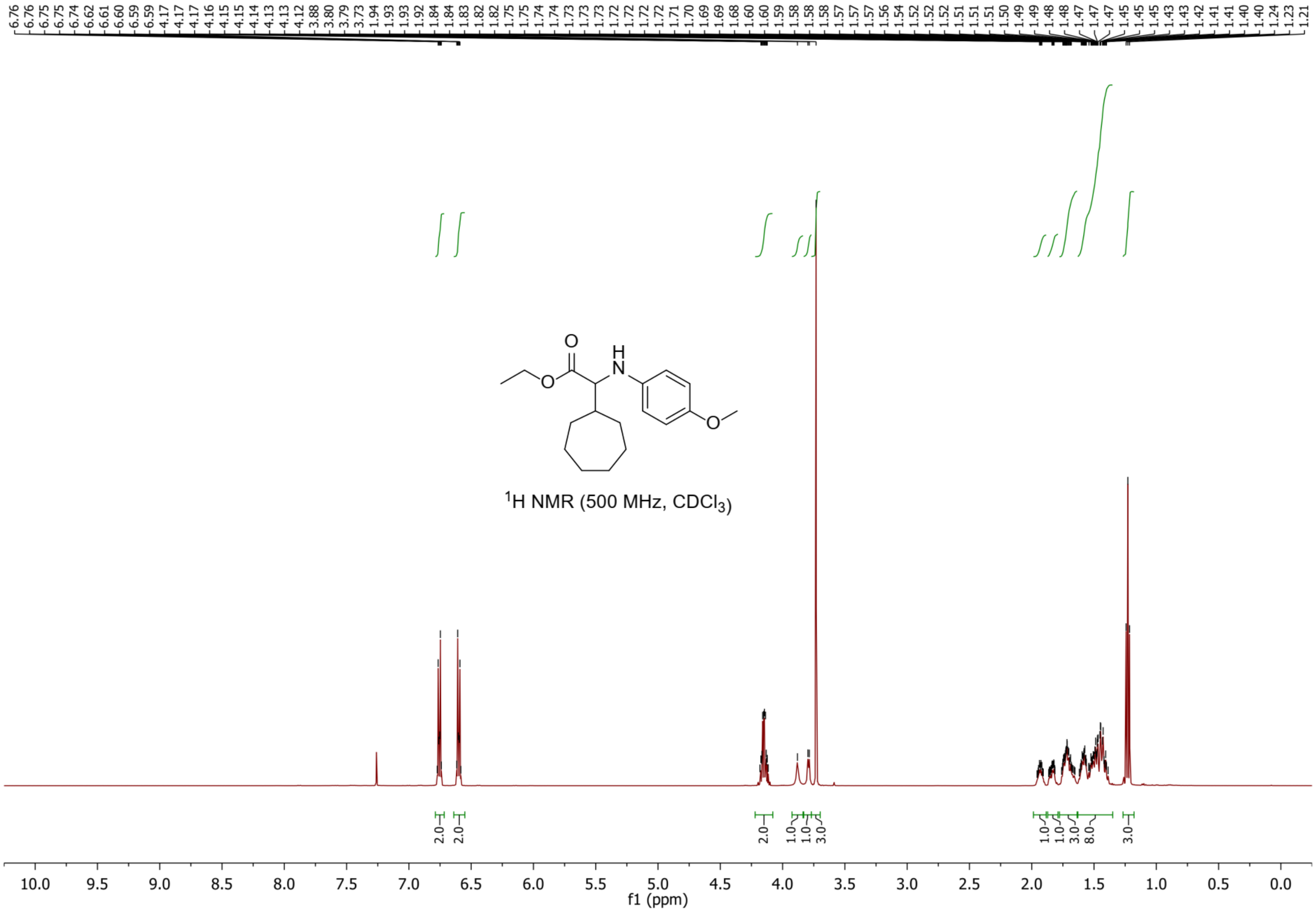

Go to table of contents

Ethyl 2-cycloheptyl-2-((4-methoxyphenyl)amino)acetate (7l)

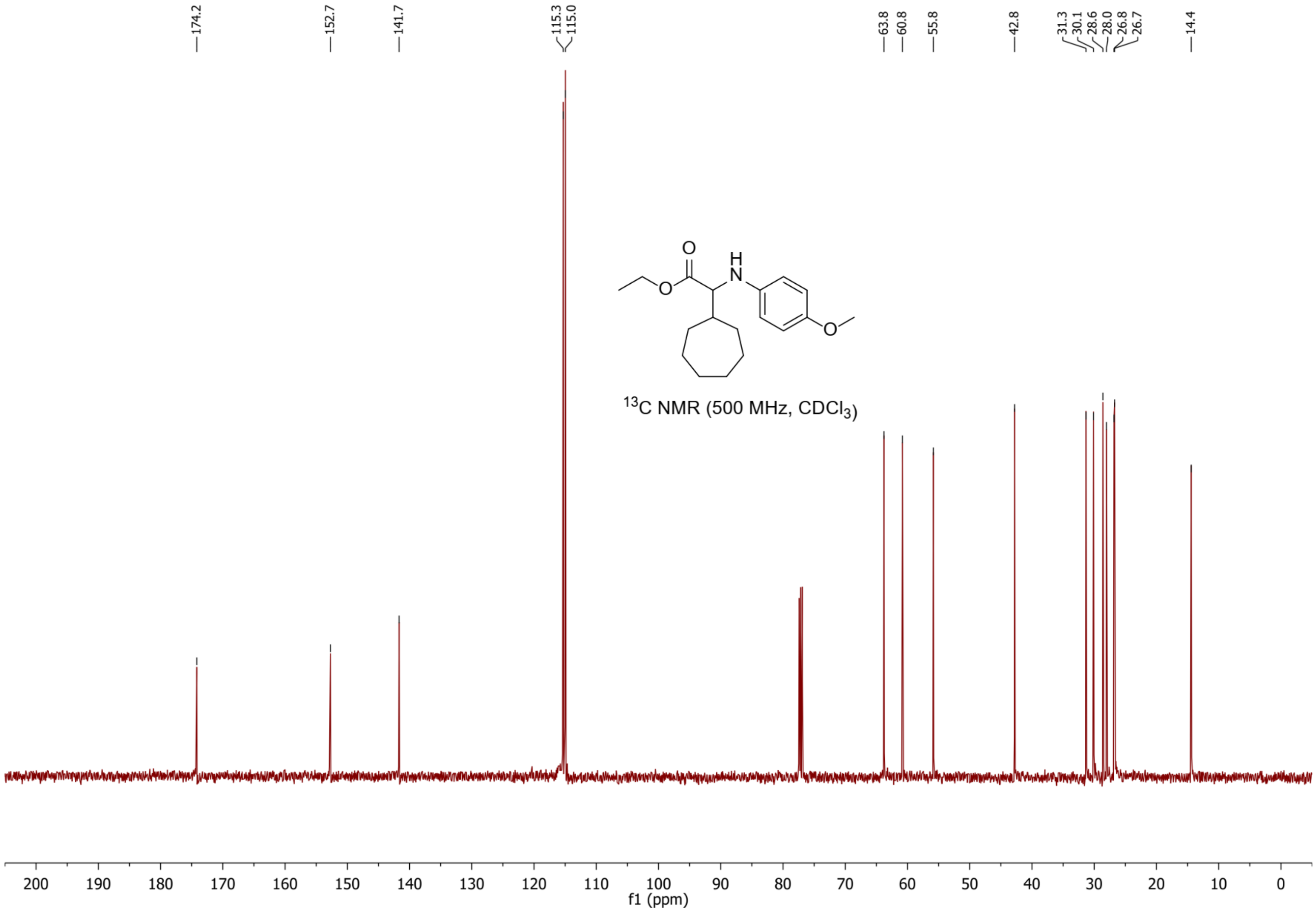

Go to table of contents

Ethyl 2-cyclobutyl-2-((4-methoxyphenyl)amino)acetate (7m)

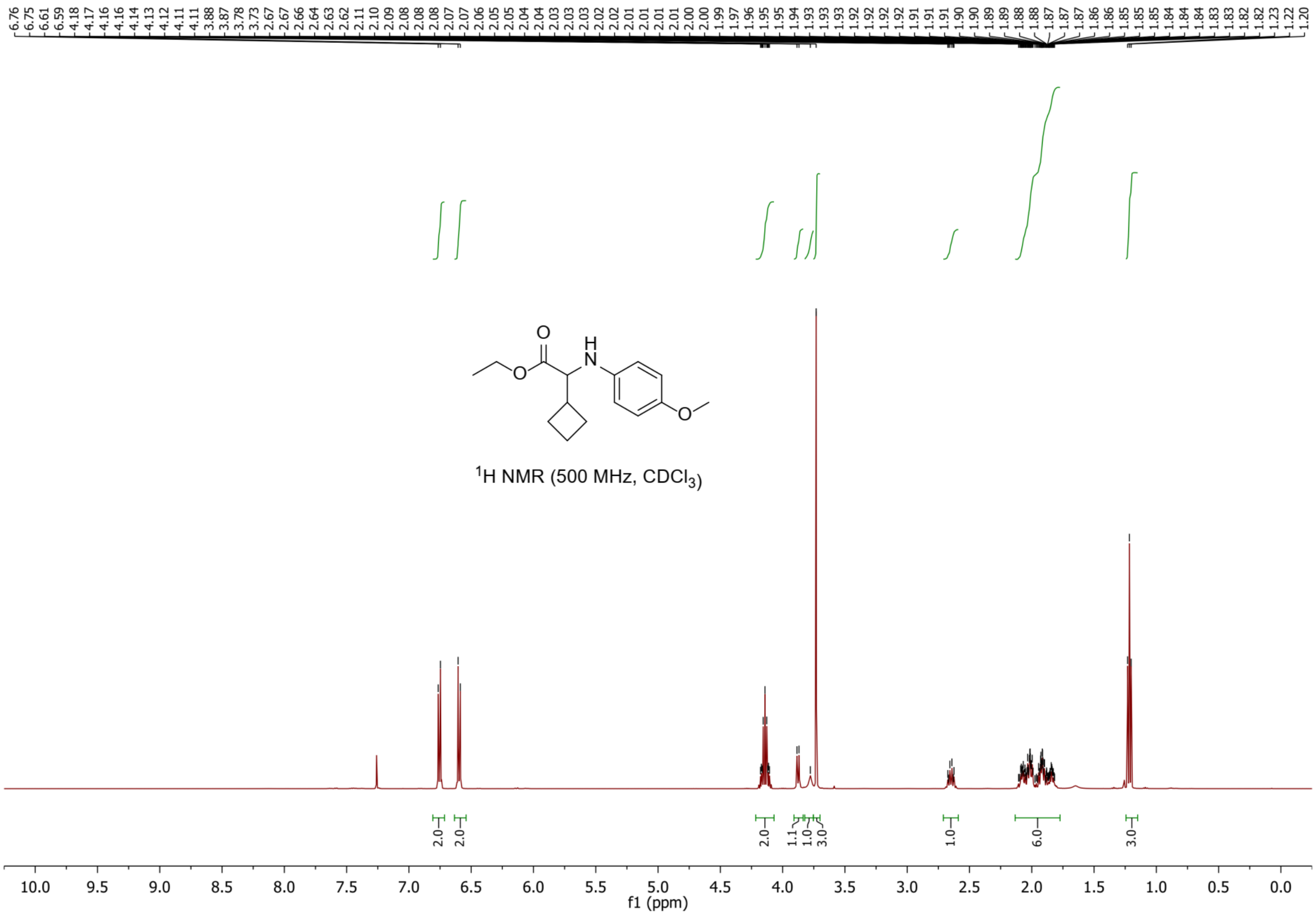

Ethyl 2-cyclobutyl-2-((4-methoxyphenyl)amino)acetate (7m)

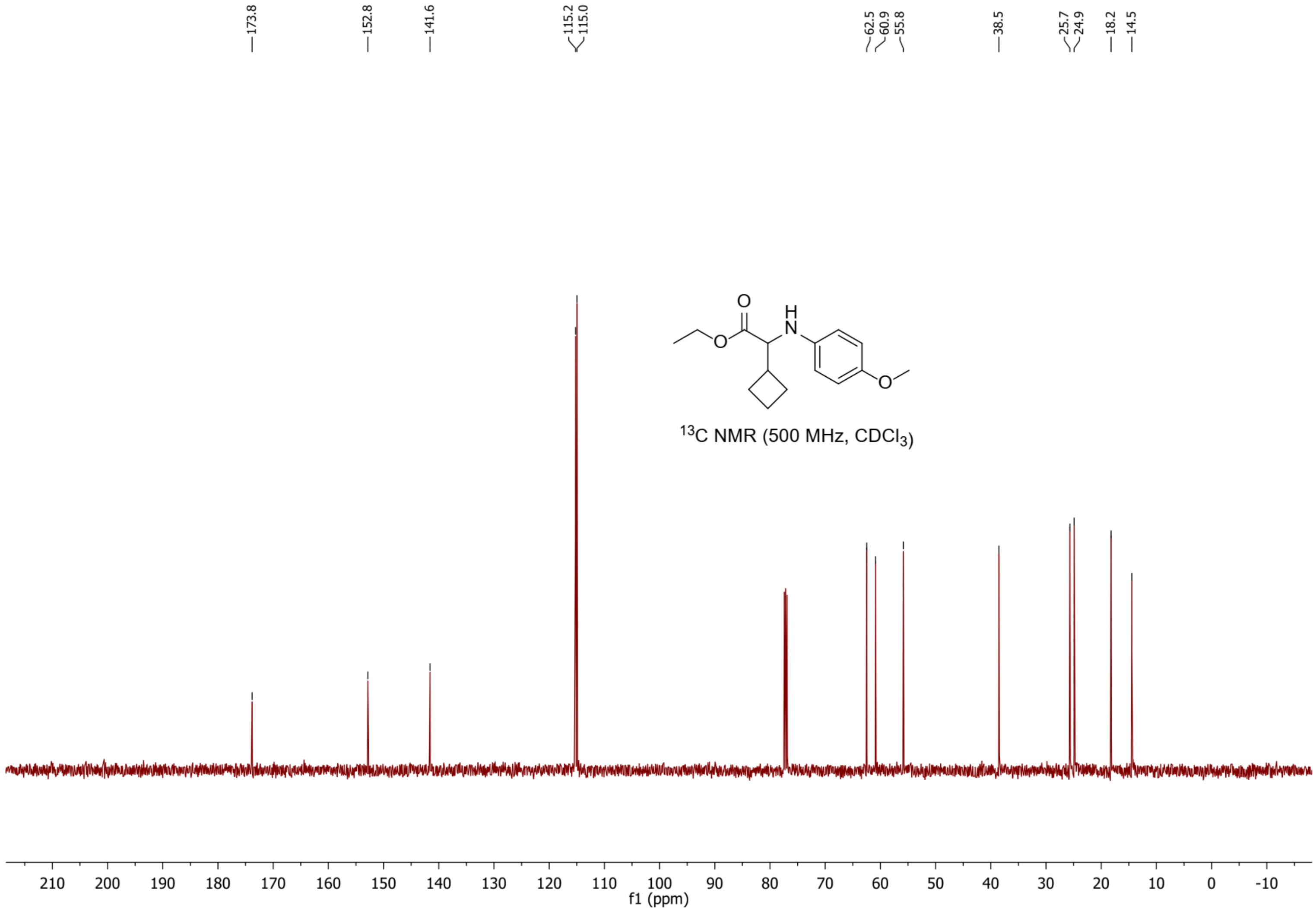

Go to table of contents

Ethyl 2-((4-methoxyphenyl)amino)-2-(tetrahydro-2*H*-pyran-4-yl)acetate (7n)

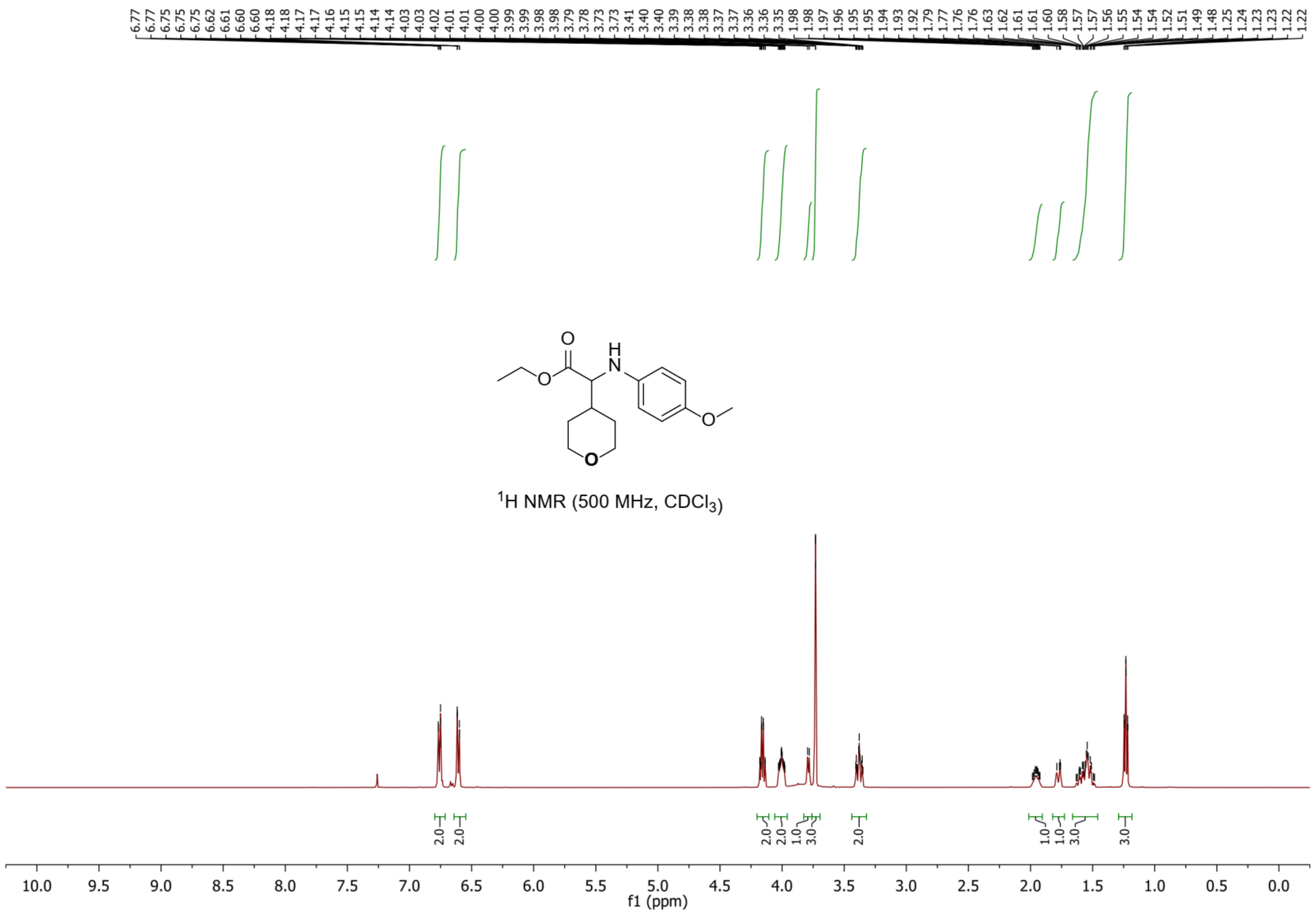

Go to table of contents

Ethyl 2-((4-methoxyphenyl)amino)-2-(tetrahydro-2*H*-pyran-4-yl)acetate (7n)

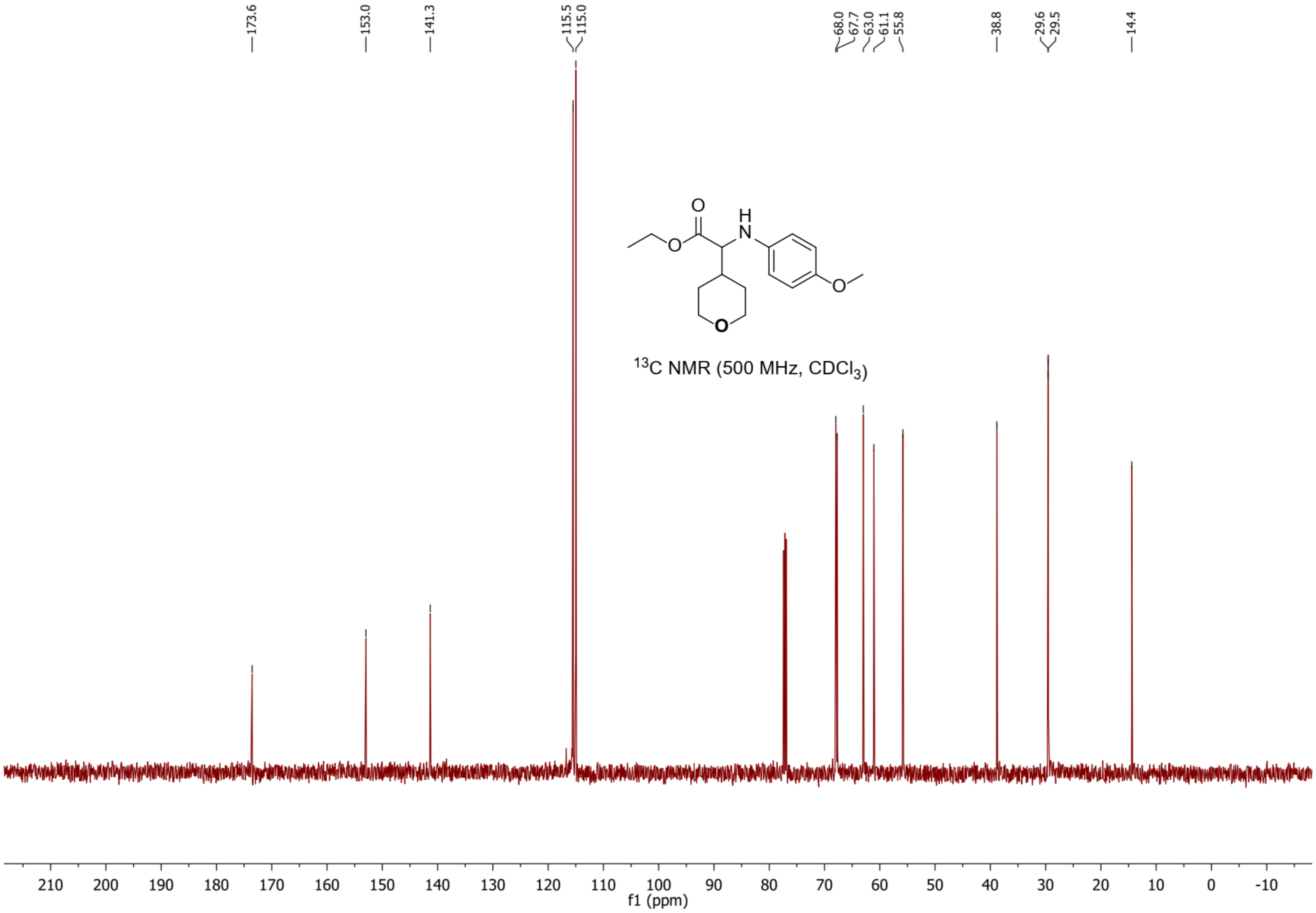

Go to table of contents

Ethyl (4-methoxyphenyl)valinate (7o)

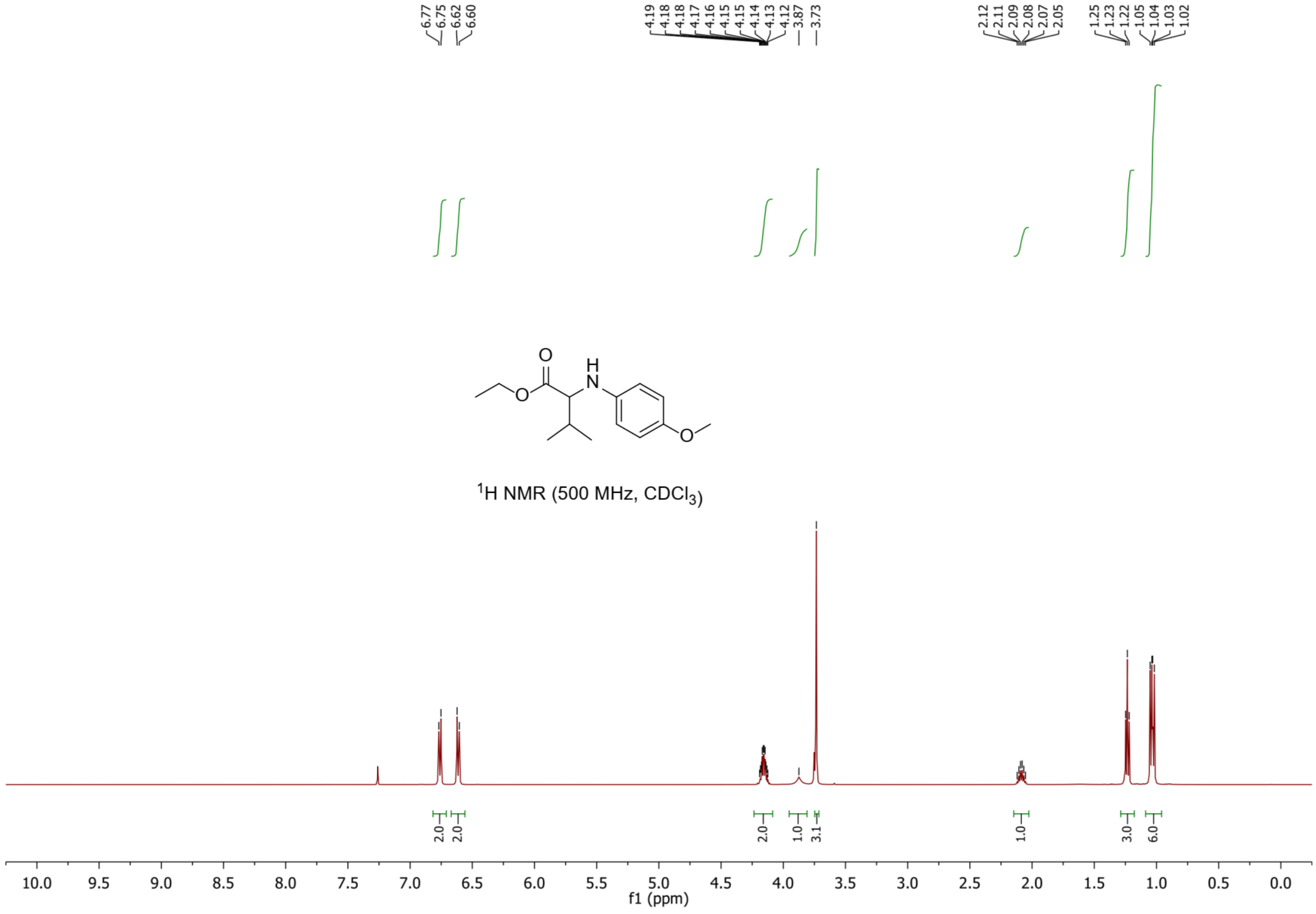

Go to table of contents

Ethyl (4-methoxyphenyl)valinate (7o)

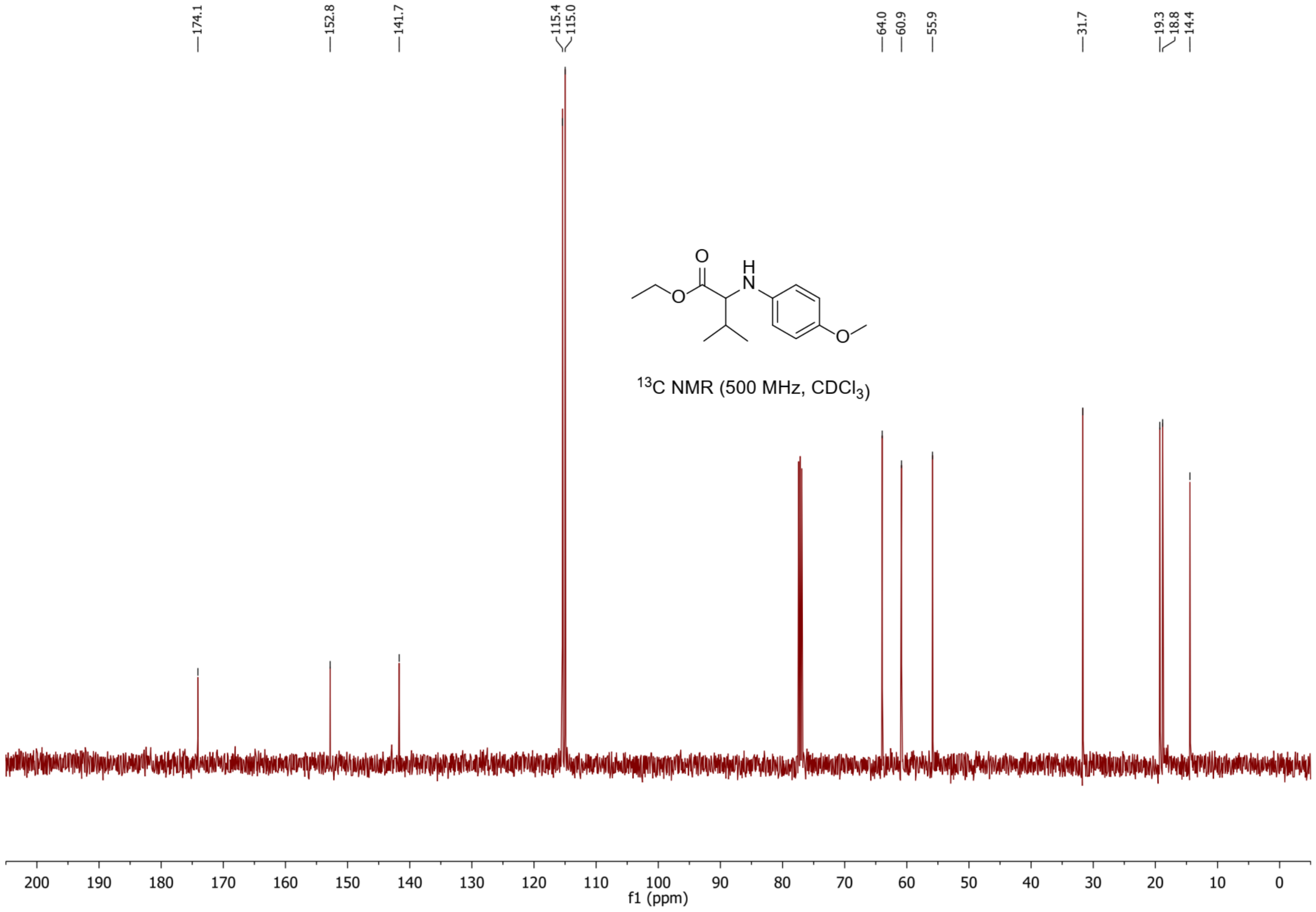

Go to table of contents

Ethyl 2-((4-methoxyphenyl)amino)-3-propylhexanoate (7p)

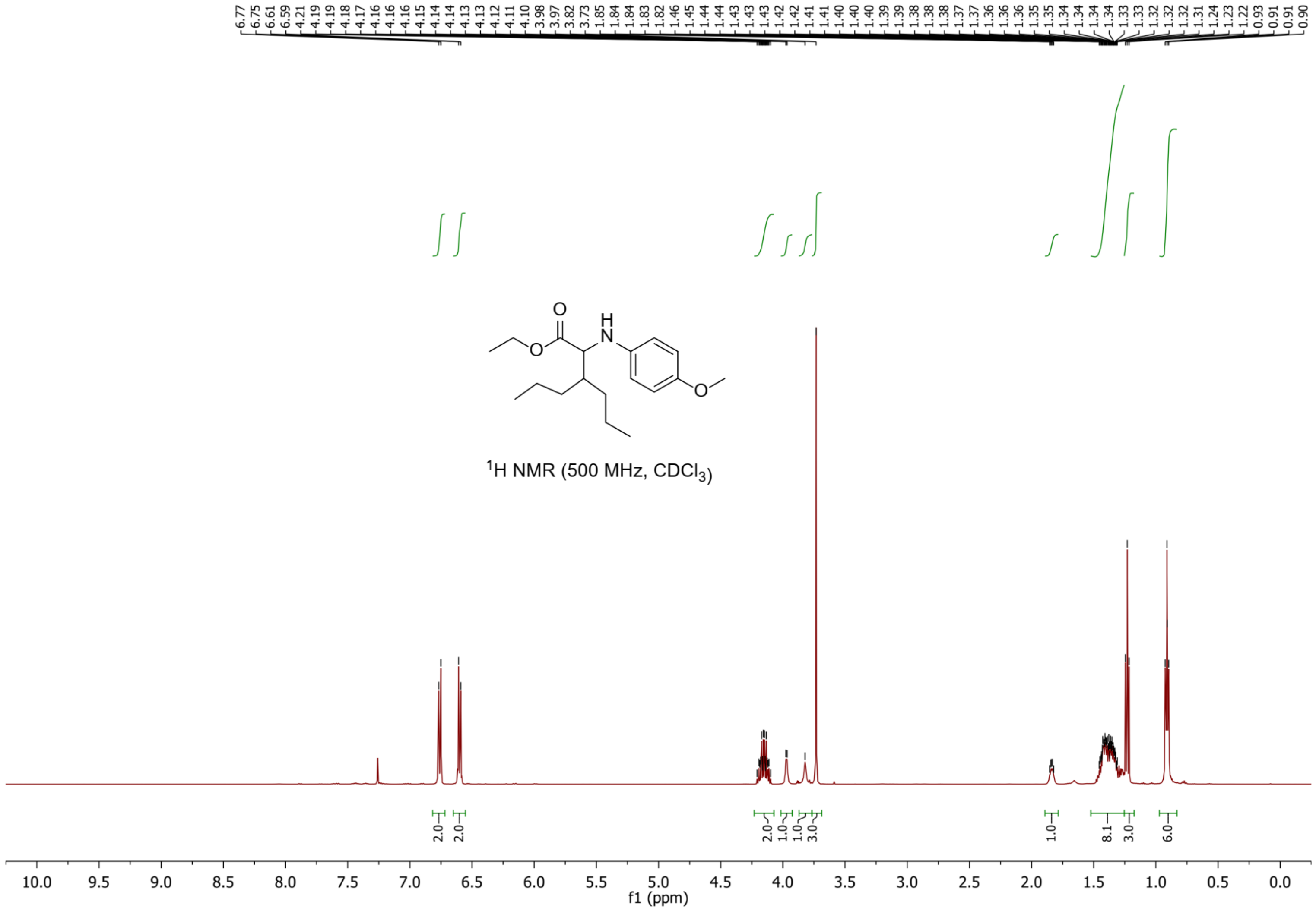

Go to table of contents

Ethyl 2-((4-methoxyphenyl)amino)-3-propylhexanoate (7p)

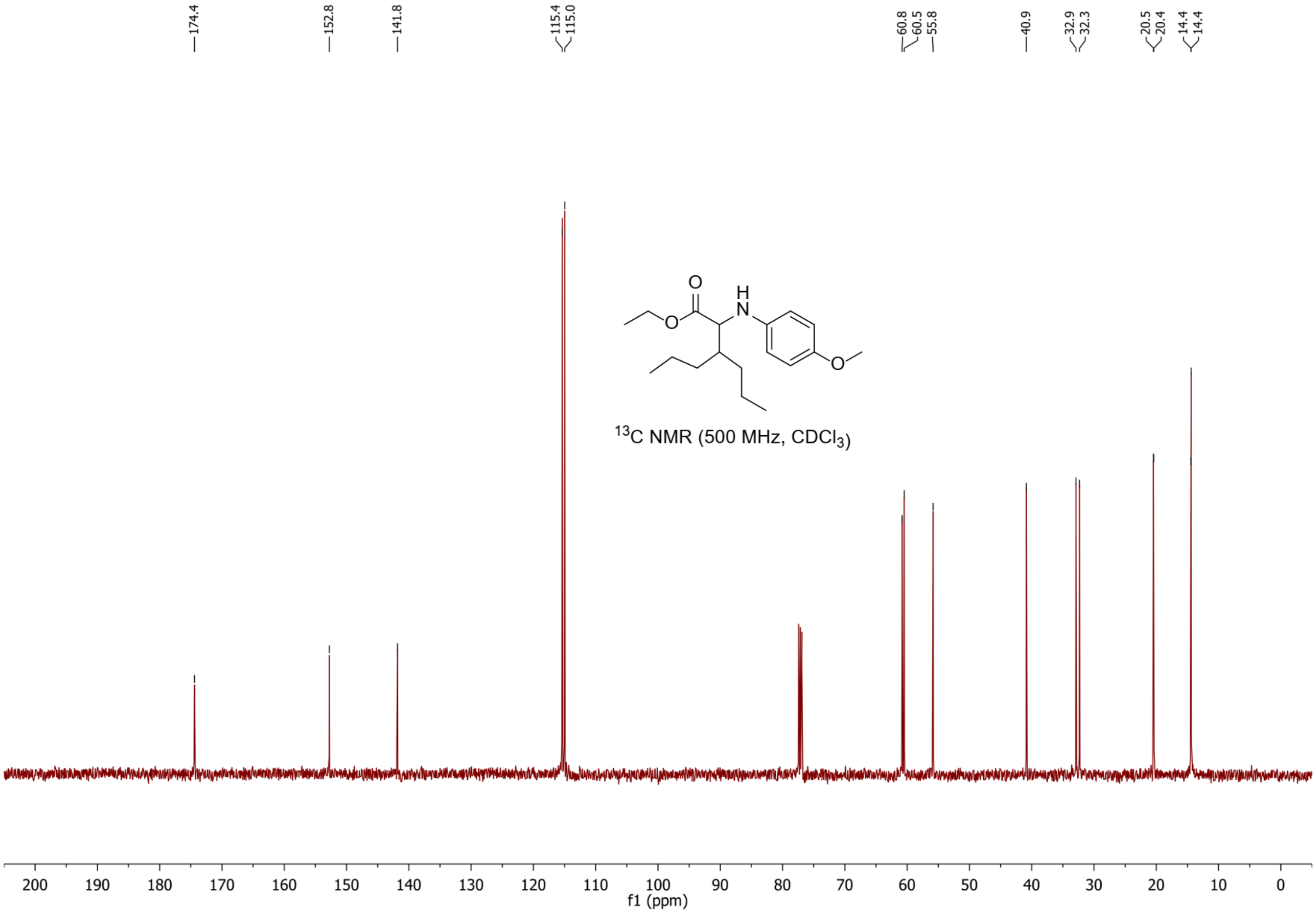

Go to table of contents

Ethyl 3-hexyl-2-((4-methoxyphenyl)amino)decanoate (7q)

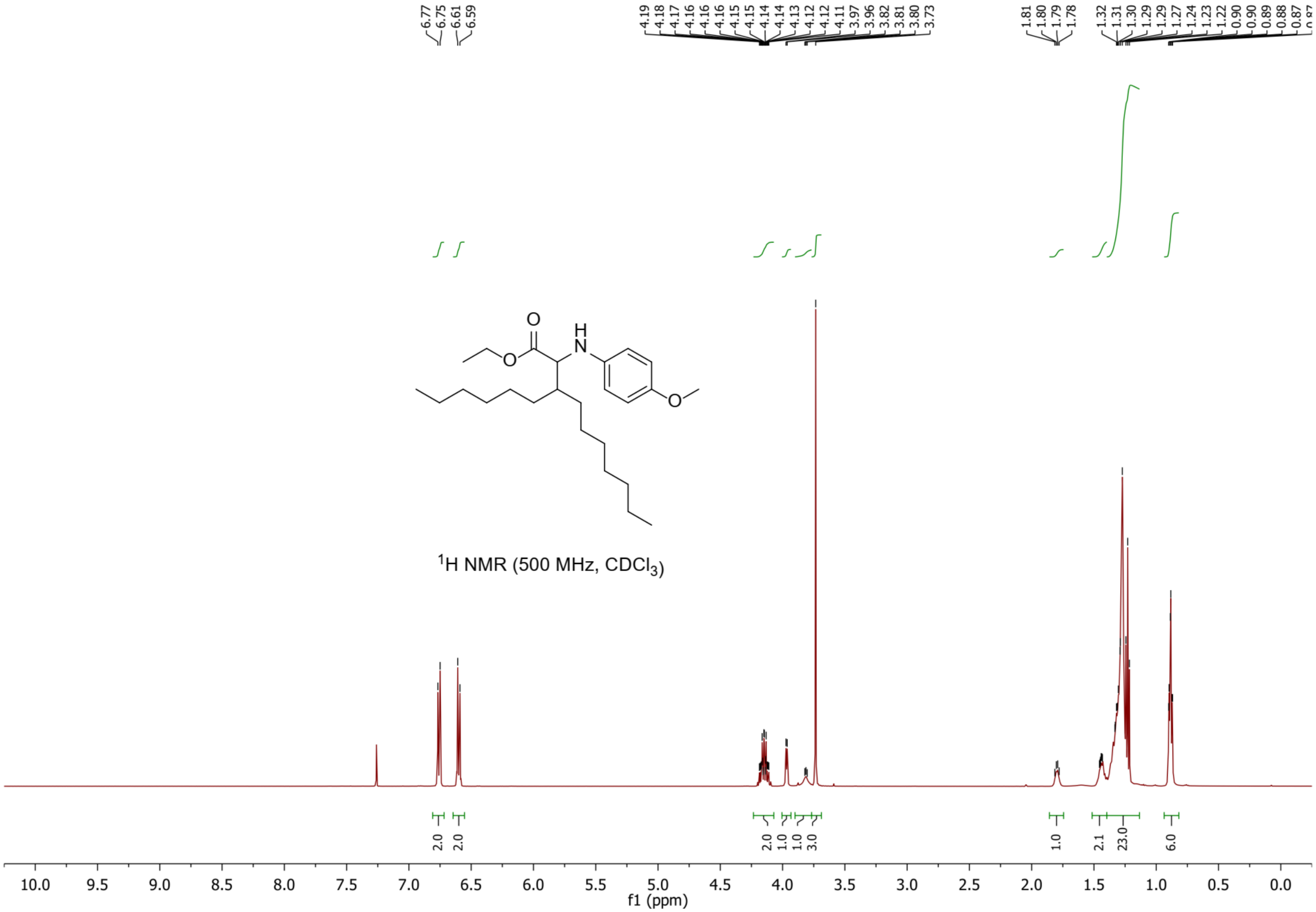

Go to table of contents

Ethyl 3-hexyl-2-((4-methoxyphenyl)amino)decanoate (7q)

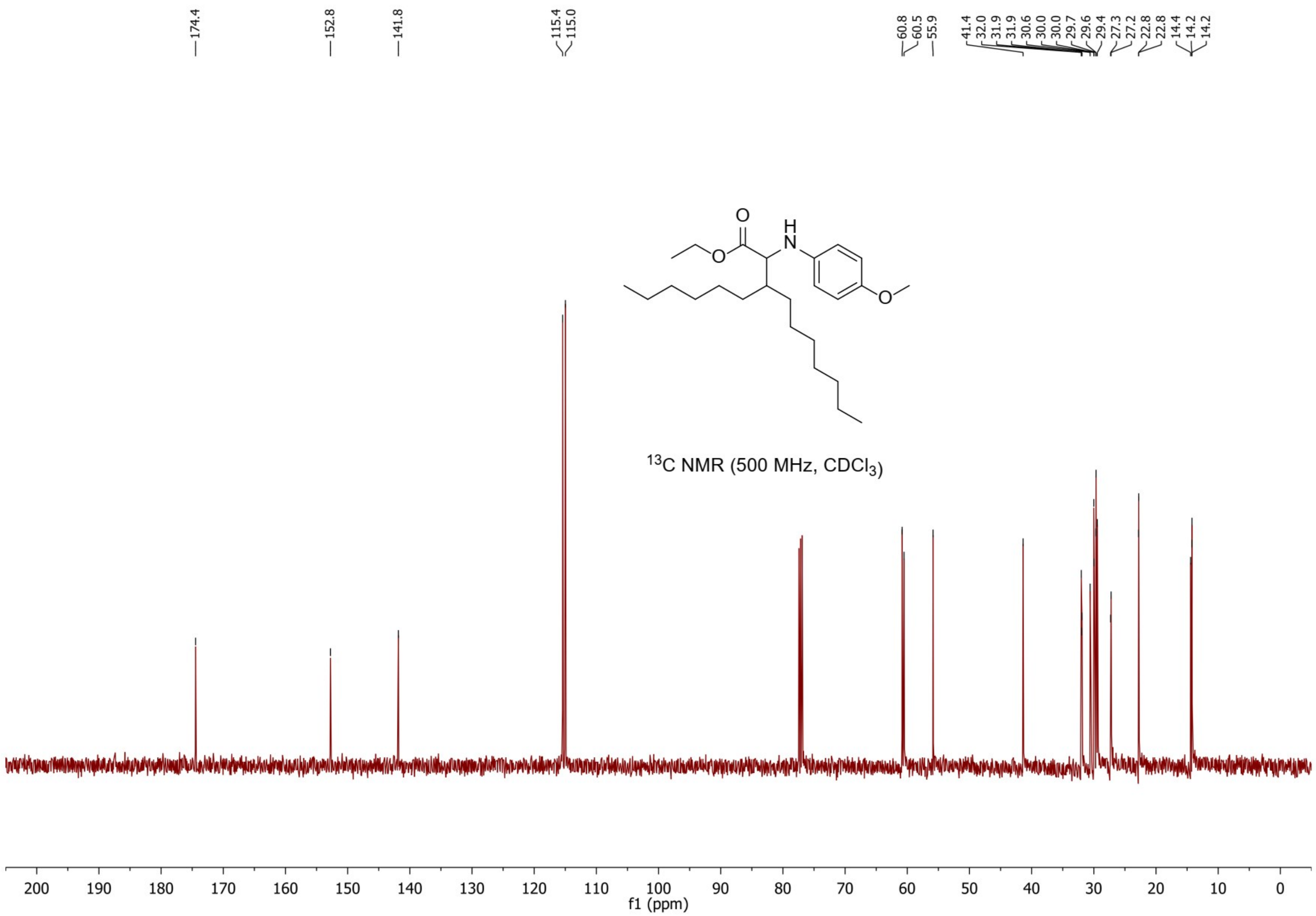

Go to table of contents

Ethyl 2-(2,3-dihydro-1*H*-inden-2-yl)-2-((4-methoxyphenyl)amino)acetate (7r)

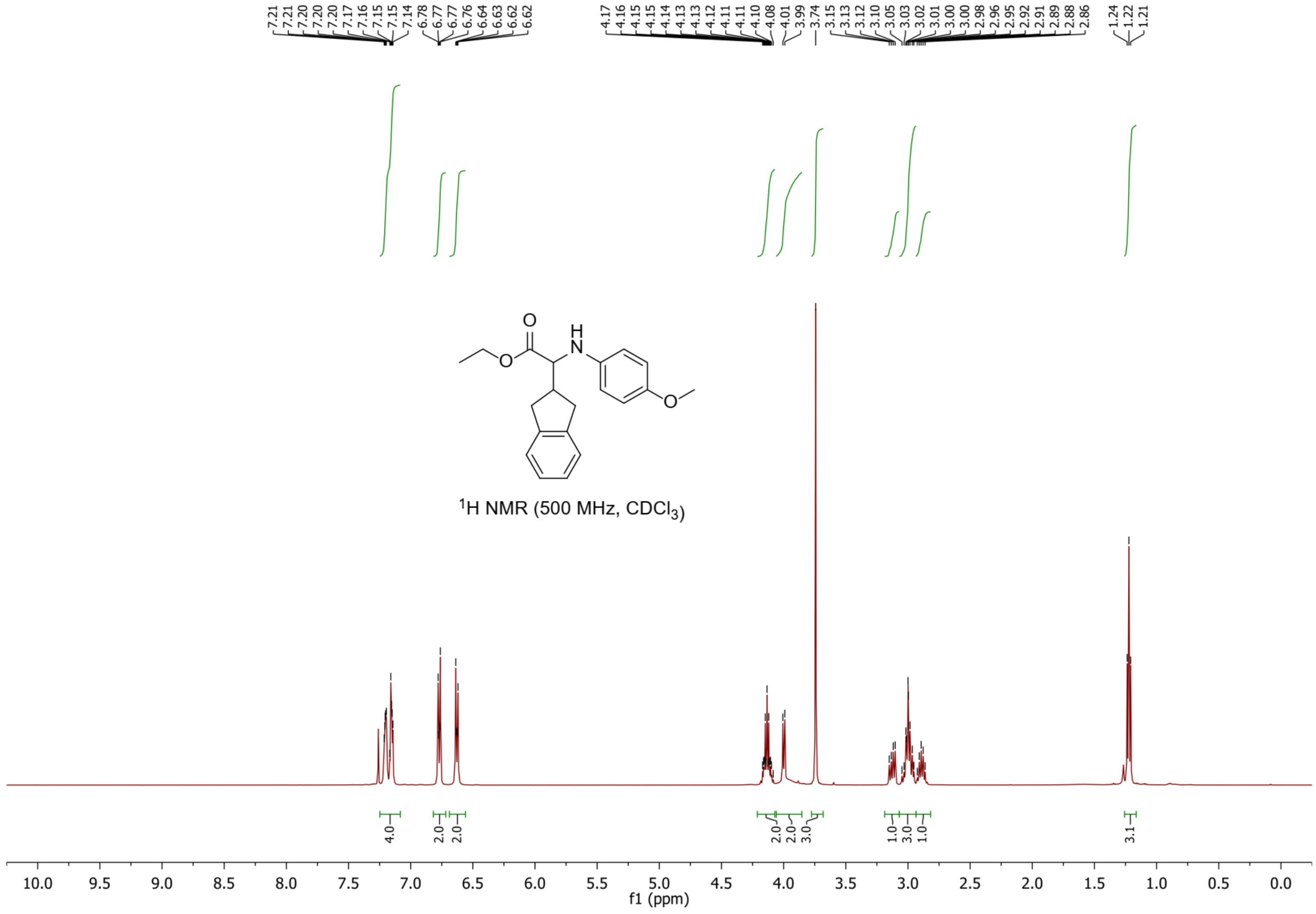

Go to table of contents

Ethyl 2-(2,3-dihydro-1*H*-inden-2-yl)-2-((4-methoxyphenyl)amino)acetate (7r)

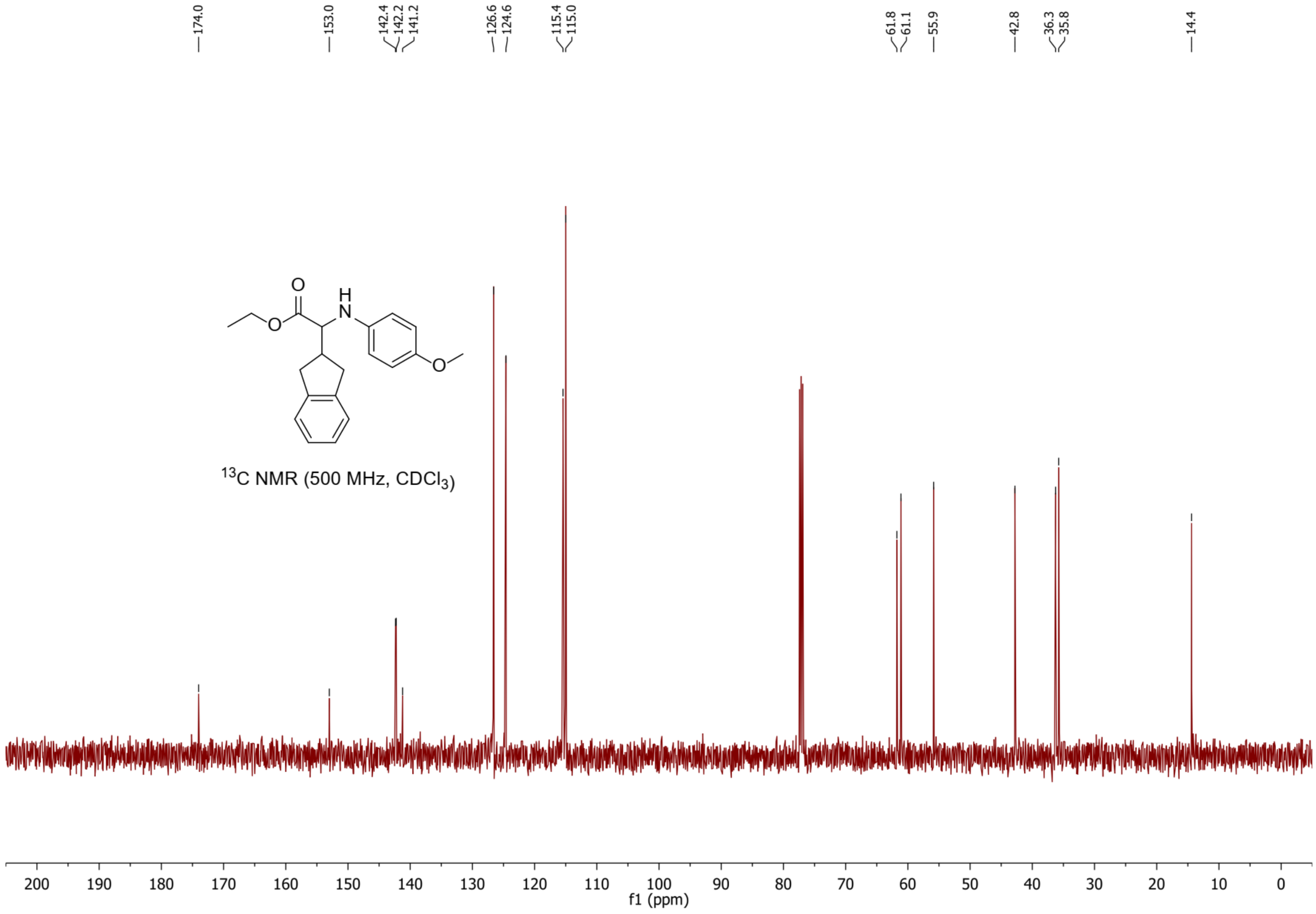

Go to table of contents

Ethyl 2-((4-methoxyphenyl)amino)-3,3-dimethylbutanoate (7s)

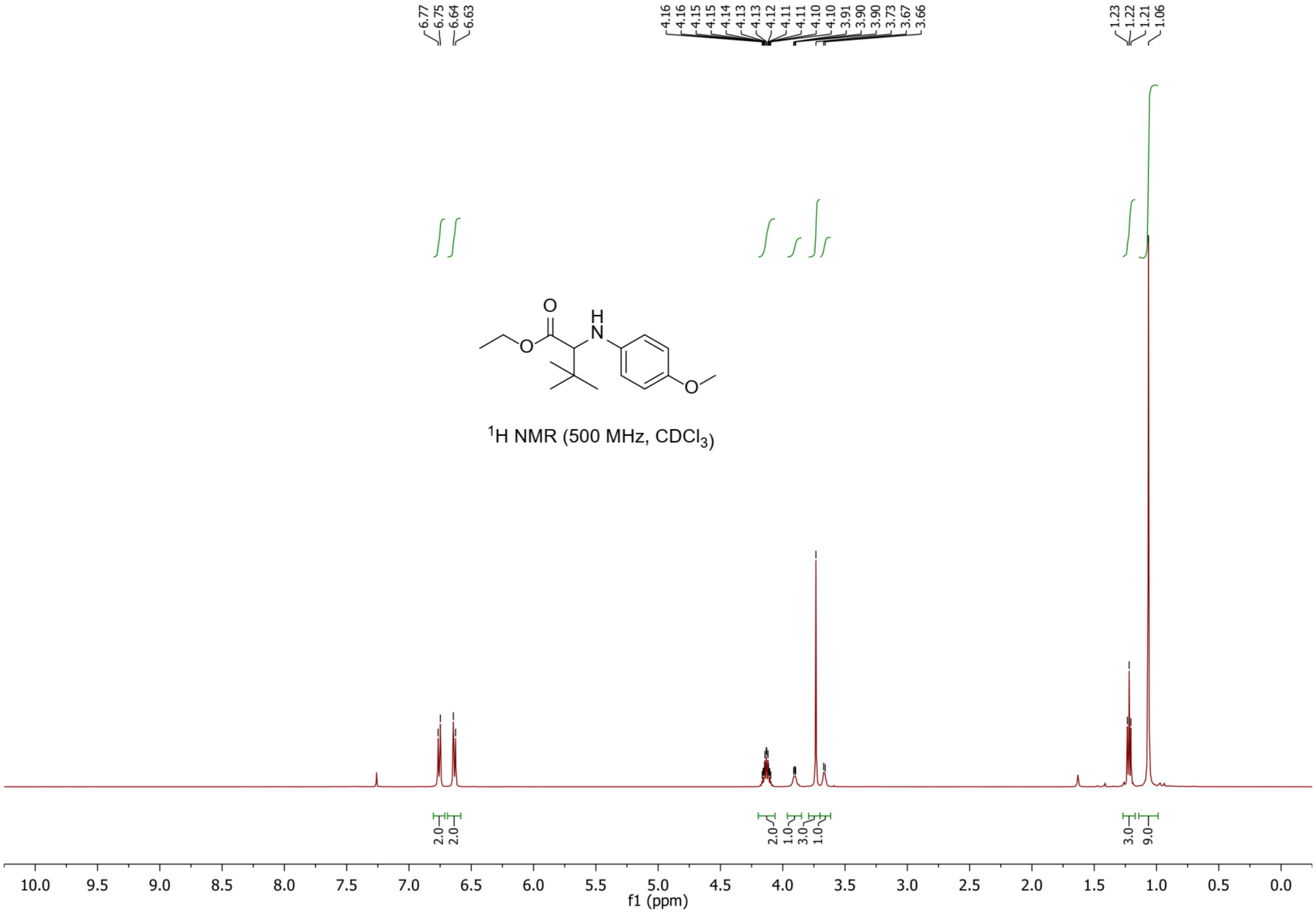

Go to table of contents

Ethyl 2-((4-methoxyphenyl)amino)-3,3-dimethylbutanoate (7s)

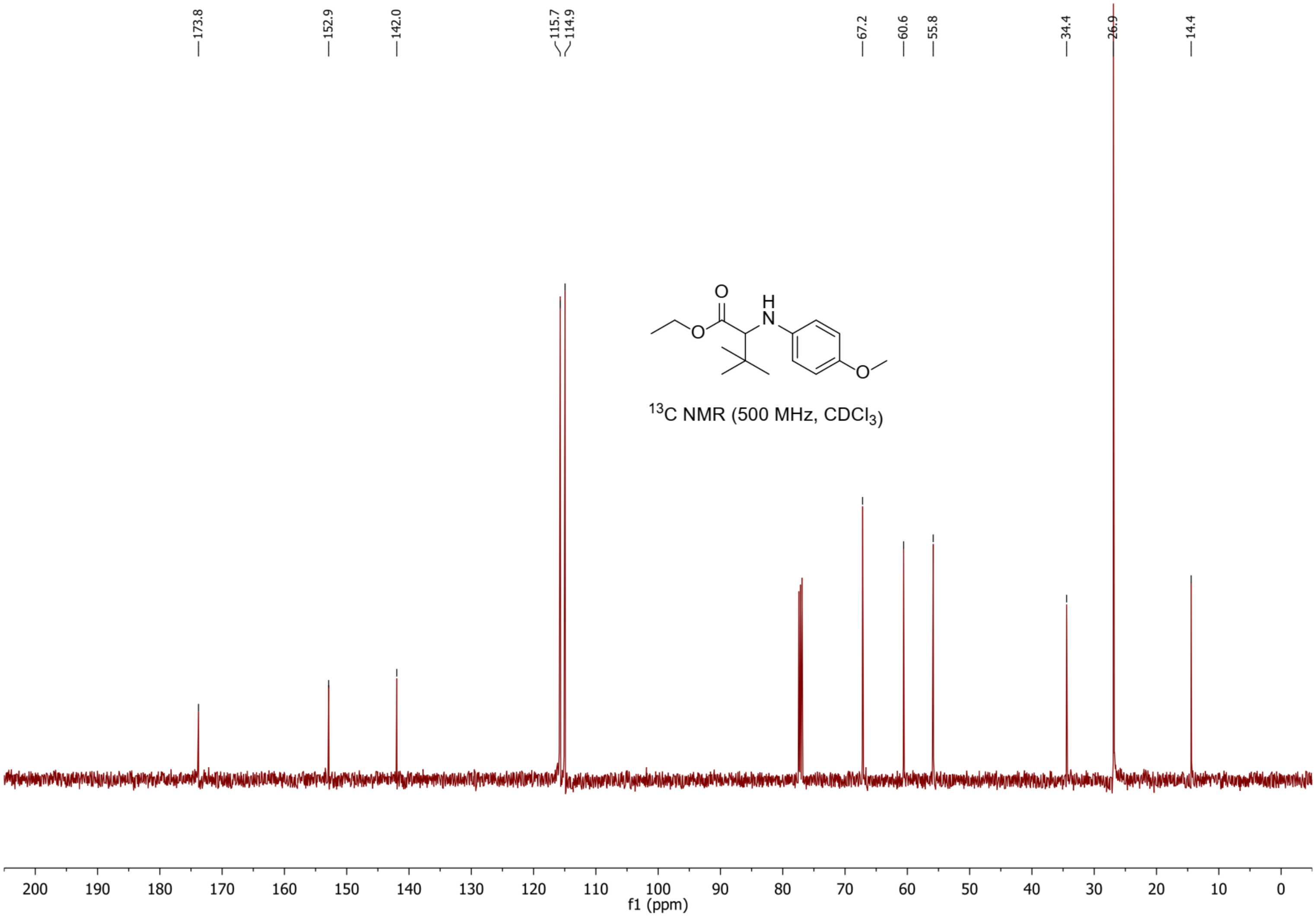

Ethyl 2-((4-methoxyphenyl)amino)-3,3-dimethylpentanoate (7t)

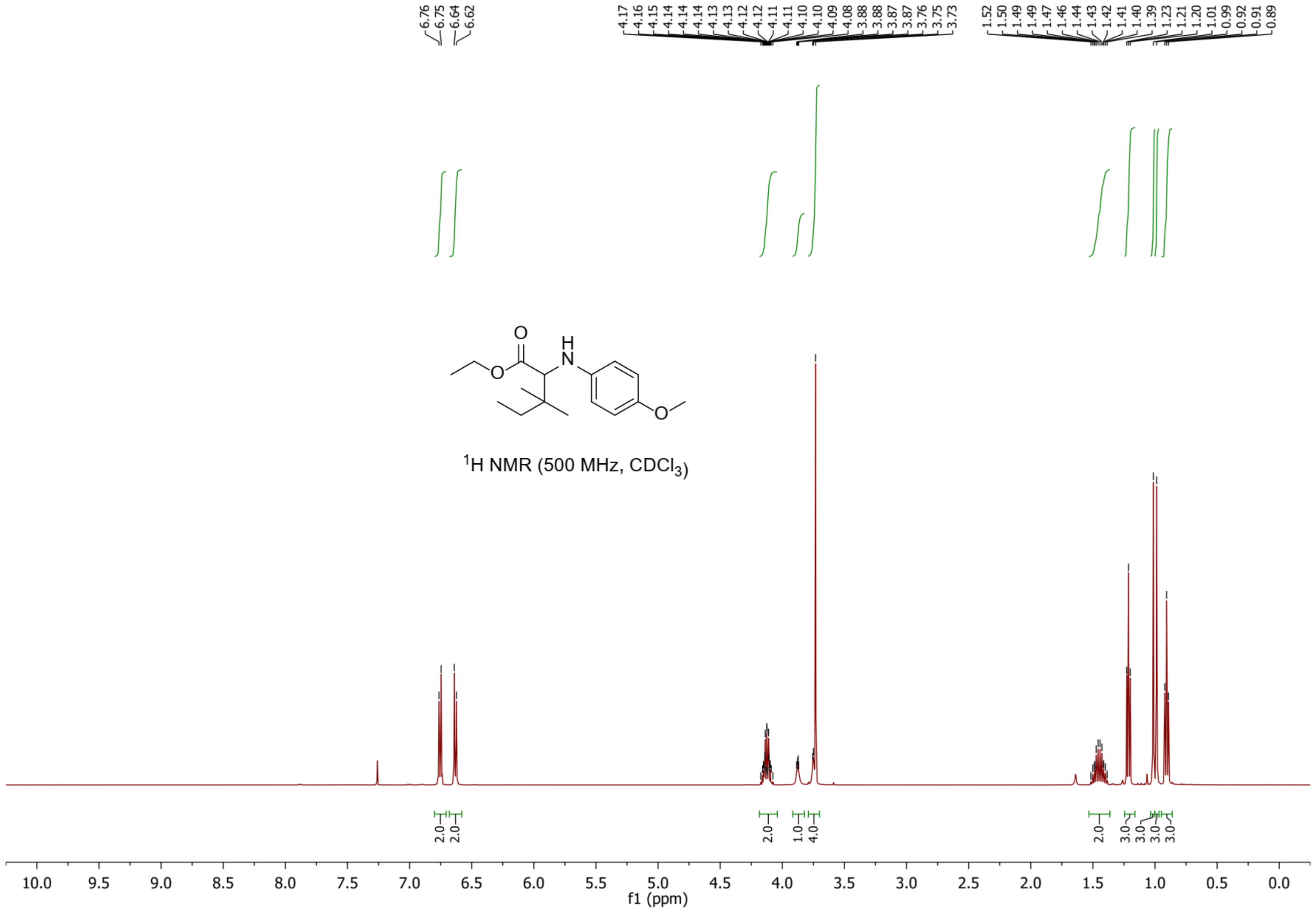

Go to table of contents

Ethyl 2-((4-methoxyphenyl)amino)-3,3-dimethylpentanoate (7t)

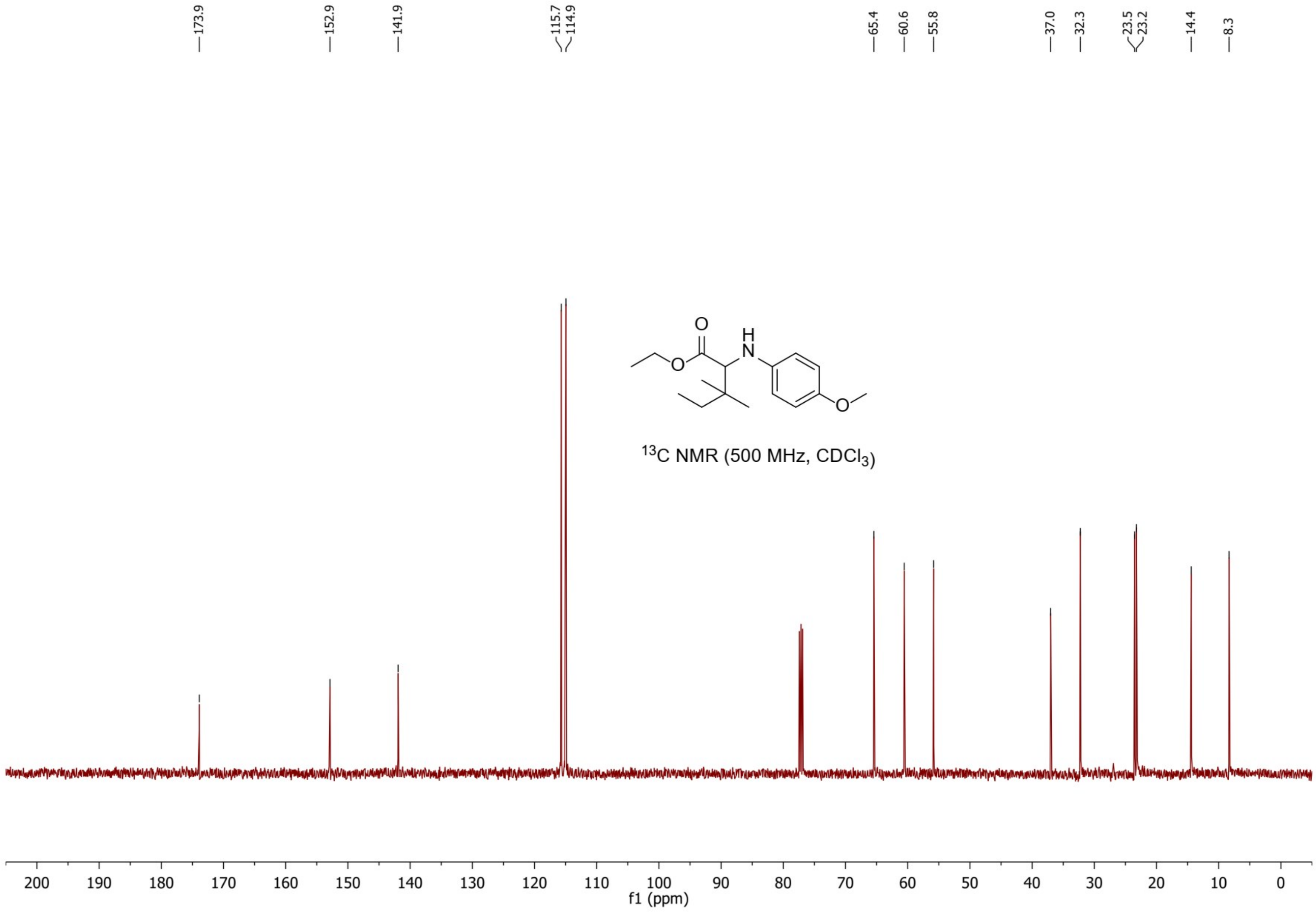

Go to table of contents

Ethyl 2-((4-methoxyphenyl)amino)-2-(1-methylcyclohexyl)acetate (7u)

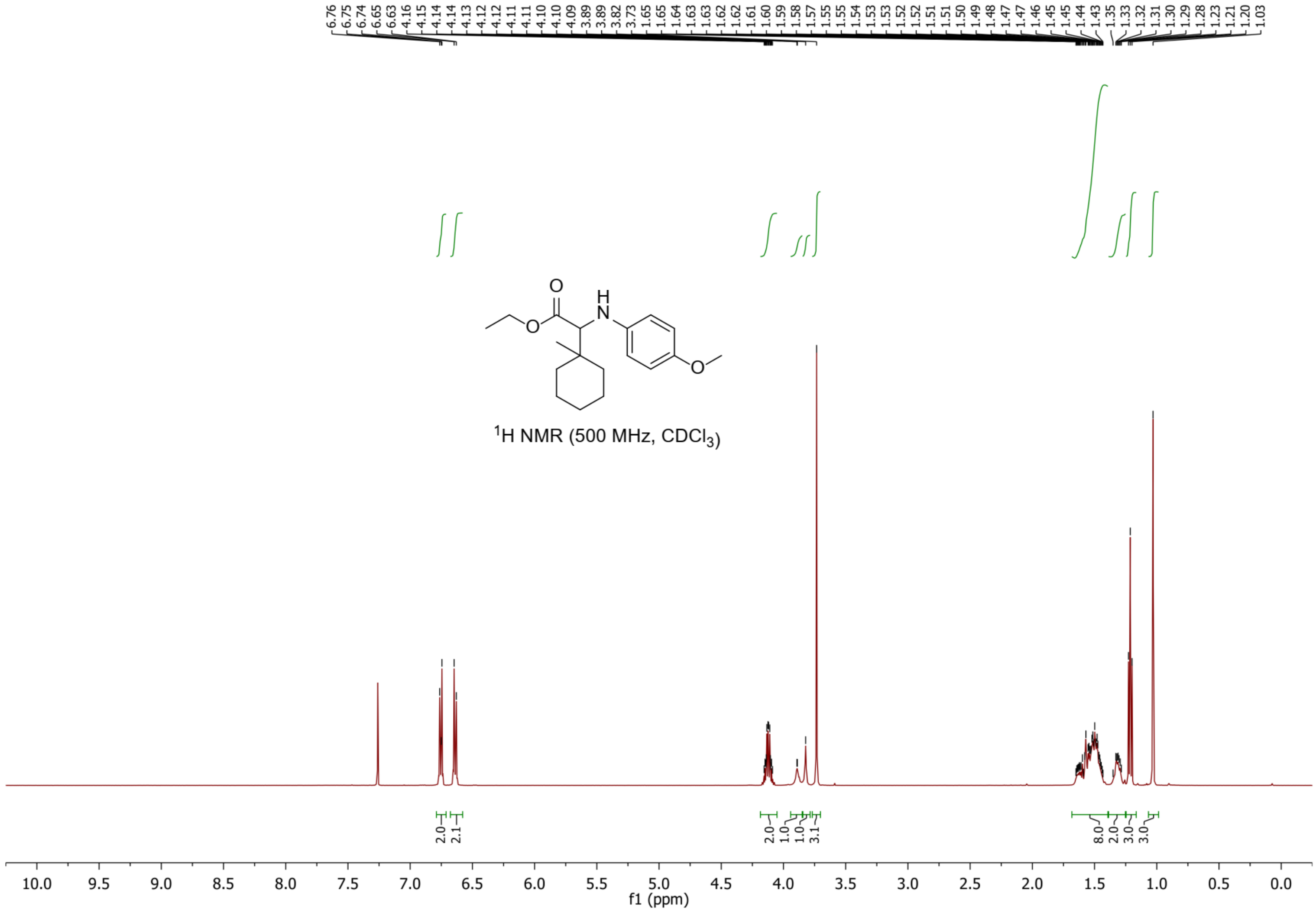

Go to table of contents

Ethyl 2-((4-methoxyphenyl)amino)-2-(1-methylcyclohexyl)acetate (7u)

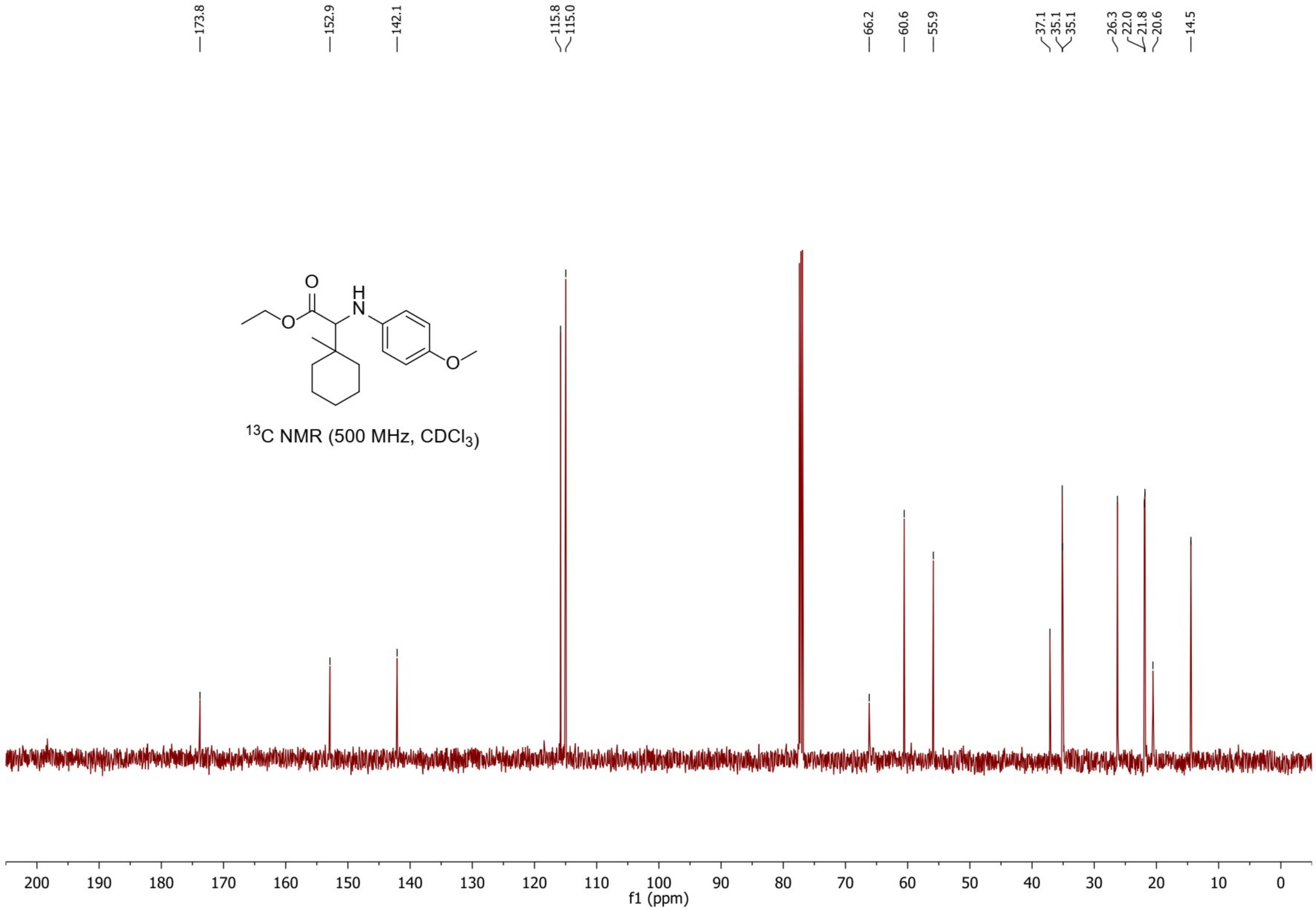

Go to table of contents

Ethyl 2-((4-methoxyphenyl)amino)-2-(4-methyltetrahydro-2*H*-pyran-4-yl)acetate (7v)

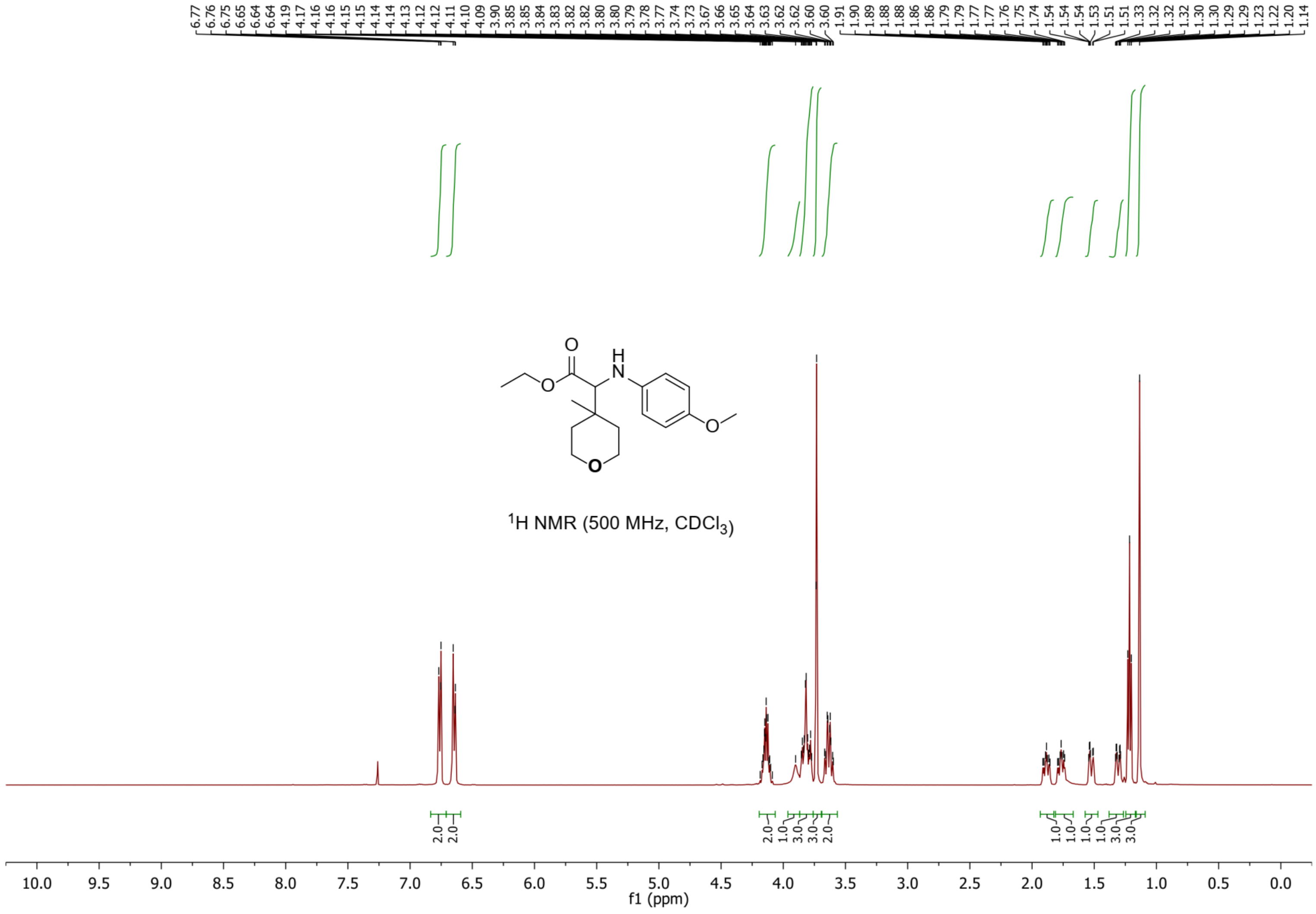

Go to table of contents

Ethyl 2-((4-methoxyphenyl)amino)-2-(4-methyltetrahydro-2*H*-pyran-4-yl)acetate (7v)

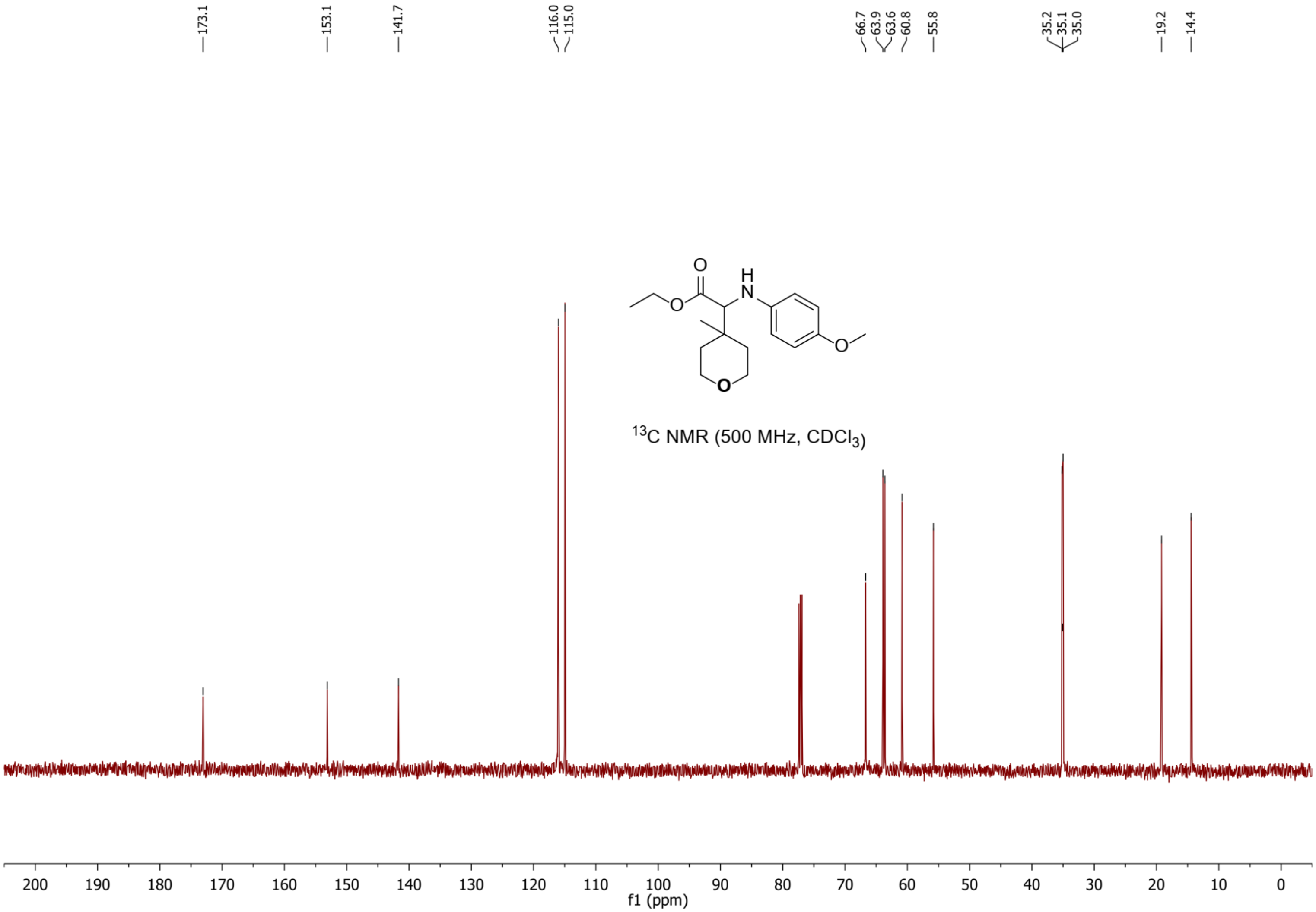

Go to table of contents

Ethyl 2-((4-methoxyphenyl)amino)-2-(1-phenylcyclohexyl)acetate (7w)

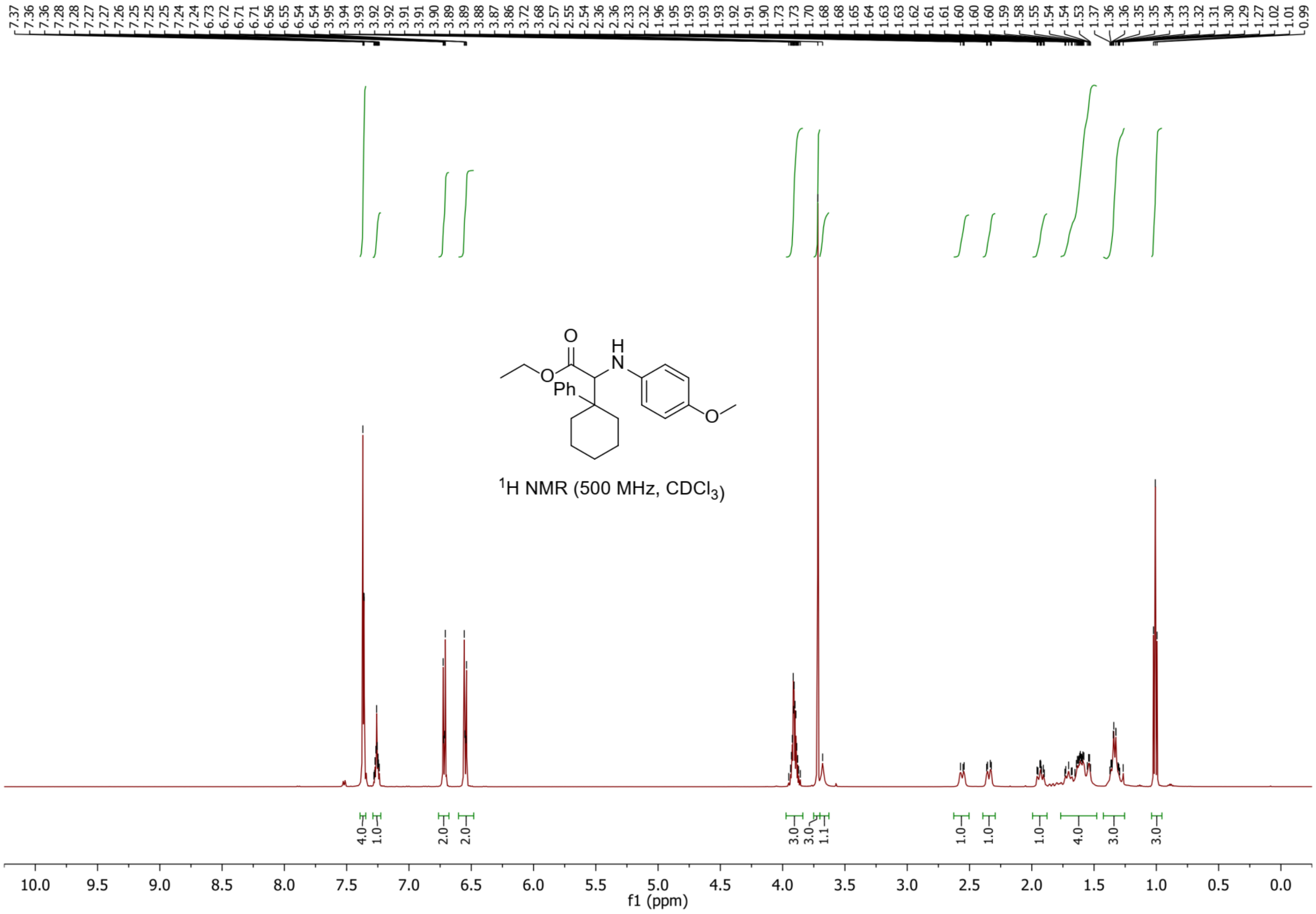

Go to table of contents

Ethyl 2-((4-methoxyphenyl)amino)-2-(1-phenylcyclohexyl)acetate (7w)

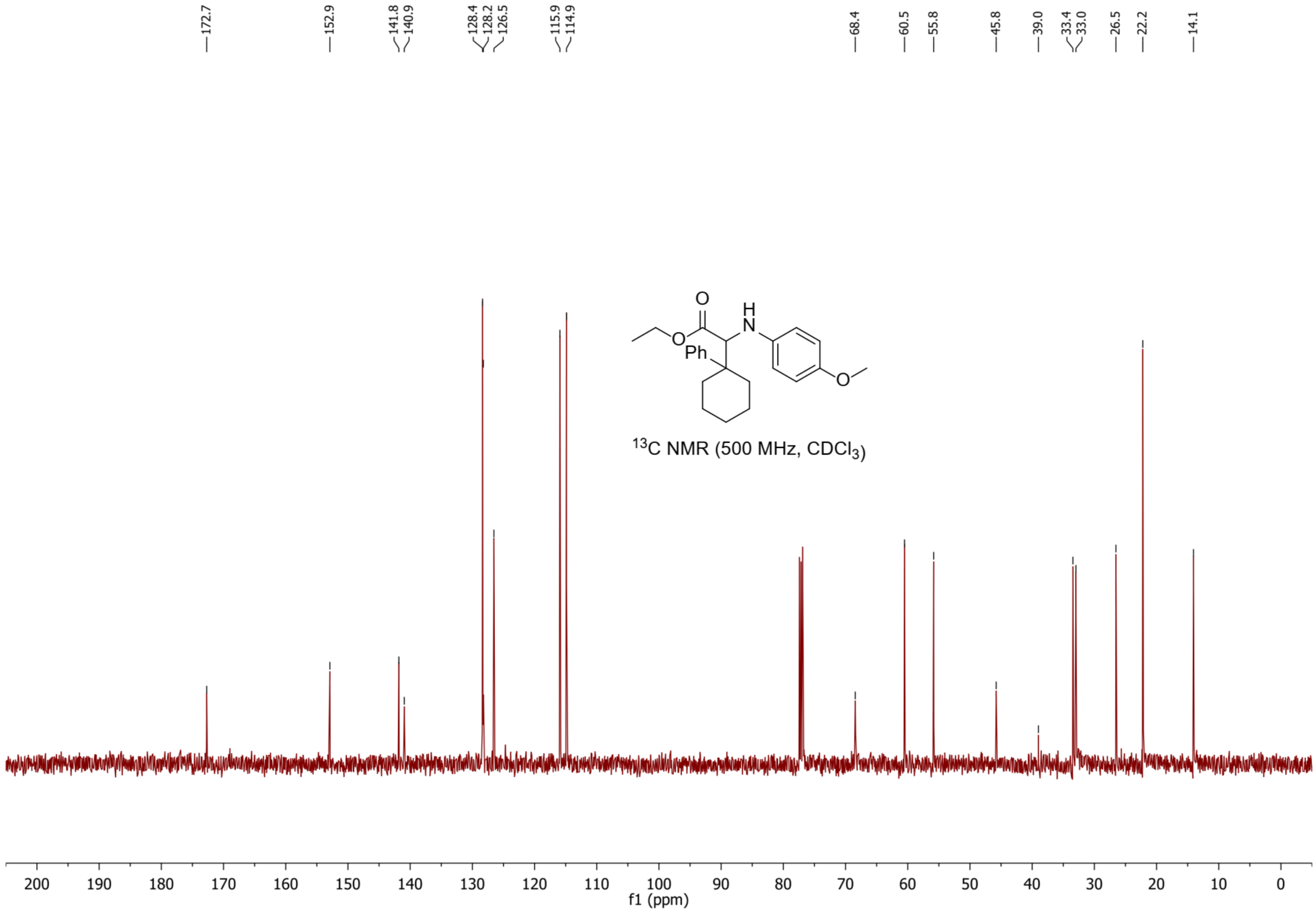

Go to table of contents

Ethyl 2-((4-methoxyphenyl)amino)-3-methyl-3-phenylbutanoate (7x)

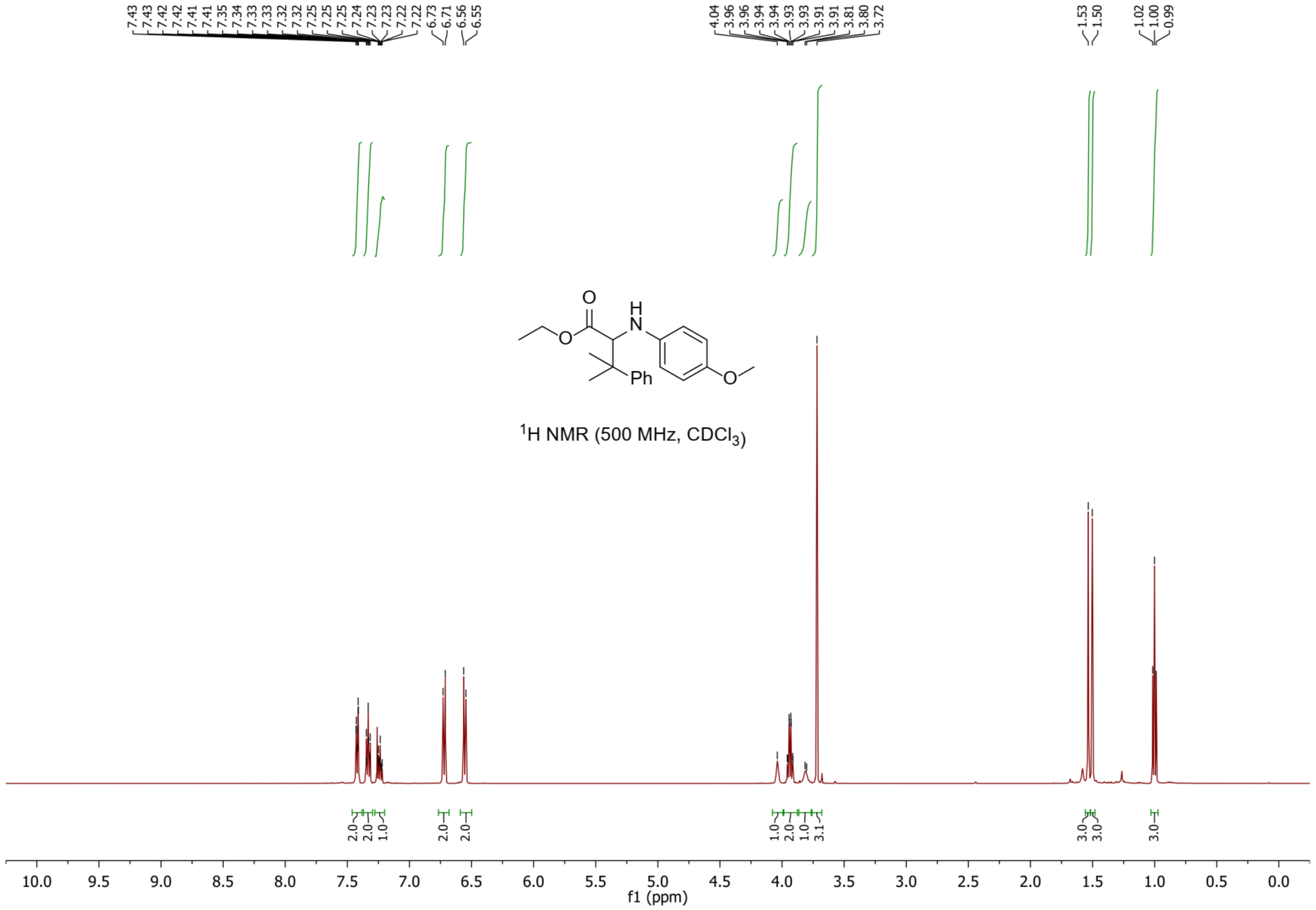

Ethyl 2-((4-methoxyphenyl)amino)-3-methyl-3-phenylbutanoate (7x)

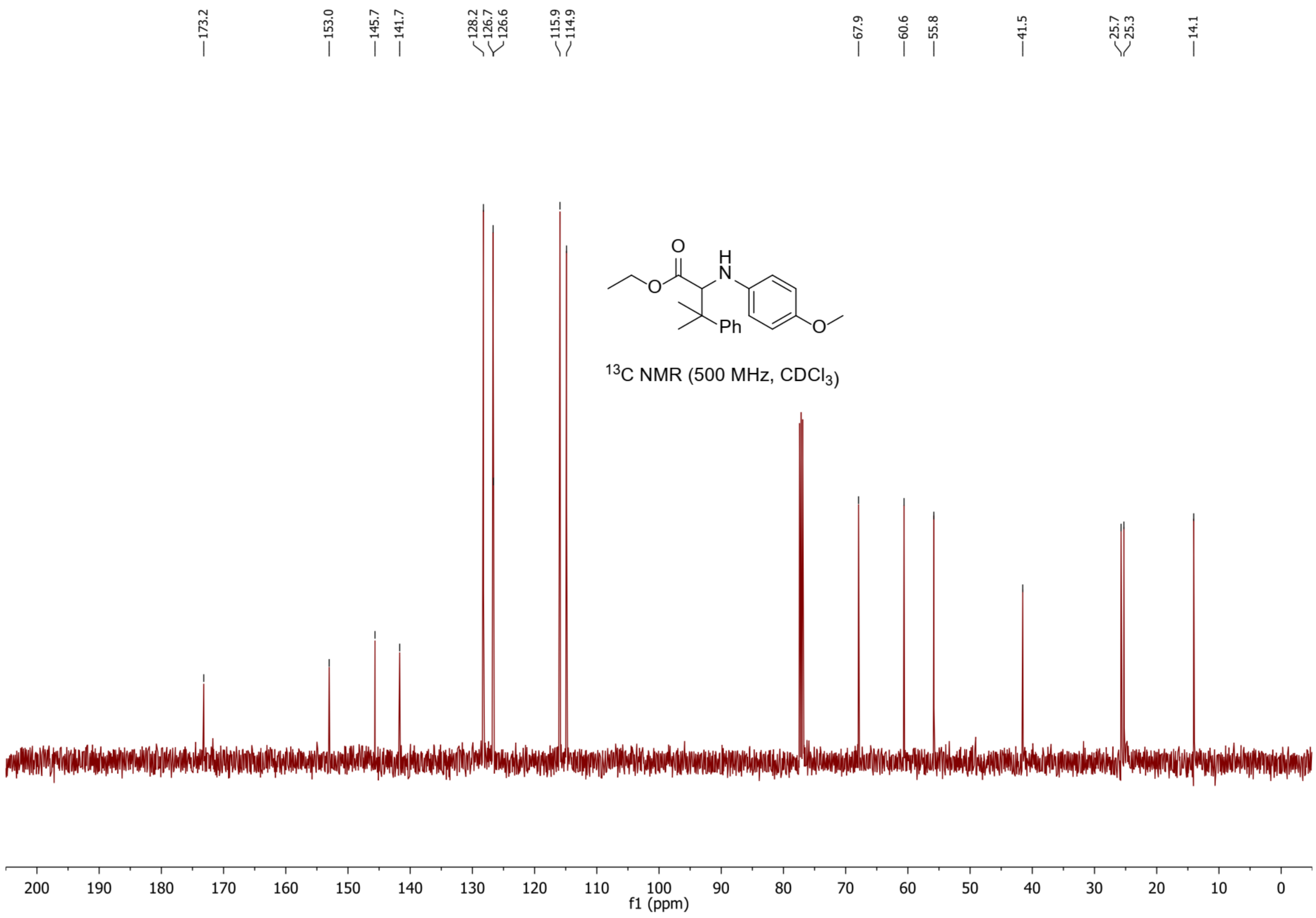

Go to table of contents

Ethyl 2-((3,5,7)-adamantan-1-yl)-2-((4-methoxyphenyl)amino)acetate (7y)

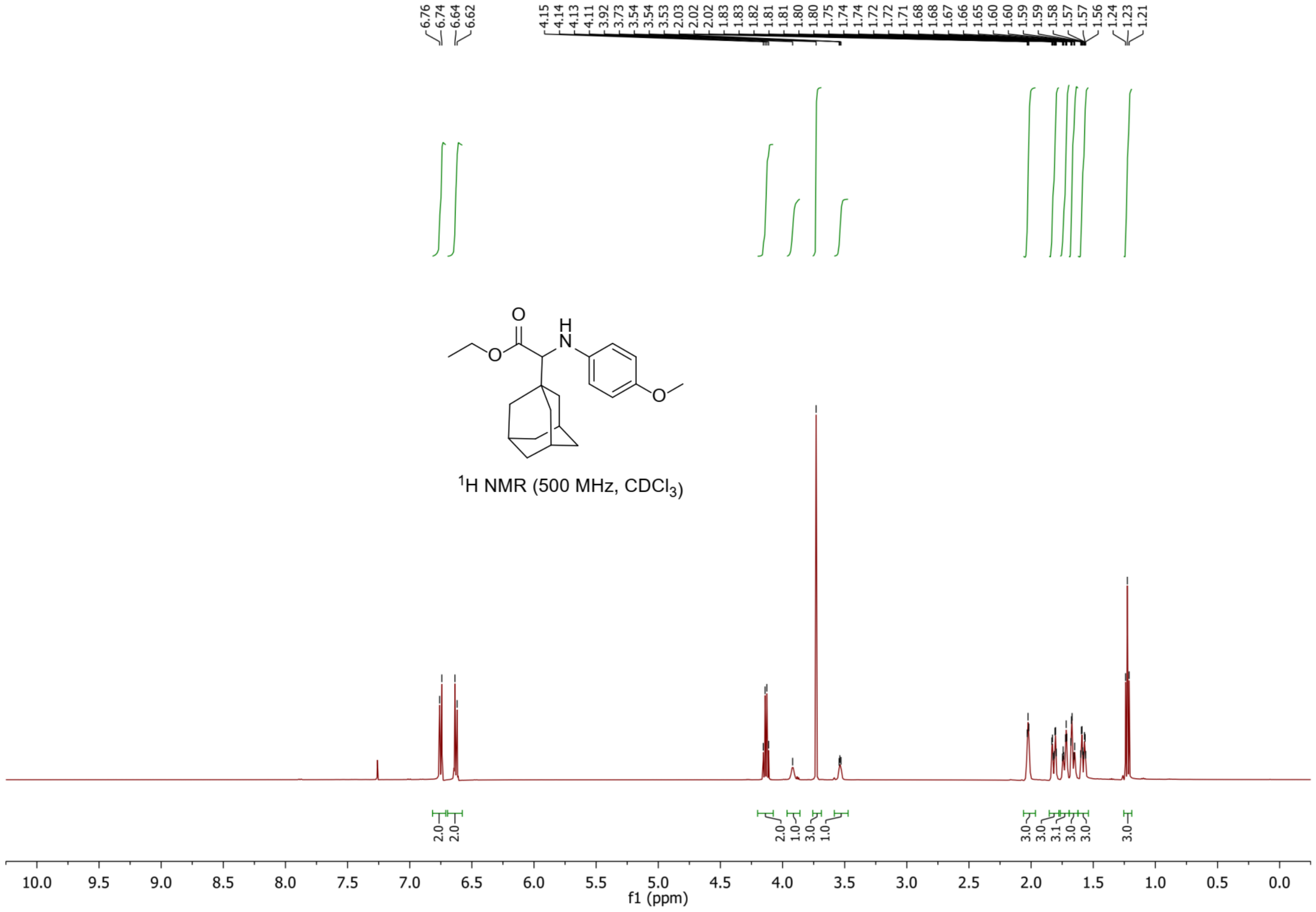

Go to table of contents

Ethyl 2-((3,5,7)-adamantan-1-yl)-2-((4-methoxyphenyl)amino)acetate (7y)

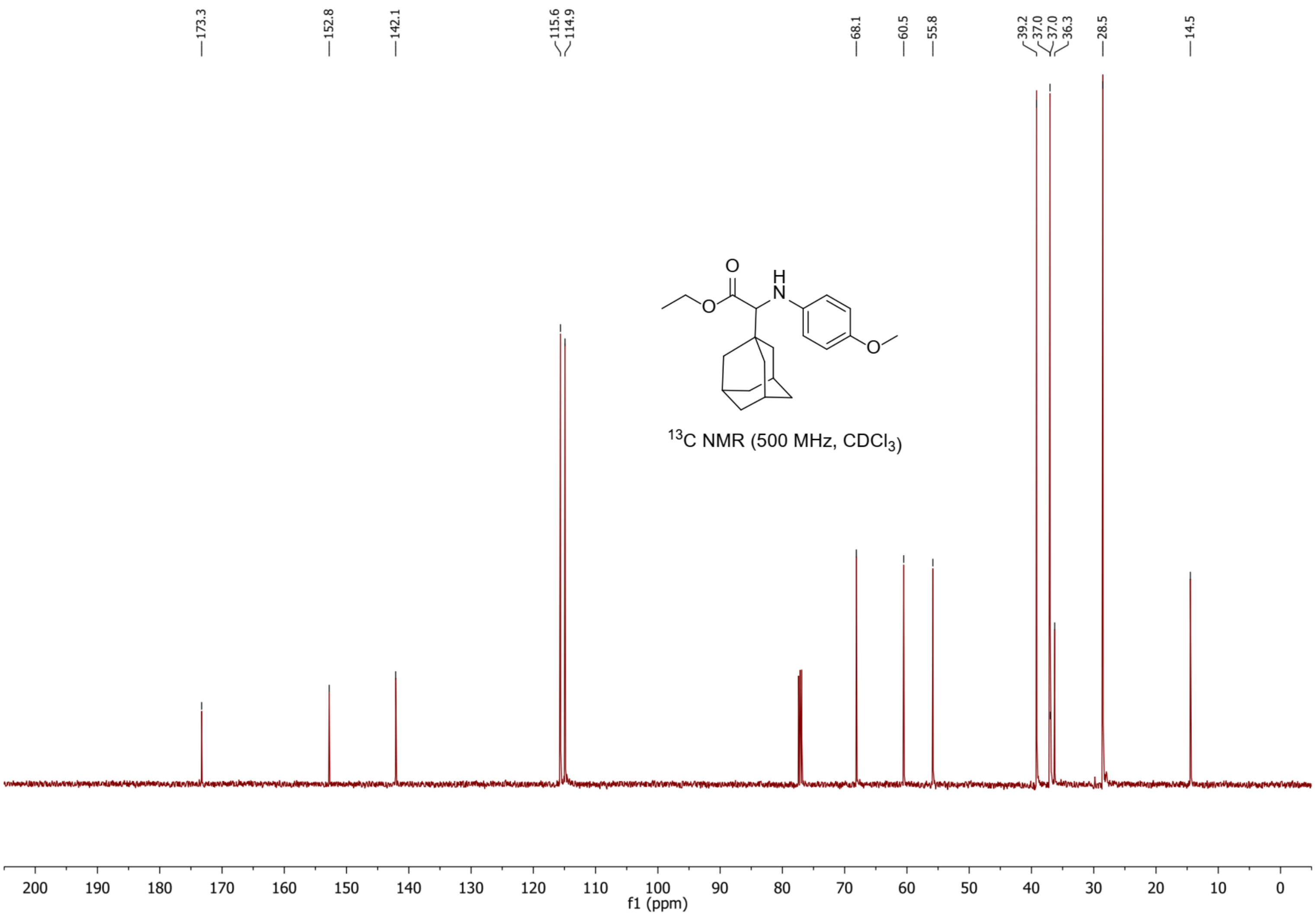

Go to table of contents

Isopropyl 2-cyclohexyl-2-((4-methoxyphenyl)amino)acetate (7z)

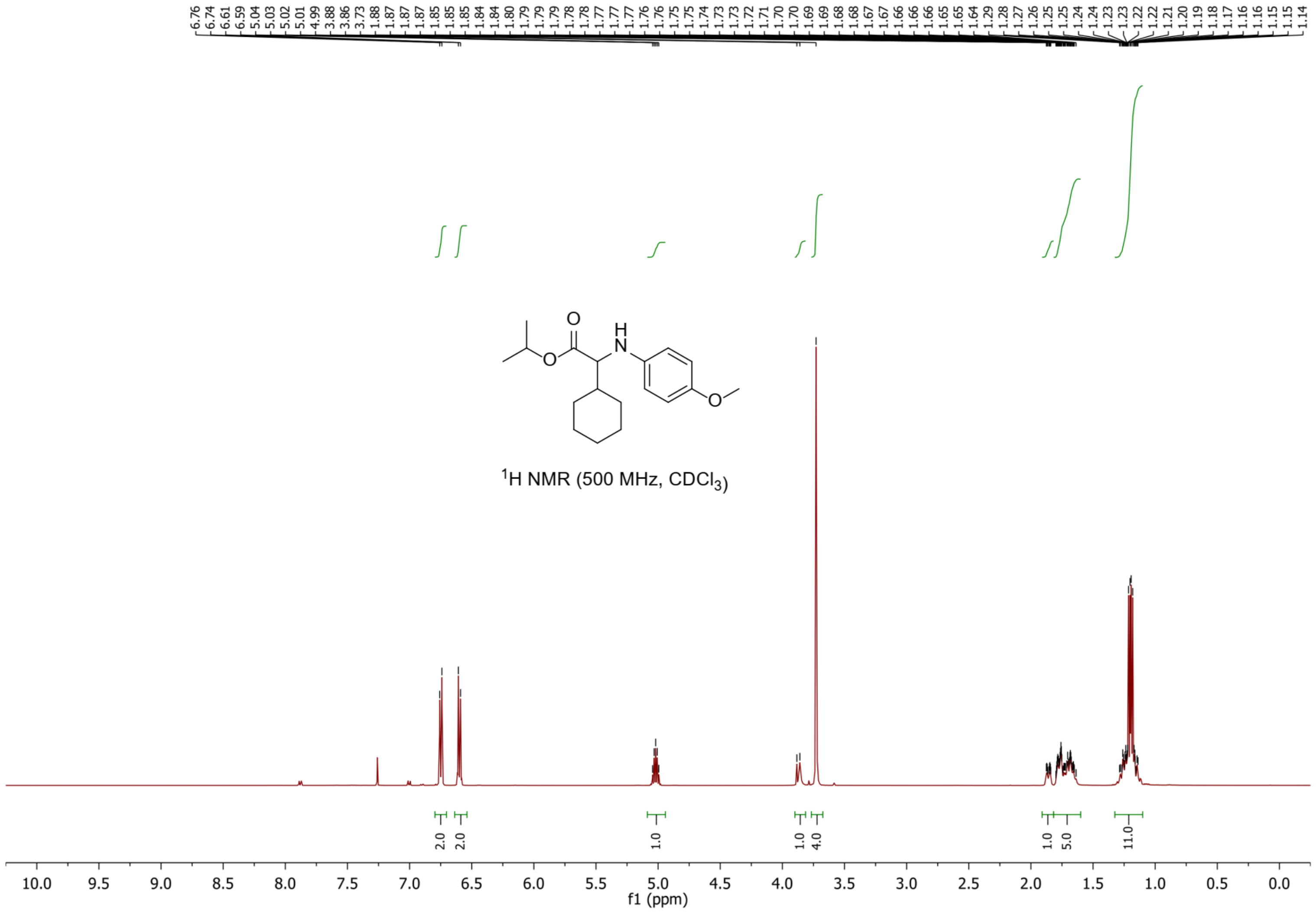

Go to table of contents

Isopropyl 2-cyclohexyl-2-((4-methoxyphenyl)amino)acetate (7z)

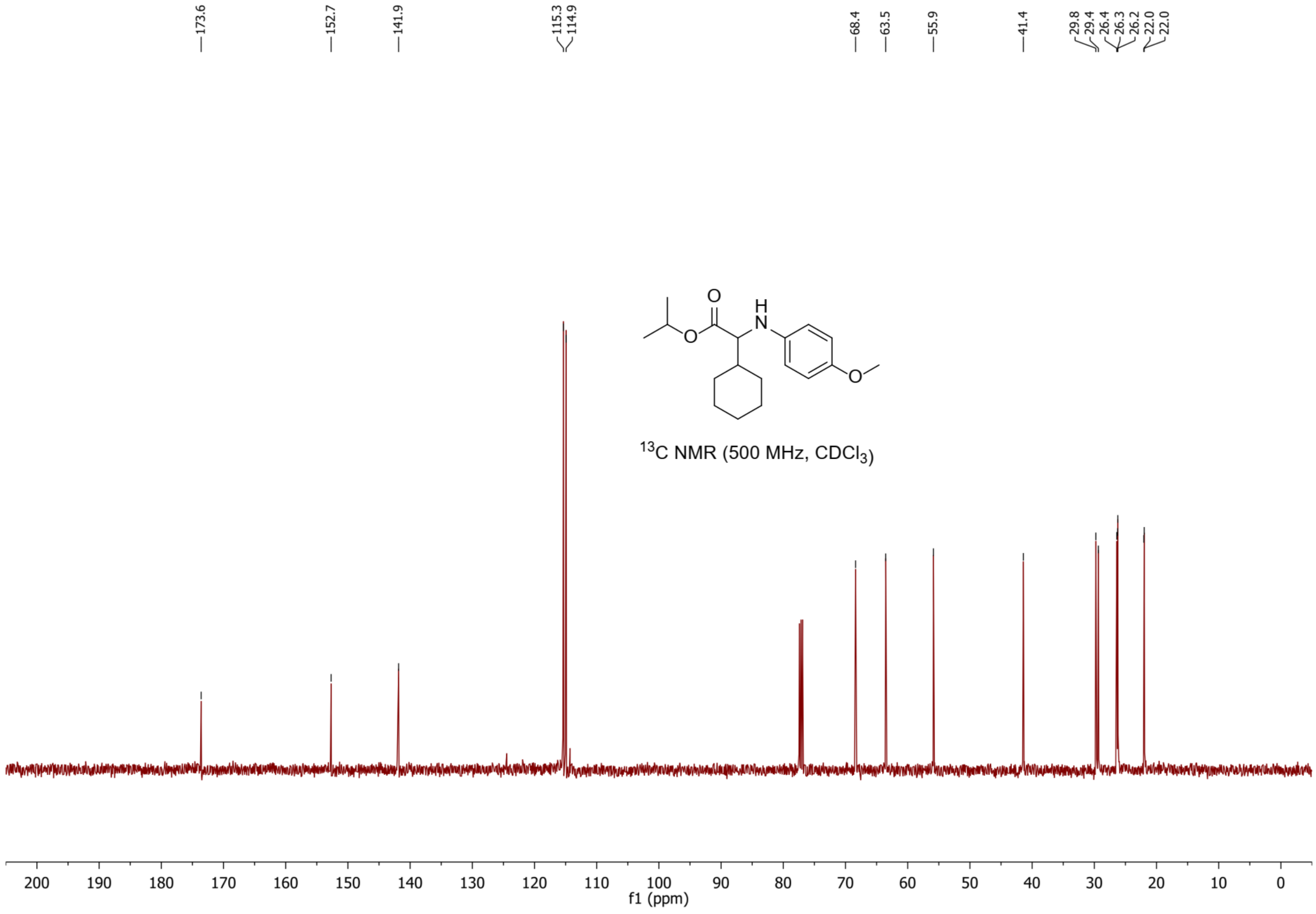

Go to table of contents

Benzyl 2-cyclohexyl-2-((4-methoxyphenyl)amino)acetate (7za)

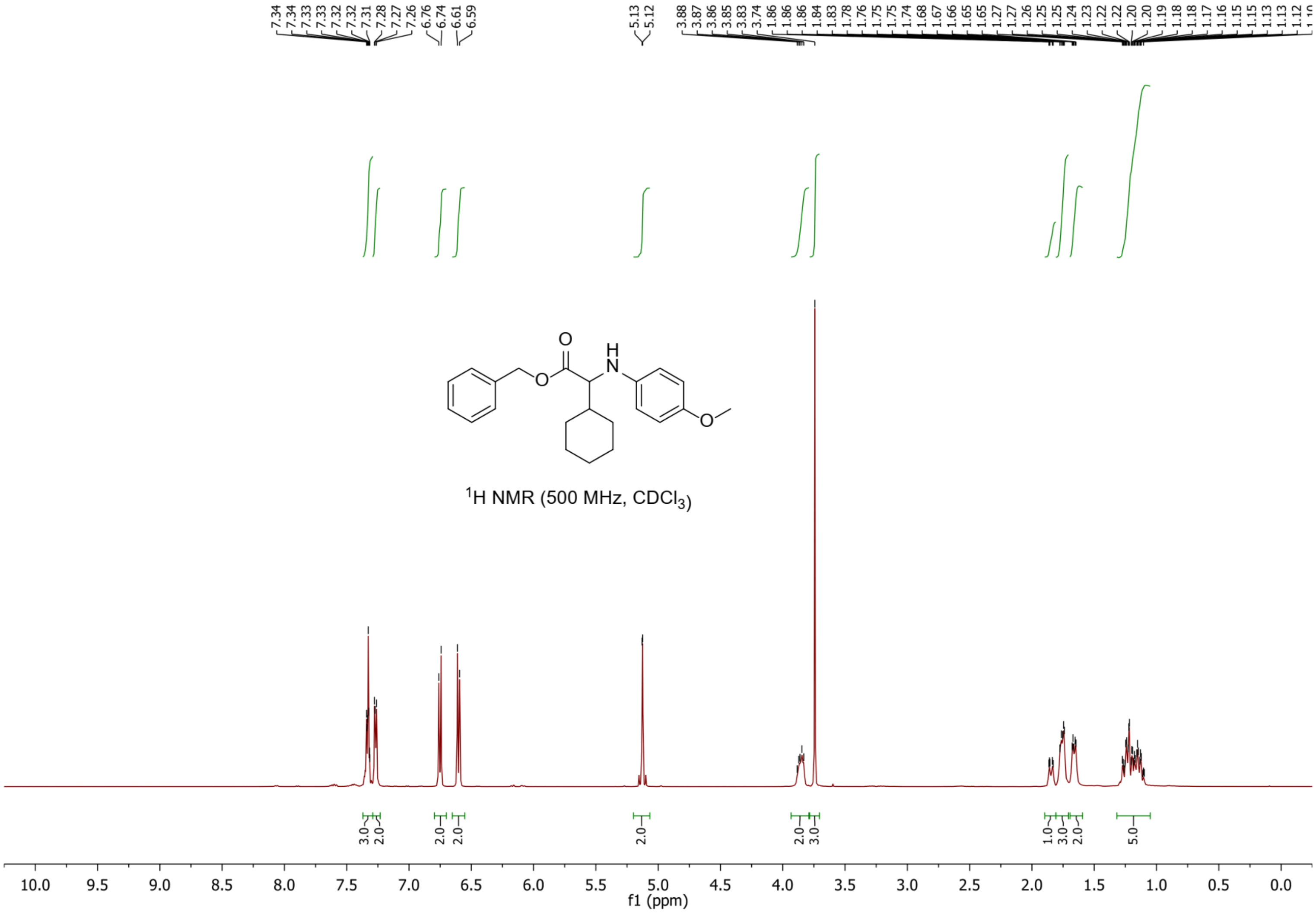

Go to table of contents

Benzyl 2-cyclohexyl-2-((4-methoxyphenyl)amino)acetate (7za)

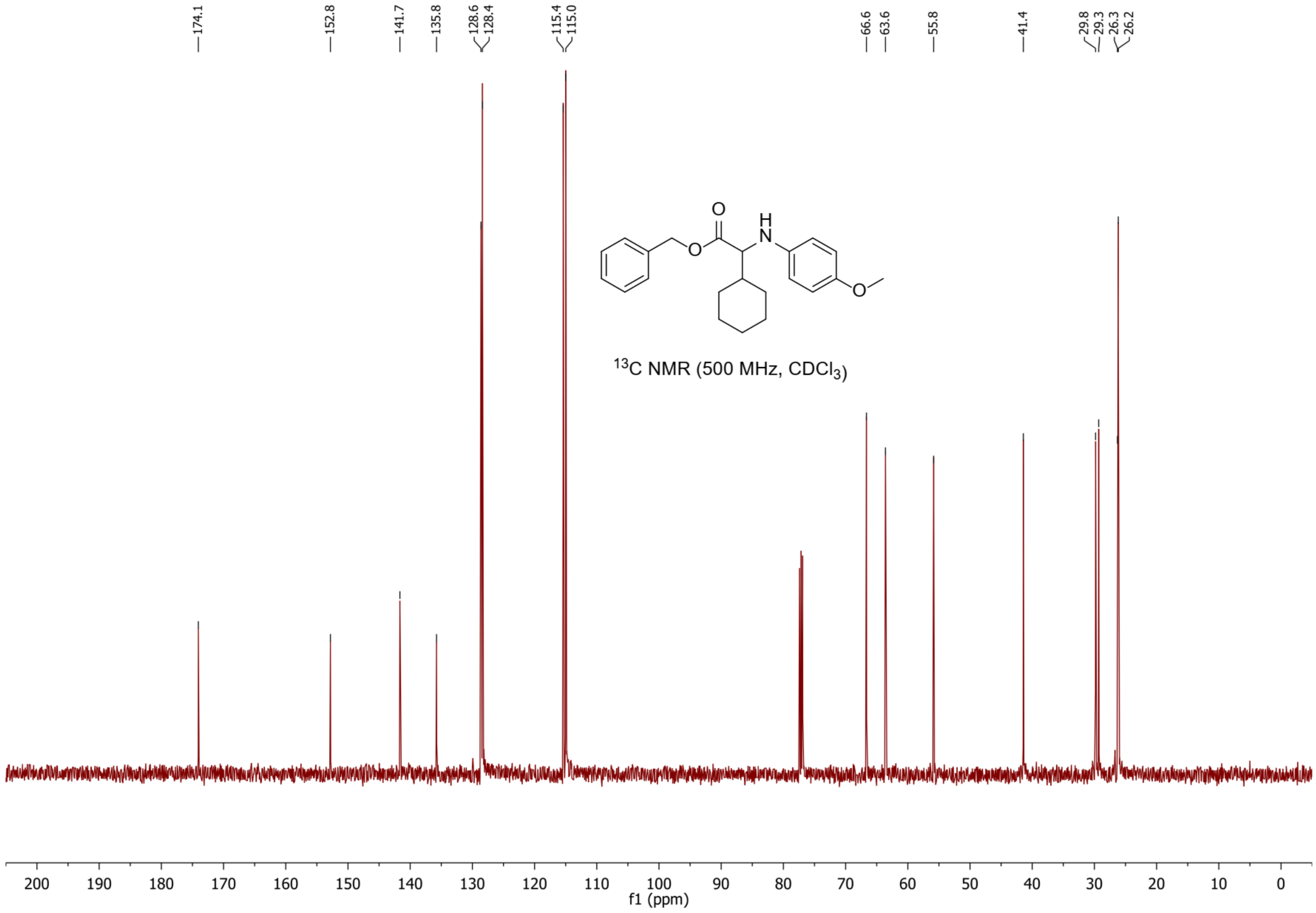

Go to table of contents

_N_-(5-(2,5-Dimethylphenoxy)-2,2-dimethyl-1-phenylpentyl)aniline (8a)

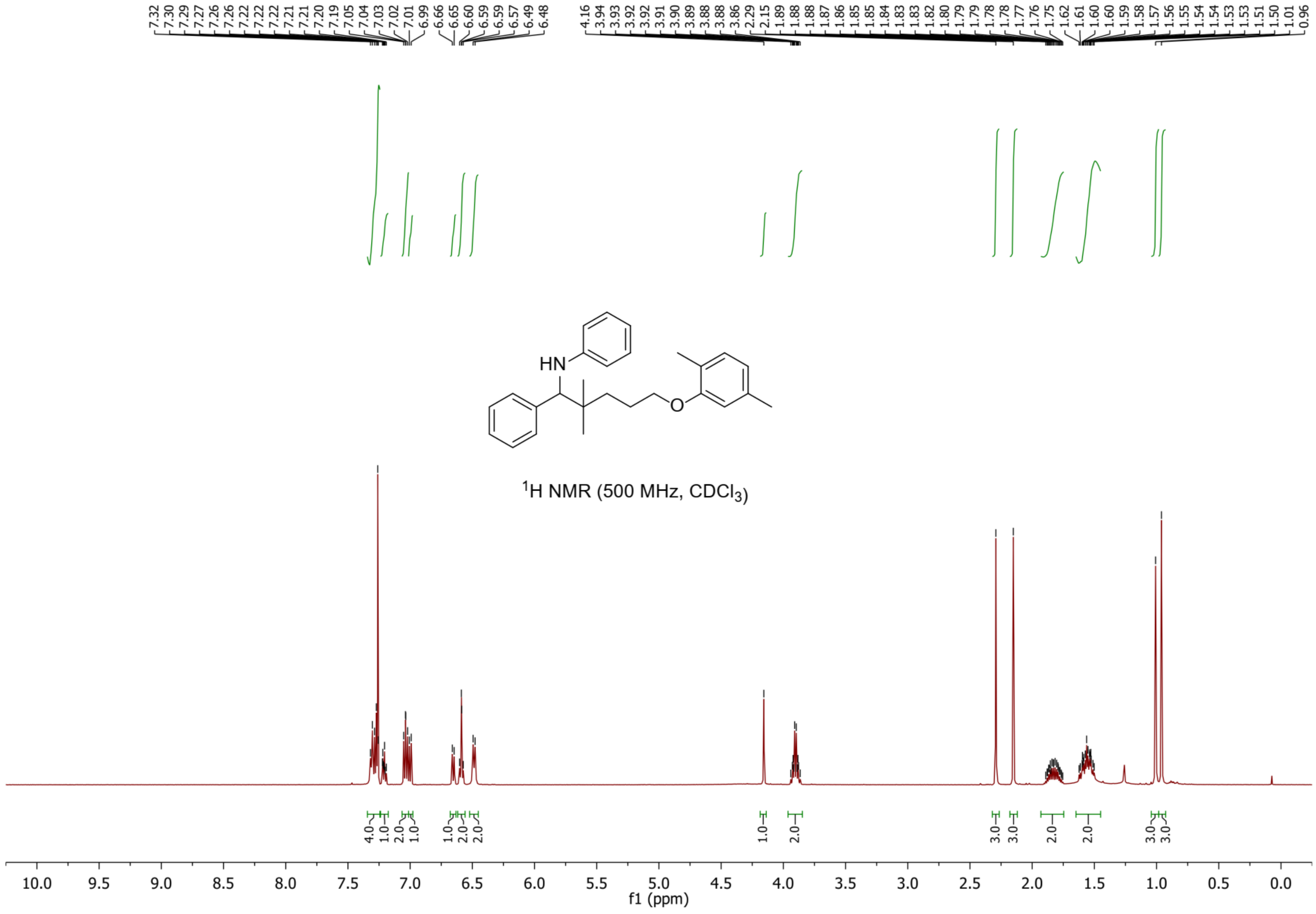

Go to table of contents

N-(5-(2,5-Dimethylphenoxy)-2,2-dimethyl-1-phenylpentyl)aniline (8a)

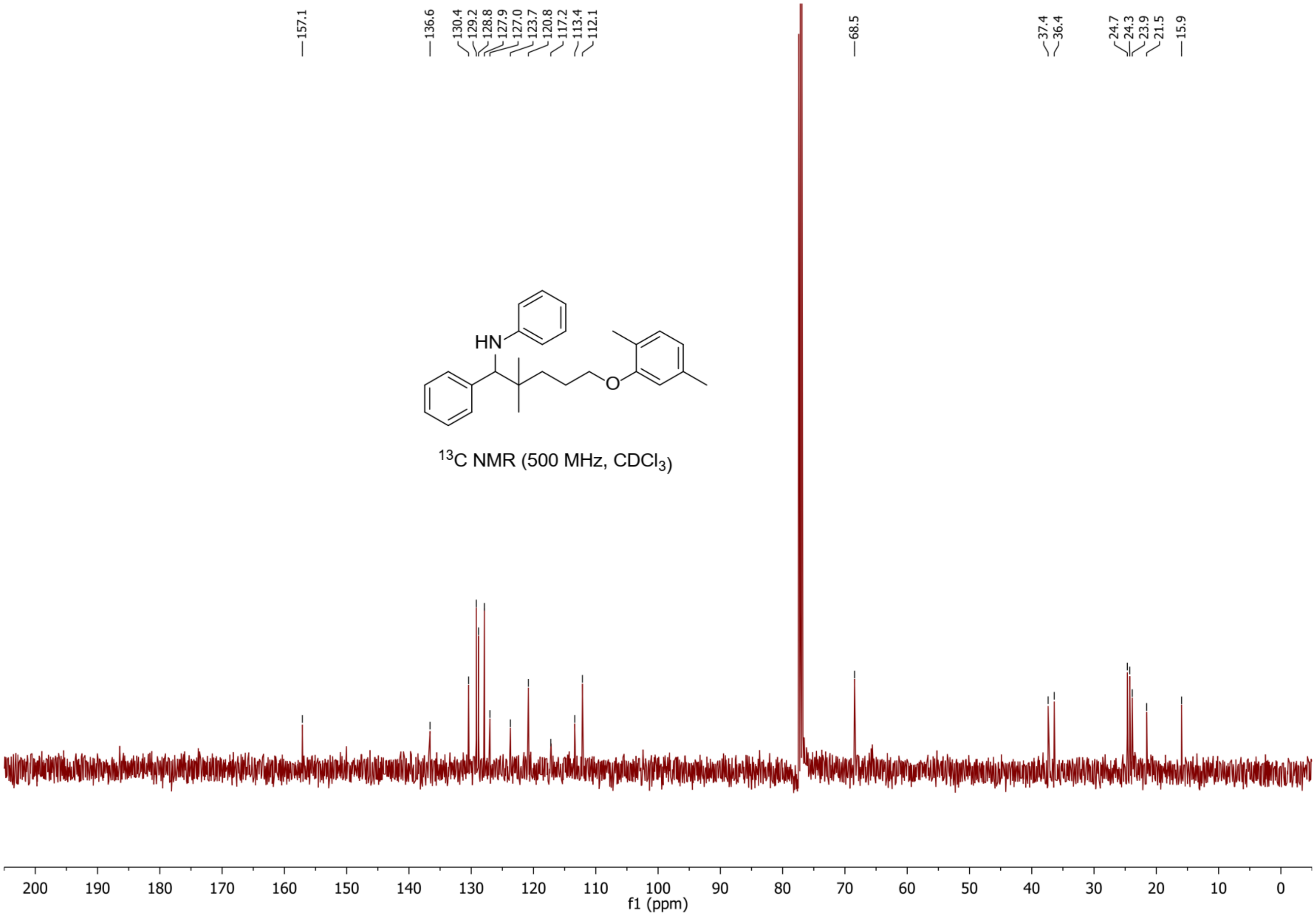

Ethyl 6-(2,5-dimethylphenoxy)-2-((4-methoxyphenyl)amino)-3,3-dimethylhexanoate (8b)

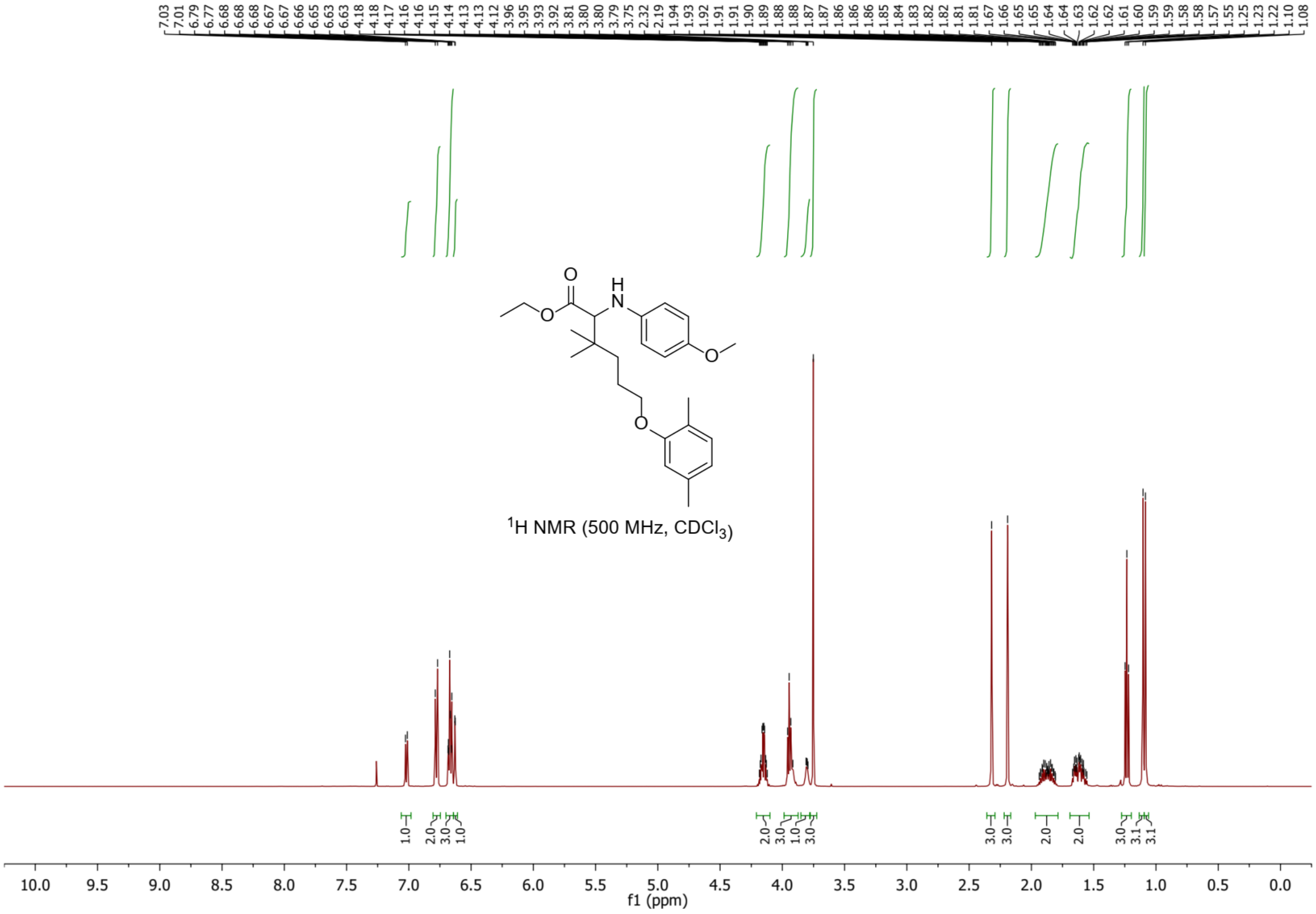

Go to table of contents

Ethyl 6-(2,5-dimethylphenoxy)-2-((4-methoxyphenyl)amino)-3,3-dimethylhexanoate (8b)

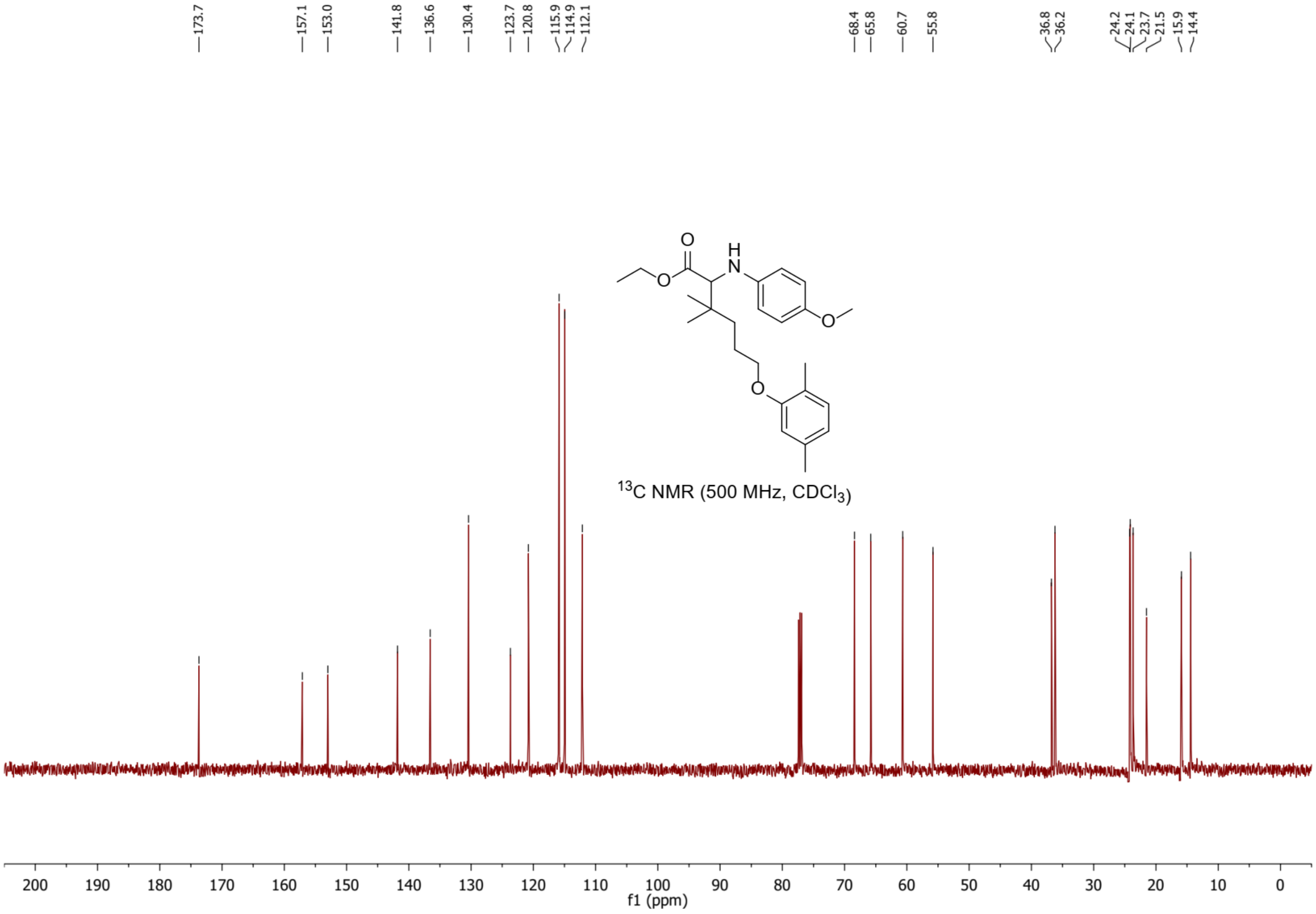

Go to table of contents

Ethyl 2-((4-methoxyphenyl)amino)-3-(11-oxo-6,11-dihydrodibenzo[b,e]oxepin-2-yl)propanoate (8c)

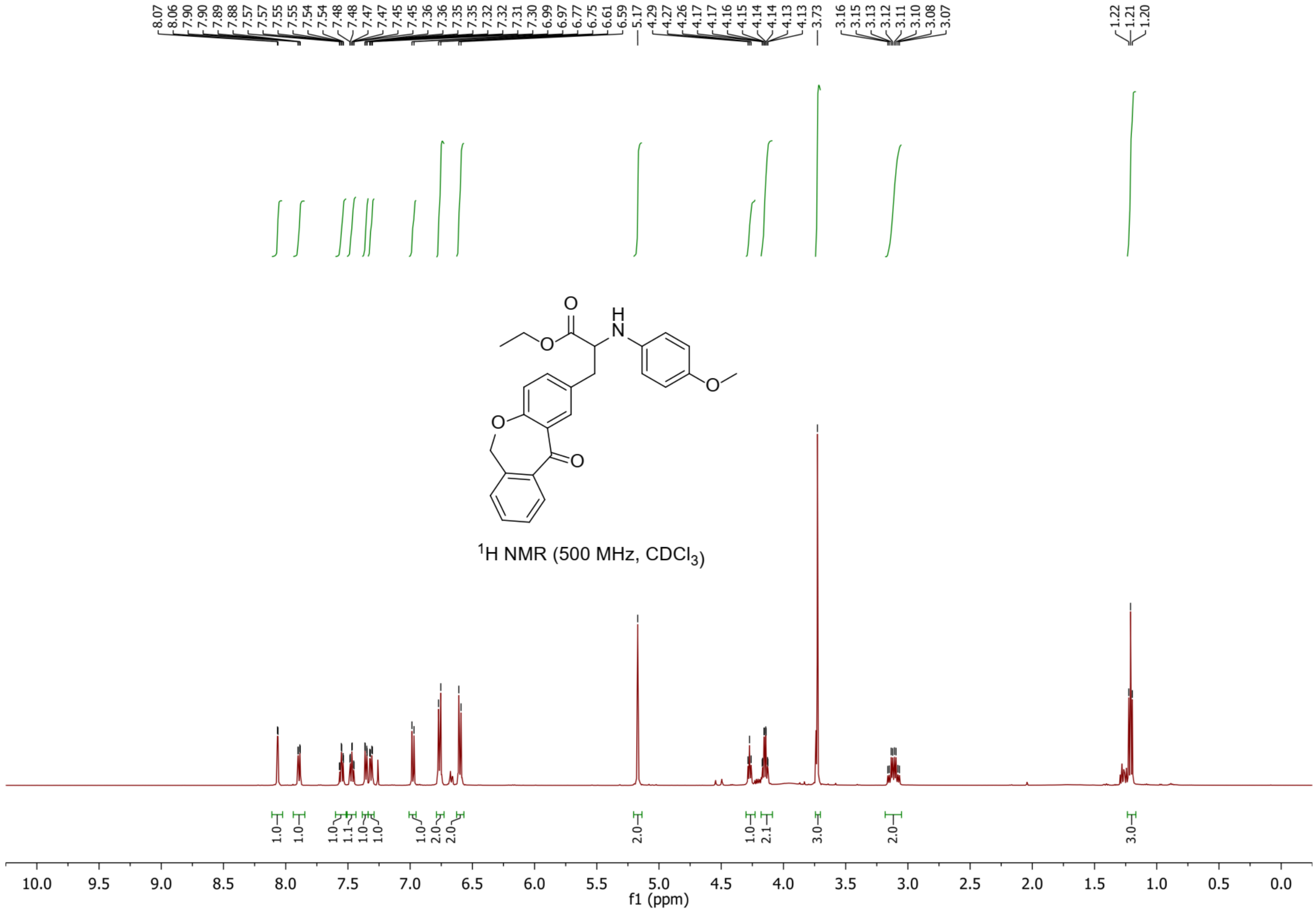

Go to table of contents

Ethyl 2-((4-methoxyphenyl)amino)-3-(11-oxo-6,11-dihydrodibenzo[*b*,*e*]oxepin-2-yl)propanoate (8c)

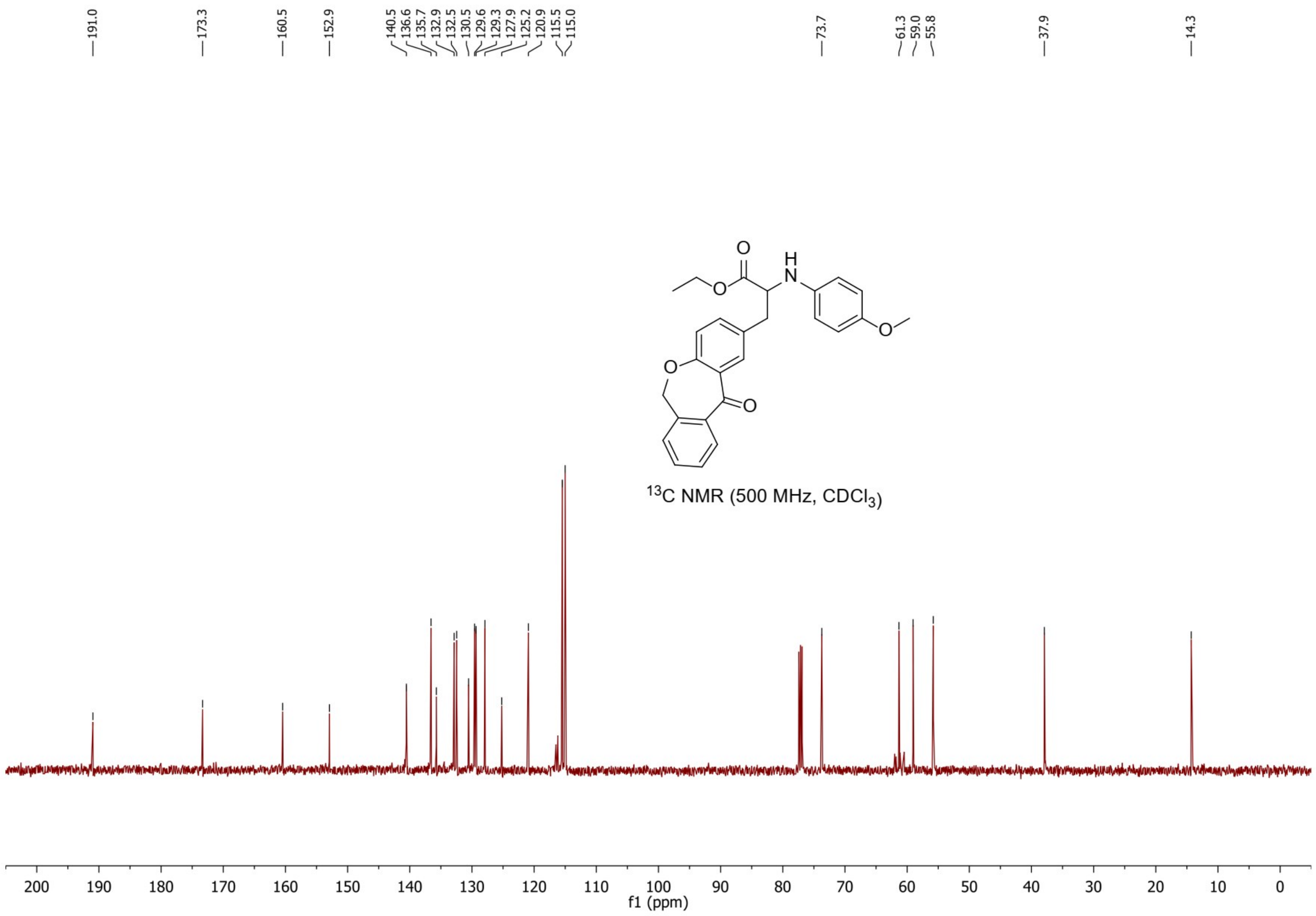

Go to table of contents

Ethyl 3-(1-(4-chlorobenzoyl)-5-methoxy-2-methyl-1*H*-indol-3-yl)-2-((4-methoxyphenyl)amino)propanoate (8d)

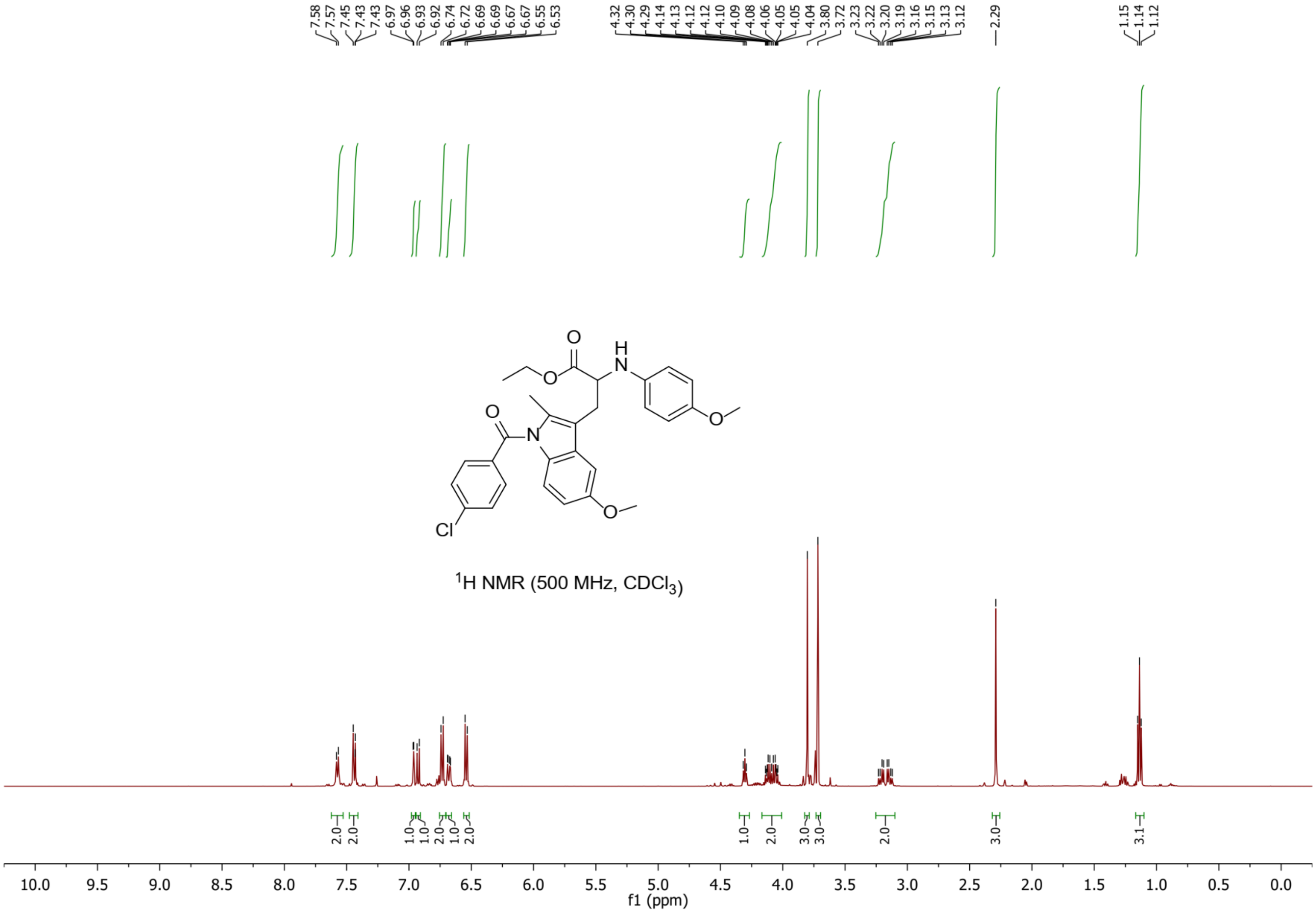

Go to table of contents

Ethyl 3-(1-(4-chlorobenzoyl)-5-methoxy-2-methyl-1*H*-indol-3-yl)-2-((4-methoxyphenyl)amino)propanoate (8d)

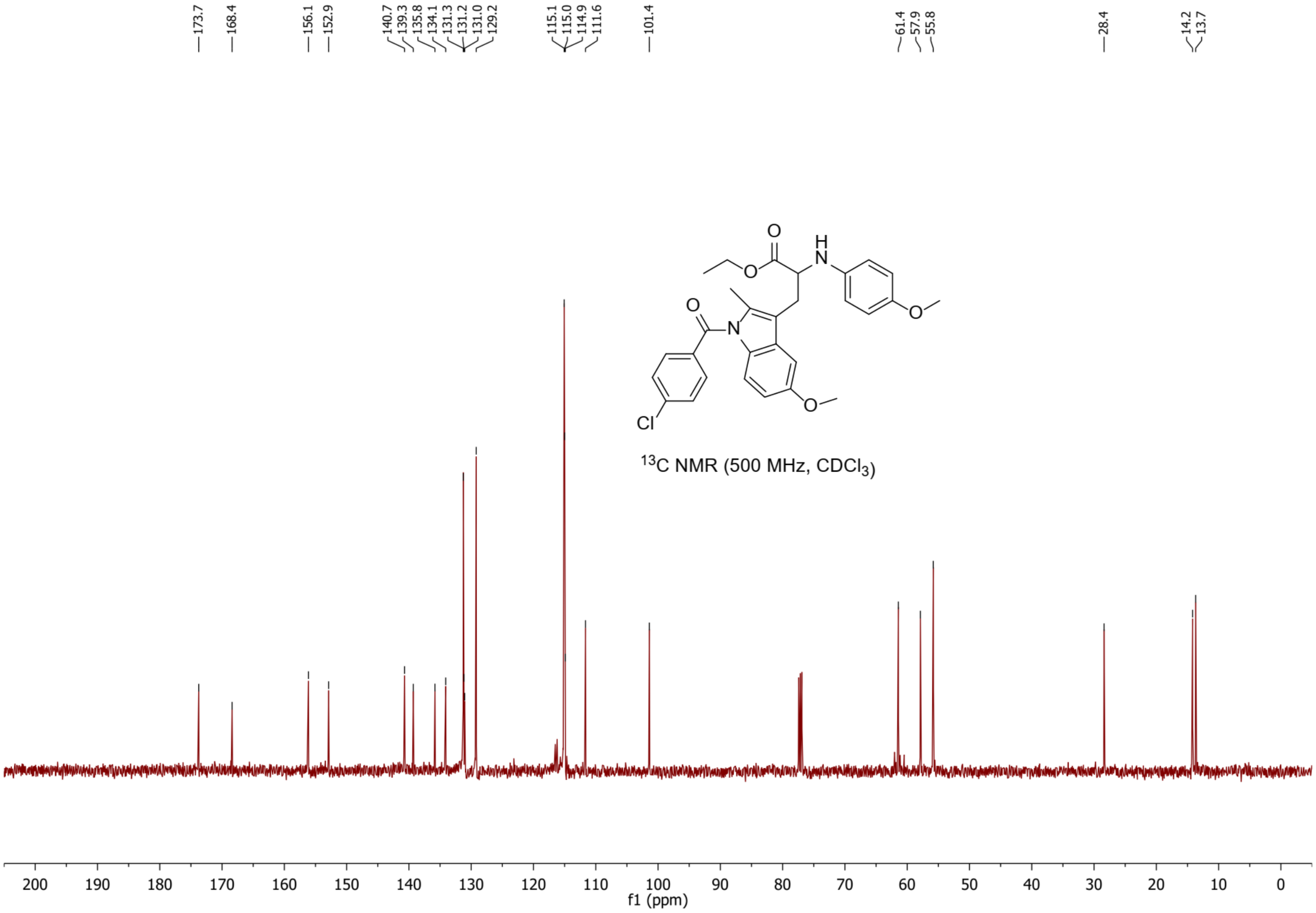

Go to table of contents

(3*R*,7*R*,8*R*,9*R*,10*S*,13*R*,14*R*,17*R*)-17-((2*R*)-6-Ethoxy-5-((4-methoxyphenyl)amino)-6-oxohexan-2-yl)-8,10,13-trimethylhexadecahydro-1H-cyclopenta[*a*]phenanthrene-3,7-diyl diacetate (8e)

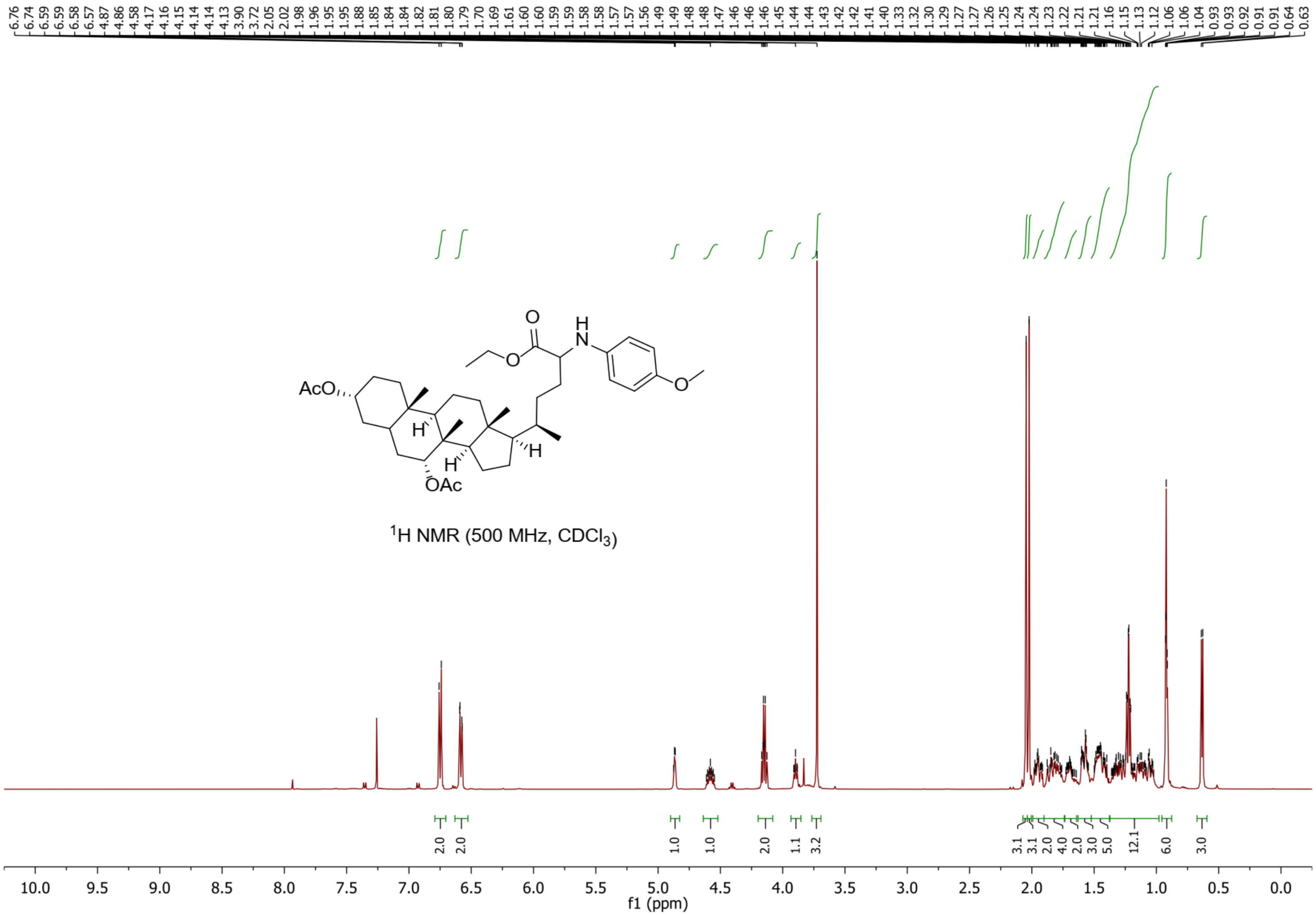

^{1}H NMR (500 MHz, $CDCl_3$)

Go to table of contents

(3*R*,7*R*,8*R*,9*R*,10*S*,13*R*,14*R*,17*R*)-17-((2*R*)-6-Ethoxy-5-((4-methoxyphenyl)amino)-6-oxohexan-2-yl)-8,10,13-trimethylhexadecahydro-1H-cyclopenta[*a*]phenanthrene-3,7-diyl diacetate (8e)

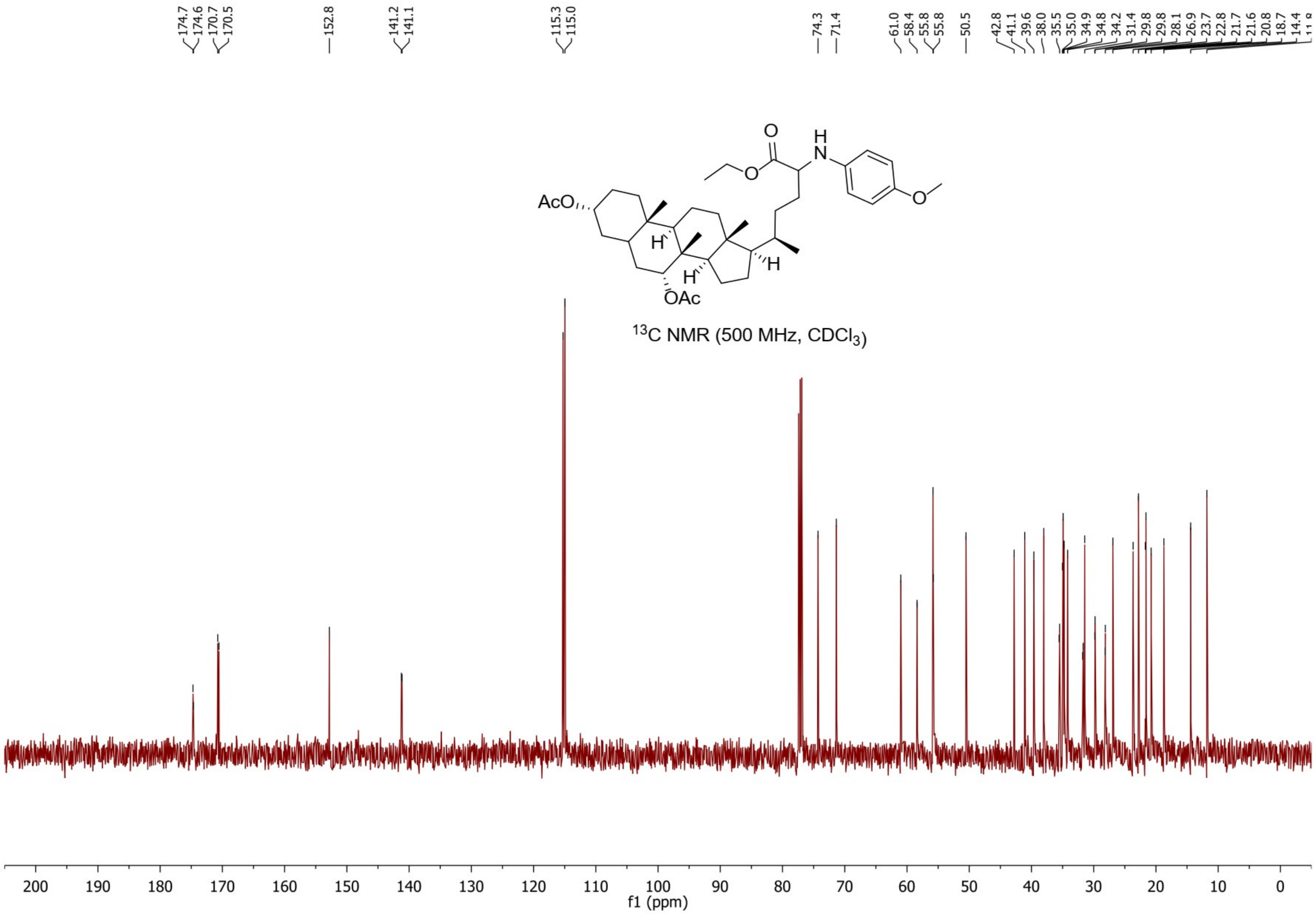

Go to table of contents

1-Cyclohexyl 5-ethyl (2*S*)-2-((tert-butoxycarbonyl)amino)-4-((4-methoxyphenyl)amino)pentanedioate (8f)

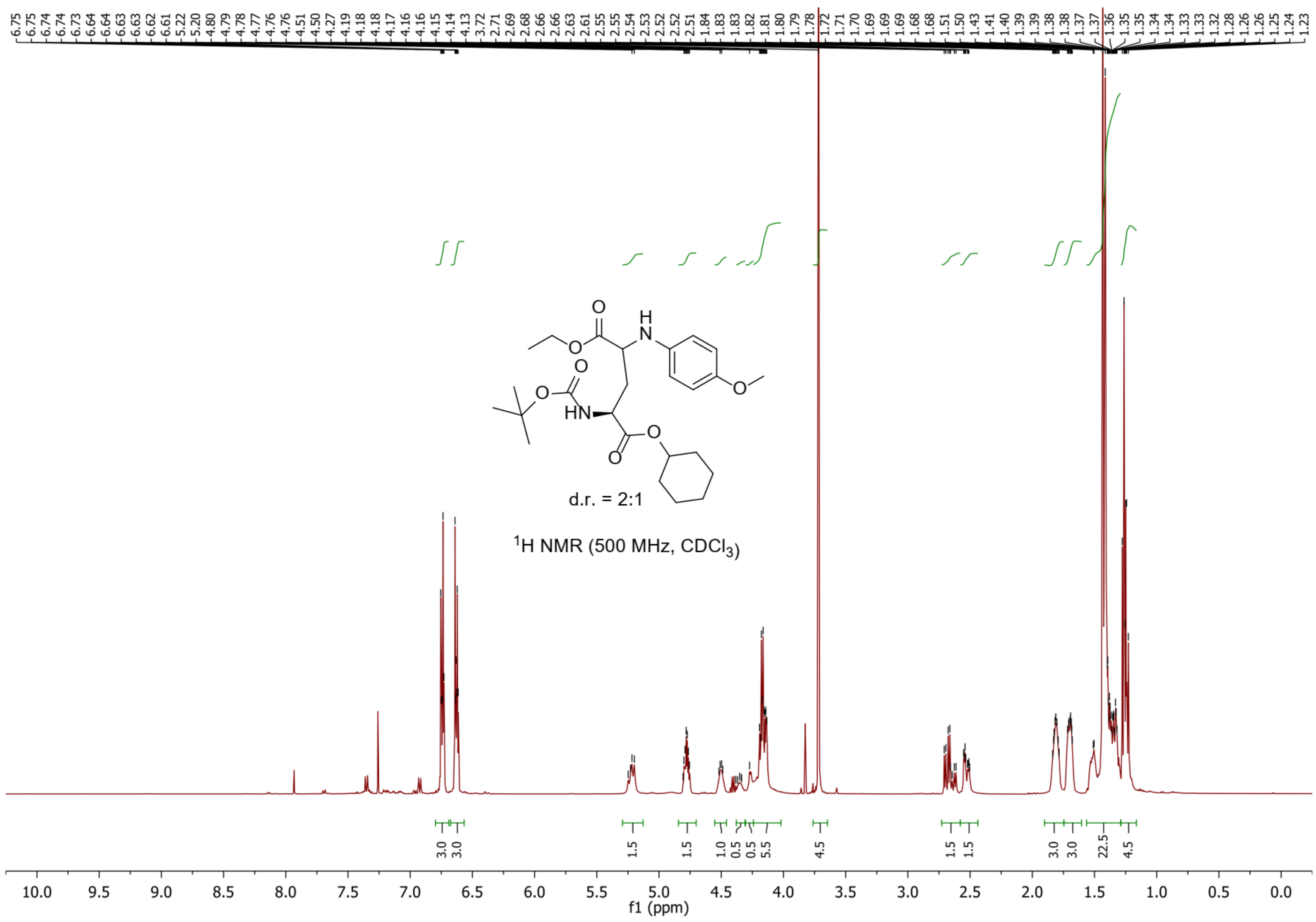

Go to table of contents

1-Cyclohexyl 5-ethyl (2*S*)-2-((tert-butoxycarbonyl)amino)-4-((4-methoxyphenyl)amino)pentanedioate (8f)

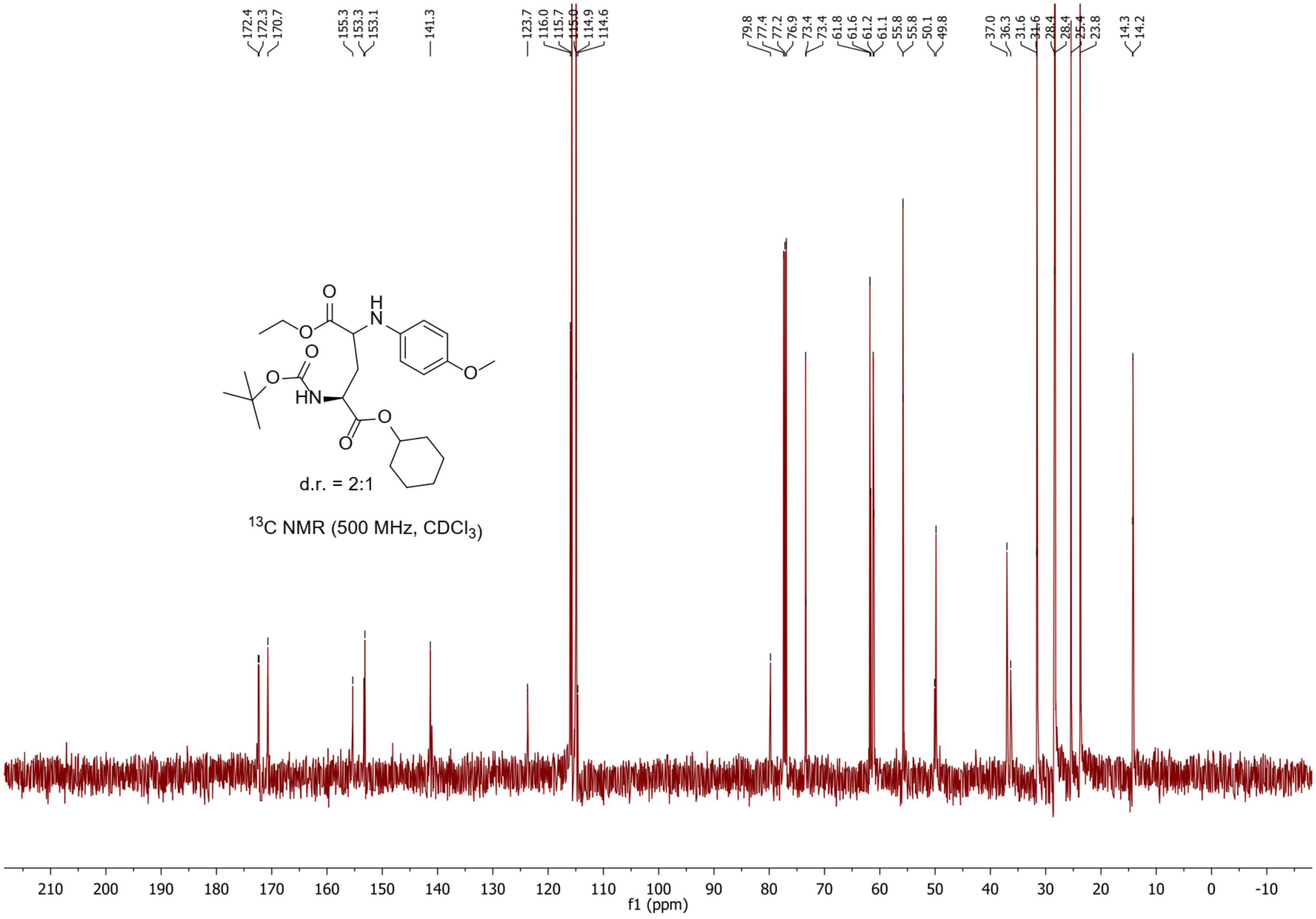

Go to table of contents

1-Benzyl 6-ethyl (2*S*)-2-(((benzyloxy)carbonyl)amino)-5-((4-methoxyphenyl)amino)hexanedioate (8g)

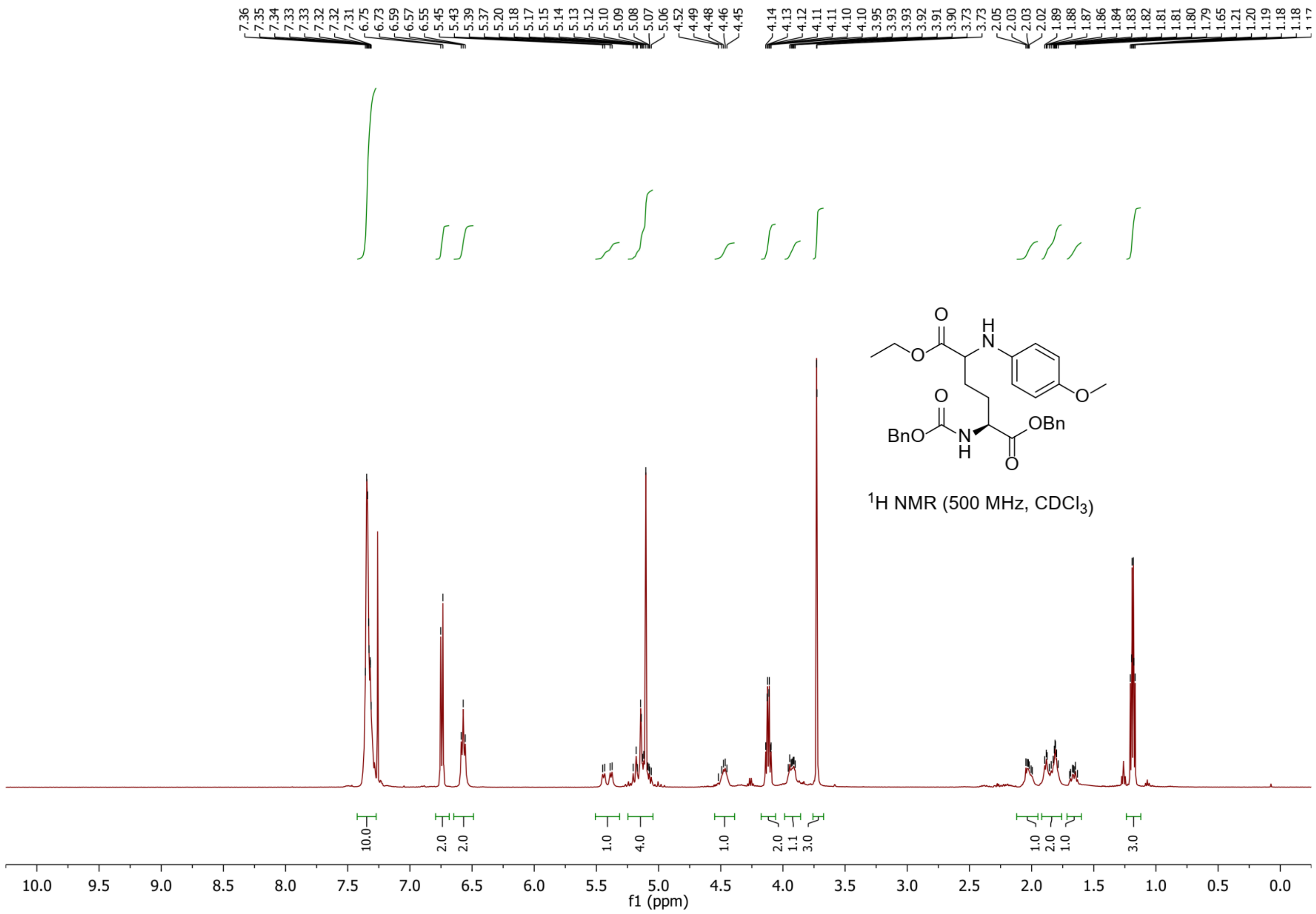

1-Benzyl 6-ethyl (2*S*)-2-(((benzyloxy)carbonyl)amino)-5-((4-methoxyphenyl)amino)hexanedioate (8g)

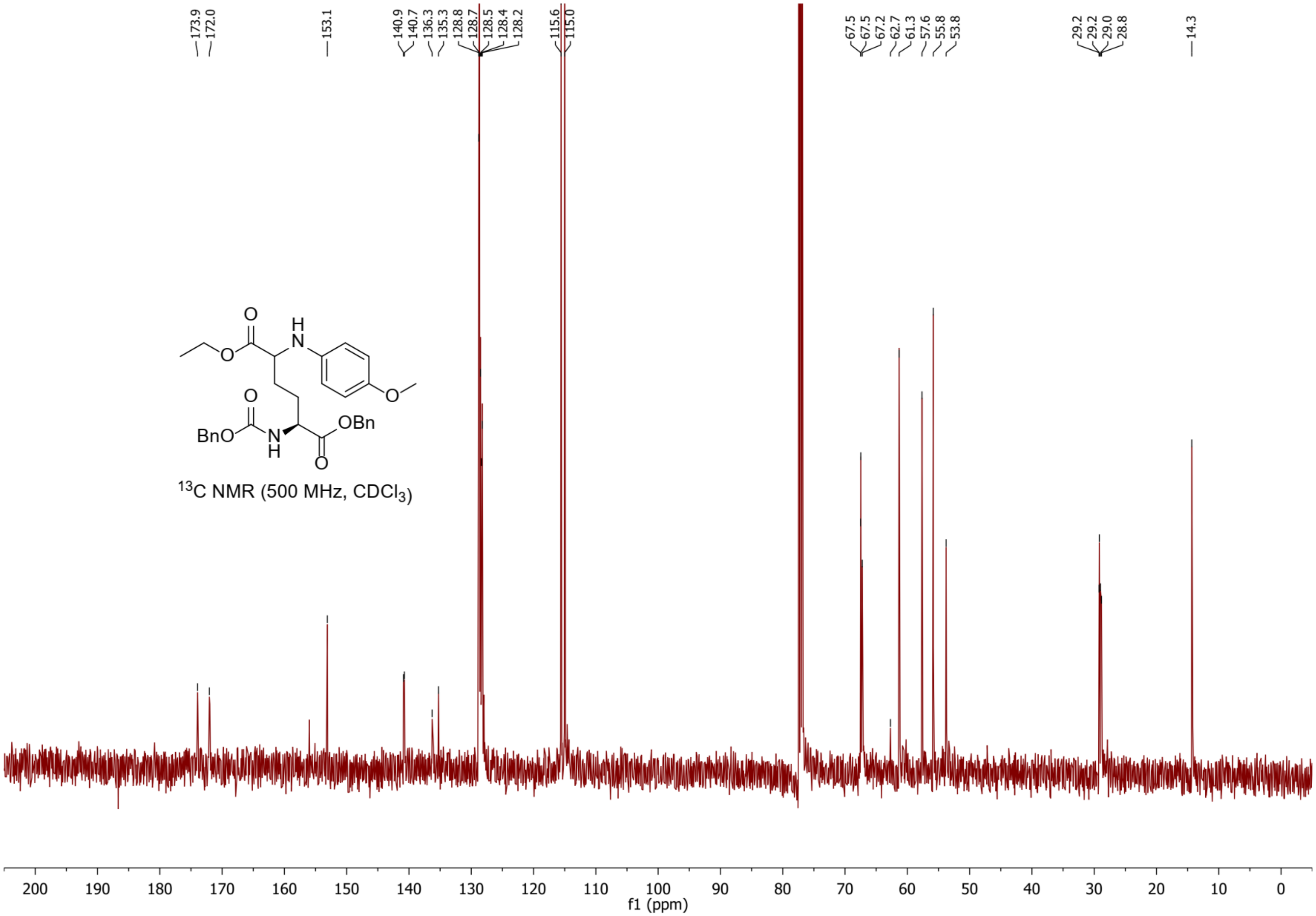

Go to table of contents

1-Ethyl 6-((3a*S*,5a*R*,8a*R*,8b*S*)-2,2,7,7-tetramethyltetrahydrobenzo[1,2-*d*:3,4-*d'*]bis([1,3]dioxole)-3a(4*H*)-yl) 2-((4-methoxyphenyl)amino)hexanedioate (8h)

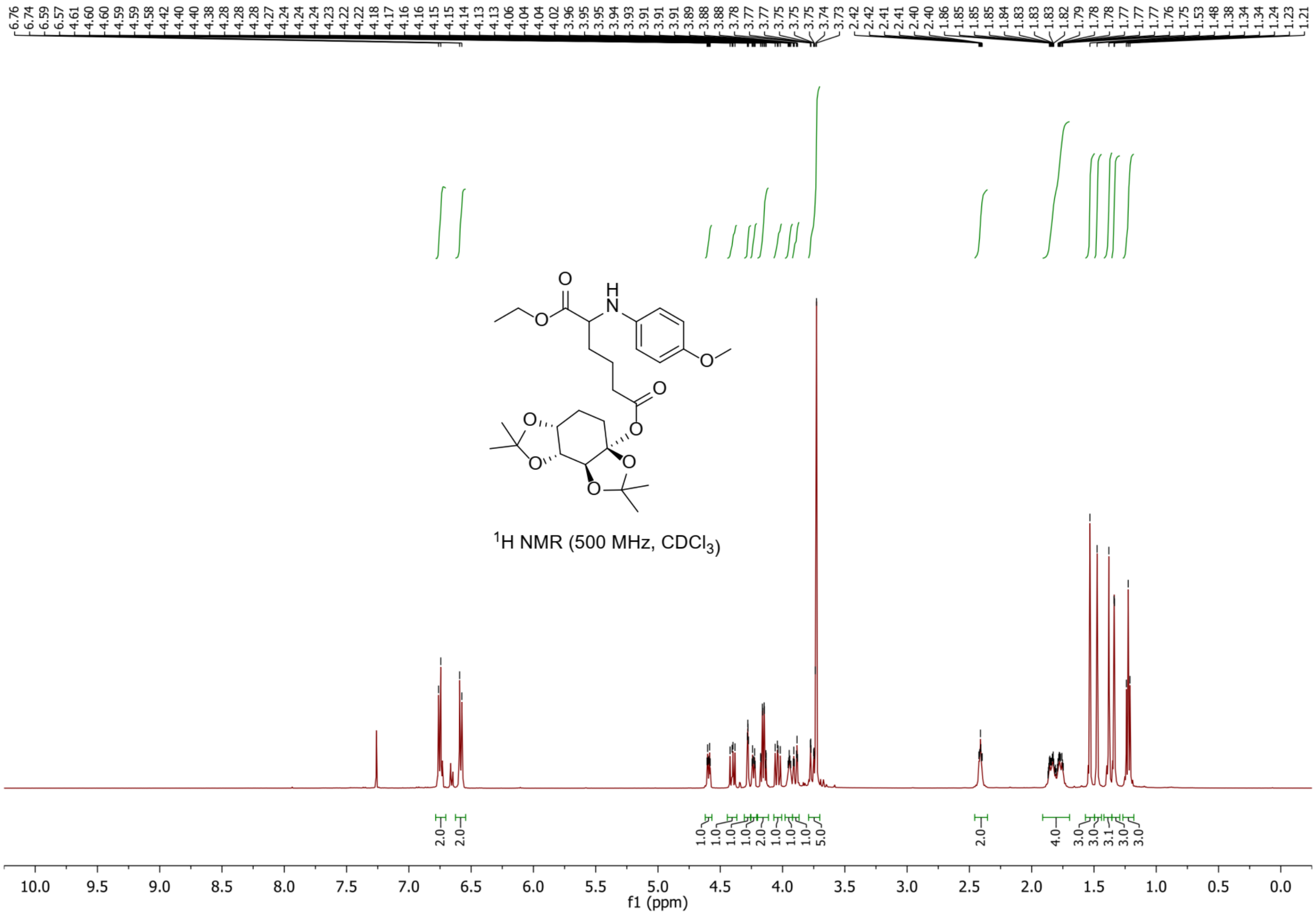

1-Ethyl 6-((3a*S*,5a*R*,8a*R*,8b*S*)-2,2,7,7-tetramethyltetrahydrobenzo[1,2-*d*:3,4-*d'*]bis([1,3]dioxole)-3a(4*H*)-yl) 2-((4-methoxyphenyl)amino)hexanedioate (8h)

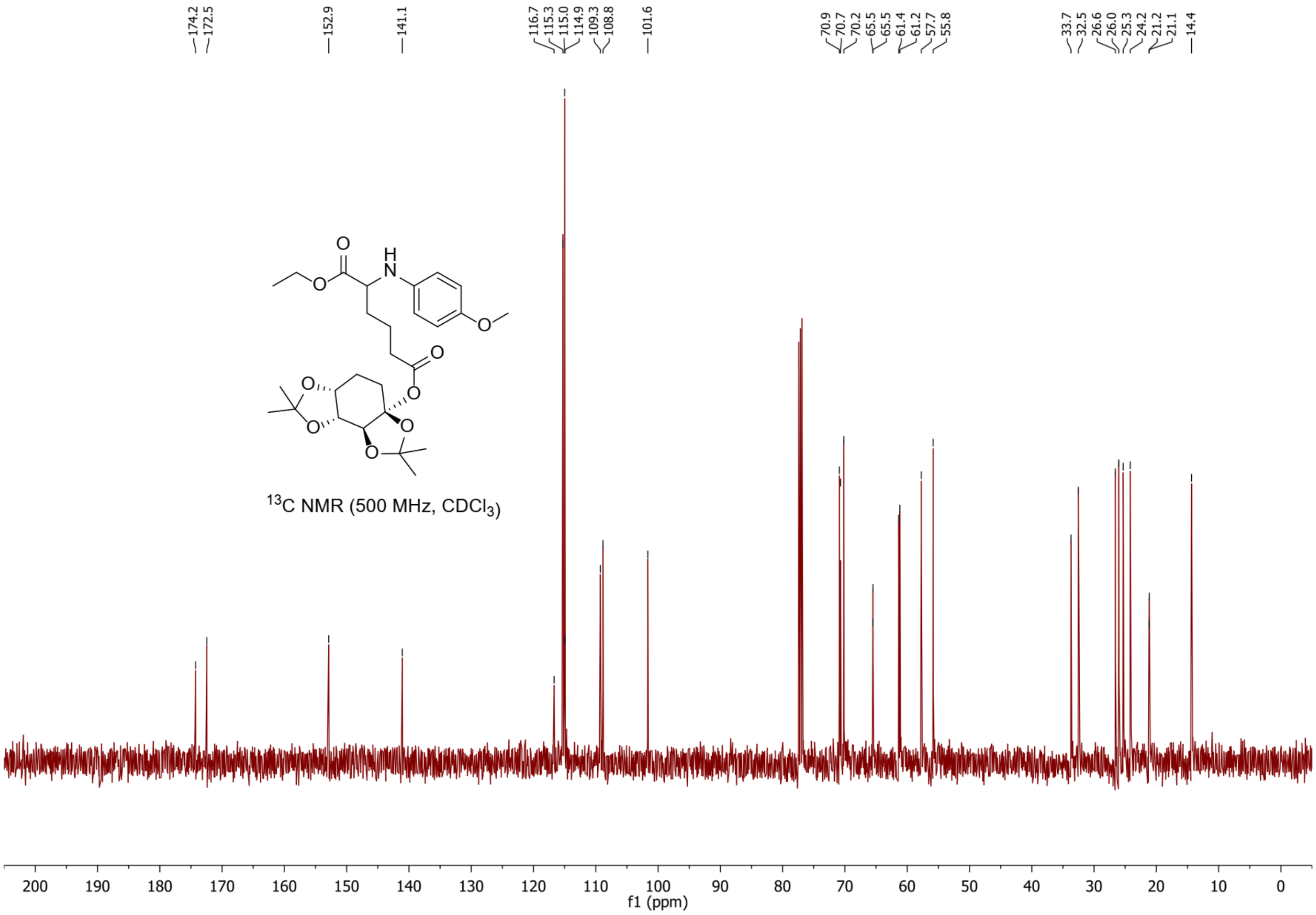

Go to table of contents

References

1. Gaussian 16, Revision A.03, M. J. Frisch, G. W. Trucks, H. B. Schlegel, G. E. Scuseria, M. A. Robb, J. R. Cheeseman, G. Scalmani, V. Barone, G. A. Petersson, H. Nakatsuji, X. Li, M. Caricato, A. V. Marenich, J. Bloino, B. G. Janesko, R. Gomperts, B. Mennucci, H. P. Hratchian, J. V. Ortiz, A. F. Izmaylov, J. L. Sonnenberg, D. Williams-Young, F. Ding, F. Lipparini, F. Egidi, J. Goings, B. Peng, A. Petrone, T. Henderson, D. Ranasinghe, V. G. Zakrzewski, J. Gao, N. Rega, G. Zheng, W. Liang, M. Hada, M. Ehara, K. Toyota, R. Fukuda, J. Hasegawa, M. Ishida, T. Nakajima, Y. Honda, O. Kitao, H. Nakai, T. Vreven, K. Throssell, J. A. Montgomery, Jr., J. E. Peralta, F. Ogliaro, M. J. Bearpark, J. J. Heyd, E. N. Brothers, K. N. Kudin, V. N. Staroverov, T. A. Keith, R. Kobayashi, J. Normand, K. Raghavachari, A. P. Rendell, J. C. Burant, S. S. Iyengar, J. Tomasi, M. Cossi, J. M. Millam, M. Klene, C. Adamo, R. Cammi, J. W. Ochterski, R. L. Martin, K. Morokuma, O. Farkas, J. B. Foresman and D. J. Fox, Gaussian, Inc., Wallingford CT, 2016.

2. R. Z. Khaliullin, E. A. Cobar, R. C. Lochan, A. T. Bell and M. Head-Gordon, *J. Phys. Chem. A* 2007, **111**, 8753–8765.

3. Y. Shao, Z. Gan, E. Epifanovsky, A. T. B. Gilbert, M. Wormit, J. Kussmann, A. W. Lange, A. Behn, J. Deng, X. Feng, D. Ghosh, M. Goldey, P. R. Horn, L. D. Jacobson, I. Kaliman, R. Z. Khaliullin, T. Kuś, A. Landau, J. Liu, E. I. Proynov, Y. M. Rhee, R. M. Richard, M. A. Rohrdanz, R. P. Steele, E. J. Sundstrom, H. L. Woodcock, P. M. Zimmerman, D. Zuev, B. Albrecht, E. Alguire, B. Austin, G. J. O. Beran, Y. A. Bernard, E. Berquist, K. Brandhorst, K. B. Bravaya, S. T. Brown, D. Casanova, C.-M. Chang, Y. Chen, S. H. Chien, K. D. Closser, D. L. Crittenden, M. Diedenhofen, R. A. DiStasio, H. Do, A. D. Dutoi, R. G. Edgar, S. Fatehi, L. Fusti-Molnar, A. Ghysels, A. Golubeva-Zadorozhnaya, J. Gomes, M. W. D. Hanson-Heine, P. H. P. Harbach, A. W. Hauser, E. G. Hohenstein, Z. C. Holden, T.-C. Jagau, H. Ji, B. Kaduk, K. Khistyaev, J. Kim, J. Kim, R. A. King, P. Klunzinger, D. Kosenkov, T. Kowalczyk, C. M. Krauter, K. U. Lao, A. D. Laurent, K. V. Lawler, S. V. Levchenko, C. Y. Lin, F. Liu, E. Livshits, R. C. Lochan, A. Luenser, P. Manohar, S. F. Manzer, S.-P. Mao, N. Mardirossian, A. V. Marenich, S. A. Maurer, N. J. Mayhall, E. Neuscamman, C. M. Oana, R. Olivares-Amaya, D. P. O'Neill, J. A. Parkhill, T. M. Perrine, R. Peverati, A. Prociuk, D. R. Rehn, E. Rosta, N. J. Russ, S. M. Sharada, S. Sharma, D. W. Small, A. Sodt, T. Stein, D. Stück, Y.-C. Su, A. J. W. Thom, T. Tsuchimochi, V. Vanovschi, L. Vogt, O. Vydrov, T. Wang, M. A. Watson, J. Wenzel, A. White, C. F. Williams, J. Yang, S. Yeganeh, S. R. Yost, Z.-Q. You, I. Y. Zhang, X. Zhang, Y. Zhao, B. R. Brooks, G. K. L. Chan, D. M. Chipman, C. J. Cramer, W. A. Goddard, M. S. Gordon, W. J. Hehre, A. Klamt, H. F. Schaefer, M. W. Schmidt, C. D. Sherrill, D. G. Truhlar, A. Warshel, X. Xu, A. Aspuru-Guzik, R. Baer, A. T. Bell, N. A. Besley, J.-D. Chai, A. Dreuw, B. D. Dunietz, T. R. Furlani, S. R. Gwaltney, C.-P. Hsu, Y. Jung, J. Kong, D. S. Lambrecht, W. Liang, C. Ochsenfeld, V. A. Rassolov, L. V. Slipchenko, J. E. Subotnik, T. Van Voorhis, J. M. Herbert, A. I. Krylov, P. M. W. Gill, and M. Head-Gordon, *Mol. Phys.* 2015, **113**, 184–215.

4. For access to chemcraft program: https://www.chemcraftprog.com

5. For access to CYLview20 program: http://www.cylview.org

6. For access to visual molecular dynamics program: W. Humphrey, A. Dalke and K. Schulten, VMD - Visual Molecular Dynamics. *J. Molec. Graphics.* 1996, **14**, 33–38

7. NBO 7.0. E. D. Glendening, J. K. Badenhoop, A. E. Reed, J. E. Carpenter, J. A. Bohmann, C. M. Morales, P. Karafiloglou, C. R. Landis and F. Weinhold, Theoretical Chemistry Institute, University of Wisconsin, Madison, 2018.

8. For access to the IboView software: http://iboview.org/_bgBqyRo.html

9. G. Luchini, J. V. Alegre-Requena, Y. Guan, I. Funes-Ardoiz and R. S. Paton, *F1000Research* 2020, **9**, 291.

10. F. M. Bickelhaupt and K. N. Houk, *Angew. Chem. Int. Ed.* 2017, **56**, 10070–10086.

11. For discussion of components obtained via ASM and ALMO-EDA2 calculations, see Supplemental Information for: S. Jin, G. C. Haug, R. Trevino, V. D. Nguyen, H. D. Arman and O. V. Larionov, *Chem. Sci.* 2021, **12**, 13914–13921.

12. E. Ramos-Cordoba, V. Postils and P. Salvador, *J. Chem. Theory Comput.* 2015, **11**, 1501–1508

13. G. Knizia, *J. Chem. Theory Comput.* 2013, **9**, 4834-4843.

14. R. A. Marcus and N. Sutin, *Biochim. Biophys. Acta* 1985, **811**, 265–322.

15 . C. Lefebvre, H. Khatabil, J.-C. Boisson, J. Contreras-Garcia, J.-P. Piquemal and E. Henon, *ChemPhysChem*. 2018, **19**, 724–735.

16 . T. Lu and F. Chen, *J. Comput. Chem.* 2012, **33**, 580-592. For MultiWFN program: http://sobereva.com/multiwfn/
